# Retrieval Augmented Generation (RAG) for Evaluating Regulatory Compliance of Drug Information and Clinical Trial Protocols

**DOI:** 10.1002/psp4.70201

**Published:** 2026-02-19

**Authors:** Shreyas Waikar, Amruta Gajanan Bhat, Murali Ramanathan

**Affiliations:** ^1^ Department of Pharmaceutical Sciences, Artificial Intelligence and Clinical Pharmacology Laboratory University at Buffalo, The State University of New York Buffalo New York USA

**Keywords:** AI, artificial intelligence, clinical pharmacology, LLM, MIDD, pharmacometrics, RAG

## Abstract

The purpose was to evaluate retrieval‐augmented generative (RAG) artificial intelligence (AI) methods for assessing the regulatory compliance of drug information and adherence to best practices in clinical trial protocols. Integrated systems containing RAG and large language model (LLM) components were employed to evaluate drug information and clinical trial protocols. The drug information for adalimumab, insulin glargine, atorvastatin calcium, sertraline, and alprazolam was evaluated for compliance with Food and Drug Administration (FDA) clinical pharmacology guidance for indications, use in specific populations, and warnings and precautions. The reasons for the withdrawal of rofecoxib, valdecoxib, and troglitazone were elicited. The clinical trial protocol evaluation system was used to assess a Phase‐2a clinical trial protocol of Rifafour in tuberculosis with the FDA E9 and E9 (R1) guidance documents. The RAG system correctly identified the indications, use in specific populations, and warnings and precautions for adalimumab, insulin glargine, atorvastatin calcium, sertraline, and alprazolam. The drug information was evaluated against the requirements in the guidance documents, confirming compliance when present and providing explanations for deficiencies. The causes underlying the withdrawal of rofecoxib, valdecoxib, and troglitazone were explained. The clinical protocol summary included study design, population definitions, treatments, dose levels, and route of administration. The summary of the statistical analysis plan included primary/secondary endpoints, statistical tests, pharmacokinetic parameters, and handling of missing data and outliers. The findings aligned with manual protocol reviews. RAG‐based AI methods can improve the usefulness of LLMs in document‐restricted settings and are a promising approach for evaluating the compliance of clinical pharmacology documents.

## Study Highlights

- What is the current knowledge on the topic?
  ○ The technical complexities involved in evaluating drug information and reviewing clinical protocols require multidisciplinary expertise to assess scientific validity, risks, and opportunities, making the process time‐consuming and expertise‐intensive. Large language models (LLMs) offer a transformative opportunity to reshape this workflow.
- What question did this study address?
  ○ The study evaluated retrieval‐augmented generative (RAG) AI methods for assessing the regulatory compliance of drug information and examining adherence to best practices in clinical trial protocols.
- What does this study add to our knowledge?
  ○ The results show that RAG systems improve the utility of LLMs and are a promising approach for diverse clinical pharmacology challenges.
- How might this change drug discovery, development, and/or therapeutics?
  ○ RAG‐based systems may help sponsors systematically review drug documents for compliance gaps before regulatory submission.

## 1 Introduction

The rapid rise of large language models (LLMs) such as ChatGPT, Gemini, Llama, Claude, Grok, and DeepSeek has reshaped an information‐technology landscape already crowded with AI innovations [1, 2, 3, 4, 5, 6]. These advances have stimulated research to apply and adapt LLMs to accelerate drug development [7, 8, 9, 10, 11, 12].

While LLMs are highly effective at integrating external knowledge and allow conversational, natural language interactions with users, they can have billions of parameters and require enormous datasets and high‐end computational resources for training. Although providers periodically update their LLMs with new data, research and clinical applications require a domain‐specialized knowledge base with up‐to‐date information. A key challenge with LLMs is effectively leveraging organizational knowledge and databases.

Lu et al. review the potential applications of LLM in drug discovery, preclinical research, and literature review automation, with several case studies including LLM‐based tools for biomedical queries, regulatory intelligence, and medical writing [13]; Shahin et al. review emerging agentic frameworks [14]. LLMs are also being used in AI‐augmented workflows for quantitative systems pharmacology modeling to improve knowledge integration and enhance reproducibility in rare diseases such as Gaucher's disease [15]. LLMs are also being studied for pharmacovigilance to predict drug‐adverse event associations through text mining of discharge summaries, in the context of pharmacoepidemiological study design [16, 17].

Retrieval‐augmented generation (RAG) is a complementary approach to LLM that allows precise control over the data domain across which training and generation occur [18]. The provenance of RAG outputs can be readily traced to the corresponding source documents, and their findings can be further manipulated. RAGs can harness data from specialized user‐provided information sources, e.g., regulatory documents, and can be used to guide LLM. These features enhance capabilities, data security, and user trust. RAG‐integrated methods have the potential to be engineered to address task‐specific needs and knowledge‐intensive problems in clinical pharmacology and pharmacometrics research.

RAG models have not been extensively investigated in the context of pharmaceutical sciences. Wu et al. reported on a RAG‐based tool called AskFDALabel, which extracts adverse event terms and drug‐induced liver injury and cardiotoxicity from a database of 200 United States Food and Drug Administration (FDA)‐approved package inserts [19].

The objective of this focused pilot study was to evaluate RAG‐based frameworks that go beyond information extraction and allow their use as decision‐making aids in a human‐in‐the‐loop workflow. To evaluate RAG outputs for decision‐making, we assessed their performance in comparing test documents to target regulatory guidance documents. We included metrics of precision and relevance for the generated output.

## 2 Methods

### 2.1 Datasets for Drug Information Evaluation

#### 2.1.1 Drug Information Datasets

We created two datasets to evaluate the RAG system for this pilot study. The first dataset comprised package inserts for five diverse and representative FDA‐approved drugs: adalimumab (HUMIRA, AbbVie), insulin glargine (LANTUS, Sanofi), atorvastatin calcium (LIPITOR, Pfizer), alprazolam (XANAX, Upjohn, Pfizer), and sertraline (ZOLOFT, Pfizer), which are currently marketed. We refer to this dataset as the Approved Dataset.

The second dataset consisted of package inserts and FDA documents for the following drugs: valdecoxib (BEXTRA, Searle), rofecoxib (VIOXX, Merck), and troglitazone (REZULIN, Pfizer), which are exemplars of drugs that were withdrawn from the market or severely restricted by the FDA because of adverse events during clinical use. We refer to this dataset as the Withdrawn Dataset.

#### 2.1.2 FDA Guidance Documents for Drug Information Evaluations

We used the Code of Federal Regulations, Title 21: Food and Drugs, to evaluate the package insert labeling sections for indications, use in specific populations, and warnings and precautions [20]. FDA guidance documents for the indications [21], and warnings and precautions [22] sections were used. For evaluating the use in specific populations section of package inserts, we created a composite binder containing six FDA guidance documents: Ethical Considerations for Clinical Investigations of Medical Products Involving Children [23], E11 (R1) Addendum: Clinical Investigation of Medicinal Products in the Pediatric Population [24], Pregnant Women: Scientific and Ethical Considerations for Inclusion in Clinical Trials [25], Clinical Lactation Studies: Considerations for Study Design [26], Considerations for the Inclusion of Adolescent Patients in Adult Oncology Clinical Trials [27], and Inclusion of Older Adults in Cancer Clinical Trials [28].

The package inserts for the Approved Dataset and the FDA guidance documents are in Data S1.

### 2.2 Datasets for Clinical Trial Protocol Evaluation

#### 2.2.1 Clinical Trial Protocol Datasets

The protocol and statistical analysis plan (SAP) for NCT03557281 “An Early Bactericidal Activity, Safety and Tolerability of GSK3036656 in Subjects with Drug‐Sensitive Pulmonary Tuberculosis,” [29, 30] were downloaded from ClinicalTrials.gov. NCT03557281 is a Phase 2a interventional study of Rifafour (a fixed‐dose combination of the four antituberculosis drugs, rifampicin 150 mg, isoniazid 75 mg, pyrazinamide 400 mg, and ethambutol HCI 275 mg) conducted in Cape Town, South Africa, and sponsored by GlaxoSmithKline (GSK). The protocol and SAP are in the Data S2.

#### 2.2.2 FDA Guidance Documents for Clinical Trial Protocol Evaluations

The E9 Statistical Principles for Clinical Trials [31] and the E9 (R1) Statistical Principles for Clinical Trials: Addendum: Estimands and Sensitivity Analysis in Clinical Trials [32] guidance documents were downloaded from the United States Food and Drug Administration website. These guidance documents provide directions for statistical analyses of clinical trial data across all stages of drug (clinical) development, with a focus on assessing both safety and efficacy. The E9 (R1) addendum [32] introduces estimands and outlines strategies for conducting sensitivity analyses.

### 2.3 Design of the Drug Information Evaluation System

The implementation details for document ingestion and preprocessing, sentence embedding and indexing, query processing, language model generation, model hyperparameters, and output generation are summarized in the Data S3. Sentence embedding transforms text into a numerical vector, and indexing organizes the information to enable efficient retrieval of data relevant to the query.

#### 2.3.1 Queries

Three queries related to the drug indications, use in specific populations, and warnings and precautions sections of the package were submitted individually to the system. The queries were designed to elicit a summary of the relevant facts from the package insert, an evaluation of the compliance with guidance documents, and a critique of the missing elements.

##### 2.3.1.1 Indications Query 1

1. List the indications and usage from the “Indications and Usage” section in “Drug_name.pdf”.

2. Evaluate whether this section complies with the expectations outlined in the guidance documents “Indications.pdf” and “21 CFR 201.57.pdf”. Specifically, assess whether the general principles, content, and format requirements are clearly stated.

3. Identify any missing or inconsistent components in Drug_name.pdf relative to the requirements outlined in the relevant guidance documents. Specify which components are absent, incomplete, or misaligned, and reference the applicable sections of the guidance documents where appropriate.

##### 2.3.1.2 Specific Populations Query 2

1. List the Use in Specific Populations from “Use in Specific Populations” section in “Drug_name.pdf”.

2. Evaluate whether this section complies with the expectations outlined in the guidance documents “UseinSpecificPopulation.pdf” and “21 CFR 201.57.pdf”. Specifically, assess whether the general principles, content, and format requirements are clearly stated.

3. Identify any missing or inconsistent components in Drug_name.pdf relative to the requirements outlined in the relevant guidance documents. Specify which components are absent, incomplete, or misaligned, and reference the applicable sections of the guidance documents where appropriate.

##### 2.3.1.3 Warnings and Precautions Query 3

1. List the Warnings and Precautions from “Warnings and Precautions” section in “Drug_name.pdf”.

2. Evaluate whether this section complies with the expectations outlined in the guidance documents “Warnings&Precautions.pdf” and “21 CFR 201.57.pdf”. Specifically, assess whether the general principles, content, and format requirements are clearly stated.

3. Identify any missing or inconsistent components in ‘Drug_name.pdf’ relative to the requirements outlined in the relevant guidance documents. Specify which components are absent, incomplete, or misaligned, and reference the applicable sections of the guidance documents where appropriate.

#### 2.3.2 Scoring Metrics

Two metrics Recall‐Oriented Understudy for Gisting Evaluation—Longest Common Subsequence (ROUGE‐L) and Metric for Evaluation of Translation with Explicit Ordering (METEOR).

ROUGE‐L evaluates the overlap between a generated and a reference answer using words' longest common subsequence (LCS). It considers content and word order, calculating recall (i.e., how much of the reference was covered by the prediction) and precision metrics. ROUGE‐L ranges from 0 to 1. A value of 0 indicates no overlap of word sequences, while a value of 1 signifies a perfect match. ROUGE‐L is useful for comparing sentence‐level coherence, with scores between 0.1 and 0.7 considered acceptable depending on length and complexity.

METEOR evaluates the alignment between a generated and a reference answer by considering semantic meaning, word order, and linguistic variations. METEOR matches unigrams (individual words) between the candidate and reference answer, where occurrences of exact matches, stemming (e.g., “inject” vs. “injecting”), synonym matching (using WordNet or similar sources), and paraphrase matches are assessed. It computes a harmonic mean of precision and recall and applies a fragmentation penalty to reward fluent, contiguous matches over scattered word overlap. METEOR, unlike ROUGE‐L, considers synonyms, stems, and paraphrases, which makes it more effective at capturing partial matches and variations in phrasing. METEOR ranges from 0 to 1. A value of 0 indicates no meaningful overlap, while a value of 1 signifies a perfect semantic and lexical match. In practice, METEOR scores range from 0.1 to 0.6 for good answers in question‐and‐answer and summarization tasks (e.g., 0.3–0.5 often indicates moderate to high relevance).

### 2.4 Design of the Clinical Trial Protocol Evaluation System

The document ingestion and preprocessing, sentence embedding and indexing, query processing, prompt construction, language model generation, model hyperparameters, web augmentation, and output management are summarized in the Data S3.

#### 2.4.1 Queries

Two queries were submitted to the LLM. Query 1: “Extract and summarize the clinical trial protocol and statistical analysis plan of the given clinical trial.” Query 2: “Based on the FDA E9 Statistical Principles for Clinical Trials, evaluate the statistical analysis plan of the clinical trial protocol.”

#### 2.4.2 Scoring Metrics

We used Deepeval [33], an open‐source framework for evaluating LLM responses to tasks from RAGs, agents, and chatbots. We employed three metrics: answer relevancy, faithfulness, and contextual relevancy to clinical pharmacology (ClinPharm metric).

Answer Relevancy assesses whether the LLM's response effectively and succinctly addresses the user query. Faithfulness evaluates the factual accuracy of the generated output against the ground truth. Contextual Relevancy measures how well the retrieved context aligns with the query.

The ClinPharm metric was constructed with GEval, a flexible evaluation framework within DeepEval [34] that uses an LLM‐as‐a‐judge approach with a chain‐of‐thought process to assess generated outputs with human‐like accuracy. The metric was defined to identify contradictions to the principles of clinical pharmacology and to penalize omissions, using the following steps: “Check whether the facts in ‘actual output’ contradicts clinical pharmacology principles”, “You should penalize omission of detail”, and “Check whether the facts in ‘actual output’ are relevant to drug development”. PEARL's responses were evaluated using *gpt‐4o* as the judge model.

### 2.5 Independent Verification

The Drug Information and Clinical Trial Protocol evaluation results were verified by authors S.W. and A.G.B. The Missing or Inconsistent Components responses corresponding to Indications, Use in Specific Populations, and Warnings and Precautions package label sections of HUMIRA and LIPITOR were manually checked against the source documents. The clinical trial protocol comments (Query 2) were compared with the FDA E9 guideline.

### 2.6 Prompt Variants

The impact of variations in prompt design on the RAG system outcomes for drug information and clinical trial protocol evaluations was examined for HUMIRA and LIPITOR. Two prompt features were altered: the persona and the inclusion of a fact‐checklist to summarize key points. The methods for the prompt variant experiments are in Data S4.

### 2.7 Comparison to GPT‐4o

We compared the RAG system directly to the GPT‐4o model querying without retrieval or a rule‐based search to contextualize our findings and assess the value of the RAG architecture. Queries were submitted without specific regulatory guidance documents.

Each drug in the Approved Dataset was assessed using a single query. The Indications Query 1 was utilized for LIPITOR and XANAX, the Specific Populations Query 2 was utilized for LANTUS, the Warnings and Precautions Query 3 was utilized for HUMIRA and ZOLOFT. The Withdrawn Dataset was assessed in the context of the web augmentation call to GPT‐4o from the RAG.

The two Clinical Trial Protocol queries were similarly submitted without specific regulatory guidance documents.

## 3 Results

### 3.1 Drug Characteristics

The drugs in the Approved and Withdrawn datasets are summarized in Table 1.

**TABLE 1:** Pharmacological characteristics of drugs in the approved and withdrawn datasets.

Approved drugs		
Drug (brand name)	Mechanism of action	Indication
Insulin glargine (LANTUS)	Reduces blood glucose	Diabetes
Atorvastatin calcium (LIPITOR)	HMG‐CoA inhibitor, reduces cholesterol synthesis	Hypercholesterolemia
Alprazolam (XANAX)	Benzodiazepine	Anxiety
Adalimumab (HUMIRA)	Inhibits TNF‐alpha	Arthritis, psoriasis, IBD, etc.
Sertraline (ZOLOFT)	Serotonin reuptake inhibitor	Depression

Withdrawn drugs			
Drug (trade name)	Mechanism of action	Indication	Reason for withdrawal
Valdecoxib (BEXTRA)	COX‐2 inhibitor, NSAID	Arthritis, pain	Skin reactions, Stevens‐Johnson syndrome, risk of cardiovascular diseases
Rofecoxib (VIOXX)	COX‐2 inhibitor, NSAID	Arthritis, pain	Risk of cardiovascular diseases
Troglitazone (REZULIN)	Thiazolidinedione	Type II diabetes	Hepatic toxicity

Abbreviations: COX‐2, cyclooxygenase‐2; HMG‐CoA, 3‐hydroxy‐3‐methylglutaryl‐coenzyme A reductase; IBD, inflammatory bowel disease; NSAID, non‐steroid anti‐inflammatory drug; TNF‐alpha, tumor necrosis factor alpha.

The five drugs in the Approved Dataset were selected to represent different therapeutic classes and disease indications. Insulin glargine (LANTUS) is a long‐acting, synthetic insulin that regulates blood glucose levels and is used to treat diabetes. Atorvastatin (LIPITOR) is a 3‐hydroxy‐3‐methylglutaryl‐coenzyme A reductase (HMG‐CoA) enzyme inhibitor that is used to treat hypercholesterolemia and lowers the risk of cardiovascular events. Adalimumab (HUMIRA) is an immunosuppressive monoclonal antibody that blocks tumor necrosis factor‐alpha and is used to treat various inflammatory diseases, including arthritis, psoriasis, and inflammatory bowel disease. Alprazolam (XANAX) is a benzodiazepine that is used to treat anxiety. Sertraline (ZOLOFT) is a selective serotonin reuptake inhibitor that is used to treat depression.

The Withdrawn Dataset consisted of two cyclooxygenase‐2 (COX‐2) enzyme inhibitor analgesics, rofecoxib and valdecoxib, and troglitazone, which were withdrawn by the FDA due to adverse events during clinical use. Rofecoxib was removed from the market in September 2004 and is one of the most widely used drugs ever to be withdrawn. Valdecoxib was withdrawn in April 2005. Troglitazone, approved for treating Type 2 diabetes, was withdrawn in March 2000.

The contrastive nature of the Approved and Withdrawn datasets provides a framework for evaluating the RAG system's performance relative to the FDA Regulatory Guidance document set.

### 3.2 Description of the RAG Systems

**FIGURE 1:**
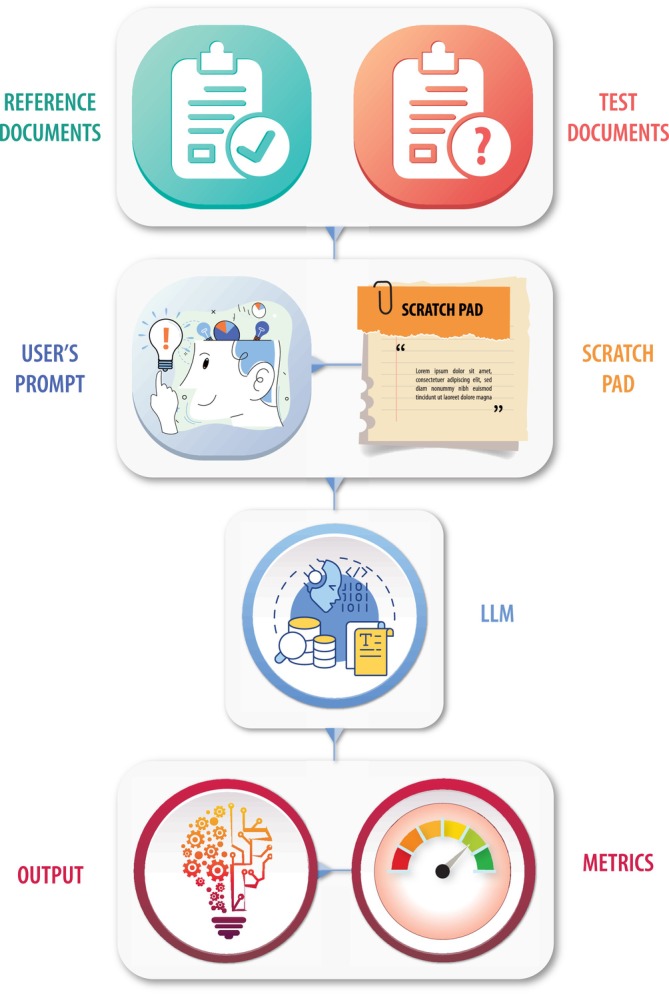
Schematic of the retrieval‐augmented generation (RAG) model. The system comprises natural language processing (NLP), RAG, and large language model (LLM) components that interact with each other. The NLP component preprocesses the reference FDA guidance documents, the test documents, the scratchpad, and the user query. The retrieved context is input to the LLM to obtain output.

Figure 1 is a schematic of the RAG system for drug information and clinical trial protocol evaluations, which was implemented using the LangChain platform. The system comprises natural language processing (NLP), RAG, and large language model (LLM) components that interact with each other. The NLP component preprocesses the guidance and test documents. The RAG retrieves the context from the documents that are relevant to the query. The prompt is engineered by combining persona, context, scratchpad, and the user query. The prompt is input to the LLM to obtain output. The Streamlit framework provides the user interface for interacting with the RAG system.

### 3.3 Drug Information Evaluation

**FIGURE 2:**
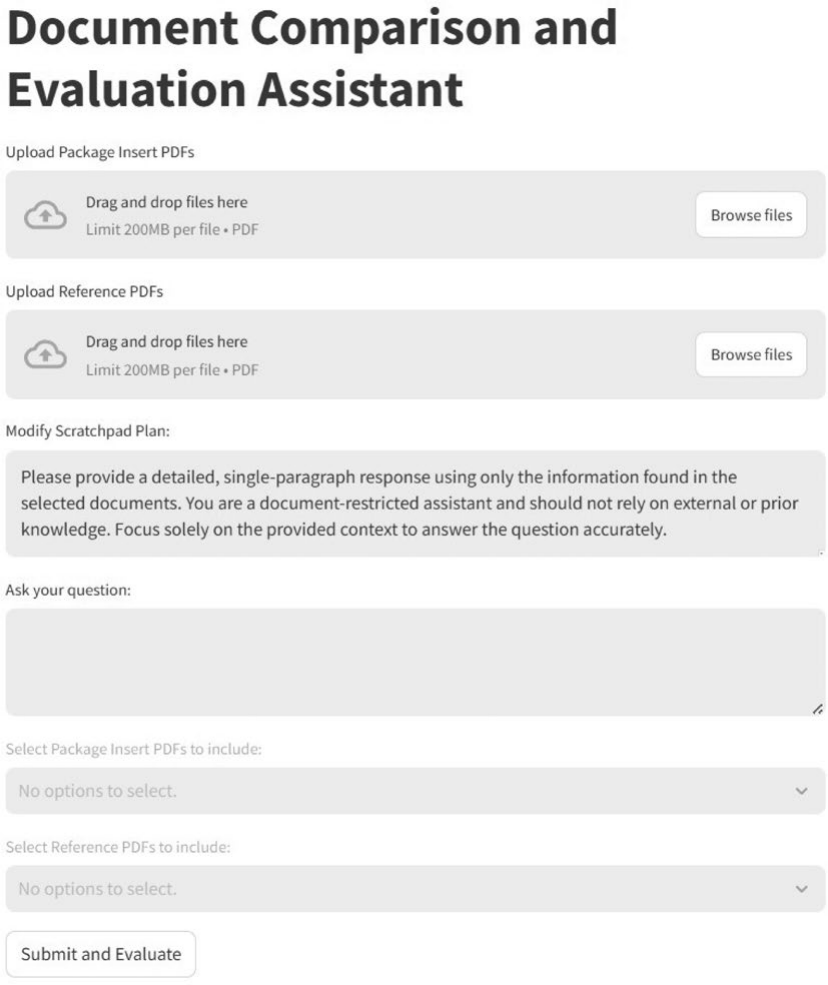
Screenshot from the retrieval‐augmented generation (RAG) model implementation for drug package insert evaluation experiments.

Figure 2 shows the components of the RAG system user interface for the drug package insert evaluation. The user‐friendly interface is organized into sections for package inserts, guidance documents, a scratchpad, and a query.

Tables 2, 3, 4 present the prompts and ChatGPT responses used to assess the indications, use in specific populations, and warnings and precautions sections of the package insert labeling, as per FDA guidelines for adalimumab. The results for insulin glargine, atorvastatin calcium, sertraline, and alprazolam are summarized in Table S1 in Data S3.

**TABLE 2:** Results from the RAG system for the indications section of the adalimumab package insert against the FDA guidance.

*Query for Guidance‐1 Indications*: 1. List the indications and usage from “Indications and Usage” section in “Humira.pdf”. 2. Evaluate whether this section complies with the expectations outlined in the guidance documents “Indications.pdf” and “21 CFR 201.57.pdf”. Specifically, assess whether the general principles, content, and format requirements are clearly stated. 3. Identify any missing or inconsistent components in ‘Humira.pdf’ relative to the requirements outlined in the relevant guidance documents. Specify which components are absent, incomplete, or misaligned, and reference the applicable sections of the guidance documents where appropriate.
*Response*: The “Indications and Usage” section in “Humira.pdf” lists the following indications: Rheumatoid Arthritis (RA): Reducing signs and symptoms, inducing major clinical response, inhibiting the progression of structural damage, and improving physical function in adult patients with moderately to severely active RA. Juvenile Idiopathic Arthritis (JIA): Reducing signs and symptoms of moderately to severely active polyarticular JIA in patients 2 years of age and older. Psoriatic Arthritis (PsA): Reducing signs and symptoms, inhibiting the progression of structural damage, and improving physical function in adult patients with active PsA. Ankylosing Spondylitis (AS): Reducing signs and symptoms in adult patients with active AS. Crohn's Disease (CD): Treatment of moderately to severely active Crohn's disease in adults and pediatric patients 6 years of age and older. Ulcerative Colitis (UC): Treatment of moderately to severely active ulcerative colitis in adults and pediatric patients 5 years of age and older. Plaque Psoriasis (Ps): Treatment of adult patients with moderate to severe chronic plaque psoriasis who are candidates for systemic therapy or phototherapy, and when other systemic therapies are medically less appropriate. Hidradenitis Suppurativa (HS): Treatment of moderate to severe hidradenitis suppurativa in patients 12 years of age and older. Uveitis (UV): Treatment of non‐infectious intermediate, posterior, and panuveitis in adults and pediatric patients 2 years of age and older. The section also includes a “Limitations of Use” statement: “Effectiveness has not been established in patients who have lost response to or were intolerant to TNF blockers.” Compliance Evaluation: General Principles: The “Indications and Usage” section in “Humira.pdf” clearly communicates the drug's approved indications, making it easy for health care practitioners to identify appropriate therapies for patients. This aligns with the general principles outlined in both guidance documents. Content: The section states the diseases or conditions for which Humira is approved, and whether it is indicated for treatment or symptom relief. It also includes a “Limitations of Use” statement. This meets the content requirements from both guidance documents. Format: The section is organized and formatted to be clear and concise. Each indication is listed separately, and the “Limitations of Use” statement is also clearly formatted and listed separately. This meets the format requirements from both guidance documents. Missing or Inconsistent Components: The “Indications and Usage” section in “Humira.pdf” does not explicitly state if Humira is used as an adjunct to a primary mode of therapy, which is a requirement in “21 CFR 201.57.pdf” (Key Requirements for Indications and Usage Sections). The section does not describe if the drug is only safe and effective for certain subgroups, nor does it identify any specific tests necessary for patient selection or monitoring. These are requirements in “21 CFR 201.57.pdf” (Key Requirements for Indications and Usage Sections). The section does not state any specific conditions for long‐term use, which is a requirement in “21 CFR 201.57.pdf” (Key Requirements for Indications and Usage Sections). The section does not include any statements comparing the safety or effectiveness of Humira with other agents for the same indications, which is a requirement in “Indications.pdf” (Content Requirements). The section does not include any information about a common belief about Humira's effectiveness for a certain use, but evidence shows it is ineffective or risky, which is a requirement in “21 CFR 201.57.pdf” (Key Requirements for Indications and Usage Sections).

**TABLE 3:** Results from the RAG system for the use in specific populations section of the adalimumab package insert against the FDA guidance.

*Query for Guidance‐2 Use in Specific Populations*: 1. List the Use in Specific Populations from “Use in Specific Populations” section in “Humira.pdf”. 2. Evaluate whether this section complies with the expectations outlined in the guidance documents “UseinSpecificPopulation.pdf” and “21 CFR 201.57.pdf”. Specifically, assess whether the general principles, content, and format requirements are clearly stated. 3. Identify any missing or inconsistent components in ‘Humira.pdf’ relative to the requirements outlined in the relevant guidance documents. Specify which components are absent, incomplete, or misaligned, and reference the applicable sections of the guidance documents where appropriate.
*Response*: The “Use in Specific Populations” section in “Humira.pdf” includes the following information: 8.1 Pregnancy: Provides information about the risk of using adalimumab during pregnancy, including data from the OTIS/MotherToBaby HUMIRA Pregnancy Registry and an embryo‐fetal perinatal development study conducted in cynomolgus monkeys. It also discusses the potential impact of adalimumab on the immune response of the in utero exposed infant. 8.2 Lactation: Discusses the presence of adalimumab in human milk and the potential effects on the breastfed infant. 8.4 Pediatric Use: Details the safety and effectiveness of HUMIRA for various conditions in pediatric patients of different age groups. It also mentions the potential impact of HUMIRA on the immune response of infants exposed in utero. 8.5 Geriatric Use: Discusses the use of HUMIRA in patients 65 years of age and older, including the frequency of serious infection and malignancy in this population. Compliance Evaluation: General Principles: The section complies with the general principles outlined in “21 CFR 201.57.pdf” as it provides information about the use of the drug in specific populations, including pregnant women, lactating women, pediatric patients, and geriatric patients. However, it does not fully comply with the principles outlined in “UseinSpecificPopulation.pdf” as it does not explicitly mention the ethical considerations for clinical investigations involving children. Content Requirements: The section meets the content requirements of “21 CFR 201.57.pdf” by providing information about the drug's use in specific populations, including its dosage, contraindications, warnings and precautions, and adverse reactions. However, it does not fully meet the content requirements of “UseinSpecificPopulation.pdf” as it does not explicitly mention provisions for soliciting the assent of children and the permission of their parents or guardians. Format Requirements: The section complies with the format requirements of “21 CFR 201.57.pdf” by including the required headings and subheadings and presenting the information in the required order. Missing or Inconsistent Components: The section does not explicitly mention the ethical considerations for clinical investigations involving children, which is a requirement outlined in “UseinSpecificPopulation.pdf” (General Principles). The section does not explicitly mention provisions for soliciting the assent of children and the permission of their parents or guardians, which is a requirement outlined in “UseinSpecificPopulation.pdf” (Content Requirements). While the section provides information about the use of the drug in specific populations, it does not provide specific information about the use of the drug as an adjunct to a primary mode of therapy, which is a requirement outlined in “21 CFR 201.57.pdf” (Key Requirements for Indications and Usage Sections)

**TABLE 4:** Results from the RAG system for the warnings and precautions section of the adalimumab package insert against the FDA guidance document.

*Query for Guidance 3 Warnings and Precautions*: 1. List the Warnings and Precautions from “Warnings and Precautions” section in “Humira.pdf”. 2. Evaluate whether this section complies with the expectations outlined in the guidance documents “Warnings&Precautions.pdf” and “21 CFR 201.57.pdf”. Specifically, assess whether the general principles, content, and format requirements are clearly stated. 3. Identify any missing or inconsistent components in ‘Humira.pdf’ relative to the requirements outlined in the relevant guidance documents. Specify which components are absent, incomplete, or misaligned, and reference the applicable sections of the guidance documents where appropriate.
*Response*: Warnings and Precautions from Humira.pdf: Humira should not be started during an active infection. Serious infections, invasive fungal infections, malignancies, anaphylaxis, hepatitis B virus reactivation, demyelinating disease, cytopenias, heart failure, and lupus‐like syndrome are potential risks. Patients should be monitored for active tuberculosis and tested for latent infection before and during therapy. Compliance Evaluation: General Principles: The Warnings and Precautions section in Humira.pdf does not explicitly violate any general principles outlined in the guidance documents. The FDA guidance documents do not establish legally enforceable responsibilities but provide the Agency's current thinking on a topic. The use of the word “should” in Agency guidances means that something is suggested or recommended, but not required. Content Requirements: The Warnings and Precautions section in Humira.pdf includes serious adverse reactions and potential safety hazards, which aligns with the content requirements from the guidance documents. However, it does not include information on any clinically significant interference with a laboratory test and drug interactions as required by the “Warnings&Precautions.pdf” guidance. Format Requirements: The Warnings and Precautions section in Humira.pdf is organized in a clear and consistent manner, which aligns with the format requirements from the guidance documents. However, it does not use individual subsections to organize the information, nor does it use cross‐referencing or emphasis in text to highlight important information as suggested by the “Warnings&Precautions.pdf” guidance. Missing or Inconsistent Components: The Warnings and Precautions section in Humira.pdf does not include information on any clinically significant interference with a laboratory test and drug interactions, which is a requirement according to the “Warnings&Precautions.pdf” guidance (Content Requirements). The section does not use individual subsections to organize the information, nor does it use cross‐referencing or emphasis in text to highlight important information, which is suggested by the “Warnings&Precautions.pdf” guidance (Format Requirements).

The RAG system correctly identified the multiple indications for adalimumab (Table 2) and sertraline, as well as the fewer indications for insulin glargine, atorvastatin calcium, and alprazolam (Table S1 and Data S3). The RAG system rigorously critiqued each package insert against the individual requirements in the guidance. Importantly, it recognized instances where the compliance was satisfactory and provided explanations for each deficiency. Interestingly, the response to the Use in Specific Populations query for adalimumab, insulin glargine, and sertraline highlighted the ethical requirements for participant assent and parental consent, which are required in pediatric clinical trials, but are not a part of labeling requirements.

Table S2 (Data S3) summarizes the evaluation of withdrawn drugs. The focus here was to understand why the drugs were withdrawn. The results from the RAG system were also compared to ChatGPT's answers. The answers provided by the RAG system were generally concordant with the ChatGPT outputs but contained more clinical details. The ROUGE‐L and METEOR scores, shown as percentages in Table S2, are in the acceptable range.

The RAG response correctly noted that rofecoxib was withdrawn because of the increased risk of cardiovascular disease, whereas another COX‐2 inhibitor, valdecoxib, was withdrawn because of the increased risk of cardiovascular disease and life‐threatening skin reactions. Valdecoxib was linked to deaths due to Stevens‐Johnson syndrome. Troglitazone, a thiazolidinedione drug for Type 2 diabetes, was withdrawn due to hepatic failure.

### 3.4 Clinical Trial Protocol Evaluation

NCT03557281 was an interventional study that evaluated the early bactericidal activity, safety, and tolerability of GSK3036656 in patients with drug‐sensitive pulmonary tuberculosis.

**FIGURE 3:**
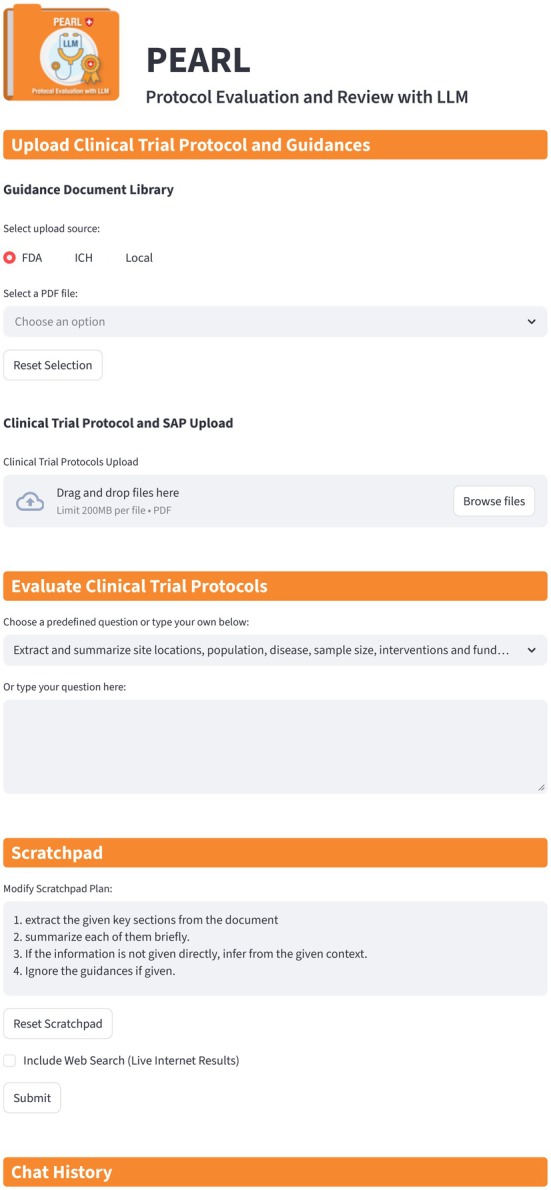
Screenshot of PEARL (Protocol Evaluation and Review with LLM). The application consists of four main components: Upload Clinical Trial Protocol and Guidance, which allows users to upload guidance documents, clinical trial protocols, and SAPs; Evaluate Clinical Trial Protocol, which provides a set of predefined queries that also serve as examples to help users formulate their own questions; Scratchpad, which enables users to guide the LLM's reasoning process by providing additional instructions; and Chat History, where conversations with the LLM are stored. Once the Submit button is clicked, the LLM‐generated response is displayed and appended to the Chat History, which can then be exported as a PDF.

Figure 3 is a schematic of the RAG system for clinical trial protocol evaluation. The RAG system's response to Query 1: “Extract and summarize the clinical trial protocol and statistical analysis plan of the given clinical trial” is in Data S2.

The protocol summary included study design, population definitions, treatments, dose levels, unit dose strength, route of administration, and protocol amendments. It correctly noted that the number of participants per treatment arm was revised from 20 to a range of 12–20, with the study comprising up to five cohorts. Within each cohort, 9–15 participants are assigned to receive GSK3036656 and 3–5 participants to the standard‐of‐care (SoC) regimen for drug‐sensitive tuberculosis (DS‐TB), following a 3:1 randomization ratio. While the summary indicated that the primary objective is to assess the early bactericidal activity of GSK3036656, it failed to also extract the primary endpoint as *Rate of change in log10 CFU sputum samples over the period baseline to Day 14*.

The SAP summary outlined statistical methods, primary and secondary endpoint analyses, pharmacokinetic parameters, handling of missing data and outliers, and additional considerations such as the impact of COVID‐19, protocol deviation management, regulatory compliance, and data handling. Its primary objective to evaluate the early bacterial activity of GSK3036656 was identified. It also noted that safety data would be presented in tabular and/or graphical formats, and that adverse events would be reported using the MedDRA coding system. Answer relevancy and faithfulness were rated at 100% and 95%, respectively, indicating that the response was complete and mostly grounded in the uploaded documents. The ClinPharm metric was 88%, reflecting the omission of minor details.

For Query 2: “Based on the FDA E9 Statistical Principles for Clinical Trials, evaluate the statistical analysis plan of the clinical trial protocol”, the RAG system generated feedback categorized by importance as high, medium, or low while assessing the SAP against the E9 guideline criteria (see Data S2). The evaluation covered key elements such as sample size determination, statistical analysis methods, population definitions, safety data analysis, the reporting and analysis plan, microbiological efficacy/activity assessments, vital signs, and assessment of causality. The RAG system highlighted that the method for sample size re‐estimation was not detailed, which limits reproducibility and clarity. In the pharmacokinetic section, the RAG system noted that the potential effect of body mass index (BMI) as a covariate on results was not addressed. It also recommended incorporating key minimum inhibitory concentration (MIC) findings into the main report to provide a more comprehensive picture of study outcomes.

The mention of handling abnormal vital signs was considered irrelevant for SAP evaluation, bringing the accuracy score to 85.7%. Faithfulness remained at 100%, showing that the response was grounded in the uploaded documents. The ClinPharm metric was lower at 65.9%, mainly due to missing critical details on statistical methods for dose escalation and how protocol deviations were handled.

### 3.5 Independent Verification

The results of the independent verification of the Missing or Inconsistent Components responses corresponding to Indications, Use in Specific Populations, and Warnings and Precautions package label sections of HUMIRA and LIPITOR are summarized in Data S4. The independent verification assessments, which are indicated in the shaded areas of Tables S1 and S2 (Data S4), were mixed and had some errors.

Independent verification of the protocol evaluation comments confirmed that there were no conflicts with the FDA E9 guidance.

### 3.6 Effect of Prompt Variants

The results for the two prompt variants in the context of the Approved Dataset and the Clinical Trial Protocol evaluations are in Data S5.

### 3.7 Comparison to GPT‐4o

We compared the RAG system directly to GPT‐4o model querying, without retrieval or a rule‐based search, to contextualize our findings and assess the value, if any, of the RAG architecture.

#### 3.7.1 Drug Information Evaluation

Data S6 summarizes the GPT‐4o results for evaluation of the Approved Drug data set. Table S2 (Data S3) compares the RAG and GPT‐4o evaluations of withdrawn drugs.

#### 3.7.2 Clinical Trial Protocol Evaluation

Data S6 summarizes the evaluation of the Clinical Trial Protocol with GPT‐4o. The protocol summary generated by the RAG‐based clinical trial evaluation system (Query 1) was largely concordant with that produced by ChatGPT‐4o. ChatGPT‐4o provided concise bullet‐point results, while the RAG system produced short, structured paragraphs as prompted.

ChatGPT‐4o drew on a wider range of regulatory documents, including the EMA Reflection Paper, FDA and EMA PK guidance, adaptive design guidance, and ICH E3/E9—whereas the RAG system relied solely on FDA E9. For Query 2, ChatGPT‐4o identified multiplicity in secondary endpoints as a key deficiency and correctly pointed to the missing‐data section in Appendix 7 (Data S2), which the RAG system did not highlight.

ChatGPT‐4o included a “Final Verdict” and a compliance table, while the RAG system provided more granular feedback, detailing E9‐aligned sections and specific gaps. ChatGPT‐4o highlighted seven compliant items and one partially compliant item (multiplicity), while the RAG system provided a structured set of improvement recommendations.

Overall, ChatGPT‐4o's assessment was more descriptive and less critical, whereas the RAG‐based evaluation emphasized actionable guidance for improvement and decision‐making.

## 4 Discussion

In this pilot study, we evaluated integrated RAG–LLM systems. One system assessed how well drug package inserts and clinical trial protocols complied with regulatory guidance, while the other evaluated protocol compliance with best‐practice recommendations. We examined how closely the documents aligned with the semantic intent of these guidance and best‐practice standards.

A key limitation is that we could assess only a few withdrawn drugs, since many were voluntarily withdrawn and lacked sufficient unbiased FDA documentation on the clinical rationale. We also lacked information on notable drug withdrawals, e.g., terfenadine and fenfluramine, which occurred before the widespread availability of FDA resources and government information online. Terfenadine, a non‐sedating histamine blocker that was approved for treating allergies, was withdrawn from the market in 1997 due to the occurrence of torsades de pointes, a fatal cardiac arrhythmia associated with prolongation of the QT interval [35, 36, 37]. Fenfluramine, a serotonin and norepinephrine‐releasing agent that suppresses appetite, was approved to treat obesity, but was withdrawn in September 1997 because of increased valvular heart disease [38]. Likewise, we could not include cerivastatin (BAYCOL), a hydroxymethylglutaryl‐coenzyme A reductase (HMG‐CoA) inhibitor that blocks cholesterol synthesis for the treatment of hypercholesterolemia, which was withdrawn due to the risk of rhabdomyolysis and kidney failure. We did not have the FDA withdrawal documents, but only the Federal Register announcement of the withdrawal (Federal Register Vol. 82, No. 159).

While we evaluated RAGs in the context of regulatory documents and clinical trial protocol tasks, the approach might also be useful for other drug development applications. For example, it could be used in trial design to compare clinical endpoints, inclusion/exclusion criteria, dosing regimens, and outcomes with other drugs for the same indication, or in the same therapeutic class. In the preclinical stages, RAGs could be used to summarize the bioinformatics of drug targets, their signaling pathways, disease implications, and possible off‐target interactions. Our approach may also enable sponsors to check documents for compliance within the context of AI‐enabled drug evaluation processes at the FDA, which now include the recently launched ELSA, a secure LLM‐based tool for scientific evaluations and clinical protocol reviews [39].

Although LLMs are trained on broadly sourced, uncurated datasets compiled from the internet, they are nonetheless effective at solving nuanced research problems in specialized fields such as pharmacometrics and clinical pharmacology. It is useful to compare the strengths and weaknesses of RAG versus LLMs. RAG can integrate information from diverse, user‐relevant datasets and document collections without retraining the underlying model, helping overcome challenges in leveraging organizational knowledge. Although the two RAG tools require further validation, this proof‐of‐concept study shows promise for document‐restricted applications. However, modest technological advances in commercial LLMs may narrow RAG's competitive advantages.

We built separate RAG systems for drug information review and clinical trial protocol evaluation for this pilot demonstration. This modular design enables task‐specific interfaces and prompts. The two tasks differed markedly: package inserts are highly structured and standardized for patient and pharmacist use, and extracting consistent information from them requires careful attention to detail. In contrast, protocol evaluation relies on technical biostatistical expertise.

We found it necessary to address the multimodality of content organization, which refers to the information presented in tables, bulleted and numbered lists, and graphs, using specialized NLP packages, to improve the results from the RAG implementation. However, the PK analyses for Rifafour were organized in a table within a table see Data S2 (Page 188 of PDF), and the pharmacodynamics were in a graph (Data S2, Page 215), which we did not address. PK analysis and other advanced pharmacometric applications may require tools to extract content from graphs and equations. Furthermore, we note the guidance documents (ICH E9 and E9 (R1)) used for SAP evaluation are fit‐for‐purpose for statistical evaluation, but do not address other aspects of protocol compliance, such as pharmacokinetic (PK) analyses.

In conclusion, RAG‐based systems may enable product sponsors to systematically review drug information documents, such as package inserts, for compliance weaknesses before submission to regulatory agencies. Additional validation studies across broader datasets and therapeutic areas are vital to unlock its drug‐development potential. However, advances in LLMs and agentic AI may narrow the range of application areas where RAG systems retain a competitive advantage.

## Author Contributions

S.W., A.G.B., and M.R. wrote the manuscript. M.R. designed the research. S.W. and A.G.B. performed the research. S.W., A.G.B., and M.R. analyzed the data.

## Funding

This work was funded by INV‐080729 from the Design, Analyze, Communicate Integrated Development Global Health Division of the Gates Foundation. The funders had no role in the study's design or data analysis.

## Conflicts of Interest

The authors declare no conflicts of interest.

## Supporting information


**Data S1:** psp470201‐sup‐0001‐Supinfo.zip.
