# Supplementary material for: Retrieval Augmented Generation (RAG) for Evaluating Regulatory Compliance of Drug Information and Clinical Trial Protocols
Source: CPT Pharmacometrics Syst Pharmacol. 2026 Feb 19;15(3):e70201. doi: 10.1002/psp4.70201 (PMC12917324; doi:10.1002/psp4.70201)
Supplement: Supplementary file 1 — Data S1: psp470201‐sup‐0001‐Supinfo.zip. [file PSP4-15-e70201-s001.zip › PSP-2025-0274-s01.pdf]

SUPPLEMENTARY FILE S1

PACKAGE INSERTS & FDA GUIDANCE DOCUMENTS

Retrieval Augmented Generation (RAG) for Evaluating Compliance of Drug Information to FDA Regulatory Guidance Documents

Shreyas Waikar, Amruta Gajanan Bhat, and Murali Ramanathan
Artificial Intelligence & Clinical Pharmacology Laboratory, Department of Pharmaceutical Sciences, University at Buffalo, The State University of New York, Buffalo, NY, USA.

CORRESPONDING AUTHOR: Murali Ramanathan
355 Pharmacy, Department of Pharmaceutical Sciences
University at Buffalo, Buffalo, NY 14214-8033.
(716)-645-4846 and FAX 716-829-6569. E-mail Murali@Buffalo.Edu

Running Head: RAGs for Drug Development
Keywords: Artificial Intelligence, AI, LLM, RAG, Pharmacometrics, MIDD, Clinical Pharmacology

PACKAGE INSERTS

HUMIRA

HUMIRA- adalimumab
Cordavis Limited

HIGHLIGHTS OF PRESCRIBING INFORMATION
These highlights do not include all the information needed to use HUMIRA safely and effectively. See full prescribing information for HUMIRA.

HUMIRA® (adalimumab) injection, for subcutaneous use
Initial U.S. Approval: 2002

WARNING: SERIOUS INFECTIONS AND MALIGNANCY

See full prescribing information for complete boxed warning.

SERIOUS INFECTIONS (5.1**,** 6.1**):**

- **Increased risk of serious infections leading to hospitalization or death, including tuberculosis (TB), bacterial sepsis, invasive fungal infections (such as histoplasmosis), and infections due to other opportunistic pathogens.**
- **Discontinue HUMIRA if a patient develops a serious infection or sepsis during treatment.**
- **Perform test for latent TB; if positive, start treatment for TB prior to starting HUMIRA.**
- **Monitor all patients for active TB during treatment, even if initial latent TB test is negative.**

MALIGNANCY (5.2**):**

- **Lymphoma and other malignancies, some fatal, have been reported in children and adolescent patients treated with TNF blockers including HUMIRA.**
- **Post-marketing cases of hepatosplenic T-cell lymphoma (HSTCL), a rare type of T-cell lymphoma, have occurred in adolescent and young adults with inflammatory bowel disease treated with TNF blockers including HUMIRA.**

INDICATIONS AND USAGE

HUMIRA is a tumor necrosis factor (TNF) blocker indicated for:

- **Rheumatoid Arthritis (RA)** (1.1): reducing signs and symptoms, inducing major clinical response, inhibiting the progression of structural damage, and improving physical function in adult patients with moderately to severely active RA.
- **Juvenile Idiopathic Arthritis (JIA) (**1.2**)**: reducing signs and symptoms of moderately to severely active polyarticular JIA in patients 2 years of age and older.
- **Psoriatic Arthritis (PsA) (**1.3**)**: reducing signs and symptoms, inhibiting the progression of structural damage, and improving physical function in adult patients with active PsA.
- **Ankylosing Spondylitis (AS) (**1.4**)**: reducing signs and symptoms in adult patients with active AS.
- **Crohn's Disease (CD) (**1.5**)**: treatment of moderately to severely active Crohn's disease in adults and pediatric patients 6 years of age and older.
- **Ulcerative Colitis (UC) (**1.6**)**: treatment of moderately to severely active ulcerative colitis in adults and pediatric patients 5 years of age and older.
  Limitations of Use: Effectiveness has not been established in patients who have lost response to or were intolerant to TNF blockers.
- **Plaque Psoriasis (Ps) (**1.7**)**: treatment of adult patients with moderate to severe chronic plaque psoriasis who are candidates for systemic therapy or phototherapy, and when other systemic therapies are medically less appropriate.
- **Hidradenitis Suppurativa (HS) (**1.8**)**: treatment of moderate to severe hidradenitis suppurativa in patients 12 years of age and older.
- **Uveitis (UV) (**1.9**)**: treatment of non-infectious intermediate, posterior, and panuveitis in adults and pediatric patients 2 years of age and older.

DOSAGE AND ADMINISTRATION

- Administer by subcutaneous injection (2)

Rheumatoid Arthritis, Psoriatic Arthritis, Ankylosing Spondylitis (2.1**):**

- *Adults:* 40 mg every other week.
    - Some patients with RA not receiving methotrexate may benefit from increasing the dosage to 40 mg every week or 80 mg every other week.

Juvenile Idiopathic Arthritis or Pediatric Uveitis (2.2**):**

Pediatric Weight 2 Years of Age and Older	Recommended Dosage
10 kg (22 lbs) to less than 15 kg (33 lbs)	10 mg every other week
15 kg (33 lbs) to less than 30 kg (66 lbs)	20 mg every other week
30 kg (66 lbs) and greater	40 mg every other week

Crohn's Disease (2.3**):**
- *Adults:* 160 mg on Day 1 (given in one day or split over two consecutive days); 80 mg on Day 15; and 40 mg every other week starting on Day 29.
- *Pediatric Patients 6 Years of Age and Older*:

Pediatric Weight	Recommended Dosage	
	Days 1 and 15	Starting on Day 29
17 kg (37 lbs) to less than 40 kg (88 lbs)	Day 1: 80 mg<br>Day 15: 40 mg	20 mg every other week
40 kg (88 lbs) and greater	Day 1: 160 mg (single dose or split over two consecutive days)<br>Day 15: 80 mg	40 mg every other week

Ulcerative Colitis (2.4**):**
- *Adults:* 160 mg on Day 1 (given in one day or split over two consecutive days), 80 mg on Day 15 and 40 mg every other week starting on Day 29. Discontinue in patients without evidence of clinical remission by eight weeks (Day 57).
- *Pediatric Patients 5 Years of Age and Older*:

Pediatric Weight	Recommended Dosage	
	Days 1 through 15	Starting on Day 29*
20 kg (44 lbs) to less than 40 kg (88 lbs)	Day 1: 80 mg<br>Day 8: 40 mg<br>Day 15: 40 mg	40 mg every other week<br>or<br>20 mg every week
40 kg (88 lbs) and greater	Day 1: 160 mg (single dose or split over two consecutive days)<br>Day 8: 80 mg<br>Day 15: 80 mg	80 mg every other week<br>or<br>40 mg every week

* Continue the recommended pediatric dosage in patients who turn 18 years of age and who are well-controlled on their HUMIRA regimen.

Plaque Psoriasis or Adult Uveitis (2.5**):**
- *Adults*: 80 mg initial dose, followed by 40 mg every other week starting one week after initial dose.

Hidradenitis Suppurativa (2.6):
- *Adults*:
    - Day 1: 160 mg (given in one day or split over two consecutive days)
    - Day 15: 80 mg
    - Day 29 and subsequent doses: 40 mg every week or 80 mg every other week
- *Adolescents 12 years of age and older:*

Adolescent Weight	Recommended Dosage
30 kg (66 lbs) [illegible]	Day 1: 80 mg

to less than 60 kg (132 lbs)	[illegible]<br>Day 8 and subsequent doses: 40 mg every other week
60 kg (132 lbs) and greater	Day 1: 160 mg (given in one day or split over two consecutive days)<br>Day 15: 80 mg<br>Day 29 and subsequent doses: 40 mg every week or 80 mg every other week

DOSAGE FORMS AND STRENGTHS

Injection:

- Single-dose prefilled pen (HUMIRA Pen): 80 mg/0.8 mL, 40 mg/0.8 mL, and 40 mg/0.4 mL (3)
- Single-dose prefilled glass syringe: 80 mg/0.8 mL, 40 mg/0.8 mL, 40 mg/0.4 mL, 20 mg/0.4 mL, 20 mg/0.2 mL, 10 mg/0.2 mL, 10 mg/0.1 mL (3)
- Single-dose glass vial for institutional use only: 40 mg/0.8 mL (3)

CONTRAINDICATIONS

None (4)

WARNINGS AND PRECAUTIONS

- *Serious infections:* Do not start HUMIRA during an active infection. If an infection develops, monitor carefully, and stop HUMIRA if infection becomes serious. (5.1)
- *Invasive fungal infections:* For patients who develop a systemic illness on HUMIRA, consider empiric antifungal therapy for those who reside or travel to regions where mycoses are endemic. (5.1)
- *Malignancies:* Incidence of malignancies was greater in HUMIRA-treated patients than in controls (5.2)
- *Anaphylaxis or serious hypersensitivity reactions* may occur (5.3)
- *Hepatitis B virus reactivation:* Monitor HBV carriers during and several months after therapy. If reactivation occurs, stop HUMIRA and begin anti-viral therapy. (5.4)
- *Demyelinating disease:* Exacerbation or new onset, may occur. (5.5)
- *Cytopenias, pancytopenia:* Advise patients to seek immediate medical attention if symptoms develop, and consider stopping HUMIRA. (5.6)
- *Heart failure:* Worsening or new onset, may occur. (5.8)
- *Lupus-like syndrome:* Stop HUMIRA if syndrome develops. (5.9)

ADVERSE REACTIONS

Most common adverse reactions (>10%) are: infections (e.g. upper respiratory, sinusitis), injection site reactions, headache and rash. (6.1)

To report SUSPECTED ADVERSE REACTIONS, contact AbbVie Inc. at 1-800-633-9110 or FDA at 1-800-FDA-1088 or www.fda.gov/medwatch

DRUG INTERACTIONS

- *Abatacept:* Increased risk of serious infection. (5.1, 5.11, 7.2)
- *Anakinra:* Increased risk of serious infection. (5.1, 5.7, 7.2)
- *Live vaccines:* Avoid use with HUMIRA. (5.10, 7.3)

See 17 for PATIENT COUNSELING INFORMATION and Medication Guide.

Revised: 11/2023

FULL PRESCRIBING INFORMATION: CONTENTS*

WARNING: SERIOUS INFECTIONS AND MALIGNANCY

1 INDICATIONS AND USAGE

1.1 Rheumatoid Arthritis

1.2 Juvenile Idiopathic Arthritis

1.3 Psoriatic Arthritis

1.4 Ankylosing Spondylitis

1.5 Crohn’s Disease

1.6 Ulcerative Colitis

1.7 Plaque Psoriasis
1.8 Hidradenitis Suppurativa
1.9 Uveitis
2 DOSAGE AND ADMINISTRATION
2.1 Rheumatoid Arthritis, Psoriatic Arthritis, and Ankylosing Spondylitis
2.2 Juvenile Idiopathic Arthritis or Pediatric Uveitis
2.3 Crohn’s Disease
2.4 Ulcerative Colitis
2.5 Plaque Psoriasis or Adult Uveitis
2.6 Hidradenitis Suppurativa
2.7 Monitoring to Assess Safety
2.8 General Considerations for Administration
3 DOSAGE FORMS AND STRENGTHS
4 CONTRAINDICATIONS
5 WARNINGS AND PRECAUTIONS
5.1 Serious Infections
5.2 Malignancies
5.3 Hypersensitivity Reactions
5.4 Hepatitis B Virus Reactivation
5.5 Neurologic Reactions
5.6 Hematological Reactions
5.7 Increased Risk of Infection when Used with Anakinra
5.8 Heart Failure
5.9 Autoimmunity
5.10 Immunizations
5.11 Increased Risk of Infection When Used with Abatacept
6 ADVERSE REACTIONS
6.1 Clinical Trials Experience
6.2 Immunogenicity
6.3 Postmarketing Experience
7 DRUG INTERACTIONS
7.1 Methotrexate
7.2 Biological Products
7.3 Live Vaccines
7.4 Cytochrome P450 Substrates
8 USE IN SPECIFIC POPULATIONS
8.1 Pregnancy
8.2 Lactation
8.4 Pediatric Use
8.5 Geriatric Use
10 OVERDOSAGE
11 DESCRIPTION
12 CLINICAL PHARMACOLOGY
12.1 Mechanism of Action
12.2 Pharmacodynamics
12.3 Pharmacokinetics
13 NONCLINICAL TOXICOLOGY
13.1 Carcinogenesis, Mutagenesis, Impairment of Fertility
14 CLINICAL STUDIES
14.1 Rheumatoid Arthritis

14.2 Juvenile Idiopathic Arthritis
14.3 Psoriatic Arthritis
14.4 Ankylosing Spondylitis
14.5 Adult Crohn's Disease
14.6 Pediatric Crohn's Disease
14.7 Adult Ulcerative Colitis
14.8 Pediatric Ulcerative Colitis
14.9 Plaque Psoriasis
14.10 Hidradenitis Suppurativa
14.11 Adult Uveitis
14.12 Pediatric Uveitis
15 REFERENCES
16 HOW SUPPLIED/STORAGE AND HANDLING
17 PATIENT COUNSELING INFORMATION
* Sections or subsections omitted from the full prescribing information are not listed.

FULL PRESCRIBING INFORMATION

WARNING: SERIOUS INFECTIONS AND MALIGNANCY

SERIOUS INFECTIONS

Patients treated with HUMIRA are at increased risk for developing serious infections that may lead to hospitalization or death *[see Warnings and Precautions (5.1)]*. Most patients who developed these infections were taking concomitant immunosuppressants such as methotrexate or corticosteroids.

Discontinue HUMIRA if a patient develops a serious infection or sepsis.

Reported infections include:

- **Active tuberculosis (TB), including reactivation of latent TB. Patients with TB have frequently presented with disseminated or extrapulmonary disease. Test patients for latent TB before HUMIRA use and during therapy. Initiate treatment for latent TB prior to HUMIRA use.**
- **Invasive fungal infections, including histoplasmosis, coccidioidomycosis, candidiasis, aspergillosis, blastomycosis, and pneumocystosis. Patients with histoplasmosis or other invasive fungal infections may present with disseminated, rather than localized, disease. Antigen and antibody testing for histoplasmosis may be negative in some patients with active infection. Consider empiric anti-fungal therapy in patients at risk for invasive fungal infections who develop severe systemic illness.**
- **Bacterial, viral and other infections due to opportunistic pathogens, including Legionella and Listeria.**

Carefully consider the risks and benefits of treatment with HUMIRA prior to initiating therapy in patients with chronic or recurrent infection.

Monitor patients closely for the development of signs and symptoms of infection during and after treatment with HUMIRA, including the possible development of TB in patients who tested negative for latent TB infection prior to initiating therapy *[see Warnings and Precautions (5.1) and Adverse Reactions (6.1)]*.

MALIGNANCY

Lymphoma and other malignancies, some fatal, have been reported in children and adolescent patients treated with TNF blockers including HUMIRA *[see Warnings and Precautions (5.2)]*. Post-marketing cases of hepatosplenic T-cell lymphoma (HSTCL), a rare type of T-cell lymphoma, have been reported in patients treated with TNF blockers including HUMIRA. These cases have had a very aggressive disease course and have been fatal. The majority of reported TNF blocker cases have occurred in patients with Crohn's disease or ulcerative colitis and the majority were in adolescent and young adult males. Almost all these patients had received treatment with azathioprine or 6-mercaptopurine (6–MP) concomitantly with a TNF blocker at or prior to diagnosis. It is uncertain whether the occurrence of HSTCL is related to use of a TNF blocker or a TNF blocker in combination with these other

immunosuppressants *[see Warnings and Precautions (5.2)]*.

1 INDICATIONS AND USAGE

1.1 Rheumatoid Arthritis

HUMIRA is indicated for reducing signs and symptoms, inducing major clinical response, inhibiting the progression of structural damage, and improving physical function in adult patients with moderately to severely active rheumatoid arthritis. HUMIRA can be used alone or in combination with methotrexate or other non-biologic disease-modifying anti-rheumatic drugs (DMARDs).

1.2 Juvenile Idiopathic Arthritis

HUMIRA is indicated for reducing signs and symptoms of moderately to severely active polyarticular juvenile idiopathic arthritis in patients 2 years of age and older. HUMIRA can be used alone or in combination with methotrexate.

1.3 Psoriatic Arthritis

HUMIRA is indicated for reducing signs and symptoms, inhibiting the progression of structural damage, and improving physical function in adult patients with active psoriatic arthritis. HUMIRA can be used alone or in combination with non-biologic DMARDs.

1.4 Ankylosing Spondylitis

HUMIRA is indicated for reducing signs and symptoms in adult patients with active ankylosing spondylitis.

1.5 Crohn's Disease

HUMIRA is indicated for the treatment of moderately to severely active Crohn's disease in adults and pediatric patients 6 years of age and older.

1.6 Ulcerative Colitis

HUMIRA is indicated for the treatment of moderately to severely active ulcerative colitis in adults and pediatric patients 5 years of age and older.

<u>Limitations of Use</u>
The effectiveness of HUMIRA has not been established in patients who have lost response to or were intolerant to TNF blockers *[see Clinical Studies (14.7, 14.8)]*.

1.7 Plaque Psoriasis

HUMIRA is indicated for the treatment of adult patients with moderate to severe chronic plaque psoriasis who are candidates for systemic therapy or phototherapy, and when other systemic therapies are medically less appropriate. HUMIRA should only be administered to patients who will be closely monitored and have regular follow-up visits with a physician *[see Warnings and Precautions (5)]*.

1.8 Hidradenitis Suppurativa

HUMIRA is indicated for the treatment of moderate to severe hidradenitis suppurativa in patients 12 years of age and older.

1.9 Uveitis

HUMIRA is indicated for the treatment of non-infectious intermediate, posterior, and panuveitis in adults and pediatric patients 2 years of age and older.

2 DOSAGE AND ADMINISTRATION

2.1 Rheumatoid Arthritis, Psoriatic Arthritis, and Ankylosing Spondylitis

The recommended subcutaneous dosage of HUMIRA for adult patients with rheumatoid arthritis (RA), psoriatic arthritis (PsA), or ankylosing spondylitis (AS) is 40 mg administered every other week. Methotrexate (MTX), other non-biologic DMARDS, glucocorticoids, nonsteroidal anti-inflammatory drugs (NSAIDs), and/or analgesics may be continued during treatment with HUMIRA. In the treatment of RA, some patients not taking concomitant MTX may derive additional benefit from increasing the dosage of HUMIRA to 40 mg every week or 80 mg every other week.

2.2 Juvenile Idiopathic Arthritis or Pediatric Uveitis

The recommended subcutaneous dosage of HUMIRA for patients 2 years of age and older with polyarticular juvenile idiopathic arthritis (JIA) or pediatric uveitis is based on weight as shown below. MTX, glucocorticoids, NSAIDs, and/or analgesics may be continued during treatment with HUMIRA.

Pediatric Weight (2 Years of Age and older)	Recommended Dosage
10 kg (22 lbs) to less than 15 kg (33 lbs)	10 mg every other week
15 kg (33 lbs) to less than 30 kg (66 lbs)	20 mg every other week
30 kg (66 lbs) and greater	40 mg every other week

HUMIRA has not been studied in patients with polyarticular JIA or pediatric uveitis less than 2 years of age or in patients with a weight below 10 kg.

2.3 Crohn's Disease

Adults

The recommended subcutaneous dosage of HUMIRA for adult patients with Crohn's disease (CD) is 160 mg initially on Day 1 (given in one day or split over two consecutive days), followed by 80 mg two weeks later (Day 15). Two weeks later (Day 29) begin a dosage of 40 mg every other week. Aminosalicylates and/or corticosteroids may be continued during treatment with HUMIRA. Azathioprine, 6-mercaptopurine (6-MP) *[see Warnings and Precautions (5.2)]* or MTX may be continued during treatment with HUMIRA if necessary.

Pediatrics

The recommended subcutaneous dosage of HUMIRA for pediatric patients 6 years of

age and older with Crohn's disease (CD) is based on body weight as shown below:

Pediatric Weight	Recommended Dosage	
	Days 1 through 15	Starting on Day 29
17 kg (37 lbs) to less than 40 kg (88 lbs)	Day 1: 80 mg<br>Day 15: 40 mg	20 mg every other week
40 kg (88 lbs) and greater	Day 1: 160 mg (single dose or split over two consecutive days)<br>Day 15: 80 mg	40 mg every other week

2.4 Ulcerative Colitis

Adults

The recommended subcutaneous dosage of HUMIRA for adult patients with ulcerative colitis is 160 mg initially on Day 1 (given in one day or split over two consecutive days), followed by 80 mg two weeks later (Day 15). Two weeks later (Day 29) continue with a dosage of 40 mg every other week.

Discontinue HUMIRA in adult patients without evidence of clinical remission by eight weeks (Day 57) of therapy. Aminosalicylates and/or corticosteroids may be continued during treatment with HUMIRA. Azathioprine and 6-mercaptopurine (6-MP) *[see Warnings and Precautions (5.2)]* may be continued during treatment with HUMIRA if necessary.

Pediatrics

The recommended subcutaneous dosage of HUMIRA for pediatric patients 5 years of age and older with ulcerative colitis is based on body weight as shown below:

Pediatric Weight	Recommended Dosage	
	Days 1 through 15	Starting on Day 29*
20 kg (44 lbs) to less than 40 kg (88 lbs)	Day 1: 80 mg<br>Day 8: 40 mg<br>Day 15: 40 mg	40 mg every other week<br>or<br>20 mg every week
40 kg (88 lbs) and greater	Day 1: 160 mg (single dose or split over two consecutive days) Day 8: 80 mg<br>Day 15: 80 mg	80 mg every other week<br>or<br>40 mg every week

* Continue the recommended pediatric dosage in patients who turn 18 years of age and who are well-controlled on their HUMIRA regimen.

2.5 Plaque Psoriasis or Adult Uveitis

The recommended subcutaneous dosage of HUMIRA for adult patients with plaque psoriasis (Ps) or Uveitis (UV) is an initial dose of 80 mg, followed by 40 mg given every other week starting one week after the initial dose. The use of HUMIRA in moderate to severe chronic Ps beyond one year has not been evaluated in controlled clinical studies.

2.6 Hidradenitis Suppurativa

Adults

The recommended subcutaneous dosage of HUMIRA for adult patients with hidradenitis suppurativa (HS) is an initial dose of 160 mg (given in one day or split over two consecutive days), followed by 80 mg two weeks later (Day 15). Begin 40 mg weekly or 80 mg every other week dosing two weeks later (Day 29).

Adolescents

The recommended subcutaneous dosage of HUMIRA for adolescent patients 12 years of age and older weighing at least 30 kg with hidradenitis suppurativa (HS) is based on body weight as shown below *[see Use in Specific Populations (8.4) and Clinical Pharmacology (12.3)]*:

Body Weight of Adolescent Patients (12 years of age and older)	Recommended Dosage
30 kg (66 lbs) to less than 60 kg (132 lbs)	• Day 1: 80 mg<br>• Day 8 and subsequent doses: 40 mg every other week
60 kg (132 lbs) and greater	• Day 1: 160 mg (given in one day or split over two consecutive days);<br>• Day 15: 80 mg<br>• Day 29 and subsequent doses: 40 mg every week or 80 mg every other week

2.7 Monitoring to Assess Safety

Prior to initiating HUMIRA and periodically during therapy, evaluate patients for active tuberculosis and test for latent infection *[see Warnings and Precautions (5.1)]*.

2.8 General Considerations for Administration

HUMIRA is intended for use under the guidance and supervision of a physician. A patient may self-inject HUMIRA or a caregiver may inject HUMIRA using either the HUMIRA Pen or prefilled syringe if a physician determines that it is appropriate, and with medical follow-up, as necessary, after proper training in subcutaneous injection technique.

HUMIRA can be taken out of the refrigerator for 15 to 30 minutes before injecting to allow the liquid to come to room temperature. Do not remove the cap or cover while allowing it to reach room temperature. Carefully inspect the solution in the HUMIRA Pen, prefilled syringe, or single-dose institutional use vial for particulate matter and discoloration prior to subcutaneous administration. If particulates and discolorations are noted, do not use the product. HUMIRA does not contain preservatives; therefore, discard unused portions of drug remaining from the syringe. NOTE: Instruct patients sensitive to latex not to handle the needle cover of the HUMIRA 40 mg/0.8 mL Pen and

40 mg/0.8 mL, 20 mg/0.4 mL and 10 mg/0.2 mL prefilled syringe because it may contain natural rubber latex *[see How Supplied/Storage and Handling (16)]*.

Instruct patients using the HUMIRA Pen or prefilled syringe to inject the full amount in the syringe, according to the directions provided in the Instructions for Use *[see Instructions for Use]*.

Injections should occur at separate sites in the thigh or abdomen. Rotate injection sites and do not give injections into areas where the skin is tender, bruised, red or hard.

If a dose is missed, administer the dose as soon as possible. Thereafter, resume dosing at the regular scheduled time.

The HUMIRA single-dose institutional use vial is for administration within an institutional setting only, such as a hospital, physician’s office or clinic. Withdraw the dose using a sterile needle and syringe and administer promptly by a healthcare provider within an institutional setting. Only administer one dose per vial. The vial does not contain preservatives; therefore, discard unused portions.

3 DOSAGE FORMS AND STRENGTHS

HUMIRA is a clear and colorless solution available as:

- **Pen** (HUMIRA Pen)
  Injection: 80 mg/0.8 mL in a single-dose pen.
  Injection: 40 mg/0.8 mL in a single-dose pen.
  Injection: 40 mg/0.4 mL in a single-dose pen.
- **Prefilled Syringe**
  Injection: 80 mg/0.8 mL in a single-dose prefilled glass syringe.
  Injection: 40 mg/0.8 mL in a single-dose prefilled glass syringe.
  Injection: 40 mg/0.4 mL in a single-dose prefilled glass syringe.
  Injection: 20 mg/0.4 mL in a single-dose prefilled glass syringe.
  Injection: 20 mg/0.2 mL in a single-dose prefilled glass syringe.
  Injection: 10 mg/0.2 mL in a single-dose prefilled glass syringe.
  Injection: 10 mg/0.1 mL in a single-dose prefilled glass syringe.
- **Single-Dose Institutional Use Vial**
  Injection: 40 mg/0.8 mL in a single-dose, glass vial for institutional use only.

4 CONTRAINDICATIONS

None.

5 WARNINGS AND PRECAUTIONS

5.1 Serious Infections

Patients treated with HUMIRA are at increased risk for developing serious infections involving various organ systems and sites that may lead to hospitalization or death. Opportunistic infections due to bacterial, mycobacterial, invasive fungal, viral, parasitic, or other opportunistic pathogens including aspergillosis, blastomycosis, candidiasis, coccidioidomycosis, histoplasmosis, legionellosis, listeriosis, pneumocystosis and tuberculosis have been reported with TNF blockers. Patients have frequently presented

with disseminated rather than localized disease.

The concomitant use of a TNF blocker and abatacept or anakinra was associated with a higher risk of serious infections in patients with rheumatoid arthritis (RA); therefore, the concomitant use of HUMIRA and these biologic products is not recommended in the treatment of patients with RA *[see Warnings and Precautions (5.7, 5.11) and Drug Interactions (7.2)]*.

Treatment with HUMIRA should not be initiated in patients with an active infection, including localized infections. Patients 65 years of age and older, patients with co-morbid conditions and/or patients taking concomitant immunosuppressants (such as corticosteroids or methotrexate), may be at greater risk of infection. Consider the risks and benefits of treatment prior to initiating therapy in patients:

- with chronic or recurrent infection;
- who have been exposed to tuberculosis;
- with a history of an opportunistic infection;
- who have resided or traveled in areas of endemic tuberculosis or endemic mycoses, such as histoplasmosis, coccidioidomycosis, or blastomycosis; or
- with underlying conditions that may predispose them to infection.

Tuberculosis

Cases of reactivation of tuberculosis and new onset tuberculosis infections have been reported in patients receiving HUMIRA, including patients who have previously received treatment for latent or active tuberculosis. Reports included cases of pulmonary and extrapulmonary (i.e., disseminated) tuberculosis. Evaluate patients for tuberculosis risk factors and test for latent infection prior to initiating HUMIRA and periodically during therapy.

Treatment of latent tuberculosis infection prior to therapy with TNF blocking agents has been shown to reduce the risk of tuberculosis reactivation during therapy. Prior to initiating HUMIRA, assess if treatment for latent tuberculosis is needed; and consider an induration of ≥ 5 mm a positive tuberculin skin test result, even for patients previously vaccinated with Bacille Calmette-Guerin (BCG).

Consider anti-tuberculosis therapy prior to initiation of HUMIRA in patients with a past history of latent or active tuberculosis in whom an adequate course of treatment cannot be confirmed, and for patients with a negative test for latent tuberculosis but having risk factors for tuberculosis infection. Despite prophylactic treatment for tuberculosis, cases of reactivated tuberculosis have occurred in patients treated with HUMIRA. Consultation with a physician with expertise in the treatment of tuberculosis is recommended to aid in the decision whether initiating anti-tuberculosis therapy is appropriate for an individual patient.

Strongly consider tuberculosis in the differential diagnosis in patients who develop a new infection during HUMIRA treatment, especially in patients who have previously or recently traveled to countries with a high prevalence of tuberculosis, or who have had close contact with a person with active tuberculosis.

Monitoring

Closely monitor patients for the development of signs and symptoms of infection during and after treatment with HUMIRA, including the development of tuberculosis in patients who tested negative for latent tuberculosis infection prior to initiating therapy. Tests for

latent tuberculosis infection may also be falsely negative while on therapy with HUMIRA.

Discontinue HUMIRA if a patient develops a serious infection or sepsis. For a patient who develops a new infection during treatment with HUMIRA, closely monitor them, perform a prompt and complete diagnostic workup appropriate for an immunocompromised patient, and initiate appropriate antimicrobial therapy.

Invasive Fungal Infections

If patients develop a serious systemic illness and they reside or travel in regions where mycoses are endemic, consider invasive fungal infection in the differential diagnosis. Antigen and antibody testing for histoplasmosis may be negative in some patients with active infection. Consider appropriate empiric antifungal therapy, taking into account both the risk for severe fungal infection and the risks of antifungal therapy, while a diagnostic workup is being performed. To aid in the management of such patients, consider consultation with a physician with expertise in the diagnosis and treatment of invasive fungal infections.

5.2 Malignancies

Consider the risks and benefits of TNF-blocker treatment including HUMIRA prior to initiating therapy in patients with a known malignancy other than a successfully treated non-melanoma skin cancer (NMSC) or when considering continuing a TNF blocker in patients who develop a malignancy.

Malignancies in Adults

In the controlled portions of clinical trials of some TNF-blockers, including HUMIRA, more cases of malignancies have been observed among TNF-blocker-treated adult patients compared to control-treated adult patients. During the controlled portions of 39 global HUMIRA clinical trials in adult patients with rheumatoid arthritis (RA), psoriatic arthritis (PsA), ankylosing spondylitis (AS), Crohn’s disease (CD), ulcerative colitis (UC), plaque psoriasis (Ps), hidradenitis suppurativa (HS) and uveitis (UV), malignancies, other than non-melanoma (basal cell and squamous cell) skin cancer, were observed at a rate (95% confidence interval) of 0.7 (0.48, 1.03) per 100 patient-years among 7973 HUMIRA-treated patients versus a rate of 0.7 (0.41, 1.17) per 100 patient-years among 4848 control-treated patients (median duration of treatment of 4 months for HUMIRA-treated patients and 4 months for control-treated patients). In 52 global controlled and uncontrolled clinical trials of HUMIRA in adult patients with RA, PsA, AS, CD, UC, Ps, HS and UV, the most frequently observed malignancies, other than lymphoma and NMSC, were breast, colon, prostate, lung, and melanoma. The malignancies in HUMIRA-treated patients in the controlled and uncontrolled portions of the studies were similar in type and number to what would be expected in the general U.S. population according to the SEER database (adjusted for age, gender, and race).[1]

In controlled trials of other TNF blockers in adult patients at higher risk for malignancies (i.e., patients with COPD with a significant smoking history and cyclophosphamide-treated patients with Wegener’s granulomatosis), a greater portion of malignancies occurred in the TNF blocker group compared to the control group.

Non-Melanoma Skin Cancer

During the controlled portions of 39 global HUMIRA clinical trials in adult patients with RA, PsA, AS, CD, UC, Ps, HS and UV, the rate (95% confidence interval) of NMSC was

0.8 (0.52, 1.09) per 100 patient-years among HUMIRA-treated patients and 0.2 (0.10, 0.59) per 100 patient-years among control-treated patients. Examine all patients, and in particular patients with a medical history of prior prolonged immunosuppressant therapy or psoriasis patients with a history of PUVA treatment for the presence of NMSC prior to and during treatment with HUMIRA.

Lymphoma and Leukemia

In the controlled portions of clinical trials of all the TNF-blockers in adults, more cases of lymphoma have been observed among TNF-blocker-treated patients compared to control-treated patients. In the controlled portions of 39 global HUMIRA clinical trials in adult patients with RA, PsA, AS, CD, UC, Ps, HS and UV, 2 lymphomas occurred among 7973 HUMIRA-treated patients versus 1 among 4848 control-treated patients. In 52 global controlled and uncontrolled clinical trials of HUMIRA in adult patients with RA, PsA, AS, CD, UC, Ps, HS and UV with a median duration of approximately 0.7 years, including 24,605 patients and over 40,215 patient-years of HUMIRA, the observed rate of lymphomas was approximately 0.11 per 100 patient-years. This is approximately 3-fold higher than expected in the general U.S. population according to the SEER database (adjusted for age, gender, and race).[1] Rates of lymphoma in clinical trials of HUMIRA cannot be compared to rates of lymphoma in clinical trials of other TNF blockers and may not predict the rates observed in a broader patient population. Patients with RA and other chronic inflammatory diseases, particularly those with highly active disease and/or chronic exposure to immunosuppressant therapies, may be at a higher risk (up to several fold) than the general population for the development of lymphoma, even in the absence of TNF blockers. Post-marketing cases of acute and chronic leukemia have been reported in association with TNF-blocker use in RA and other indications. Even in the absence of TNF-blocker therapy, patients with RA may be at a higher risk (approximately 2-fold) than the general population for the development of leukemia.

Malignancies in Pediatric Patients and Young Adults

Malignancies, some fatal, have been reported among children, adolescents, and young adults who received treatment with TNF-blockers (initiation of therapy ≤ 18 years of age), of which HUMIRA is a member. Approximately half the cases were lymphomas, including Hodgkin's and non-Hodgkin's lymphoma. The other cases represented a variety of different malignancies and included rare malignancies usually associated with immunosuppression and malignancies that are not usually observed in children and adolescents. The malignancies occurred after a median of 30 months of therapy (range 1 to 84 months). Most of the patients were receiving concomitant immunosuppressants. These cases were reported post-marketing and are derived from a variety of sources including registries and spontaneous postmarketing reports.

Postmarketing cases of hepatosplenic T-cell lymphoma (HSTCL), a rare type of T-cell lymphoma, have been reported in patients treated with TNF blockers including HUMIRA. These cases have had a very aggressive disease course and have been fatal. The majority of reported TNF blocker cases have occurred in patients with Crohn's disease or ulcerative colitis and the majority were in adolescent and young adult males. Almost all of these patients had received treatment with the immunosuppressants azathioprine or 6-mercaptopurine (6–MP) concomitantly with a TNF blocker at or prior to diagnosis. It is uncertain whether the occurrence of HSTCL is related to use of a TNF blocker or a TNF blocker in combination with these other immunosuppressants. The potential risk with the combination of azathioprine or 6-mercaptopurine and HUMIRA should be carefully

considered.

5.3 Hypersensitivity Reactions

Anaphylaxis and angioneurotic edema have been reported following HUMIRA administration. If an anaphylactic or other serious allergic reaction occurs, immediately discontinue administration of HUMIRA and institute appropriate therapy. In clinical trials of HUMIRA, hypersensitivity reactions (e.g., rash, anaphylactoid reaction, fixed drug reaction, non-specified drug reaction, urticaria) have been observed.

5.4 Hepatitis B Virus Reactivation

Use of TNF blockers, including HUMIRA, may increase the risk of reactivation of hepatitis B virus (HBV) in patients who are chronic carriers of this virus. In some instances, HBV reactivation occurring in conjunction with TNF blocker therapy has been fatal. The majority of these reports have occurred in patients concomitantly receiving other medications that suppress the immune system, which may also contribute to HBV reactivation. Evaluate patients at risk for HBV infection for prior evidence of HBV infection before initiating TNF blocker therapy. Exercise caution in prescribing TNF blockers for patients identified as carriers of HBV. Adequate data are not available on the safety or efficacy of treating patients who are carriers of HBV with anti-viral therapy in conjunction with TNF blocker therapy to prevent HBV reactivation. For patients who are carriers of HBV and require treatment with TNF blockers, closely monitor such patients for clinical and laboratory signs of active HBV infection throughout therapy and for several months following termination of therapy. In patients who develop HBV reactivation, stop HUMIRA and initiate effective anti-viral therapy with appropriate supportive treatment. The safety of resuming TNF blocker therapy after HBV reactivation is controlled is not known. Therefore, exercise caution when considering resumption of HUMIRA therapy in this situation and monitor patients closely.

5.5 Neurologic Reactions

Use of TNF blocking agents, including HUMIRA, has been associated with rare cases of new onset or exacerbation of clinical symptoms and/or radiographic evidence of central nervous system demyelinating disease, including multiple sclerosis (MS) and optic neuritis, and peripheral demyelinating disease, including Guillain-Barré syndrome. Exercise caution in considering the use of HUMIRA in patients with preexisting or recent-onset central or peripheral nervous system demyelinating disorders; discontinuation of HUMIRA should be considered if any of these disorders develop. There is a known association between intermediate uveitis and central demyelinating disorders.

5.6 Hematological Reactions

Rare reports of pancytopenia including aplastic anemia have been reported with TNF blocking agents. Adverse reactions of the hematologic system, including medically significant cytopenia (e.g., thrombocytopenia, leukopenia) have been infrequently reported with HUMIRA. The causal relationship of these reports to HUMIRA remains unclear. Advise all patients to seek immediate medical attention if they develop signs and symptoms suggestive of blood dyscrasias or infection (e.g., persistent fever, bruising, bleeding, pallor) while on HUMIRA. Consider discontinuation of HUMIRA therapy in patients with confirmed significant hematologic abnormalities.

5.7 Increased Risk of Infection when Used with Anakinra

Concurrent use of anakinra (an interleukin-1 antagonist) and another TNF-blocker, was associated with a greater proportion of serious infections and neutropenia and no added benefit compared with the TNF-blocker alone in patients with RA. Therefore, the combination of HUMIRA and anakinra is not recommended *[see Drug Interactions (7.2)].*

5.8 Heart Failure

Cases of worsening congestive heart failure (CHF) and new onset CHF have been reported with TNF blockers. Cases of worsening CHF have also been observed with HUMIRA. HUMIRA has not been formally studied in patients with CHF; however, in clinical trials of another TNF blocker, a higher rate of serious CHF-related adverse reactions was observed. Exercise caution when using HUMIRA in patients who have heart failure and monitor them carefully.

5.9 Autoimmunity

Treatment with HUMIRA may result in the formation of autoantibodies and, rarely, in the development of a lupus-like syndrome. If a patient develops symptoms suggestive of a lupus-like syndrome following treatment with HUMIRA, discontinue treatment *[see Adverse Reactions (6.1)]*.

5.10 Immunizations

In a placebo-controlled clinical trial of patients with RA, no difference was detected in anti-pneumococcal antibody response between HUMIRA and placebo treatment groups when the pneumococcal polysaccharide vaccine and influenza vaccine were administered concurrently with HUMIRA. Similar proportions of patients developed protective levels of anti-influenza antibodies between HUMIRA and placebo treatment groups; however, titers in aggregate to influenza antigens were moderately lower in patients receiving HUMIRA. The clinical significance of this is unknown. Patients on HUMIRA may receive concurrent vaccinations, except for live vaccines. No data are available on the secondary transmission of infection by live vaccines in patients receiving HUMIRA.

It is recommended that pediatric patients, if possible, be brought up to date with all immunizations in agreement with current immunization guidelines prior to initiating HUMIRA therapy. Patients on HUMIRA may receive concurrent vaccinations, except for live vaccines.

The safety of administering live or live-attenuated vaccines in infants exposed to HUMIRA *in utero* is unknown. Risks and benefits should be considered prior to vaccinating (live or live-attenuated) exposed infants *[see Use in Specific Populations (8.1, 8.4)]*.

5.11 Increased Risk of Infection When Used with Abatacept

In controlled trials, the concurrent administration of TNF-blockers and abatacept was associated with a greater proportion of serious infections than the use of a TNF-blocker alone; the combination therapy, compared to the use of a TNF-blocker alone, has not demonstrated improved clinical benefit in the treatment of RA. Therefore, the combination of abatacept with TNF-blockers including HUMIRA is not recommended *[see Drug Interactions (7.2)]*.

6 ADVERSE REACTIONS

The following clinically significant adverse reactions are described elsewhere in the labeling:

- Serious Infections *[see Warnings and Precautions (5.1)]*
- Malignancies *[see Warnings and Precautions (5.2)]*
- Hypersensitivity Reactions *[see Warnings and Precautions (5.3)]*
- Hepatitis B Virus Reactivation *[see Warnings and Precautions (5.4)]*
- Neurologic Reactions *[see Warnings and Precautions (5.5)]*
- Hematological Reactions *[see Warnings and Precautions (5.6)]*
- Heart Failure *[see Warnings and Precautions (5.8)]*
- Autoimmunity *[see Warnings and Precautions (5.9)]*

6.1 Clinical Trials Experience

Because clinical trials are conducted under widely varying conditions, adverse reaction rates observed in the clinical trials of a drug cannot be directly compared to rates in the clinical trials of another drug and may not reflect the rates observed in practice.

The most common adverse reaction with HUMIRA was injection site reactions. In placebo-controlled trials, 20% of patients treated with HUMIRA developed injection site reactions (erythema and/or itching, hemorrhage, pain or swelling), compared to 14% of patients receiving placebo. Most injection site reactions were described as mild and generally did not necessitate drug discontinuation.

The proportion of patients who discontinued treatment due to adverse reactions during the double-blind, placebo-controlled portion of studies in patients with RA (i.e., Studies RA-I, RA-II, RA-III and RA-IV) was 7% for patients taking HUMIRA and 4% for placebo-treated patients. The most common adverse reactions leading to discontinuation of HUMIRA in these RA studies were clinical flare reaction (0.7%), rash (0.3%) and pneumonia (0.3%).

<u>Infections</u>

In the controlled portions of the 39 global HUMIRA clinical trials in adult patients with RA, PsA, AS, CD, UC, Ps, HS and UV, the rate of serious infections was 4.3 per 100 patient-years in 7973 HUMIRA-treated patients versus a rate of 2.9 per 100 patient-years in 4848 control-treated patients. Serious infections observed included pneumonia, septic arthritis, prosthetic and post-surgical infections, erysipelas, cellulitis, diverticulitis, and pyelonephritis *[see Warnings and Precautions (5.1)]*.

Tuberculosis and Opportunistic Infections

In 52 global controlled and uncontrolled clinical trials in RA, PsA, AS, CD, UC, Ps, HS and UV that included 24,605 HUMIRA-treated patients, the rate of reported active tuberculosis was 0.20 per 100 patient-years and the rate of positive PPD conversion was 0.09 per 100 patient-years. In a subgroup of 10,113 U.S. and Canadian HUMIRA-treated patients, the rate of reported active TB was 0.05 per 100 patient-years and the rate of positive PPD conversion was 0.07 per 100 patient-years. These trials included reports of miliary, lymphatic, peritoneal, and pulmonary TB. Most of the TB cases occurred within the first eight months after initiation of therapy and may reflect recrudescence of latent disease. In these global clinical trials, cases of serious

opportunistic infections have been reported at an overall rate of 0.05 per 100 patient-years. Some cases of serious opportunistic infections and TB have been fatal *[see Warnings and Precautions (5.1)]*.

<u>Autoantibodies</u>

In the rheumatoid arthritis controlled trials, 12% of patients treated with HUMIRA and 7% of placebo-treated patients that had negative baseline ANA titers developed positive titers at week 24. Two patients out of 3046 treated with HUMIRA developed clinical signs suggestive of new-onset lupus-like syndrome. The patients improved following discontinuation of therapy. No patients developed lupus nephritis or central nervous system symptoms. The impact of long-term treatment with HUMIRA on the development of autoimmune diseases is unknown.

<u>Liver Enzyme Elevations</u>

There have been reports of severe hepatic reactions including acute liver failure in patients receiving TNF-blockers. In controlled Phase 3 trials of HUMIRA (40 mg SC every other week) in patients with RA, PsA, and AS with control period duration ranging from 4 to 104 weeks, ALT elevations ≥ 3 x ULN occurred in 3.5% of HUMIRA-treated patients and 1.5% of control-treated patients. Since many of these patients in these trials were also taking medications that cause liver enzyme elevations (e.g., NSAIDS, MTX), the relationship between HUMIRA and the liver enzyme elevations is not clear. In a controlled Phase 3 trial of HUMIRA in patients with polyarticular JIA who were 4 to 17 years, ALT elevations ≥ 3 x ULN occurred in 4.4% of HUMIRA-treated patients and 1.5% of control-treated patients (ALT more common than AST); liver enzyme test elevations were more frequent among those treated with the combination of HUMIRA and MTX than those treated with HUMIRA alone. In general, these elevations did not lead to discontinuation of HUMIRA treatment. No ALT elevations ≥ 3 x ULN occurred in the open-label study of HUMIRA in patients with polyarticular JIA who were 2 to <4 years.

In controlled Phase 3 trials of HUMIRA (initial doses of 160 mg and 80 mg, or 80 mg and 40 mg on Days 1 and 15, respectively, followed by 40 mg every other week) in adult patients with Crohn’s Disease with a control period duration ranging from 4 to 52 weeks, ALT elevations ≥ 3 x ULN occurred in 0.9% of HUMIRA-treated patients and 0.9% of control-treated patients. In the Phase 3 trial of HUMIRA in pediatric patients with Crohn’s disease which evaluated efficacy and safety of two body weight based maintenance dose regimens following body weight based induction therapy up to 52 weeks of treatment, ALT elevations ≥ 3 x ULN occurred in 2.6% (5/192) of patients, of whom 4 were receiving concomitant immunosuppressants at baseline; none of these patients discontinued due to abnormalities in ALT tests. In controlled Phase 3 trials of HUMIRA (initial doses of 160 mg and 80 mg on Days 1 and 15 respectively, followed by 40 mg every other week) in adult patients with UC with control period duration ranging from 1 to 52 weeks, ALT elevations ≥3 x ULN occurred in 1.5% of HUMIRA-treated patients and 1.0% of control-treated patients. In the controlled Phase 3 trial of HUMIRA in patients with pediatric ulcerative colitis (N=93), which evaluated efficacy and safety of a maintenance dose of 0.6 mg/kg (maximum of 40 mg) every other week (N=31) and a maintenance dose of 0.6 mg/kg (maximum of 40 mg) every week (N=32), following body weight based induction doses of 2.4 mg/kg (maximum of 160 mg) at Week 0 and Week 1, and 1.2 mg/kg (maximum of 80 mg) at Week 2 (N=63), or an induction dose of 2.4 mg/kg (maximum of 160 mg) at Week 0, placebo at Week 1, and 1.2 mg/kg (maximum of 80 mg) at Week 2 (N=30), ALT elevations ≥ 3 X ULN occurred in 1.1%

(1/93) of patients. In controlled Phase 3 trials of HUMIRA (initial dose of 80 mg then 40 mg every other week) in patients with Ps with control period duration ranging from 12 to 24 weeks, ALT elevations ≥ 3 x ULN occurred in 1.8% of HUMIRA-treated patients and 1.8% of control-treated patients. In controlled trials of HUMIRA (initial doses of 160 mg at Week 0 and 80 mg at Week 2, followed by 40 mg every week starting at Week 4), in subjects with HS with a control period duration ranging from 12 to 16 weeks, ALT elevations ≥ 3 x ULN occurred in 0.3% of HUMIRA-treated subjects and 0.6% of control-treated subjects. In controlled trials of HUMIRA (initial doses of 80 mg at Week 0 followed by 40 mg every other week starting at Week 1) in adult patients with uveitis with an exposure of 165.4 PYs and 119.8 PYs in HUMIRA-treated and control-treated patients, respectively, ALT elevations ≥ 3 x ULN occurred in 2.4% of HUMIRA-treated patients and 2.4% of control-treated patients.

Other Adverse Reactions

Rheumatoid Arthritis Clinical Studies

The data described below reflect exposure to HUMIRA in 2468 patients, including 2073 exposed for 6 months, 1497 exposed for greater than one year and 1380 in adequate and well-controlled studies (Studies RA-I, RA-II, RA-III, and RA-IV). HUMIRA was studied primarily in placebo-controlled trials and in long-term follow up studies for up to 36 months duration. The population had a mean age of 54 years, 77% were female, 91% were Caucasian and had moderately to severely active rheumatoid arthritis. Most patients received 40 mg HUMIRA every other week *[see Clinical Studies (14.1)].*

Table 1 summarizes reactions reported at a rate of at least 5% in patients treated with HUMIRA 40 mg every other week compared to placebo and with an incidence higher than placebo. In Study RA-III, the types and frequencies of adverse reactions in the second year open-label extension were similar to those observed in the one-year double-blind portion.

Table 1. Adverse Reactions Reported by ≥5% of Patients Treated with HUMIRA During Placebo-Controlled Period of Pooled RA Studies (Studies RA-I, RA-II, RA-III, and RA-IV)

	HUMIRA<br>40 mg subcutaneous<br>Every Other Week	Placebo
	(N=705)	(N=690)
Adverse Reaction (Preferred Term)		
**Respiratory**		
Upper respiratory infection	17%	13%
Sinusitis	11%	9%
Flu syndrome	7%	6%
**Gastrointestinal**		
Nausea	9%	8%
Abdominal pain	7%	4%
**Laboratory Tests***		
Laboratory test abnormal	8%	7%
Hypercholesterolemia	6%	4%
Hyperlipidemia	7%	5%

Hematuria	5%	4%
Alkaline phosphatase increased	5%	3%
**Other**		
Headache	12%	8%
Rash	12%	6%
Accidental injury	10%	8%
Injection site reaction **	8%	1%
Back pain	6%	4%
Urinary tract infection	8%	5%
Hypertension	5%	3%
* Laboratory test abnormalities were reported as adverse reactions in European trials<br>** Does not include injection site erythema, itching, hemorrhage, pain or swelling		

Less Common Adverse Reactions in Rheumatoid Arthritis Clinical Studies

Other infrequent serious adverse reactions that do not appear in the Warnings and Precautions or Adverse Reaction sections that occurred at an incidence of less than 5% in HUMIRA-treated patients in RA studies were:

Body As A Whole: Pain in extremity, pelvic pain, surgery, thorax pain

Cardiovascular System: Arrhythmia, atrial fibrillation, chest pain, coronary artery disorder, heart arrest, hypertensive encephalopathy, myocardial infarct, palpitation, pericardial effusion, pericarditis, syncope, tachycardia

Digestive System: Cholecystitis, cholelithiasis, esophagitis, gastroenteritis, gastrointestinal hemorrhage, hepatic necrosis, vomiting

Endocrine System: Parathyroid disorder

Hemic And Lymphatic System: Agranulocytosis, polycythemia

Metabolic And Nutritional Disorders: Dehydration, healing abnormal, ketosis, paraproteinemia, peripheral edema

Musculo-Skeletal System: Arthritis, bone disorder, bone fracture (not spontaneous), bone necrosis, joint disorder, muscle cramps, myasthenia, pyogenic arthritis, synovitis, tendon disorder

Neoplasia: Adenoma

Nervous System: Confusion, paresthesia, subdural hematoma, tremor

Respiratory System: Asthma, bronchospasm, dyspnea, lung function decreased, pleural effusion

Special Senses: Cataract

Thrombosis: Thrombosis leg

Urogenital System: Cystitis, kidney calculus, menstrual disorder

Juvenile Idiopathic Arthritis Clinical Studies

In general, the adverse reactions in the HUMIRA-treated patients in the polyarticular

juvenile idiopathic arthritis (JIA) trials (Studies JIA-I and JIA-II) *[see Clinical Studies (14.2)]* were similar in frequency and type to those seen in adult patients *[see Warnings and Precautions (5), Adverse Reactions (6)]*. Important findings and differences from adults are discussed in the following paragraphs.

In Study JIA-I, HUMIRA was studied in 171 patients who were 4 to 17 years of age, with polyarticular JIA. Severe adverse reactions reported in the study included neutropenia, streptococcal pharyngitis, increased aminotransferases, herpes zoster, myositis, metrorrhagia, and appendicitis. Serious infections were observed in 4% of patients within approximately 2 years of initiation of treatment with HUMIRA and included cases of herpes simplex, pneumonia, urinary tract infection, pharyngitis, and herpes zoster.

In Study JIA-I, 45% of patients experienced an infection while receiving HUMIRA with or without concomitant MTX in the first 16 weeks of treatment. The types of infections reported in HUMIRA-treated patients were generally similar to those commonly seen in polyarticular JIA patients who are not treated with TNF blockers. Upon initiation of treatment, the most common adverse reactions occurring in this patient population treated with HUMIRA were injection site pain and injection site reaction (19% and 16%, respectively). A less commonly reported adverse event in patients receiving HUMIRA was granuloma annulare which did not lead to discontinuation of HUMIRA treatment.

In the first 48 weeks of treatment in Study JIA-I, non-serious hypersensitivity reactions were seen in approximately 6% of patients and included primarily localized allergic hypersensitivity reactions and allergic rash.

In Study JIA-I, 10% of patients treated with HUMIRA who had negative baseline anti-dsDNA antibodies developed positive titers after 48 weeks of treatment. No patient developed clinical signs of autoimmunity during the clinical trial.

Approximately 15% of patients treated with HUMIRA developed mild-to-moderate elevations of creatine phosphokinase (CPK) in Study JIA-I. Elevations exceeding 5 times the upper limit of normal were observed in several patients. CPK concentrations decreased or returned to normal in all patients. Most patients were able to continue HUMIRA without interruption.

In Study JIA-II, HUMIRA was studied in 32 patients who were 2 to <4 years of age or 4 years of age and older weighing <15 kg with polyarticular JIA. The safety profile for this patient population was similar to the safety profile seen in patients 4 to 17 years of age with polyarticular JIA.

In Study JIA-II, 78% of patients experienced an infection while receiving HUMIRA. These included nasopharyngitis, bronchitis, upper respiratory tract infection, otitis media, and were mostly mild to moderate in severity. Serious infections were observed in 9% of patients receiving HUMIRA in the study and included dental caries, rotavirus gastroenteritis, and varicella.

In Study JIA-II, non-serious allergic reactions were observed in 6% of patients and included intermittent urticaria and rash, which were all mild in severity.

Psoriatic Arthritis and Ankylosing Spondylitis Clinical Studies

HUMIRA has been studied in 395 patients with psoriatic arthritis (PsA) in two placebo-controlled trials and in an open label study and in 393 patients with ankylosing spondylitis (AS) in two placebo-controlled studies *[see Clinical Studies (14.3, 14.4)]*. The safety profile for patients with PsA and AS treated with HUMIRA 40 mg every other week

was similar to the safety profile seen in patients with RA, HUMIRA Studies RA-I through IV.

Crohn's Disease Clinical Studies

Adults: The safety profile of HUMIRA in 1478 adult patients with Crohn's disease from four placebo-controlled and two open-label extension studies *[see Clinical Studies (14.5)]* was similar to the safety profile seen in patients with RA.

Pediatric Patients 6 Years to 17 Years: The safety profile of HUMIRA in 192 pediatric patients from one double-blind study (Study PCD-I) and one open-label extension study *[see Clinical Studies (14.6)]* was similar to the safety profile seen in adult patients with Crohn's disease.

During the 4-week open label induction phase of Study PCD-I, the most common adverse reactions occurring in the pediatric population treated with HUMIRA were injection site pain and injection site reaction (6% and 5%, respectively).

A total of 67% of children experienced an infection while receiving HUMIRA in Study PCD-I. These included upper respiratory tract infection and nasopharyngitis.

A total of 5% of children experienced a serious infection while receiving HUMIRA in Study PCD-I. These included viral infection, device related sepsis (catheter), gastroenteritis, H1N1 influenza, and disseminated histoplasmosis.

In Study PCD-I, allergic reactions were observed in 5% of children which were all non-serious and were primarily localized reactions.

Ulcerative Colitis Clinical Studies

Adults: The safety profile of HUMIRA in 1010 adult patients with ulcerative colitis (UC) from two placebo-controlled studies and one open-label extension study *[see Clinical Studies (14.7)]* was similar to the safety profile seen in patients with RA.

Pediatric Patients 5 Years to 17 Years: The safety profile of HUMIRA in 93 pediatric patients with ulcerative colitis from one double-blind study and one open-label extension study *[see Clinical Studies (14.8)]* was similar to the safety profile seen in adult patients with ulcerative colitis.

Plaque Psoriasis Clinical Studies

HUMIRA has been studied in 1696 subjects with plaque psoriasis (Ps) in placebo-controlled and open-label extension studies *[see Clinical Studies (14.9)]*. The safety profile for subjects with Ps treated with HUMIRA was similar to the safety profile seen in subjects with RA with the following exceptions. In the placebo-controlled portions of the clinical trials in Ps subjects, HUMIRA-treated subjects had a higher incidence of arthralgia when compared to controls (3% *vs.* 1%).

Hidradenitis Suppurativa Clinical Studies

HUMIRA has been studied in 727 subjects with hidradenitis suppurativa (HS) in three placebo-controlled studies and one open-label extension study *[see Clinical Studies (14.10)]*. The safety profile for subjects with HS treated with HUMIRA weekly was consistent with the known safety profile of HUMIRA.

Flare of HS, defined as ≥25% increase from baseline in abscesses and inflammatory nodule counts and with a minimum of 2 additional lesions, was documented in 22 (22%)

of the 100 subjects who were withdrawn from HUMIRA treatment following the primary efficacy timepoint in two studies.

<u>Uveitis Clinical Studies</u>

HUMIRA has been studied in 464 adult patients with uveitis (UV) in placebo-controlled and open-label extension studies and in 90 pediatric patients with uveitis (Study PUV-I) *[see Clinical Studies (14.11, 14.12)]* . The safety profile for patients with UV treated with HUMIRA was similar to the safety profile seen in patients with RA.

6.2 Immunogenicity

As with all therapeutic proteins, there is potential for immunogenicity. The detection of antibody formation is highly dependent on the sensitivity and specificity of the assay. Additionally, the observed incidence of antibody (including neutralizing antibody) positivity in an assay may be influenced by several factors including assay methodology, sample handling, timing of sample collection, concomitant medications, and underlying disease. For these reasons, comparison of the incidence of antibodies in the studies described below with the incidence of antibodies in other studies or to other adalimumab products may be misleading.

There are two assays that have been used to measure anti-adalimumab antibodies. With the ELISA, antibodies to adalimumab could be detected only when serum adalimumab concentrations were < 2 mcg/mL. The ECL assay can detect anti-adalimumab antibody titers independent of adalimumab concentrations in the serum samples. The incidence of anti-adalimumab antibody (AAA) development in patients treated with HUMIRA are presented in Table 2.

Table 2: Anti-Adalimumab Antibody Development Determined by ELISA and ECL Assay in Patients Treated with HUMIRA

**Indications**		**Study Duration**	**Anti-Adalimumab Antibody Incidence by ELISA (n/N)**		**Anti-Adalimumab Antibody Incidence by ECL Assay (n/N)**
			In all patients who received adalimumab	In patients with serum adalimumab concentrations < 2 mcg/mL	
Rheumatoid Arthritis[a]		6 to 12 months	5% (58/1062)	NR	NA
Juvenile Idiopathic Arthritis (JIA)	4 to 17 years of age[b]	48 weeks	16% (27/171)	NR	NA
	2 to 4 years of age or ≥ 4 years of age and weighing	24 weeks	7% (1/15)[c]	NR	NA

	< 15 kg				
Psoriatic Arthritis[d]		48 weeks[e]	13% (24/178)	NR	NA
Ankylosing Spondylitis		24 weeks	9% (16/185)	NR	NA
Adult Crohn’s Disease		56 weeks	3% (7/269)	8% (7/86)	NA
Pediatric Crohn’s Disease		52 weeks	3% (6/182)	10% (6/58)	NA
Adult Ulcerative Colitis		52 weeks	5% (19/360)	21% (19/92)	NA
Pediatric Ulcerative Colitis		52 weeks	3% (3/100)	13% (3/23)	33% (33/100)[i]
Plaque Psoriasis[f]		Up to 52 weeks[g]	8% (77/920)	21% (77/372)	NA
Hidradenitis Suppurativa		36 weeks	7% (30/461)	28% (58/207)[h]	61% (272/445)[j]
Non-infectious Uveitis		52 weeks	5% (12/249)	21% (12/57)	40% (99/249)[k]

n: number of patients with anti-adalimumab antibody; NR: not reported; NA: Not applicable (not performed)

[a] In patients receiving concomitant methotrexate (MTX), the incidence of anti-adalimumab antibody was 1% compared to 12% with HUMIRA monotherapy

[b] In patients receiving concomitant MTX, the incidence of anti-adalimumab antibody was 6% compared to 26% with HUMIRA monotherapy

[c] This patient received concomitant MTX

[d] In patients receiving concomitant MTX, the incidence of antibody development was 7% compared to 1% in RA

[e] Subjects enrolled after completing 2 previous studies of 24 weeks or 12 weeks of treatments.

[f] In plaque psoriasis patients who were on HUMIRA monotherapy and subsequently withdrawn from the treatment, the rate of antibodies to adalimumab after retreatment was similar to the rate observed prior to withdrawal

[g] One 12-week Phase 2 study and one 52-week Phase 3 study

[h] Among subjects in the 2 Phase 3 studies who stopped HUMIRA treatment for up to 24 weeks and in whom adalimumab serum levels subsequently declined to <2 mcg/mL (approximately 22% of total subjects studied)

[i] No apparent association between antibody development and safety was observed. The association of antibody development and efficacy outcome was not assessed due to limited number of subjects in each treatment group stratified by anti-adalimumab antibody titer.

[j] No apparent association between antibody development and safety was observed

[k] No correlation of antibody development to safety or efficacy outcomes was observed

Rheumatoid Arthritis and Psoriatic Arthritis: Patients in Studies RA-I, RA-II, and RA-III were tested at multiple time points for antibodies to adalimumab using the ELISA during the 6- to 12-month period. No apparent correlation of antibody development to adverse

reactions was observed. With monotherapy, patients receiving every other week dosing may develop antibodies more frequently than those receiving weekly dosing. In patients receiving the recommended dosage of 40 mg every other week as monotherapy, the ACR 20 response was lower among antibody-positive patients than among antibody-negative patients. The long-term immunogenicity of HUMIRA is unknown.

6.3 Postmarketing Experience

The following adverse reactions have been identified during post-approval use of HUMIRA. Because these reactions are reported voluntarily from a population of uncertain size, it is not always possible to reliably estimate their frequency or establish a causal relationship to HUMIRA exposure.

Gastrointestinal disorders: Diverticulitis, large bowel perforations including perforations associated with diverticulitis and appendiceal perforations associated with appendicitis, pancreatitis

General disorders and administration site conditions: Pyrexia

Hepato-biliary disorders: Liver failure, hepatitis

Immune system disorders: Sarcoidosis

Neoplasms benign, malignant and unspecified (including cysts and polyps): Merkel Cell Carcinoma (neuroendocrine carcinoma of the skin)

Nervous system disorders: Demyelinating disorders (e.g., optic neuritis, Guillain-Barré syndrome), cerebrovascular accident

Respiratory disorders: Interstitial lung disease, including pulmonary fibrosis, pulmonary embolism

Skin reactions: Stevens Johnson Syndrome, cutaneous vasculitis, erythema multiforme, new or worsening psoriasis (all sub-types including pustular and palmoplantar), alopecia, lichenoid skin reaction

Vascular disorders: Systemic vasculitis, deep vein thrombosis

7 DRUG INTERACTIONS

7.1 Methotrexate

HUMIRA has been studied in rheumatoid arthritis (RA) patients taking concomitant methotrexate (MTX). Although MTX reduced the apparent adalimumab clearance, the data do not suggest the need for dose adjustment of either HUMIRA or MTX *[see Clinical Pharmacology (12.3)]*.

7.2 Biological Products

In clinical studies in patients with RA, an increased risk of serious infections has been observed with the combination of TNF blockers with anakinra or abatacept, with no added benefit; therefore, use of HUMIRA with abatacept or anakinra is not recommended in patients with RA *[see Warnings and Precautions (5.7, 5.11)]*. A higher rate of serious infections has also been observed in patients with RA treated with rituximab who received subsequent treatment with a TNF blocker. There is insufficient

information regarding the concomitant use of HUMIRA and other biologic products for the treatment of RA, PsA, AS, CD, UC, Ps, HS and UV. Concomitant administration of HUMIRA with other biologic DMARDS (e.g., anakinra and abatacept) or other TNF blockers is not recommended based upon the possible increased risk for infections and other potential pharmacological interactions.

7.3 Live Vaccines

Avoid the use of live vaccines with HUMIRA *[see Warnings and Precautions (5.10)]*.

7.4 Cytochrome P450 Substrates

The formation of CYP450 enzymes may be suppressed by increased concentrations of cytokines (e.g., TNFα, IL-6) during chronic inflammation. It is possible for a molecule that antagonizes cytokine activity, such as adalimumab, to influence the formation of CYP450 enzymes. Upon initiation or discontinuation of HUMIRA in patients being treated with CYP450 substrates with a narrow therapeutic index, monitoring of the effect (e.g., warfarin) or drug concentration (e.g., cyclosporine or theophylline) is recommended and the individual dose of the drug product may be adjusted as needed.

8 USE IN SPECIFIC POPULATIONS

8.1 Pregnancy

Risk Summary

Available studies with use of adalimumab during pregnancy do not reliably establish an association between adalimumab and major birth defects. Clinical data are available from the Organization of Teratology Information Specialists (OTIS)/MotherToBaby HUMIRA Pregnancy Registry in pregnant women with rheumatoid arthritis (RA) or Crohn’s disease (CD). Registry results showed a rate of 10% for major birth defects with first trimester use of adalimumab in pregnant women with RA or CD and a rate of 7.5% for major birth defects in the disease-matched comparison cohort. The lack of pattern of major birth defects is reassuring and differences between exposure groups may have impacted the occurrence of birth defects *(see Data)*.

Adalimumab is actively transferred across the placenta during the third trimester of pregnancy and may affect immune response in the *in-utero* exposed infant *(see Clinical Considerations)*. In an embryo-fetal perinatal development study conducted in cynomolgus monkeys, no fetal harm or malformations were observed with intravenous administration of adalimumab during organogenesis and later in gestation, at doses that produced exposures up to approximately 373 times the maximum recommended human dose (MRHD) of 40 mg subcutaneous without methotrexate *(see Data)*.

The estimated background risk of major birth defects and miscarriage for the indicated populations is unknown. All pregnancies have a background risk of birth defect, loss, or other adverse outcomes. In the U.S. general population, the estimated background risk of major birth defects and miscarriage in clinically recognized pregnancies is 2-4% and 15-20%, respectively.

Clinical Considerations

Disease-associated maternal and embryo/fetal risk

Published data suggest that the risk of adverse pregnancy outcomes in women with RA or inflammatory bowel disease (IBD) is associated with increased disease activity. Adverse pregnancy outcomes include preterm delivery (before 37 weeks of gestation), low birth weight (less than 2500 g) infants, and small for gestational age at birth.

Fetal/Neonatal Adverse Reactions

Monoclonal antibodies are increasingly transported across the placenta as pregnancy progresses, with the largest amount transferred during the third trimester *(see Data)*. Risks and benefits should be considered prior to administering live or live-attenuated vaccines to infants exposed to HUMIRA *in utero [see Use in Specific Populations (8.4)]*.

<u>Data</u>

Human Data

A prospective cohort pregnancy exposure registry conducted by OTIS/MotherToBaby in the U.S. and Canada between 2004 and 2016 compared the risk of major birth defects in live-born infants of 221 women (69 RA, 152 CD) treated with adalimumab during the first trimester and 106 women (74 RA, 32 CD) not treated with adalimumab.

The proportion of major birth defects among live-born infants in the adalimumab-treated and untreated cohorts was 10% (8.7% RA, 10.5% CD) and 7.5% (6.8% RA, 9.4% CD), respectively. The lack of pattern of major birth defects is reassuring and differences between exposure groups may have impacted the occurrence of birth defects. This study cannot reliably establish whether there is an association between adalimumab and major birth defects because of methodological limitations of the registry, including small sample size, the voluntary nature of the study, and the non-randomized design.

In an independent clinical study conducted in ten pregnant women with IBD treated with HUMIRA, adalimumab concentrations were measured in maternal serum as well as in cord blood (n=10) and infant serum (n=8) on the day of birth. The last dose of HUMIRA was given between 1 and 56 days prior to delivery. Adalimumab concentrations were 0.16-19.7 µg/mL in cord blood, 4.28-17.7 µg/mL in infant serum, and 0-16.1 µg/mL in maternal serum. In all but one case, the cord blood concentration of adalimumab was higher than the maternal serum concentration, suggesting adalimumab actively crosses the placenta. In addition, one infant had serum concentrations at each of the following: 6 weeks (1.94 µg/mL), 7 weeks (1.31 µg/mL), 8 weeks (0.93 µg/mL), and 11 weeks (0.53 µg/mL), suggesting adalimumab can be detected in the serum of infants exposed *in utero* for at least 3 months from birth.

Animal Data

In an embryo-fetal perinatal development study, pregnant cynomolgus monkeys received adalimumab from gestation days 20 to 97 at doses that produced exposures up to 373 times that achieved with the MRHD without methotrexate (on an AUC basis with maternal IV doses up to 100 mg/kg/week). Adalimumab did not elicit harm to the fetuses or malformations.

8.2 Lactation

<u>Risk Summary</u>

Limited data from case reports in the published literature describe the presence of adalimumab in human milk at infant doses of 0.1% to 1% of the maternal serum

concentration. Published data suggest that the systemic exposure to a breastfed infant is expected to be low because adalimumab is a large molecule and is degraded in the gastrointestinal tract. However, the effects of local exposure in the gastrointestinal tract are unknown. There are no reports of adverse effects of adalimumab on the breastfed infant and no effects on milk production. The developmental and health benefits of breastfeeding should be considered along with the mother's clinical need for HUMIRA and any potential adverse effects on the breastfed child from HUMIRA or from the underlying maternal condition.

8.4 Pediatric Use

The safety and effectiveness of HUMIRA have been established for:

- reducing signs and symptoms of moderately to severely active polyarticular JIA in pediatric patients 2 years of age and older.
- the treatment of moderately to severely active Crohn's disease in pediatric patients 6 years of age and older.
- the treatment of moderately to severely active ulcerative colitis in pediatric patients 5 years of age and older.
- the treatment of moderate to severe hidradenitis suppurativa in patients 12 years of age and older.
- the treatment of non-infectious intermediate, posterior, and panuveitis in pediatric patients 2 years of age and older.

Due to its inhibition of TNFα, HUMIRA administered during pregnancy could affect immune response in the *in utero*-exposed newborn and infant. Data from eight infants exposed to HUMIRA *in utero* suggest adalimumab crosses the placenta *[see Use in Specific Populations (8.1)]*. The clinical significance of elevated adalimumab concentrations in infants is unknown. The safety of administering live or live-attenuated vaccines in exposed infants is unknown. Risks and benefits should be considered prior to vaccinating (live or live-attenuated) exposed infants.

Post-marketing cases of lymphoma, including hepatosplenic T-cell lymphoma and other malignancies, some fatal, have been reported among children, adolescents, and young adults who received treatment with TNF-blockers including HUMIRA *[see Warnings and Precautions (5.2)]*.

Juvenile Idiopathic Arthritis

In Study JIA-I, HUMIRA was shown to reduce signs and symptoms of active polyarticular JIA in patients 4 to 17 years of age *[see Clinical Studies (14.2)]*. In Study JIA-II, the safety profile for patients 2 to <4 years of age was similar to the safety profile for patients 4 to 17 years of age with polyarticular JIA *[see Adverse Reactions (6.1)]*. HUMIRA has not been studied in patients with polyarticular JIA less than 2 years of age or in patients with a weight below 10 kg.

The safety of HUMIRA in patients in the polyarticular JIA trials was generally similar to that observed in adults with certain exceptions *[see Adverse Reactions (6.1)]*.

The safety and effectiveness of HUMIRA have not been established in pediatric patients with JIA less than 2 years of age.

Pediatric Crohn's Disease

The safety and effectiveness of HUMIRA for the treatment of moderately to severely

active Crohn's disease have been established in pediatric patients 6 years of age and older. Use of HUMIRA for this indication is supported by evidence from adequate and well-controlled studies in adults with additional data from a randomized, double-blind, 52-week clinical study of two dose concentrations of HUMIRA in 192 pediatric patients (6 years to 17 years of age) *[see Adverse Reactions (6.1), Clinical Pharmacology (12.2, 12.3), Clinical Studies (14.6)]*. The adverse reaction profile in patients 6 years to 17 years of age was similar to adults.

The safety and effectiveness of HUMIRA have not been established in pediatric patients with Crohn's disease less than 6 years of age.

<u>Pediatric Ulcerative Colitis</u>

The safety and effectiveness of HUMIRA for the treatment of moderately to severely active ulcerative colitis have been established in pediatric patients 5 years of age and older. Use of HUMIRA for this indication is supported by evidence from adequate and well-controlled studies in adults with additional data from a randomized, double-blind, 52-week clinical study of two dose concentrations of HUMIRA in 93 pediatric patients (5 years to 17 years of age) *[see Adverse Reactions (6.1), Clinical Pharmacology (12.3), Clinical Studies (14.8)]*. The adverse reaction profile in patients 5 years to 17 years of age was similar to adults.

The effectiveness of HUMIRA has not been established in patients who have lost response or were intolerant to TNF blockers.

The safety and effectiveness of HUMIRA have not been established in pediatric patients with ulcerative colitis less than 5 years of age.

<u>Pediatric Uveitis</u>

The safety and effectiveness of HUMIRA for the treatment of non-infectious uveitis have been established in pediatric patients 2 years of age and older. The use of HUMIRA is supported by evidence from adequate and well-controlled studies of HUMIRA in adults and a 2:1 randomized, controlled clinical study in 90 pediatric patients *[see Clinical Studies (14.12)]*. The safety and effectiveness of HUMIRA have not been established in pediatric patients with uveitis less than 2 years of age.

<u>Hidradenitis Suppurativa</u>

Use of HUMIRA in pediatric patients 12 years of age and older for HS is supported by evidence from adequate and well-controlled studies of HUMIRA in adult HS patients. Additional population pharmacokinetic modeling and simulation predicted that weight-based dosing of HUMIRA in pediatric patients 12 years of age and older can provide generally similar exposure to adult HS patients. The course of HS is sufficiently similar in adult and adolescent patients to allow extrapolation of data from adult to adolescent patients. The recommended dosage in pediatric patients 12 years of age or older is based on body weight *[see Dosage and Administration (2.6), Clinical Pharmacology (12.3), and Clinical Studies (14.10)]*.

The safety and effectiveness of HUMIRA have not been established in patients less than 12 years of age with HS.

8.5 Geriatric Use

A total of 519 RA patients 65 years of age and older, including 107 patients 75 years of

age and older, received HUMIRA in clinical studies RA-I through IV. No overall difference in effectiveness was observed between these patients and younger patients. The frequency of serious infection and malignancy among HUMIRA treated patients 65 years of age and older was higher than for those less than 65 years of age. Consider the benefits and risks of HUMIRA in patients 65 years of age and older. In patients treated with HUMIRA, closely monitor for the development of infection or malignancy *[see Warnings and Precautions (5.1, 5.2)]*.

10 OVERDOSAGE

Doses up to 10 mg/kg have been administered to patients in clinical trials without evidence of dose-limiting toxicities. In case of overdosage, it is recommended that the patient be monitored for any signs or symptoms of adverse reactions or effects and appropriate symptomatic treatment instituted immediately.

11 DESCRIPTION

Adalimumab is a tumor necrosis factor blocker. Adalimumab is a recombinant human IgG1 monoclonal antibody created using phage display technology resulting in an antibody with human derived heavy and light chain variable regions and human IgG1:k constant regions. Adalimumab is produced by recombinant DNA technology in a mammalian cell (Chinese Hamster Ovary (CHO)) expression system and is purified by a process that includes specific viral inactivation and removal steps. It consists of 1330 amino acids and has a molecular weight of approximately 148 kilodaltons.

HUMIRA (adalimumab) injection is supplied as a sterile, preservative-free solution for subcutaneous administration. The drug product is supplied as either a single-dose, prefilled pen (HUMIRA Pen), as a single-dose, 1 mL prefilled glass syringe, or as a single-dose institutional use vial. Enclosed within the pen is a single-dose, 1 mL prefilled glass syringe. The solution of HUMIRA is clear and colorless, with a pH of about 5.2.

Each 80 mg/0.8 mL prefilled syringe or prefilled pen delivers 0.8 mL (80 mg) of drug product. Each 0.8 mL of HUMIRA contains adalimumab (80 mg), mannitol (33.6 mg), polysorbate 80 (0.8 mg), and Water for Injection, USP.

Each 40 mg/0.4 mL prefilled syringe or prefilled pen delivers 0.4 mL (40 mg) of drug product. Each 0.4 mL of HUMIRA contains adalimumab (40 mg), mannitol (16.8 mg), polysorbate 80 (0.4 mg), and Water for Injection, USP.

Each 40 mg/0.8 mL prefilled syringe, prefilled pen, or single-dose institutional use vial delivers 0.8 mL (40 mg) of drug product. Each 0.8 mL of HUMIRA contains adalimumab (40 mg), citric acid monohydrate (1.04 mg), dibasic sodium phosphate dihydrate (1.22 mg), mannitol (9.6 mg), monobasic sodium phosphate dihydrate (0.69 mg), polysorbate 80 (0.8 mg), sodium chloride (4.93 mg), sodium citrate (0.24 mg) and Water for Injection, USP. Sodium hydroxide is added as necessary to adjust pH.

Each 20 mg/0.2 mL prefilled syringe delivers 0.2 mL (20 mg) of drug product. Each 0.2 mL of HUMIRA contains adalimumab (20 mg), mannitol (8.4 mg), polysorbate 80 (0.2 mg), and Water for Injection, USP.

Each 20 mg/0.4 mL prefilled syringe delivers 0.4 mL (20 mg) of drug product. Each 0.4 mL of HUMIRA contains adalimumab (20 mg), citric acid monohydrate (0.52 mg), dibasic

sodium phosphate dihydrate (0.61 mg), mannitol (4.8 mg), monobasic sodium phosphate dihydrate (0.34 mg), polysorbate 80 (0.4 mg), sodium chloride (2.47 mg), sodium citrate (0.12 mg) and Water for Injection, USP. Sodium hydroxide is added as necessary to adjust pH.

Each 10 mg/0.1 mL prefilled syringe delivers 0.1 mL (10 mg) of drug product. Each 0.1 mL of HUMIRA contains adalimumab (10 mg), mannitol (4.2 mg), polysorbate 80 (0.1 mg), and Water for Injection, USP.

Each 10 mg/0.2 mL prefilled syringe delivers 0.2 mL (10 mg) of drug product. Each 0.2 mL of HUMIRA contains adalimumab (10 mg), citric acid monohydrate (0.26 mg), dibasic sodium phosphate dihydrate (0.31 mg), mannitol (2.4 mg), monobasic sodium phosphate dihydrate (0.17 mg), polysorbate 80 (0.2 mg), sodium chloride (1.23 mg), sodium citrate (0.06 mg) and Water for Injection, USP. Sodium hydroxide is added as necessary to adjust pH.

12 CLINICAL PHARMACOLOGY

12.1 Mechanism of Action

Adalimumab binds specifically to TNF-alpha and blocks its interaction with the p55 and p75 cell surface TNF receptors. Adalimumab also lyses surface TNF expressing cells *in vitro* in the presence of complement. Adalimumab does not bind or inactivate lymphotoxin (TNF-beta). TNF is a naturally occurring cytokine that is involved in normal inflammatory and immune responses. Elevated concentrations of TNF are found in the synovial fluid of patients with RA, JIA, PsA, and AS and play an important role in both the pathologic inflammation and the joint destruction that are hallmarks of these diseases. Increased concentrations of TNF are also found in psoriasis plaques. In Ps, treatment with HUMIRA may reduce the epidermal thickness and infiltration of inflammatory cells. The relationship between these pharmacodynamic activities and the mechanism(s) by which HUMIRA exerts its clinical effects is unknown.

Adalimumab also modulates biological responses that are induced or regulated by TNF, including changes in the concentrations of adhesion molecules responsible for leukocyte migration (ELAM-1, VCAM-1, and ICAM-1 with an IC_{50} of 1-2 X 10^{-10}M).

12.2 Pharmacodynamics

After treatment with HUMIRA, a decrease in concentrations of acute phase reactants of inflammation (C-reactive protein [CRP] and erythrocyte sedimentation rate [ESR]) and serum cytokines (IL-6) was observed compared to baseline in patients with rheumatoid arthritis. A decrease in CRP concentrations was also observed in patients with Crohn’s disease, ulcerative colitis and hidradenitis suppurativa. Serum concentrations of matrix metalloproteinases (MMP-1 and MMP-3) that produce tissue remodeling responsible for cartilage destruction were also decreased after HUMIRA administration.

For pediatric patients 5 years to 17 years with ulcerative colitis, the recommended dosage of HUMIRA is based on modeled dose/exposure-efficacy relationships and pharmacokinetic data. There are no anticipated clinically relevant differences in efficacy between the studied higher dosage administered in the clinical trial (Weeks 0 to 52 in Study PUC-I) *[see Clinical Studies (14.8)]* and the recommended dosage *[see Dosage and Administration (2.4)].*

12.3 Pharmacokinetics

The pharmacokinetics of adalimumab were linear over the dose range of 0.5 to 10 mg/kg following administration of a single intravenous dose (HUMIRA is not approved for intravenous use). Following 20, 40, and 80 mg every other week and every week subcutaneous administration, adalimumab mean serum trough concentrations at steady state increased approximately proportionally with dose in RA patients. The mean terminal half-life was approximately 2 weeks, ranging from 10 to 20 days across studies. Healthy subjects and patients with RA displayed similar adalimumab pharmacokinetics.

Adalimumab exposure in patients treated with 80 mg every other week is estimated to be comparable with that in patients treated with 40 mg every week.

Absorption

The average absolute bioavailability of adalimumab following a single 40 mg subcutaneous dose was 64%. The mean time to reach the maximum concentration was 5.5 days (131 ± 56 hours) and the maximum serum concentration was 4.7 ± 1.6 mcg/mL in healthy subjects following a single 40 mg subcutaneous administration of HUMIRA.

Distribution

The distribution volume (V_{ss}) ranged from 4.7 to 6.0 L following intravenous administration of doses ranging from 0.25 to 10 mg/kg in RA patients.

Elimination

The single dose pharmacokinetics of adalimumab in RA patients were determined in several studies with intravenous doses ranging from 0.25 to 10 mg/kg. The systemic clearance of adalimumab is approximately 12 mL/hr. In long-term studies with dosing more than two years, there was no evidence of changes in clearance over time in RA patients.

Patient Population

Rheumatoid Arthritis and Ankylosing Spondylitis: In patients receiving 40 mg HUMIRA every other week, adalimumab mean steady-state trough concentrations were approximately 5 mcg/mL and 8 to 9 mcg/mL, without and with MTX concomitant treatment, respectively. Adalimumab concentrations in the synovial fluid from five rheumatoid arthritis patients ranged from 31 to 96% of those in serum. The pharmacokinetics of adalimumab in patients with AS were similar to those in patients with RA.

Psoriatic Arthritis: In patients receiving 40 mg every other week, adalimumab mean steady-state trough concentrations were 6 to 10 mcg/mL and 8.5 to 12 mcg/mL, without and with MTX concomitant treatment, respectively.

Plaque Psoriasis: Adalimumab mean steady-state trough concentration was approximately 5 to 6 mcg/mL during HUMIRA 40 mg every other week treatment.

Adult Uveitis: Adalimumab mean steady concentration was approximately 8 to 10 mcg/mL during HUMIRA 40 mg every other week treatment.

Adult Hidradenitis Suppurativa: Adalimumab trough concentrations were approximately 7 to 8 mcg/mL at Week 2 and Week 4, respectively, after receiving 160 mg on Week 0

followed by 80 mg on Week 2. Mean steady-state trough concentrations at Week 12 through Week 36 were approximately 7 to 11 mcg/mL during HUMIRA 40 mg every week treatment.

Adult Crohn's Disease: Adalimumab mean trough concentrations were approximately 12 mcg/mL at Week 2 and Week 4 after receiving 160 mg on Week 0 followed by 80 mg on Week 2. Mean steady-state trough concentrations were 7 mcg/mL at Week 24 and Week 56 during HUMIRA 40 mg every other week treatment.

Adult Ulcerative Colitis: Adalimumab mean trough concentrations were approximately 12 mcg/mL at Week 2 and Week 4 after receiving 160 mg on Week 0 followed by 80 mg on Week 2. Mean steady-state trough concentrations were approximately 8 mcg/mL and 15 mcg/mL at Week 52 after receiving a dose of HUMIRA 40 mg every other week and 40 mg every week, respectively.

<u>Anti-Drug Antibody Effects on Pharmacokinetics</u>

Rheumatoid Arthritis: A trend toward higher apparent clearance of adalimumab in the presence of anti-adalimumab antibodies was identified.

Pediatric Ulcerative Colitis: Antibodies to adalimumab by ECL assay were associated with reduced serum adalimumab concentrations in pediatric patients with moderately to severely active ulcerative colitis.

Hidradenitis Suppurativa: In subjects with moderate to severe HS, antibodies to adalimumab were associated with reduced serum adalimumab concentrations. In general, the extent of reduction in serum adalimumab concentrations is greater with increasing titers of antibodies to adalimumab.

<u>Specific Populations</u>

Geriatric Patients: A lower clearance with increasing age was observed in patients with RA aged 40 to >75 years.

Pediatric Patients:

<u>Juvenile Idiopathic Arthritis</u>:

- *4 years to 17 years of age:* The adalimumab mean steady-state trough concentrations were 6.8 mcg/mL and 10.9 mcg/mL in patients weighing <30 kg receiving 20 mg HUMIRA subcutaneously every other week as monotherapy or with concomitant MTX, respectively. The adalimumab mean steady-state trough concentrations were 6.6 mcg/mL and 8.1 mcg/mL in patients weighing ≥30 kg receiving 40 mg HUMIRA subcutaneously every other week as monotherapy or with MTX concomitant treatment, respectively.
- *2 years to <4 years of age or 4 years of age and older weighing <15 kg:* The adalimumab mean steady-state trough adalimumab concentrations were 6.0 mcg/mL and 7.9 mcg/mL in patients receiving HUMIRA subcutaneously every other week as monotherapy or with MTX concomitant treatment, respectively.

<u>Pediatric Hidradenitis Suppurativa</u>: Adalimumab concentrations in adolescent patients with HS receiving the recommended dosage regimens are predicted to be similar to those observed in adult subjects with HS based on population pharmacokinetic modeling and simulation.

<u>Pediatric Crohn's Disease</u>: Adalimumab mean ± SD concentrations were 15.7±6.5

mcg/mL at Week 4 following 160 mg at Week 0 and 80 mg at Week 2, and 10.5±6.0 mcg/mL at Week 52 following 40 mg every other week dosing in patients weighing ≥ 40 kg. Adalimumab mean ± SD concentrations were 10.6±6.1 mcg/mL at Week 4 following dosing 80 mg at Week 0 and 40 mg at Week 2, and 6.9±3.6 mcg/mL at Week 52 following 20 mg every other week dosing in patients weighing < 40 kg.

Pediatric Ulcerative Colitis: The adalimumab mean steady-state trough concentration was 5.0±3.3 mcg/mL at Week 52 following subcutaneous administration of 0.6 mg/kg (maximum of 40 mg) every other week in pediatric UC patients 5 years to 17 years of age. In patients who received 0.6 mg/kg (maximum of 40 mg) every week, the mean steady-state trough concentration was 15.7±5.6 mcg/mL at Week 52 in pediatric UC patients 5 years to 17 years of age.

Male and Female Patients: No gender-related pharmacokinetic differences were observed after correction for a patient's body weight. Healthy subjects and patients with rheumatoid arthritis displayed similar adalimumab pharmacokinetics.

Patients with Renal or Hepatic Impairment: No pharmacokinetic data are available in patients with hepatic or renal impairment.

Rheumatoid factor or CRP concentrations: Minor increases in apparent clearance were predicted in RA patients receiving doses lower than the recommended dose and in RA patients with high rheumatoid factor or CRP concentrations. These increases are not likely to be clinically important.

Drug Interaction Studies:

Methotrexate: MTX reduced adalimumab apparent clearance after single and multiple dosing by 29% and 44% respectively, in patients with RA *[see Drug Interactions (7.1)]*.

13 NONCLINICAL TOXICOLOGY

13.1 Carcinogenesis, Mutagenesis, Impairment of Fertility

Long-term animal studies of HUMIRA have not been conducted to evaluate the carcinogenic potential or its effect on fertility.

14 CLINICAL STUDIES

14.1 Rheumatoid Arthritis

The efficacy and safety of HUMIRA were assessed in five randomized, double-blind studies in patients ≥18 years of age with active rheumatoid arthritis (RA) diagnosed according to American College of Rheumatology (ACR) criteria. Patients had at least 6 swollen and 9 tender joints. HUMIRA was administered subcutaneously in combination with methotrexate (MTX) (12.5 to 25 mg, Studies RA-I, RA-III and RA-V) or as monotherapy (Studies RA-II and RA-V) or with other disease-modifying anti-rheumatic drugs (DMARDs) (Study RA-IV).

Study RA-I evaluated 271 patients who had failed therapy with at least one but no more than four DMARDs and had inadequate response to MTX. Doses of 20, 40 or 80 mg of HUMIRA or placebo were given every other week for 24 weeks.

Study RA-II evaluated 544 patients who had failed therapy with at least one DMARD. Doses of placebo, 20 or 40 mg of HUMIRA were given as monotherapy every other week or weekly for 26 weeks.

Study RA-III evaluated 619 patients who had an inadequate response to MTX. Patients received placebo, 40 mg of HUMIRA every other week with placebo injections on alternate weeks, or 20 mg of HUMIRA weekly for up to 52 weeks. Study RA-III had an additional primary endpoint at 52 weeks of inhibition of disease progression (as detected by X-ray results). Upon completion of the first 52 weeks, 457 patients enrolled in an open-label extension phase in which 40 mg of HUMIRA was administered every other week for up to 5 years.

Study RA-IV assessed safety in 636 patients who were either DMARD-naive or were permitted to remain on their pre-existing rheumatologic therapy provided that therapy was stable for a minimum of 28 days. Patients were randomized to 40 mg of HUMIRA or placebo every other week for 24 weeks.

Study RA-V evaluated 799 patients with moderately to severely active RA of less than 3 years duration who were ≥18 years old and MTX naïve. Patients were randomized to receive either MTX (optimized to 20 mg/week by week 8), HUMIRA 40 mg every other week or HUMIRA/MTX combination therapy for 104 weeks. Patients were evaluated for signs and symptoms, and for radiographic progression of joint damage. The median disease duration among patients enrolled in the study was 5 months. The median MTX dose achieved was 20 mg.

Clinical Response

The percent of HUMIRA treated patients achieving ACR 20, 50 and 70 responses in Studies RA-II and III are shown in Table 3.

Table 3. ACR Responses in Studies RA-II and RA-III (Percent of Patients)

	**Study RA-II Monotherapy (26 weeks)**			**Study RA-III Methotrexate Combination (24 and 52 weeks)**	
Response	Placebo	HUMIRA	HUMIRA	Placebo/MTX	HUMIRA/MTX
		40 mg every	40 mg weekly		40 mg every
		other week			other week
	N=110	N=113	N=103	N=200	N=207
**ACR20**					
Month 6	19%	46%*	53%*	30%	63%*
Month 12	NA	NA	NA	24%	59%*
**ACR50**					
Month 6	8%	22%*	35%*	10%	39%*
Month 12	NA	NA	NA	10%	42%*
**ACR70**					
Month 6	2%	12%*	18%*	3%	21%*
Month 12	NA	NA	NA	5%	23%*
* p<0.01, HUMIRA *vs.* placebo					

The results of Study RA-I were similar to Study RA-III; patients receiving HUMIRA 40 mg every other week in Study RA-I also achieved ACR 20, 50 and 70 response rates of 65%, 52% and 24%, respectively, compared to placebo responses of 13%, 7% and 3% respectively, at 6 months ($p<0.01$).

The results of the components of the ACR response criteria for Studies RA-II and RA-III are shown in Table 4. ACR response rates and improvement in all components of ACR response were maintained to week 104. Over the 2 years in Study RA-III, 20% of HUMIRA patients receiving 40 mg every other week achieved a major clinical response, defined as maintenance of an ACR 70 response over a 6-month period. ACR responses were maintained in similar proportions of patients for up to 5 years with continuous HUMIRA treatment in the open-label portion of Study RA-III.

Table 4. Components of ACR Response in Studies RA-II and RA-III

	**Study RA-II**				**Study RA-III**			
**Parameter (median)**	**Placebo N=110**		**HUMIRA[a] N=113**		**Placebo/MTX N=200**		**HUMIRA[a]/MTX N=207**	
	**Baseline**	**Wk 26**	**Baseline**	**Wk 26**	**Baseline**	**Wk 24**	**Baseline**	**Wk 24**
Number of tender joints (0-68)	35	26	31	16*	26	15	24	8*
Number of swollen joints (0-66)	19	16	18	10*	17	11	18	5*
Physician global assessment[b]	7.0	6.1	6.6	3.7*	6.3	3.5	6.5	2.0*
Patient global assessment[b]	7.5	6.3	7.5	4.5*	5.4	3.9	5.2	2.0*
Pain[b]	7.3	6.1	7.3	4.1*	6.0	3.8	5.8	2.1*
Disability index (HAQ)[c]	2.0	1.9	1.9	1.5*	1.5	1.3	1.5	0.8*
CRP (mg/dL)	3.9	4.3	4.6	1.8*	1.0	0.9	1.0	0.4*

[a] 40 mg HUMIRA administered every other week
[b] Visual analogue scale; 0 = best, 10 = worst
[c] Disability Index of the Health Assessment Questionnaire; 0 = best, 3 = worst, measures the patient’s ability to perform the following: dress/groom, arise, eat, walk, reach, grip, maintain hygiene, and maintain daily activity
* $p<0.001$, HUMIRA *vs.* placebo, based on mean change from baseline

The time course of ACR 20 response for Study RA-III is shown in Figure 1.

In Study RA-III, 85% of patients with ACR 20 responses at week 24 maintained the response at 52 weeks. The time course of ACR 20 response for Study RA-I and Study RA-II were similar.

Figure 1. Study RA-III ACR 20 Responses over 52 Weeks

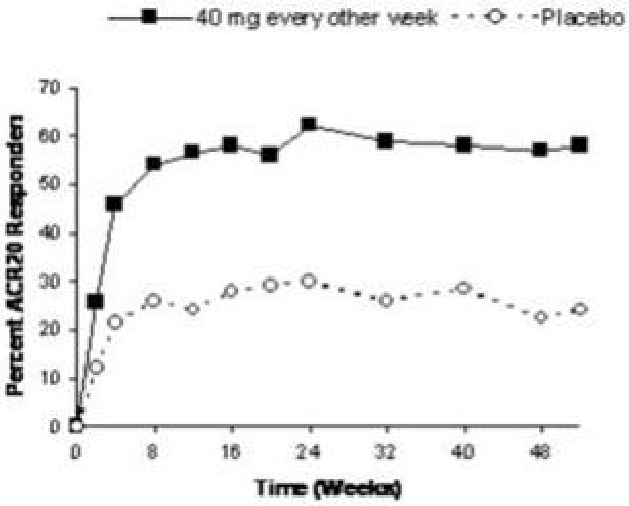

In Study RA-IV, 53% of patients treated with HUMIRA 40 mg every other week plus standard of care had an ACR 20 response at week 24 compared to 35% on placebo plus standard of care ($p<0.001$). No unique adverse reactions related to the combination of HUMIRA (adalimumab) and other DMARDs were observed.

In Study RA-V with MTX naïve patients with recent onset RA, the combination treatment with HUMIRA plus MTX led to greater percentages of patients achieving ACR responses than either MTX monotherapy or HUMIRA monotherapy at Week 52 and responses were sustained at Week 104 (see Table 5).

Table 5. ACR Response in Study RA-V (Percent of Patients)

Response	MTX[b] N=257	HUMIRA[c] N=274	HUMIRA/MTX N=268
**ACR20**			
Week 52	63%	54%	73%
Week 104	56%	49%	69%
**ACR50**			
Week 52	46%	41%	62%
Week 104	43%	37%	59%
**ACR70**			
Week 52	27%	26%	46%
Week 104	28%	28%	47%
Major Clinical Response [a]	28%	25%	49%

[a] Major clinical response is defined as achieving an ACR70 response for a continuous six month period
[b] $p<0.05$, HUMIRA/MTX *vs.* MTX for ACR 20
$p<0.001$, HUMIRA/MTX *vs.* MTX for ACR 50 and 70, and Major Clinical Response
[c] $p<0.001$, HUMIRA/MTX *vs.* HUMIRA

At Week 52, all individual components of the ACR response criteria for Study RA-V improved in the HUMIRA/MTX group and improvements were maintained to Week 104.

Radiographic Response

In Study RA-III, structural joint damage was assessed radiographically and expressed as change in Total Sharp Score (TSS) and its components, the erosion score and Joint Space Narrowing (JSN) score, at month 12 compared to baseline. At baseline, the median TSS was approximately 55 in the placebo and 40 mg every other week groups. The results are shown in Table 6. HUMIRA/MTX treated patients demonstrated less radiographic progression than patients receiving MTX alone at 52 weeks.

Table 6. Radiographic Mean Changes Over 12 Months in Study RA-III

	Placebo/MTX	HUMIRA/MTX 40 mg every other week	Placebo/MTX-HUMIRA/MTX (95% Confidence Interval*)	P-value**
Total Sharp score	2.7	0.1	2.6 (1.4, 3.8)	<0.001
Erosion score	1.6	0.0	1.6 (0.9, 2.2)	<0.001
JSN score	1.0	0.1	0.9 (0.3, 1.4)	0.002

*95% confidence intervals for the differences in change scores between MTX and HUMIRA.
**Based on rank analysis

In the open-label extension of Study RA-III, 77% of the original patients treated with any dose of HUMIRA were evaluated radiographically at 2 years. Patients maintained inhibition of structural damage, as measured by the TSS. Fifty-four percent had no progression of structural damage as defined by a change in the TSS of zero or less. Fifty-five percent (55%) of patients originally treated with 40 mg HUMIRA every other week have been evaluated radiographically at 5 years. Patients had continued inhibition of structural damage with 50% showing no progression of structural damage defined by a change in the TSS of zero or less.

In Study RA-V, structural joint damage was assessed as in Study RA-III. Greater inhibition of radiographic progression, as assessed by changes in TSS, erosion score and JSN was observed in the HUMIRA/MTX combination group as compared to either the MTX or HUMIRA monotherapy group at Week 52 as well as at Week 104 (see Table 7).

Table 7. Radiographic Mean Change* in Study RA-V

		**MTX[a] N=257**	**HUMIRA[a,b] N=274**	**HUMIRA/MTX N=268**
52 Weeks	Total Sharp score	5.7 (4.2, 7.3)	3.0 (1.7, 4.3)	1.3 (0.5, 2.1)
	Erosion score	3.7 (2.7, 4.8)	1.7 (1.0, 2.4)	0.8 (0.4, 1.2)
	JSN score	2.0 (1.2, 2.8)	1.3 (0.5, 2.1)	0.5 (0.0, 1.0)
104 Weeks	Total Sharp score	10.4 (7.7, 13.2)	5.5 (3.6, 7.4)	1.9 (0.9, 2.9)
	Erosion score	6.4 (4.6, 8.2)	3.0 (2.0, 4.0)	1.0 (0.4, 1.6)
	JSN score	4.1 (2.7, 5.4)	2.6 (1.5, 3.7)	0.9 (0.3, 1.5)

* mean (95% confidence interval)
[a] p<0.001, HUMIRA/MTX *vs.* MTX at 52 and 104 weeks and for HUMIRA/MTX *vs.* HUMIRA at 104 weeks
[b] p<0.01, for HUMIRA/MTX *vs.* HUMIRA at 52 weeks

Physical Function Response

In studies RA-I through IV, HUMIRA showed significantly greater improvement than placebo in the disability index of Health Assessment Questionnaire (HAQ-DI) from baseline to the end of study, and significantly greater improvement than placebo in the health-outcomes as assessed by The Short Form Health Survey (SF 36). Improvement was seen in both the Physical Component Summary (PCS) and the Mental Component Summary (MCS).

In Study RA-III, the mean (95% CI) improvement in HAQ-DI from baseline at week 52 was 0.60 (0.55, 0.65) for the HUMIRA patients and 0.25 (0.17, 0.33) for placebo/MTX ($p<0.001$) patients. Sixty-three percent of HUMIRA-treated patients achieved a 0.5 or greater improvement in HAQ-DI at week 52 in the double-blind portion of the study. Eighty-two percent of these patients maintained that improvement through week 104 and a similar proportion of patients maintained this response through week 260 (5 years) of open-label treatment. Mean improvement in the SF-36 was maintained through the end of measurement at week 156 (3 years).

In Study RA-V, the HAQ-DI and the physical component of the SF-36 showed greater improvement ($p<0.001$) for the HUMIRA/MTX combination therapy group versus either the MTX monotherapy or the HUMIRA monotherapy group at Week 52, which was maintained through Week 104.

14.2 Juvenile Idiopathic Arthritis

The safety and efficacy of HUMIRA was assessed in two studies (Studies JIA-I and JIA-II) in patients with active polyarticular juvenile idiopathic arthritis (JIA).

Study JIA-I

The safety and efficacy of HUMIRA were assessed in a multicenter, randomized, withdrawal, double-blind, parallel-group study in 171 patients who were 4 to 17 years of age with polyarticular JIA. In the study, the patients were stratified into two groups: MTX-treated or non-MTX-treated. All patients had to show signs of active moderate or severe disease despite previous treatment with NSAIDs, analgesics, corticosteroids, or DMARDS. Patients who received prior treatment with any biologic DMARDS were excluded from the study.

The study included four phases: an open-label lead in phase (OL-LI; 16 weeks), a double-blind randomized withdrawal phase (DB; 32 weeks), an open-label extension phase (OLE-BSA; up to 136 weeks), and an open-label fixed dose phase (OLE-FD; 16 weeks). In the first three phases of the study, HUMIRA was administered based on body surface area at a dose of 24 mg/m^2 up to a maximum total body dose of 40 mg subcutaneously (SC) every other week. In the OLE-FD phase, the patients were treated with 20 mg of HUMIRA SC every other week if their weight was less than 30 kg and with 40 mg of HUMIRA SC every other week if their weight was 30 kg or greater. Patients remained on stable doses of NSAIDs and or prednisone (≤0.2 mg/kg/day or 10 mg/day maximum).

Patients demonstrating a Pediatric ACR 30 response at the end of OL-LI phase were randomized into the double blind (DB) phase of the study and received either HUMIRA or placebo every other week for 32 weeks or until disease flare. Disease flare was defined as a worsening of ≥30% from baseline in ≥3 of 6 Pediatric ACR core criteria, ≥2 active joints, and improvement of >30% in no more than 1 of the 6 criteria. After 32 weeks or

at the time of disease flare during the DB phase, patients were treated in the open-label extension phase based on the BSA regimen (OLE-BSA), before converting to a fixed dose regimen based on body weight (OLE-FD phase).

Study JIA-I Clinical Response

At the end of the 16-week OL-LI phase, 94% of the patients in the MTX stratum and 74% of the patients in the non-MTX stratum were Pediatric ACR 30 responders. In the DB phase significantly fewer patients who received HUMIRA experienced disease flare compared to placebo, both without MTX (43% *vs.* 71%) and with MTX (37% *vs.* 65%). More patients treated with HUMIRA continued to show pediatric ACR 30/50/70 responses at Week 48 compared to patients treated with placebo. Pediatric ACR responses were maintained for up to two years in the OLE phase in patients who received HUMIRA throughout the study.

Study JIA-II

HUMIRA was assessed in an open-label, multicenter study in 32 patients who were 2 to <4 years of age or 4 years of age and older weighing <15 kg with moderately to severely active polyarticular JIA. Most patients (97%) received at least 24 weeks of HUMIRA treatment dosed 24 mg/m^2 up to a maximum of 20 mg every other week as a single SC injection up to a maximum of 120 weeks duration. During the study, most patients used concomitant MTX, with fewer reporting use of corticosteroids or NSAIDs. The primary objective of the study was evaluation of safety *[see Adverse Reactions (6.1)]*.

14.3 Psoriatic Arthritis

The safety and efficacy of HUMIRA was assessed in two randomized, double-blind, placebo controlled studies in 413 patients with psoriatic arthritis (PsA). Upon completion of both studies, 383 patients enrolled in an open-label extension study, in which 40 mg HUMIRA was administered every other week.

Study PsA-I enrolled 313 adult patients with moderately to severely active PsA (>3 swollen and >3 tender joints) who had an inadequate response to NSAID therapy in one of the following forms: (1) distal interphalangeal (DIP) involvement (N=23); (2) polyarticular arthritis (absence of rheumatoid nodules and presence of plaque psoriasis) (N=210); (3) arthritis mutilans (N=1); (4) asymmetric PsA (N=77); or (5) AS-like (N=2). Patients on MTX therapy (158 of 313 patients) at enrollment (stable dose of ≤30 mg/week for >1 month) could continue MTX at the same dose. Doses of HUMIRA 40 mg or placebo every other week were administered during the 24-week double-blind period of the study.

Compared to placebo, treatment with HUMIRA resulted in improvements in the measures of disease activity (see Tables 8 and 9). Among patients with PsA who received HUMIRA, the clinical responses were apparent in some patients at the time of the first visit (two weeks) and were maintained up to 88 weeks in the ongoing open-label study. Similar responses were seen in patients with each of the subtypes of psoriatic arthritis, although few patients were enrolled with the arthritis mutilans and ankylosing spondylitis-like subtypes. Responses were similar in patients who were or were not receiving concomitant MTX therapy at baseline.

Patients with psoriatic involvement of at least three percent body surface area (BSA) were evaluated for Psoriatic Area and Severity Index (PASI) responses. At 24 weeks, the

proportions of patients achieving a 75% or 90% improvement in the PASI were 59% and 42% respectively, in the HUMIRA group (N=69), compared to 1% and 0% respectively, in the placebo group (N=69) (p<0.001). PASI responses were apparent in some patients at the time of the first visit (two weeks). Responses were similar in patients who were or were not receiving concomitant MTX therapy at baseline.

Table 8. ACR Response in Study PsA-I (Percent of Patients)

	Placebo N=162	HUMIRA* N=151
**ACR20**		
Week 12	14%	58%
Week 24	15%	57%
**ACR50**		
Week 12	4%	36%
Week 24	6%	39%
**ACR70**		
Week 12	1%	20%
Week 24	1%	23%

* p<0.001 for all comparisons between HUMIRA and placebo

Table 9. Components of Disease Activity in Study PsA-I

	Placebo N=162		HUMIRA* N=151	
Parameter: median	Baseline	24 weeks	Baseline	24 weeks
Number of tender joints[a]	23.0	17.0	20.0	5.0
Number of swollen joints[b]	11.0	9.0	11.0	3.0
Physician global assessment[c]	53.0	49.0	55.0	16.0
Patient global assessment[c]	49.5	49.0	48.0	20.0
Pain[c]	49.0	49.0	54.0	20.0
Disability index (HAQ)[d]	1.0	0.9	1.0	0.4
CRP (mg/dL)[e]	0.8	0.7	0.8	0.2

* p<0.001 for HUMIRA *vs.* placebo comparisons based on median changes
[a] Scale 0-78
[b] Scale 0-76
[c] Visual analog scale; 0=best, 100=worst
[d] Disability Index of the Health Assessment Questionnaire; 0=best, 3=worst; measures the patient's ability to perform the following: dress/groom, arise, eat, walk, reach, grip, maintain hygiene, and maintain daily activity.
[e] Normal range: 0-0.287 mg/dL

Similar results were seen in an additional, 12-week study in 100 patients with moderate to severe psoriatic arthritis who had suboptimal response to DMARD therapy as manifested by ≥3 tender joints and ≥3 swollen joints at enrollment.

<u>Radiographic Response</u>

Radiographic changes were assessed in the PsA studies. Radiographs of hands, wrists,

and feet were obtained at baseline and Week 24 during the double-blind period when patients were on HUMIRA or placebo and at Week 48 when all patients were on open-label HUMIRA. A modified Total Sharp Score (mTSS), which included distal interphalangeal joints (i.e., not identical to the TSS used for rheumatoid arthritis), was used by readers blinded to treatment group to assess the radiographs.

HUMIRA-treated patients demonstrated greater inhibition of radiographic progression compared to placebo-treated patients and this effect was maintained at 48 weeks (see Table 10).

Table 10. Change in Modified Total Sharp Score in Psoriatic Arthritis

	**Placebo N=141**	**HUMIRA N=133**	
	Week 24	Week 24	Week 48
Baseline mean	22.1	23.4	23.4
Mean Change ± SD	0.9 ± 3.1	-0.1 ± 1.7	-0.2 ± 4.9*
* <0.001 for the difference between HUMIRA, Week 48 and Placebo, Week 24 (primary analysis)			

<u>Physical Function Response</u>

In Study PsA-I, physical function and disability were assessed using the HAQ Disability Index (HAQ-DI) and the SF-36 Health Survey. Patients treated with 40 mg of HUMIRA every other week showed greater improvement from baseline in the HAQ-DI score (mean decreases of 47% and 49% at Weeks 12 and 24 respectively) in comparison to placebo (mean decreases of 1% and 3% at Weeks 12 and 24 respectively). At Weeks 12 and 24, patients treated with HUMIRA showed greater improvement from baseline in the SF-36 Physical Component Summary score compared to patients treated with placebo, and no worsening in the SF-36 Mental Component Summary score. Improvement in physical function based on the HAQ-DI was maintained for up to 84 weeks through the open-label portion of the study.

14.4 Ankylosing Spondylitis

The safety and efficacy of HUMIRA 40 mg every other week was assessed in 315 adult patients in a randomized, 24 week double-blind, placebo-controlled study in patients with active ankylosing spondylitis (AS) who had an inadequate response to glucocorticoids, NSAIDs, analgesics, methotrexate or sulfasalazine. Active AS was defined as patients who fulfilled at least two of the following three criteria: (1) a Bath AS disease activity index (BASDAI) score ≥4 cm, (2) a visual analog score (VAS) for total back pain ≥ 40 mm, and (3) morning stiffness ≥ 1 hour. The blinded period was followed by an open-label period during which patients received HUMIRA 40 mg every other week subcutaneously for up to an additional 28 weeks.

Improvement in measures of disease activity was first observed at Week 2 and maintained through 24 weeks as shown in Figure 2 and Table 11.

Responses of patients with total spinal ankylosis (n=11) were similar to those without total ankylosis.

Figure 2. ASAS 20 Response By Visit, Study AS-I

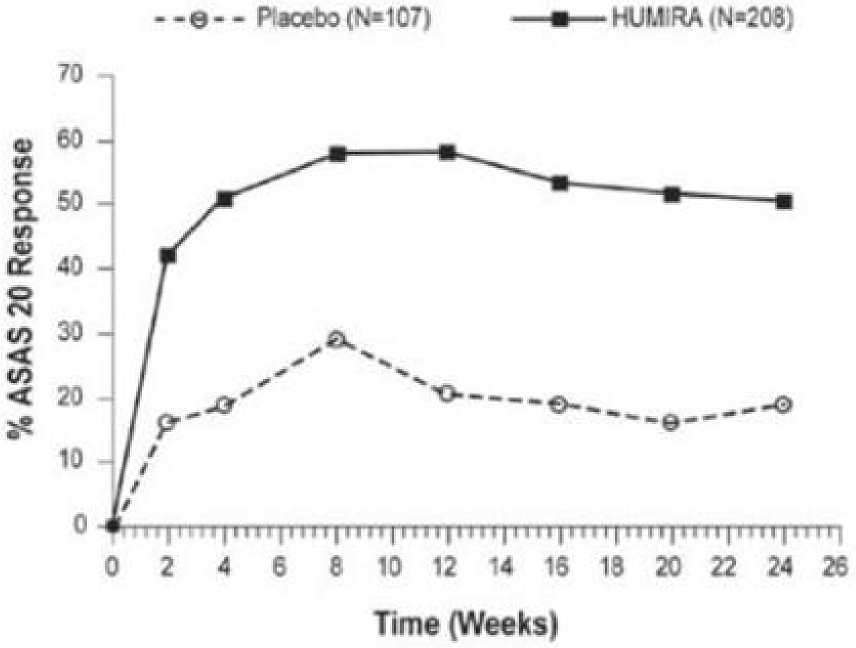

At 12 weeks, the ASAS 20/50/70 responses were achieved by 58%, 38%, and 23%, respectively, of patients receiving HUMIRA, compared to 21%, 10%, and 5% respectively, of patients receiving placebo (p <0.001). Similar responses were seen at Week 24 and were sustained in patients receiving open-label HUMIRA for up to 52 weeks.

A greater proportion of patients treated with HUMIRA (22%) achieved a low level of disease activity at 24 weeks (defined as a value <20 [on a scale of 0 to 100 mm] in each of the four ASAS response parameters) compared to patients treated with placebo (6%).

Table 11. Components of Ankylosing Spondylitis Disease Activity

	Placebo N=107		HUMIRA N=208	
	Baseline mean	Week 24 mean	Baseline mean	Week 24 mean
ASAS 20 Response Criteria*				
Patient's Global Assessment of Disease Activity[a]*	65	60	63	38
Total back pain*	67	58	65	37
Inflammation[b]*	6.7	5.6	6.7	3.6
BASFI[c]*	56	51	52	34
BASDAI[d] score*	6.3	5.5	6.3	3.7
BASMI[e] score*	4.2	4.1	3.8	3.3
Tragus to wall (cm)	15.9	15.8	15.8	15.4
Lumbar flexion (cm)	4.1	4.0	4.2	4.4
Cervical rotation (degrees)	42.2	42.1	48.4	51.6
Lumbar side flexion (cm)	8.9	9.0	9.7	11.7

Intermalleolar distance (cm)	92.9	94.0	93.5	100.8
CRP[f*]	2.2	2.0	1.8	0.6

[a] Percent of subjects with at least a 20% and 10-unit improvement measured on a Visual Analog Scale (VAS) with 0 = “none” and 100 = “severe”
[b] mean of questions 5 and 6 of BASDAI (defined in ‘d’)
[c] Bath Ankylosing Spondylitis Functional Index
[d] Bath Ankylosing Spondylitis Disease Activity Index
[e] Bath Ankylosing Spondylitis Metrology Index
[f] C-Reactive Protein (mg/dL)
* statistically significant for comparisons between HUMIRA and placebo at Week 24

A second randomized, multicenter, double-blind, placebo-controlled study of 82 patients with ankylosing spondylitis showed similar results.

Patients treated with HUMIRA achieved improvement from baseline in the Ankylosing Spondylitis Quality of Life Questionnaire (ASQoL) score (-3.6 *vs.* -1.1) and in the Short Form Health Survey (SF-36) Physical Component Summary (PCS) score (7.4 *vs.* 1.9) compared to placebo-treated patients at Week 24.

14.5 Adult Crohn’s Disease

The safety and efficacy of multiple doses of HUMIRA were assessed in adult patients with moderately to severely active Crohn’s disease, CD, (Crohn’s Disease Activity Index (CDAI) ≥ 220 and ≤ 450) in randomized, double-blind, placebo-controlled studies. Concomitant stable doses of aminosalicylates, corticosteroids, and/or immunomodulatory agents were permitted, and 79% of patients continued to receive at least one of these medications.

Induction of clinical remission (defined as CDAI < 150) was evaluated in two studies. In Study CD-I, 299 TNF-blocker naïve patients were randomized to one of four treatment groups: the placebo group received placebo at Weeks 0 and 2, the 160/80 group received 160 mg HUMIRA at Week 0 and 80 mg at Week 2, the 80/40 group received 80 mg at Week 0 and 40 mg at Week 2, and the 40/20 group received 40 mg at Week 0 20 mg at Week 2. Clinical results were assessed at Week 4.

In the second induction study, Study CD-II, 325 patients who had lost response to, or were intolerant to, previous infliximab therapy were randomized to receive either 160 mg HUMIRA at Week 0 and 80 mg at Week 2, or placebo at Weeks 0 and 2. Clinical results were assessed at Week 4.

Maintenance of clinical remission was evaluated in Study CD-III. In this study, 854 patients with active disease received open-label HUMIRA, 80 mg at week 0 and 40 mg at Week 2. Patients were then randomized at Week 4 to 40 mg HUMIRA every other week, 40 mg HUMIRA every week, or placebo. The total study duration was 56 weeks. Patients in clinical response (decrease in CDAI ≥70) at Week 4 were stratified and analyzed separately from those not in clinical response at Week 4.

<u>Induction of Clinical Remission</u>

A greater percentage of the patients treated with 160/80 mg HUMIRA achieved induction of clinical remission versus placebo at Week 4 regardless of whether the patients were TNF blocker naïve (CD-I), or had lost response to or were intolerant to infliximab (CD-II)

(see Table 12).

Table 12. Induction of Clinical Remission in Studies CD-I and CD-II (Percent of Patients)

	CD-I		CD-II	
	**Placebo N=74**	**HUMIRA 160/80 mg N=76**	**Placebo N=166**	**HUMIRA 160/80 mg N=159**
Week 4				
Clinical remission	12%	36%*	7%	21%*
Clinical response	34%	58%**	34%	52%**

Clinical remission is CDAI score < 150; clinical response is decrease in CDAI of at least 70 points.
* p<0.001 for HUMIRA *vs.* placebo pairwise comparison of proportions
** p<0.01 for HUMIRA *vs.* placebo pairwise comparison of proportions

<u>Maintenance of Clinical Remission</u>

In Study CD-III at Week 4, 58% (499/854) of patients were in clinical response and were assessed in the primary analysis. At Weeks 26 and 56, greater proportions of patients who were in clinical response at Week 4 achieved clinical remission in the HUMIRA 40 mg every other week maintenance group compared to patients in the placebo maintenance group (see Table 13). The group that received HUMIRA therapy every week did not demonstrate significantly higher remission rates compared to the group that received HUMIRA every other week.

Table 13. Maintenance of Clinical Remission in CD-III (Percent of Patients)

	**Placebo**	**40 mg HUMIRA every other week**
	**N=170**	**N=172**
**Week 26**		
Clinical remission	17%	40%*
Clinical response	28%	54%*
**Week 56**		
Clinical remission	12%	36%*
Clinical response	18%	43%*

Clinical remission is CDAI score < 150; clinical response is decrease in CDAI of at least 70 points.
*p<0.001 for HUMIRA vs. placebo pairwise comparisons of proportions

Of those in response at Week 4 who attained remission during the study, patients in the HUMIRA every other week group maintained remission for a longer time than patients in the placebo maintenance group. Among patients who were not in response by Week 12, therapy continued beyond 12 weeks did not result in significantly more responses.

14.6 Pediatric Crohn’s Disease

A randomized, double-blind, 52-week clinical study of 2 dose concentrations of HUMIRA (Study PCD-I) was conducted in 192 pediatric patients (6 to 17 years of age) with moderately to severely active Crohn's disease (defined as Pediatric Crohn's Disease Activity Index (PCDAI) score > 30). Enrolled patients had over the previous two year period an inadequate response to corticosteroids or an immunomodulator (i.e., azathioprine, 6-mercaptopurine, or methotrexate). Patients who had previously received a TNF blocker were allowed to enroll if they had previously had loss of response or intolerance to that TNF blocker.

Patients received open-label induction therapy at a dose based on their body weight (≥40 kg and <40 kg). Patients weighing ≥40 kg received 160 mg (at Week 0) and 80 mg (at Week 2). Patients weighing <40 kg received 80 mg (at Week 0) and 40 mg (at Week 2). At Week 4, patients within each body weight category (≥40 kg and <40 kg) were randomized 1:1 to one of two maintenance dose regimens (high dose and low dose). The high dose was 40 mg every other week for patients weighing ≥40 kg and 20 mg every other week for patients weighing <40 kg. The low dose was 20 mg every other week for patients weighing ≥40 kg and 10 mg every other week for patients weighing <40 kg.

Concomitant stable dosages of corticosteroids (prednisone dosage ≤40 mg/day or equivalent) and immunomodulators (azathioprine, 6-mercaptopurine, or methotrexate) were permitted throughout the study.

At Week 12, patients who experienced a disease flare (increase in PCDAI of ≥ 15 from Week 4 and absolute PCDAI > 30) or who were non-responders (did not achieve a decrease in the PCDAI of ≥ 15 from baseline for 2 consecutive visits at least 2 weeks apart) were allowed to dose-escalate (i.e., switch from blinded every other week dosing to blinded every week dosing); patients who dose-escalated were considered treatment failures.

At baseline, 38% of patients were receiving corticosteroids, and 62% of patients were receiving an immunomodulator. Forty-four percent (44%) of patients had previously lost response or were intolerant to a TNF blocker. The median baseline PCDAI score was 40.

Of the 192 patients total, 188 patients completed the 4 week induction period, 152 patients completed 26 weeks of treatment, and 124 patients completed 52 weeks of treatment. Fifty-one percent (51%) (48/95) of patients in the low maintenance dose group dose-escalated, and 38% (35/93) of patients in the high maintenance dose group dose-escalated.

At Week 4, 28% (52/188) of patients were in clinical remission (defined as PCDAI ≤ 10).

The proportions of patients in clinical remission (defined as PCDAI ≤ 10) and clinical response (defined as reduction in PCDAI of at least 15 points from baseline) were assessed at Weeks 26 and 52.

At both Weeks 26 and 52, the proportion of patients in clinical remission and clinical response was numerically higher in the high dose group compared to the low dose group (Table 14). The recommended maintenance regimen is 20 mg every other week for patients weighing < 40 kg and 40 mg every other week for patients weighing ≥ 40 kg. Every week dosing is not the recommended maintenance dosing regimen *[see Dosage and Administration (2.3)]*.

Table 14. Clinical Remission and Clinical Response in Study PCD-I

	**Low Maintenance Dose† (20 or 10 mg every other week) N = 95**	**High Maintenance Dose# (40 or 20 mg every other week) N = 93**
**Week 26**		
Clinical Remission‡	28%	39%
Clinical Response§	48%	59%
**Week 52**		
Clinical Remission‡	23%	33%
Clinical Response§	28%	42%

†The low maintenance dose was 20 mg every other week for patients weighing ≥ 40 kg and 10 mg every other week for patients weighing < 40 kg.
#The high maintenance dose was 40 mg every other week for patients weighing ≥ 40 kg and 20 mg every other week for patients weighing < 40 kg.
‡Clinical remission defined as PCDAI ≤ 10.
§Clinical response defined as reduction in PCDAI of at least 15 points from baseline.

14.7 Adult Ulcerative Colitis

The safety and efficacy of HUMIRA were assessed in adult patients with moderately to severely active ulcerative colitis (Mayo score 6 to 12 on a 12 point scale, with an endoscopy subscore of 2 to 3 on a scale of 0 to 3) despite concurrent or prior treatment with immunosuppressants such as corticosteroids, azathioprine, or 6-MP in two randomized, double-blind, placebo-controlled clinical studies (Studies UC-I and UC-II). Both studies enrolled TNF-blocker naïve patients, but Study UC-II also allowed entry of patients who lost response to or were intolerant to TNF-blockers. Forty percent (40%) of patients enrolled in Study UC-II had previously used another TNF-blocker.

Concomitant stable doses of aminosalicylates and immunosuppressants were permitted. In Studies UC-I and II, patients were receiving aminosalicylates (69%), corticosteroids (59%) and/or azathioprine or 6-MP (37%) at baseline. In both studies, 92% of patients received at least one of these medications.

Induction of clinical remission (defined as Mayo score ≤ 2 with no individual subscores > 1) at Week 8 was evaluated in both studies. Clinical remission at Week 52 and sustained clinical remission (defined as clinical remission at both Weeks 8 and 52) were evaluated in Study UC-II.

In Study UC-I, 390 TNF-blocker naïve patients were randomized to one of three treatment groups for the primary efficacy analysis. The placebo group received placebo at Weeks 0, 2, 4 and 6. The 160/80 group received 160 mg HUMIRA at Week 0 and 80 mg at Week 2, and the 80/40 group received 80 mg HUMIRA at Week 0 and 40 mg at Week 2. After Week 2, patients in both HUMIRA treatment groups received 40 mg every other week.

In Study UC-II, 518 patients were randomized to receive either HUMIRA 160 mg at Week 0, 80 mg at Week 2, and 40 mg every other week starting at Week 4 through Week 50, or placebo starting at Week 0 and every other week through Week 50. Corticosteroid taper was permitted starting at Week 8.

In both Studies UC-I and UC-II, a greater percentage of the patients treated with 160/80 mg of HUMIRA compared to patients treated with placebo achieved induction of clinical remission. In Study UC-II, a greater percentage of the patients treated with 160/80 mg of HUMIRA compared to patients treated with placebo achieved sustained clinical remission (clinical remission at both Weeks 8 and 52) (Table 15).

Table 15. Induction of Clinical Remission in Studies UC-I and UC-II and Sustained Clinical Remission in Study UC-II (Percent of Patients)

	**Study UC-I**			**Study UC-II**		
	**Placebo N=130**	**HUMIRA 160/80 mg N=130**	**Treatment Difference (95% CI)**	**Placebo N=246**	**HUMIRA 160/80 mg N=248**	**Treatment Difference (95% CI)**
Induction of Clinical Remission (Clinical Remission at Week 8)	9.2%	18.5%	9.3%* (0.9%, 17.6%)	9.3%	16.5%	7.2%* (1.2%, 12.9%)
Sustained Clinical Remission (Clinical Remission at both Weeks 8 and 52)	N/A	N/A	N/A	4.1%	8.5%	4.4%* (0.1%, 8.6%)
Clinical remission is defined as Mayo score ≤ 2 with no individual subscores > 1.<br>CI=Confidence interval<br>* p<0.05 for HUMIRA *vs.* placebo pairwise comparison of proportions						

In Study UC-I, there was no statistically significant difference in clinical remission observed between the HUMIRA 80/40 mg group and the placebo group at Week 8.

In Study UC-II, 17.3% (43/248) in the HUMIRA group were in clinical remission at Week 52 compared to 8.5% (21/246) in the placebo group (treatment difference: 8.8%; 95% confidence interval (CI): [2.8%, 14.5%]; p<0.05).

In the subgroup of patients in Study UC-II with prior TNF-blocker use, the treatment difference for induction of clinical remission appeared to be lower than that seen in the whole study population, and the treatment differences for sustained clinical remission and clinical remission at Week 52 appeared to be similar to those seen in the whole study population. The subgroup of patients with prior TNF-blocker use achieved induction of clinical remission at 9% (9/98) in the HUMIRA group versus 7% (7/101) in the placebo group, and sustained clinical remission at 5% (5/98) in the HUMIRA group versus 1% (1/101) in the placebo group. In the subgroup of patients with prior TNF-blocker use, 10% (10/98) were in clinical remission at Week 52 in the HUMIRA group versus 3% (3/101) in the placebo group.

14.8 Pediatric Ulcerative Colitis

The safety and efficacy of HUMIRA were assessed in a multicenter, randomized, double-blind trial (Study PUC-I, NCT02065557) in 93 pediatric patients 5 years to 17 years of age with moderately to severely active ulcerative colitis (Mayo score 6 to 12 with endoscopy subscore of 2 to 3 points, confirmed by centrally read endoscopy) who had an inadequate response or intolerance to therapy with corticosteroids and/or an

immunomodulator (i.e., azathioprine, 6-mercaptopurine, or methotrexate). Fifteen out of 93 patients (16%) in the study had prior experience with a TNF blocker. Patients who received corticosteroids at enrollment were allowed to taper their corticosteroid therapy after Week 4.

Seventy-seven patients were initially randomized 3:2 to receive double-blind treatment with one of two dosages of HUMIRA. Patients in both dosage groups received 2.4 mg/kg (maximum of 160 mg) at Week 0, 1.2 mg/kg (maximum of 80 mg) at Week 2, and 0.6 mg/kg (maximum of 40 mg) at Weeks 4 and 6. The higher dosage group also received an additional dosage of 2.4 mg/kg (maximum of 160 mg) at Week 1. Following an amendment to the study design, 16 additional patients were enrolled and received open-label treatment with HUMIRA at the higher dosage.

At Week 8, 62 patients who demonstrated clinical response per Partial Mayo Score (PMS; a subset of the Mayo score with no endoscopic component and defined as a decrease in PMS ≥ 2 points and ≥ 30% from baseline) were randomized equally to receive double-blind treatment with HUMIRA 0.6 mg/kg (maximum of 40 mg) every other week (lower dosage group), or 0.6 mg/kg (maximum of 40 mg) every week (higher dosage group). Prior to an amendment to the study design, 12 additional patients who demonstrated clinical response per PMS were randomized to receive placebo.

There are no anticipated clinically relevant differences in efficacy between the studied higher dosage administered during the 52-week PUC-I trial and the recommended dosage of HUMIRA *[see Dosage and Administration (2.4), Clinical Pharmacology (12.2)].*

Patients who met criteria for disease flare at or after Week 12 were randomized to receive a re-induction dose of 2.4 mg/kg (maximum of 160 mg) or a dose of 0.6 mg/kg (maximum of 40 mg) and then continued the dose to which they were randomized at Week 8.

The co-primary endpoints of the study were clinical remission per PMS (defined as PMS ≤ 2 and no individual subscore > 1) at Week 8, and clinical remission per the Mayo Score (defined as Mayo Score ≤ 2 and no individual subscore > 1) at Week 52 in patients who achieved clinical response per PMS at Week 8. Secondary endpoints included Mayo Score response (defined as a decrease in Mayo Score of ≥ 3 points and ≥ 30% from baseline) at Week 52 in Week 8 PMS responders, endoscopic improvement (defined as a Mayo endoscopy subscore ≤ 1) at Week 52 in Week 8 PMS responders, and Mayo Score remission at Week 52 in Week 8 PMS remitters.

Week 8 Results

At Week 8, PMS remission was achieved by 60% [28/47; 95% confidence interval (CI): (44%, 74%)] of patients in the higher dosage group (not including the 16 patients receiving open-label higher dosage) and 43% [13/30; 95% CI: (25%, 63%)] of patients in the lower dosage group. Results from the higher dosage group are representative of the results expected with the recommended dosage *[see Dosage and Administration (2.4), Clinical Pharmacology (12.2)].*

Week 52 Results

At Week 52, endpoints were assessed in the population of patients who received double-blind placebo, HUMIRA 0.6 mg/kg (maximum of 40 mg) every other week, or HUMIRA 0.6 mg/kg (maximum of 40 mg) every week between Week 8 and Week 52 (Table 16).

Table 16. Clinical Remission, Clinical Response and Endoscopic Improvement at Week 52 in Pediatric Patients with Ulcerative Colitis (Study PUC-1)

	Placebo[a]<br><br>n/N (%), 95% CI	HUMIRA Maximum of 40 mg (0.6 mg/kg) every other week[b]<br><br>n/N (%), 95% CI	HUMIRA Maximum of 40 mg (0.6 mg/kg) every week[c]<br><br>n/N (%), 95% CI
Clinical remission in Week 8 PMS responders	4/12 (33%)<br>(10%, 65%)	9/31 (29%)<br>(14%, 48%)	14/31 (45%)<br>(27%, 64%)
Clinical response in Week 8 PMS responders	4/12 (33%)<br>(10%, 65%)	19/31 (61%)<br>(42%, 78%)	21/31 (68%)<br>(49%, 83%)
Endoscopic improvement in Week 8 PMS responders	4/12 (33%)<br>(10%, 65%)	12/31 (39%)<br>(22%, 58%)	16/31 (52%)<br>(33%, 70%)
Clinical remission in Week 8 PMS remitters	3/8 (38%)<br>(9%, 76%)	9/21 (43%)<br>(22%, 66%)	10/22 (45%)<br>(24%, 68%)

CI=Confidence interval

[a] Twelve patients who demonstrated clinical response per PMS at Week 8 were randomized to receive placebo. There are limitations to the interpretability of the placebo data due to the small sample size.

[b] The every other week dosage studied during the 52-week PUC-I trial is a lower dosage than the recommended dosage of HUMIRA *[see Dosage and Administration (2.4)]*.

[c] There are no anticipated clinically relevant differences in efficacy between the studied higher dosage administered during the 52-week PUC-I trial and the recommended dosage of HUMIRA.

Note: Patients with missing values at Week 52 or who were randomized to receive re-induction or maintenance treatment due to disease flare were considered non-responders for Week 52 endpoints.

14.9 Plaque Psoriasis

The safety and efficacy of HUMIRA were assessed in randomized, double-blind, placebo-controlled studies in 1696 adult subjects with moderate to severe chronic plaque psoriasis (Ps) who were candidates for systemic therapy or phototherapy.

Study Ps-I evaluated 1212 subjects with chronic Ps with ≥10% body surface area (BSA) involvement, Physician's Global Assessment (PGA) of at least moderate disease severity, and Psoriasis Area and Severity Index (PASI) ≥12 within three treatment periods. In period A, subjects received placebo or HUMIRA at an initial dose of 80 mg at Week 0 followed by a dose of 40 mg every other week starting at Week 1. After 16 weeks of therapy, subjects who achieved at least a PASI 75 response at Week 16, defined as a PASI score improvement of at least 75% relative to baseline, entered period B and received open-label 40 mg HUMIRA every other week. After 17 weeks of open label

therapy, subjects who maintained at least a PASI 75 response at Week 33 and were originally randomized to active therapy in period A were re-randomized in period C to receive 40 mg HUMIRA every other week or placebo for an additional 19 weeks. Across all treatment groups the mean baseline PASI score was 19 and the baseline Physician's Global Assessment score ranged from "moderate" (53%) to "severe" (41%) to "very severe" (6%).

Study Ps-II evaluated 99 subjects randomized to HUMIRA and 48 subjects randomized to placebo with chronic plaque psoriasis with ≥10% BSA involvement and PASI ≥12. Subjects received placebo, or an initial dose of 80 mg HUMIRA at Week 0 followed by 40 mg every other week starting at Week 1 for 16 weeks. Across all treatment groups the mean baseline PASI score was 21 and the baseline PGA score ranged from "moderate" (41%) to "severe" (51%) to "very severe" (8%).

Studies Ps-I and II evaluated the proportion of subjects who achieved "clear" or "minimal" disease on the 6-point PGA scale and the proportion of subjects who achieved a reduction in PASI score of at least 75% (PASI 75) from baseline at Week 16 (see Table 17 and 18).

Additionally, Study Ps-I evaluated the proportion of subjects who maintained a PGA of "clear" or "minimal" disease or a PASI 75 response after Week 33 and on or before Week 52.

Table 17. Efficacy Results at 16 Weeks in Study Ps-I Number of Subjects (%)

	HUMIRA 40 mg every other week	Placebo
	N = 814	N = 398
PGA: *Clear* or *minimal**	506 (62%)	17 (4%)
PASI 75	578 (71%)	26 (7%)

* Clear = no plaque elevation, no scale, plus or minus hyperpigmentation or diffuse pink or red coloration
Minimal = possible but difficult to ascertain whether there is slight elevation of plaque above normal skin, plus or minus surface dryness with some white coloration, plus or minus up to red coloration

Table 18. Efficacy Results at 16 Weeks in Study Ps-II Number of Subjects (%)

	HUMIRA 40 mg every other week	Placebo
	N = 99	N = 48
PGA: *Clear* or *minimal**	70 (71%)	5 (10%)
PASI 75	77 (78%)	9 (19%)

* Clear = no plaque elevation, no scale, plus or minus hyperpigmentation or diffuse pink or red coloration
Minimal = possible but difficult to ascertain whether there is slight elevation of plaque above normal skin, plus or minus surface dryness with some white coloration, plus or minus up to red coloration

Additionally, in Study Ps-I, subjects on HUMIRA who maintained a PASI 75 were re-

randomized to HUMIRA (N = 250) or placebo (N = 240) at Week 33. After 52 weeks of treatment with HUMIRA, more subjects on HUMIRA maintained efficacy when compared to subjects who were re-randomized to placebo based on maintenance of PGA of “clear” or “minimal” disease (68% *vs.* 28%) or a PASI 75 (79% *vs.* 43%).

A total of 347 stable responders participated in a withdrawal and retreatment evaluation in an open-label extension study. Median time to relapse (decline to PGA “moderate” or worse) was approximately 5 months. During the withdrawal period, no subject experienced transformation to either pustular or erythrodermic psoriasis. A total of 178 subjects who relapsed re-initiated treatment with 80 mg of HUMIRA, then 40 mg every other week beginning at week 1. At week 16, 69% (123/178) of subjects had a response of PGA “clear” or “minimal”.

A randomized, double-blind study (Study Ps-III) compared the efficacy and safety of HUMIRA versus placebo in 217 adult subjects. Subjects in the study had to have chronic plaque psoriasis of at least moderate severity on the PGA scale, fingernail involvement of at least moderate severity on a 5-point Physician’s Global Assessment of Fingernail Psoriasis (PGA-F) scale, a Modified Nail Psoriasis Severity Index (mNAPSI) score for the target-fingernail of ≥ 8, and either a BSA involvement of at least 10% or a BSA involvement of at least 5% with a total mNAPSI score for all fingernails of ≥ 20. Subjects received an initial dose of 80 mg HUMIRA followed by 40 mg every other week (starting one week after the initial dose) or placebo for 26 weeks followed by open-label HUMIRA treatment for an additional 26 weeks. This study evaluated the proportion of subjects who achieved “clear” or “minimal” assessment with at least a 2-grade improvement on the PGA-F scale and the proportion of subjects who achieved at least a 75% improvement from baseline in the mNAPSI score (mNAPSI 75) at Week 26.

At Week 26, a higher proportion of subjects in the HUMIRA group than in the placebo group achieved the PGA-F endpoint. Furthermore, a higher proportion of subjects in the HUMIRA group than in the placebo group achieved mNAPSI 75 at Week 26 (see Table 19).

Table 19. Efficacy Results at 26 Weeks

Endpoint	HUMIRA 40 mg every other week* N=109	Placebo N=108
PGA-F: ≥2-grade improvement and *clear* or *minimal*	49%	7%
mNAPSI 75	47%	3%

*Subjects received 80 mg of HUMIRA at Week 0, followed by 40 mg every other week starting at Week 1.

Nail pain was also evaluated and improvement in nail pain was observed in Study Ps-III.

14.10 Hidradenitis Suppurativa

Two randomized, double-blind, placebo-controlled studies (Studies HS-I and II) evaluated the safety and efficacy of HUMIRA in a total of 633 adult subjects with moderate to severe hidradenitis suppurativa (HS) with Hurley Stage II or III disease and with at least 3 abscesses or inflammatory nodules. In both studies, subjects received placebo or

HUMIRA at an initial dose of 160 mg at Week 0, 80 mg at Week 2, and 40 mg every week starting at Week 4 and continued through Week 11. Subjects used topical antiseptic wash daily. Concomitant oral antibiotic use was allowed in Study HS-II.

Both studies evaluated Hidradenitis Suppurativa Clinical Response (HiSCR) at Week 12. HiSCR was defined as at least a 50% reduction in total abscess and inflammatory nodule count with no increase in abscess count and no increase in draining fistula count relative to baseline (see Table 18). Reduction in HS-related skin pain was assessed using a Numeric Rating Scale in patients who entered the study with an initial baseline score of 3 or greater on a 11 point scale.

In both studies, a higher proportion of HUMIRA- than placebo-treated subjects achieved HiSCR (see Table 20).

Table 20. Efficacy Results at 12 Weeks in Subjects with Moderate to Severe Hidradenitis Suppurativa

	HS Study I		HS Study II*	
	**Placebo**	**Humira 40 mg Weekly**	**Placebo**	**Humira 40 mg Weekly**
Hidradenitis Suppurativa Clinical Response (HiSCR)	N = 154<br>40 (26%)	N = 153<br>64 (42%)	N=163<br>45 (28%)	N=163<br>96 (59%)
*19.3% of subjects in Study HS-II continued baseline oral antibiotic therapy during the study.				

In both studies, from Week 12 to Week 35 (Period B), subjects who had received HUMIRA were re-randomized to 1 of 3 treatment groups (HUMIRA 40 mg every week, HUMIRA 40 mg every other week, or placebo). Subjects who had been randomized to placebo were assigned to receive HUMIRA 40 mg every week (Study HS-I) or placebo (Study HS-II).

During Period B, flare of HS, defined as ≥25% increase from baseline in abscesses and inflammatory nodule counts and with a minimum of 2 additional lesions, was documented in 22 (22%) of the 100 subjects who were withdrawn from HUMIRA treatment following the primary efficacy timepoint in two studies.

14.11 Adult Uveitis

The safety and efficacy of HUMIRA were assessed in adult patients with non-infectious intermediate, posterior and panuveitis excluding patients with isolated anterior uveitis, in two randomized, double-masked, placebo-controlled studies (UV I and II). Patients received placebo or HUMIRA at an initial dose of 80 mg followed by 40 mg every other week starting one week after the initial dose. The primary efficacy endpoint in both studies was ´time to treatment failure´.

Treatment failure was a multi-component outcome defined as the development of new inflammatory chorioretinal and/or inflammatory retinal vascular lesions, an increase in anterior chamber (AC) cell grade or vitreous haze (VH) grade or a decrease in best corrected visual acuity (BCVA).

Study UV I evaluated 217 patients with active uveitis while being treated with

corticosteroids (oral prednisone at a dose of 10 to 60 mg/day). All patients received a standardized dose of prednisone 60 mg/day at study entry followed by a mandatory taper schedule, with complete corticosteroid discontinuation by Week 15.

Study UV II evaluated 226 patients with inactive uveitis while being treated with corticosteroids (oral prednisone 10 to 35 mg/day) at baseline to control their disease. Patients subsequently underwent a mandatory taper schedule, with complete corticosteroid discontinuation by Week 19.

Clinical Response

Results from both studies demonstrated statistically significant reduction of the risk of treatment failure in patients treated with HUMIRA versus patients receiving placebo. In both studies, all components of the primary endpoint contributed cumulatively to the overall difference between HUMIRA and placebo groups (Table 21).

Table 21. Time to Treatment Failure in Studies UV I and UV II

	UV I			UV II		
	Placebo (N = 107)	HUMIRA (N = 110)	HR [95% CI][a]	Placebo (N = 111)	HUMIRA (N = 115)	HR [95% CI][a]
Failure[b] n (%)	84 (78.5)	60 (54.5)	0.50 [0.36, 0.70]	61 (55.0)	45 (39.1)	0.57 [0.39, 0.84]
Median Time to Failure (Months) [95% CI]	3.0 [2.7, 3.7]	5.6 [3.9, 9.2]	N/A	8.3 [4.8, 12.0]	NE[c]	N/A

[a] HR of HUMIRA versus placebo from proportional hazards regression with treatment as factor.
[b] Treatment failure at or after Week 6 in Study UV I, or at or after Week 2 in Study UV II, was counted as event. Subjects who discontinued the study were censored at the time of dropping out.
[c] NE = not estimable. Fewer than half of at-risk subjects had an event.

Figure 3: Kaplan-Meier Curves Summarizing Time to Treatment Failure on or after Week 6 (Study UV I) or Week 2 (Study UV II)

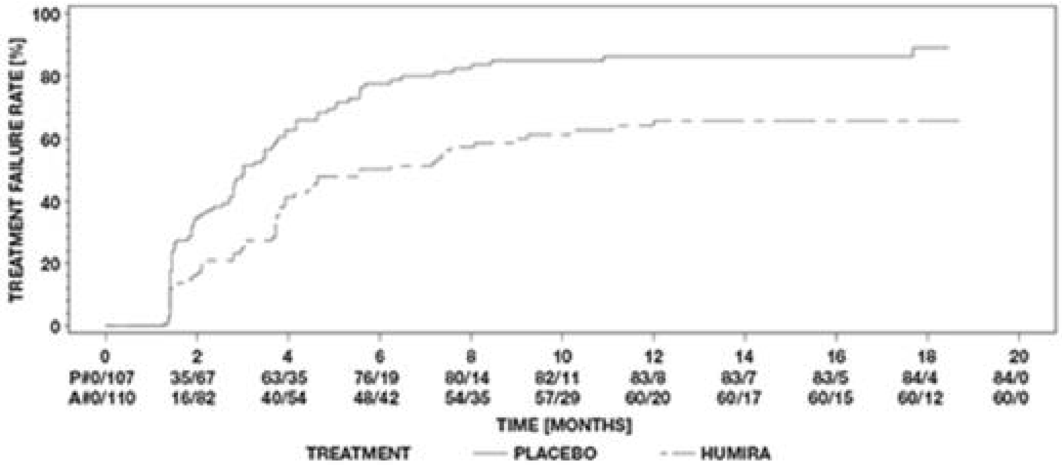

Study UV I

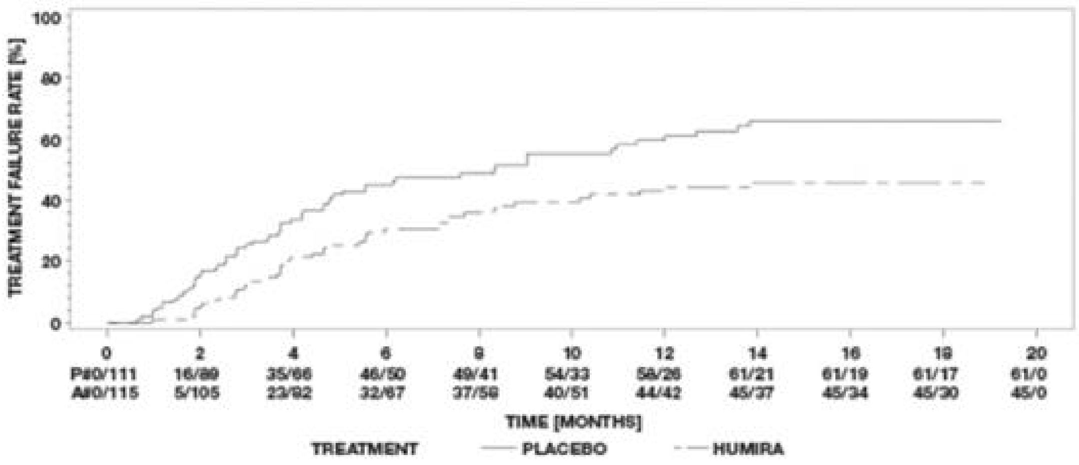

Study UV II

Note: P# = Placebo (Number of Events/Number at Risk); A# = HUMIRA (Number of Events/Number at Risk).

14.12 Pediatric Uveitis

The safety and efficacy of HUMIRA were assessed in a randomized, double-masked, placebo-controlled study of 90 pediatric patients from 2 to < 18 years of age with active JIA-associated non-infectious uveitis (PUV-I). Patients received either placebo or 20 mg adalimumab (if < 30 kg) or 40 mg adalimumab (if ≥ 30 kg) every other week in combination with a dose of methotrexate. Concomitant dosages of corticosteroids were permitted at study entry followed by a mandatory reduction in topical corticosteroids within 3 months.

The primary endpoint was ‘time to treatment failure’. The criteria determining treatment failure were worsening or sustained non-improvement in ocular inflammation, or worsening of ocular co-morbidities.

Clinical Response

HUMIRA significantly decreased the risk of treatment failure by 75% relative to placebo (HR = 0.25 [95% CI: 0.12, 0.49]) (Table 22).

Table 22. Analysis Results of Time to Treatment Failure (Study PUV-I)

	Placebo (N=30)	HUMIRA (N=60)	HR (95% CI)[a]
**Failure (n[%])**	18 (60%)	16 (26.7%)	0.25 (0.12, 0.49)
**Median Time to Failure (Weeks) (95% CI)[b]**	24.1 (12.4, 81.0)	NE[c]	

[a] HR of adalimumab versus placebo from proportional hazards regression with treatment as factor.
[b] Estimated based on Kaplan-Meier curve.
[c] NE = not estimable. Fewer than half of at-risk subjects had an event.

Figure 4: Kaplan-Meier Curves Summarizing Time to Treatment Failure (Study PUV-I)

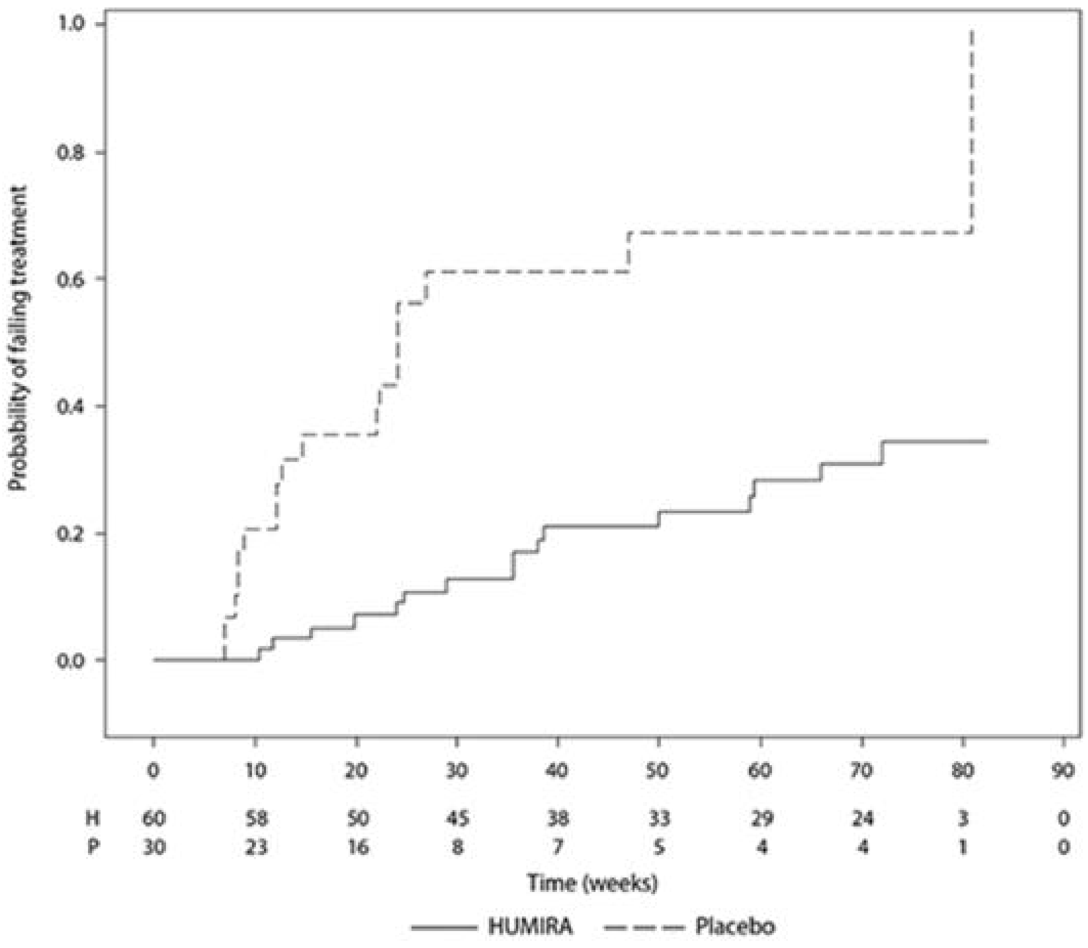

Study PUV-I

Note: P = Placebo (Number at Risk); H = HUMIRA (Number at Risk).

15 REFERENCES

1. National Cancer Institute. Surveillance, Epidemiology, and End Results Database (SEER) Program. SEER Incidence Crude Rates, 17 Registries, 2000-2007.

16 HOW SUPPLIED/STORAGE AND HANDLING

HUMIRA® (adalimumab) is supplied as a preservative-free, sterile, clear and colorless solution for subcutaneous administration. The following packaging configurations are available.

- **HUMIRA Pen Carton - 40 mg/0.4 mL**
  HUMIRA is supplied in a carton containing two alcohol preps and two dose trays. Each dose tray consists of a single-dose pen, containing a 1 mL prefilled glass syringe with a fixed thin wall, ½ inch needle, providing 40 mg/0.4 mL of HUMIRA. The black needle cover is not made with natural rubber latex. The NDC number is 83457-554-02
- **HUMIRA Pen Carton – 80 mg/0.8 mL**
  HUMIRA is supplied in a carton containing two alcohol preps and two dose trays. Each dose tray consists of a single-dose pen, containing a 1 mL prefilled glass

syringe with a fixed thin wall, ½ inch needle, providing 80 mg/0.8 mL of HUMIRA. The black needle cover is not made with natural rubber latex. The NDC number is 83457-124-02.

- **Prefilled Syringe Carton - 40 mg/0.4 mL**
  HUMIRA is supplied in a carton containing two alcohol preps and two dose trays. Each dose tray consists of a single-dose, 1 mL prefilled glass syringe with a fixed thin wall, ½ inch needle, providing 40 mg/0.4 mL of HUMIRA. The black needle cover is not made with natural rubber latex. The NDC number is 83457-243-02.
- **Prefilled Syringe Carton - 20 mg/0.2 mL**
  HUMIRA is supplied in a carton containing two alcohol preps and two dose trays. Each dose tray consists of a single-dose, 1 mL prefilled glass syringe with a fixed thin wall, ½ inch needle, providing 20 mg/0.2 mL of HUMIRA. The black needle cover is not made with natural rubber latex. The NDC number is 83457-616-02.
- **Prefilled Syringe Carton - 10 mg/0.1 mL**
  HUMIRA is supplied in a carton containing two alcohol preps and two dose trays. Each dose tray consists of a single-dose, 1 mL prefilled glass syringe with a fixed thin wall, ½ inch needle, providing 10 mg/0.1 mL of HUMIRA. The black needle cover is not made with natural rubber latex. The NDC number is 83457-817-02.

<u>Storage and Stability</u>

Do not use beyond the expiration date on the container. HUMIRA must be refrigerated at 36°F to 46°F (2°C to 8°C). DO NOT FREEZE. Do not use if frozen even if it has been thawed.

Store in original carton until time of administration to protect from light.

If needed, for example when traveling, HUMIRA may be stored at room temperature up to a maximum of 77°F (25°C) for a period of up to 14 days, with protection from light. HUMIRA should be discarded if not used within the 14-day period. Record the date when HUMIRA is first removed from the refrigerator in the spaces provided on the carton and dose tray.

Do not store HUMIRA in extreme heat or cold.

17 PATIENT COUNSELING INFORMATION

Advise the patient or caregiver to read the FDA-approved patient labeling (Medication Guide and Instructions for Use).

<u>Infections</u>
Inform patients that HUMIRA may lower the ability of their immune system to fight infections. Instruct patients of the importance of contacting their doctor if they develop any symptoms of infection, including tuberculosis, invasive fungal infections, and reactivation of hepatitis B virus infections *[see Warnings and Precautions (5.1, 5.2, 5.4)]*.

<u>Malignancies</u>
Counsel patients about the risk of malignancies while receiving HUMIRA *[see Warnings and Precautions (5.2)]*

<u>Hypersensitivity Reactions</u>
Advise patients to seek immediate medical attention if they experience any symptoms of severe hypersensitivity reactions. Advise latex-sensitive patients that the needle cap of

the HUMIRA 40 mg/0.8 mL Pen and 40 mg/0.8 mL, 20 mg/0.4 mL and 10 mg/0.2 mL prefilled syringe may contain natural rubber latex *[see Warnings and Precautions (5.3), How Supplied/Storage and Handling (16)]*.

Other Medical Conditions

Advise patients to report any signs of new or worsening medical conditions such as congestive heart failure, neurological disease, autoimmune disorders, or cytopenias. Advise patients to report any symptoms suggestive of a cytopenia such as bruising, bleeding, or persistent fever *[see Warnings and Precautions (5.5, 5.6, 5.8, 5.9)]*.

Instructions on Injection Technique

Inform patients that the first injection is to be performed under the supervision of a qualified health care professional. If a patient or caregiver is to administer HUMIRA, instruct them in injection techniques and assess their ability to inject subcutaneously to ensure the proper administration of HUMIRA *[see Instructions for Use].*

For patients who will use the HUMIRA Pen, tell them that they:

- Will hear a **loud 'click'** when the plum-colored activator button is pressed. The loud click means the **start** of the injection.
- Must keep holding the HUMIRA Pen against their squeezed, raised skin until all of the medicine is injected. This can take up to 15 seconds.
- Will know that the injection has finished when the yellow marker fully appears in the window view and stops moving.

Instruct patients to dispose of their used needles and syringes or used Pen in a FDA-cleared sharps disposal container immediately after use. **Instruct patients not to dispose of loose needles and syringes or Pen in their household trash.** Instruct patients that if they do not have a FDA-cleared sharps disposal container, they may use a household container that is made of a heavy-duty plastic, can be closed with a tight-fitting and puncture-resistant lid without sharps being able to come out, upright and stable during use, leak-resistant, and properly labeled to warn of hazardous waste inside the container.

Instruct patients that when their sharps disposal container is almost full, they will need to follow their community guidelines for the correct way to dispose of their sharps disposal container. Instruct patients that there may be state or local laws regarding disposal of used needles and syringes. Refer patients to the FDA's website at http://www.fda.gov/safesharpsdisposal for more information about safe sharps disposal, and for specific information about sharps disposal in the state that they live in.

Instruct patients not to dispose of their used sharps disposal container in their household trash unless their community guidelines permit this. Instruct patients not to recycle their used sharps disposal container.

AbbVie Inc.
North Chicago, IL 60064, U.S.A.

US License Number 1889

20082819 11/2023

Manufactured for:
Cordavis Trading Limited
Dublin, Ireland

MEDICATION GUIDE
HUMIRA® (Hu-MARE-ah)
(adalimumab)
injection, for subcutaneous use

Read the Medication Guide that comes with HUMIRA before you start taking it and each time you get a refill. There may be new information. This Medication Guide does not take the place of talking with your doctor about your medical condition or treatment.

What is the most important information I should know about HUMIRA?

HUMIRA is a medicine that affects your immune system. HUMIRA can lower the ability of your immune system to fight infections. **Serious infections have happened in people taking HUMIRA. These serious infections include tuberculosis (TB) and infections caused by viruses, fungi or bacteria that have spread throughout the body. Some people have died from these infections.**

- Your doctor should test you for TB before starting HUMIRA.
- Your doctor should check you closely for signs and symptoms of TB during treatment with HUMIRA.

You should not start taking HUMIRA if you have any kind of infection unless your doctor says it is okay.

Before starting HUMIRA, tell your doctor if you:

- think you have an infection or have symptoms of an infection such as:
    - fever, sweats, or chills
    - muscle aches
    - cough
    - shortness of breath
    - blood in phlegm
    - warm, red, or painful skin or sores on your body
    - diarrhea or stomach pain
    - burning when you urinate or urinate more often than normal
    - feel very tired
    - weight loss
- are being treated for an infection.
- get a lot of infections or have infections that keep coming back.
- have diabetes.
- have TB, or have been in close contact with someone with TB.
- were born in, lived in, or traveled to countries where there is more risk for getting TB. Ask your doctor if you are not sure.
- live or have lived in certain parts of the country (such as the Ohio and Mississippi River valleys) where there is an increased risk for getting certain kinds of fungal infections (histoplasmosis, coccidioidomycosis, or blastomycosis). These infections may happen or become more severe if you use HUMIRA. Ask your doctor if you do not know if you have lived in an area where these infections are common.
- have or have had hepatitis B.
- use the medicine ORENCIA (abatacept), KINERET (anakinra), RITUXAN

(rituximab), IMURAN (azathioprine), or PURINETHOL (6–mercaptopurine, 6-MP).
- are scheduled to have major surgery.

After starting HUMIRA, call your doctor right away if you have an infection, or any sign of an infection.
HUMIRA can make you more likely to get infections or make any infection that you may have worse.

Cancer

- For children and adults taking Tumor Necrosis Factor (TNF)-blockers, including HUMIRA, the chances of getting cancer may increase.
- There have been cases of unusual cancers in children, teenagers, and young adults using TNF-blockers.
- People with rheumatoid arthritis (RA), especially more serious RA, may have a higher chance for getting a kind of cancer called lymphoma.
- If you use TNF blockers including HUMIRA your chance of getting two types of skin cancer may increase (basal cell cancer and squamous cell cancer of the skin). These types of cancer are generally not life-threatening if treated. Tell your doctor if you have a bump or open sore that does not heal.
- Some people receiving TNF blockers including HUMIRA developed a rare type of cancer called hepatosplenic T-cell lymphoma. This type of cancer often results in death. Most of these people were male teenagers or young men. Also, most people were being treated for Crohn's disease or ulcerative colitis with another medicine called IMURAN (azathioprine) or PURINETHOL (6-mercaptopurine, 6–MP).

What is HUMIRA?

HUMIRA is a medicine called a Tumor Necrosis Factor (TNF) blocker. HUMIRA is used:

- To reduce the signs and symptoms of:
  - **moderate to severe RA in adults.** HUMIRA can be used alone, with methotrexate, or with certain other medicines.
  - **moderate to severe polyarticular juvenile idiopathic arthritis (JIA) in children** 2 years and older. HUMIRA can be used alone or with methotrexate.
  - **psoriatic arthritis (PsA) in adults.** HUMIRA can be used alone or with certain other medicines.
  - **ankylosing spondylitis (AS) in adults.**
  - **moderate to severe hidradenitis suppurativa (HS) in people 12 years and older.**

- **To treat moderate to severe Crohn's disease (CD) in adults and children 6 years of age and older**.
  - **To treat moderate to severe ulcerative colitis (UC) in adults and children 5 years of age and older**. It is not known if HUMIRA is effective in people who stopped responding to or could not tolerate TNF-blocker medicines.
  - **To treat moderate to severe chronic (lasting a long time) plaque psoriasis (Ps) in adults** who have the condition in many areas of their body and who may benefit from taking injections or pills

(systemic therapy) or phototherapy (treatment using ultraviolet light alone or with pills).
- **To treat non-infectious intermediate, posterior, and panuveitis in adults and children 2 years of age and older.**

What should I tell my doctor before taking HUMIRA?
HUMIRA may not be right for you. Before starting HUMIRA, tell your doctor about all of your medical conditions, including if you:

- have an infection. See **"What is the most important information I should know about HUMIRA?"**
- have or have had cancer.
- have any numbness or tingling or have a disease that affects your nervous system such as multiple sclerosis or Guillain-Barré syndrome.
- have or had heart failure.
- have recently received or are scheduled to receive a vaccine. You may receive vaccines, except for live vaccines while using HUMIRA. Children should be brought up to date with all vaccines before starting HUMIRA.
- are allergic to rubber or latex. Tell your doctor if you have any allergies to rubber or latex.
    - The needle cover for the HUMIRA Pen 40 mg/0.8 mL, HUMIRA 40 mg/0.8 mL prefilled syringe, HUMIRA 20 mg/0.4 mL prefilled syringe, and HUMIRA 10 mg/0.2 mL prefilled syringe may contain natural rubber or latex.
    - The black needle cover for the HUMIRA Pen 80 mg/0.8 mL, HUMIRA 80 mg/0.8 mL prefilled syringe, HUMIRA Pen 40 mg/0.4 mL, HUMIRA 40 mg/0.4 mL prefilled syringe, HUMIRA 20 mg/0.2 mL prefilled syringe, HUMIRA 10 mg/0.1 mL prefilled syringe and the vial stopper on the HUMIRA institutional use vial are not made with natural rubber or latex.

- are allergic to HUMIRA or to any of its ingredients. See the end of this Medication Guide for a list of ingredients in HUMIRA.
- are pregnant or plan to become pregnant, breastfeeding or plan to breastfeed. You and your doctor should decide if you should take HUMIRA while you are pregnant or breastfeeding.
- have a baby and you were using HUMIRA during your pregnancy. Tell your baby's doctor before your baby receives any vaccines.

Tell your doctor about all the medicines you take, including prescription and over-the-counter medicines, vitamins, and herbal supplements.
Especially tell your doctor if you use:

- ORENCIA (abatacept), KINERET (anakinra), REMICADE (infliximab), ENBREL (etanercept), CIMZIA (certolizumab pegol) or SIMPONI (golimumab), because you should not use HUMIRA while you are also using one of these medicines.
- RITUXAN (rituximab). Your doctor may not want to give you HUMIRA if you have received RITUXAN (rituximab) recently.
- IMURAN (azathioprine) or PURINETHOL (6–mercaptopurine, 6-MP).

Keep a list of your medicines with you to show your doctor and pharmacist each time you get a new medicine.

How should I take HUMIRA?

- HUMIRA is given by an injection under the skin. Your doctor will tell you how often to take an injection of HUMIRA. This is based on your condition to be treated. **Do not inject HUMIRA more often than you were prescribed.**
- See the **Instructions for Use** inside the carton for complete instructions for the right way to prepare and inject HUMIRA.
- Make sure you have been shown how to inject HUMIRA before you do it yourself. You can call your doctor or 1-800-4HUMIRA (1-800-448-6472) if you have any questions about giving yourself an injection. Someone you know can also help you with your injection after they have been shown how to prepare and inject HUMIRA.
- **Do not** try to inject HUMIRA yourself until you have been shown the right way to give the injections. If your doctor decides that you or a caregiver may be able to give your injections of HUMIRA at home, you should receive training on the right way to prepare and inject HUMIRA.
- Do not miss any doses of HUMIRA unless your doctor says it is okay. If you forget to take HUMIRA, inject a dose as soon as you remember. Then, take your next dose at your regular scheduled time. This will put you back on schedule. In case you are not sure when to inject HUMIRA, call your doctor or pharmacist.
- If you take more HUMIRA than you were told to take, call your doctor.

What are the possible side effects of HUMIRA?

HUMIRA can cause serious side effects, including:

See "What is the most important information I should know about HUMIRA?"

- **Serious Infections.**
  Your doctor will examine you for TB and perform a test to see if you have TB. If your doctor feels that you are at risk for TB, you may be treated with medicine for TB before you begin treatment with HUMIRA and during treatment with HUMIRA. Even if your TB test is negative your doctor should carefully monitor you for TB infections while you are taking HUMIRA. People who had a negative TB skin test before receiving HUMIRA have developed active TB. Tell your doctor if you have any of the following symptoms while taking or after taking HUMIRA:
    - cough that does not go away
    - low grade fever
    - weight loss
    - loss of body fat and muscle (wasting)
- **Hepatitis B infection in people who carry the virus in their blood.**
  If you are a carrier of the hepatitis B virus (a virus that affects the liver), the virus can become active while you use HUMIRA. Your doctor should do blood tests before you start treatment, while you are using HUMIRA, and for several months after you stop treatment with HUMIRA. Tell your doctor if you have any of the following symptoms of a possible hepatitis B infection:
    - muscle aches

- muscle aches
- feel very tired
- dark urine
- skin or eyes look yellow
- little or no appetite
- vomiting
- clay-colored bowel movements
- fever
- chills
- stomach discomfort
- skin rash

- **Allergic reactions.** Allergic reactions can happen in people who use HUMIRA. Call your doctor or get medical help right away if you have any of these symptoms of a serious allergic reaction:
  - hives
  - swelling of your face, eyes, lips or mouth
  - trouble breathing
- **Nervous system problems.** Signs and symptoms of a nervous system problem include: numbness or tingling, problems with your vision, weakness in your arms or legs, and dizziness.
- **Blood problems.** Your body may not make enough of the blood cells that help fight infections or help to stop bleeding. Symptoms include a fever that does not go away, bruising or bleeding very easily, or looking very pale.
- **New heart failure or worsening of heart failure you already have. Call your doctor right away** if you get new worsening symptoms of heart failure while taking HUMIRA, including:
  - shortness of breath
  - sudden weight gain
  - swelling of your ankles or feet
- **Immune reactions including a lupus-like syndrome.** Symptoms include chest discomfort or pain that does not go away, shortness of breath, joint pain, or a rash on your cheeks or arms that gets worse in the sun. Symptoms may improve when you stop HUMIRA.
- **Liver problems.** Liver problems can happen in people who use TNF-blocker medicines. These problems can lead to liver failure and death. Call your doctor right away if you have any of these symptoms:
  - feel very tired
  - poor appetite or vomiting
  - skin or eyes look yellow
  - pain on the right side of your stomach (abdomen)
- **Psoriasis.** Some people using HUMIRA had new psoriasis or worsening of psoriasis they already had. Tell your doctor if you develop red scaly patches or raised bumps that are filled with pus. Your doctor may decide to stop your treatment with HUMIRA.

Call your doctor or get medical care right away if you develop any of the above symptoms. Your treatment with HUMIRA may be stopped.

The most common side effects of HUMIRA include:

- injection site reactions: redness, rash, swelling, itching, or bruising. These symptoms usually will go away within a few days. Call your doctor right away if you have pain, redness or swelling around the injection site that does not go away within a few days or gets worse.
- upper respiratory infections (including sinus infections).
- headaches.
- rash.

These are not all the possible side effects with HUMIRA. Tell your doctor if you have any side effect that bothers you or that does not go away. Ask your doctor or pharmacist for more information.
Call your doctor for medical advice about side effects. You may report side effects to FDA at 1-800-FDA-1088.

How should I store HUMIRA?

- Store HUMIRA in the refrigerator at 36ºF to 46ºF (2ºC to 8ºC). Store HUMIRA in the original carton until use to protect it from light.
- **Do not freeze HUMIRA.** Do not use HUMIRA if frozen, even if it has been thawed.
- Refrigerated HUMIRA may be used until the expiration date printed on the HUMIRA carton, dose tray, Pen or prefilled syringe. Do not use HUMIRA after the expiration date.
- If needed, for example when you are traveling, you may also store HUMIRA at room temperature up to 77°F (25°C) for up to 14 days. Store HUMIRA in the original carton until use to protect it from light.
- Throw away HUMIRA if it has been kept at room temperature and not been used within 14 days.
- Record the date you first remove HUMIRA from the refrigerator in the spaces provided on the carton and dose tray.
- Do not store HUMIRA in extreme heat or cold.
- Do not use a Pen or prefilled syringe if the liquid is cloudy, discolored, or has flakes or particles in it.
- Do not drop or crush HUMIRA. The prefilled syringe is glass.

Keep HUMIRA, injection supplies, and all other medicines out of the reach of children.

General information about the safe and effective use of HUMIRA.
Medicines are sometimes prescribed for purposes other than those listed in a Medication Guide. Do not use HUMIRA for a condition for which it was not prescribed. Do not give HUMIRA to other people, even if they have the same condition. It may harm them.
This Medication Guide summarizes the most important information about HUMIRA. If you would like more information, talk with your doctor. You can ask your pharmacist or doctor for information about HUMIRA that is written for health professionals.

What are the ingredients in HUMIRA?
Active ingredient: adalimumab

HUMIRA Pen 40 mg/0.8 mL, HUMIRA 40 mg/0.8 mL prefilled syringe, HUMIRA 20 mg/0.4 mL prefilled syringe, HUMIRA 10 mg/0.2 mL prefilled syringe, and HUMIRA 40 mg/0.8 mL institutional use vial:

Inactive ingredients: citric acid monohydrate, dibasic sodium phosphate dihydrate, mannitol, monobasic sodium phosphate dihydrate, polysorbate 80, sodium chloride, sodium citrate and Water for Injection. Sodium hydroxide is added as necessary to adjust pH.

HUMIRA Pen 80 mg/0.8 mL, HUMIRA 80 mg/0.8 mL prefilled syringe, HUMIRA Pen 40 mg/0.4 mL, HUMIRA 40 mg/0.4 mL prefilled syringe, HUMIRA 20 mg/0.2 mL prefilled syringe and HUMIRA 10 mg/0.1 mL prefilled syringe:

Inactive ingredients: mannitol, polysorbate 80, and Water for Injection.

Manufactured by: AbbVie Inc., North Chicago, IL 60064, U.S.A.
For more information go to www.HUMIRA.com or you can enroll in a patient support program by calling 1-800-4HUMIRA (1-800-448-6472).
US License Number 1889

Manufactured for: Cordavis Trading Limited, Dublin, Ireland

This Medication Guide has been approved by the U.S. Food and Drug Administration.<br>20082821	Revised: 11/2023

INSTRUCTIONS FOR USE
HUMIRA® (Hu-MARE-ah)
(adalimumab)
40 mg/0.4 mL
Single-Dose Pen

Before Injecting: Your healthcare provider should show you how to use HUMIRA before you use it for the first time. Call your healthcare provider or **1-800-4HUMIRA** (1-800-448-6472) if you need help.

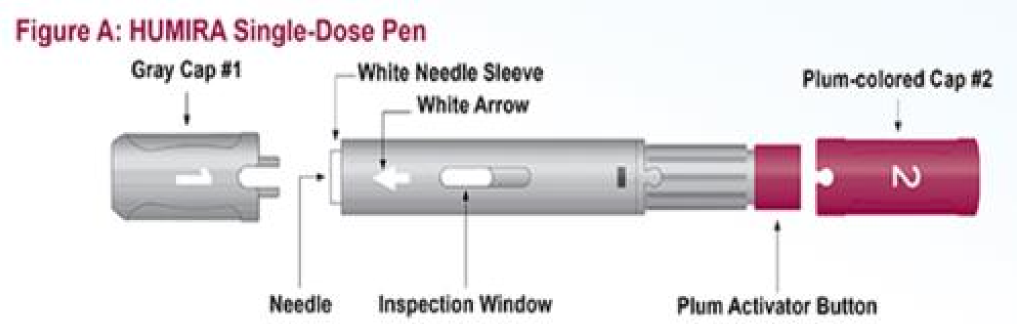

Important Information You Need to Know Before Injecting HUMIRA

Do not use the Pen and call your healthcare provider or pharmacist if:

- Liquid is cloudy, discolored, or has flakes or particles in it
- Expiration date has passed
- Liquid has been frozen (even if thawed) or left in direct sunlight
- The Pen has been dropped or crushed

Keep the caps on until right before your injection.
How should I store HUMIRA?

- Store HUMIRA in the refrigerator between 36°F to 46°F (2°C to 8°C).
- Store HUMIRA in the original carton until use to protect it from light.
- **Do not freeze**
- Refrigerated HUMIRA may be used until the expiration date printed on the HUMIRA carton, dose tray or Pen.
- If needed, for example when you are traveling, you may also store HUMIRA at room temperature up to 77°F (25°C) for up to **14** days.
- Throw away HUMIRA if it has been kept at room temperature and not used within **14** days.
- Record the date you first remove HUMIRA from the refrigerator in the spaces provided on the carton and dose tray.
- Do not store HUMIRA in extreme heat or cold.

Keep HUMIRA, injection supplies, and all other medicines out of reach of children.

Read Instructions on All Pages Before Using the HUMIRA Pen

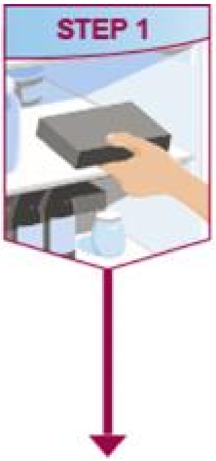

Take HUMIRA out of the refrigerator.
Leave HUMIRA at room temperature for **15 to 30 minutes** before injecting.

- **Do not** remove the Gray Cap (Cap #1) or Plum-colored Cap (Cap #2) while allowing HUMIRA to reach room temperature
- **Do not** warm HUMIRA in any other way. For example, **do not** warm it in a microwave or in hot water.
- **Do not** use the Pen if liquid has been frozen (even if thawed)

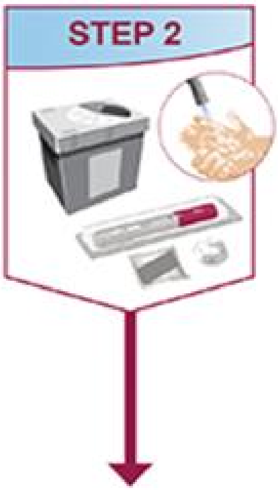

Check expiration date on the Pen label.
Do not use the Pen if expiration date has passed.
Place the following on a clean, flat surface:

- 1 single-dose Pen and alcohol swab
- 1 cotton ball or gauze pad (not included)
- Puncture-resistant sharps disposal container (not included). See Step 9 at the end of this Instructions for Use for instructions on how to throw away (dispose of) your HUMIRA Pen

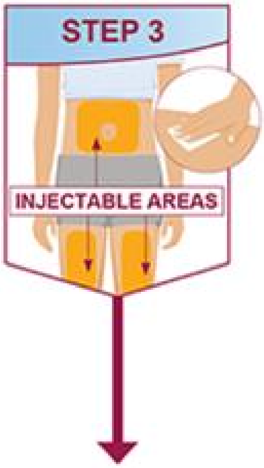

Wash and dry your hands.

Choose an injection site:

- On the front of your thighs or
- Your abdomen (belly) at least 2 inches from your navel (belly button)
- Different from your last injection site

Wipe the injection site in a circular motion with the alcohol swab.

- **Do not** inject through clothes
- **Do not** inject into skin that is sore, bruised, red, hard, scarred, has stretch marks, or areas with psoriasis plaques

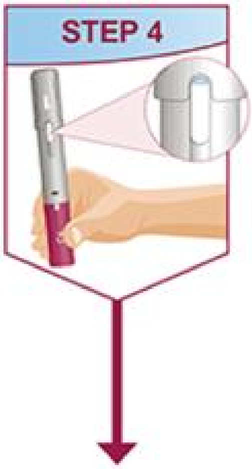

Hold the Pen with the Gray Cap #1 facing up. **Check** the window.

- It is normal to see 1 or more bubbles in the window
- Make sure the liquid is clear and colorless
- **Do not** use the Pen if the liquid is cloudy, discolored, or has flakes or particles in it
- **Do not** use the Pen if it has been dropped or crushed

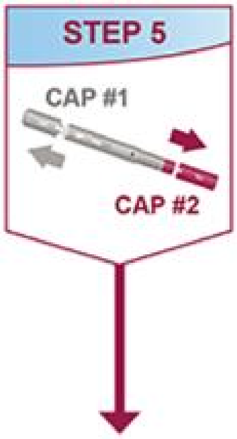

Pull the Gray Cap #1 straight off.
Throw the cap away.

- It is normal to see a few drops of liquid come out of the needle

Pull the Plum-colored Cap #2 straight off.
Throw the cap away.
Turn the Pen so that the white arrow points toward the injection site.

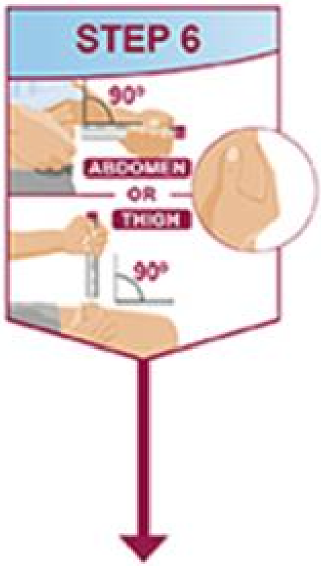

Squeeze the skin at your injection site to make a raised area and hold it firmly until the injection is complete.
Point the white arrow toward the injection site.
Place the white needle sleeve straight **(90° angle)** against the injection site.
Hold the Pen so that you can see the inspection window.
Do not press the plum activator button until you are ready to inject.

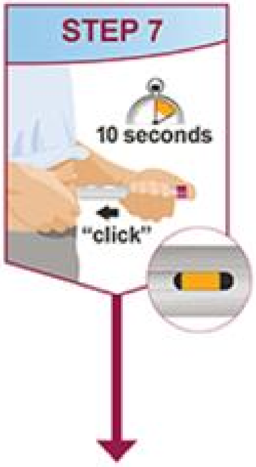

It is important that you firmly push the Pen down all the way against the injection site before starting the injection.
Keep pushing down to prevent the Pen from moving away from the skin during the injection.
Press the plum activator button and count slowly for **10** seconds.

- A loud "click" will signal the **start** of the injection
- **Keep pushing** the Pen **down firmly** against the injection site until the injection is complete
- Injection is complete when the yellow indicator has stopped moving

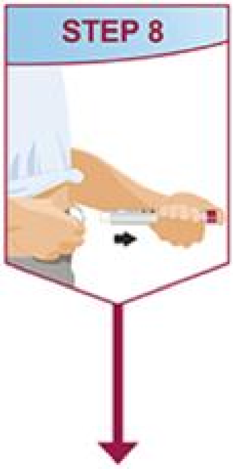

When the injection is completed, slowly pull the Pen from the skin. The white needle sleeve will cover the needle tip.

- A small amount of liquid on the injection site is normal

If there are more than a few drops of liquid on the injection site, call **1-800-4HUMIRA** (1-800-448-6472) for help.
After completing the injection, place a cotton ball or gauze pad on the skin of the injection site.

- **Do not** rub
- Slight bleeding at the injection site is normal

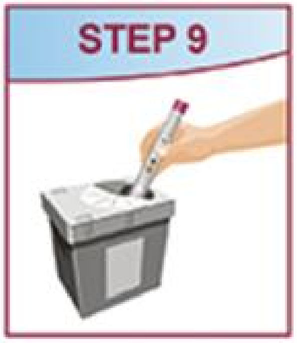

How should I dispose of the used HUMIRA Pen?

- Put your used needles, Pens, and sharps in a FDA cleared sharps disposal container right away after use. **Do not throw away (dispose of) loose needles, syringes, and the Pen in the household trash.**
- If you do not have a FDA cleared sharps disposal container, you may use a household container that is:
  - made of a heavy-duty plastic,
  - can be closed with a tight-fitting, puncture-resistant lid, without sharps being able to come out,
  - upright and stable during use,
  - leak-resistant, and
  - properly labeled to warn of hazardous waste inside the container.
- When your sharps disposal container is almost full, you will need to follow your community guidelines for the right way to dispose of your sharps disposal container. There may be state or local laws about how you should throw away used needles and syringes. For more information about safe sharps disposal, and for specific information about sharps disposal in the state that you live in, go to the FDA's website at: http://www.fda.gov/safesharpsdisposal.
- Do not dispose of your used sharps disposal container in your household trash unless your community guidelines permit this. Do not recycle your used sharps disposal container.

The Pen caps, alcohol swab, cotton ball or gauze pad, dose tray, and packaging may be placed in your household trash.

Questions About Using the HUMIRA Pen

What if I have not received in person training from a healthcare provider?

- Call your healthcare provider or **1-800-4HUMIRA (1-800-448-6472)** or visit www.HUMIRA.com if you need help

How do I know when the injection is complete?

- The yellow indicator has stopped moving. This takes up to **10** seconds.

What should I do if there are more than a few drops of liquid on the injection site?
- Call **1-800-4HUMIRA (1-800-448-6472)** for help

What if I do not have an FDA-cleared sharps disposal container or proper household container?
- Call **1-800-4HUMIRA (1-800-448-6472)** for a free FDA-cleared sharps disposal container

Always keep the Pen and the sharps disposal container out of reach of children.

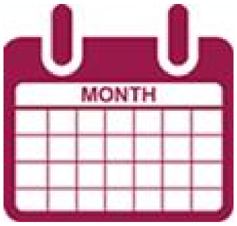

Keep a record of the dates and locations of your injections. To help remember when to take HUMIRA, mark your calendar ahead of time.

abbvie

This Instructions for Use has been approved by the U.S. Food and Drug Administration.

Revised 11/2023

Manufactured by AbbVie Inc. North Chicago, IL 60064 U.S.A.
US License Number 1889 20082820
Manufactured for: Cordavis Trading Limited, Dublin, Ireland

INSTRUCTIONS FOR USE
HUMIRA® (Hu-MARE-ah)
(adalimumab)
Packages containing 80 mg/0.8 mL
Single-Dose Pen

Before Injecting: Your healthcare provider should show you how to use HUMIRA before you use it for the first time. Call your healthcare provider or **1-800-4HUMIRA** (1-800-448-6472) if you need help.

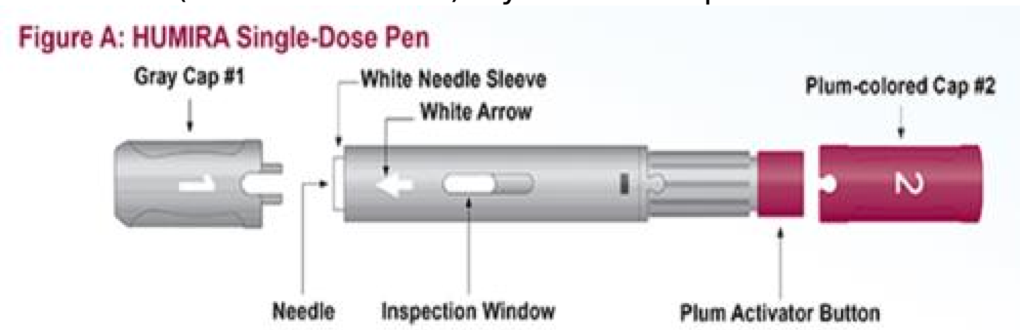

Figure A: HUMIRA Single-Dose Pen

Important Information You Need to Know Before Injecting HUMIRA

Do not use the Pen and call your healthcare provider or pharmacist if:

- Liquid is cloudy, discolored, or has flakes or particles in it
- Expiration date has passed
- Liquid has been frozen (even if thawed) or left in direct sunlight
- The Pen has been dropped or crushed

Keep the caps on until right before your injection.

How should I store HUMIRA?

- Store HUMIRA in the refrigerator between 36°F to 46°F (2°C to 8°C).
- Store HUMIRA in the original carton until use to protect it from light.
- **Do not freeze**
- Refrigerated HUMIRA may be used until the expiration date printed on the HUMIRA carton, dose tray or Pen.
- If needed, for example when you are traveling, you may also store HUMIRA at room temperature up to 77°F (25°C) for up to **14** days.
- Throw away HUMIRA if it has been kept at room temperature and not used within **14** days.
- Record the date you first remove HUMIRA from the refrigerator in the spaces provided on the carton and dose tray.
- Do not store HUMIRA in extreme heat or cold.

Keep HUMIRA, injection supplies, and all other medicines out of reach of children.

Read Instructions on All Pages Before Using the HUMIRA Pen

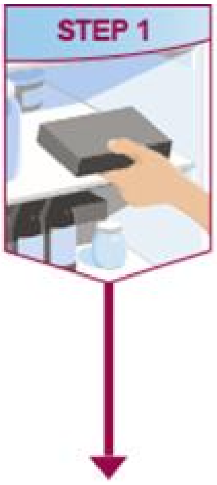

Take HUMIRA out of the refrigerator.
Leave HUMIRA at room temperature for **15 to 30 minutes** before injecting.

- **Do not** remove the Gray Cap (Cap #1) or Plum-colored Cap (Cap #2) while allowing HUMIRA to reach room temperature
- **Do not** warm HUMIRA in any other way. For example, **do not** warm it in a microwave or in hot water
- **Do not** use the Pen if liquid has been frozen (even if thawed)

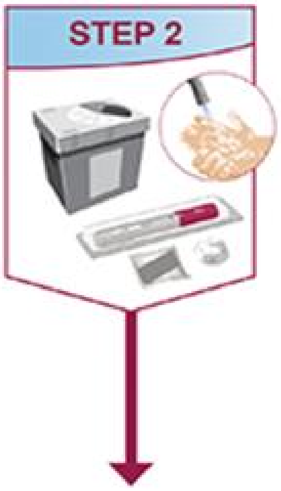

Check expiration date on the Pen label.
Do not use the Pen if expiration date has passed.
Place the following on a clean, flat surface:

- 1 single-dose Pen and alcohol swab
- 1 cotton ball or gauze pad (not included)
- Puncture-resistant sharps disposal container (not included). See Step 9 at the end of this Instructions for Use for instructions on how to throw away (dispose of) your HUMIRA Pen

Wash and dry your hands.

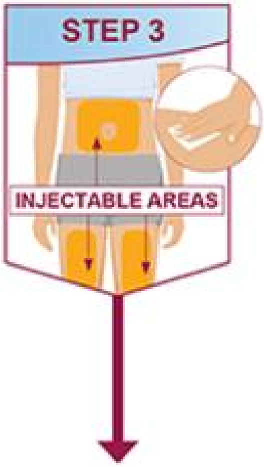

Choose an injection site:

- On the front of your thighs or
- Your abdomen (belly) at least 2 inches from your navel (belly button)
- Different from your last injection site

Wipe the injection site in a circular motion with the alcohol swab.

- **Do not** inject through clothes
- **Do not** inject into skin that is sore, bruised, red, hard, scarred, has stretch marks, or areas with psoriasis plaques

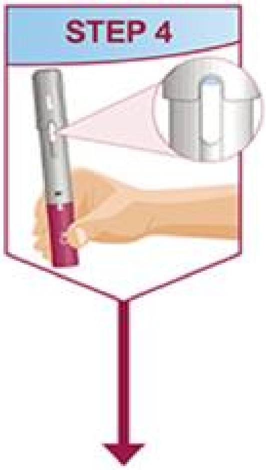

Hold the Pen with the Gray Cap #1 facing up. **Check** the window.

- It is normal to see 1 or more bubbles in the window
- Make sure the liquid is clear and colorless
- **Do not** use the Pen if the liquid is cloudy, discolored, or has flakes or particles in it
- **Do not** use the Pen if it has been dropped or crushed

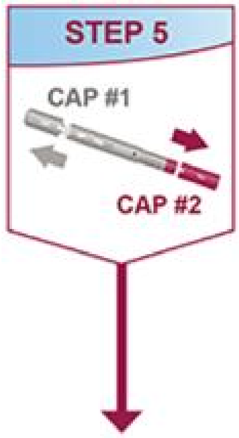

Pull the Gray Cap #1 straight off.
Throw the cap away.

- It is normal to see a few drops of liquid come out of the needle

Pull the Plum-colored Cap #2 straight off.
Throw the cap away.
Turn the Pen so that the white arrow points toward the injection site.

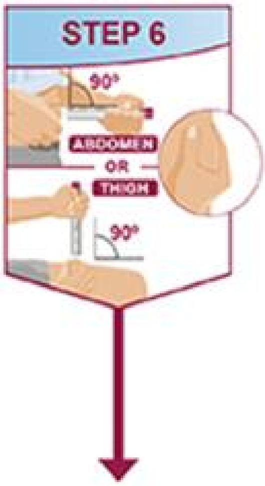

Squeeze the skin at your injection site to make a raised area and hold it firmly until the injection is complete.
Point the white arrow toward the injection site.
Place the white needle sleeve straight **(90° angle)** against the injection site.
Hold the Pen so that you can see the inspection window.

Do not press the plum activator button until you are ready to inject.

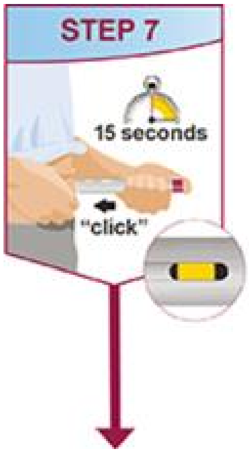

It is important that you firmly push the Pen down all the way against the injection site before starting the injection.
Press the plum activator button and count slowly for **15** seconds.

- A loud "click" will signal the **start** of the injection
- **Keep pushing** the Pen **down firmly** against the injection site until the injection is complete
- Injection is complete when the yellow indicator has stopped moving

[illegible]

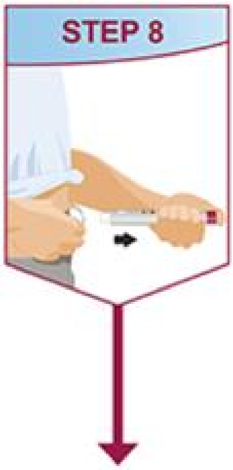

When the injection is completed, slowly pull the Pen from the skin. The white needle sleeve will cover the needle tip.

- A small amount of liquid on the injection site is normal

If there are more than a few drops of liquid on the injection site, call **1-800-4HUMIRA** (1-800-448-6472) for help. After completing the injection, place a cotton ball or gauze pad on the skin of the injection site.

- **Do not** rub
- Slight bleeding at the injection site is normal

How should I dispose of the used HUMIRA Pen?

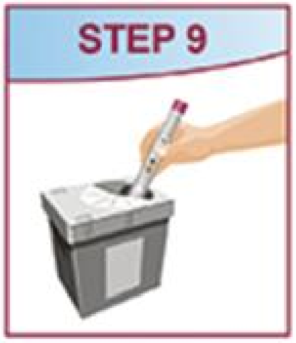

- Put your used needles, Pens, and sharps in a FDA cleared sharps disposal container right away after use. **Do not throw away (dispose of) loose needles, syringes, and the Pen in the household trash.**
- If you do not have a FDA-cleared sharps disposal container, you may use a household container that is:
  - made of a heavy-duty plastic,
  - can be closed with a tight-fitting, puncture-resistant lid, without sharps being able to come out,
  - upright and stable during use,
  - leak-resistant, and
  - properly labeled to warn of hazardous waste inside the container.

- When your sharps disposal container is almost full, you will need to follow your community guidelines for the right way to dispose of your sharps disposal container. There may be state or local laws about how you should throw away used needles and syringes. For more information about safe sharps disposal, and for specific information about sharps disposal in the state that you live in, go to the FDA's website at: http://www.fda.gov/safesharpsdisposal.
- Do not dispose of your used sharps

disposal container in your household trash unless your community guidelines permit this. Do not recycle your used sharps disposal container.

The Pen caps, alcohol swab, cotton ball or gauze pad, dose tray, and packaging may be placed in your household trash.

Questions About Using the HUMIRA Pen

What if I have not received in-person training from a healthcare provider?

- Call your healthcare provider or **1-800-4HUMIRA (1-800-448-6472)** or visit www.HUMIRA.com if you need help

How do I know when the injection is complete?

- The yellow indicator has stopped moving. This takes up to **15** seconds.

What should I do if there are more than a few drops of liquid on the injection site?

- Call **1-800-4HUMIRA (1-800-448-6472)** for help

What if I do not have an FDA-cleared sharps disposal container or proper household container?

- Call **1-800-4HUMIRA (1-800-448-6472)** for a free FDA-cleared sharps disposal container

Always keep the Pen and the sharps disposal container out of reach of children.

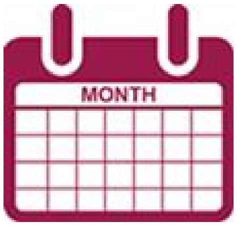

Keep a record of the dates and locations of your injections. To help remember when to take HUMIRA, mark your calendar ahead of time.

This Instructions for Use has been approved by the U.S. Food and Drug Administration.

Revised 11/2023

Manufactured by AbbVie Inc. North Chicago, IL 60064 U.S.A.
US License Number 1889 20081409
Manufactured for: Cordavis Trading Limited, Dublin, Ireland

INSTRUCTIONS FOR USE

HUMIRA® (Hu-MARE-ah)

(adalimumab)
80 mg/0.8 mL, 40 mg/0.4 mL, 20 mg/0.2 mL and 10 mg/0.1 mL
Single-Dose Prefilled Syringe

Before Injecting: Your healthcare provider should show you how to use HUMIRA before you use it for the first time. Call your healthcare provider or **1-800-4HUMIRA** (1-800-448-6472) if you need help.

Figure A: HUMIRA Single-Dose Prefilled Syringe

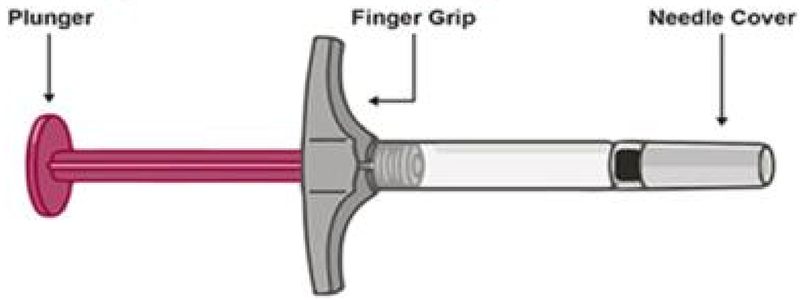

Important Information You Need to Know Before Injecting HUMIRA

Do not use the prefilled syringe and call your healthcare provider or pharmacist if:

- Liquid is cloudy, discolored, or has flakes or particles in it
- Expiration date has passed
- Liquid has been frozen (even if thawed) or left in direct sunlight
- The prefilled syringe has been dropped or crushed

Keep the needle cover on until right before your injection.

How should I store HUMIRA?

- Store HUMIRA in the refrigerator between 36°F to 46°F (2°C to 8°C).
- Store HUMIRA in the original carton until use to protect it from light.
- **Do not freeze**
- Refrigerated HUMIRA may be used until the expiration date printed on the HUMIRA carton, dose tray or prefilled syringe.
- If needed, for example when you are traveling, you may also store HUMIRA at room temperature up to 77°F (25°C) for up to **14** days.
- Throw away HUMIRA if it has been kept at room temperature and not used within **14** days.
- Record the date you first remove HUMIRA from the refrigerator in the spaces provided on the carton and dose tray.
- Do not store HUMIRA in extreme heat or cold.

Keep HUMIRA, injection supplies, and all other medicines out of reach of children.

Read Instructions on All Pages Before Using the HUMIRA Single-Dose Prefilled Syringe

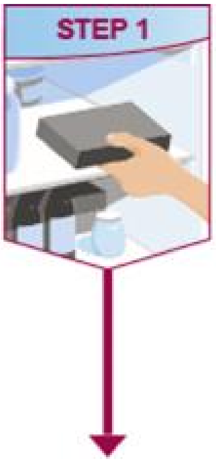

Take HUMIRA out of the refrigerator.
Leave HUMIRA at room temperature for **15 to 30 minutes** before injecting.

- **Do not** remove the needle cover while allowing HUMIRA to reach room temperature
- **Do not** warm HUMIRA in any other way. For example, **do not** warm it in a microwave or in hot water.
- **Do not** use the prefilled syringe if liquid has been frozen (even if thawed)

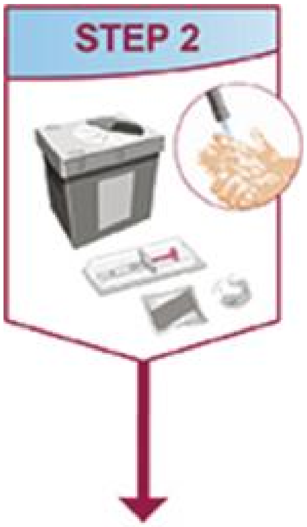

Check expiration date on the prefilled syringe label. **Do not** use the prefilled syringe if expiration date has passed.
Place the following on a clean, flat surface:

- 1 single-dose prefilled syringe and alcohol swab
- 1 cotton ball or gauze pad (not included)
- Puncture-resistant sharps disposal container (not included). See Step 9 at the end of this Instructions for Use for instructions on how to throw away (dispose of) your prefilled syringe

Wash and dry your hands.

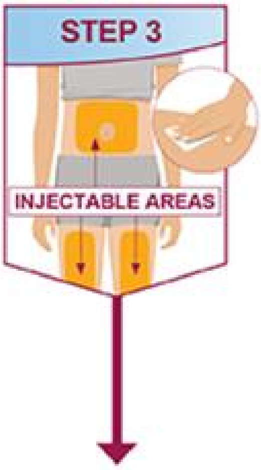

Choose an injection site:

- On the front of your thighs or
- Your abdomen (belly) at least 2 inches from your navel (belly button)
- Different from your last injection site

Wipe the injection site in a circular motion with the alcohol swab.

- **Do not** inject through clothes
- **Do not** inject into skin that is sore, bruised, red, hard, scarred, has stretch marks, or areas with psoriasis plaques

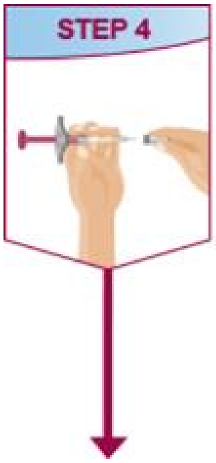

Hold the prefilled syringe in one hand.
Gently pull the needle cover straight off with the other hand.

- Throw the needle cover away
- **Do not** touch the needle with your fingers or let the needle touch anything

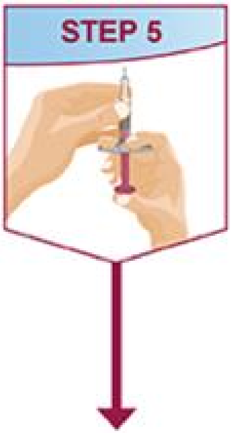

Hold the prefilled syringe with the needle facing up.

- **Hold** the prefilled syringe at eye level with one hand so you can see the air in the prefilled syringe
- Using your other hand, **slowly push** the plunger in to push the air out through the needle.
- You may see a drop of liquid at the end of the needle. This is normal.

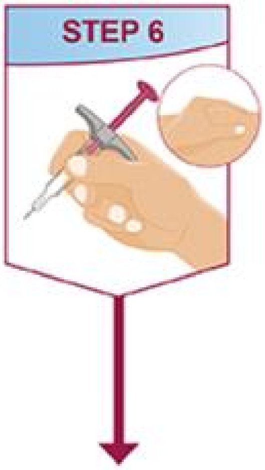

Hold the body of the prefilled syringe in one hand between the thumb and index fingers. Hold the prefilled syringe in your hand like a pencil.
Do not pull back on the plunger at any time.

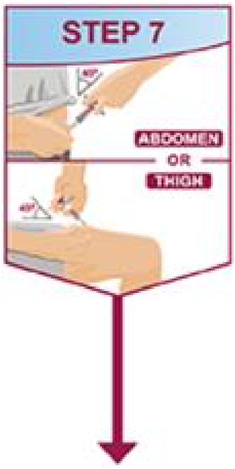

Gently squeeze the area of cleaned skin at your injection site with your other hand. Hold the skin firmly.

Insert the needle into the skin at about a 45-degree angle using a quick, dart-like motion.

- After the needle is in, let go of the skin.

Slowly push the plunger all the way in until all of the liquid is injected and the prefilled syringe is empty.

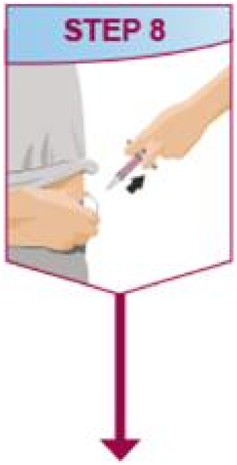

When the injection is completed, slowly pull the needle out of the skin while keeping the prefilled syringe at the same angle.
After completing the injection, place a cotton ball or gauze pad on the skin of the injection site.

- **Do not** rub
- Slight bleeding at the injection site is normal

How should I dispose of the used HUMIRA prefilled syringe?

- Put your used needles, syringes, and sharps in a FDA-cleared sharps disposal container right away after use. **Do not throw away (dispose of) loose needles and syringes in the household trash.**
- If you do not have a FDA-cleared sharps disposal container, you may use a household container that is:
  - made of a heavy-duty plastic,
  - can be closed with a tight-fitting, [illegible]

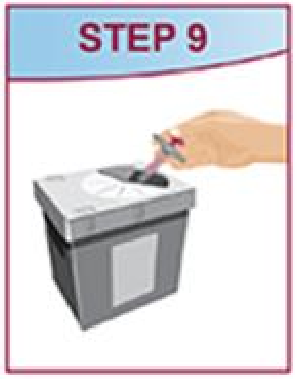

- puncture-resistant lid, without sharps being able to come out,
- upright and stable during use,
- leak-resistant, and
- properly labeled to warn of hazardous waste inside the container.

- When your sharps disposal container is almost full, you will need to follow your community guidelines for the right way to dispose of your sharps disposal container. There may be state or local laws about how you should throw away used needles and syringes. For more information about safe sharps disposal, and for specific information about sharps disposal in the state that you live in, go to the FDA's website at: http://www.fda.gov/safesharpsdisposal.
- Do not dispose of your used sharps disposal container in your household trash unless your community guidelines permit this. Do not recycle your used sharps disposal container.

The needle cover, alcohol swab, cotton ball or gauze pad, dose tray, and packaging may be placed in your household trash.

Questions About Using the HUMIRA Single-Dose Prefilled Syringe

What if I have not received in-person training from a healthcare provider?
- Call your healthcare provider or **1-800-4HUMIRA (1-800-448-6472)** or visit www.HUMIRA.com if you need help

What if I do not have an FDA-cleared sharps disposal container or proper household container?
- Call **1-800-4HUMIRA (1-800-448-6472)** for a free FDA-cleared sharps disposal container

Always keep the prefilled syringe and the sharps disposal container out of the reach of children.

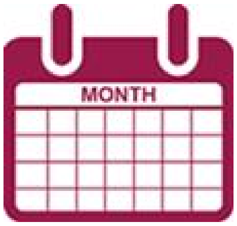

Keep a record of the dates and locations of your injections. To help remember when to take HUMIRA, mark your calendar ahead of time.

This Instructions for Use has been approved by the U.S. Food and Drug Administration.

Revised 11/2023

Manufactured by AbbVie Inc. North Chicago, IL 60064 U.S.A.
US License Number 1889 20082850
Manufactured for: Cordavis Trading Limited, Dublin, Ireland

Principal Display Panel

NDC: 83457-554-02
2 SINGLE-DOSE PREFILLED PENS
HUMIRA® PEN
adalimumab
40 mg/0.4 mL
FOR SUBCUTANEOUS USE ONLY
29 GUAGE NEEDLE
ATTENTION PHARMACIST: Each patient is required to receive the enclosed Medication Guide.
The entire carton is to be dispensed as a unit.
Return to pharmacy if dose tray seal is broken or missing.

THIS CARTON CONTAINS:

- 2 dose trays (each containing 1 single-dose prefilled pen with 29 gauge ½ inch length fixed needle)
- 2 alcohol preps • 1 Medication Guide • 1 package insert • 1 Instruction for Use

Manufactured For:
cordavis™
HUMIRA.COM
Rx only
abbvie

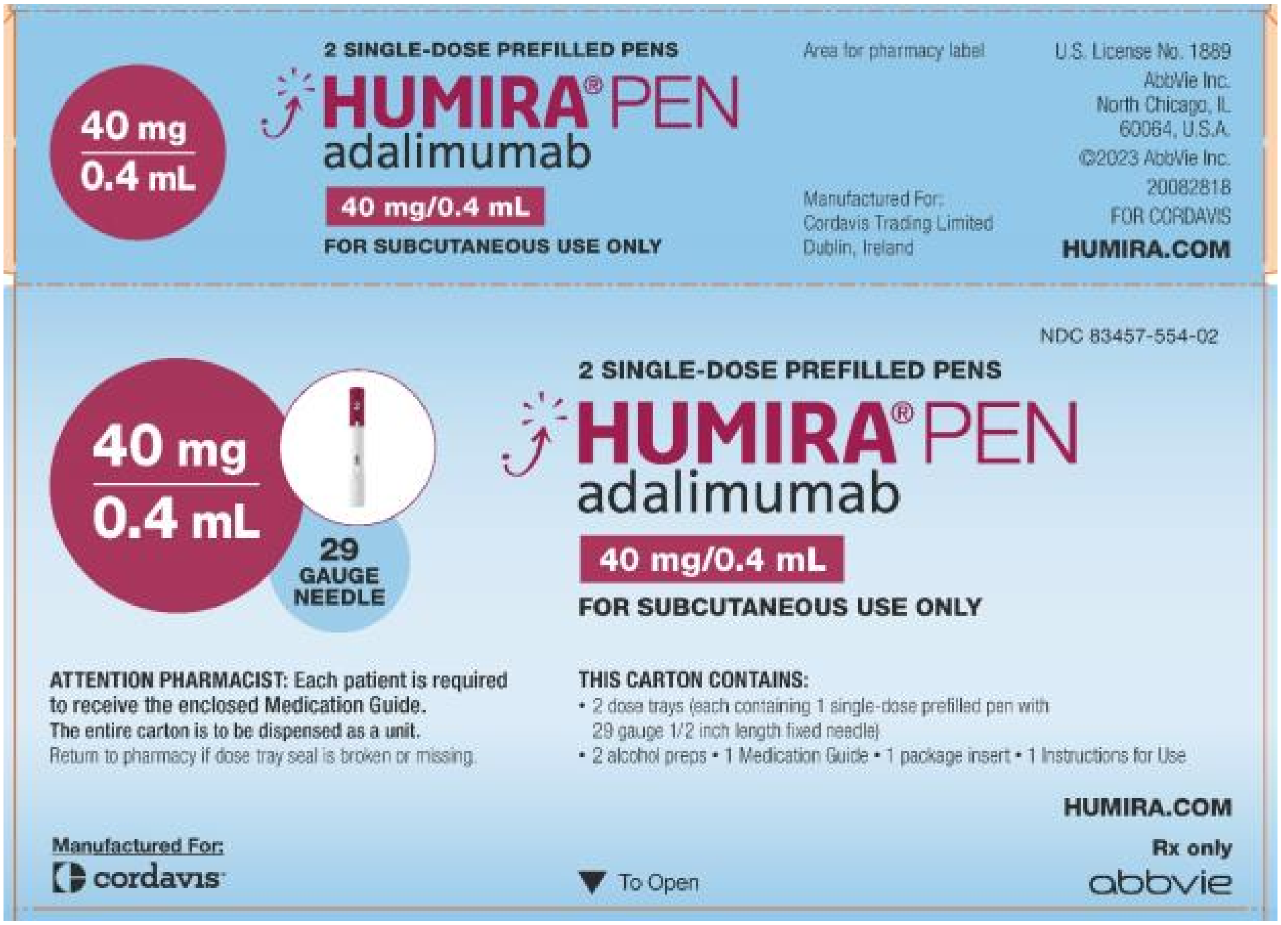

NDC: 83457-124-02

2 SINGLE-DOSE PREFILLED PENS
HUMIRA® PEN
adalimumab
80 mg/0.8 mL
FOR SUBCUTANEOUS USE ONLY
29 GUAGE NEEDLE
ATTENTION PHARMACIST: Each patient is required to receive the enclosed Medication Guide.
Needle cover for syringe is not made with natural rubber latex.
The entire carton is to be dispensed as a unit.
Return to pharmacy if dose tray seal is broken or missing.

THIS CARTON CONTAINS:

- 2 dose trays (each containing 1 single-dose prefilled pen with 29 gauge ½ inch length fixed needle)
- 2 alcohol preps • 1 Medication Guide • 1 package insert • 1 Instruction for Use

Manufactured For:
cordavis™
HUMIRA.COM
Rx only
abbvie

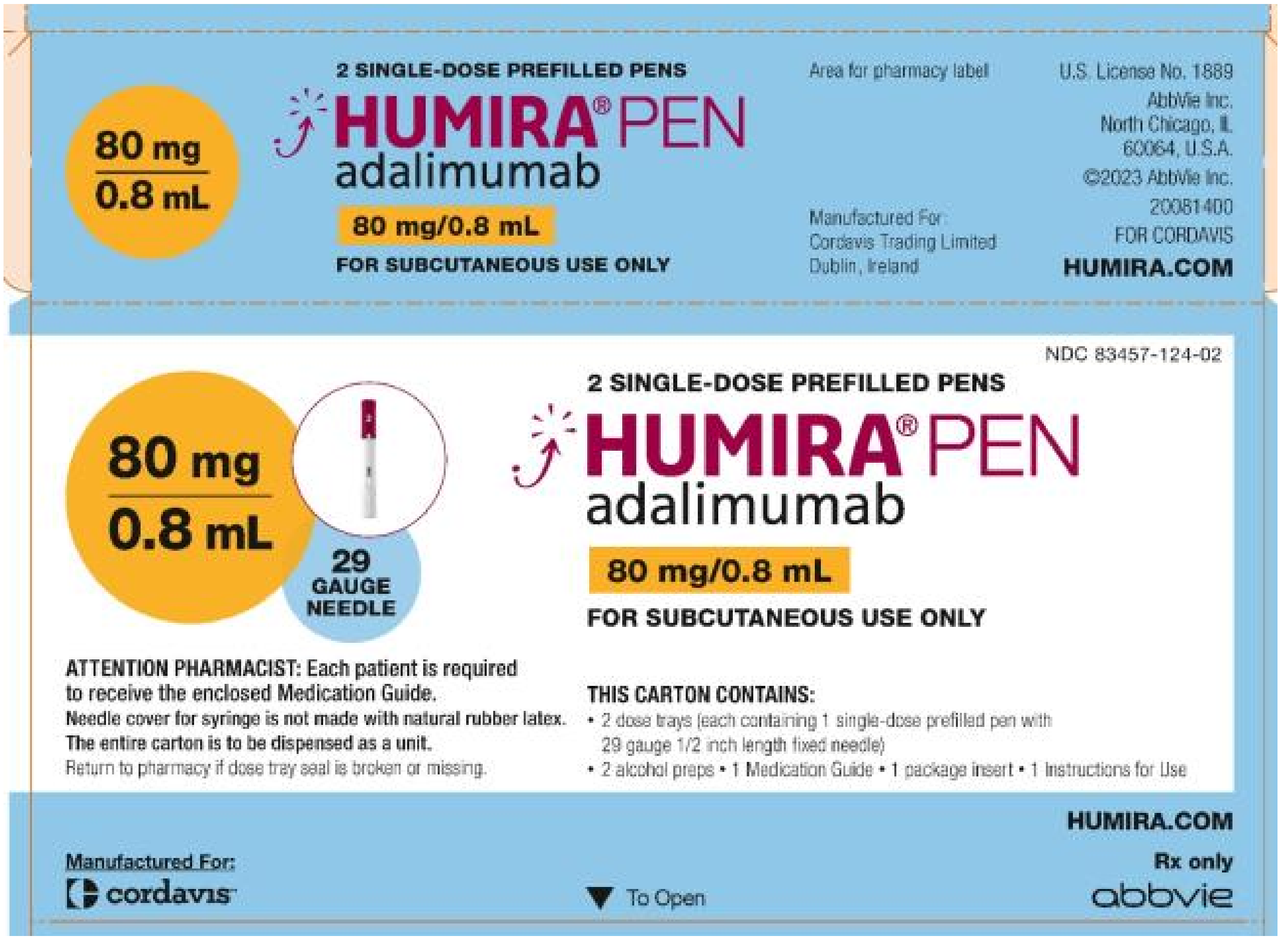

NDC: 83457-817-02

2 SINGLE-DOSE PREFILLED SYRINGES
HUMIRA®
adalimumab
10 mg/0.1 mL
FOR SUBCUTANEOUS USE ONLY
29 GUAGE NEEDLE
ATTENTION PHARMACIST: Each patient is required to receive the enclosed Medication Guide.
Needle cover for syringe is not made with natural rubber latex.
The entire carton is to be dispensed as a unit.
Return to pharmacy if dose tray seal is broken or missing.

THIS CARTON CONTAINS:

- 2 dose trays (each containing 1 single-dose prefilled pen with 29 gauge ½ inch length fixed needle)
- 2 alcohol preps • 1 Medication Guide • 1 package insert
- 1 Instruction for Use

Manufactured For:
cordavis™
HUMIRA.COM
Rx only
abbvie

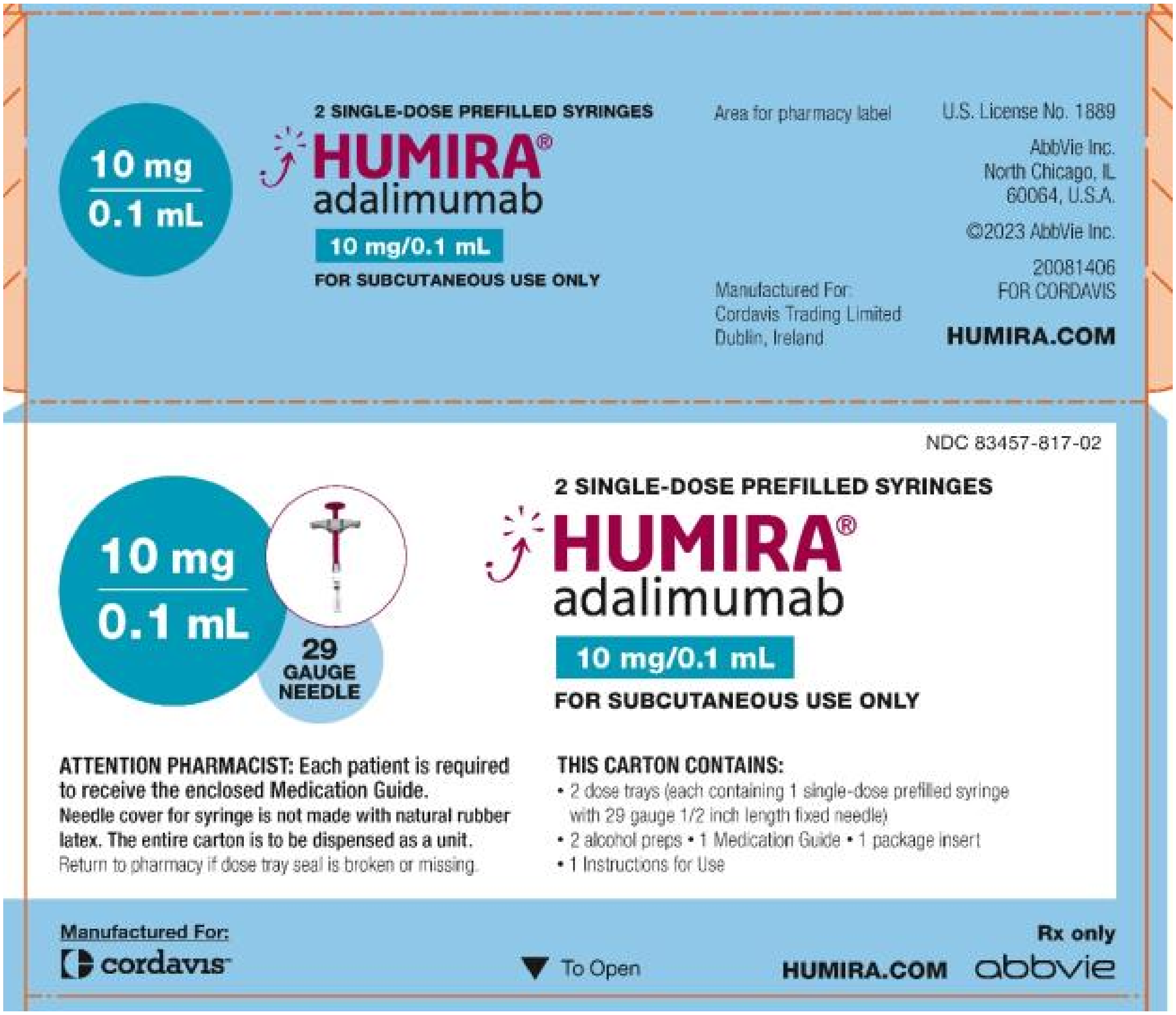

NDC: 83457-616-02

2 SINGLE-DOSE PREFILLED SYRINGES
HUMIRA®
adalimumab
20 mg/0.2 mL
FOR SUBCUTANEOUS USE ONLY
29 GUAGE NEEDLE
ATTENTION PHARMACIST: Each patient is required to receive the enclosed Medication Guide.
Needle cover for syringe is not made with natural rubber latex.
The entire carton is to be dispensed as a unit.
Return to pharmacy if dose tray seal is broken or missing.

THIS CARTON CONTAINS:

- 2 dose trays (each containing 1 single-dose prefilled pen with 29 gauge ½ inch length fixed needle)
- 2 alcohol preps • 1 Medication Guide • 1 package insert
- 1 Instruction for Use

Manufactured For:
cordavis™
HUMIRA.COM
Rx only
abbvie

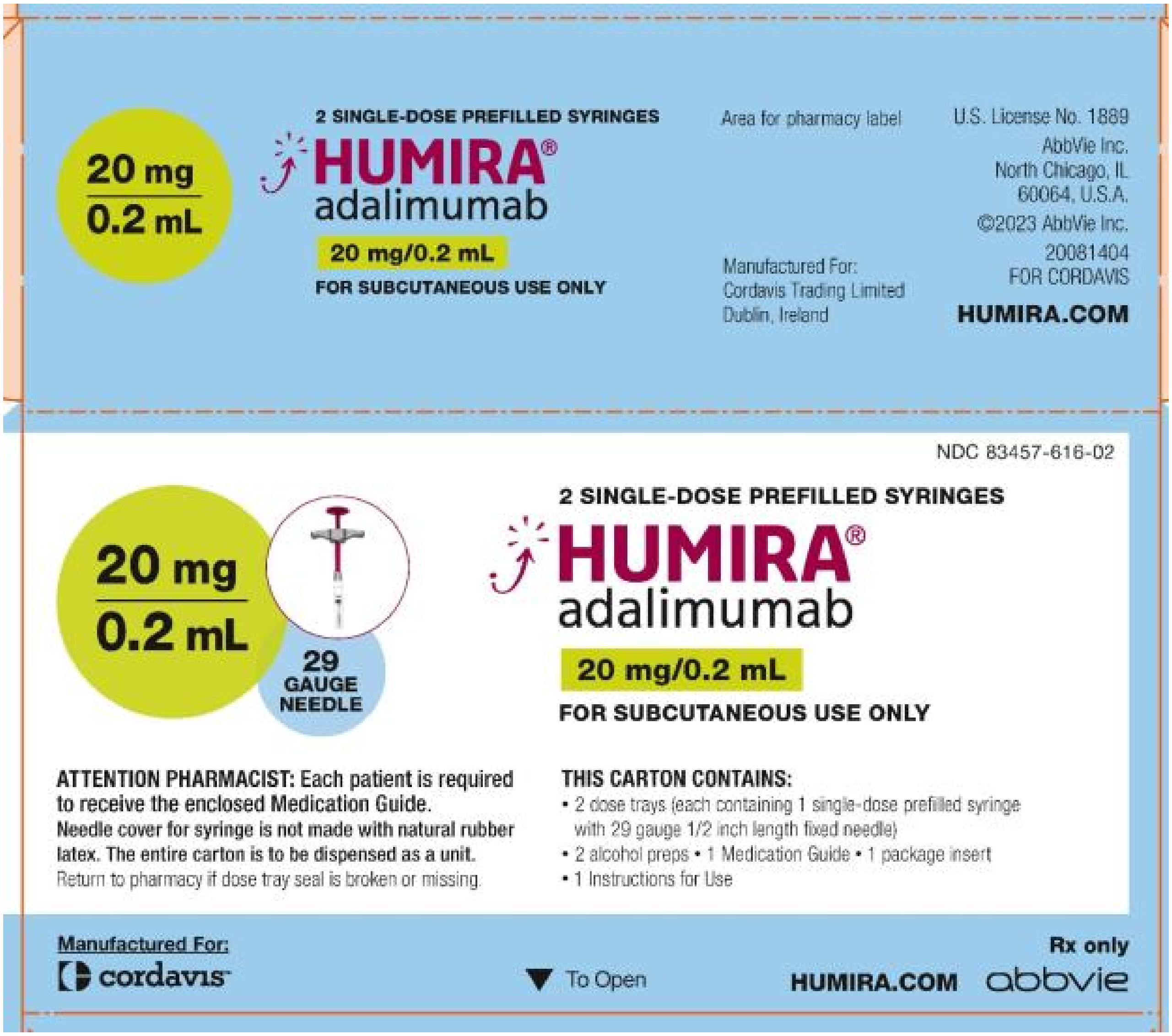

NDC: 83457-243-02

2 SINGLE-DOSE PREFILLED SYRINGES
HUMIRA®
adalimumab
40 mg/0.4 mL
FOR SUBCUTANEOUS USE ONLY
29 GUAGE NEEDLE
ATTENTION PHARMACIST: Each patient is required to receive the enclosed Medication Guide.
Needle cover for syringe is not made with natural rubber latex

THIS CARTON CONTAINS:

- 2 dose trays (each containing 1 single-dose prefilled pen with 29 gauge ½ inch length

fixed needle)
- 2 alcohol preps • 1 Medication Guide • 1 package insert
- 1 Instruction for Use

Manufactured For:
cordavis™
HUMIRA.COM
Rx only
abbvie

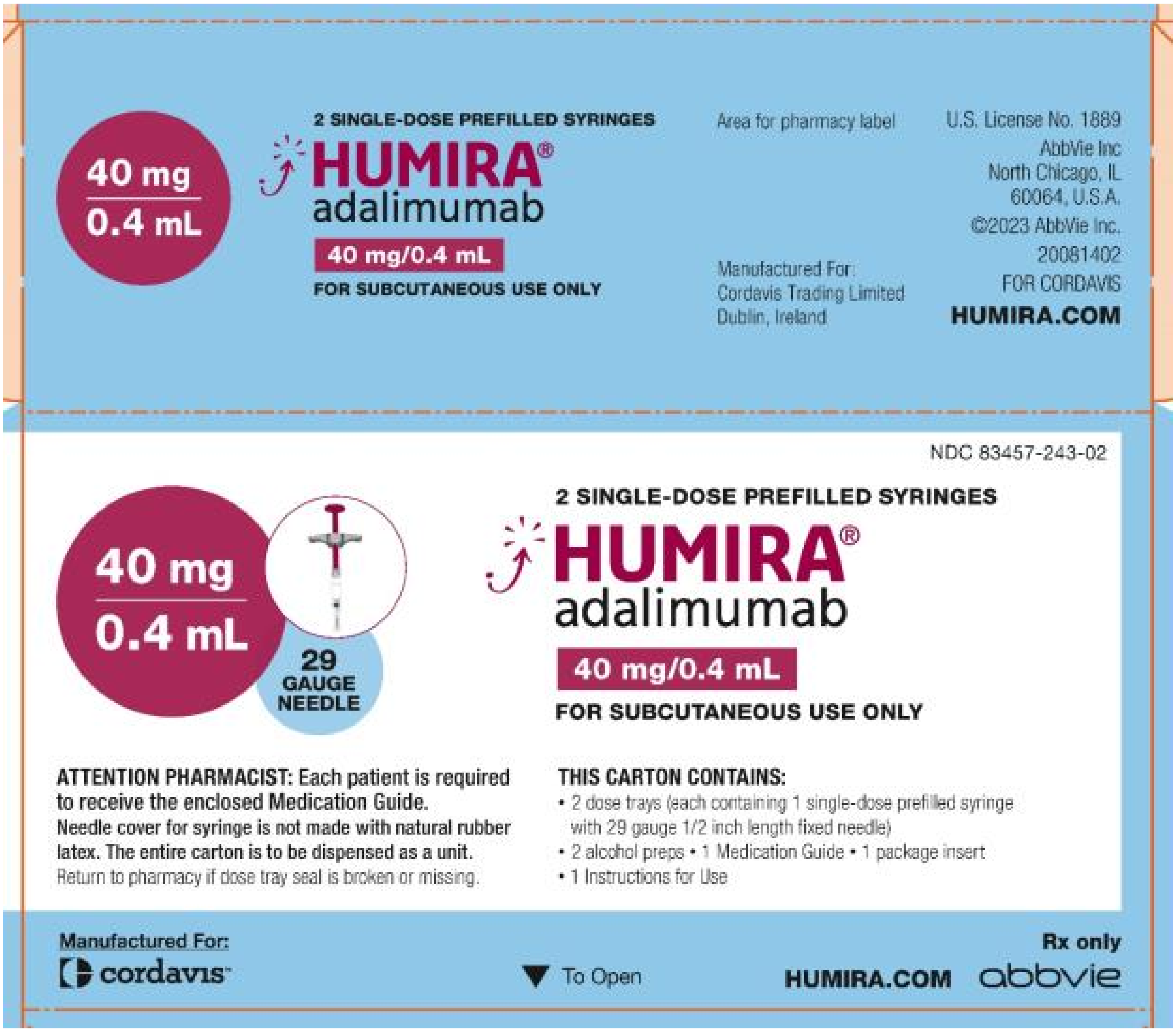

**HUMIRA** adalimumab kit			
**Product Information**			
**Product Type**	HUMAN PRESCRIPTION DRUG	**Item Code (Source)**	NDC:83457-554

Packaging

#	Item Code	Package Description	Marketing Start	Marketing End

#	Item Code	Package Description	Marketing Start Date	Marketing End Date
1	NDC:83457-554-02	2 in 1 CARTON	02/14/2024	
1		1 in 1 KIT; Type 0: Not a Combination Product		

Quantity of Parts		
**Part #**	**Package Quantity**	**Total Product Quantity**
**Part 1**	2 SYRINGE	0.8 mL in 2
**Part 2**	2 PACKET	2 mL in 2

Part 1 of 2

HUMIRA

adalimumab injection, solution

Product Information	
**Item Code (Source)**	NDC:83457-553
**Route of Administration**	SUBCUTANEOUS

Active Ingredient/Active Moiety		
**Ingredient Name**	**Basis of Strength**	**Strength**
**ADALIMUMAB** (UNII: FYS6T7F842) (ADALIMUMAB - UNII:FYS6T7F842)	ADALIMUMAB	40 mg in 0.4 mL

Inactive Ingredients	
**Ingredient Name**	**Strength**
**MANNITOL** (UNII: 3OWL53L36A)	16.8 mg in 0.4 mL
**POLYSORBATE 80** (UNII: 6OZP39ZG8H)	0.4 mg in 0.4 mL
**WATER** (UNII: 059QF0KO0R)	

Packaging				
**#**	**Item Code**	**Package Description**	**Marketing Start Date**	**Marketing End Date**
1	NDC:83457-553-01	1 in 1 TRAY		
1		0.4 mL in 1 SYRINGE; Type 3: Prefilled Biologic Delivery Device/System (syringe, patch, etc.)		

Marketing Information

Marketing Category	Application Number or Monograph Citation	Marketing Start Date	Marketing End Date

BLA	BLA125057	02/14/2024	

Part 2 of 2

ALCOHOL

isopropyl alcohol swab

Product Information	
**Item Code (Source)**	NDC:83457-927
**Route of Administration**	TOPICAL

Active Ingredient/Active Moiety

Ingredient Name	Basis of Strength	Strength
**ISOPROPYL ALCOHOL** (UNII: ND2M416302) (ISOPROPYL ALCOHOL - UNII:ND2M416302)	ISOPROPYL ALCOHOL	0.70 mL in 1 mL

Inactive Ingredients

Ingredient Name	Strength
**WATER** (UNII: 059QF0KO0R)	

Packaging

#	Item Code	Package Description	Marketing Start Date	Marketing End Date
**1**	NDC:83457-927-01	1 mL in 1 PACKET; Type 0: Not a Combination Product		

Marketing Information

Marketing Category	Application Number or Monograph Citation	Marketing Start Date	Marketing End Date
OTC Monograph Drug	M003	02/14/2024	

Marketing Information

Marketing Category	Application Number or Monograph Citation	Marketing Start Date	Marketing End Date
BLA	BLA125057	02/14/2024	

HUMIRA

adalimumab kit

Product Information			
**Product Type**	HUMAN PRESCRIPTION DRUG	**Item Code (Source)**	NDC:83457-124

Packaging				
**#**	**Item Code**	**Package Description**	**Marketing Start Date**	**Marketing End Date**
**1**	NDC:83457-124-02	2 in 1 CARTON	02/14/2024	
**1**		1 in 1 KIT; Type 0: Not a Combination Product		

Quantity of Parts		
**Part #**	**Package Quantity**	**Total Product Quantity**
**Part 1**	2 SYRINGE	1.6 mL in 2
**Part 2**	2 PACKET	2 mL in 2

Part 1 of 2

HUMIRA

adalimumab injection, solution

Product Information	
**Item Code (Source)**	NDC:83457-123
**Route of Administration**	SUBCUTANEOUS

Active Ingredient/Active Moiety		
**Ingredient Name**	**Basis of Strength**	**Strength**
**ADALIMUMAB** (UNII: FYS6T7F842) (ADALIMUMAB - UNII:FYS6T7F842)	ADALIMUMAB	80 mg in 0.8 mL

Inactive Ingredients	
**Ingredient Name**	**Strength**
**MANNITOL** (UNII: 3OWL53L36A)	33.6 mg in 0.8 mL
**POLYSORBATE 80** (UNII: 6OZP39ZG8H)	0.8 mg in 0.8 mL
**WATER** (UNII: 059QF0KO0R)	

Packaging				
**#**	**Item**	**Package Description**	**Marketing**	**Marketing**

#	Item Code	Package Description	Marketing Start Date	Marketing End Date
**1**	NDC:83457-123-01	1 in 1 TRAY		
**1**		0.8 mL in 1 SYRINGE; Type 3: Prefilled Biologic Delivery Device/System (syringe, patch, etc.)		

Marketing Information

Marketing Category	Application Number or Monograph Citation	Marketing Start Date	Marketing End Date
BLA	BLA125057	02/14/2024	

Part 2 of 2

ALCOHOL

isopropyl alcohol swab

Product Information

**Item Code (Source)**	NDC:83457-927
**Route of Administration**	TOPICAL

Active Ingredient/Active Moiety

Ingredient Name	Basis of Strength	Strength
**ISOPROPYL ALCOHOL** (UNII: ND2M416302) (ISOPROPYL ALCOHOL - UNII:ND2M416302)	ISOPROPYL ALCOHOL	0.70 mL in 1 mL

Inactive Ingredients

Ingredient Name	Strength
**WATER** (UNII: 059QF0KO0R)	

Packaging

#	Item Code	Package Description	Marketing Start Date	Marketing End Date
**1**	NDC:83457-927-01	1 mL in 1 PACKET; Type 0: Not a Combination Product		

Marketing Information

Marketing Category	Application Number or Monograph Citation	Marketing Start Date	Marketing End Date
OTC Monograph Drug	M003	02/14/2024	

Marketing Information

Marketing Category	Application Number or Monograph Citation	Marketing Start Date	Marketing End Date
BLA	BLA125057	02/14/2024	

HUMIRA
adalimumab kit

Product Information

**Product Type**	HUMAN PRESCRIPTION DRUG	**Item Code (Source)**	NDC:83457-243

Packaging

#	Item Code	Package Description	Marketing Start Date	Marketing End Date
1	NDC:83457-243-02	2 in 1 CARTON	02/14/2024	
1		1 in 1 KIT; Type 0: Not a Combination Product		

Quantity of Parts

Part #	Package Quantity	Total Product Quantity
**Part 1**	2 SYRINGE	0.8 mL in 2
**Part 2**	2 PACKET	2 mL in 2

Part 1 of 2

HUMIRA
adalimumab injection, solution

Product Information

**Item Code (Source)**	NDC:83457-242
**Route of Administration**	SUBCUTANEOUS

Active Ingredient/Active Moiety

Ingredient Name	Basis of Strength	Strength
**ADALIMUMAB** (UNII: FYS6T7F842) (ADALIMUMAB - UNII:FYS6T7F842)	ADALIMUMAB	40 mg in 0.4 mL

Inactive Ingredients

Ingredient Name	Strength
**MANNITOL** (UNII: 3OWL53L36A)	16.8 mg in 0.4 mL
**POLYSORBATE 80** (UNII: 6OZP39ZG8H)	0.4 mg in 0.4 mL
**WATER** (UNII: 059QF0KO0R)	

Packaging				
**#**	**Item Code**	**Package Description**	**Marketing Start Date**	**Marketing End Date**
**1**	NDC:83457-242-01	1 in 1 TRAY		
**1**		0.4 mL in 1 SYRINGE; Type 3: Prefilled Biologic Delivery Device/System (syringe, patch, etc.)		

Marketing Information

Marketing Category	Application Number or Monograph Citation	Marketing Start Date	Marketing End Date
BLA	BLA125057	02/14/2024	

Part 2 of 2

ALCOHOL

isopropyl alcohol swab

Product Information	
**Item Code (Source)**	NDC:83457-927
**Route of Administration**	TOPICAL

Active Ingredient/Active Moiety		
**Ingredient Name**	**Basis of Strength**	**Strength**
**ISOPROPYL ALCOHOL** (UNII: ND2M416302) (ISOPROPYL ALCOHOL - UNII:ND2M416302)	ISOPROPYL ALCOHOL	0.70 mL in 1 mL

Inactive Ingredients	
**Ingredient Name**	**Strength**
**WATER** (UNII: 059QF0KO0R)	

Packaging				
**#**	**Item Code**	**Package Description**	**Marketing Start Date**	**Marketing End Date**
[illegible]	NDC:83457-927 [illegible]	1 mL in 1 PACKET; Type 0: Not a Combination [illegible]		

1	NDC:83457-927-01	1 mL in 1 PACKET; Type 0: Not a Combination Product		

Marketing Information

Marketing Category	Application Number or Monograph Citation	Marketing Start Date	Marketing End Date
OTC Monograph Drug	M003	02/14/2024	

Marketing Information

Marketing Category	Application Number or Monograph Citation	Marketing Start Date	Marketing End Date
BLA	BLA125057	02/14/2024	

HUMIRA

adalimumab kit

Product Information

**Product Type**	HUMAN PRESCRIPTION DRUG	**Item Code (Source)**	NDC:83457-616

Packaging

#	Item Code	Package Description	Marketing Start Date	Marketing End Date
1	NDC:83457-616-02	2 in 1 CARTON	02/14/2024	
1		1 in 1 KIT; Type 0: Not a Combination Product		

Quantity of Parts

Part #	Package Quantity	Total Product Quantity
**Part 1**	2 SYRINGE	0.4 mL in 2
**Part 2**	2 PACKET	2 mL in 2

Part 1 of 2

HUMIRA

adalimumab injection, solution

Product Information

**Item Code (Source)**	NDC:83457-615
**Route of Administration**	SUBCUTANEOUS

Active Ingredient/Active Moiety		
**Ingredient Name**	**Basis of Strength**	**Strength**
**ADALIMUMAB** (UNII: FYS6T7F842) (ADALIMUMAB - UNII:FYS6T7F842)	ADALIMUMAB	20 mg in 0.2 mL

Inactive Ingredients	
**Ingredient Name**	**Strength**
**MANNITOL** (UNII: 3OWL53L36A)	4.8 mg in 0.2 mL
**POLYSORBATE 80** (UNII: 6OZP39ZG8H)	0.2 mg in 0.2 mL
**WATER** (UNII: 059QF0KO0R)	

Packaging				
**#**	**Item Code**	**Package Description**	**Marketing Start Date**	**Marketing End Date**
**1**	NDC:83457-615-01	1 in 1 TRAY		
**1**		0.2 mL in 1 SYRINGE; Type 3: Prefilled Biologic Delivery Device/System (syringe, patch, etc.)		

Marketing Information

Marketing Category	Application Number or Monograph Citation	Marketing Start Date	Marketing End Date
BLA	BLA125057	02/14/2024	

Part 2 of 2

ALCOHOL

isopropyl alcohol swab

Product Information	
**Item Code (Source)**	NDC:83457-927
**Route of Administration**	TOPICAL

Active Ingredient/Active Moiety		
**Ingredient Name**	**Basis of Strength**	**Strength**
**ISOPROPYL ALCOHOL** (UNII: ND2M416302) (ISOPROPYL ALCOHOL - UNII:ND2M416302)	ISOPROPYL ALCOHOL	0.70 mL in 1 mL

Inactive Ingredients

Ingredient Name	Strength
**WATER** (UNII: 059QF0KO0R)	

Packaging

#	Item Code	Package Description	Marketing Start Date	Marketing End Date
1	NDC:83457-927-01	1 mL in 1 PACKET; Type 0: Not a Combination Product		

Marketing Information

Marketing Category	Application Number or Monograph Citation	Marketing Start Date	Marketing End Date
OTC Monograph Drug	M003	02/14/2024	

Marketing Information

Marketing Category	Application Number or Monograph Citation	Marketing Start Date	Marketing End Date
BLA	BLA125057	02/14/2024	

HUMIRA

adalimumab kit

Product Information

**Product Type**	HUMAN PRESCRIPTION DRUG	**Item Code (Source)**	NDC:83457-817

Packaging

#	Item Code	Package Description	Marketing Start Date	Marketing End Date
1	NDC:83457-817-02	2 in 1 CARTON	02/14/2024	
1		1 in 1 KIT; Type 0: Not a Combination Product		

Quantity of Parts

Part #	Package Quantity	Total Product Quantity
**Part 1**	2 SYRINGE	0.2 mL in 2
**Part 2**	2 PACKET	2 mL in 2

Part 1 of 2

HUMIRA

adalimumab injection, solution

Product Information	
**Item Code (Source)**	NDC:83457-816
**Route of Administration**	SUBCUTANEOUS

Active Ingredient/Active Moiety		
**Ingredient Name**	**Basis of Strength**	**Strength**
**ADALIMUMAB** (UNII: FYS6T7F842) (ADALIMUMAB - UNII:FYS6T7F842)	ADALIMUMAB	10 mg in 0.1 mL

Inactive Ingredients	
**Ingredient Name**	**Strength**
**MANNITOL** (UNII: 3OWL53L36A)	4.2 mg in 0.1 mL
**POLYSORBATE 80** (UNII: 6OZP39ZG8H)	0.1 mg in 0.1 mL
**WATER** (UNII: 059QF0KO0R)	

Packaging				
**#**	**Item Code**	**Package Description**	**Marketing Start Date**	**Marketing End Date**
**1**	NDC:83457-816-01	1 in 1 TRAY		
**1**		0.1 mL in 1 SYRINGE; Type 3: Prefilled Biologic Delivery Device/System (syringe, patch, etc.)		

Marketing Information			
**Marketing Category**	**Application Number or Monograph Citation**	**Marketing Start Date**	**Marketing End Date**
BLA	BLA125057	02/14/2024	

Part 2 of 2

ALCOHOL

isopropyl alcohol swab

Product Information	
**Item Code (Source)**	NDC:83457-927
**Route of Administration**	TOPICAL

Active Ingredient/Active Moiety		
**Ingredient Name**	**Basis of Strength**	**Strength**
**ISOPROPYL ALCOHOL** (UNII: ND2M416302) (ISOPROPYL ALCOHOL - UNII:ND2M416302)	ISOPROPYL ALCOHOL	0.70 mL in 1 mL

Inactive Ingredients	
**Ingredient Name**	**Strength**
**WATER** (UNII: 059QF0KO0R)	

Packaging				
**#**	**Item Code**	**Package Description**	**Marketing Start Date**	**Marketing End Date**
**1**	NDC:83457-927-01	1 mL in 1 PACKET; Type 0: Not a Combination Product		

Marketing Information

Marketing Category	Application Number or Monograph Citation	Marketing Start Date	Marketing End Date
OTC Monograph Drug	M003	02/14/2024	

Marketing Information

Marketing Category	Application Number or Monograph Citation	Marketing Start Date	Marketing End Date
BLA	BLA125057	02/14/2024	

Labeler - Cordavis Limited (986134209)

LANTUS

LANTUS- insulin glargine injection, solution
LANTUS SOLOSTAR- insulin glargine injection, solution
sanofi-aventis U.S. LLC

HIGHLIGHTS OF PRESCRIBING INFORMATION
These highlights do not include all the information needed to use LANTUS safely and effectively. See full prescribing information for LANTUS.

LANTUS® (insulin glargine) injection, for subcutaneous use
Initial U.S. Approval: 2000

INDICATIONS AND USAGE

LANTUS is a long-acting human insulin analog indicated to improve glycemic control in adult and pediatric patients with diabetes mellitus. (1)

Limitations of Use

Not recommended for the treatment of diabetic ketoacidosis. (1)

DOSAGE AND ADMINISTRATION

- Individualize dosage based on metabolic needs, blood glucose monitoring, glycemic control, type of diabetes, and prior insulin use. (2.2)
- Administer subcutaneously into the abdominal area, thigh, or deltoid once daily at any time of day, but at the same time every day. (2.1)
- Do not dilute or mix with any other insulin or solution. (2.1)
- Rotate injection sites to reduce risk of lipodystrophy and localized cutaneous amyloidosis. (2.1)
- See Full Prescribing Information for the recommended starting dosage in patients with type 2 diabetes (2.3) and how to change to LANTUS from other insulins (2.4)
- Closely monitor glucose when switching to LANTUS and during initial weeks thereafter. (2.4)

DOSAGE FORMS AND STRENGTHS

Injection: 100 units/mL (U-100) available as:

- 10 mL multiple-dose vial (3)
- 3 mL single-patient-use SoloStar prefilled pen (3)

CONTRAINDICATIONS

- During episodes of hypoglycemia (4)
- Hypersensitivity to insulin glargine or any of the excipients in LANTUS (4)

WARNINGS AND PRECAUTIONS

- *Never share* a LANTUS SoloStar prefilled pen, insulin syringe, or needle between patients, even if the needle is changed. (5.1)
- *Hyperglycemia or hypoglycemia with changes in insulin regimen*: Make changes to a patient's insulin regimen (e.g., insulin strength, manufacturer, type, injection site or method of administration) under close medical supervision with increased frequency of blood glucose monitoring. (5.2)
- *Hypoglycemia*: May be life-threatening. Increase frequency of glucose monitoring with changes to: insulin dosage, concomitant drugs, meal pattern, physical activity; and in patients with renal or hepatic impairment and hypoglycemia unawareness. (5.3)
- *Hypoglycemia due to medication errors*: Accidental mix-ups between insulin products can occur. Instruct patients to check insulin labels before injection. (5.4)
- *Hypersensitivity reactions*: Severe, life-threatening, generalized allergy, including anaphylaxis, can occur. Discontinue LANTUS. Monitor and treat if indicated. (5.5)
- *Hypokalemia*: May be life-threatening. Monitor potassium levels in patients at risk of hypokalemia and treat if indicated. (5.6)
- *Fluid retention and heart failure with concomitant use of thiazolidinediones (TZDs)*: Observe for signs and symptoms of heart failure; consider dosage reduction or discontinuation of TZD if heart failure occurs. (5.7)

ADVERSE REACTIONS

Adverse reactions commonly associated with LANTUS include hypoglycemia, allergic reactions, injection site reactions, lipodystrophy, pruritus, rash, edema, and weight gain. (6.1)

To report SUSPECTED ADVERSE REACTIONS, contact sanofi-aventis at 1-800-633-1610 or FDA at 1-800-FDA-1088 or www.fda.gov/medwatch.

DRUG INTERACTIONS

- *Drugs that Affect Glucose Metabolism*: Adjustment of insulin dosage may be needed. (7)
- *Antiadrenergic Drugs* (*e.g., beta-blockers, clonidine, guanethidine, and reserpine*): Signs and symptoms of hypoglycemia may be reduced or absent. (7)

See 17 for PATIENT COUNSELING INFORMATION, FDA-approved patient labeling and FDA-approved patient labeling.

Revised: 6/2023

FULL PRESCRIBING INFORMATION: CONTENTS*

1 INDICATIONS AND USAGE
2 DOSAGE AND ADMINISTRATION
2.1 Important Administration Instructions
2.2 General Dosing Instructions
2.3 Initiation of LANTUS Therapy
2.4 Switching to LANTUS from Other Insulin Therapies
3 DOSAGE FORMS AND STRENGTHS
4 CONTRAINDICATIONS
5 WARNINGS AND PRECAUTIONS
5.1 Never Share a LANTUS SoloStar Prefilled Pen, Insulin Syringe, or Needle Between Patients
5.2 Hyperglycemia or Hypoglycemia with Changes in Insulin Regimen
5.3 Hypoglycemia
5.4 Hypoglycemia Due to Medication Errors
5.5 Hypersensitivity Reactions
5.6 Hypokalemia
5.7 Fluid Retention and Heart Failure with Concomitant Use of PPAR-gamma Agonists
6 ADVERSE REACTIONS
6.1 Clinical Trials Experience
6.2 Immunogenicity
6.3 Postmarketing Experience
7 DRUG INTERACTIONS
8 USE IN SPECIFIC POPULATIONS
8.1 Pregnancy
8.2 Lactation
8.4 Pediatric Use
8.5 Geriatric Use
8.6 Renal Impairment
8.7 Hepatic Impairment
10 OVERDOSAGE
11 DESCRIPTION
12 CLINICAL PHARMACOLOGY
12.1 Mechanism of Action
12.2 Pharmacodynamics
12.3 Pharmacokinetics

13 NONCLINICAL TOXICOLOGY
13.1 Carcinogenesis, Mutagenesis, Impairment of Fertility
14 CLINICAL STUDIES
14.1 Overview of Clinical Studies
14.2 Clinical Studies in Adult and Pediatric Patients with Type 1 Diabetes
14.3 Clinical Studies in Adults with Type 2 Diabetes
14.4 Additional Clinical Studies in Adults with Diabetes Type 1 and Type 2
16 HOW SUPPLIED/STORAGE AND HANDLING
16.1 How Supplied
16.2 Storage
17 PATIENT COUNSELING INFORMATION
* Sections or subsections omitted from the full prescribing information are not listed.

FULL PRESCRIBING INFORMATION

1 INDICATIONS AND USAGE

LANTUS is indicated to improve glycemic control in adult and pediatric patients with diabetes mellitus.

Limitations of Use

LANTUS is not recommended for the treatment of diabetic ketoacidosis.

2 DOSAGE AND ADMINISTRATION

2.1 Important Administration Instructions

- Always check insulin labels before administration *[see Warnings and Precautions (5.4)].*
- Visually inspect LANTUS vials and SoloStar prefilled pens for particulate matter and discoloration prior to administration. Only use if the solution is clear and colorless with no visible particles.
- Administer LANTUS subcutaneously into the abdominal area, thigh, or deltoid, and rotate injection sites within the same region from one injection to the next to reduce the risk of lipodystrophy and localized cutaneous amyloidosis. Do not inject into areas of lipodystrophy or localized cutaneous amyloidosis *[see Warnings and Precautions (5.2) and Adverse Reactions (6)]*.
- During changes to a patient's insulin regimen, increase the frequency of blood glucose monitoring *[see Warnings and Precautions (5.2)]*.
- Do not administer intravenously or via an insulin pump.
- Do not dilute or mix LANTUS with any other insulin or solution.
- The LANTUS SoloStar prefilled pen dials in 1-unit increments.
- Use LANTUS SoloStar prefilled pen with caution in patients with visual impairment who may rely on audible clicks to dial their dose.

2.2 General Dosing Instructions

- Administer LANTUS subcutaneously once daily at any time of day but at the same time every day.
- Individualize and adjust the dosage of LANTUS based on the patient's metabolic needs, blood glucose monitoring results and glycemic control goal.
- Dosage adjustments may be needed with changes in physical activity, changes in meal patterns (i.e., macronutrient content or timing of food intake), during acute illness, or changes in renal or hepatic function. Dosage adjustments should only be made under medical supervision with appropriate glucose monitoring *[see Warnings and Precautions (5.2)]*.
- In patients with type 1 diabetes, LANTUS must be used concomitantly with short-acting insulin.

2.3 Initiation of LANTUS Therapy

<u>Recommended Starting Dosage in Patients with Type 1 Diabetes</u>

The recommended starting dosage of LANTUS in patients with type 1 diabetes is approximately one-third of the total daily insulin requirements. Use short-acting, premeal insulin to satisfy the remainder of the daily insulin requirements.

<u>Recommended Starting Dosage in Patients with Type 2 Diabetes</u>

The recommended starting dosage of LANTUS in patients with type 2 diabetes who are not currently treated with insulin is 0.2 units/kg or up to 10 units once daily.

2.4 Switching to LANTUS from Other Insulin Therapies

Dosage adjustments are recommended to lower the risk of hypoglycemia when switching patients to LANTUS from other insulin therapies *[see Warnings and Precautions (5.3)].*

When switching from:
- Once-daily TOUJEO (insulin glargine 300 units/mL) to once-daily LANTUS (100 units/mL), the recommended starting LANTUS dosage is 80% of the TOUJEO dosage that is being discontinued.
- Once-daily NPH insulin to once-daily LANTUS, the recommended starting LANTUS dosage is the same as the dosage of NPH that is being discontinued.
- Twice-daily NPH insulin to once-daily LANTUS, the recommended starting LANTUS dosage is 80% of the total NPH dosage that is being discontinued.

3 DOSAGE FORMS AND STRENGTHS

Injection: 100 units/mL (U-100) a clear and colorless solution available as:
- 10 mL multiple-dose vial
- 3 mL single-patient-use LANTUS SoloStar prefilled pen

4 CONTRAINDICATIONS

LANTUS is contraindicated:
- During episodes of hypoglycemia *[see Warnings and Precautions (5.3)]*
- In patients with hypersensitivity to insulin glargine or any of the excipients in LANTUS *[see Warnings and Precautions (5.5)]*

5 WARNINGS AND PRECAUTIONS

5.1 Never Share a LANTUS SoloStar Prefilled Pen, Insulin Syringe, or Needle Between Patients

LANTUS SoloStar prefilled pens must never be shared between patients, even if the needle is changed. Patients using LANTUS vials must never re-use or share needles or syringes with another person. Sharing poses a risk for transmission of blood-borne pathogens.

5.2 Hyperglycemia or Hypoglycemia with Changes in Insulin Regimen

Changes in an insulin regimen (e.g., insulin strength, manufacturer, type, injection site or method of administration) may affect glycemic control and predispose to hypoglycemia *[see Warnings and Precautions (5.3)]* or hyperglycemia. Repeated insulin injections into areas of lipodystrophy or localized cutaneous amyloidosis have been reported to result in hyperglycemia; and a sudden change in the injection site (to unaffected area) has been reported to result in hypoglycemia *[see Adverse Reactions (6)]*.

Make any changes to a patient's insulin regimen under close medical supervision with increased frequency of blood glucose monitoring. Advise patients who have repeatedly injected into areas of lipodystrophy or localized cutaneous amyloidosis to change the injection site to unaffected areas and closely monitor for hypoglycemia. For patients with type 2 diabetes, dosage adjustments of concomitant oral and antidiabetic products may be needed.

5.3 Hypoglycemia

Hypoglycemia is the most common adverse reaction associated with insulins, including LANTUS. Severe hypoglycemia can cause seizures, may be life-threatening or cause death. Hypoglycemia can impair concentration ability and reaction time; this may place the patient and others at risk in situations where these abilities are important (e.g., driving or operating other machinery).

Hypoglycemia can happen suddenly, and symptoms may differ in each patient and change over time in the same patient. Symptomatic awareness of hypoglycemia may be less pronounced in patients with longstanding diabetes, in patients with diabetic neuropathy, using drugs that block the sympathetic nervous system (e.g., beta-blockers) *[see Drug Interactions (7)]*, or who experience recurrent hypoglycemia.

The long-acting effect of LANTUS may delay recovery from hypoglycemia.

<u>Risk Factors for Hypoglycemia</u>

The risk of hypoglycemia after an injection is related to the duration of action of the insulin and, in general, is highest when the glucose lowering effect of the insulin is maximal. As with all insulins, the glucose lowering effect time course of LANTUS may vary in different patients or at different times in the same patient and depends on many conditions, including the area of injection as well as the injection site blood supply and temperature *[see Clinical Pharmacology (12.2)]*. Other factors which may increase the risk of hypoglycemia include changes in meal pattern (e.g., macronutrient content or timing of meals), changes in level of physical activity, or changes to concomitant drugs

[see Drug Interactions (7)]. Patients with renal or hepatic impairment may be at higher risk of hypoglycemia *[see Use in Specific Populations (8.6, 8.7)]*.

Risk Mitigation Strategies for Hypoglycemia

Patients and caregivers must be educated to recognize and manage hypoglycemia. Self-monitoring of blood glucose plays an essential role in the prevention and management of hypoglycemia. In patients at higher risk for hypoglycemia and patients who have reduced symptomatic awareness of hypoglycemia, increased frequency of blood glucose monitoring is recommended.

5.4 Hypoglycemia Due to Medication Errors

Accidental mix-ups among insulin products have been reported. To avoid medication errors between LANTUS and other insulins, instruct patients to always check the insulin label before each injection *[see Adverse Reactions (6.3)]*.

5.5 Hypersensitivity Reactions

Severe, life-threatening, generalized allergy, including anaphylaxis, can occur with insulins, including LANTUS *[see Adverse Reactions (6.1)]*. If hypersensitivity reactions occur, discontinue LANTUS; treat per standard of care and monitor until symptoms and signs resolve. LANTUS is contraindicated in patients who have had hypersensitivity reactions to insulin glargine or one of the excipients.

5.6 Hypokalemia

All insulins, including LANTUS, cause a shift in potassium from the extracellular to intracellular space, possibly leading to hypokalemia. Untreated hypokalemia may cause respiratory paralysis, ventricular arrhythmia, and death. Monitor potassium levels in patients at risk for hypokalemia, if indicated (e.g., patients using potassium-lowering medications, patients taking medications sensitive to serum potassium concentrations).

5.7 Fluid Retention and Heart Failure with Concomitant Use of PPAR-gamma Agonists

Thiazolidinediones (TZDs), which are peroxisome proliferator-activated receptor (PPAR)-gamma agonists, can cause dose-related fluid retention, when used in combination with insulin. Fluid retention may lead to or exacerbate heart failure. Patients treated with insulin, including LANTUS, and a PPAR-gamma agonist should be observed for signs and symptoms of heart failure. If heart failure develops, it should be managed according to current standards of care, and discontinuation or dose reduction of the PPAR-gamma agonist must be considered.

6 ADVERSE REACTIONS

The following adverse reactions are discussed elsewhere:

- Hyperglycemia or Hypoglycemia with Changes in Insulin Regimen *[see Warnings and Precautions (5.2)]*
- Hypoglycemia *[see Warnings and Precautions (5.3)]*
- Hypoglycemia Due to Medication Errors *[see Warnings and Precautions (5.4)]*
- Hypersensitivity Reactions *[see Warnings and Precautions (5.5)]*

- Hypokalemia *[see Warnings and Precautions (5.6)]*

6.1 Clinical Trials Experience

Because clinical trials are conducted under widely varying conditions, adverse reaction rates observed in clinical trials of a drug cannot be directly compared to rates in the clinical trial of another drug and may not reflect the rates observed in practice.

The data in Table 1 reflect the exposure of 2,327 patients with type 1 diabetes to LANTUS or NPH in Studies A, B, C, and D *[see Clinical Studies (14.2)]*. The type 1 diabetes population had the following characteristics: the mean age was 39 years, 54% were male, and mean body mass index (BMI) was 25.1 kg/m^2. Ninety-seven percent were White, 2% were Black or African American and less than 1% were Asian. Approximately 3% of the patients in studies B and C were Hispanic.

The data in Table 2 reflect the exposure of 1,563 patients with type 2 diabetes to LANTUS or NPH in Studies E, F, and G *[see Clinical Studies (14.3)]*. The type 2 diabetes population had the following characteristics: the mean age was 59 years, 58% were male, and mean BMI was 29.2 kg/m^2. Eighty-seven percent were White, 8% were Black or African American and 3% were Asian. Approximately 9% of patients in Study F were Hispanic.

The frequencies of adverse reactions during LANTUS clinical studies in patients with type 1 diabetes mellitus and type 2 diabetes mellitus are listed in the tables below (Tables 1, 2, 3, and 4).

Table 1: Adverse Reactions Occurring ≥5% in Pooled Clinical Studies up to 28 Weeks Duration in Adults with Type 1 Diabetes

	LANTUS, % (n=1,257)	NPH, % (n=1,070)
Upper respiratory tract infection	22.4	23.1
Infection*	9.4	10.3
Accidental injury	5.7	6.4
Headache	5.5	4.7

* Body system not specified

Table 2: Adverse Reactions Occurring ≥5% in Pooled Clinical Studies up to 1 Year Duration in Adults with Type 2 Diabetes

	LANTUS, % (n=849)	NPH, % (n=714)
Upper respiratory tract infection	11.4	13.3
Infection*	10.4	11.6
Retinal vascular disorder	5.8	7.4

* Body system not specified

Table 3: Adverse Reactions Occurring ≥10% in a 5-Year Study of Adults with Type 2 Diabetes

	LANTUS, % (n=514)	NPH, % (n=503)
Upper respiratory tract infection	29.0	33.6
Edema peripheral	20.0	22.7
Hypertension	19.6	18.9
Influenza	18.7	19.5
Sinusitis	18.5	17.9
Cataract	18.1	15.9
Bronchitis	15.2	14.1
Arthralgia	14.2	16.1
Pain in extremity	13.0	13.1
Back pain	12.8	12.3
Cough	12.1	7.4
Urinary tract infection	10.7	10.1
Diarrhea	10.7	10.3
Depression	10.5	9.7
Headache	10.3	9.3

Table 4: Adverse Reactions Occurring ≥5% in a 28-Week Clinical Study in Pediatric Patients with Type 1 Diabetes

	LANTUS, % (n=174)	NPH, % (n=175)
Infection*	13.8	17.7
Upper respiratory tract infection	13.8	16.0
Pharyngitis	7.5	8.6
Rhinitis	5.2	5.1

* Body system not specified

Severe Hypoglycemia

Hypoglycemia was the most commonly observed adverse reaction in patients treated with LANTUS. Tables 5, 6, and 7 summarize the incidence of severe hypoglycemia in the LANTUS clinical studies. Severe symptomatic hypoglycemia was defined as an event with symptoms consistent with hypoglycemia requiring the assistance of another person and associated with either a blood glucose below 50 mg/dL (≤56 mg/dL in the 5-year study and ≤36 mg/dL in the ORIGIN study) or prompt recovery after oral carbohydrate, intravenous glucose, or glucagon administration.

Percentages of LANTUS-treated adult patients who experienced severe symptomatic hypoglycemia in the LANTUS clinical studies *[see Clinical Studies (14)]* were comparable to percentages of NPH-treated patients for all treatment regimens (see Tables 5 and 6). In the pediatric clinical study, pediatric patients with type 1 diabetes had a higher incidence of severe symptomatic hypoglycemia in the two treatment groups compared

to the adult studies with type 1 diabetes.

Table 5: Severe Symptomatic Hypoglycemia in Patients with Type 1 Diabetes

	Study A Type 1 Diabetes Adults 28 weeks In combination with regular insulin		Study B Type 1 Diabetes Adults 28 weeks In combination with regular insulin		Study C Type 1 Diabetes Adults 16 weeks In combination with insulin lispro		Study D Type 1 Diabetes Pediatrics 26 weeks In combination with regular insulin	
	**LANTUS N=292**	**NPH N=293**	**LANTUS N=264**	**NPH N=270**	**LANTUS N=310**	**NPH N=309**	**LANTUS N=174**	**NPH N=175**
Percent of patients	10.6	15.0	8.7	10.4	6.5	5.2	23.0	28.6

Table 6: Severe Symptomatic Hypoglycemia in Patients with Type 2 Diabetes

	Study E Type 2 Diabetes Adults 52 weeks In combination with oral agents		Study F Type 2 Diabetes Adults 28 weeks In combination with regular insulin		Study G Type 2 Diabetes Adults 5 years In combination with regular insulin	
	**LANTUS N=289**	**NPH N=281**	**LANTUS N=259**	**NPH N=259**	**LANTUS N=513**	**NPH N=504**
Percent of patients	1.7	1.1	0.4	2.3	7.8	11.9

Table 7 displays the proportion of patients who experienced severe symptomatic hypoglycemia in the LANTUS and Standard Care groups in the ORIGIN study *[see Clinical Studies (14)]*.

Table 7: Severe Symptomatic Hypoglycemia in the ORIGIN Study

	ORIGIN Study Median duration of follow-up: 6.2 years	
	**LANTUS N=6231**	**Standard Care N=6273**
Percent of patients	5.6	1.8

Peripheral Edema

Some patients taking LANTUS have experienced sodium retention and edema, particularly if previously poor metabolic control was improved by intensified insulin therapy.

Lipodystrophy

Administration of insulin subcutaneously, including LANTUS, has resulted in lipoatrophy (depression in the skin) or lipohypertrophy (enlargement or thickening of tissue) in some patients *[see Dosage and Administration (2.2)]*.

Insulin Initiation and Intensification of Glucose Control

Intensification or rapid improvement in glucose control has been associated with a transitory, reversible ophthalmologic refraction disorder, worsening of diabetic retinopathy, and acute painful peripheral neuropathy. However, long-term glycemic control decreases the risk of diabetic retinopathy and neuropathy.

Weight Gain

Weight gain has occurred with insulin including LANTUS and has been attributed to the anabolic effects of insulin and the decrease in glucosuria.

Hypersensitivity Reactions

Local Reactions

Patients taking LANTUS experienced injection site reactions, including redness, pain, itching, urticaria, edema, and inflammation. In clinical studies in adult patients, there was a higher incidence of injection site pain in LANTUS-treated patients (2.7%) compared to NPH insulin-treated patients (0.7%). The reports of pain at the injection site did not result in discontinuation of therapy.

Systemic Reactions

Severe, life-threatening, generalized allergy, including anaphylaxis, generalized skin reactions, angioedema, bronchospasm, hypotension, and shock have occurred with insulin, including LANTUS and may be life threatening.

6.2 Immunogenicity

As with all therapeutic proteins, there is potential for immunogenicity. All insulin products can elicit the formation of insulin antibodies. The presence of such insulin antibodies may increase or decrease the efficacy of insulin and may require adjustment of the insulin dose. In clinical studies of LANTUS, increases in titers of antibodies to insulin were observed in NPH insulin and LANTUS treatment groups with similar incidences.

6.3 Postmarketing Experience

The following adverse reactions have been identified during postapproval use of LANTUS. Because these reactions are reported voluntarily from a population of uncertain size, it is not always possible to reliably estimate their frequency or establish a causal relationship to drug exposure.

Medication errors have been reported in which rapid-acting insulins and other insulins,

have been accidentally administered instead of LANTUS.

Localized cutaneous amyloidosis at the injection site has occurred. Hyperglycemia has been reported with repeated insulin injections into areas of localized cutaneous amyloidosis; hypoglycemia has been reported with a sudden change to an unaffected injection site.

7 DRUG INTERACTIONS

Table 8 includes clinically significant drug interactions with LANTUS.

Table 8: Clinically Significant Drug Interactions with LANTUS

**Drugs that May Increase the Risk of Hypoglycemia**	
Drugs:	Antidiabetic agents, ACE inhibitors, angiotensin II receptor blocking agents, disopyramide, fibrates, fluoxetine, monoamine oxidase inhibitors, pentoxifylline, pramlintide, salicylates, somatostatin analogs (e.g., octreotide), sulfonamide antibiotics. GLP-1 receptor agonists, DPP-4 inhibitors, and SGLT-2 inhibitors.
Intervention:	Dosage reductions and increased frequency of glucose monitoring may be required when LANTUS is coadministered with these drugs.
**Drugs that May Decrease the Blood Glucose Lowering Effect of LANTUS**	
Drugs:	Atypical antipsychotics (e.g., olanzapine and clozapine), corticosteroids, danazol, diuretics, estrogens, glucagon, isoniazid, niacin, oral contraceptives, phenothiazines, progestogens (e.g., in oral contraceptives), protease inhibitors, somatropin, sympathomimetic agents (e.g., albuterol, epinephrine, terbutaline), and thyroid hormones.
Intervention:	Dosage increases and increased frequency of glucose monitoring may be required when LANTUS is coadministered with these drugs.
**Drugs that May Increase or Decrease the Blood Glucose Lowering Effect of LANTUS**	
Drugs:	Alcohol, beta-blockers, clonidine, and lithium salts. Pentamidine may cause hypoglycemia, which may sometimes be followed by hyperglycemia.
Intervention:	Dosage adjustment and increased frequency of glucose monitoring may be required when LANTUS is coadministered with these drugs.
**Drugs that May Blunt Signs and Symptoms of Hypoglycemia**	
Drugs:	Beta-blockers, clonidine, guanethidine, and reserpine.
Intervention:	Increased frequency of glucose monitoring may be required when LANTUS is coadministered with these drugs.

8 USE IN SPECIFIC POPULATIONS

8.1 Pregnancy

Risk Summary

Published studies with use of insulin glargine during pregnancy have not reported a clear association with insulin glargine and adverse developmental outcomes *(see Data)*. There are risks to the mother and fetus associated with poorly controlled diabetes in pregnancy *(see Clinical Considerations)*.

Rats and rabbits were exposed to insulin glargine in animal reproduction studies during organogenesis, respectively 50 times and 10 times the human subcutaneous dosage of 0.2 units/kg/day. Overall, the effects of insulin glargine did not generally differ from those observed with regular human insulin *(see Data)*.

In the U.S. general population, the estimated background risk of major birth defects and miscarriage in clinically recognized pregnancies is 2% to 4% and 15% to 20%, respectively. The estimated background risk of major birth defects is 6% to 10% in women with pregestational diabetes with a peri-conceptional HbA1c >7 and has been reported to be as high as 20% to 25% in women with a peri-conceptional HbA1c >10. The estimated background risk of miscarriage for the indicated population is unknown.

Clinical Considerations

Disease-Associated Maternal and/or Embryo-fetal Risk

Hypoglycemia and hyperglycemia occur more frequently during pregnancy in patients with pre-gestational diabetes. Poorly controlled diabetes in pregnancy increases the maternal risk for diabetic ketoacidosis, preeclampsia, spontaneous abortions, preterm delivery, and delivery complications. Poorly controlled diabetes increases the fetal risk for major birth defects, stillbirth, and macrosomia-related morbidity.

Data

Human Data

Published data do not report a clear association with insulin glargine and major birth defects, miscarriage, or adverse maternal or fetal outcomes when insulin glargine is used during pregnancy. However, these studies cannot definitely establish the absence of any risk because of methodological limitations including small sample size and some lacking comparator groups.

Animal Data

Subcutaneous reproduction and teratology studies have been performed with insulin glargine and regular human insulin in rats and Himalayan rabbits. Insulin glargine was given to female rats before mating, during mating, and throughout pregnancy at doses up to 0.36 mg/kg/day, which is approximately 50 times the recommended human subcutaneous starting dosage of 0.2 units/kg/day (0.007 mg/kg/day), on a mg/kg basis. In rabbits, doses of 0.072 mg/kg/day, which is approximately 10 times the recommended human subcutaneous starting dosage of 0.2 units/kg/day on a mg/kg basis, were administered during organogenesis. The effects of insulin glargine did not generally differ from those observed with regular human insulin in rats or rabbits. However, in rabbits, five fetuses from two litters of the high-dose group exhibited dilation of the cerebral ventricles. Fertility and early embryonic development appeared normal.

8.2 Lactation

Risk Summary

There are either no or only limited data on the presence of insulin glargine in human milk, the effects on breastfed infant, or the effects on milk production. Endogenous insulin is present in human milk. The developmental and health benefits of breastfeeding should be considered along with the mother's clinical need for LANTUS, and any potential adverse effects on the breastfed child from LANTUS or from the underlying maternal condition.

8.4 Pediatric Use

The safety and effectiveness of LANTUS to improve glycemic control in pediatric patients with diabetes mellitus have been established. Use of LANTUS for this indication is supported by evidence from an adequate and well-controlled study (Study D) in 174 LANTUS-treated pediatric patients aged 6 to 15 years with type 1 diabetes mellitus, and from adequate and well-controlled studies of LANTUS in adults with diabetes mellitus *[see Clinical Pharmacology (12.3), Clinical Studies (14.2)].*

In the pediatric clinical study, pediatric patients with type 1 diabetes had a higher incidence of severe symptomatic hypoglycemia compared to the adults in studies with type 1 diabetes *[see Adverse Reactions (6.1)]*.

8.5 Geriatric Use

Of the total number of subjects in controlled clinical studies of patients with type 1 and type 2 diabetes who were treated with LANTUS, 15% (n=316) were ≥65 years of age and 2% (n=42) were ≥75 years of age. No overall differences in safety or effectiveness of LANTUS have been observed between patients 65 years of age and older and younger adult patients.

Nevertheless, caution should be exercised when LANTUS is administered to geriatric patients. In geriatric patients with diabetes, the initial dosing, dosage increments, and maintenance dosage should be conservative to avoid hypoglycemic reactions. Hypoglycemia may be difficult to recognize in geriatric patients.

8.6 Renal Impairment

The effect of kidney impairment on the pharmacokinetics of LANTUS has not been studied. Some studies with human insulin have shown increased circulating levels of insulin in patients with kidney failure. Frequent glucose monitoring and dosage adjustment may be necessary for LANTUS in patients with kidney impairment *[see Warnings and Precautions (5.3)].*

8.7 Hepatic Impairment

The effect of hepatic impairment on the pharmacokinetics of LANTUS has not been studied. Frequent glucose monitoring and dosage adjustment may be necessary for LANTUS in patients with hepatic impairment *[see Warnings and Precautions (5.3)]*.

10 OVERDOSAGE

Excess insulin administration may cause hypoglycemia and hypokalemia *[see Warnings and Precautions (5.3, 5.6)]*.

Mild episodes of hypoglycemia can usually be treated with oral carbohydrates. Lowering the insulin dosage, and adjustments in meal patterns or exercise may be needed.

More severe episodes of hypoglycemia with coma, seizure, or neurologic impairment may be treated with glucagon for emergency use or concentrated intravenous glucose. After apparent clinical recovery from hypoglycemia, continued observation and additional carbohydrate intake may be necessary to avoid recurrence of hypoglycemia.

Hypokalemia must be corrected appropriately.

11 DESCRIPTION

Insulin glargine is a long-acting human insulin analog produced by recombinant DNA technology utilizing a non-pathogenic laboratory strain of *Escherichia coli* (K12). Insulin glargine differs from human insulin in that the amino acid asparagine at position A21 is replaced by glycine and two arginines are added to the C-terminus of the B-chain. Insulin glargine has a molecular weight of 6063 Da.

LANTUS (insulin glargine) injection is a sterile, clear and colorless solution for subcutaneous use in a 10 mL multiple-dose vial or a 3 mL single-patient use prefilled pen (LANTUS Solostar).

Prefilled Pen (LANTUS Solostar) and Vial: Each mL contains 100 units of insulin glargine and the inactive ingredients: glycerol 85% (20 mg), m-cresol (2.7 mg), zinc (30 mcg), and Water for Injection, USP. The vial also contains polysorbate 20 (20 mcg). The pH is adjusted by addition of aqueous solutions of hydrochloric acid and sodium hydroxide. LANTUS has a pH of approximately 4.

12 CLINICAL PHARMACOLOGY

12.1 Mechanism of Action

The primary activity of insulin, including insulin glargine, is regulation of glucose metabolism. Insulin and its analogs lower blood glucose by stimulating peripheral glucose uptake, especially by skeletal muscle and fat, and by inhibiting hepatic glucose production. Insulin inhibits lipolysis and proteolysis, and enhances protein synthesis.

12.2 Pharmacodynamics

In clinical studies, the glucose-lowering effect on a molar basis (i.e., when given at the same doses) of intravenous LANTUS is approximately the same as that for human insulin. Figure 1 shows results from a study in patients with type 1 diabetes conducted for a maximum of 24 hours after subcutaneous injection of LANTUS or NPH insulin. The median time between subcutaneous injection and the end of pharmacological effect was 14.5 hours (range: 9.5 to 19.3 hours) for NPH insulin, and 24 hours (range: 10.8 to >24 hours) (24 hours was the end of the observation period) for LANTUS.

Figure 1: Glucose-Lowering Effect Over 24 Hours in Patients with Type 1 Diabetes

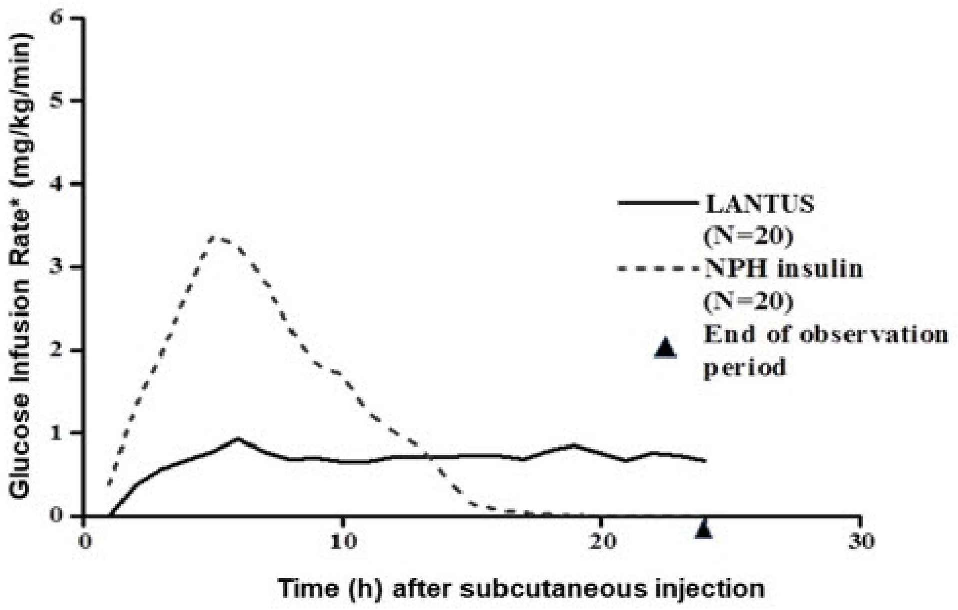

* Determined as amount of glucose infused to maintain constant plasma glucose levels

The duration of action after abdominal, deltoid, or thigh subcutaneous administration of LANTUS was similar. The time course of action of insulins, including LANTUS, may vary between patients and within the same patient.

12.3 Pharmacokinetics

Absorption

After subcutaneous injection of LANTUS in healthy subjects and in patients with diabetes, the insulin serum concentrations indicated a slower, more prolonged absorption and a relatively constant concentration/time profile over 24 hours with no pronounced peak in comparison to NPH insulin.

Elimination

Metabolism

A metabolism study in humans indicates that insulin glargine is partly metabolized at the carboxyl terminus of the B chain in the subcutaneous depot to form two active metabolites with *in vitro* activity similar to that of human insulin, M1 (21^{A}-Gly-insulin) and M2 (21^{A}-Gly-des-30^{B}-Thr-insulin). Unchanged drug and these degradation products are also present in the circulation.

Specific Populations

Age, Race, Body Mass Index, and Gender

Effect of age, race, body mass index (BMI), and gender on the pharmacokinetics of insulin glargine has not been evaluated. However, in controlled clinical studies in adults (n=3,890) and a controlled clinical study in pediatric patients (n=349), subgroup analyses based on age, race, BMI, and gender did not show differences in safety and efficacy between LANTUS and NPH insulin *[see Clinical Studies (14)]*.

13 NONCLINICAL TOXICOLOGY

13.1 Carcinogenesis, Mutagenesis, Impairment of Fertility

In mice and rats, standard two-year carcinogenicity studies with insulin glargine were performed at doses up to 0.455 mg/kg, which was for the rat approximately 65 times the recommended human subcutaneous starting dosage of 0.2 units/kg/day (0.007 mg/kg/day) on a mg/kg basis. Histiocytomas were found at injection sites in male rats and mice in acid vehicle containing groups and are considered a response to chronic tissue irritation and inflammation in rodents. These tumors were not found in female animals, in saline control, or insulin comparator groups using a different vehicle.

Insulin glargine was not mutagenic in tests for detection of gene mutations in bacteria and mammalian cells (Ames and HGPRT-test) and in tests for detection of chromosomal aberrations (cytogenetics *in vitro* in V79 cells and in vivo in Chinese hamsters).

In a combined fertility and prenatal and postnatal study in male and female rats at subcutaneous doses up to 0.36 mg/kg/day, which was approximately 50 times the recommended human subcutaneous starting dose of 0.2 units/kg/day (0.007 mg/kg/day) maternal toxicity due to dose-dependent hypoglycemia, including some deaths, was observed. Consequently, a reduction of the rearing rate occurred in the high-dose group only. Similar effects were observed with NPH insulin.

14 CLINICAL STUDIES

14.1 Overview of Clinical Studies

The safety and effectiveness of LANTUS given once-daily at bedtime was compared to that of once-daily and twice-daily NPH insulin in open-label, randomized, active-controlled, parallel studies of 2,327 adult patients and 349 pediatric patients with type 1 diabetes mellitus and 1,563 adult patients with type 2 diabetes mellitus (see Tables 9–11). In general, the reduction in glycated hemoglobin (HbA1c) with LANTUS was similar to that with NPH insulin.

14.2 Clinical Studies in Adult and Pediatric Patients with Type 1 Diabetes

Adult Patients with Type 1 Diabetes

In two clinical studies (Studies A and B), adult patients with type 1 diabetes (Study A, n=585, Study B n=534) were randomized to 28 weeks of basal-bolus treatment with LANTUS or NPH insulin. Regular human insulin was administered before each meal. LANTUS was administered at bedtime. NPH insulin was administered either as once daily at bedtime or in the morning and at bedtime when used twice daily.

In Study A, the average age was 39 years. The majority of patients were White (99%) and 56% were male. The mean BMI was approximately 24.9 kg/m^2. The mean duration of diabetes was 16 years.

In Study B, the average age was 39 years. The majority of patients were White (95%) and 51% were male. The mean BMI was approximately 25.8 kg/m^2. The mean duration of diabetes was 17 years.

In another clinical study (Study C), patients with type 1 diabetes (n=619) were randomized to 16 weeks of basal-bolus treatment with LANTUS or NPH insulin. Insulin lispro was used before each meal. LANTUS was administered once daily at bedtime and NPH insulin was administered once or twice daily. The average age was 39 years. The majority of patients were White (97%) and 51% were male. The mean BMI was approximately 25.6 kg/m^2. The mean duration of diabetes was 19 years.

In these 3 adult studies, LANTUS and NPH insulin had similar effects on HbA1c (Table 9) with a similar overall rate of severe symptomatic hypoglycemia *[see Adverse Reactions (6.1)]*.

Table 9: Type 1 Diabetes Mellitus – Adults

**Treatment duration Treatment in combination with**	**Study A 28 weeks Regular insulin**		**Study B 28 weeks Regular insulin**		**Study C 16 weeks Insulin lispro**	
	**LANTUS**	**NPH**	**LANTUS**	**NPH**	**LANTUS**	**NPH**
Number of subjects treated	292	293	264	270	310	309
**HbA1c**						
Baseline HbA1c	8.0	8.0	7.7	7.7	7.6	7.7
Adjusted mean change at study end	+0.2	+0.1	-0.2	-0.2	-0.1	-0.1
Treatment Difference (95% CI)	+0.1 (0.0; +0.2)		+0.1 (-0.1; +0.2)		0.0 (-0.1; +0.1)	
**Basal insulin dose**						
Baseline mean	21	23	29	29	28	28
Mean change from baseline	-2	0	-4	+2	-5	+1
**Total insulin dose**						
Baseline mean	48	52	50	51	50	50
Mean change from baseline	-1	0	0	+4	-3	0
**Fasting blood glucose (mg/dL)**						
Baseline mean	167	166	166	175	175	173
Adj. mean change from baseline	-21	-16	-20	-17	-29	-12
**Body weight (kg)**						
Baseline mean	73.2	74.8	75.5	75.0	74.8	75.6
Mean change from baseline	0.1	-0.0	0.7	1.0	0.1	0.5

<u>Pediatric Patients with Type 1 Diabetes</u>

In a randomized, controlled clinical study (Study D), pediatric patients (age range 6 to 15

years) with type 1 diabetes (n=349) were treated for 28 weeks with a basal-bolus insulin regimen where regular human insulin was used before each meal. LANTUS was administered once daily at bedtime and NPH insulin was administered once or twice daily. The average age was 11.7 years. The majority of patients were White (97%) and 52% were male. The mean BMI was approximately 18.9 kg/m^2. The mean duration of diabetes was 5 years. Similar effects on HbA1c (Table 10) were observed in both treatment groups *[see Adverse Reactions (6.1)]*.

Table 10: Type 1 Diabetes Mellitus – Pediatric Patients

**Treatment duration**<br>**Treatment in combination with**	**Study D**<br>**28 weeks**<br>**Regular insulin**	
	**LANTUS + Regular insulin**	**NPH + Regular insulin**
Number of subjects treated	174	175
**HbA1c**		
Baseline mean	8.5	8.8
Change from baseline (adjusted mean)	+0.3	+0.3
Difference from NPH (adjusted mean)	0.0	
(95% CI)	(-0.2; +0.3)	
**Basal insulin dose**		
Baseline mean	19	19
Mean change from baseline	-1	+2
**Total insulin dose**		
Baseline mean	43	43
Mean change from baseline	+2	+3
**Fasting blood glucose (mg/dL)**		
Baseline mean	194	191
Mean change from baseline	-23	-12
**Body weight (kg)**		
Baseline mean	45.5	44.6
Mean change from baseline	2.2	2.5

14.3 Clinical Studies in Adults with Type 2 Diabetes

In a randomized, controlled clinical study (Study E) in 570 adults with type 2 diabetes, LANTUS was evaluated for 52 weeks in combination with oral antidiabetic medications (a sulfonylurea, metformin, acarbose, or combinations of these drugs). The average age was 60 years old. The majority of patients were White (93%) and 54% were male. The mean BMI was approximately 29.1 kg/m^2. The mean duration of diabetes was 10 years. LANTUS administered once daily at bedtime was as effective as NPH insulin administered once daily at bedtime in reducing HbA1c and fasting glucose (Table 11). The rate of severe symptomatic hypoglycemia was similar in LANTUS and NPH insulin treated patients *[see Adverse Reactions (6.1)]*.

In a randomized, controlled clinical study (Study F), in adult patients with type 2 diabetes

not using oral antidiabetic medications (n=518), a basal-bolus regimen of LANTUS once daily at bedtime or NPH insulin administered once or twice daily was evaluated for 28 weeks. Regular human insulin was used before meals, as needed. The average age was 59 years. The majority of patients were White (81%) and 60% were male. The mean BMI was approximately 30.5 kg/m^2. The mean duration of diabetes was 14 years. LANTUS had similar effectiveness as either once- or twice-daily NPH insulin in reducing HbA1c and fasting glucose (Table 11) with a similar incidence of hypoglycemia *[see Adverse Reactions (6.1)]*.

In a randomized, controlled clinical study (Study G), adult patients with type 2 diabetes were randomized to 5 years of treatment with once-daily LANTUS or twice-daily NPH insulin. For patients not previously treated with insulin, the starting dosage of LANTUS or NPH insulin was 10 units daily. Patients who were already treated with NPH insulin either continued on the same total daily NPH insulin dose or started LANTUS at a dosage that was 80% of the total previous NPH insulin dosage. The primary endpoint for this study was a comparison of the progression of diabetic retinopathy by 3 or more steps on the Early Treatment Diabetic Retinopathy Study (ETDRS) scale. HbA1c change from baseline was a secondary endpoint. Similar glycemic control in the 2 treatment groups was desired in order to not confound the interpretation of the retinal data. Patients or study personnel used an algorithm to adjust the LANTUS and NPH insulin dosages to a target fasting plasma glucose ≤100 mg/dL. After the LANTUS or NPH insulin dosage was adjusted, other antidiabetic agents, including premeal insulin were to be adjusted or added. The average age was 55 years. The majority of patients were White (85%) and 54% were male. The mean BMI was approximately 34.3 kg/m^2. The mean duration of diabetes was 11 years. The LANTUS group had a smaller mean reduction from baseline in HbA1c compared to the NPH insulin group, which may be explained by the lower daily basal insulin doses in the LANTUS group (Table 11). The incidences of severe symptomatic hypoglycemia were similar between groups *[see Adverse Reactions (6.1)]*.

Table 11: Type 2 Diabetes Mellitus – Adults

**Treatment duration Treatment in combination with**	**Study E 52 weeks Oral agents**		**Study F 28 weeks Regular insulin**		**Study G 5 years Regular insulin**	
	**LANTUS**	**NPH**	**LANTUS**	**NPH**	**LANTUS**	**NPH**
Number of subjects treated	289	281	259	259	513	504
**HbA1c**						
Baseline mean	9.0	8.9	8.6	8.5	8.4	8.3
Adjusted mean change from baseline	-0.5	-0.4	-0.4	-0.6	-0.6	-0.8
LANTUS – NPH	-0.1		+0.2		+0.2	
95% CI for Treatment difference	(-0.3; +0.1)		(0.0; +0.4)		(+0.1; +0.4)	
**Basal insulin dose***						
Baseline mean	14	15	44.1	45.5	39	44

Mean change from baseline	+12	+9	-1	+7	+23	+30
**Total insulin dose***						
Baseline mean	14	15	64	67	48	53
Mean change from baseline	+12	+9	+10	+13	+41	+40
**Fasting blood glucose (mg/dL)**						
Baseline mean	179	180	164	166	190	180
Adj. mean change from baseline	-49	-46	-24	-22	-45	-44
**Body weight (kg)**						
Baseline mean	83.5	82.1	89.6	90.7	100	99
Adj. mean change from baseline	2.0	1.9	0.4	1.4	3.7	4.8

* In Study G, the baseline dose of basal or total insulin was the first available on-treatment dose prescribed during the study (on visit month 1.5).

14.4 Additional Clinical Studies in Adults with Diabetes Type 1 and Type 2

Different Timing of LANTUS Administration in Diabetes Type 1 and Diabetes Type 2

The safety and efficacy of once daily LANTUS administered either at pre-breakfast, pre-dinner, or at bedtime were evaluated in a randomized, controlled clinical study in adult patients with type 1 diabetes (Study H, n=378). Patients were also treated with insulin lispro at mealtime. The average age was 41 years. All patients were White (100%) and 54% were male. The mean BMI was approximately 25.3 kg/m^2. The mean duration of diabetes was 17 years.

LANTUS administered at pre-breakfast or at pre-dinner (both once daily) resulted in similar reductions in HbA1c compared to that with bedtime administration (see Table 12). In these patients, data are available from 8-point home glucose monitoring. The maximum mean blood glucose was observed just prior to LANTUS injection regardless of time of administration. In this study, 5% of patients in the LANTUS-breakfast group discontinued treatment because of lack of efficacy. No patients in the other two groups (pre-dinner, bedtime) discontinued for this reason.

The safety and efficacy of once daily LANTUS administered pre-breakfast or at bedtime were also evaluated in a randomized, active-controlled clinical study (Study I, n=697) in patients with type 2 diabetes not adequately controlled on oral antidiabetic therapy. All patients in this study also received glimepiride 3 mg daily. The average age was 61 years. The majority of patients were White (97%) and 54% were male. The mean BMI was approximately 28.7 kg/m^2. The mean duration of diabetes was 10 years. LANTUS given before breakfast was at least as effective in lowering HbA1c as LANTUS given at bedtime or NPH insulin given at bedtime (see Table 12).

Table 12: Study of Different Times of Once Daily LANTUS Dosing in Type 1 (Study H) and Type 2 (Study I) Diabetes Mellitus

Treatment duration Treatment	Study H	Study I

Treatment in combination with	24 weeks Insulin lispro			24 weeks Glimepiride		
	**LANTUS Before Breakfast**	**LANTUS Before Dinner**	**LANTUS Bedtime**	**LANTUS Before Breakfast**	**LANTUS Bedtime**	**NPH Bedtime**
Number of subjects treated*	112	124	128	234	226	227
**HbA1c**						
Baseline mean	7.6	7.5	7.6	9.1	9.1	9.1
Mean change from baseline	-0.2	-0.1	0.0	-1.3	-1.0	-0.8
**Basal insulin dose (Units)**						
Baseline mean	22	23	21	19	20	19
Mean change from baseline	5	2	2	11	18	18
**Total insulin dose (Units)**	–	–	–	NA†	NA†	NA†
Baseline mean	52	52	49	–	–	–
Mean change from baseline	2	3	2	–	–	–
**Body weight (kg)**						
Baseline mean	77.1	77.8	74.5	80.7	82	81
Mean change from baseline	0.7	0.1	0.4	3.9	3.7	2.9

* Intent-to-treat
† Not applicable

Progression of Retinopathy Evaluation in Adults with Diabetes Type 1 and Diabetes Type 2

LANTUS was compared to NPH insulin in a 5-year randomized clinical study that evaluated the progression of retinopathy as assessed with fundus photography using a grading protocol derived from the Early Treatment Diabetic Retinopathy Scale (ETDRS). Patients had type 2 diabetes (mean age 55 years) with no (86%) or mild (14%) retinopathy at baseline. Mean baseline HbA1c was 8.4%. The primary outcome was progression by 3 or more steps on the ETDRS scale at study endpoint. Patients with prespecified postbaseline eye procedures (pan-retinal photocoagulation for proliferative or severe nonproliferative diabetic retinopathy, local photocoagulation for new vessels, and vitrectomy for diabetic retinopathy) were also considered as 3-step progressors regardless of actual change in ETDRS score from baseline. Retinopathy graders were blinded to treatment group assignment.

The results for the primary endpoint are shown in Table 13 for both the per-protocol

and intent-to-treat populations, and indicate similarity of LANTUS to NPH in the progression of diabetic retinopathy as assessed by this outcome. In this study, the numbers of retinal adverse events reported for LANTUS and NPH insulin treatment groups were similar for adult patients with type 1 and type 2 diabetes.

Table 13: Number (%) of Patients with 3 or More Step Progression on ETDRS Scale at Endpoint

	LANTUS (%)	NPH (%)	Difference*,† (SE)	95% CI for difference
**Per-protocol**	53/374 (14.2%)	57/363 (15.7%)	-2.0% (2.6%)	-7.0% to +3.1%
**Intent-to-Treat**	63/502 (12.5%)	71/487 (14.6%)	-2.1% (2.1%)	-6.3% to +2.1%

* Difference = LANTUS – NPH
† Using a generalized linear model (SAS GENMOD) with treatment and baseline HbA1c strata (cutoff 9.0%) as the classified independent variables, and with binomial distribution and identity link function

The ORIGIN Study of Major Cardiovascular Outcomes in Patients with Established CV Disease or CV Risk Factors

The Outcome Reduction with Initial Glargine Intervention study (i.e., ORIGIN) was an open-label, randomized, 2-by-2, factorial design study. One intervention in ORIGIN compared the effect of LANTUS to standard care on major adverse cardiovascular (CV) outcomes in 12,537 adults ≥ 50 years of age with:

- Abnormal glucose levels (i.e., impaired fasting glucose [IFG] and/or impaired glucose tolerance [IGT]) or early type 2 diabetes mellitus and
- Established CV disease or CV risk factors at baseline.

The objective of the study was to demonstrate that LANTUS use could significantly lower the risk of major CV outcomes compared to standard care. There were two coprimary composite CV endpoints:

- The first coprimary endpoint was the time to first occurrence of a major adverse CV event defined as the composite of CV death, nonfatal myocardial infarction, and nonfatal stroke.
- The second coprimary endpoint was the time to the first occurrence of CV death or nonfatal myocardial infarction or nonfatal stroke or revascularization procedure or hospitalization for heart failure.

Patients were randomized to either LANTUS (N=6,264) titrated to a goal fasting plasma glucose of ≤95 mg/dL or to standard care (N=6,273). Anthropometric and disease characteristics were balanced at baseline. The mean age was 64 years and 8% of patients were 75 years of age or older. The majority of patients were male (65%). Fifty nine percent were White, 25% were Latin, 10% were Asian and 3% were Black or African American. The median baseline BMI was 29 kg/m^2. Approximately 12% of patients had abnormal glucose levels (IGT and/or IFG) at baseline and 88% had type 2 diabetes. For patients with type 2 diabetes, 59% were treated with a single oral antidiabetic drug, 23% had known diabetes but were on no antidiabetic drug and 6% were newly diagnosed during the screening procedure. The mean HbA1c (SD) at baseline was 6.5% (1.0). Fifty-nine percent of the patients had had a prior CV event and 39% had documented

coronary artery disease or other CV risk factors.

Vital status was available for 99.9% and 99.8% of patients randomized to LANTUS and standard care respectively at end of study. The median duration of follow-up was 6.2 years (range: 8 days to 7.9 years). The mean HbA1c (SD) at the end of the study was 6.5% (1.1) and 6.8% (1.2) in the LANTUS and standard care group respectively. The median dose of LANTUS at end of study was 0.45 U/kg. Eighty-one percent of patients randomized to LANTUS were using LANTUS at end of the study. The mean change in body weight from baseline to the last treatment visit was 2.2 kg greater in the LANTUS group than in the standard care group.

Overall, the incidence of major adverse CV outcomes was similar between groups (see Table 14). All-cause mortality was also similar between groups.

Table 14: Cardiovascular Outcomes in ORIGIN in Patients with Established CV Disease or CV Risk Factors – Time to First Event Analyses

	LANTUS N=6,264	Standard Care N=6,273	LANTUS vs Standard Care
	n (Events per 100 PY)	n (Events per 100 PY)	Hazard Ratio (95% CI)
**Coprimary endpoints**			
CV death, nonfatal myocardial infarction, or nonfatal stroke	1041 (2.9)	1013 (2.9)	1.02 (0.94, 1.11)
CV death, nonfatal myocardial infarction, nonfatal stroke, hospitalization for heart failure or revascularization procedure	1792 (5.5)	1727 (5.3)	1.04 (0.97, 1.11)
**Components of coprimary endpoints**			
CV death	580	576	1.00 (0.89, 1.13)
Myocardial Infarction (fatal or nonfatal)	336	326	1.03 (0.88, 1.19)
Stroke (fatal or nonfatal)	331	319	1.03 (0.89, 1.21)
Revascularizations	908	860	1.06 (0.96, 1.16)
Hospitalization for heart failure	310	343	0.90 (0.77, 1.05)

In the ORIGIN study, the overall incidence of cancer (all types combined) or death from cancer (Table 15) was similar between treatment groups.

Table 15: Cancer Outcomes in ORIGIN – Time to First Event Analyses

	LANTUS N=6,264	Standard Care N=6.273	LANTUS vs Standard Care
	n (Events per 100 PY)	n (Events per 100 PY)	Hazard Ratio (95% CI)
**Cancer endpoints**			
Any cancer event (new or recurrent)	559 (1.56)	561 (1.56)	0.99 (0.88, 1.11)
New cancer events	524 (1.46)	535 (1.49)	0.96 (0.85, 1.09)
Death due to Cancer	189 (0.51)	201 (0.54)	0.94 (0.77, 1.15)

16 HOW SUPPLIED/STORAGE AND HANDLING

16.1 How Supplied

LANTUS (insulin glargine) injection is supplied as a clear and colorless solution containing 100 units/mL (U-100) available as follows:

LANTUS	NDC number	Package size
10 mL Multiple-dose vial	0088-2220-33	1 vial per carton
3 mL SoloStar single-patient-use prefilled pen	0088-2219-05	5 pens per carton

Additional Information about LANTUS Solostar:
- The LANTUS SoloStar prefilled pen dials in 1-unit increments.
- Needles are not included in the packs.

Use BD Ultra-Fine® needles[1] with the SoloStar prefilled pens (these BD manufactured needles are sold separately).

---

1 Other brands listed are the trademarks of their respective owners and are not trademarks of sanofi-aventis U.S. LLC

16.2 Storage

Dispense in the original sealed carton with the enclosed Instructions for Use.

Store unused LANTUS in a refrigerator between 36°F and 46°F (2°C and 8°C). Do not freeze. Discard LANTUS if it has been frozen. Protect LANTUS from direct heat and light.

Storage conditions are summarized in the following table.

	Not in-use (unopened) Refrigerated	Not in-use (unopened) Room Temperature	In-use (opened)

	(36°F–46°F [2°C–8°C])	(up to 86°F [30°C])	(see temperature below)
10 mL multiple-dose vial	Until expiration date	28 days	28 days Refrigerated or room temperature
3 mL single-patient-use SoloStar prefilled pen	Until expiration date	28 days	28 days Room temperature only (Do not refrigerate)

17 PATIENT COUNSELING INFORMATION

Advise the patient to read the FDA-approved patient labeling (Patient Information and Instructions for Use). There are separate Instructions for Use for the Vial and LANTUS SoloStar Pen.

Never Share a LANTUS SoloStar Prefilled Pen or Insulin Syringe Between Patients

Advise patients that they must never share a LANTUS SoloStar pen with another person, even if the needle is changed. Advise patients using LANTUS vials not to re-use or share needles or insulin syringes with another person. Sharing carries a risk for transmission of blood-borne pathogens *[see Warnings and Precautions (5.1)]*.

Hyperglycemia or Hypoglycemia

Inform patients that hypoglycemia is the most common adverse reaction with insulin. Inform patients of the symptoms of hypoglycemia (e.g., impaired ability to concentrate and react). This may present a risk in situations where these abilities are especially important, such as driving or operating other machinery. Advise patients who have frequent hypoglycemia or reduced or absent warning signs of hypoglycemia to use caution when driving or operating machinery *[see Warnings and Precautions (5.3)].*

Advise patients that changes in insulin regimen can predispose to hyperglycemia or hypoglycemia and that changes in insulin regimen should be made under close medical supervision *[see Warnings and Precautions (5.2)]*.

Hypoglycemia Due to Medications Errors

Instruct patients to always check the insulin label before each injection to reduce the risk of a medication error *[see Warnings and Precautions (5.4)]*.

Hypersensitivity Reactions

Advise patients that hypersensitivity reactions have occurred with LANTUS. Inform patients about the symptoms of hypersensitivity reactions *[see Warnings and Precautions (5.5)].*

Manufactured by:
sanofi-aventis U.S. LLC
Bridgewater, NJ 08807
A SANOFI COMPANY

U.S. License No. 1752

©2023 sanofi-aventis U.S. LLC

LANTUS, TOUJEO and SoloStar are registered trademarks of sanofi-aventis U.S. LLC.

PATIENT INFORMATION
LANTUS® (LAN-tus)
(insulin glargine)
injection, for subcutaneous use
VIAL:100 units/mL (U-100)

Do not share your syringes with other people, even if the needle has been changed. You may give other people a serious infection, or get a serious infection from them.

What is LANTUS?

LANTUS is a long-acting man-made-insulin used to control high blood sugar in adults and children with diabetes mellitus. LANTUS is not for use to treat diabetic ketoacidosis.

Who should not use LANTUS?

Do not use LANTUS if you:

- are having an episode of low blood sugar (hypoglycemia).
- have an allergy to insulin glargine or any of the ingredients in LANTUS. See the end of this Patient Information leaflet for a complete list of ingredients in LANTUS.

What should I tell my healthcare provider before using LANTUS?

Before using LANTUS, tell your healthcare provider about all your medical conditions including if you:

- have liver or kidney problems.
- take other medicines, especially ones called TZDs (thiazolidinediones).
- have heart failure or other heart problems. If you have heart failure, it may get worse while you take TZDs with LANTUS.
- are pregnant, planning to become pregnant, or are breastfeeding. It is not known if LANTUS may harm your unborn baby or breastfeeding baby.

Tell your healthcare provider about all the medicines you take including prescription and over-the-counter medicines, vitamins, and herbal supplements.

Before you start using LANTUS, talk to your healthcare provider about low blood sugar and how to manage it.

How should I use LANTUS?

- Read the detailed **Instructions for Use** that come with your LANTUS insulin.
- Use LANTUS exactly as your healthcare provider tells you to. Your healthcare provider should tell you how much LANTUS to use and when to use it.
- Know the amount of LANTUS you use. **Do not** change the amount of LANTUS you use unless your healthcare provider tells you to.
- Check your insulin label each time you give your injection to make sure you are using the correct insulin.
- **Do not** re-use needles. Always use a new needle for each injection. Re-use of needles increases your risk of having blocked needles, which may cause you to get the wrong dose of LANTUS. Using a new needle for each injection lowers your risk of getting an infection.
- You may take LANTUS at any time during the day but you must take it at the same

time every day.

- Only use LANTUS that is clear and colorless. If your LANTUS is cloudy or slightly colored, return it to your pharmacy for a replacement.
- LANTUS is injected under the skin (subcutaneously) of your upper legs (thighs), upper arms, or stomach area (abdomen).
- **Do not** use LANTUS in an insulin pump or inject LANTUS into your vein (intravenously).
- **Change (rotate) injection sites within the area you chose with each dose** to reduce your risk of getting lipodystrophy (pits in skin or thickened skin) and localized cutaneous amyloidosis (skin with lumps) at the injection sites.
  - **Do not** use the exact same spot for each injection.
  - **Do not** inject where the skin has pits, is thickened, or has lumps.
  - **Do not** inject where the skin is tender, bruised, scaly or hard, or into scars or damaged skin.
- **Do not** mix LANTUS with any other type of insulin or liquid medicine.
- **Check your blood sugar levels.** Ask your healthcare provider what your blood sugar should be and when you should check your blood sugar levels.

Keep LANTUS and all medicines out of the reach of children.

Your dose of LANTUS may need to change because of:

- a change in level of physical activity or exercise, weight gain or loss, increased stress, illness, change in diet, or because of the medicines you take.

What should I avoid while using LANTUS?
While using LANTUS do not:

- drive or operate heavy machinery, until you know how LANTUS affects you.
- drink alcohol or use over-the-counter medicines that contain alcohol.

What are the possible side effects of LANTUS and other insulins?
LANTUS may cause serious side effects that can lead to death, including:

- **low blood sugar (hypoglycemia).** Signs and symptoms that may indicate low blood sugar include:
  - dizziness or light-headedness, sweating, confusion, headache, blurred vision, slurred speech, shakiness, fast heartbeat, anxiety, irritability or mood change, hunger.
- **severe allergic reaction (whole body reaction). Get medical help right away if you have any of these signs or symptoms of a severe allergic reaction:**
  - a rash over your whole body, trouble breathing, a fast heartbeat, or sweating.
- **low potassium in your blood (hypokalemia).**
- **heart failure.** Taking certain diabetes pills called TZDs (thiazolidinediones) with LANTUS may cause heart failure in some people. This can happen even if you have never had heart failure or heart problems before. If you already have heart failure it may get worse while you take TZDs with LANTUS. Your healthcare provider should monitor you closely while you are taking TZDs with LANTUS. Tell your healthcare provider if you have any new or worse symptoms of heart failure including:
  - shortness of breath, swelling of your ankles or feet, sudden weight gain.

  Treatment with TZDs and LANTUS may need to be changed or stopped by your healthcare provider if you have new or worse heart failure.

Get emergency medical help if you have:

- trouble breathing; shortness of breath; fast heartbeat; swelling of your face, tongue, or throat; sweating; extreme drowsiness; dizziness; confusion.

The most common side effects of LANTUS include:

- low blood sugar (hypoglycemia); weight gain; allergic reactions, including reactions at your injection site; skin thickening or pits at the injection site (lipodystrophy).

These are not all the possible side effects of LANTUS. Call your doctor for medical advice about side effects. You may report side effects to FDA at 1-800-FDA-1088.

General information about the safe and effective use of LANTUS.
Medicines are sometimes prescribed for purposes other than those listed in a Patient Information leaflet. **Do not** use LANTUS for a condition for which it was not prescribed. **Do not** give LANTUS to other people, even if they have the same symptoms that you have. It may harm them.
This Patient Information leaflet summarizes the most important information about LANTUS. If you would like more information, talk with your healthcare provider. You can ask your pharmacist or healthcare provider for information about LANTUS that is written for healthcare professionals. For more information, go to www.lantus.com or call 1-800-633-1610.

What are the ingredients in LANTUS?

- **Active ingredient:** insulin glargine
- **10 mL vial inactive ingredients:** glycerol 85%, m-cresol, polysorbate 20, zinc, and Water for Injection, USP. Hydrochloric acid and sodium hydroxide may be added to adjust the pH.

Manufactured by: sanofi-aventis U.S. LLC, Bridgewater, NJ 08807, A SANOFI COMPANY.
U.S. License No. 1752

This Patient Information has been approved by the U.S. Food and Drug Administration.
Revised: June 2023

PATIENT INFORMATION
LANTUS® SOLOSTAR (LAN-tus)
(insulin glargine)
injection, for subcutaneous use
100 units/mL (U-100)

Do not share your LANTUS SoloStar® pen with other people, even if the needle has been changed. You may give other people a serious infection, or get a serious infection from them.

What is LANTUS?
LANTUS is a long-acting man-made insulin used to control high blood sugar in adults and children with diabetes mellitus. LANTUS is not for use to treat diabetic ketoacidosis.

Who should not use LANTUS?
Do not use LANTUS if you:

- are having an episode of low blood sugar (hypoglycemia).
- have an allergy to insulin glargine or any of the ingredients in LANTUS. See the end of this Patient Information leaflet for a complete list of ingredients in LANTUS.

What should I tell my healthcare provider before using LANTUS?
Before using LANTUS, tell your healthcare provider about all your medical conditions including if you:

- have liver or kidney problems.
- take other medicines, especially ones called TZDs (thiazolidinediones).
- have heart failure or other heart problems. If you have heart failure, it may get worse while you take TZDs with LANTUS.
- are pregnant, planning to become pregnant, or are breastfeeding. It is not known if LANTUS may harm your unborn baby or breastfeeding baby.

Tell your healthcare provider about all the medicines you take including prescription and over-the-counter medicines, vitamins, and herbal supplements.
Before you start using LANTUS, talk to your healthcare provider about low blood sugar and how to manage it.

How should I use LANTUS SoloStar?

- Read the detailed **Instructions for Use** that come with your LANTUS SoloStar single-patient-use prefilled pen.
- Use LANTUS exactly as your healthcare provider tells you to. Your healthcare provider should tell you how much LANTUS to use and when to use it.
- Know the amount of LANTUS you use. **Do not** change the amount of LANTUS you use unless your healthcare provider tells you to.
- Check your insulin label each time you give your injection to make sure you are using the correct insulin.
- The dose counter on your SoloStar pen shows your dose of LANTUS. Do not make any dose changes unless your healthcare provider tells you to.
- **Do not** use a syringe to remove LANTUS from your SoloStar disposable prefilled pen.
- **Do not** re-use needles. Always use a new needle for each injection. Re-use of needles increases your risk of having blocked needles, which may cause you to get the wrong dose of LANTUS. Using a new needle for each injection lowers your risk of getting an infection. If your needle is blocked, follow the instructions in **Step 3** of the **Instructions for Use**.
- You may take LANTUS at any time during the day but you must take it at the same time every day.
- LANTUS is injected under the skin (subcutaneously) of your upper legs (thighs), upper arms, or stomach area (abdomen).
- **Do not** use LANTUS in an insulin pump or inject LANTUS into your vein (intravenously).
- **Change (rotate) your injection sites within area you chose with each dose** to reduce your risk of getting lipodystrophy (pits in skin or thickened skin) and localized cutaneous amyloidosis (skin with lumps) at the injection sites.
  - **Do not** use the exact same spot for each injection.
  - **Do not** inject where the skin has pits, is thickened, or has lumps.
  - **Do not** inject where skin is tender, bruised, scaly or hard, or into scars or damaged skin.
- **Do not** mix LANTUS with any other type of insulin or liquid medicine.
- **Check your blood sugar levels.** Ask your healthcare provider what your blood sugar should be and when you should check your blood sugar levels.

Keep LANTUS and all medicines out of the reach of children.

Your dose of LANTUS may need to change because of:

- a change in level of physical activity or exercise, weight gain or loss, increased stress, illness, change in diet, or because of the medicines you take.

What should I avoid while using LANTUS?
While using LANTUS do not:

- drive or operate heavy machinery, until you know how LANTUS affects you.
- drink alcohol or use over-the-counter medicines that contain alcohol.

What are the possible side effects of LANTUS and other insulins?
LANTUS may cause serious side effects that can lead to death, including:

- **low blood sugar (hypoglycemia).** Signs and symptoms that may indicate low blood sugar include:
  - dizziness or light-headedness, sweating, confusion, headache, blurred vision, slurred speech, shakiness, fast heartbeat, anxiety, irritability or mood change, hunger.
- **severe allergic reaction (whole body reaction). Get medical help right away if you have any of these signs or symptoms of a severe allergic reaction:**
  - a rash over your whole body, trouble breathing, a fast heartbeat, or sweating.
- **low potassium in your blood (hypokalemia).**
- **heart failure.** Taking certain diabetes pills called TZDs (thiazolidinediones) with LANTUS may cause heart failure in some people. This can happen even if you have never had heart failure or heart problems before. If you already have heart failure it may get worse while you take TZDs with LANTUS. Your healthcare provider should monitor you closely while you are taking TZDs with LANTUS. Tell your healthcare provider if you have any new or worse symptoms of heart failure including:
  - shortness of breath, swelling of your ankles or feet, sudden weight gain.

  Treatment with TZDs and LANTUS may need to be changed or stopped by your healthcare provider if you have new or worse heart failure.

Get emergency medical help if you have:

- trouble breathing; shortness of breath; fast heartbeat; swelling of your face, tongue, or throat; sweating; extreme drowsiness; dizziness; confusion.

The most common side effects of LANTUS include:

- low blood sugar (hypoglycemia); weight gain; allergic reactions, including reactions at your injection site; skin thickening or pits at the injection site (lipodystrophy).

These are not all the possible side effects of LANTUS. Call your doctor for medical advice about side effects. You may report side effects to FDA at 1-800-FDA-1088.

General information about the safe and effective use of LANTUS.
Medicines are sometimes prescribed for purposes other than those listed in a Patient Information leaflet. **Do not** use LANTUS for a condition for which it was not prescribed. **Do not** give LANTUS to other people, even if they have the same symptoms that you have. It may harm them.
This Patient Information leaflet summarizes the most important information about LANTUS. If you would like more information, talk with your healthcare provider. You can

ask your healthcare provider or pharmacist for information about LANTUS that is written for healthcare professionals. For more information about LANTUS call 1-800-633-1610 or go to the website www.lantus.com.
**What are the ingredients in LANTUS?**<br>• **Active ingredient:** insulin glargine<br>• **3 mL SoloStar prefilled pen inactive ingredients:** glycerol 85%, m-cresol, zinc, and Water for Injection, USP. Hydrochloric acid and sodium hydroxide may be added to adjust the pH.
Manufactured by: sanofi-aventis U.S. LLC, Bridgewater, NJ 08807, A SANOFI COMPANY.<br>U.S. License No. 1752

This Patient Information has been approved by the U.S. Food and Drug Administration.

Revised: June 2023

INSTRUCTIONS FOR USE
LANTUS® (LAN-tus)
(insulin glargine)
injection, for subcutaneous use
VIAL: 100 units/mL (U-100)

These Instructions for Use contain information on how to inject LANTUS using the vial. Read these Instructions for Use before you start taking LANTUS and each time you get a new LANTUS vial. There may be new information. This information does not take the place of talking to your healthcare provider about your medical condition or your treatment.

Do not share your LANTUS syringes with other people, even if the needle has been changed. You may give other people a serious infection, or get a serious infection from them.

Supplies Needed to Give Your Injection:

- a LANTUS 10 mL vial
- a U-100 insulin syringe and needle
- 2 alcohol swabs
- 1 sharps container for throwing away used needles and syringes. See **"Disposing of used needles and syringes"** at the end of these instructions.

Preparing to Inject LANTUS:

- Wash your hands with soap and water or clean your hands with alcohol.
- Check the LANTUS label to make sure you are taking the right type of insulin. This is especially important if you use more than 1 type of insulin.
- Check the LANTUS in the vial to make sure it is clear and colorless. **Do not** use LANTUS if it is colored or cloudy, or if you see particles in the solution.
- **Do not** use LANTUS after the expiration date stamped on the label or 28 days after you first use it.
- **Always use a syringe that is marked for U-100 insulin.** If you use a syringe other than a U-100 insulin syringe, you may get the wrong dose of LANTUS.
- **Always use a new syringe and a new needle for each injection to help prevent infections and prevent blocked needles.**

Step 1:

If you are using a new LANTUS vial, remove the protective cap. **Do not** remove the stopper.

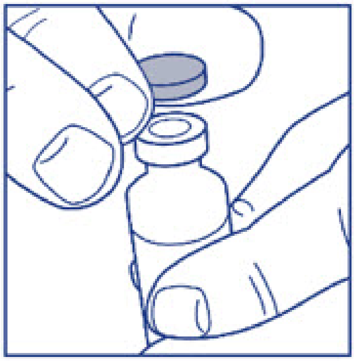

Step 2:

Wipe the top of the vial with an alcohol swab. You do not have to shake the vial of LANTUS before use.

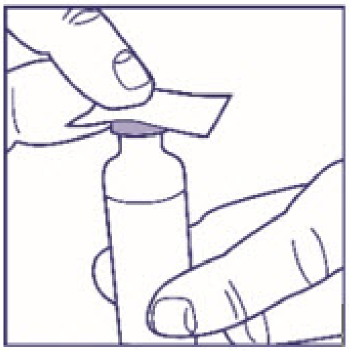

Step 3:

Draw air into the syringe equal to your LANTUS dose. Put the needle through the rubber top of the vial and push the plunger to inject the air into the vial.

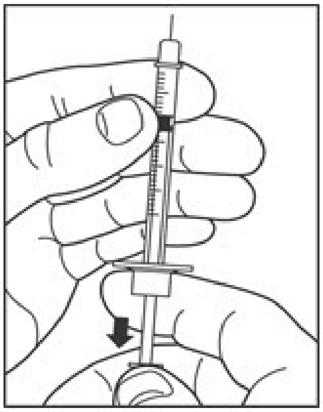

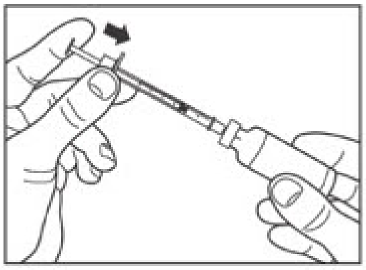

Step 4:

Leave the syringe in the vial and turn both upside down. Hold the syringe and vial firmly

in one hand. Make sure the tip of the needle is in the LANTUS solution. With your free hand, pull the plunger to withdraw the correct dose into the syringe.

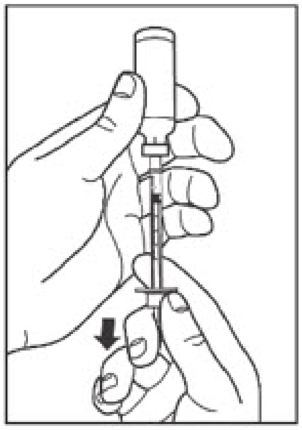

Step 5:

Before you take the needle out of the vial, check the syringe for air bubbles. If bubbles are in the syringe, hold the syringe straight up and tap the side of the syringe until the bubbles float to the top. Push the bubbles out with the plunger and draw insulin back in until you have the correct dose.

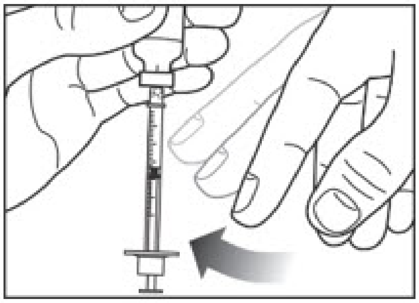

Step 6:

Remove the needle from the vial. Do not let the needle touch anything. You are now ready to inject.

Injecting LANTUS

- Inject your LANTUS (with a syringe) exactly as your healthcare provider has shown you.
- LANTUS is injected once daily at any time of the day but at the same time every day.

Step 7:

Choose your injection site:

- LANTUS is injected under the skin (subcutaneously) of your upper arms, thighs, or stomach area (abdomen).
- **Change (rotate) your injection sites within the area you choose for each dose** to reduce your risk of getting lipodystrophy (pits in the skin or thickened skin) and localized cutaneous amyloidosis (skin with lumps) at the injection sites.
- **Do not** inject where the skin has pits, is thickened, or has lumps.
- **Do not** inject where the skin is tender, bruised, scaly or hard, or into scars or damaged skin.

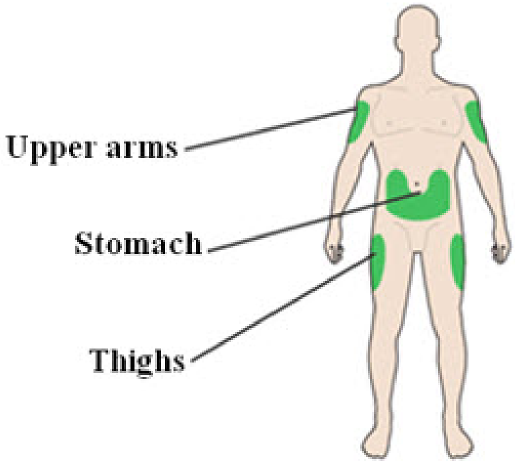

- Wipe the skin with an alcohol swab to clean the injection site. Let the injection site dry before you inject your dose.

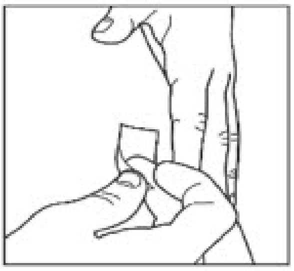

Step 8:
- Pinch the skin.
- Insert the needle under the skin in the way your healthcare provider showed you.
- Release the skin.
- Slowly push in the plunger of the syringe all the way, making sure you have injected all the LANTUS.
- Leave the needle in the skin for about **10** seconds.

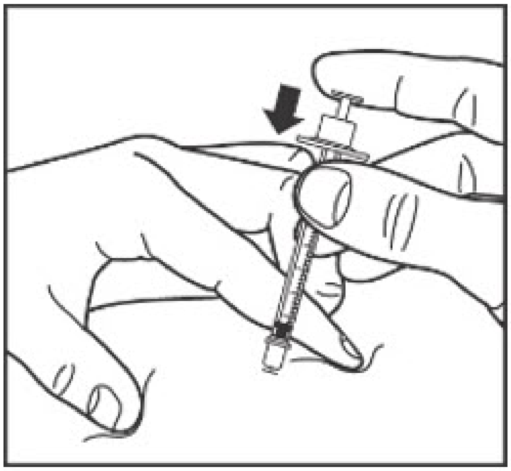

Step 9:
- Pull the needle straight out of your skin.
- Gently press the injection site for several seconds. **Do not** rub the area.

- **Do not** recap the used needle. Recapping the needle can lead to a needle-stick injury.

Disposing of Used Needles and Syringes

- Put your used needles and syringes in a FDA-cleared sharps disposal container right away after use. **Do not** throw away (dispose of) loose needles and syringes in your household trash.
- If you do not have a FDA-cleared sharps container, you may use a household container that is:
  - made of a heavy-duty plastic,
  - can be closed with a tight-fitting, puncture-resistant lid, without sharps being able to come out,
  - upright and stable during use,
  - leak resistant, and
  - properly labeled to warn of hazardous waste inside the container.
- When your sharps disposal container is almost full, you will need to follow your community guidelines for the right way to dispose of your sharps disposal container. There may be state or local laws about how you should throw away used needles and syringes. For more information about safe sharps disposal, and for specific information about sharps disposal in the state that you live in, go to the FDA's website at: http://www.fda.gov/safesharpsdisposal.
- **Do not** dispose of your used sharps disposal container in your household trash unless your community guidelines permit this. **Do not** recycle your used sharps disposal container.

Storing and Disposing LANTUS?

Unopened (not in-use) LANTUS vials

- Store unused LANTUS vials in the refrigerator from 36°F to 46°F (2°C to 8°C).
- **Do not** freeze LANTUS.
- Keep LANTUS away from direct heat and light.
- If a vial has been frozen or overheated, throw it away.
- Unopened vials can be used until the expiration date on the carton and vial label if they have been stored in the refrigerator (they can be stored past 28 days in the refrigerator).
- Unopened vials should be thrown away after 28 days if they are stored at room temperature.

After LANTUS vials have been opened (in-use)

- Store in-use (opened) LANTUS vials in a refrigerator from 36°F to 46°F (2°C to 8°C) or at room temperature below 86°F (30°C) for up to **28 days**.
- **Do not** freeze LANTUS. If a vial has been frozen, throw it away.
- Keep LANTUS out of direct heat and light.
- The LANTUS vial you are using should be thrown away after **28** days or if the expiration date has passed, even if it still has insulin left in it.

This Instructions for Use has been approved by the U.S. Food and Drug Administration.

Manufactured by:
sanofi-aventis U.S. LLC
Bridgewater, NJ 08807
A SANOFI COMPANY

U.S. License No. 1752
©2022 sanofi-aventis U.S. LLC.

Revised: June 2023

INSTRUCTIONS FOR USE
LANTUS® SOLOSTAR® (LAN-tus)
(insulin glargine)
injection, for subcutaneous use
3 mL Single-Patient-Use PREFILLED PEN: 100 units/mL (U-100)

Read these Instructions for Use before you start taking the LANTUS Solostar pen and each time you get a new LANTUS SoloStar pen. There may be new information. This information does not take the place of talking to your healthcare provider about your medical condition or your treatment.

Do not share your LANTUS SoloStar pen with other people, even if the needle has been changed. You may give other people a serious infection, or get a serious infection from them.

People who are blind or have vision problems should not use the LANTUS SoloStar prefilled pen without help from a person trained to use the LANTUS SoloStar prefilled pen.

LANTUS SoloStar is a disposable prefilled pen used to inject LANTUS. Each LANTUS SoloStar pen has 300 units of insulin which can be used for multiple injections. You can select doses from 1 to 80 units in steps of 1 unit. The pen plunger moves with each dose. The plunger will only move to the end of the cartridge when 300 units of LANTUS have been given.

Important Information You Need to Know Before Injecting LANTUS

- **Do not** use your pen if it is damaged or if you are not sure that it is working properly.
- **Do not** use a syringe to remove LANTUS from your pen.
- **Do not reuse needles.** If you do, you might get the wrong dose of LANTUS and/or increase the chance of getting an infection.
- Always perform a safety test (see **Step 3**).
- Always carry a spare pen and spare needles in case they got lost or stop working.
- Change (rotate) your injection sites within the area you choose for each dose (see **"Places to inject"**).

Learn to Inject

- Talk with your healthcare provider about how to inject before using your pen.
- Ask for help if you have problems handling the pen, for example if you have problems with your sight.
- Read all these instructions before using your pen. If you do not follow all these instructions, you may get too much or too little insulin.

Need Help?

If you have any questions about your pen or about diabetes, ask your healthcare provider, or go to www.Lantus.com or call sanofi-aventis at 1-800-633-1610.

Extra Items You Will Need

- a new sterile needle (see **Step 2**).
- an alcohol swab.
- a puncture-resistant container for used needles and pens. (See **"Throwing your pen away"**)

Places to Inject

- Inject your insulin exactly as your healthcare provider has shown you.
- Inject your insulin under the skin (subcutaneously) of your upper legs (thighs), upper arms, or stomach area (abdomen).
- Change (rotate) your injection sites within the area you choose for each dose to reduce your risk of getting lipodystrophy (pits in skin or thickened skin) and localized cutaneous amyloidosis (skin with lumps) at the injection sites.
- **Do not** inject where the skin has pits, is thickened, or has lumps.
- **Do not** inject where the skin is tender, bruised, scaly or hard, or into scars or damaged skin.

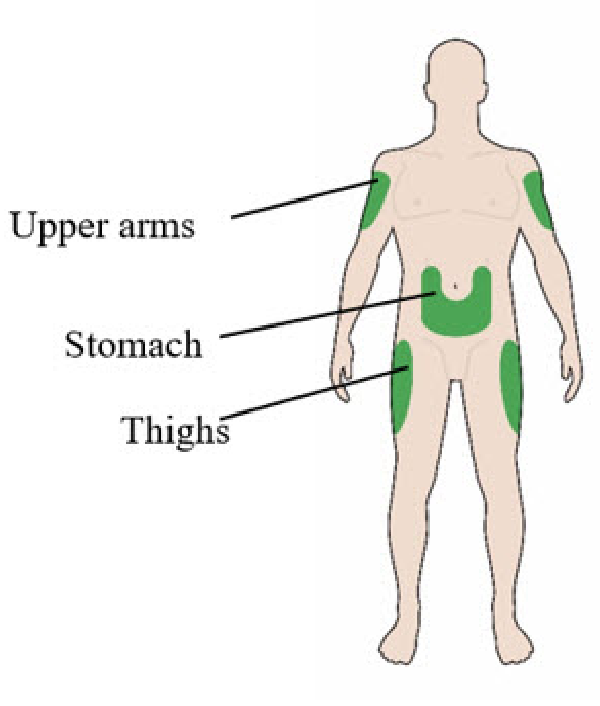

Get to know your pen

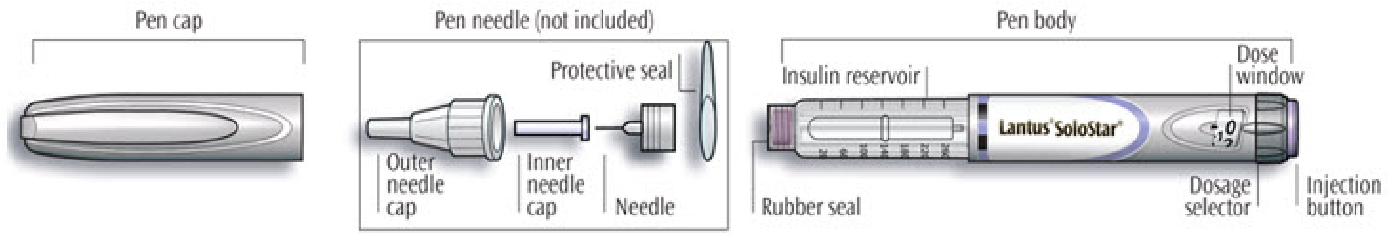

Step 1: Check your pen

Take a new pen out of the refrigerator at least **1** hour before you inject. Cold insulin is more painful to inject.

1A Check the name and expiration date on the label of your pen.

- Make sure you have the correct insulin.

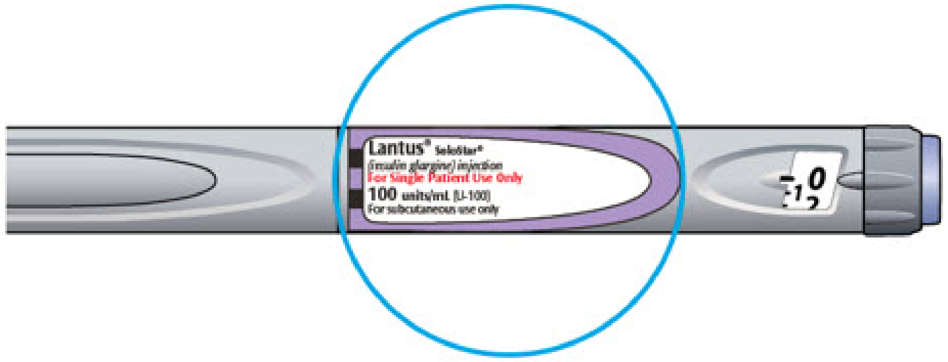

- **Do not** use your pen after the expiration date.

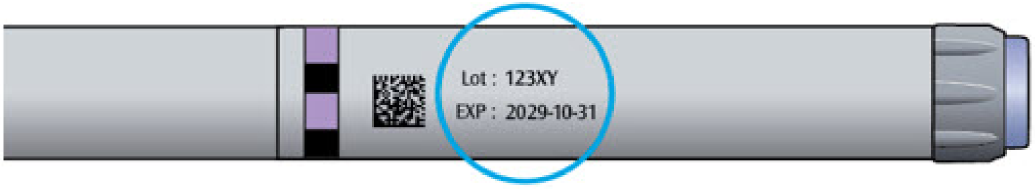

1B Pull off the pen cap.

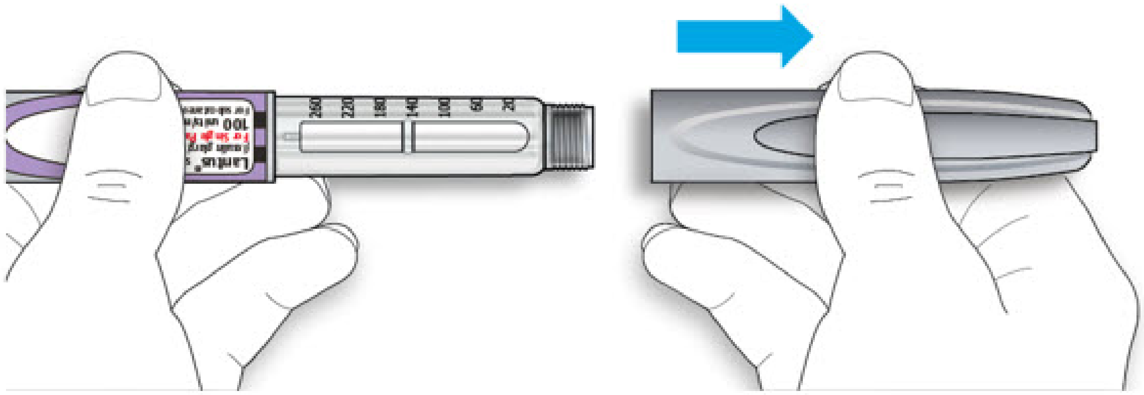

1C Check that the insulin is clear.

- **Do not** use the pen if the insulin looks cloudy, colored or contains particles.

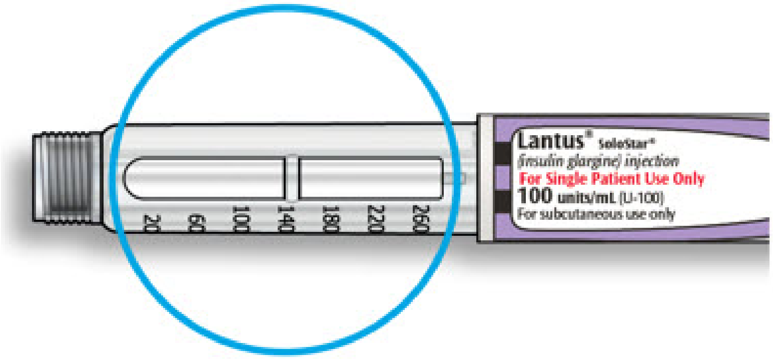

1D Wipe the rubber seal with an alcohol swab.

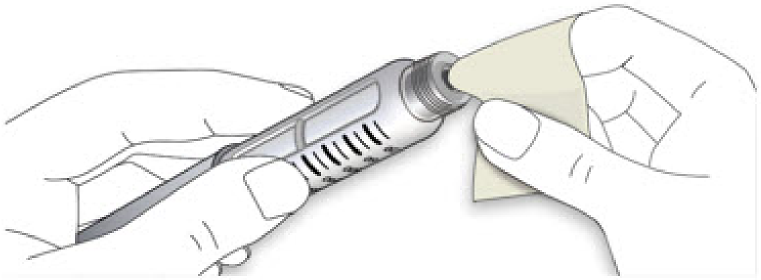

If you have other injector pens:

- Making sure you have the correct medicine is especially important if you have other injector pens.

Step 2: Attach a new needle

- **Do not** reuse needles. Always use a new sterile needle for each injection. This helps stop blocked needles, contamination, and infection.

Only use needles[2] that are compatible for use with LANTUS SoloStar, such as BD Ultra-Fine®.

2A Take a new needle and peel off the protective seal.

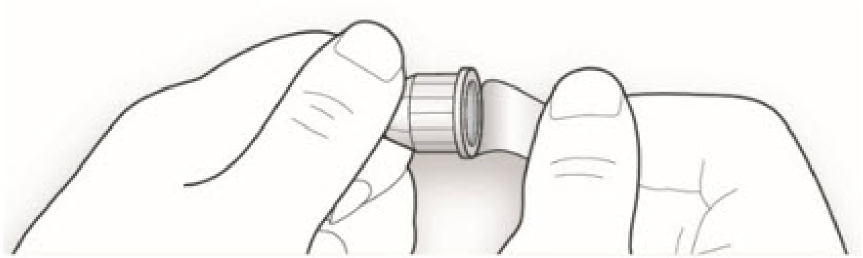

2B Keep the needle straight and screw it onto the pen until fixed. Do not over-tighten.

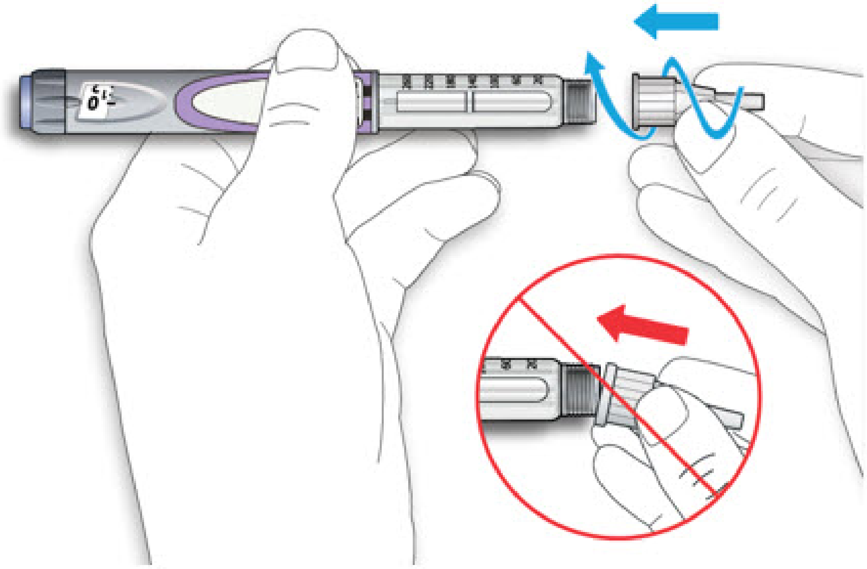

2C Pull off the outer needle cap. Keep this for later.

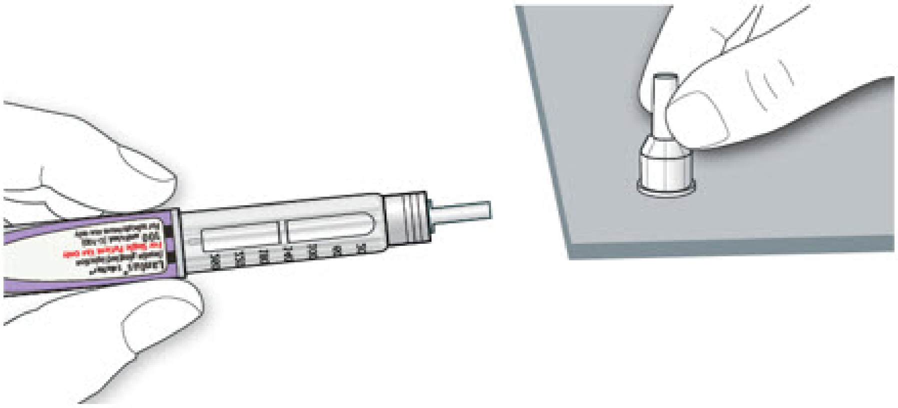

2D Pull off the inner needle cap and throw away.

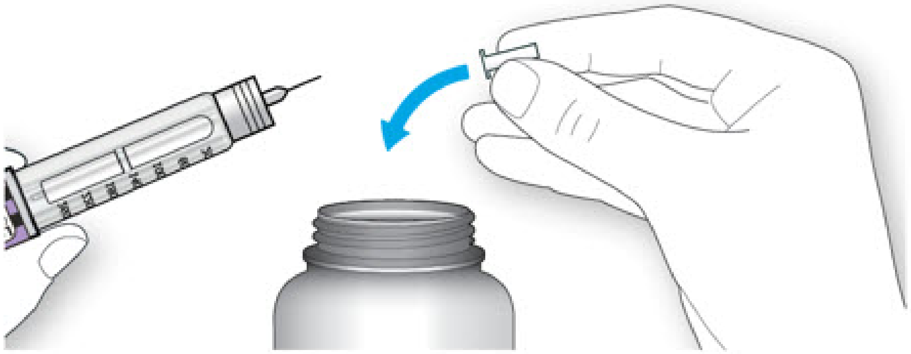

Handling needles

- Take care when handling needles to prevent needle-stick injury and cross-infection.

Step 3: Do a safety test

Always do a safety test before each injection to:

- Check your pen and the needle to make sure they are working properly.
- Make sure that you get the correct LANTUS dose.

3A Select 2 units by turning the dose selector until the dose pointer is at the 2 mark.

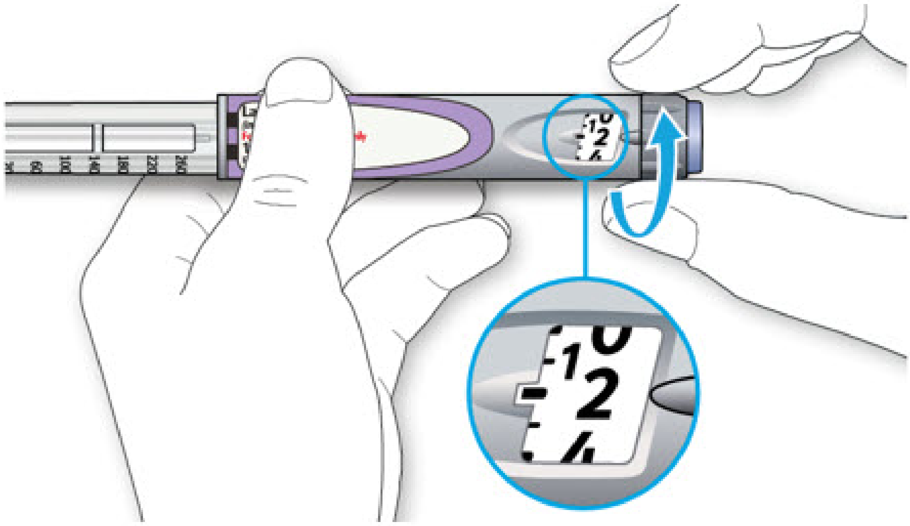

3B Press the injection button all the way in.

When insulin comes out of the needle tip, your pen is working correctly:

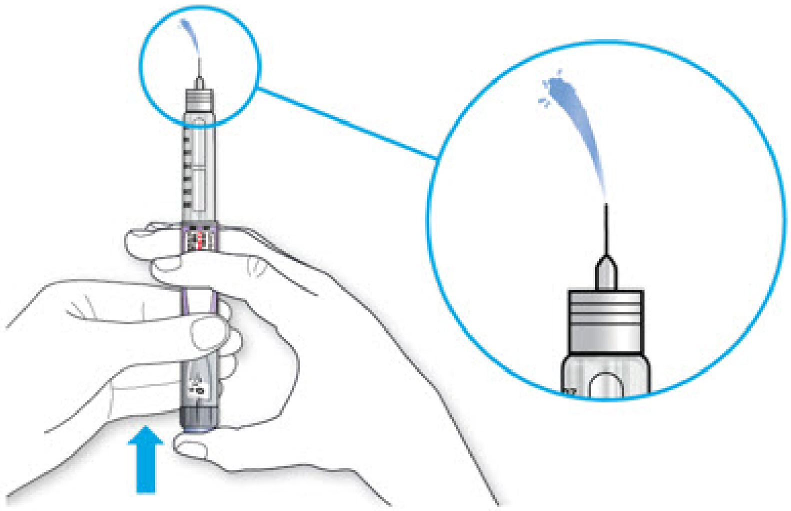

If no insulin appears:

- You may need to repeat this step up to 3 times before seeing insulin.
- If no insulin comes out after the third time, the needle may be blocked. If this happens:
    - change the needle (see **Step 6** and **Step 2**),
    - then repeat the safety test (**Step 3**).
- **Do not** use your pen if there is still no insulin coming out of the needle tip. Use a new pen.
- **Do not** use a syringe to remove insulin from your pen.

If you see air bubbles:

- You may see air bubbles in the insulin. This is normal, they will not harm you.

Step 4: Select the dose

Do not select a dose or press the injection button without a needle attached. This may damage your pen.

4A Make sure a needle is attached and the dose is set to "0."

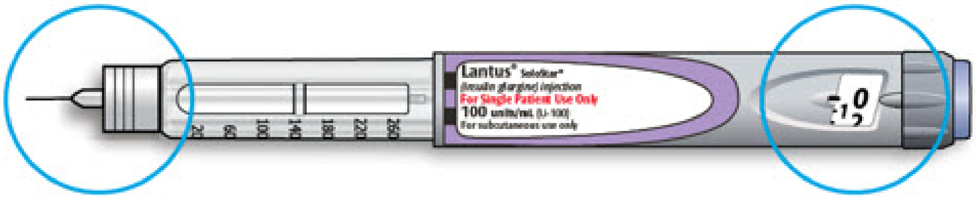

4B Turn the **dose selector until the dose pointer lines up with your dose.**

- If you turn past your dose, you can turn back down.
- If there are not enough units left in your pen for your dose, the dose selector will stop at the number of units left.
- If you cannot select your full prescribed dose, use a new pen or inject the remaining units and use a new pen to complete your dose.

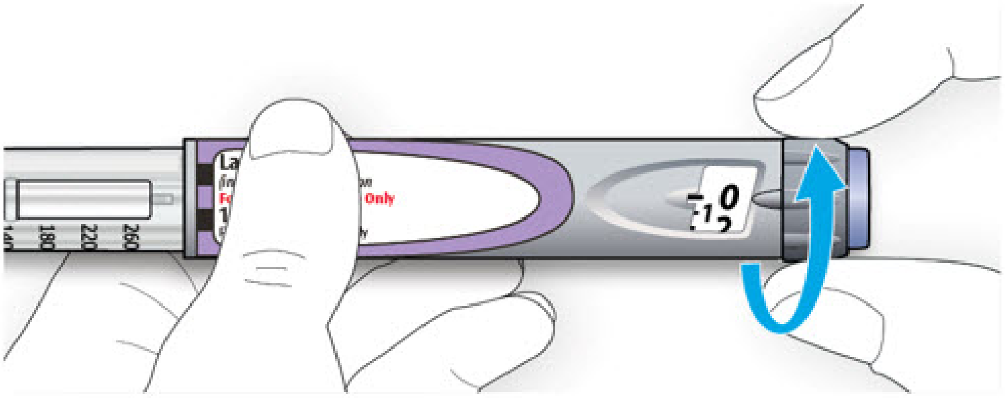

How to read the dose window

Even numbers are shown in line with dose pointer.

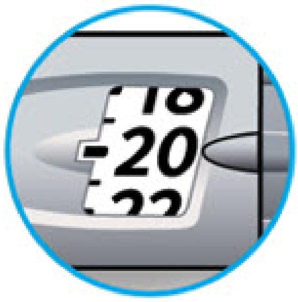

20 units selected

Odd numbers are shown as a line between even numbers.

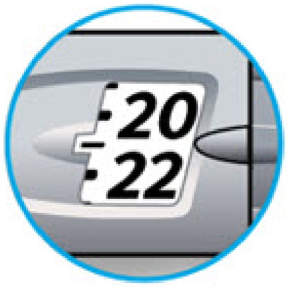

21 units selected

Units of LANTUS in your pen:

- Your pen contains a total of **300** units of LANTUS. You can select doses from **1** to **80** units in steps of **1** unit. Each pen contains more than 1 dose.
- You can see roughly how many units of insulin are left by looking at where the plunger is on the insulin scale.

Step 5: Injecting Your LANTUS Dose

If you find it hard to press the injection button in, **do not** force it as this may break your pen. See the section below for help.

5A Choose a place to inject as shown in the picture above.

5B Push the needle into your skin as shown by your healthcare provider.

Do not touch the injection button yet.

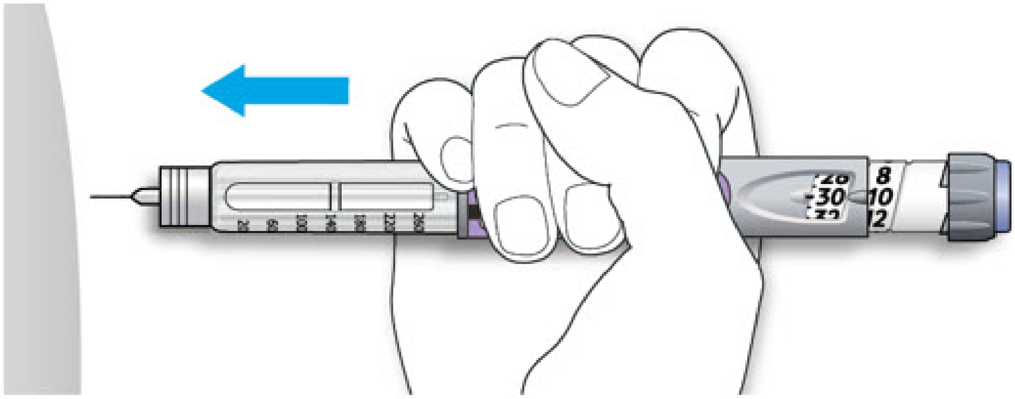

5C Place your thumb on the injection button. Then press all the way in and hold.

- **Do not** press at an angle. Your thumb could block the dose selector from turning.

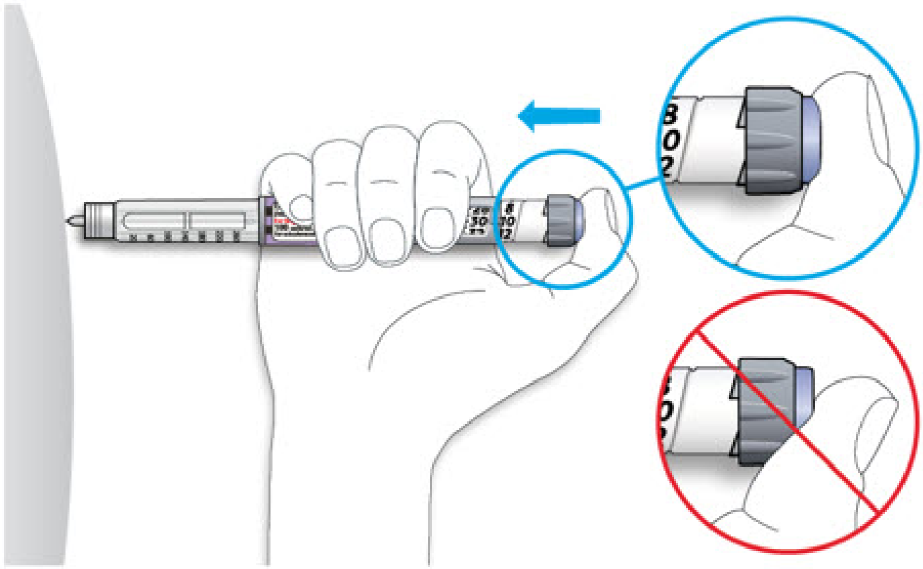

5D Keep the injection button **held in and when you see "0" in the dose window, slowly count to 10.**

- This will make sure you get your full dose.

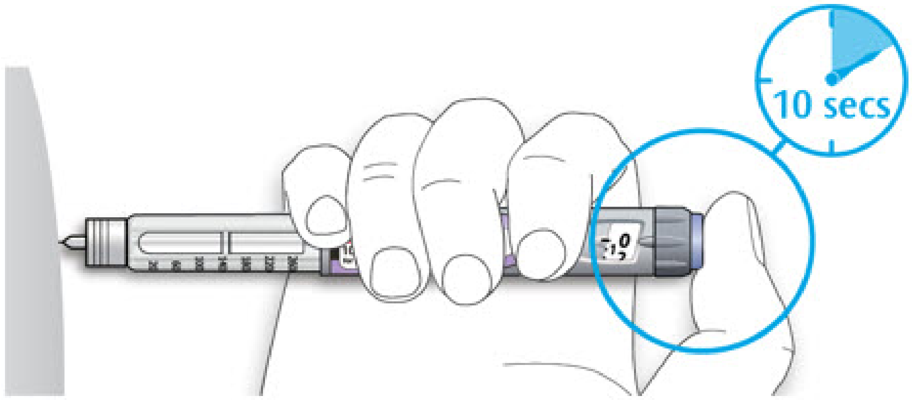

5E After holding and slowly counting to 10, release the injection button. Then remove the needle from your skin.

If you find it hard to press the button in:

- Change the needle (see **Step 6** and **Step 2**) then do a safety test (see **Step 3**).
- If you still find it hard to press in, get a new pen.
- **Do not** use a syringe to remove insulin from your pen.

Step 6: Remove the needle

- Take care when handling needles to prevent needle-stick injury and cross-infection.
- **Do not** put the inner needle cap back on.

6A Grip the widest part of the outer needle cap. Keep the needle straight and guide it into the outer needle cap. Then push firmly on.

- The needle can puncture the cap if it is recapped at an angle.

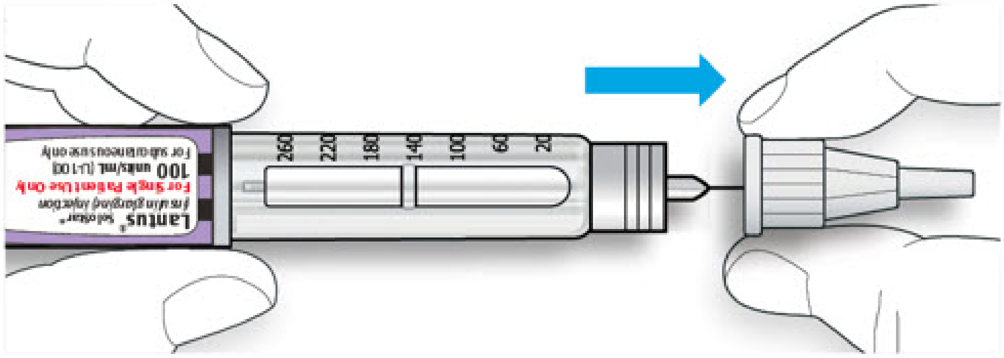

6B Grip and squeeze the widest part of the outer needle cap. Turn your pen several times with your other hand to remove the needle.

- Try again if the needle does not come off the first time.

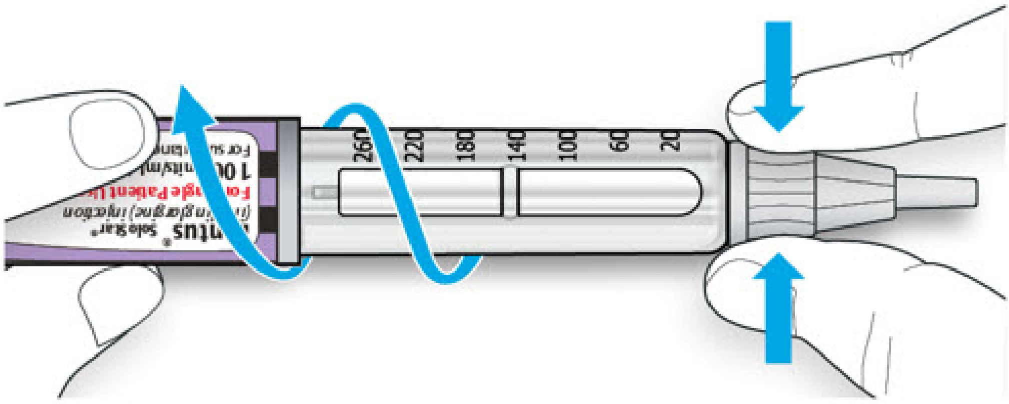

6C Throw away the used needle in a puncture-resistant container (see **"Throwing your pen away"** at the end of this Instructions for Use).

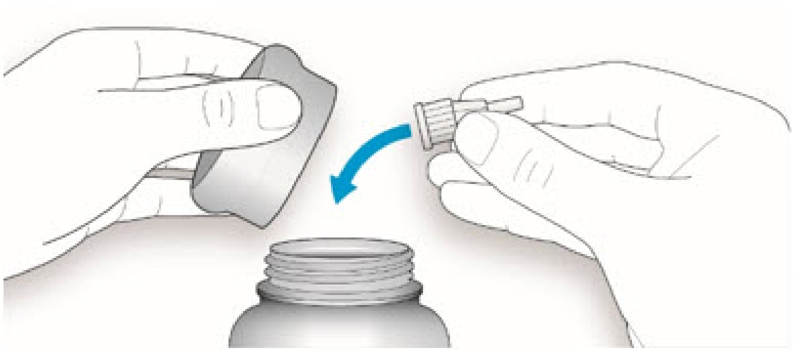

6D Put your pen cap back on.

- Do not put the pen back in the refrigerator.

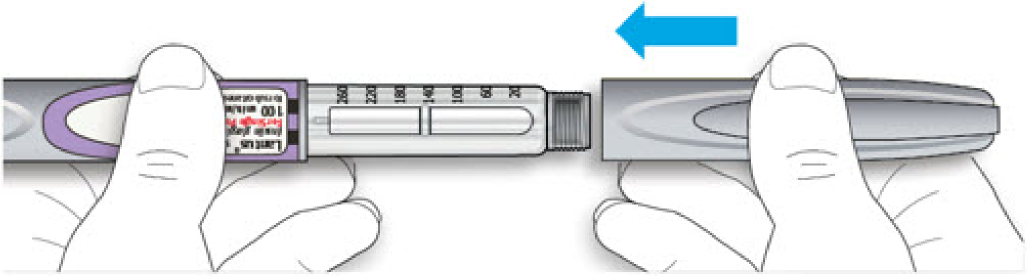

Storing the LANTUS Solostar Pen

Before first use

- Keep new pens in the refrigerator between **36°F to 46°F (2°C to 8°C)**.
- **Do not** freeze. **Do not** use LANTUS if it has been frozen.

After first use

- Keep your pen at room temperature **below 86°F (30°C)**.
- Keep your pen away from heat or light.
- Store your pen with the pen cap on.
- **Do not** put your pen back in the refrigerator.
- **Do not** store your pen with the needle attached.
- **Keep out of the reach of children.**
- Only use your pen for **up to 28 days** after its first use. Throw away the LANTUS SoloStar pen you are using after 28 days, even if it still has insulin left in it.

Caring for Your LANTUS SoloStar Pen

Handle your pen with care

- Do not drop your pen or knock it against hard surfaces.
- If you think that your pen may be damaged, **do not** try to fix it. Use a new one.

Protect **your pen from dust and dirt**

- You can clean the outside of your pen by wiping it with a damp cloth (water only). **Do not** soak, wash or lubricate your pen. This may damage it.

Throwing your pen away

- The used LANTUS SoloStar pen may be thrown away in your household trash after you have removed the needle.
- Put the used needle in an FDA-cleared sharps disposal container right away after use. **Do not** throw away (dispose of) the used needles in your household trash.
- If you do not have a FDA-cleared sharps disposal container, you may use a household container that is:

  - made of a heavy-duty plastic,
  - can be closed with a tight-fitting, puncture-resistant lid, without sharps being able to come out,
  - upright and stable during use,
  - leak-resistant, and
  - properly labeled to warn of hazardous waste inside the container.

- When your sharps disposal container is almost full, you will need to follow your community guidelines for the right way to dispose of your sharps disposal container. There may be state or local laws about how you should throw away used needles and syringes. For more information about safe sharps disposal, and for specific information about sharps disposal in the state that you live in, go to the FDA's website at: http://www.fda.gov/safesharpsdisposal.
- **Do not** dispose of your used sharps disposal container in your household trash unless your community guidelines permit this. **Do not** recycle your used sharps disposal container.

This Instructions for Use has been approved by the U.S. Food and Drug Administration.

Manufactured by:
sanofi-aventis U.S. LLC
Bridgewater, NJ 08807
A SANOFI COMPANY
U.S. License No. 1752
©2023 sanofi-aventis U.S. LLC.

Revised: June 2023

---

2 Other brands listed are the registered trademarks of their respective owners and are not trademarks of sanofi-aventis U.S. LLC.

PRINCIPAL DISPLAY PANEL - 10 mL Vial Package

NDC 0088-5021-01
Rx only

Lantus®
insulin glargine
injection

100 units/mL
(U-100)
For subcutaneous
injection only

Do not mix with
other insulins

Use only if solution
is clear and colorless
with no particles visible

Use with U-100
syringe only

One 10 mL multiple-dose vial

novaplus™

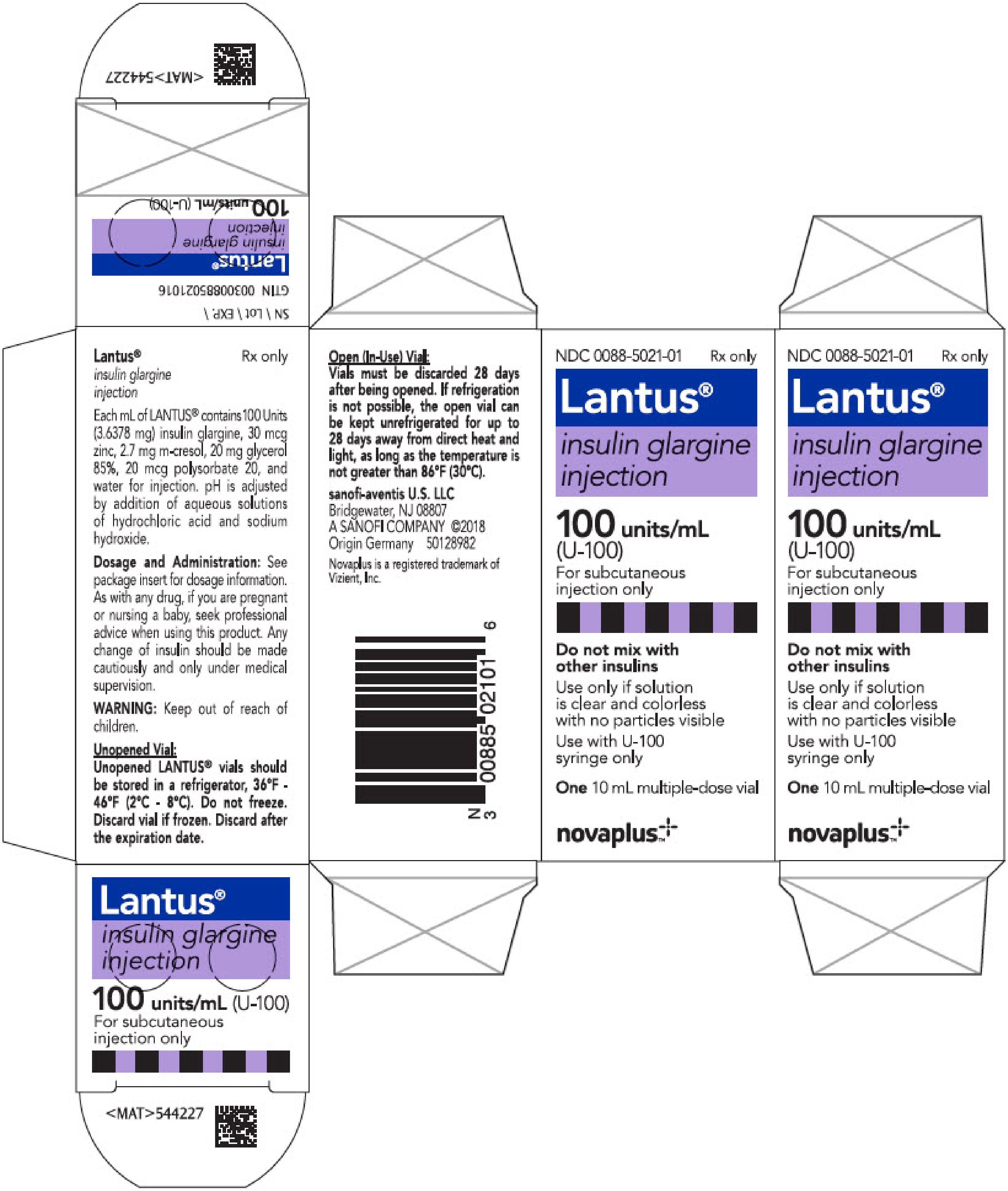

PRINCIPAL DISPLAY PANEL - 3 mL Syringe Package

NDC 0088-5020-05
Rx only

Lantus® SoloStar®

insulin glargine injection
For Single Patient Use Only
100 units/mL (U-100)
Five 3 mL Prefilled Pens

Solution for injection in a disposable insulin delivery device
Do not mix with other insulins
For subcutaneous injection only
Use only if solution is clear and colorless with no particles visible
Use within 28 days after initial use *Needles not include (see back panel)

novaplus™

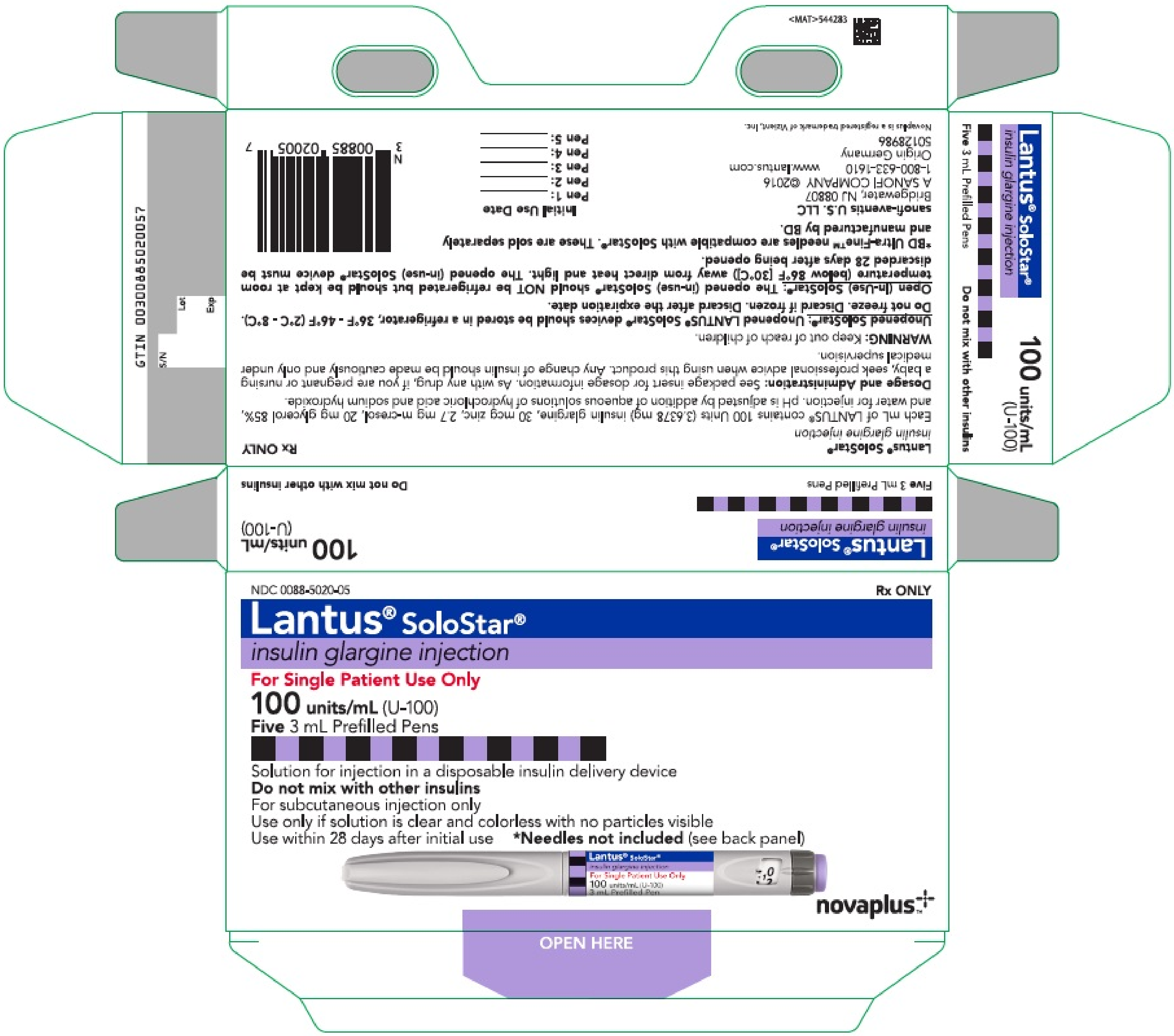

**LANTUS**
insulin glargine injection, solution

Product Information

**Product Type**	HUMAN PRESCRIPTION DRUG	**Item Code (Source)**	NDC:0088-5021
**Route of Administration**	SUBCUTANEOUS		

Active Ingredient/Active Moiety

Ingredient Name	Basis of Strength	Strength
**INSULIN GLARGINE** (UNII: 2ZM8CX04RZ) (INSULIN GLARGINE - UNII:2ZM8CX04RZ)	INSULIN GLARGINE	100 [iU] in 1 mL

Inactive Ingredients

Ingredient Name	Strength
**ZINC** (UNII: J41CSQ7QDS)	30 ug in 1 mL
**METACRESOL** (UNII: GGO4Y809LO)	2.7 mg in 1 mL
**GLYCERIN** (UNII: PDC6A3C0OX)	20 mg in 1 mL
**POLYSORBATE 20** (UNII: 7T1F30V5YH)	20 ug in 1 mL
**WATER** (UNII: 059QF0KO0R)	
**HYDROCHLORIC ACID** (UNII: QTT17582CB)	
**SODIUM HYDROXIDE** (UNII: 55X04QC32I)	

Packaging

#	Item Code	Package Description	Marketing Start Date	Marketing End Date
**1**	NDC:0088-5021-01	1 in 1 PACKAGE	06/04/2017	07/31/2024
**1**		10 mL in 1 VIAL, GLASS; Type 0: Not a Combination Product		

Marketing Information

Marketing Category	Application Number or Monograph Citation	Marketing Start Date	Marketing End Date
BLA	BLA021081	06/04/2017	

LANTUS SOLOSTAR

insulin glargine injection, solution

Product Information

**Product Type**	HUMAN PRESCRIPTION DRUG	**Item Code (Source)**	NDC:0088-5020
**Route of Administration**	SUBCUTANEOUS		

Active Ingredient/Active Moiety

Ingredient Name	Basis of Strength	Strength
**INSULIN GLARGINE** (UNII: 2ZM8CX04RZ) (INSULIN GLARGINE - UNII:2ZM8CX04RZ)	INSULIN GLARGINE	100 [iU] in 1 mL

Inactive Ingredients	
**Ingredient Name**	**Strength**
**ZINC** (UNII: J41CSQ7QDS)	30 ug in 1 mL
**METACRESOL** (UNII: GGO4Y809LO)	2.7 mg in 1 mL
**GLYCERIN** (UNII: PDC6A3C0OX)	20 mg in 1 mL
**WATER** (UNII: 059QF0KO0R)	
**HYDROCHLORIC ACID** (UNII: QTT17582CB)	
**SODIUM HYDROXIDE** (UNII: 55X04QC32I)	

Packaging				
**#**	**Item Code**	**Package Description**	**Marketing Start Date**	**Marketing End Date**
**1**	NDC:0088-5020-05	5 in 1 PACKAGE	06/04/2017	11/30/2023
**1**	NDC:0088-5020-01	3 mL in 1 SYRINGE; Type 3: Prefilled Biologic Delivery Device/System (syringe, patch, etc.)		

Marketing Information

Marketing Category	Application Number or Monograph Citation	Marketing Start Date	Marketing End Date
BLA	BLA021081	06/04/2017	

Labeler - sanofi-aventis U.S. LLC (783243835)

Establishment

Name	Address	ID/FEI	Business Operations
Sanofi-Aventis Deutschland GmbH		313218430	analysis(0088-5020, 0088-5021) , manufacture(0088-5020, 0088-5021) , api manufacture(0088-5020, 0088-5021) , pack(0088-5020, 0088-5021) , label(0088-5020, 0088-5021)

Establishment

Name	Address	ID/FEI	Business Operations
Sanofi S.r.l.		338454274	analysis(0088-5021) , manufacture(0088-5021) , pack(0088-5021) , label(0088-5021)

LIPITOR

LIPITOR- atorvastatin calcium tablet, film coated
Parke-Davis Div of Pfizer Inc

HIGHLIGHTS OF PRESCRIBING INFORMATION
These highlights do not include all the information needed to use LIPITOR safely and effectively. See full prescribing information for LIPITOR.

LIPITOR® (atorvastatin calcium) tablets, for oral use
Initial U.S. Approval: 1996

RECENT MAJOR CHANGES

Contraindications, Pregnancy and Lactation (4) Removed 12/2022
Warnings and Precautions, CNS Toxicity (5.5) Removed 12/2022

INDICATIONS AND USAGE

LIPITOR is an HMG-CoA reductase inhibitor (statin) indicated (1):

- To reduce the risk of:
  - Myocardial infarction (MI), stroke, revascularization procedures, and angina in adults with multiple risk factors for coronary heart disease (CHD) but without clinically evident CHD.
  - MI and stroke in adults with type 2 diabetes mellitus with multiple risk factors for CHD but without clinically evident CHD.
  - Non-fatal MI, fatal and non-fatal stroke, revascularization procedures, hospitalization for congestive heart failure, and angina in adults with clinically evident CHD.
- As an adjunct to diet to reduce low-density lipoprotein (LDL-C) in:
  - Adults with primary hyperlipidemia.
  - Adults and pediatric patients aged 10 years and older with heterozygous familial hypercholesterolemia (HeFH).
- As an adjunct to other LDL-C-lowering therapies to reduce LDL-C in adults and pediatric patients aged 10 years and older with homozygous familial hypercholesterolemia.
- As an adjunct to diet for the treatment of adults with:
  - Primary dysbetaliproteinemia.
  - Hypertriglyceridemia.

DOSAGE AND ADMINISTRATION

- Take orally once daily with or without food (2.1).
- Assess LDL-C when clinically appropriate, as early as 4 weeks after initiating LIPITOR, and adjust dosage if necessary (2.1).
- *Adults* (2.2):
  - Recommended starting dosage is 10 or 20 mg once daily; dosage range is 10 mg to 80 mg once daily.
  - Patients requiring LDL-C reduction >45% may start at 40 mg once daily.
- *Pediatric Patients Aged 10 Years of Age and Older with HeFH:* Recommended starting dosage is 10 mg once daily; dosage range is 10 to 20 mg once daily (2.3).
- *Pediatric Patients Aged 10 Years of Age and Older with HoFH:* Recommended starting dosage is 10 to 20 mg once daily; dosage range is 10 to 80 mg once daily (2.4).
- See full prescribing information for LIPITOR dosage modifications due to drug interactions (2.5).

DOSAGE FORMS AND STRENGTHS

Tablets: 10 mg; 20 mg; 40 mg; 80 mg of atorvastatin (3).

CONTRAINDICATIONS

- Acute liver failure or decompensated cirrhosis (4).
- Hypersensitivity to atorvastatin or any excipient in LIPITOR (4).

WARNINGS AND PRECAUTIONS

- *Myopathy and Rhabdomyolysis:* Risk factors include age 65 years or greater, uncontrolled hypothyroidism, renal impairment, concomitant use with certain other drugs, and higher LIPITOR dosage. Discontinue LIPITOR if markedly elevated CK levels occur or myopathy is diagnosed or suspected. Temporarily discontinue LIPITOR in patients experiencing an acute or serious condition at high risk of developing renal failure secondary to rhabdomyolysis. Inform patients of the risk of myopathy and rhabdomyolysis when starting or increasing LIPITOR dosage. Instruct patients to promptly report unexplained muscle pain, tenderness, or weakness, particularly if accompanied by malaise or fever (2.5, 5.1, 7.1, 8.5, 8.6).
- *Immune-Mediated Necrotizing Myopathy (IMNM):* Rare reports of IMNM, an autoimmune myopathy, have been reported with statin use. Discontinue LIPITOR if IMNM is suspected (5.2).
- *Hepatic Dysfunction:* Increases in serum transaminases have occurred, some persistent. Rare reports of fatal and non-fatal hepatic failure have occurred. Consider testing liver enzymes before initiating therapy and as clinically indicated thereafter. If serious hepatic injury with clinical symptoms and/or hyperbilirubinemia or jaundice occurs, promptly discontinue LIPITOR (5.3).

ADVERSE REACTIONS

Most common adverse reactions (incidence ≥5%) are nasopharyngitis, arthralgia, diarrhea, pain in extremity, and urinary tract infection (6.1).

To report SUSPECTED ADVERSE REACTIONS, contact Viatris at 1-877-446-3679 (1-877-4-INFO-RX) or FDA at 1-800-FDA-1088 or www.fda.gov/medwatch.

DRUG INTERACTIONS

- See full prescribing information for details regarding concomitant use of LIPITOR with other drugs or grapefruit juice that increase the risk of myopathy and rhabdomyolysis (2.5, 7.1).
- *Rifampin:* May reduce atorvastatin plasma concentrations. Administer simultaneously with LIPITOR (7.2).
- *Oral Contraceptives:* May increase plasma levels of norethindrone and ethinyl estradiol; consider this effect when selecting an oral contraceptive (7.3).
- *Digoxin:* May increase digoxin plasma levels; monitor patients appropriately (7.3).

USE IN SPECIFIC POPULATIONS

- *Pregnancy:* May cause fetal harm. (8.1).
- *Lactation:* Breastfeeding not recommended during treatment with LIPITOR (8.2).

See 17 for PATIENT COUNSELING INFORMATION and FDA-approved patient labeling.

Revised: 12/2022

FULL PRESCRIBING INFORMATION: CONTENTS*

1 INDICATIONS AND USAGE
2 DOSAGE AND ADMINISTRATION
2.1 Important Dosage Information
2.2 Recommended Dosage in Adult Patients
2.3 Recommended Dosage in Pediatric Patients 10 Years of Age and Older with HeFH
2.4 Recommended Dosage in Pediatric Patients 10 Years of Age and Older with HoFH
2.5 Dosage Modifications Due to Drug Interactions
3 DOSAGE FORMS AND STRENGTHS
4 CONTRAINDICATIONS
5 WARNINGS AND PRECAUTIONS
5.1 Myopathy and Rhabdomyolysis
5.2 Immune-Mediated Necrotizing Myopathy
5.3 Hepatic Dysfunction

5.4 Increases in HbA1c and Fasting Serum Glucose Levels
5.5 Increased Risk of Hemorrhagic Stroke in Patients on LIPITOR 80 mg with Recent Hemorrhagic Stroke
6 ADVERSE REACTIONS
6.1 Clinical Trials Experience
6.2 Postmarketing Experience
7 DRUG INTERACTIONS
7.1 Drug Interactions that may Increase the Risk of Myopathy and Rhabdomyolysis with LIPITOR
7.2 Drug Interactions that may Decrease Exposure to LIPITOR
7.3 LIPITOR Effects on Other Drugs
8 USE IN SPECIFIC POPULATIONS
8.1 Pregnancy
8.2 Lactation
8.4 Pediatric Use
8.5 Geriatric Use
8.6 Renal Impairment
8.7 Hepatic Impairment
10 OVERDOSAGE
11 DESCRIPTION
12 CLINICAL PHARMACOLOGY
12.1 Mechanism of Action
12.2 Pharmacodynamics
12.3 Pharmacokinetics
13 NONCLINICAL TOXICOLOGY
13.1 Carcinogenesis, Mutagenesis, Impairment of Fertility
14 CLINICAL STUDIES
16 HOW SUPPLIED/STORAGE AND HANDLING
17 PATIENT COUNSELING INFORMATION
* Sections or subsections omitted from the full prescribing information are not listed.

FULL PRESCRIBING INFORMATION

1 INDICATIONS AND USAGE

LIPITOR is indicated:

- To reduce the risk of:
  - Myocardial infarction (MI), stroke, revascularization procedures, and angina in adults with multiple risk factors for coronary heart disease (CHD) but without clinically evident CHD
  - MI and stroke in adults with type 2 diabetes mellitus with multiple risk factors for CHD but without clinically evident CHD
  - Non-fatal MI, fatal and non-fatal stroke, revascularization procedures, hospitalization for congestive heart failure, and angina in adults with clinically evident CHD

- As an adjunct to diet to reduce low-density lipoprotein cholesterol (LDL-C) in:
    - o Adults with primary hyperlipidemia.
    - o Adults and pediatric patients aged 10 years and older with heterozygous familial hypercholesterolemia (HeFH).
- As an adjunct to other LDL-C-lowering therapies, or alone if such treatments are unavailable, to reduce LDL-C in adults and pediatric patients aged 10 years and older with homozygous familial hypercholesterolemia (HoFH).
- As an adjunct to diet for the treatment of adults with:
    - o Primary dysbetalipoproteinemia
    - o Hypertriglyceridemia

2 DOSAGE AND ADMINISTRATION

2.1 Important Dosage Information

- Take Lipitor orally once daily at any time of the day, with or without food.
- Assess LDL-C when clinically appropriate, as early as 4 weeks after initiating LIPITOR, and adjust the dosage if necessary.

2.2 Recommended Dosage in Adult Patients

The recommended starting dosage of LIPITOR is 10 mg to 20 mg once daily. The dosage range is 10 mg to 80 mg once daily. Patients who require reduction in LDL-C greater than 45% may be started at 40 mg once daily.

2.3 Recommended Dosage in Pediatric Patients 10 Years of Age and Older with HeFH

The recommended starting dosage of LIPITOR is 10 mg once daily. The dosage range is 10 mg to 20 mg once daily.

2.4 Recommended Dosage in Pediatric Patients 10 Years of Age and Older with HoFH

The recommended starting dosage of LIPITOR is 10 mg to 20 mg once daily. The dosage range is 10 mg to 80 mg once daily.

2.5 Dosage Modifications Due to Drug Interactions

Concomitant use of LIPITOR with the following drugs requires dosage modification of LIPITOR *[see Warnings and Precautions (5.1) and Drug Interactions (7.1)]*.

<u>Anti-Viral Medications</u>

- In patients taking saquinavir plus ritonavir, darunavir plus ritonavir, fosamprenavir, fosamprenavir plus ritonavir, elbasvir plus grazoprevir or letermovir, do not exceed LIPITOR 20 mg once daily.
- In patients taking nelfinavir, do not exceed LIPITOR 40 mg once daily.

Select Azole Antifungals or Macrolide Antibiotics

- In patients taking clarithromycin or itraconazole, do not exceed LIPITOR 20 mg once daily.

For additional recommendations regarding concomitant use of LIPITOR with other anti-viral medications, azole antifungals or macrolide antibiotics, *see Drug Interactions (7.1)*.

3 DOSAGE FORMS AND STRENGTHS

LIPITOR tablets:

- 10 mg of atorvastatin: white elliptical, film-coated tablets with "PD 155" on one side and "10" on the other
- 20 mg of atorvastatin: white elliptical, film-coated tablets with "PD 156" on one side and "20" on the other
- 40 mg of atorvastatin: white elliptical, film-coated tablets with "PD 157" on one side and "40" on the other
- 80 mg of atorvastatin: white elliptical, film-coated tablets with "PD 158" on one side and "80" on the other

4 CONTRAINDICATIONS

- Acute liver failure or decompensated cirrhosis *[see Warnings and Precautions (5.3)]*
- Hypersensitivity to atorvastatin or any excipients in LIPITOR. Hypersensitivity reactions, including anaphylaxis, angioneurotic edema, erythema multiforme, Stevens-Johnson syndrome, and toxic epidermal necrolysis, have been reported *[see Adverse Reactions (6.2)]*.

5 WARNINGS AND PRECAUTIONS

5.1 Myopathy and Rhabdomyolysis

LIPITOR may cause myopathy (muscle pain, tenderness, or weakness associated with elevated creatine kinase [CK]) and rhabdomyolysis. Acute kidney injury secondary to myoglobinuria and rare fatalities have occurred as a result of rhabdomyolysis in patients treated with statins, including LIPITOR.

Risk Factors for Myopathy

Risk factors for myopathy include age 65 years or greater, uncontrolled hypothyroidism, renal impairment, concomitant use with certain other drugs (including other lipid-lowering therapies), and higher LIPITOR dosage *[see Drug Interactions (7.1) and Use in Specific Populations (8.5, 8.6)]*.

Steps to Prevent or Reduce the Risk of Myopathy and Rhabdomyolysis

LIPITOR exposure may be increased by drug interactions due to inhibition of cytochrome P450 enzyme 3A4 (CYP3A4) and/or transporters (e.g., breast cancer resistant protein [BCRP], organic anion-transporting polypeptide [OATP1B1/OATP1B3]

and P-glycoprotein [P-gp]), resulting in an increased risk of myopathy and rhabdomyolysis. Concomitant use of cyclosporine, gemfibrozil, tipranavir plus ritonavir, or glecaprevir plus pibrentasvir with LIPITOR is not recommended. LIPITOR dosage modifications are recommended for patients taking certain anti-viral, azole antifungals, or macrolide antibiotic medications *[see Dosage and Administration (2.5)]*. Cases of myopathy/rhabdomyolysis have been reported with atorvastatin co-administered with lipid modifying doses (>1 gram/day) of niacin, fibrates, colchicine, and ledipasvir plus sofosbuvir. Consider if the benefit of use of these products outweighs the increased risk of myopathy and rhabdomyolysis *[see Drug Interactions (7.1)]*.

Concomitant intake of large quantities, more than 1.2 liters daily, of grapefruit juice is not recommended in patients taking LIPITOR *[see Drug Interactions (7.1)]*.

Discontinue LIPITOR if markedly elevated CK levels occur or if myopathy is either diagnosed or suspected. Muscle symptoms and CK elevations may resolve if LIPITOR is discontinued. Temporarily discontinue LIPITOR in patients experiencing an acute or serious condition at high risk of developing renal failure secondary to rhabdomyolysis (e.g., sepsis; shock; severe hypovolemia; major surgery; trauma; severe metabolic, endocrine, or electrolyte disorders; or uncontrolled epilepsy).

Inform patients of the risk of myopathy and rhabdomyolysis when starting or increasing the LIPITOR dosage. Instruct patients to promptly report any unexplained muscle pain, tenderness or weakness, particularly if accompanied by malaise or fever.

5.2 Immune-Mediated Necrotizing Myopathy

There have been rare reports of immune-mediated necrotizing myopathy (IMNM), an autoimmune myopathy, associated with statin use, including reports of recurrence when the same or a different statin was administered. IMNM is characterized by proximal muscle weakness and elevated serum creatine kinase that persists despite discontinuation of statin treatment; positive anti-HMG CoA reductase antibody; muscle biopsy showing necrotizing myopathy; and improvement with immunosuppressive agents. Additional neuromuscular and serologic testing may be necessary. Treatment with immunosuppressive agents may be required. Discontinue LIPITOR if IMNM is suspected.

5.3 Hepatic Dysfunction

Increases in serum transaminases have been reported with use of LIPITOR *[see Adverse Reactions (6.1)]*. In most cases, these changes appeared soon after initiation, were transient, were not accompanied by symptoms, and resolved or improved on continued therapy or after a brief interruption in therapy. Persistent increases to more than three times the ULN in serum transaminases have occurred in approximately 0.7% of patients receiving LIPITOR in clinical trials. There have been rare postmarketing reports of fatal and non-fatal hepatic failure in patients taking statins, including LIPITOR.

Patients who consume substantial quantities of alcohol and/or have a history of liver disease may be at increased risk for hepatic injury *[see Use in Specific Populations (8.7)]*.

Consider liver enzyme testing before LIPITOR initiation and when clinically indicated thereafter. LIPITOR is contraindicated in patients with acute liver failure or decompensated cirrhosis *[see Contraindications (4)]*. If serious hepatic injury with clinical symptoms and/or hyperbilirubinemia or jaundice occurs, promptly discontinue LIPITOR.

5.4 Increases in HbA1c and Fasting Serum Glucose Levels

Increases in HbA1c and fasting serum glucose levels have been reported with statins, including LIPITOR. Optimize lifestyle measures, including regular exercise, maintaining a healthy body weight, and making healthy food choices.

5.5 Increased Risk of Hemorrhagic Stroke in Patients on LIPITOR 80 mg with Recent Hemorrhagic Stroke

In a post-hoc analysis of the Stroke Prevention by Aggressive Reduction in Cholesterol Levels (SPARCL) trial where 2365 adult patients, without CHD who had a stroke or TIA within the preceding 6 months, were treated with LIPITOR 80 mg, a higher incidence of hemorrhagic stroke was seen in the LIPITOR 80 mg group compared to placebo (55, 2.3% LIPITOR vs. 33, 1.4% placebo; HR: 1.68, 95% CI: 1.09, 2.59; p=0.0168). The incidence of fatal hemorrhagic stroke was similar across treatment groups (17 vs. 18 for the atorvastatin and placebo groups, respectively). The incidence of non-fatal hemorrhagic stroke was significantly higher in the LIPITOR group (38, 1.6%) as compared to the placebo group (16, 0.7%). Some baseline characteristics, including hemorrhagic and lacunar stroke on study entry, were associated with a higher incidence of hemorrhagic stroke in the LIPITOR group *[see Adverse Reactions (6.1)]*. Consider the risk/benefit of use of LIPITOR 80 mg in patients with recent hemorrhagic stroke.

6 ADVERSE REACTIONS

The following important adverse reactions are described below and elsewhere in the labeling:

- Myopathy and Rhabdomyolysis *[see Warnings and Precautions (5.1)]*
- Immune-Mediated Necrotizing Myopathy *[see Warnings and Precautions (5.2)]*
- Hepatic Dysfunction *[see Warnings and Precautions (5.3)]*
- Increases in HbA1c and Fasting Serum Glucose Levels *[see Warnings and Precautions (5.4)]*

6.1 Clinical Trials Experience

Because clinical trials are conducted under widely varying conditions, the adverse reaction rates observed in the clinical trials of a drug cannot be directly compared to rates in the clinical trials of another drug and may not reflect the rates observed in practice.

In the LIPITOR placebo-controlled clinical trial database of 16,066 patients (8755 LIPITOR vs. 7311 placebo; age range 10-93 years, 39% women, 91% White, 3% Black, 2% Asian, 4% other) with a median treatment duration of 53 weeks, the most common adverse reactions in patients treated with LIPITOR that led to treatment discontinuation and occurred at a rate greater than placebo were: myalgia (0.7%), diarrhea (0.5%), nausea (0.4%), alanine aminotransferase increase (0.4%), and hepatic enzyme increase (0.4%).

Table 1 summarizes adverse reactions reported in ≥ 2% and at a rate greater than placebo in patients treated with LIPITOR (n=8755), from seventeen placebo-controlled trials.

Table 1: Adverse Reactions Occurring in ≥ 2% in Patients LIPITOR-Treated with any Dose and Greater than Placebo

**Adverse Reaction**	**% Placebo N=7311**	**% 10 mg N=3908**	**% 20 mg N=188**	**% 40 mg N=604**	**% 80 mg N=4055**	**% Any dose N=8755**
Nasopharyngitis	8.2	12.9	5.3	7.0	4.2	8.3
Arthralgia	6.5	8.9	11.7	10.6	4.3	6.9
Diarrhea	6.3	7.3	6.4	14.1	5.2	6.8
Pain in extremity	5.9	8.5	3.7	9.3	3.1	6.0
Urinary tract infection	5.6	6.9	6.4	8.0	4.1	5.7
Dyspepsia	4.3	5.9	3.2	6.0	3.3	4.7
Nausea	3.5	3.7	3.7	7.1	3.8	4.0
Musculoskeletal pain	3.6	5.2	3.2	5.1	2.3	3.8
Muscle spasms	3.0	4.6	4.8	5.1	2.4	3.6
Myalgia	3.1	3.6	5.9	8.4	2.7	3.5
Insomnia	2.9	2.8	1.1	5.3	2.8	3.0
Pharyngolaryngeal pain	2.1	3.9	1.6	2.8	0.7	2.3

Other adverse reactions reported in placebo-controlled trials include:

Body as a whole: malaise, pyrexia

Digestive system: abdominal discomfort, eructation, flatulence, hepatitis, cholestasis

Musculoskeletal system: musculoskeletal pain, muscle fatigue, neck pain, joint swelling

Metabolic and nutritional system: transaminases increase, liver function test abnormal, blood alkaline phosphatase increase, creatine phosphokinase increase, hyperglycemia

Nervous system: nightmare

Respiratory system: epistaxis

Skin and appendages: urticaria

Special senses: vision blurred, tinnitus

Urogenital system: white blood cells urine positive

Elevations in Liver Enzyme Tests

Persistent elevations in serum transaminases, defined as more than 3 times the ULN and occurring on 2 or more occasions, occurred in 0.7% of patients who received LIPITOR in clinical trials. The incidence of these abnormalities was 0.2%, 0.2%, 0.6%, and 2.3% for 10, 20, 40, and 80 mg, respectively.

One patient in clinical trials developed jaundice. Increases in liver enzyme tests in other patients were not associated with jaundice or other clinical signs or symptoms. Upon dose reduction, drug interruption, or discontinuation, transaminase levels returned to or near pretreatment levels without sequelae. Eighteen of 30 patients with persistent liver enzyme elevations continued treatment with a reduced dose of LIPITOR.

Treating to New Targets Study (TNT)

In TNT, *[see Clinical Studies (14.1)]* 10,001 patients (age range 29-78 years, 19% women; 94% White, 3% Black, 1% Asian, 2% other) with clinically evident CHD were treated with LIPITOR 10 mg daily (n=5006) or LIPITOR 80 mg daily (n=4995). In the high-dose LIPITOR group, there were more patients with serious adverse reactions (1.8%) and discontinuations due to adverse reactions (9.9%) as compared to the low-dose group (1.4%; 8.1%, respectively) during a median follow-up of 4.9 years. Persistent transaminase elevations (≥3 x ULN twice within 4-10 days) occurred in 1.3% of individuals with LIPITOR 80 mg and in 0.2% of individuals with LIPITOR 10 mg. Elevations of CK (≥ 10 x ULN) were higher in the high-dose LIPITOR group (0.3%) compared to the low-dose LIPITOR group (0.1%).

Stroke Prevention by Aggressive Reduction in Cholesterol Levels (SPARCL)

In SPARCL, 4731 patients (age range 21-92 years, 40% women; 93% White, 3% Black, 1% Asian, 3% other) without clinically evident CHD but with a stroke or transient ischemic attack (TIA) within the previous 6 months were treated with LIPITOR 80 mg (n=2365) or placebo (n=2366) for a median follow-up of 4.9 years. There was a higher incidence of persistent hepatic transaminase elevations (≥ 3 x ULN twice within 4-10 days) in the LIPITOR group (0.9%) compared to placebo (0.1%). Elevations of CK (>10 x ULN) were rare, but were higher in the LIPITOR group (0.1%) compared to placebo (0.0%). Diabetes was reported as an adverse reaction in 6.1% of subjects in the LIPITOR group and 3.8% of subjects in the placebo group.

In a post-hoc analysis, LIPITOR 80 mg reduced the incidence of ischemic stroke (9.2% vs. 11.6%) and increased the incidence of hemorrhagic stroke (2.3% vs. 1.4%) compared to placebo. The incidence of fatal hemorrhagic stroke was similar between groups (17 LIPITOR vs. 18 placebo). The incidence of non-fatal hemorrhagic strokes was significantly greater in the LIPITOR group (38 non-fatal hemorrhagic strokes) as compared to the placebo group (16 non-fatal hemorrhagic strokes). Patients who entered the trial with a hemorrhagic stroke appeared to be at increased risk for hemorrhagic stroke (16% LIPITOR vs. 4% placebo).

Adverse Reactions from Clinical Studies of LIPITOR in Pediatric Patients with HeFH

In a 26-week controlled study in pediatric patients with HeFH (ages 10 years to 17 years) (n=140, 31% female; 92% White, 1.6% Blacks, 1.6% Asians, 4.8% other), the safety and tolerability profile of LIPITOR 10 to 20 mg daily, as an adjunct to diet to reduce total cholesterol, LDL-C, and apo B levels, was generally similar to that of placebo *[see Use in Specific Populations (8.4) and Clinical Studies (14.6)]*.

6.2 Postmarketing Experience

The following adverse reactions have been identified during post-approval use of LIPITOR. Because these reactions are reported voluntarily from a population of uncertain size, it is not always possible to reliably estimate their frequency or establish a causal relationship to drug exposure.

Gastrointestinal disorders: pancreatitis

General disorders: fatigue

Hepatobiliary Disorders: fatal and non-fatal hepatic failure

Immune system disorders: anaphylaxis

Injury: tendon rupture

Musculoskeletal and connective tissue disorders: rhabdomyolysis, myositis.

There have been rare reports of immune-mediated necrotizing myopathy associated with statin use.

Nervous system disorders: dizziness, peripheral neuropathy.

There have been rare reports of cognitive impairment (e.g., memory loss, forgetfulness, amnesia, memory impairment, confusion) associated with the use of all statins. Cognitive impairment was generally nonserious, and reversible upon statin discontinuation, with variable times to symptom onset (1 day to years) and symptom resolution (median of 3 weeks).

Psychiatric disorders: depression

Respiratory disorders: interstitial lung disease

Skin and subcutaneous tissue disorders: angioneurotic edema, bullous rashes (including erythema multiforme, Stevens-Johnson syndrome, and toxic epidermal necrolysis)

7 DRUG INTERACTIONS

7.1 Drug Interactions that may Increase the Risk of Myopathy and Rhabdomyolysis with LIPITOR

LIPITOR is a substrate of CYP3A4 and transporters (e.g., OATP1B1/1B3, P-gp, or BCRP). LIPITOR plasma levels can be significantly increased with concomitant administration of inhibitors of CYP3A4 and transporters. Table 2 includes a list of drugs that may increase exposure to LIPITOR and may increase the risk of myopathy and rhabdomyolysis when used concomitantly and instructions for preventing or managing them *[see Warnings and Precautions (5.1) and Clinical Pharmacology (12.3)]*.

Table 2: Drug Interactions that may Increase the Risk of Myopathy and Rhabdomyolysis with LIPITOR

**Cyclosporine or Gemfibrozil**	
Clinical Impact:	Atorvastatin plasma levels were significantly increased with concomitant administration of LIPITOR and cyclosporine, an inhibitor of CYP3A4 and OATP1B1 *[see Clinical Pharmacology (12.3)]*. Gemfibrozil may cause myopathy when given alone. The risk of myopathy and rhabdomyolysis is increased with concomitant use of cyclosporine or gemfibrozil with LIPITOR.
Intervention:	Concomitant use of cyclosporine or gemfibrozil with LIPITOR is not recommended.
**Anti-Viral Medications**	
Clinical Impact:	Atorvastatin plasma levels were significantly increased with concomitant administration of LIPITOR with many anti-viral medications, which are inhibitors of CYP3A4 and/or transporters (e.g., BCRP, OATP1B1/1B3, P-gp, MRP2, and/or OAT2) *[see Clinical Pharmacology (12.3)]*. Cases of myopathy and rhabdomyolysis have been reported with concomitant use of ledipasvir plus sofosbuvir with

	LIPITOR.
Intervention:	• Concomitant use of tipranavir plus ritonavir or glecaprevir plus pibrentasvir with LIPITOR is not recommended.<br>• In patients taking lopinavir plus ritonavir, or simeprevir, consider the risk/benefit of concomitant use with atorvastatin.<br>• In patients taking saquinavir plus ritonavir, darunavir plus ritonavir, fosamprenavir, fosamprenavir plus ritonavir, elbasvir plus grazoprevir or letermovir, do not exceed LIPITOR 20 mg.<br>• In patients taking nelfinavir, do not exceed LIPITOR 40 mg *[see Dosage and Administration (2.5)].*<br>• Consider the risk/benefit of concomitant use of ledipasvir plus sofosbuvir with LIPITOR.<br>• Monitor all patients for signs and symptoms of myopathy particularly during initiation of therapy and during upward dose titration of either drug.
Examples:	Tipranavir plus ritonavir, glecaprevir plus pibrentasvir, lopinavir plus ritonavir, simeprevir, saquinavir plus ritonavir, darunavir plus ritonavir, fosamprenavir, fosamprenavir plus ritonavir, elbasvir plus grazoprevir, letermovir, nelfinavir, and ledipasvir plus sofosbuvir.
**Select Azole Antifungals or Macrolide Antibiotics**	
Clinical Impact:	Atorvastatin plasma levels were significantly increased with concomitant administration of LIPITOR with select azole antifungals or macrolide antibiotics, due to inhibition of CYP3A4 and/or transporters *[see Clinical Pharmacology (12.3)]*.
Intervention:	In patients taking clarithromycin or itraconazole, do not exceed LIPITOR 20 mg *[see Dosage and Administration (2.5)]*. Consider the risk/benefit of concomitant use of other azole antifungals or macrolide antibiotics with LIPITOR. Monitor all patients for signs and symptoms of myopathy particularly during initiation of therapy and during upward dose titration of either drug.
Examples:	Erythromycin, clarithromycin, itraconazole, ketoconazole, posaconazole, and voriconazole.
**Niacin**	
Clinical Impact:	Cases of myopathy and rhabdomyolysis have been observed with concomitant use of lipid modifying dosages of niacin (≥1 gram/day niacin) with LIPITOR.
Intervention:	Consider if the benefit of using lipid modifying dosages of niacin concomitantly with LIPITOR outweighs the increased risk of myopathy and rhabdomyolysis. If concomitant use is decided, monitor patients for signs and symptoms of myopathy particularly during initiation of therapy and during upward dose titration of either drug.
**Fibrates (other than Gemfibrozil)**	
Clinical Impact:	Fibrates may cause myopathy when given alone. The risk of myopathy and rhabdomyolysis is increased with concomitant use of fibrates with LIPITOR.
	Consider if the benefit of using fibrates concomitantly with LIPITOR

Intervention:	outweighs the increased risk of myopathy and rhabdomyolysis. If concomitant use is decided, monitor patients for signs and symptoms of myopathy particularly during initiation of therapy and during upward dose titration of either drug.
**Colchicine**	
Clinical Impact:	Cases of myopathy and rhabdomyolysis have been reported with concomitant use of colchicine with LIPITOR.
Intervention:	Consider the risk/benefit of concomitant use of colchicine with LIPITOR. If concomitant use is decided, monitor patients for signs and symptoms of myopathy particularly during initiation of therapy and during upward dose titration of either drug.
**Grapefruit Juice**	
Clinical Impact:	Grapefruit juice consumption, especially excessive consumption, more than 1.2 liters/daily, can raise the plasma levels of atorvastatin and may increase the risk of myopathy and rhabdomyolysis.
Intervention:	Avoid intake of large quantities of grapefruit juice, more than 1.2 liters daily, when taking LIPITOR.

7.2 Drug Interactions that may Decrease Exposure to LIPITOR

Table 3 presents drug interactions that may decrease exposure to LIPITOR and instructions for preventing or managing them.

Table 3: Drug Interactions that may Decrease Exposure to LIPITOR

**Rifampin**	
Clinical Impact:	Concomitant administration of LIPITOR with rifampin, an inducer of cytochrome P450 3A4 and inhibitor of OATP1B1, can lead to variable reductions in plasma concentrations of atorvastatin. Due to the dual interaction mechanism of rifampin, delayed administration of LIPITOR after administration of rifampin has been associated with a significant reduction in atorvastatin plasma concentrations.
Intervention:	Administer LIPITOR and rifampin simultaneously.

7.3 LIPITOR Effects on Other Drugs

Table 4 presents LIPITOR’s effect on other drugs and instructions for preventing or managing them.

Table 4: LIPITOR Effects on Other Drugs

**Oral Contraceptives**	
Clinical Impact:	Co-administration of LIPITOR and an oral contraceptive increased plasma concentrations of norethindrone and ethinyl estradiol *[see Clinical Pharmacology (12.3)].*

Intervention:	Consider this when selecting an oral contraceptive for patients taking LIPITOR.
**Digoxin**	
Clinical Impact:	When multiple doses of LIPITOR and digoxin were co-administered, steady state plasma digoxin concentrations increased *[see Clinical Pharmacology (12.3)].*
Intervention:	Monitor patients taking digoxin appropriately.

8 USE IN SPECIFIC POPULATIONS

8.1 Pregnancy

Risk Summary

Discontinue LIPITOR when pregnancy is recognized. Alternatively, consider the ongoing therapeutic needs of the individual patient. LIPITOR decreases synthesis of cholesterol and possibly other biologically active substances derived from cholesterol; therefore, LIPITOR may cause fetal harm when administered to pregnant patients based on the mechanism of action *[see Clinical Pharmacology (12.1)]*. In addition, treatment of hyperlipidemia is not generally necessary during pregnancy. Atherosclerosis is a chronic process and the discontinuation of lipid-lowering drugs during pregnancy should have little impact on the outcome of long-term therapy of primary hyperlipidemia for most patients.

Available data from case series and prospective and retrospective observational cohort studies over decades of use with statins in pregnant women have not identified a drug-associated risk of major congenital malformations. Published data from prospective and retrospective observational cohort studies with LIPITOR use in pregnant women are insufficient to determine if there is a drug-associated risk of miscarriage *(see Data)*. In animal reproduction studies, no adverse developmental effects were observed in pregnant rats or rabbits orally administered atorvastatin at doses that resulted in up to 30 and 20 times, respectively, the human exposure at the maximum recommended human dose (MRHD) of 80 mg, based on body surface area (mg/m^2). In rats administered atorvastatin during gestation and lactation, decreased postnatal growth and development delay were observed at doses ≥ 6 times the MRHD *(see Data)*.

The estimated background risk of major birth defects and miscarriage for the indicated population is unknown. In the U.S. general population, the estimated background risk of major birth defects and miscarriage in clinically recognized pregnancies is 2-4% and 15-20%, respectively.

Data

Human Data

A Medicaid cohort linkage study of 1152 statin-exposed pregnant women compared to 886,996 controls did not find a significant teratogenic effect from maternal use of statins in the first trimester of pregnancy, after adjusting for potential confounders –

including maternal age, diabetes mellitus, hypertension, obesity, and alcohol and tobacco use – using propensity score-based methods. The relative risk of congenital malformations between the group with statin use and the group with no statin use in the first trimester was 1.07 (95% confidence interval 0.85 to 1.37) after controlling for confounders, particularly pre-existing diabetes mellitus. There were also no statistically significant increases in any of the organ-specific malformations assessed after accounting for confounders. In the majority of pregnancies, statin treatment was initiated prior to pregnancy and was discontinued at some point in the first trimester when pregnancy was identified. Study limitations include reliance on physician coding to define the presence of a malformation, lack of control for certain confounders such as body mass index, use of prescription dispensing as verification for the use of a statin, and lack of information on non-live births.

Animal Data

Atorvastatin was administered to pregnant rats and rabbits during organogenesis at oral doses up to 300 mg/kg/day and 100 mg/kg/day, respectively. Atorvastatin was not teratogenic in rats at doses up to 300 mg/kg/day or in rabbits at doses up to 100 mg/kg/day. These doses resulted in multiples of about 30 times (rat) or 20 times (rabbit) the human exposure at the MRHD based on surface area (mg/m2). In rats, the maternally toxic dose of 300 mg/kg resulted in increased post-implantation loss and decreased fetal body weight. At the maternally toxic doses of 50 and 100 mg/kg/day in rabbits, there was increased post-implantation loss, and at 100 mg/kg/day fetal body weights were decreased.

In a study in pregnant rats administered 20, 100, or 225 mg/kg/day from gestation day 7 through to lactation day 20 (weaning), there was decreased survival at birth, postnatal day 4, weaning, and post-weaning in pups of mothers dosed with 225 mg/kg/day, a dose at which maternal toxicity was observed. Pup body weight was decreased through postnatal day 21 at 100 mg/kg/day, and through postnatal day 91 at 225 mg/kg/day. Pup development was delayed (rotorod performance at 100 mg/kg/day and acoustic startle at 225 mg/kg/day; pinnae detachment and eye-opening at 225 mg/kg/day). These doses correspond to 6 times (100 mg/kg) and 22 times (225 mg/kg) the human exposure at the MRHD, based on AUC.

Atorvastatin crosses the rat placenta and reaches a level in fetal liver equivalent to that of maternal plasma.

8.2 Lactation

Risk Summary

There is no information about the presence of atorvastatin in human milk, the effects of the drug on the breastfed infant or the effects of the drug on milk production. However, it has been shown that another drug in this class passes into human milk. Studies in rats have shown that atorvastatin and/or its metabolites are present in the breast milk of lactating rats. When a drug is present in animal milk, it is likely that the drug will be present in human milk (see Data). Statins, including LIPITOR, decrease cholesterol synthesis and possibly the synthesis of other biologically active substances derived from cholesterol and may cause harm to the breastfed infant.

Because of the potential for serious adverse reactions in a breastfed infant, based on

the mechanism of action, advise patients that breastfeeding is not recommended during treatment with LIPITOR *[see Use in Specific Populations (8.1), Clinical Pharmacology (12.1)]*.

Data

Following a single oral administration of 10 mg/kg of radioactive atorvastatin to lactating rats, the concentration of total radioactivity was determined. Atorvastatin and/or its metabolites were measured in the breast milk and pup plasma at a 2:1 ratio (milk:plasma).

8.4 Pediatric Use

The safety and effectiveness of LIPITOR as an adjunct to diet to reduce LDL-C have been established pediatric patients 10 years of age and older with HeFH. Use of LIPITOR for this indication is based on a double-blind, placebo-controlled clinical trial in 187 pediatric patients 10 years of age and older with HeFH. In this limited controlled trial, there was no significant effect on growth or sexual maturation in the boys or girls, or on menstrual cycle length in girls.

The safety and effectiveness of LIPITOR as an adjunct to other LDL-C-lowering therapies to reduce LDL-C have been established pediatric patients 10 years of age and older with HoFH. Use of LIPITOR for this indication is based on a trial without a concurrent control group in 8 pediatric patients 10 years of age and older with HoFH *[see Clinical Studies (14)]*.

The safety and effectiveness of LIPITOR have not been established in pediatric patients younger than 10 years of age with HeFH or HoFH, or in pediatric patients with other types of hyperlipidemia (other than HeFH or HoFH).

8.5 Geriatric Use

Of the total number of LIPITOR-treated patients in clinical trials, 15,813 (40%) were ≥65 years old and 2,800 (7%) were ≥75 years old. No overall differences in safety or effectiveness were observed between these patients and younger patients.

Advanced age (≥65 years) is a risk factor for LIPITOR-associated myopathy and rhabdomyolysis. Dose selection for an elderly patient should be cautious, recognizing the greater frequency of decreased hepatic, renal, or cardiac function, and of concomitant disease or other drug therapy and the higher risk of myopathy. Monitor geriatric patients receiving LIPITOR for the increased risk of myopathy *[see Warnings and Precautions (5.1) and Clinical Pharmacology (12.3)]*.

8.6 Renal Impairment

Renal impairment is a risk factor for myopathy and rhabdomyolysis. Monitor all patients with renal impairment for development of myopathy. Renal impairment does not affect the plasma concentrations of LIPITOR, therefore there is no dosage adjustment in patients with renal impairment *[see Warnings and Precautions (5.1) and Clinical Pharmacology (12.3)]*.

8.7 Hepatic Impairment

In patients with chronic alcoholic liver disease, plasma concentrations of LIPITOR are

markedly increased. Cmax and AUC are each 4-fold greater in patients with Childs-Pugh A disease. Cmax and AUC are approximately 16-fold and 11-fold increased, respectively, in patients with Childs-Pugh B disease. LIPITOR is contraindicated in patients with acute liver failure or decompensated cirrhosis *[see Contraindications (4)]*.

10 OVERDOSAGE

No specific antidotes for LIPITOR are known. Contact Poison Control (1-800-222-1222) for latest recommendations. Due to extensive drug binding to plasma proteins, hemodialysis is not expected to significantly enhance LIPITOR clearance.

11 DESCRIPTION

LIPITOR (atorvastatin) is an inhibitor of 3-hydroxy-3-methylglutaryl-coenzyme A (HMG-CoA) reductase.

Atorvastatin calcium is [R-(R*, R*)]-2-(4-fluorophenyl)-ß, δ-dihydroxy-5-(1-methylethyl)-3-phenyl-4-[(phenylamino)carbonyl]-1H-pyrrole-1-heptanoic acid, calcium salt (2:1) trihydrate. The empirical formula of atorvastatin calcium is $(C_{33}H_{34}FN_2O_5)_2Ca \bullet 3H_2O$ and its molecular weight is 1209.42. Its structural formula is:

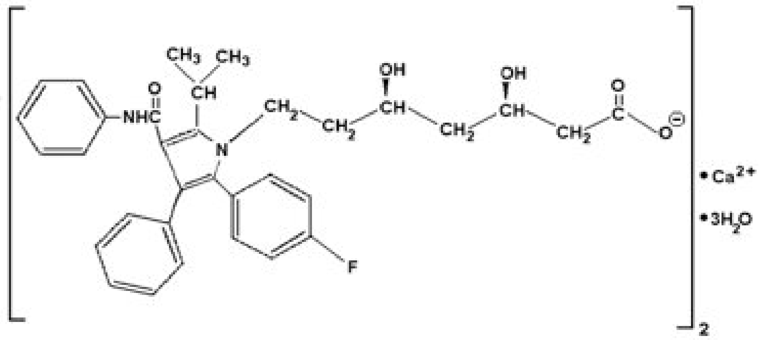

Atorvastatin calcium is a white to off-white crystalline powder that is insoluble in aqueous solutions of pH 4 and below. Atorvastatin calcium is very slightly soluble in distilled water, pH 7.4 phosphate buffer, and acetonitrile; slightly soluble in ethanol; and freely soluble in methanol.

LIPITOR tablets for oral use contain atorvastatin 10 mg, 20 mg, 40 mg, or 80 mg (equivalent to 10.36 mg, 20.72 mg, 41.44 mg, or 82.88 mg atorvastatin calcium anhydrous) and the following inactive ingredients: calcium carbonate, USP; candelilla wax, FCC; croscarmellose sodium, NF; hydroxypropyl cellulose, NF; lactose monohydrate, NF; magnesium stearate, NF; microcrystalline cellulose, NF; Opadry White YS-1-7040 (hypromellose, polyethylene glycol, talc, titanium dioxide); polysorbate 80, NF; simethicone emulsion.

12 CLINICAL PHARMACOLOGY

12.1 Mechanism of Action

LIPITOR is a selective, competitive inhibitor of HMG-CoA reductase, the rate-limiting enzyme that converts 3-hydroxy-3-methylglutaryl-coenzyme A to mevalonate, a precursor of sterols, including cholesterol. In animal models, LIPITOR lowers plasma cholesterol and lipoprotein levels by inhibiting HMG-CoA reductase and cholesterol synthesis in the liver and by increasing the number of hepatic LDL receptors on the cell surface to enhance uptake and catabolism of LDL; LIPITOR also reduces LDL production and the number of LDL particles.

12.2 Pharmacodynamics

LIPITOR, as well as some of its metabolites, are pharmacologically active in humans. The liver is the primary site of action and the principal site of cholesterol synthesis and LDL clearance. Drug dosage, rather than systemic drug concentration, correlates better with LDL-C reduction. Individualization of drug dosage should be based on therapeutic response *[see Dosage and Administration (2)]*.

12.3 Pharmacokinetics

<u>Absorption</u>

LIPITOR is rapidly absorbed after oral administration; maximum plasma concentrations occur within 1 to 2 hours. Extent of absorption increases in proportion to LIPITOR dose. The absolute bioavailability of atorvastatin (parent drug) is approximately 14% and the systemic availability of HMG-CoA reductase inhibitory activity is approximately 30%. The low systemic availability is attributed to presystemic clearance in gastrointestinal mucosa and/or hepatic first-pass metabolism. Although food decreases the rate and extent of drug absorption by approximately 25% and 9%, respectively, as assessed by Cmax and AUC, LDL-C reduction is similar whether LIPITOR is given with or without food. Plasma LIPITOR concentrations are lower (approximately 30% for Cmax and AUC) following evening drug administration compared with morning. However, LDL-C reduction is the same regardless of the time of day of drug administration.

<u>Distribution</u>

Mean volume of distribution of LIPITOR is approximately 381 liters. LIPITOR is ≥98% bound to plasma proteins. A blood/plasma ratio of approximately 0.25 indicates poor drug penetration into red blood cells.

<u>Elimination</u>

Metabolism

LIPITOR is extensively metabolized to ortho- and parahydroxylated derivatives and various beta-oxidation products. *In vitro* inhibition of HMG-CoA reductase by ortho- and parahydroxylated metabolites is equivalent to that of LIPITOR. Approximately 70% of circulating inhibitory activity for HMG-CoA reductase is attributed to active metabolites. *In vitro* studies suggest the importance of LIPITOR metabolism by cytochrome P450 3A4, consistent with increased plasma concentrations of LIPITOR in humans following co-administration with erythromycin, a known inhibitor of this isozyme *[see Drug Interactions (7.1)]*. In animals, the ortho-hydroxy metabolite undergoes further glucuronidation.

Excretion

LIPITOR and its metabolites are eliminated primarily in bile following hepatic and/or extra-hepatic metabolism; however, the drug does not appear to undergo enterohepatic recirculation. Mean plasma elimination half-life of LIPITOR in humans is approximately 14 hours, but the half-life of inhibitory activity for HMG-CoA reductase is 20 to 30 hours due to the contribution of active metabolites. Less than 2% of a dose of LIPITOR is recovered in urine following oral administration.

Specific Populations

Geriatric

Plasma concentrations of LIPITOR are higher (approximately 40% for Cmax and 30% for AUC) in healthy elderly subjects (age ≥65 years) than in young adults.

Pediatric

Apparent oral clearance of atorvastatin in pediatric subjects appeared similar to that of adults when scaled allometrically by body weight as the body weight was the only significant covariate in atorvastatin population PK model with data including pediatric HeFH patients (ages 10 years to 17 years of age, n=29) in an open-label, 8-week study.

Gender

Plasma concentrations of LIPITOR in women differ from those in men (approximately 20% higher for Cmax and 10% lower for AUC); however, there is no clinically significant difference in LDL-C reduction with LIPITOR between men and women.

Renal Impairment

Renal disease has no influence on the plasma concentrations or LDL-C reduction of LIPITOR *[see Use in Specific Populations (8.6)]*.

While studies have not been conducted in patients with end-stage renal disease, hemodialysis is not expected to significantly enhance clearance of LIPITOR since the drug is extensively bound to plasma proteins.

Hepatic Impairment

In patients with chronic alcoholic liver disease, plasma concentrations of LIPITOR are markedly increased. Cmax and AUC are each 4-fold greater in patients with Childs-Pugh A disease. Cmax and AUC are approximately 16-fold and 11-fold increased, respectively, in patients with Childs-Pugh B disease *[see Use in Specific Populations (8.7)]*.

Drug Interactions

Atorvastatin is a substrate of the hepatic transporters, OATP1B1 and OATP1B3 transporter. Metabolites of atorvastatin are substrates of OATP1B1. Atorvastatin is also identified as a substrate of the efflux transporter BCRP, which may limit the intestinal absorption and biliary clearance of atorvastatin.

Table 5: Effect of Co-administered Drugs on the Pharmacokinetics of

Atorvastatin

Co-administered drug and dosing regimen	Atorvastatin		
	Dose (mg)	Ratio of AUC*	Ratio of Cmax*
†Cyclosporine 5.2 mg/kg/day, stable dose	10 mg QD‡ for 28 days	8.69	10.66
†Tipranavir 500 mg BID§/ritonavir 200 mg BID§, 7 days	10 mg SD¶	9.36	8.58
†Glecaprevir 400 mg QD‡/pibrentasvir 120 mg QD‡, 7 days	10 mg QD‡ for 7 days	8.28	22.00
†Telaprevir 750 mg q8h#, 10 days	20 mg SD¶	7.88	10.60
†, ÞSaquinavir 400 mg BID§/ritonavir 400 mg BID§, 15 days	40 mg QD‡ for 4 days	3.93	4.31
†Elbasvir 50 mg QD‡/grazoprevir 200 mg QD‡, 13 days	10 mg SD¶	1.94	4.34
†Simeprevir 150 mg QD‡, 10 days	40 mg SD¶	2.12	1.70
†Clarithromycin 500 mg BID§, 9 days	80 mg QD‡ for 8 days	4.54	5.38
†Darunavir 300 mg BID§/ritonavir 100 mg BID§, 9 days	10 mg QD‡ for 4 days	3.45	2.25
†Itraconazole 200 mg QD‡, 4 days	40 mg SD¶	3.32	1.20
†Letermovir 480 mg QD‡, 10 days	20 mg SD¶	3.29	2.17
†Fosamprenavir 700 mg BID§/ritonavir 100 mg BID§, 14 days	10 mg QD‡ for 4 days	2.53	2.84
†Fosamprenavir 1400 mg BID§, 14 days	10 mg QD‡ for 4 days	2.30	4.04
†Nelfinavir 1250 mg BID§, 14 days	10 mg QD‡ for 28 days	1.74	2.22
†Grapefruit Juice, 240 mL QD‡,ß	40 mg SD¶	1.37	1.16
Diltiazem 240 mg QD‡, 28 days	40 mg SD¶	1.51	1.00
Erythromycin 500 mg QIDà, 7 days	10 mg SD¶	1.33	1.38
Amlodipine 10 mg, single dose	80 mg SD¶	1.18	0.91
Cimetidine 300 mg QIDà, 2 weeks	10 mg QD‡for 2 weeks	1.00	0.89
Colestipol 10 g BID§, 24 weeks	40 mg QD‡ for 8 weeks	NA	0.74è
Maalox TC® 30 mL QIDà, 17 days	10 mg QD‡ for 15 days	0.66	0.67
Efavirenz 600 mg QD‡, 14 days	10 mg for 3 days	0.59	1.01
†Rifampin 600 mg QD‡, 7 days (co-administered)ð	40 mg SD¶	1.12	2.90
†Rifampin 600 mg QD‡, 5 days (doses separated)ð	40 mg SD¶	0.20	0.60
†Gemfibrozil 600 mg BID§, 7 days	40 mg SD¶	1.35	1.00
†Fenofibrate 160 mg QD‡, 7 days	40 mg SD¶	1.03	1.02

[illegible]	[illegible]		
Boceprevir 800 mg TID[ø], 7 days	40 mg SD[¶]	2.32	2.66

* Represents ratio of treatments (co-administered drug plus atorvastatin vs. atorvastatin alone).
† See Sections 5.1 and 7 for clinical significance.
‡ Once daily
§ Twice daily
¶ Single dose
\# Every 8 hours
Þ The dose of saquinavir plus ritonavir in this study is not the clinically used dose. The increase in atorvastatin exposure when used clinically is likely to be higher than what was observed in this study. Therefore, caution should be applied and the lowest dose necessary should be used.
ß Greater increases in AUC (ratio of AUC up to 2.5) and/or Cmax (ratio of Cmax up to 1.71) have been reported with excessive grapefruit consumption (≥ 750 mL-1.2 liters per day).
à Four times daily
è Ratio based on a single sample taken 8-16 h post dose.
ð Due to the dual interaction mechanism of rifampin, simultaneous co-administration of atorvastatin with rifampin is recommended, as delayed administration of atorvastatin after administration of rifampin has been associated with a significant reduction in atorvastatin plasma concentrations.
ø Three times daily

Table 6: Effect of Atorvastatin on the Pharmacokinetics of Co-administered Drugs

Atorvastatin	Co-administered drug and dosing regimen		
	**Drug/Dose (mg)**	**Ratio of AUC**	**Ratio of Cmax**
80 mg QD[*] for 15 days	Antipyrine, 600 mg SD[†]	1.03	0.89
80 mg QD[*] for 10 days	[‡]Digoxin 0.25 mg QD[*], 20 days	1.15	1.20
40 mg QD[*] for 22 days	Oral contraceptive QD[*], 2 months<br>- norethindrone 1 mg<br>- ethinyl estradiol 35 µg	 1.28<br>1.19	 1.23<br>1.30
10 mg SD[†]	Tipranavir 500 mg BID[§]/ritonavir 200 mg BID[§], 7 days	1.08	0.96
10 mg QD[*] for 4 days	Fosamprenavir 1400 mg BID[§], 14 days	0.73	0.82
10 mg QD[*] for 4 days	Fosamprenavir 700 mg BID[§]/ritonavir 100 mg BID[§], 14 days	0.99	0.94

* Once daily
† Single dose
‡ See Section 7 for clinical significance.
§ Twice daily

LIPITOR had no clinically significant effect on prothrombin time when administered to patients receiving chronic warfarin treatment.

13 NONCLINICAL TOXICOLOGY

13.1 Carcinogenesis, Mutagenesis, Impairment of Fertility

In a 2-year carcinogenicity study in rats at dose levels of 10, 30, and 100 mg/kg/day, 2 rare tumors were found in muscle in high-dose females: in one, there was a rhabdomyosarcoma and, in another, there was a fibrosarcoma. This dose represents a plasma AUC (0-24) value of approximately 16 times the mean human plasma drug exposure after an 80 mg oral dose.

A 2-year carcinogenicity study in mice given 100, 200, or 400 mg/kg/day resulted in a significant increase in liver adenomas in high-dose males and liver carcinomas in high-dose females. These findings occurred at plasma AUC (0-24) values of approximately 6 times the mean human plasma drug exposure after an 80 mg oral dose.

In vitro, atorvastatin was not mutagenic or clastogenic in the following tests with and without metabolic activation: the Ames test with *Salmonella typhimurium* and *Escherichia coli*, the HGPRT forward mutation assay in Chinese hamster lung cells, and the chromosomal aberration assay in Chinese hamster lung cells. Atorvastatin was negative in the *in vivo* mouse micronucleus test.

In female rats, atorvastatin at doses up to 225 mg/kg (56 times the human exposure) did not cause adverse effects on fertility. Studies in male rats performed at doses up to 175 mg/kg (15 times the human exposure) produced no changes in fertility. There was aplasia and aspermia in the epididymis of 2 of 10 rats treated with 100 mg/kg/day of atorvastatin for 3 months (16 times the human AUC at the 80 mg dose); testis weights were significantly lower at 30 and 100 mg/kg and epididymal weight was lower at 100 mg/kg. Male rats given 100 mg/kg/day for 11 weeks prior to mating had decreased sperm motility, spermatid head concentration, and increased abnormal sperm. Atorvastatin caused no adverse effects on semen parameters, or reproductive organ histopathology in dogs given doses of 10, 40, or 120 mg/kg for 2 years.

14 CLINICAL STUDIES

<u>Prevention of Cardiovascular Disease</u>

In the Anglo-Scandinavian Cardiac Outcomes Trial (ASCOT), the effect of LIPITOR on fatal and non-fatal coronary heart disease was assessed in 10,305 patients with hypertension, 40-80 years of age (mean of 63 years; 19% women; 95% White, 3% Black, 1% South Asian, 1% other), without a previous myocardial infarction and with total cholesterol (TC) levels ≤251 mg/dL. Additionally, all patients had at least 3 of the following cardiovascular risk factors: male gender (81%), age >55 years (85%), smoking (33%), diabetes (24%), history of CHD in a first-degree relative (26%), TC:HDL >6 (14%), peripheral vascular disease (5%), left ventricular hypertrophy (14%), prior cerebrovascular event (10%), specific ECG abnormality (14%), proteinuria/albuminuria (62%). In this double-blind, placebo-controlled trial, patients were treated with anti-hypertensive therapy (goal BP <140/90 mm Hg for patients without diabetes; <130/80 mm Hg for patients with diabetes) and allocated to either LIPITOR 10 mg daily (n=5168) or placebo (n=5137), using a covariate adaptive method which took into account the distribution of nine baseline characteristics of patients already enrolled and minimized the imbalance of those characteristics across the groups. Patients were followed for a median duration of 3.3 years.

The effect of 10 mg/day of LIPITOR on lipid levels was similar to that seen in previous clinical trials.

LIPITOR significantly reduced the rate of coronary events [either fatal coronary heart disease (46 events in the placebo group vs. 40 events in the LIPITOR group) or non-fatal MI (108 events in the placebo group vs. 60 events in the LIPITOR group)] with a relative risk reduction of 36% [(based on incidences of 1.9% for LIPITOR vs. 3.0% for placebo), p=0.0005 (see Figure 1)]. The risk reduction was consistent regardless of age, smoking status, obesity, or presence of renal dysfunction. The effect of LIPITOR was seen regardless of baseline LDL levels.

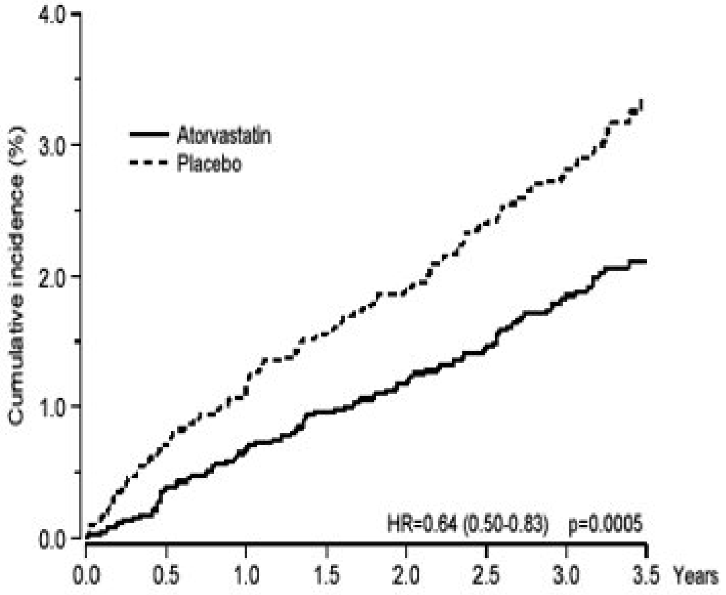

Figure 1: Effect of LIPITOR 10 mg/day on Cumulative Incidence of Non-Fatal Myocardial Infarction or Coronary Heart Disease Death (in ASCOT-LLA)

LIPITOR also significantly decreased the relative risk for revascularization procedures by 42% (incidences of 1.4% for LIPITOR and 2.5% for placebo). Although the reduction of fatal and non-fatal strokes did not reach a pre-defined significance level (p=0.01), a favorable trend was observed with a 26% relative risk reduction (incidences of 1.7% for LIPITOR and 2.3% for placebo). There was no significant difference between the treatment groups for death due to cardiovascular causes (p=0.51) or noncardiovascular causes (p=0.17).

In the Collaborative Atorvastatin Diabetes Study (CARDS), the effect of LIPITOR on cardiovascular disease (CVD) endpoints was assessed in 2838 subjects (94% White, 2% Black, 2% South Asian, 1% other; 68% male), ages 40-75 with type 2 diabetes based on WHO criteria, without prior history of cardiovascular disease and with LDL ≤160 mg/dL and triglycerides (TG) ≤600 mg/dL. In addition to diabetes, subjects had 1 or more of the following risk factors: current smoking (23%), hypertension (80%), retinopathy (30%), or microalbuminuria (9%) or macroalbuminuria (3%). No subjects on hemodialysis were enrolled in the trial. In this multicenter, placebo-controlled, double-blind clinical trial, subjects were randomly allocated to either LIPITOR 10 mg daily (1429) or placebo (1411) in a 1:1 ratio and were followed for a median duration of 3.9 years. The primary endpoint was the occurrence of any of the major cardiovascular events: myocardial infarction, acute CHD death, unstable angina, coronary revascularization, or

stroke. The primary analysis was the time to first occurrence of the primary endpoint.

Baseline characteristics of subjects were: mean age of 62 years, mean HbA1c 7.7%; median LDL-C 120 mg/dL; median TC 207 mg/dL; median TG 151 mg/dL; median HDL-C 52 mg/dL.

The effect of LIPITOR 10 mg/day on lipid levels was similar to that seen in previous clinical trials.

LIPITOR significantly reduced the rate of major cardiovascular events (primary endpoint events) (83 events in the LIPITOR group vs. 127 events in the placebo group) with a relative risk reduction of 37%, HR 0.63, 95% CI (0.48, 0.83) (p=0.001) (see Figure 2). An effect of LIPITOR was seen regardless of age, sex, or baseline lipid levels.

LIPITOR significantly reduced the risk of stroke by 48% (21 events in the LIPITOR group vs. 39 events in the placebo group), HR 0.52, 95% CI (0.31, 0.89) (p=0.016) and reduced the risk of MI by 42% (38 events in the LIPITOR group vs. 64 events in the placebo group), HR 0.58, 95.1% CI (0.39, 0.86) (p=0.007). There was no significant difference between the treatment groups for angina, revascularization procedures, and acute CHD death.

There were 61 deaths in the LIPITOR group vs. 82 deaths in the placebo group (HR 0.73, p=0.059).

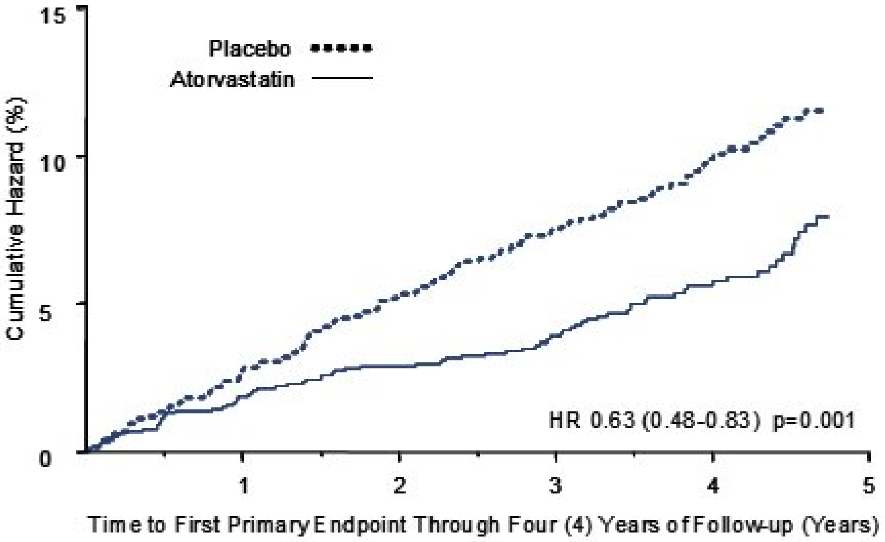

Figure 2: Effect of LIPITOR 10 mg/day on Time to Occurrence of Major Cardiovascular Event (myocardial infarction, acute CHD death, unstable angina, coronary revascularization, or stroke) in CARDS

In the Treating to New Targets Study (TNT), the effect of LIPITOR 80 mg/day vs. LIPITOR 10 mg/day on the reduction in cardiovascular events was assessed in 10,001 subjects (94% White, 81% male, 38% ≥65 years) with clinically evident coronary heart disease who had achieved a target LDL-C level <130 mg/dL after completing an 8-week, open-label, run-in period with LIPITOR 10 mg/day. Subjects were randomly assigned to either 10 mg/day or 80 mg/day of LIPITOR and followed for a median duration of 4.9 years. The primary endpoint was the time-to-first occurrence of any of the following major cardiovascular events (MCVE): death due to CHD, non-fatal myocardial infarction,

resuscitated cardiac arrest, and fatal and non-fatal stroke. The mean LDL-C, TC, TG, non-HDL, and HDL cholesterol levels at 12 weeks were 73, 145, 128, 98, and 47 mg/dL during treatment with 80 mg of LIPITOR and 99, 177, 152, 129, and 48 mg/dL during treatment with 10 mg of LIPITOR.

Treatment with LIPITOR 80 mg/day significantly reduced the rate of MCVE (434 events in the 80 mg/day group vs. 548 events in the 10 mg/day group) with a relative risk reduction of 22%, HR 0.78, 95% CI (0.69, 0.89), p=0.0002 (see Figure 3 and Table 7). The overall risk reduction was consistent regardless of age (<65, ≥65) or sex.

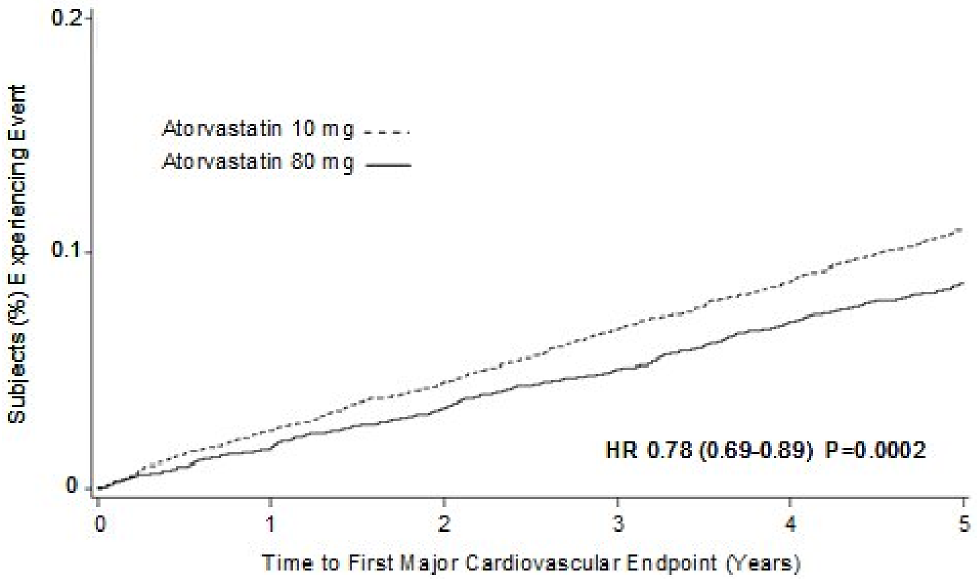

Figure 3: Effect of LIPITOR 80 mg/day vs. 10 mg/day on Time to Occurrence of Major Cardiovascular Events (TNT)

Table 7: Overview of Efficacy Results in TNT

**Endpoint**	**Atorvastatin 10 mg (N=5006)**		**Atorvastatin 80 mg (N=4995)**		**HR* (95%CI)**
**PRIMARY ENDPOINT**	n	(%)	n	(%)	
First major cardiovascular endpoint	548	(10.9)	434	(8.7)	0.78 (0.69, 0.89)
**Components of the Primary Endpoint**					
CHD death	127	(2.5)	101	(2.0)	0.80 (0.61, 1.03)
Non-fatal, non-procedure related MI	308	(6.2)	243	(4.9)	0.78 (0.66, 0.93)
Resuscitated cardiac arrest	26	(0.5)	25	(0.5)	0.96 (0.56, 1.67)
Stroke (fatal and non-fatal)	155	(3.1)	117	(2.3)	0.75 (0.59, 0.96)
**SECONDARY ENDPOINTS†**					
First CHF with hospitalization	164	(3.3)	122	(2.4)	0.74 (0.59, 0.94)
First PVD endpoint	282	(5.6)	275	(5.5)	0.97 (0.83, 1.15)

First CABG or other coronary revascularization procedure‡	904	(18.1)	667	(13.4)	0.72 (0.65, 0.80)
First documented angina endpoint‡	615	(12.3)	545	(10.9)	0.88 (0.79, 0.99)
All-cause mortality	282	(5.6)	284	(5.7)	1.01 (0.85, 1.19)
**Components of All-Cause Mortality**					
Cardiovascular death	155	(3.1)	126	(2.5)	0.81 (0.64, 1.03)
Noncardiovascular death	127	(2.5)	158	(3.2)	1.25 (0.99, 1.57)
Cancer death	75	(1.5)	85	(1.7)	1.13 (0.83, 1.55)
Other non-CV death	43	(0.9)	58	(1.2)	1.35 (0.91, 2.00)
Suicide, homicide, and other traumatic non-CV death	9	(0.2)	15	(0.3)	1.67 (0.73, 3.82)

* Atorvastatin 80 mg: atorvastatin 10 mg
† Secondary endpoints not included in primary endpoint
‡ Component of other secondary endpoints

HR=hazard ratio; CHD=coronary heart disease; CI=confidence interval; MI=myocardial infarction; CHF=congestive heart failure; CV=cardiovascular; PVD=peripheral vascular disease; CABG=coronary artery bypass graft

Confidence intervals for the Secondary Endpoints were not adjusted for multiple comparisons

Of the events that comprised the primary efficacy endpoint, treatment with LIPITOR 80 mg/day significantly reduced the rate of non-fatal, non-procedure related MI and fatal and non-fatal stroke, but not CHD death or resuscitated cardiac arrest (Table 7). Of the predefined secondary endpoints, treatment with LIPITOR 80 mg/day significantly reduced the rate of coronary revascularization, angina, and hospitalization for heart failure, but not peripheral vascular disease. The reduction in the rate of CHF with hospitalization was only observed in the 8% of patients with a prior history of CHF.

There was no significant difference between the treatment groups for all-cause mortality (Table 7). The proportions of subjects who experienced cardiovascular death, including the components of CHD death and fatal stroke, were numerically smaller in the LIPITOR 80 mg group than in the LIPITOR 10 mg treatment group. The proportions of subjects who experienced noncardiovascular death were numerically larger in the LIPITOR 80 mg group than in the LIPITOR 10 mg treatment group.

Primary Hyperlipidemia in Adults

LIPITOR reduces total-C, LDL-C, apo B, and TG, and increases HDL-C in patients with hyperlipidemia (heterozygous familial and nonfamilial) and mixed dyslipidemia. Therapeutic response is seen within 2 weeks, and maximum response is usually achieved within 4 weeks and maintained during chronic therapy.

In two multicenter, placebo-controlled, dose-response trials in patients with hyperlipidemia, LIPITOR given as a single dose over 6 weeks, significantly reduced total-C, LDL-C, apo B, and TG. (Pooled results are provided in Table 8.)

Table 8: Dose Response in Patients with Primary Hyperlipidemia (Adjusted

Mean % Change From Baseline)*

Dose	N	TC	LDL-C	Apo B	TG	HDL-C
Placebo	21	4	4	3	10	-3
10	22	-29	-39	-32	-19	6
20	20	-33	-43	-35	-26	9
40	21	-37	-50	-42	-29	6
80	23	-45	-60	-50	-37	5

* Results are pooled from 2 dose response trials.

In three multicenter, double-blind trials in patients with hyperlipidemia, LIPITOR was compared to other statins. After randomization, patients were treated for 16 weeks with either LIPITOR 10 mg per day or a fixed dose of the comparative agent (Table 9).

Table 9: Mean Percentage Change From Baseline at Endpoint (Double-Blind, Randomized, Active-Controlled Trials)

**Treatment** (Daily Dose)	N	Total-C	LDL-C	Apo B	TG	HDL-C
Trial 1						
LIPITOR 10 mg	707	-27*	-36*	-28*	-17*	+7
Lovastatin 20 mg	191	-19	-27	-20	-6	+7
95% CI for Diff†		-9.2, -6.5	-10.7, -7.1	-10.0, -6.5	-15.2, -7.1	-1.7, 2.0
Trial 2						
LIPITOR 10 mg	222	-25‡	-35‡	-27‡	-17‡	+6
Pravastatin 20 mg	77	-17	-23	-17	-9	+8
95% CI for Diff†		-10.8, -6.1	-14.5, -8.2	-13.4, -7.4	-14.1, -0.7	-4.9, 1.6
Trial 3						
LIPITOR 10 mg	132	-29§	-37§	-34§	-23§	+7
Simvastatin 10 mg	45	-24	-30	-30	-15	+7
95% CI for Diff†		-8.7, -2.7	-10.1, -2.6	-8.0, -1.1	-15.1, -0.7	-4.3, 3.9

* Significantly different from lovastatin, ANCOVA, $p \leq 0.05$
† A negative value for the 95% CI for the difference between treatments favors LIPITOR for all except HDL-C, for which a positive value favors LIPITOR. If the range does not include 0, this indicates a statistically significant difference.
‡ Significantly different from pravastatin, ANCOVA, $p \leq 0.05$
§ Significantly different from simvastatin, ANCOVA, $p \leq 0.05$

Table 9 does not contain data comparing the effects of LIPITOR 10 mg and higher doses of lovastatin, pravastatin, and simvastatin. The drugs compared in the trials summarized in the table are not necessarily interchangeable.

<u>Hypertriglyceridemia in Adults</u>

The response to LIPITOR in 64 patients with isolated hypertriglyceridemia treated across several clinical trials is shown in the table below (Table 10). For the LIPITOR-treated patients, median (min, max) baseline TG level was 565 (267-1502).

Table 10: Combined Patients with Isolated Elevated TG: Median (min, max)

Percentage Change From Baseline

	Placebo (N=12)	LIPITOR 10 mg (N=37)	LIPITOR 20 mg (N=13)	LIPITOR 80 mg (N=14)
Triglycerides	-12.4 (-36.6, 82.7)	-41.0 (-76.2, 49.4)	-38.7 (-62.7, 29.5)	-51.8 (-82.8, 41.3)
Total-C	-2.3 (-15.5, 24.4)	-28.2 (-44.9, -6.8)	-34.9 (-49.6, -15.2)	-44.4 (-63.5, -3.8)
LDL-C	3.6 (-31.3, 31.6)	-26.5 (-57.7, 9.8)	-30.4 (-53.9, 0.3)	-40.5 (-60.6, -13.8)
HDL-C	3.8 (-18.6, 13.4)	13.8 (-9.7, 61.5)	11.0 (-3.2, 25.2)	7.5 (-10.8, 37.2)
non-HDL-C	-2.8 (-17.6, 30.0)	-33.0 (-52.1, -13.3)	-42.7 (-53.7, -17.4)	-51.5 (-72.9, -4.3)

Dysbetalipoproteinemia in Adults

The results of an open-label crossover trial of 16 patients (genotypes: 14 apo E2/E2 and 2 apo E3/E2) with dysbetalipoproteinemia are shown in the table below (Table 11).

Table 11: Open-Label Crossover Trial of 16 Patients with Dysbetalipoproteinemia (Fredrickson Type III)

		Median % Change (min, max)	
	Median (min, max) at Baseline (mg/dL)	LIPITOR 10 mg	LIPITOR 80 mg
Total-C	442 (225, 1320)	-37 (-85, 17)	-58 (-90, -31)
Triglycerides	678 (273, 5990)	-39 (-92, -8)	-53 (-95, -30)
IDL-C + VLDL-C	215 (111, 613)	-32 (-76, 9)	-63 (-90, -8)
non-HDL-C	411 (218, 1272)	-43 (-87, -19)	-64 (-92, -36)

HoFH in Adults and Pediatric Patients

In a trial without a concurrent control group, 29 patients (mean age of 22 years, median age of 24 years, 31% <18 years) with HoFH received maximum daily doses of 20 to 80 mg of LIPITOR. The mean LDL-C reduction in this trial was 18%. Twenty-five patients with a reduction in LDL-C had a mean response of 20% (range of 7% to 53%, median of 24%); the remaining 4 patients had 7% to 24% increases in LDL-C. Five of the 29 patients had absent LDL-receptor function. Of these, 2 patients also had a portacaval shunt and had no significant reduction in LDL-C. The remaining 3 receptor-negative patients had a mean LDL-C reduction of 22%.

HeFH in Pediatric Patients

In a double-blind, placebo-controlled trial followed by an open-label phase, 187 boys and post-menarchal girls 10 years to 17 years of age (mean age 14.1 years; 31% female; 92% White, 1.6% Blacks, 1.6% Asians, 4.8% other) with heterozygous familial hypercholesterolemia (HeFH) or severe hypercholesterolemia, were randomized to

LIPITOR (n=140) or placebo (n=47) for 26 weeks and then all received LIPITOR for 26 weeks. Inclusion in the trial required 1) a baseline LDL-C level ≥ 190 mg/dL or 2) a baseline LDL-C level ≥ 160 mg/dL and positive family history of FH or documented premature cardiovascular disease in a first or second-degree relative. The mean baseline LDL-C value was 219 mg/dL (range: 139-385 mg/dL) in the LIPITOR group compared to 230 mg/dL (range: 160-325 mg/dL) in the placebo group. The dosage of LIPITOR (once daily) was 10 mg for the first 4 weeks and uptitrated to 20 mg if the LDL-C level was >130 mg/dL. The number of LIPITOR-treated patients who required uptitration to 20 mg after Week 4 during the double-blind phase was 78 (56%).

LIPITOR significantly decreased plasma levels of total-C, LDL-C, triglycerides, and apolipoprotein B during the 26-week double-blind phase (see Table 12).

Table 12: Lipid-altering Effects of LIPITOR in Adolescent Boys and Girls with Heterozygous Familial Hypercholesterolemia or Severe Hypercholesterolemia (Mean Percentage Change From Baseline at Endpoint in Intention-to-Treat Population)

DOSAGE	N	Total-C	LDL-C	HDL-C	TG	Apolipoprotein B
Placebo	47	-1.5	-0.4	-1.9	1.0	0.7
LIPITOR	140	-31.4	-39.6	2.8	-12.0	-34.0

The mean achieved LDL-C value was 130.7 mg/dL (range: 70.0-242.0 mg/dL) in the LIPITOR group compared to 228.5 mg/dL (range: 152.0-385.0 mg/dL) in the placebo group during the 26-week double-blind phase.

Atorvastatin was also studied in a three year open-label, uncontrolled trial that included 163 patients with HeFH who were 10 years to 15 years old (82 boys and 81 girls). All patients had a clinical diagnosis of HeFH confirmed by genetic analysis (if not already confirmed by family history). Approximately 98% were White, and less than 1% were Black or Asian. Mean LDL-C at baseline was 232 mg/dL. The starting atorvastatin dosage was 10 mg once daily and doses were adjusted to achieve a target of <130 mg/dL LDL-C. The reductions in LDL-C from baseline were generally consistent across age groups within the trial as well as with previous clinical trials in both adult and pediatric placebo-controlled trials.

16 HOW SUPPLIED/STORAGE AND HANDLING

LIPITOR tablets are supplied as follows:

**Strength**	**How Supplied**	**NDC**	**Tablet Description**
10 mg of atorvastatin	bottles of 90	0071-0155-23	white elliptical, film-coated tablets with “PD 155” on one side and “10” on the other
	10 x 10 unit dose blisters	0071-0155-40	
	bottles of 90	0071-0156-	white elliptical, film-coated tablets

20 mg of atorvastatin	bottles of 90	[illegible] 23	white elliptical, film-coated tablets with “PD 156” on one side and “20” on the other
	10 x 10 unit dose blisters	0071-0156-40	
40 mg of atorvastatin	bottles of 90	0071-0157-23	white elliptical, film-coated tablets with “PD 157” on one side and “40” on the other
	10 x 10 unit dose blisters	0071-0157-40	
80 mg of atorvastatin	bottles of 90	0071-0158-23	white elliptical, film-coated tablets with “PD 158” on one side and “80” on the other
	8 x 8 unit dose blisters	0071-0158-92	

Storage

Store at controlled room temperature 20 - 25°C (68 - 77°F).

17 PATIENT COUNSELING INFORMATION

Advise the patient to read the FDA-approved patient labeling (Patient Information).

Myopathy and Rhabdomyolysis

Advise patients that LIPITOR may cause myopathy and rhabdomyolysis. Inform patients that the risk is also increased when taking certain types of medication or consuming large quantities of grapefruit juice and they should discuss all medication, both prescription and over the counter, with their healthcare provider. Instruct patients to promptly report any unexplained muscle pain, tenderness or weakness particularly if accompanied by malaise or fever *[see Warnings and Precautions (5.1), Drug Interactions (7.1)]*.

Hepatic Dysfunction

Inform patients that LIPITOR may cause liver enzyme elevations and possibly liver failure. Advise patients to promptly report fatigue, anorexia, right upper abdominal discomfort, dark urine or jaundice *[see Warnings and Precautions (5.3)]*.

Increases in HbA1c and Fasting Serum Glucose Levels

Inform patients that increases in HbA1c and fasting serum glucose levels may occur with LIPITOR. Encourage patients to optimize lifestyle measures, including regular exercise, maintaining a healthy body weight, and making healthy food choices *[see Warnings and Precautions (5.4)]*.

Pregnancy

Advise pregnant patients and patients who can become pregnant of the potential risk to a fetus. Advise patients to inform their healthcare provider of a known or suspected pregnancy to discuss if LIPITOR should be discontinued *[see Use in Specific Populations (8.1)]*.

Lactation

Advise patients that breastfeeding is not recommended during treatment with LIPITOR *[see Use in Specific Populations (8.2)]*.

This product's labeling may have been updated. For the most recent prescribing information, please visit www.lipitor.com.

Distributed by:
Viatris Specialty LLC
Morgantown, WV 26505 U.S.A.

UPJ:LPTR: RX2

Patient Information

LIPITOR (LIP-ih-tore)
atorvastatin calcium
tablets, for oral use

What is LIPITOR?
LIPITOR is a prescription medicine that contains a cholesterol lowering medicine (statin) called atorvastatin. LIPITOR is used:

- to reduce the risk of:
    - heart attack, stroke, certain types of heart surgery and chest pain in adults who do not have heart disease but have other multiple risk factors for heart disease.
    - heart attack and stroke in adults with type 2 diabetes mellitus who do not have heart disease but have other multiple risk factors.
    - heart attack that does not cause death, stroke, certain types of heart surgery, hospitalization for congestive heart failure, and chest pain in adults with heart disease.
- along with diet to reduce low density lipoprotein cholesterol (LDL-C) or bad cholesterol:
    - in adults with primary hyperlipidemia.
    - in adults and children aged 10 years and older with heterozygous familial hypercholesterolemia (HeFH). This is an inherited condition that causes high levels of bad cholesterol.
- along with other cholesterol lowering treatments or alone if such treatments are unavailable in adults and children aged 10 years and older with homozygous familial hypercholesterolemia (HoFH). This is an inherited condition that causes high levels of bad cholesterol.
- along with diet for the treatment of adults with:
    - primary dysbetalipoproteinemia (an inherited condition that causes high levels of cholesterol and fat).
    - hypertriglyceridemia.

It is not known if LIPITOR is safe and effective in children younger than 10 years of age

with HeFH or HoFH or in children with other types of hyperlipidemias (other than HeFH or HoFH).

Do not take LIPITOR if you:

- have liver problems (acute liver failure or decompensated cirrhosis)
- are allergic to atorvastatin or any of the ingredients in LIPITOR. Stop using LIPITOR and get medical help right away if you have symptoms of a serious allergic reaction including:
  - swelling of your face, lips, tongue or throat
  - problems breathing or swallowing
  - fainting or feeling dizzy
  - very rapid heartbeat
  - severe skin rash or itching
  - flu-like symptoms including fever, sore throat, cough, tiredness, and joint pain

See the end of this leaflet for a complete list of ingredients in LIPITOR.

Before you take LIPITOR, tell your doctor about all of your medical conditions, including if you:

- have unexplained muscle aches or weakness
- drink more than 2 glasses of alcohol daily
- have diabetes
- have thyroid problems
- have kidney problems
- had a stroke
- are pregnant or plan to become pregnant. LIPITOR may harm your unborn baby. If you become pregnant, stop taking LIPITOR and call your doctor right away.
- are breastfeeding or plan to breastfeed. You and your doctor should decide if you will take LIPITOR or breastfeed. You should not do both. Talk to your doctor about the best way to feed your baby if you take LIPITOR.

Tell your doctor about all the medicines you take, including prescription and over-the-counter medicines, vitamins, and herbal supplements. LIPITOR and certain other medicines can increase the risk of muscle problems or other side effects. Especially tell your doctor if you take medicines for:

- your immune system (cyclosporine)
- cholesterol (gemfibrozil)
- infections (erythromycin, clarithromycin, itraconazole, ketoconazole, posaconazole, and voriconazole)
- birth control pills
- heart failure (digoxin)
- gout (colchicine)
- niacin
- fibrates
- viruses that treat HIV, AIDS, or hepatitis C (anti-virals)

- tipranavir plus ritonavir
- glecaprevir plus pibrentasvir
- ledipasvir plus sofosbuvir
- simeprevir

- o saquinavir plus ritonavir
- o fosamprenavir
- o elbasvir plus grazoprevir
- o nelfinavir
- o darunavir plus ritonavir
- o fosamprenavir plus ritonavir
- o letermovir

Ask your doctor or pharmacist for a list of medicines if you are not sure. Know all the medicines you take. Keep a list of them to show your doctor and pharmacist when you get a new medicine.

How should I take LIPITOR?

- Take LIPITOR exactly as your doctor tells you to take it.
- Do not change your dose or stop LIPITOR without talking to your doctor.
- Your doctor may do blood tests to check your cholesterol levels during your treatment with LIPITOR. Your dose of LIPITOR may be changed based on these blood test results.
- Take LIPITOR each day at any time of day. LIPITOR can be taken with or without food.
- Your doctor may start you on a cholesterol lowering diet before giving you LIPITOR. Stay on this low-fat diet when you take LIPITOR.
- If you miss a dose of LIPITOR, take it as soon as you remember. Do not take LIPITOR if it has been more than 12 hours since you missed your last dose. Wait and take the next dose at your regular time. Do not take 2 doses of LIPITOR at the same time. If you take too much LIPITOR or overdose, call your doctor or Poison Control Center at 1-800-222-1222 or go to the nearest emergency room right away.

What should I avoid while taking LIPITOR?

- Avoid drinking more than 1.2 liters of grapefruit juice each day.

What are the possible side effects of LIPITOR?
LIPITOR may cause serious side effects including:

- **Muscle pain, tenderness and weakness (myopathy).** Muscle problems, including muscle breakdown, can be serious in some people and, rarely, cause kidney damage that can lead to death.
  Tell your doctor right away if you have:
  - o unexplained muscle pain, tenderness, or weakness, especially if you also have a fever or feel more tired than usual while you take LIPITOR.
  - o muscle problems that do not go away after your doctor has told you to stop taking LIPITOR. Your doctor may do further tests to diagnose the cause of your muscle problems.

  Your chances of getting muscle problems are higher if you:

  - o are taking certain other medicines while you take LIPITOR
  - o drink large amounts of grapefruit juice
  - o are 65 years of age or older
  - o have thyroid problems (hypothyroidism) that are not controlled
  - o have kidney problems

- o are taking higher doses of LIPITOR

- **Liver problems.** Your doctor should do blood tests to check your liver before you start taking LIPITOR and if you have symptoms of liver problems while you take LIPITOR. Call your doctor right away if you have the following symptoms of liver problems:
  - o feel tired or weak
  - o nausea or vomiting
  - o loss of appetite
  - o upper belly pain
  - o dark amber colored urine
  - o yellowing of your skin or the whites of your eyes

- Increase in blood sugar level. Your blood sugar level may increase while you are taking LIPITOR.
  Exercise regularly and make healthy food choices to maintain healthy body weight.

The most common side effects of LIPITOR include:

- nasal congestion, sore throat, runny nose
- diarrhea
- urinary tract infection
- nausea
- muscle spasms
- throat pain
- muscle and joint pain
- pain in extremity
- upset stomach
- musculoskeletal pain
- trouble sleeping

Talk to your doctor or pharmacist if you have side effects that bother you or that will not go away.
These are not all the side effects of LIPITOR. Call your doctor for medical advice about side effects. You may report side effects to FDA at 1-800-FDA-1088.

How do I store LIPITOR?

- Store LIPITOR at room temperature between 68°F to 77°F (20 C to 25°C).
- Do not keep medicine that is out of date or that you no longer need.
- **Keep LIPITOR and all medicines out of the reach of children.**

General information about the safe and effective use of LIPITOR.
Medicines are sometimes prescribed for purposes other than those listed in a Patient Information leaflet. Do not use LIPITOR for a condition for which it was not prescribed. Do not give LIPITOR to other people, even if they have the same symptoms that you have. It may harm them. If you would like more information about LIPITOR, talk with your doctor. You can ask your pharmacist or doctor for information about LIPITOR that is written for health professionals.

What are the ingredients in LIPITOR?
Active Ingredient: atorvastatin calcium
Inactive Ingredients: calcium carbonate, USP; candelilla wax, FCC; croscarmellose sodium, NF; hydroxypropyl cellulose, NF; lactose monohydrate, NF; magnesium stearate, NF; microcrystalline cellulose, NF; Opadry White YS-1-7040 (hypromellose, polyethylene glycol, talc, titanium dioxide); polysorbate 80, NF; simethicone emulsion.

Distributed by:
Viatris Specialty LLC
Morgantown, WV 26505 U.S.A.

For more information, go to the LIPITOR website at www.lipitor.com

This Patient Package Information has been approved by the U.S. Food and Drug Administration 12/2022

UPJ:PL:LPTR: RX2

PRINCIPAL DISPLAY PANEL – 10 mg

NDC 0071-0155-23

Lipitor®
atorvastatin calcium)
tablets

10 mg*

90 Tablets **Rx only**

Store at controlled room temperature 20-25ºC (68-77ºF).

Dispense in tight containers (USP).

DOSAGE AND USE

See package insert for full prescribing information.

* Each tablet contains atorvastatin 10 mg (equivalent to 10.36 mg atorvastatin calcium anhydrous).

MADE IN IRELAND

Distributed by
Parke-Davis
Division of Pfizer Inc
NY, NY 10017

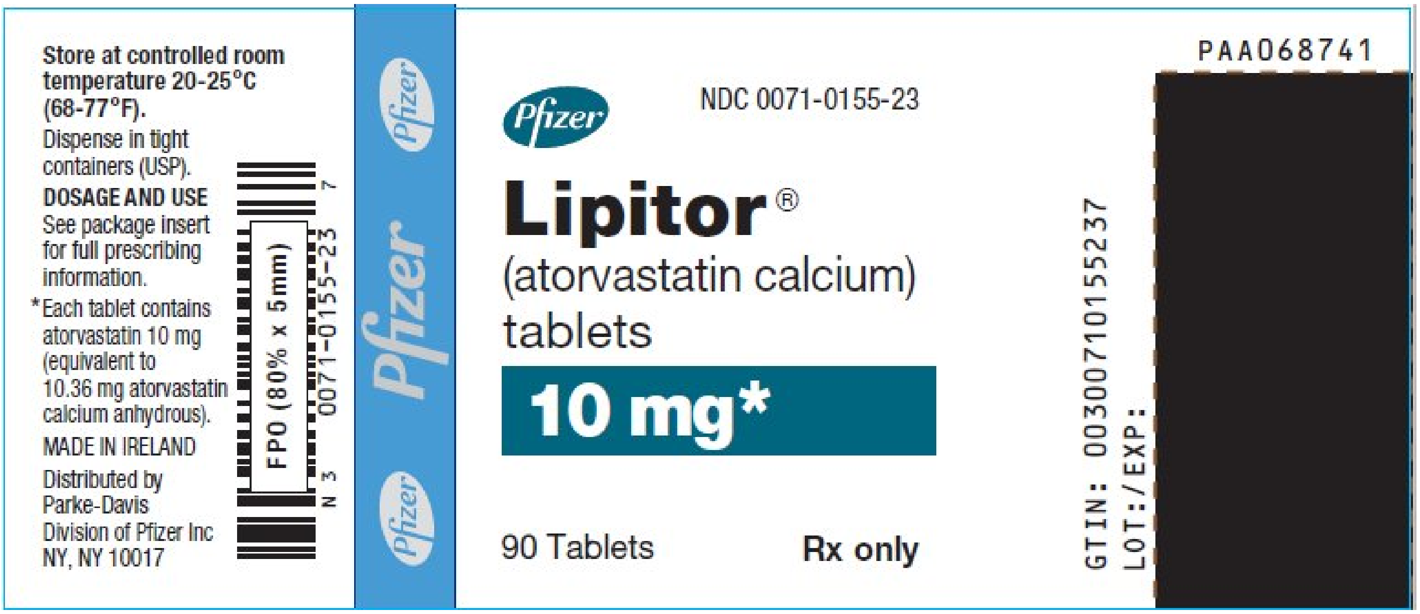

PRINCIPAL DISPLAY PANEL – 20 mg

NDC 0071-0156-23

Lipitor®
(atorvastatin calcium)
tablets

20 mg*

90 Tablets **Rx only**

Store at controlled room temperature 20-25ºC (68-77ºF).

Dispense in tight containers (USP).

DOSAGE AND USE

See package insert for full prescribing information.

* Each tablet contains atorvastatin 20 mg (equivalent to 20.72 mg atorvastatin calcium anhydrous).

MADE IN IRELAND

Distributed by
Parke-Davis
Division of Pfizer Inc
NY, NY 10017

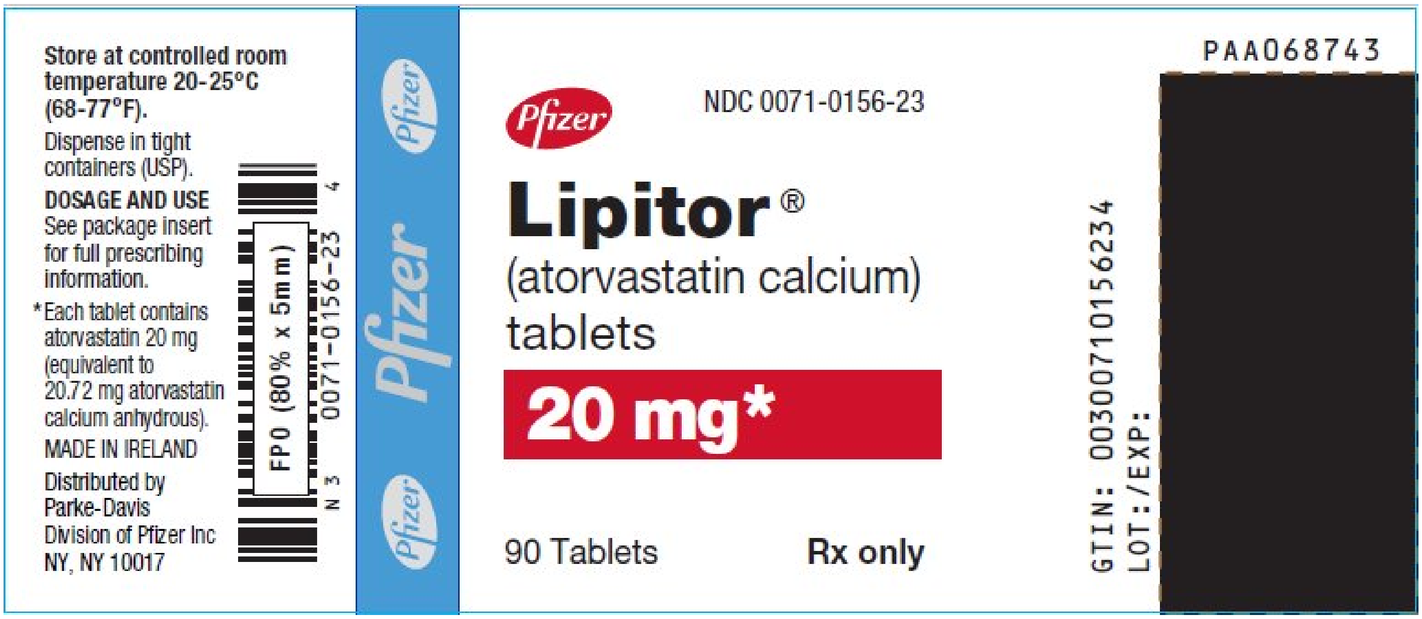

PRINCIPAL DISPLAY PANEL – 40 mg

NDC 0071-0157-23

Lipitor®
(atorvastatin calcium)

40 mg*

tablets

90 Tablets **Rx only**

Store at controlled room temperature 20-25ºC (68-77ºF).

Dispense in tight containers (USP).

DOSAGE AND USE
See package insert for full prescribing information.

* Each tablet contains atorvastatin 40 mg (equivalent to 41.44 mg atorvastatin calcium anhydrous).

Distributed by
Parke-Davis
Division of Pfizer Inc
NY, NY 10017

MADE IN IRELAND

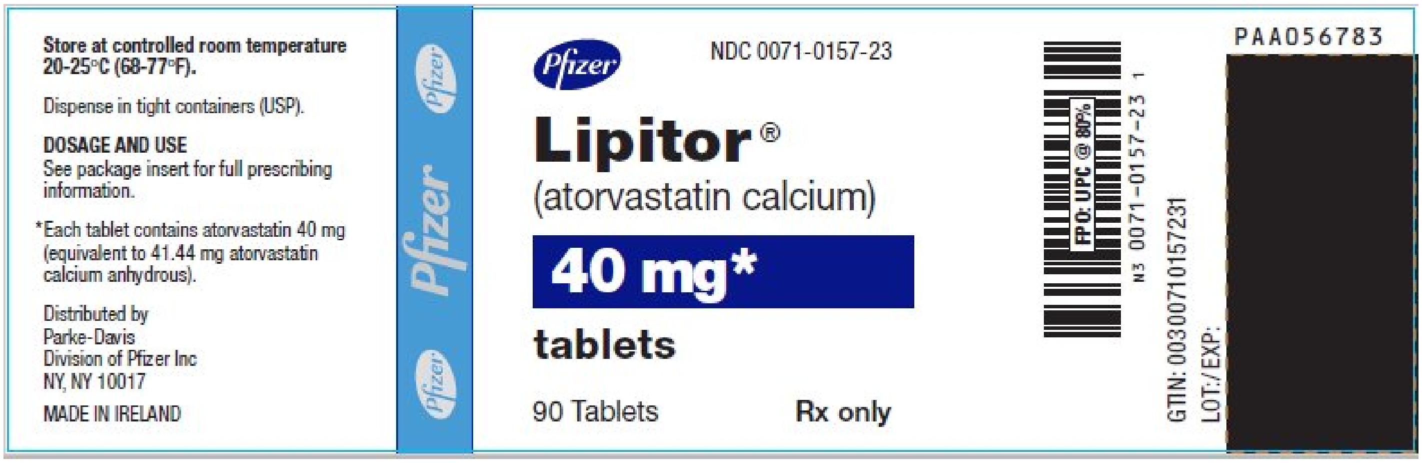

PRINCIPAL DISPLAY PANEL – 80 mg

NDC 0071-0158-23

Lipitor®
(atorvastatin calcium)

80 mg*

tablets

90 Tablets **Rx only**

Store at controlled room temperature 20-25ºC (68-77ºF).

Dispense in tight containers (USP).

DOSAGE AND USE
See package insert for full prescribing information.

* Each tablet contains atorvastatin 80 mg (equivalent to 82.88 mg atorvastatin calcium anhydrous).

MADE IN IRELAND

Distributed by
Parke-Davis
Division of Pfizer Inc
NY, NY 10017

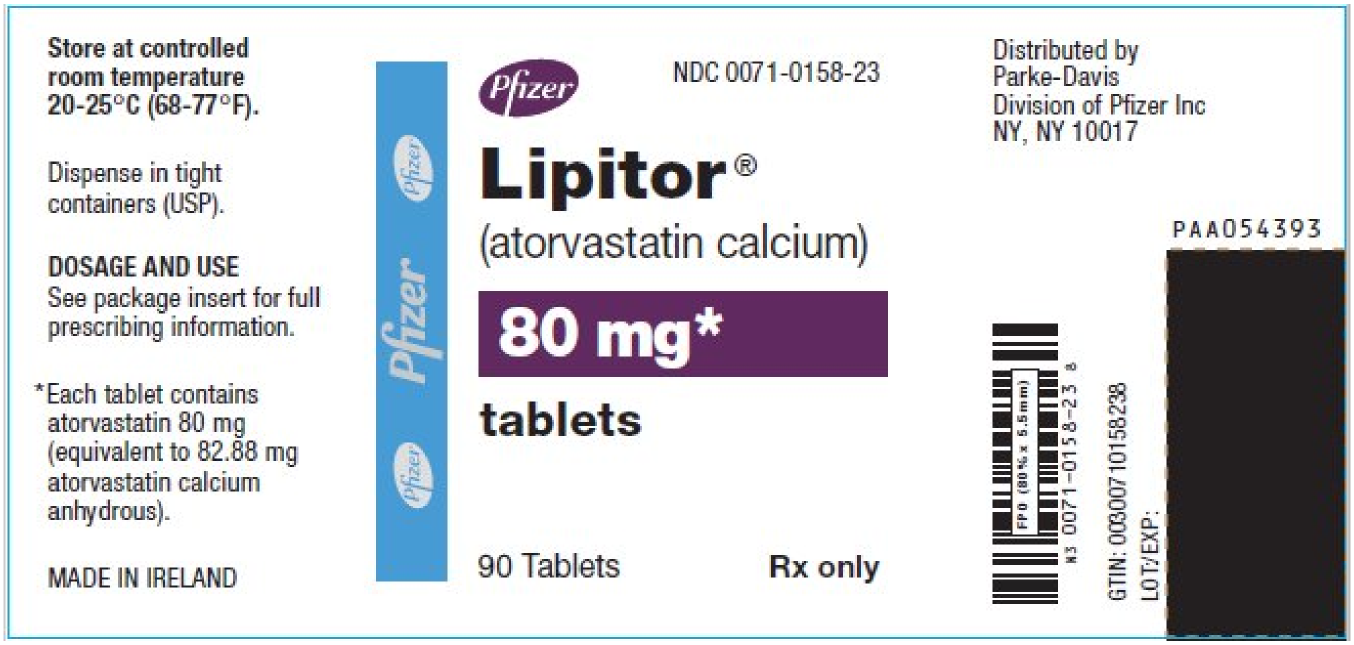

LIPITOR

atorvastatin calcium tablet, film coated

Product Information			
**Product Type**	HUMAN PRESCRIPTION DRUG	**Item Code (Source)**	NDC:0071-0155
**Route of Administration**	ORAL		

Active Ingredient/Active Moiety		
**Ingredient Name**	**Basis of Strength**	**Strength**
**ATORVASTATIN CALCIUM TRIHYDRATE** (UNII: 48A5M73Z4Q) (ATORVASTATIN - UNII:A0JWA85V8F)	ATORVASTATIN	10 mg

Inactive Ingredients	
**Ingredient Name**	**Strength**
**CALCIUM CARBONATE** (UNII: H0G9379FGK)	
**CANDELILLA WAX** (UNII: WL0328HX19)	
**CROSCARMELLOSE SODIUM** (UNII: M28OL1HH48)	
**HYDROXYPROPYL CELLULOSE (1600000 WAMW)** (UNII: RFW2ET671P)	
**LACTOSE MONOHYDRATE** (UNII: EWQ57Q8I5X)	
**MAGNESIUM STEARATE** (UNII: 70097M6I30)	
**MICROCRYSTALLINE CELLULOSE** (UNII: OP1R32D61U)	
**HYPROMELLOSE, UNSPECIFIED** (UNII: 3NXW29V3WO)	
**POLYETHYLENE GLYCOL, UNSPECIFIED** (UNII: 3WQ0SDW1A)	
**TALC** (UNII: 7SEV7J4R1U)	
**TITANIUM DIOXIDE** (UNII: 15FIX9V2JP)	
**POLYSORBATE 80** (UNII: 6OZP39ZG8H)	

Product Characteristics

**Color**	WHITE	**Score**	no score
**Shape**	OVAL (elliptical)	**Size**	10mm
**Flavor**		**Imprint Code**	PD;155;10
**Contains**			

Packaging

#	Item Code	Package Description	Marketing Start Date	Marketing End Date
1	NDC:0071-0155-23	90 in 1 BOTTLE; Type 0: Not a Combination Product	12/17/1996	04/30/2025
2	NDC:0071-0155-40	100 in 1 CARTON	12/17/1996	03/31/2023
2		1 in 1 BLISTER PACK; Type 0: Not a Combination Product		

Marketing Information

Marketing Category	Application Number or Monograph Citation	Marketing Start Date	Marketing End Date
NDA	NDA020702	12/17/1996	04/30/2025

LIPITOR

atorvastatin calcium tablet, film coated

Product Information

**Product Type**	HUMAN PRESCRIPTION DRUG	**Item Code (Source)**	NDC:0071-0156
**Route of Administration**	ORAL		

Active Ingredient/Active Moiety

Ingredient Name	Basis of Strength	Strength
**ATORVASTATIN CALCIUM TRIHYDRATE** (UNII: 48A5M73Z4Q) (ATORVASTATIN - UNII:A0JWA85V8F)	ATORVASTATIN	20 mg

Inactive Ingredients

Ingredient Name	Strength
**CALCIUM CARBONATE** (UNII: H0G9379FGK)	
**CANDELILLA WAX** (UNII: WL0328HX19)	
**CROSCARMELLOSE SODIUM** (UNII: M28OL1HH48)	
**HYDROXYPROPYL CELLULOSE (1600000 WAMW)** (UNII: RFW2ET671P)	
**LACTOSE MONOHYDRATE** (UNII: EWQ57Q8I5X)	
**MAGNESIUM STEARATE** (UNII: 70097M6I30)	
**MICROCRYSTALLINE CELLULOSE** (UNII: OP1R32D61U)	

**HYPROMELLOSE, UNSPECIFIED** (UNII: 3NXW29V3WO)	
**POLYETHYLENE GLYCOL, UNSPECIFIED** (UNII: 3WJQ0SDW1A)	
**TALC** (UNII: 7SEV7J4R1U)	
**TITANIUM DIOXIDE** (UNII: 15FIX9V2JP)	
**POLYSORBATE 80** (UNII: 6OZP39ZG8H)	

Product Characteristics

**Color**	WHITE	**Score**	no score
**Shape**	OVAL (elliptical)	**Size**	12mm
**Flavor**		**Imprint Code**	PD;156;20
**Contains**			

Packaging

#	Item Code	Package Description	Marketing Start Date	Marketing End Date
**1**	NDC:0071-0156-23	90 in 1 BOTTLE; Type 0: Not a Combination Product	12/17/1996	09/30/2025
**2**	NDC:0071-0156-40	100 in 1 CARTON	12/17/1996	11/30/2023
**2**		1 in 1 BLISTER PACK; Type 0: Not a Combination Product		

Marketing Information

Marketing Category	Application Number or Monograph Citation	Marketing Start Date	Marketing End Date
NDA	NDA020702	12/17/1996	09/30/2025

LIPITOR

atorvastatin calcium tablet, film coated

Product Information

**Product Type**	HUMAN PRESCRIPTION DRUG	**Item Code (Source)**	NDC:0071-0157
**Route of Administration**	ORAL		

Active Ingredient/Active Moiety

Ingredient Name	Basis of Strength	Strength
**ATORVASTATIN CALCIUM TRIHYDRATE** (UNII: 48A5M73Z4Q) (ATORVASTATIN - UNII:A0JWA85V8F)	ATORVASTATIN	40 mg

Inactive Ingredients

Ingredient Name	Strength

**CALCIUM CARBONATE** (UNII: H0G9379FGK)	
**CANDELILLA WAX** (UNII: WL0328HX19)	
**CROSCARMELLOSE SODIUM** (UNII: M28OL1HH48)	
**HYDROXYPROPYL CELLULOSE (1600000 WAMW)** (UNII: RFW2ET671P)	
**LACTOSE MONOHYDRATE** (UNII: EWQ57Q8I5X)	
**MAGNESIUM STEARATE** (UNII: 70097M6I30)	
**MICROCRYSTALLINE CELLULOSE** (UNII: OP1R32D61U)	
**HYPROMELLOSE, UNSPECIFIED** (UNII: 3NXW29V3WO)	
**POLYETHYLENE GLYCOL, UNSPECIFIED** (UNII: 3WJQ0SDW1A)	
**TALC** (UNII: 7SEV7J4R1U)	
**TITANIUM DIOXIDE** (UNII: 15FIX9V2JP)	
**POLYSORBATE 80** (UNII: 6OZP39ZG8H)	

Product Characteristics

**Color**	WHITE	**Score**	no score
**Shape**	OVAL (elliptical)	**Size**	15mm
**Flavor**		**Imprint Code**	PD;157;40
**Contains**			

Packaging

#	Item Code	Package Description	Marketing Start Date	Marketing End Date
**1**	NDC:0071-0157-23	90 in 1 BOTTLE; Type 0: Not a Combination Product	12/17/1996	03/31/2026
**2**	NDC:0071-0157-40	100 in 1 CARTON	12/17/1996	09/30/2023
**2**		1 in 1 BLISTER PACK; Type 0: Not a Combination Product		

Marketing Information

Marketing Category	Application Number or Monograph Citation	Marketing Start Date	Marketing End Date
NDA	NDA020702	12/17/1996	03/31/2026

LIPITOR

atorvastatin calcium tablet, film coated

Product Information

**Product Type**	HUMAN PRESCRIPTION DRUG	**Item Code (Source)**	NDC:0071-0158
**Route of Administration**	ORAL		

Active Ingredient/Active Moiety

Ingredient Name	Basis of	Strength

Ingredient Name	Basis of Strength	Strength
**ATORVASTATIN CALCIUM TRIHYDRATE** (UNII: 48A5M73Z4Q) (ATORVASTATIN - UNII:A0JWA85V8F)	ATORVASTATIN	80 mg

Inactive Ingredients	
**Ingredient Name**	**Strength**
**CALCIUM CARBONATE** (UNII: H0G9379FGK)	
**CANDELILLA WAX** (UNII: WL0328HX19)	
**CROSCARMELLOSE SODIUM** (UNII: M28OL1HH48)	
**HYDROXYPROPYL CELLULOSE (1600000 WAMW)** (UNII: RFW2ET671P)	
**LACTOSE MONOHYDRATE** (UNII: EWQ57Q8I5X)	
**MAGNESIUM STEARATE** (UNII: 70097M6I30)	
**MICROCRYSTALLINE CELLULOSE** (UNII: OP1R32D61U)	
**HYPROMELLOSE, UNSPECIFIED** (UNII: 3NXW29V3WO)	
**POLYETHYLENE GLYCOL, UNSPECIFIED** (UNII: 3WJQ0SDW1A)	
**TALC** (UNII: 7SEV7J4R1U)	
**TITANIUM DIOXIDE** (UNII: 15FIX9V2JP)	
**POLYSORBATE 80** (UNII: 6OZP39ZG8H)	

Product Characteristics			
**Color**	WHITE	**Score**	no score
**Shape**	OVAL (elliptical)	**Size**	19mm
**Flavor**		**Imprint Code**	PD;158;80
**Contains**			

Packaging				
**#**	**Item Code**	**Package Description**	**Marketing Start Date**	**Marketing End Date**
**1**	NDC:0071-0158-23	90 in 1 BOTTLE; Type 0: Not a Combination Product	04/07/2000	09/30/2025
**2**	NDC:0071-0158-92	64 in 1 CARTON	04/07/2000	07/31/2023
**2**		1 in 1 BLISTER PACK; Type 0: Not a Combination Product		

Marketing Information			
**Marketing Category**	**Application Number or Monograph Citation**	**Marketing Start Date**	**Marketing End Date**
NDA	NDA020702	04/07/2000	09/30/2025

Labeler - Parke-Davis Div of Pfizer Inc (829076962)

XANAX

XANAX- alprazolam tablet
PHARMACIA & UPJOHN COMPANY LLC

HIGHLIGHTS OF PRESCRIBING INFORMATION
These highlights do not include all the information needed to use XANAX safely and effectively. See full prescribing information for XANAX.

XANAX (alprazolam) tablets, for oral use, CIV
Initial U.S. Approval: 1981

WARNING: RISKS FROM CONCOMITANT USE WITH OPIOIDS; ABUSE, MISUSE, AND ADDICTION; and DEPENDENCE AND WITHDRAWAL REACTIONS

See full prescribing information for complete boxed warning.

- **Concomitant use of benzodiazepines and opioids may result in profound sedation, respiratory depression, coma, and death. Reserve concomitant prescribing for use in patients for whom alternative treatment options are inadequate. Limit dosages and durations to the minimum required. Follow patients for signs and symptoms of respiratory depression and sedation. (5.1, 7.1)**
- **The use of benzodiazepines, including XANAX, exposes users to risks of abuse, misuse, and addiction, which can lead to overdose or death. Before prescribing XANAX and throughout treatment, assess each patient's risk for abuse, misuse, and addiction. (5.2)**
- **Abrupt discontinuation or rapid dosage reduction of XANAX after continued use may precipitate acute withdrawal reactions, which can be life-threatening. To reduce the risk of withdrawal reactions, use a gradual taper to discontinue XANAX or reduce the dosage. (2.2, 5.3)**

RECENT MAJOR CHANGES

Warnings and Precautions (5.8) 1/2023

INDICATIONS AND USAGE

XANAX is a benzodiazepine indicated for the:

- Acute treatment of generalized anxiety disorder in adults. (1)
- Treatment of panic disorder with or without agoraphobia in adults. (1)

DOSAGE AND ADMINISTRATION

- Generalized Anxiety Disorder: (2.1)
  - Recommended starting oral dosage is 0.25 mg to 0.5 mg three times daily.
  - Dosage may be increased, at intervals of every 3 to 4 days, to a maximum recommended daily dose of 4 mg, given in divided doses.
  - Use the lowest possible effective dose and frequently assess the need for continued treatment.
- Panic Disorder: Recommended starting oral dosage is 0.5 mg three times daily. The dosage may be increased at intervals of every 3 to 4 days in increments of no more than 1 mg per day. (2.2)
- When tapering, decrease dosage by no more than 0.5 mg every 3 days. Some patients may require an even slower dosage reduction. (2.3, 5.2)
- See the Full Prescribing Information for the recommended dosage in geriatric patients, patients with hepatic impairment, and with use with ritonavir. (2.4, 2.5, 2.6)

DOSAGE FORMS AND STRENGTHS

Tablets: 0.25 mg, 0.5 mg, 1 mg, and 2 mg (3)

CONTRAINDICATIONS

- Known hypersensitivity to alprazolam or other benzodiazepines. (4)
- Concomitant use with strong cytochrome P450 3A (CYP3A) inhibitors, except ritonavir. (4, 5.5, 7.1)

WARNINGS AND PRECAUTIONS

- Effects on Driving and Operating Machinery: Patients receiving XANAX should be cautioned against operating machinery or driving a motor vehicle, as well as avoiding concomitant use of alcohol and other central nervous system (CNS) depressant drugs. (5.4)
- Patients with Depression: Exercise caution in patients with signs or symptoms of depression. Prescribe the least number of tablets feasible to avoid intentional overdosage. (5.6)
- Neonatal Sedation and Withdrawal Syndrome: XANAX use during pregnancy can result in neonatal sedation and/or neonatal withdrawal. (5.8, 8.1)

ADVERSE REACTIONS

The most common adverse reactions reported in clinical trials for generalized anxiety disorder and panic disorder (incidence >5% and at least twice that of placebo) include: impaired coordination, hypotension, dysarthria, and increased libido. (6.1)

To report SUSPECTED ADVERSE REACTIONS, contact Viatris at 1-877-446-3679 (1-877-4-INFO-RX) or FDA at 1-800-FDA-1088 or www.fda.gov/medwatch.

DRUG INTERACTIONS

- Use with Opioids: Increase the risk of respiratory depression. (7.1)
- Use with Other CNS Depressants: Produces additive CNS depressant effects. (7.1)
- Use with Digoxin: Increase the risk of digoxin toxicity. (7.1)
- Use with CYP3A Inhibitors (except ritonavir): Increase the risk of adverse reactions of alprazolam. (4, 5.5, 7.1)
- Use with CYP3A Inducers: Increase the risk of reduced efficacy of alprazolam. (7.1)

USE IN SPECIFIC POPULATIONS

Lactation: Breastfeeding not recommended. (8.2)

See 17 for PATIENT COUNSELING INFORMATION and Medication Guide.

Revised: 1/2023

FULL PRESCRIBING INFORMATION: CONTENTS*

WARNING: RISKS FROM CONCOMITANT USE WITH OPIOIDS; ABUSE, MISUSE, AND ADDICTION; and DEPENDENCE AND WITHDRAWAL REACTIONS
1 INDICATIONS AND USAGE
2 DOSAGE AND ADMINISTRATION
2.1 Dosage in Generalized Anxiety Disorder
2.2 Dosage in Panic Disorder
2.3 Discontinuation or Dosage Reduction of XANAX
2.4 Dosage Recommendations in Geriatric Patients
2.5 Dosage Recommendations in Patients with Hepatic Impairment
2.6 Dosage Modifications for Drug Interactions
3 DOSAGE FORMS AND STRENGTHS
4 CONTRAINDICATIONS
5 WARNINGS AND PRECAUTIONS
5.1 Risks from Concomitant Use with Opioids
5.2 Abuse, Misuse, and Addiction
5.3 Dependence and Withdrawal Reactions
5.4 Effects on Driving and Operating Machinery
5.5 Interaction with Drugs that Inhibit Metabolism via Cytochrome P450 3A
5.6 Patients with Depression
5.7 Mania
5.8 Neonatal Sedation and Withdrawal Syndrome

5.9 Risk in Patients with Impaired Respiratory Function
6 ADVERSE REACTIONS
6.1 Clinical Trials Experience
6.2 Postmarketing Experience
7 DRUG INTERACTIONS
7.1 Drugs Having Clinically Important Interactions with XANAX
7.2 Drug/Laboratory Test Interactions
8 USE IN SPECIFIC POPULATIONS
8.1 Pregnancy
8.2 Lactation
8.4 Pediatric Use
8.5 Geriatric Use
8.6 Hepatic Impairment
9 DRUG ABUSE AND DEPENDENCE
9.1 Controlled Substance
9.2 Abuse
9.3 Dependence
10 OVERDOSAGE
11 DESCRIPTION
12 CLINICAL PHARMACOLOGY
12.1 Mechanism of Action
12.3 Pharmacokinetics
13 NONCLINICAL TOXICOLOGY
13.1 Carcinogenesis, Mutagenesis, Impairment of Fertility
13.2 Animal Toxicology and/or Pharmacology
14 CLINICAL STUDIES
14.1 Generalized Anxiety Disorder
14.2 Panic Disorder
16 HOW SUPPLIED/STORAGE AND HANDLING
17 PATIENT COUNSELING INFORMATION
* Sections or subsections omitted from the full prescribing information are not listed.

FULL PRESCRIBING INFORMATION

WARNING: RISKS FROM CONCOMITANT USE WITH OPIOIDS; ABUSE, MISUSE, AND ADDICTION; and DEPENDENCE AND WITHDRAWAL REACTIONS

- **Concomitant use of benzodiazepines and opioids may result in profound sedation, respiratory depression, coma, and death. Reserve concomitant prescribing of these drugs for patients for whom alternative treatment options are inadequate. Limit dosages and durations to the minimum required. Follow patients for signs and symptoms of respiratory depression and sedation *[see Warnings and Precautions (5.1), Drug Interactions (7.1)]*.**
- **The use of benzodiazepines, including XANAX, exposes users to risks of abuse, misuse, and addiction, which can lead to overdose or death. Abuse and misuse of benzodiazepines commonly involve concomitant use of other medications, alcohol, and/or illicit substances, which is associated with an increased frequency of serious adverse outcomes. Before prescribing XANAX and throughout treatment, assess each patient's risk for abuse, misuse, and addiction *[see Warnings and Precautions (5.2)]*.**
- **The continued use of benzodiazepines, including XANAX, may lead to clinically significant physical dependence. The risks of dependence and withdrawal increase with longer treatment duration and higher daily dose. Abrupt discontinuation or rapid dosage reduction of XANAX after continued use may precipitate acute withdrawal reactions, which can be life-threatening. To reduce the risk of withdrawal reactions, use a gradual taper to discontinue XANAX or reduce the dosage *[see Dosage and Administration (2.2), Warnings and Precautions (5.3)]*.**

1 INDICATIONS AND USAGE

XANAX is indicated for the:

- acute treatment of generalized anxiety disorder (GAD) in adults.
- treatment of panic disorder (PD), with or without agoraphobia in adults.

2 DOSAGE AND ADMINISTRATION

2.1 Dosage in Generalized Anxiety Disorder

The recommended starting oral dosage of XANAX for the acute treatment of patients with GAD is 0.25 mg to 0.5 mg administered three times daily. Depending upon the response, the dosage may be adjusted at intervals of every 3 to 4 days. The maximum recommended dosage is 4 mg daily (in divided doses).

Use the lowest possible effective dose and frequently assess the need for continued treatment *[see Warnings and Precautions (5.2)]*.

2.2 Dosage in Panic Disorder

The recommended starting oral dosage of XANAX for the treatment of PD is 0.5 mg three times daily. Depending on the response, the dosage may be increased at intervals of every 3 to 4 days in increments of no more than 1 mg per day.

Controlled trials of XANAX in the treatment of panic disorder included dosages in the range of 1 mg to 10 mg daily. The mean dosage was approximately 5 mg to 6 mg daily. Occasional patients required as much as 10 mg per day.

For patients receiving doses greater than 4 mg per day, periodic reassessment and consideration of dosage reduction is advised. In a controlled postmarketing dose-response study, patients treated with doses of XANAX greater than 4 mg per day for 3 months were able to taper to 50% of their total maintenance dose without apparent loss of clinical benefit.

The necessary duration of treatment for PD in patients responding to XANAX is unknown. After a period of extended freedom from panic attacks, a carefully supervised tapered discontinuation may be attempted, but there is evidence that this may often be difficult to accomplish without recurrence of symptoms and/or the manifestation of withdrawal phenomena *[see Dosage and Administration (2.3)]*.

2.3 Discontinuation or Dosage Reduction of XANAX

To reduce the risk of withdrawal reactions, use a gradual taper to discontinue XANAX or reduce the dosage. If a patient develops withdrawal reactions, consider pausing the taper or increasing the dosage to the previous tapered dosage level. Subsequently decrease the dosage more slowly *[see Warnings and Precautions (5.3), Drug Abuse and Dependence (9.3)]*.

Reduced the dosage by no more than 0.5 mg every 3 days. Some patients may benefit from an even more gradual discontinuation. Some patients may prove resistant to all discontinuation regimens.

In a controlled postmarketing discontinuation study of panic disorder patients which compared the recommended taper schedule with a slower taper schedule, no difference was observed between the groups in the proportion of patients who tapered to zero dose; however, the slower schedule was associated with a reduction in symptoms associated with a withdrawal syndrome.

2.4 Dosage Recommendations in Geriatric Patients

In geriatric patients, the recommended starting oral dosage of XANAX is 0.25 mg, given 2 or 3 times daily. This may be gradually increased if needed and tolerated. Geriatric patients may be especially sensitive to the effects of benzodiazepines. If adverse reactions occur at the recommended starting dosage, the dosage may be reduced *[see Use in Specific Populations (8.5), Clinical Pharmacology (12.3)]*.

2.5 Dosage Recommendations in Patients with Hepatic Impairment

In patients with hepatic impairment, the recommended starting oral dosage of XANAX is 0.25 mg, given 2 or 3 times daily. This may be gradually increased if needed and tolerated. If adverse reactions occur at the recommended starting dose, the dosage may be reduced *[see Use in Specific Populations (8.6), Clinical Pharmacology (12.3)]*.

2.6 Dosage Modifications for Drug Interactions

XANAX should be reduced to half of the recommended dosage when a patient is started on ritonavir and XANAX together, or when ritonavir administered to a patient treated with XANAX. Increase the XANAX dosage to the target dose after 10 to 14 days of dosing ritonavir and XANAX together. It is not necessary to reduce XANAX dose in patients who have been taking ritonavir for more than 10 to 14 days.

XANAX is contraindicated with concomitant use of all strong CYP3A inhibitors, except ritonavir *[see Contraindications (4), Warnings and Precautions (5.5)]*.

3 DOSAGE FORMS AND STRENGTHS

XANAX tablets are available as:

- 0.25 mg: white, oval, scored, imprinted “XANAX 0.25”
- 0.5 mg: peach, oval, scored, imprinted “XANAX 0.5”
- 1 mg: blue, oval, scored, imprinted “XANAX 1.0”
- 2 mg: white, oblong, multi-scored, imprinted “XANAX ” on one side and “2” on the reverse side

4 CONTRAINDICATIONS

XANAX is contraindicated in patients:

- with known hypersensitivity to alprazolam or other benzodiazepines. Angioedema has been reported *[see Adverse Reactions (6.2)]*.
- taking strong cytochrome P450 3A (CYP3A) inhibitors (e.g., ketoconazole, itraconazole), except ritonavir *[see Dosage and Administration (2.6), Warnings and Precautions (5.5), Drug Interactions (7.1)]*

5 WARNINGS AND PRECAUTIONS

5.1 Risks from Concomitant Use with Opioids

Concomitant use of benzodiazepines, including XANAX, and opioids may result in profound sedation, respiratory depression, coma, and death. Because of these risks, reserve concomitant prescribing of these drugs in patients for whom alternative treatment options are inadequate.

Observational studies have demonstrated that concomitant use of opioid analgesics and benzodiazepines increases the risk of drug-related mortality compared to use of opioids alone. If a decision is made to prescribe XANAX concomitantly with opioids, prescribe the lowest effective dosages and minimum durations of concomitant use, and follow patients closely for signs and symptoms of respiratory depression and sedation. In patients already receiving an opioid analgesic, prescribe a lower initial dose of XANAX than indicated in the absence of an opioid and titrate based on clinical response. If an opioid is initiated in a patient already taking XANAX, prescribe a lower initial dose of the opioid and titrate based upon clinical response.

Advise both patients and caregivers about the risks of respiratory depression and

sedation when XANAX is used with opioids. Advise patients not to drive or operate heavy machinery until the effects of concomitant use with the opioid have been determined *[see Drug Interactions (7.1)]*.

5.2 Abuse, Misuse, and Addiction

The use of benzodiazepines, including XANAX, exposes users to the risks of abuse, misuse, and addiction, which can lead to overdose or death. Abuse and misuse of benzodiazepines often (but not always) involve the use of doses greater than the maximum recommended dosage and commonly involve concomitant use of other medications, alcohol, and/or illicit substances, which is associated with an increased frequency of serious adverse outcomes, including respiratory depression, overdose, or death *[see Drug Abuse and Dependence (9.2)]*.

Before prescribing XANAX and throughout treatment, assess each patient's risk for abuse, misuse, and addiction (e.g., using a standardized screening tool). Use of XANAX, particularly in patients at elevated risk, necessitates counseling about the risks and proper use of XANAX along with monitoring for signs and symptoms of abuse, misuse, and addiction. Prescribe the lowest effective dosage; avoid or minimize concomitant use of CNS depressants and other substances associated with abuse, misuse, and addiction (e.g., opioid analgesics, stimulants); and advise patients on the proper disposal of unused drug. If a substance use disorder is suspected, evaluate the patient and institute (or refer them for) early treatment, as appropriate.

5.3 Dependence and Withdrawal Reactions

To reduce the risk of withdrawal reactions, use a gradual taper to discontinue XANAX or reduce the dosage (a patient-specific plan should be used to taper the dose) *[see Dosage and Administration (2.3)]*.

Patients at an increased risk of withdrawal adverse reactions after benzodiazepine discontinuation or rapid dosage reduction include those who take higher dosages, and those who have had longer durations of use.

<u>Acute Withdrawal Reactions</u>

The continued use of benzodiazepines, including XANAX, may lead to clinically significant physical dependence. Abrupt discontinuation or rapid dosage reduction of XANAX after continued use, or administration of flumazenil (a benzodiazepine antagonist) may precipitate acute withdrawal reactions, which can be life-threatening (e.g., seizures) *[see Drug Abuse and Dependence (9.3)]*.

<u>Protracted Withdrawal Syndrome</u>

In some cases, benzodiazepine users have developed a protracted withdrawal syndrome with withdrawal symptoms lasting weeks to more than 12 months *[see Drug Abuse and Dependence (9.3)]*.

Certain adverse clinical events, some life-threatening, are a direct consequence of physical dependence to XANAX. These include a spectrum of withdrawal symptoms; the most important is seizure *[see Drug Abuse and Dependence (9.3)]*. Even after relatively short-term use at doses of ≤4 mg/day, there is some risk of dependence. Spontaneous reporting system data suggest that the risk of dependence and its severity appear to be

greater in patients treated with doses greater than 4 mg/day and for long periods (more than 12 weeks). However, in a controlled postmarketing discontinuation study of panic disorder patients who received XANAX, the duration of treatment (3 months compared to 6 months) had no effect on the ability of patients to taper to zero dose. In contrast, patients treated with doses of XANAX greater than 4 mg/day had more difficulty tapering to zero dose than those treated with less than 4 mg/day.

In a controlled clinical trial in which 63 patients were randomized to XANAX and where withdrawal symptoms were specifically sought, the following were identified as symptoms of withdrawal: heightened sensory perception, impaired concentration, dysosmia, clouded sensorium, paresthesias, muscle cramps, muscle twitch, diarrhea, blurred vision, appetite decrease, and weight loss. Other symptoms, such as anxiety and insomnia, were frequently seen during discontinuation, but it could not be determined if they were due to return of illness, rebound, or withdrawal.

Interdose Symptoms

Early morning anxiety and emergence of anxiety symptoms between doses of XANAX have been reported in patients with panic disorder taking prescribed maintenance doses. These symptoms may reflect the development of tolerance or a time interval between doses which is longer than the duration of clinical action of the administered dose. In either case, it is presumed that the prescribed dose is not sufficient to maintain plasma levels above those needed to prevent relapse, rebound, or withdrawal symptoms over the entire course of the interdosing interval.

5.4 Effects on Driving and Operating Machinery

Because of its CNS depressant effects, patients receiving XANAX should be cautioned against engaging in hazardous occupations or activities requiring complete mental alertness such as operating machinery or driving a motor vehicle. For the same reason, patients should be cautioned about the concomitant use of alcohol and other CNS depressant drugs during treatment with XANAX *[see Drug Interactions (7.1)]*.

5.5 Interaction with Drugs that Inhibit Metabolism via Cytochrome P450 3A

The initial step in alprazolam metabolism is hydroxylation catalyzed by cytochrome P450 3A (CYP3A). Drugs that inhibit this metabolic pathway may have a profound effect on the clearance of alprazolam.

Strong CYP3A Inhibitors

XANAX is contraindicated in patients receiving strong inhibitors of CYP3A (such as azole antifungal agents), except ritonavir *[see Contraindications (4)]*. Ketoconazole and itraconazole have been shown in vivo to increase plasma alprazolam concentrations 3.98 fold and 2.70 fold, respectively.

Dosage adjustment is necessary when XANAX and ritonavir are initiated concomitantly or when ritonavir is added to a stable dosage of XANAX *[see Dosage and Administration (2.6), Drug Interactions (7.1)]*.

Drugs demonstrated to be CYP3A inhibitors on the basis of clinical studies involving alprazolam: nefazodone, fluvoxamine, and cimetidine *[see Drug Interaction (7.1), Clinical Pharmacology (12.3)]*. Use caution and consider dose reduction of XANAX, as

appropriate, during co-administration with these drugs.

5.6 Patients with Depression

Benzodiazepines may worsen depression. Panic disorder has been associated with primary and secondary major depressive disorders and increased reports of suicide among untreated patients. Consequently, appropriate precautions (e.g., limiting the total prescription size and increased monitoring for suicidal ideation) should be considered in patients with depression.

5.7 Mania

Episodes of hypomania and mania have been reported in association with the use of XANAX in patients with depression *[see Adverse Reactions (6.2)]*.

5.8 Neonatal Sedation and Withdrawal Syndrome

Use of XANAX late in pregnancy can result in sedation (respiratory depression, lethargy, hypotonia) and/or withdrawal symptoms (hyperreflexia, irritability, restlessness, tremors, inconsolable crying, and feeding difficulties) in the neonate *[see Use in Specific Populations (8.1)]*. Monitor neonates exposed to XANAX during pregnancy or labor for signs of sedation and monitor neonates exposed to XANAX during pregnancy for signs of withdrawal; manage these neonates accordingly.

5.9 Risk in Patients with Impaired Respiratory Function

There have been reports of death in patients with severe pulmonary disease shortly after the initiation of treatment with XANAX. Closely monitor patients with impaired respiratory function. If signs and symptoms of respiratory depression, hypoventilation, or apnea occur, discontinue XANAX.

6 ADVERSE REACTIONS

The following clinically significant adverse reactions are described elsewhere in the labeling:

- Risks from Concomitant Use with Opioids *[see Warnings and Precautions (5.1)]*
- Abuse, Misuse, and Addiction *[see Warnings and Precautions (5.2)]*
- Dependence and Withdrawal Reactions *[see Warnings and Precautions (5.3)]*
- Effects on Driving and Operating Machinery *[see Warnings and Precautions (5.4)]*
- Patients with Depression *[see Warnings and Precautions (5.6)]*
- Neonatal Sedation and Withdrawal Syndrome *[see Warnings and Precautions (5.8)]*
- Risks in Patients with Impaired Respiratory Function *[see Warnings and Precautions (5.9)]*

6.1 Clinical Trials Experience

Because clinical trials are conducted under widely varying conditions, adverse reaction rates observed in the clinical trials of a drug cannot be directly compared to rates in the clinical trials of another drug and may not reflect the rates observed in practice.

The data in the two tables below are estimates of adverse reaction incidence among adult patients who participated in:

- 4-week placebo-controlled clinical studies with XANAX dosages up to 4 mg per day for the acute treatment of generalized anxiety disorder (Table 1)
- Short-term (up to 10 weeks) placebo-controlled clinical studies with XANAX dosages up to 10 mg per day for panic disorder, with or without agoraphobia (Table 2).

Table 1: Adverse Reactions Occurring in ≥1% in XANAX-treated Patients and Greater than Placebo-treated Patients in Placebo-Controlled Trials for Generalized Anxiety

	**XANAX n=565**	**Placebo n=505**
**Nervous system disorders**		
Drowsiness	41%	22%
Light-headedness	21%	19%
Dizziness	2%	1%
Akathisia	2%	1%
**Gastrointestinal disorders**		
Dry mouth	15%	13%
Increased salivation	4%	2%
**Cardiovascular disorders**		
Hypotension	5%	2%
**Skin and subcutaneous tissue disorders**		
Dermatitis/allergy	4%	3%

In addition to the adverse reactions (i.e., greater than 1%) enumerated in the table above for patients with generalized anxiety disorder, the following adverse reactions have been reported in association with the use of benzodiazepines: dystonia, irritability, concentration difficulties, anorexia, transient amnesia or memory impairment, loss of coordination, fatigue, seizures, sedation, slurred speech, jaundice, musculoskeletal weakness, pruritus, diplopia, dysarthria, changes in libido, menstrual irregularities, incontinence and urinary retention.

Table 2: Adverse Reactions Occurring in ≥1% in XANAX-treated Patients and Greater than Placebo-treated Patients in Placebo-Controlled Trials (Up to 10 Weeks) for Panic Disorder

	**XANAX n=1388**	**Placebo n=1231**
Drowsiness	77%	43%
Fatigue and Tiredness	49%	42%
Impaired Coordination	40%	18%
Irritability	33%	30%
Memory Impairment	33%	22%
Cognitive Disorder	29%	21%

Decreased Libido	14%	8%
Dysarthria	23%	6%
Confusional state	10%	8%
Increased libido	8%	4%
Change in libido (not specified)	7%	6%
Disinhibition	3%	2%
Talkativeness	2%	1%
Derealization	2%	1%
**Gastrointestinal disorders**		
Constipation	26%	15%
Increased salivation	6%	4%
**Skin and subcutaneous tissue disorders**		
Rash	11%	8%
**Other**		
Increased appetite	33%	23%
Decreased appetite	28%	24%
Weight gain	27%	18%
Weight loss	23%	17%
Micturition difficulties	12%	9%
Menstrual disorders	11%	9%
Sexual dysfunction	7%	4%
Incontinence	2%	1%

In addition to the reactions (i.e., greater than 1%) enumerated in the table above for patients with panic disorder, the following adverse reactions have been reported in association with the use of XANAX: seizures, hallucinations, depersonalization, taste alterations, diplopia, elevated bilirubin, elevated hepatic enzymes, and jaundice.

Adverse Reactions Reported as Reasons for Discontinuation in Treatment of Panic Disorder in Placebo-Controlled Trials

In a larger database comprised of both controlled and uncontrolled studies in which 641 patients received XANAX, discontinuation-emergent symptoms which occurred at a rate of over 5% in patients treated with XANAX and at a greater rate than the placebo-treated group are shown in Table 3.

Table 3: Discontinuation-Emergent Symptom Incidence Reported in ≥5% of XANAX-treated Patients and > Placebo-treated Patients

	**XANAX-treated Patients n=641**
**Nervous system disorders**	
Insomnia	29.5%
Light-headedness	19.3%

Abnormal involuntary movement	17.3%
Headache	17.0%
Muscular twitching	6.9%
Impaired coordination	6.6%
Muscle tone disorders	5.9%
Weakness	5.8%
**Psychiatric disorders**	
Anxiety	19.2%
Fatigue and Tiredness	18.4%
Irritability	10.5%
Cognitive disorder	10.3%
Memory impairment	5.5%
Depression	5.1%
Confusional state	5.0%
**Gastrointestinal disorders**	
Nausea/Vomiting	16.5%
Diarrhea	13.6%
Decreased salivation	10.6%
**Metabolism and nutrition disorders**	
Weight loss	13.3%
Decreased appetite	12.8%
**Dermatological disorders**	
Sweating	14.4%
**Cardiovascular disorders**	
Tachycardia	12.2%
**Special Senses**	
Blurred vision	10.0%
n=number of patients.	

There have also been reports of withdrawal seizures upon rapid decrease or abrupt discontinuation of XANAX *[see Warning and Precautions (5.2) and Drug Abuse and Dependence (9.3)]*.

Paradoxical reactions such as stimulation, increased muscle spasticity, sleep disturbances, hallucinations, and other adverse behavioral effects such as agitation, rage, irritability, and aggressive or hostile behavior have been reported rarely. In many of the spontaneous case reports of adverse behavioral effects, patients were receiving other CNS drugs concomitantly and/or were described as having underlying psychiatric conditions. Should any of the above events occur, alprazolam should be discontinued. Isolated published reports involving small numbers of patients have suggested that patients who have borderline personality disorder, a prior history of violent or aggressive behavior, or alcohol or substance abuse may be at risk for such events. Instances of irritability, hostility, and intrusive thoughts have been reported during discontinuation of alprazolam in patients with posttraumatic stress disorder.

6.2 Postmarketing Experience

The following adverse reactions have been identified during postapproval use of XANAX. Because these reactions are reported voluntarily from a population of uncertain size, it is not always possible to reliably estimate their frequency or establish a causal relationship to drug exposure.

Endocrine disorders: Hyperprolactinemia

General disorders and administration site conditions: Edema peripheral

Hepatobiliary disorders: Hepatitis, hepatic failure

Investigations: Liver enzyme elevations

Psychiatric disorders: Hypomania, mania

Reproductive system and breast disorders: Gynecomastia, galactorrhea

Skin and subcutaneous tissue disorders: Photosensitivity reaction, angioedema, Stevens-Johnson syndrome

7 DRUG INTERACTIONS

7.1 Drugs Having Clinically Important Interactions with XANAX

Table 4 includes clinically significant drug interactions with XANAX *[see Clinical Pharmacology (12.3)]*.

Table 4: Clinically Significant Drug Interactions with XANAX

**Opioids**	
Clinical implication	The concomitant use of benzodiazepines and opioids increases the risk of respiratory depression because of actions at different receptor sites in the CNS that control respiration. Benzodiazepines interact at gamma-aminobutyric acid ($GABA_A$) sites and opioids interact primarily at mu receptors. When benzodiazepines and opioids are combined, the potential for benzodiazepines to significantly worsen opioid-related respiratory depression exists.
Prevention or management	Limit dosage and duration of concomitant use of XANAX and opioids, and monitor patients closely for respiratory depression and sedation *[see Warnings and Precautions (5.1)]*.
Examples	Morphine, buprenorphine, hydromorphone, oxymorphone, oxycodone, fentanyl, methadone, alfentanil, butorphanol, codeine, dihydrocodeine, meperidine, pentazocine, remifentanil, sufentanil, tapentadol, tramadol.
**CNS Depressants**	
Clinical implication	The benzodiazepines, including alprazolam, produce additive CNS depressant effects when coadministered with other CNS depressants.
Prevention or management	Limit dosage and duration of XANAX during concomitant use with CNS depressants *[see Warnings and Precautions (5.3)]*.

Examples	Psychotropic medications, anticonvulsants, antihistaminics, ethanol, and other drugs which themselves produce CNS depression.
**Strong Inhibitors of CYP3A (except ritonavir)**	
Clinical implication	Concomitant use of XANAX with strong CYP3A inhibitors has a profound effect on the clearance of alprazolam, resulting in increased concentrations of alprazolam and increased risk of adverse reactions *[see Clinical Pharmacology (12.3)].*
Prevention or management	Concomitant use of XANAX with a strong CYP3A4 inhibitor (except ritonavir) is contraindicated *[see Contraindications (4), Warnings and Precautions (5.5)].*
Examples	Ketoconazole, itraconazole, clarithromycin
**Moderate or Weak Inhibitors of CYP3A**	
Clinical implication	Concomitant use of XANAX with CYP3A inhibitors may increase the concentrations of XANAX, resulting in increased risk of adverse reactions of alprazolam *[see Clinical Pharmacology (12.3)].*
Prevention or management	Avoid use and consider appropriate dose reduction when XANAX is coadministered with a moderate or weak CYP3A inhibitor *[see Warnings and Precautions (5.5)].*
Examples	Nefazodone, fluvoxamine, cimetidine, erythromycin
**CYP3A Inducers**	
Clinical implication	Concomitant use of CYP3A inducers can increase alprazolam metabolism and therefore can decease plasma levels of alprazolam *[see Clinical Pharmacology (12.3)]*.
Prevention or management	Caution is recommended during coadministration with XANAX.
Examples	Carbamazepine, phenytoin
**Ritonavir**	
Clinical implication	Interactions involving ritonavir and alprazolam are complex and time dependent. Short term administration of ritonavir increased alprazolam exposure due to CYP3A4 inhibition. Following long term treatment of ritonavir (>10 to 14 days), CYP3A4 induction offsets this inhibition.<br>Alprazolam exposure was not meaningfully affected in the presence of ritonavir.
Prevention or management	Reduce XANAX dosage when ritonavir and XANAX are initiated concomitantly, or when ritonavir is added to a regimen where XANAX is stabilized.<br>Increase XANAX dosage to the target dosage after 10 to 14 days of dosing ritonavir and XANAX concomitantly. No dosage adjustment of XANAX is necessary in patients receiving ritonavir for more than 10 to14 days *[see Dosage and Administration (2.6)]*.<br>Concomitant use of XANAX with a strong CYP3A inhibitor, except ritonavir, is contraindicated *[see Contraindications (4), Warnings and Precautions (5.5)].*
**Digoxin**	

Clinical implication	Increased digoxin concentrations have been reported when alprazolam was given, especially in geriatric patients (>65 years of age).
Prevention or management	In patients on digoxin therapy, measure serum digoxin concentrations before initiating XANAX. Continue monitoring digoxin serum concentration and toxicity frequently. Reduce the digoxin dose if necessary.

7.2 Drug/Laboratory Test Interactions

Although interactions between benzodiazepines and commonly employed clinical laboratory tests have occasionally been reported, there is no consistent pattern for a specific drug or specific test.

8 USE IN SPECIFIC POPULATIONS

8.1 Pregnancy

Pregnancy Exposure Registry

There is a pregnancy exposure registry that monitors pregnancy outcomes in women exposed to psychiatric medications, including XANAX, during pregnancy. Healthcare providers are encouraged to register patients by calling the National Pregnancy Registry for Psychiatric Medications at 1-866-961-2388 or visiting online at https://womensmentalhealth.org/research/pregnancyregistry/.

Risk Summary

Neonates born to mothers using benzodiazepines late in pregnancy have been reported to experience symptoms of sedation and/or neonatal withdrawal *[see Warnings and Precautions (5.8) and Clinical Considerations)]*. Available data from published observational studies of pregnant women exposed to benzodiazepines do not report a clear association with benzodiazepines and major birth defects (*see Data*).

The background risk of major birth defects and miscarriage for the indicated population is unknown. All pregnancies have a background risk of birth defect, loss, or other adverse outcomes. In the U.S. general population, the estimated risk of major birth defects and of miscarriage in clinically recognized pregnancies is 2% to 4% and 15% to 20%, respectively.

Clinical Considerations

Fetal/Neonatal adverse reactions

Benzodiazepines cross the placenta and may produce respiratory depression, hypotonia, and sedation in neonates. Monitor neonates exposed to XANAX during pregnancy or labor for signs of sedation, respiratory depression, hypotonia, and feeding problems. Monitor neonates exposed to XANAX during pregnancy for signs of withdrawal. Manage these neonates accordingly *[see Warnings and Precautions (5.8)]*.

Data

Human Data

Published data from observational studies on the use of benzodiazepines during pregnancy do not report a clear association with benzodiazepines and major birth defects. Although early studies reported an increased risk of congenital malformations with diazepam and chlordiazepoxide, there was no consistent pattern noted. In addition, the majority of recent case-control and cohort studies of benzodiazepine use during pregnancy, which were adjusted for confounding exposures to alcohol, tobacco, and other medications, have not confirmed these findings.

8.2 Lactation

Risk Summary

Limited data from published literature reports the presence of alprazolam in human breast milk. There are reports of sedation, poor feeding and poor weight gain in infants exposed to benzodiazepines through breast milk. The effects of alprazolam on lactation are unknown.

Because of the potential for serious adverse reactions, including sedation and withdrawal symptoms in breastfed infants, advise patients that breastfeeding is not recommended during treatment with XANAX.

8.4 Pediatric Use

Safety and effectiveness of XANAX have not been established in pediatric patients.

8.5 Geriatric Use

XANAX-treated geriatric patients had higher plasma concentrations of alprazolam (due to reduced clearance) compared to younger adult patients receiving the same doses. Therefore, dosage reduction of XANAX is recommended in geriatric patients *[see Dosage and Administration (2.4) and Clinical Pharmacology (12.3)]*.

8.6 Hepatic Impairment

Patients with alcoholic liver disease exhibit a longer elimination half-life (19.7 hours), compared to healthy subjects (11.4 hours). This may be caused by decreased clearance of alprazolam in patients with alcoholic liver disease. Dosage reduction of XANAX is recommended in patients with hepatic impairment *[see Dosage and Administration (2.4), Clinical Pharmacology (12.3)]*.

9 DRUG ABUSE AND DEPENDENCE

9.1 Controlled Substance

XANAX contains alprazolam, which is a Schedule IV controlled substance.

9.2 Abuse

XANAX is a benzodiazepine and a CNS depressant with a potential for abuse and addiction. Abuse is the intentional, non-therapeutic use of a drug, even once, for its desirable psychological or physiological effects. Misuse is the intentional use, for therapeutic purposes, of a drug by an individual in a way other than prescribed by a health care provider or for whom it was not prescribed. Drug addiction is a cluster of behavioral, cognitive, and physiological phenomena that may include a strong desire to take the drug, difficulties in controlling drug use (e.g., continuing drug use despite harmful consequences, giving a higher priority to drug use than other activities and obligations), and possible tolerance or physical dependence. Even taking benzodiazepines as prescribed may put patients at risk for abuse and misuse of their medication. Abuse and misuse of benzodiazepines may lead to addiction.

Abuse and misuse of benzodiazepines often (but not always) involve the use of doses greater than the maximum recommended dosage and commonly involve concomitant use of other medications, alcohol, and/or illicit substances, which is associated with an increased frequency of serious adverse outcomes, including respiratory depression, overdose, or death. Benzodiazepines are often sought by individuals who abuse drugs and other substances, and by individuals with addictive disorders *[see Warnings and Precautions (5.2)]*.

The following adverse reactions have occurred with benzodiazepine abuse and/or misuse: abdominal pain, amnesia, anorexia, anxiety, aggression, ataxia, blurred vision, confusion, depression, disinhibition, disorientation, dizziness, euphoria, impaired concentration and memory, indigestion, irritability, muscle pain, slurred speech, tremors, and vertigo.

The following severe adverse reactions have occurred with benzodiazepine abuse and/or misuse: delirium, paranoia, suicidal ideation and behavior, seizures, coma, breathing difficulty, and death. Death is more often associated with polysubstance use (especially benzodiazepines with other CNS depressants such as opioids and alcohol).

9.3 Dependence

XANAX may produce physical dependence from continued therapy. Physical dependence is a state that develops as a result of physiological adaptation in response to repeated drug use, manifested by withdrawal signs and symptoms after abrupt discontinuation or a significant dose reduction of a drug. Abrupt discontinuation or rapid dosage reduction of benzodiazepines or administration of flumazenil, a benzodiazepine antagonist, may precipitate acute withdrawal reactions, including seizures, which can be life-threatening. Patients at an increased risk of withdrawal adverse reactions after benzodiazepine discontinuation or rapid dosage reduction include those who take higher dosages (i.e., higher and/or more frequent doses) and those who have had longer durations of use *[see Warnings and Precautions (5.3)]*.

To reduce the risk of withdrawal reactions, use a gradual taper to discontinue XANAX or reduce the dosage *[see Dosage and Administration (2.3), Warnings and Precautions (5.3)]*.

Acute Withdrawal Signs and Symptoms

Acute withdrawal signs and symptoms associated with benzodiazepines have included abnormal involuntary movements, anxiety, blurred vision, depersonalization, depression, derealization, dizziness, fatigue, gastrointestinal adverse reactions (e.g., nausea,

vomiting, diarrhea, weight loss, decreased appetite), headache, hyperacusis, hypertension, irritability, insomnia, memory impairment, muscle pain and stiffness, panic attacks, photophobia, restlessness, tachycardia, and tremor. More severe acute withdrawal signs and symptoms, including life-threatening reactions, have included catatonia, convulsions, delirium tremens, depression, hallucinations, mania, psychosis, seizures, and suicidality.

Protracted Withdrawal Syndrome

Protracted withdrawal syndrome associated with benzodiazepines is characterized by anxiety, cognitive impairment, depression, insomnia, formication, motor symptoms (e.g., weakness, tremor, muscle twitches), paresthesia, and tinnitus that persists beyond 4 to 6 weeks after initial benzodiazepine withdrawal. Protracted withdrawal symptoms may last weeks to more than 12 months. As a result, there may be difficulty in differentiating withdrawal symptoms from potential re-emergence or continuation of symptoms for which the benzodiazepine was being used.

Tolerance

Tolerance to XANAX may develop from continued therapy. Tolerance is a physiological state characterized by a reduced response to a drug after repeated administration (i.e., a higher dose of a drug is required to produce the same effect that was once obtained at a lower dose). Tolerance to the therapeutic effect of XANAX may develop; however, little tolerance develops to the amnestic reactions and other cognitive impairments caused by benzodiazepines.

10 OVERDOSAGE

Overdosage of benzodiazepines is characterized by central nervous system depression ranging from drowsiness to coma. In mild to moderate cases, symptoms can include drowsiness, confusion, dysarthria, lethargy, hypnotic state, diminished reflexes, ataxia, and hypotonia. Rarely, paradoxical or disinhibitory reactions (including agitation, irritability, impulsivity, violent behavior, confusion, restlessness, excitement, and talkativeness) may occur. In severe overdosage cases, patients may develop respiratory depression and coma. Overdosage of benzodiazepines in combination with other CNS depressants (including alcohol and opioids) may be fatal *[see Warnings and Precautions (5.2)]*. Markedly abnormal (lowered or elevated) blood pressure, heart rate, or respiratory rate raise the concern that additional drugs and/or alcohol are involved in the overdosage.

In managing benzodiazepine overdosage, employ general supportive measures, including intravenous fluids and airway management. Flumazenil, a specific benzodiazepine receptor antagonist indicated for the complete or partial reversal of the sedative effects of benzodiazepines in the management of benzodiazepine overdosage, can lead to withdrawal and adverse reactions, including seizures, particularly in the context of mixed overdosage with drugs that increase seizure risk (e.g., tricyclic and tetracyclic antidepressants) and in patients with long-term benzodiazepine use and physical dependency. The risk of withdrawal seizures with flumazenil use may be increased in patients with epilepsy. Flumazenil is contraindicated in patients who have received a benzodiazepine for control of a potentially life-threatening condition (e.g., status epilepticus). If the decision is made to use flumazenil, it should be used as an adjunct to, not as a substitute for, supportive management of benzodiazepine

overdosage. See the flumazenil injection Prescribing Information.

Consider contacting the Poison Help Line (1-800-222-1222), or a medical toxicologist for additional overdosage management recommendations.

11 DESCRIPTION

XANAX contains alprazolam which is a triazolo analog of the 1,4 benzodiazepine class of central nervous system-active compounds.

The chemical name of alprazolam is 8-Chloro-1-methyl-6-phenyl-4H-s-triazolo [4,3-α] [1,4] benzodiazepine.

The structural formula is:

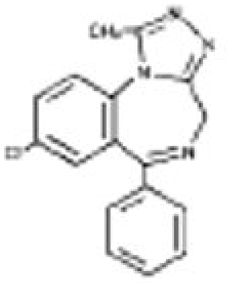

Alprazolam is a white crystalline powder, which is soluble in methanol or ethanol but which has no appreciable solubility in water at physiological pH.

Each XANAX tablet, for oral administration, contains 0.25 mg, 0.5 mg, 1 mg, or 2 mg of alprazolam.

Inactive ingredients: cellulose, corn starch, docusate sodium, lactose, magnesium stearate, silicon dioxide and sodium benzoate. In addition, the 0.5 mg tablet contains FD&C Yellow No. 6 and the 1 mg tablet contains FD&C Blue No. 2.

12 CLINICAL PHARMACOLOGY

12.1 Mechanism of Action

Alprazolam is a 1,4 benzodiazepine. Alprazolam exerts its effect for the acute treatment of generalized anxiety disorder and panic disorder through binding to the benzodiazepine site of gamma-aminobutyric acid-A ($GABA_A$) receptors in the brain and enhances GABA-mediated synaptic inhibition.

12.3 Pharmacokinetics

Plasma levels of alprazolam increase proportionally to the dose over the range of 0.5 to 3.0 mg.

Absorption

Following oral administration, peak plasma concentration of alprazolam (C_{max}) occurs in 1 to 2 hours post dose.

Distribution

Alprazolam is 80% bound to human serum protein, and albumin accounts for the majority of the binding.

Elimination

The mean plasma elimination half-life ($T_{1/2}$) of alprazolam is approximately 11.2 hours (range: 6.3 to 26.9 hours) in healthy adults.

Metabolism

Alprazolam is extensively metabolized in humans, primarily by cytochrome P450 3A4 (CYP3A4), to 2 major active metabolites in the plasma: 4-hydroxyalprazolam and α-hydroxyalprazolam. The plasma circulation levels of the two active metabolites are less than 4% of the parent. The reported relative potencies in benzodiazepine receptor binding experiments and in animal models of induced seizure inhibition are 0.20 and 0.66, respectively, for 4-hydroxyalprazolam and α-hydroxyalprazolam. The low concentrations and low potencies of 4-hydroxyalprazolam and α-hydroxyalprazolam indicate that they unlikely contribute much to the effects of alprazolam. A benzophenone derived from alprazolam is also found in humans. Their half-lives appear to be similar to that of alprazolam.

Excretion

Alprazolam and its metabolites are excreted primarily in the urine.

Specific Populations

Geriatric Patients

The mean $T_{1/2}$ of alprazolam was 16.3 hours (range: 9.0 to 26.9 hours) in healthy elderly subjects compared to 11.0 hours (range: 6.3 to -15.8 hours, n=16) in healthy younger adult subjects.

Obese Patients

The mean $T_{1/2}$ of alprazolam was 21.8 hours (range: 9.9 to 40.4 hours) in a group of obese subjects.

Patients with Hepatic Impairment

The mean $T_{1/2}$ of alprazolam was 19.7 hours (range: 5.8 to 65.3 hours) in patients with alcoholic liver disease.

Racial or Ethnic Groups

Maximal concentrations and $T_{1/2}$ of alprazolam are approximately 15% and 25% higher in Asians compared to Caucasians.

Smoking

Alprazolam concentrations may be reduced by up to 50% in smokers compared to non-smokers.

Drug Interaction Studies

In Vivo Studies

Most of the interactions that have been documented with alprazolam are with drugs that modulate CYP3A4 activity.

Compounds that are inhibitors or inducers of CYP3A would be expected to increase or decrease plasma alprazolam concentrations, respectively. Drug products that have been studied in vivo, along with their effect on increasing alprazolam AUC, are as follows: ketoconazole, 3.98 fold; itraconazole, 2.66 fold; nefazodone, 1.98 fold; fluvoxamine, 1.96 fold; and erythromycin, 1.61 fold *[see Contraindications (4), Warnings and Precautions (5.5), Drug Interactions (7.2)]*. Other studied drugs include:

Cimetidine: Coadministration of cimetidine increased the maximum plasma concentration of alprazolam by 82%, decreased clearance by 42%, and increased $T_{1/2}$ by 16%.

Fluoxetine: Coadministration of fluoxetine with alprazolam increased the maximum plasma concentration of alprazolam by 46%, decreased clearance by 21%, increased $T_{1/2}$ by 17%, and decreased measured psychomotor performance.

Oral Contraceptives: Coadministration of oral contraceptives increased the maximum plasma concentration of alprazolam by 18%, decreased clearance by 22%, and increased $T_{1/2}$ by 29%.

Carbamazepine: The oral clearance of alprazolam (given in a 0.8 mg single dose) was increased from 0.90±0.21 mL/min/kg to 2.13±0.54 mL/min/kg and the elimination $T_{1/2}$ was shortened (from 17.1±4.9 to 7.7±1.7 hour) following administration of 300 mg per day carbamazepine for 10 days *[see Drug Interactions (7.2)]*. However, the carbamazepine dose used in this study was fairly low compared to the recommended doses (1000-1200 mg per day); the effect at usual carbamazepine doses is unknown.

Ritonavir: Interactions involving HIV protease inhibitors (e.g., ritonavir) and alprazolam are complex and time dependent. Short-term low doses of ritonavir (4 doses of 200 mg) increased mean AUC of alprazolam by about 2.5-fold, and did not significantly affect C_{max} of alprazolam. The elimination $T_{1/2}$ was prolonged (30 hours versus 13 hours). However, upon extended exposure to ritonavir (500 mg, twice daily for 10 days), CYP3A induction offset this inhibition. Alprazolam AUC and C_{max} was reduced by 12% and 16%, respectively, in the presence of ritonavir. The elimination $T_{1/2}$ of alprazolam was not significantly changed *[see Warnings and Precautions (5.5)]*.

Sertraline: A single dose of alprazolam 1 mg and steady state dose of sertraline (50 mg to 150 mg per day) did not reveal any clinically significant changes in the pharmacokinetics of alprazolam.

Imipramine and Desipramine: The steady state plasma concentrations of imipramine and desipramine have been reported to be increased an average of 31% and 20%, respectively, by the concomitant administration of XANAX in doses up to 4 mg per day.

Warfarin: Alprazolam did not affect the prothrombin or plasma warfarin levels in male volunteers administered sodium warfarin orally.

In Vitro Studies

Data from in vitro studies of alprazolam suggest a possible drug interaction of alprazolam with paroxetine. The ability of alprazolam to induce human hepatic enzyme systems has not yet been determined.

13 NONCLINICAL TOXICOLOGY

13.1 Carcinogenesis, Mutagenesis, Impairment of Fertility

Carcinogenesis

No evidence of carcinogenic potential was observed in rats or mice administered alprazolam for 2 years at doses up to 30 and 10 mg/kg day respectively. These doses are 29 times and 4.8 times the maximum recommended human dose of 10 mg/day based on mg/m^2 body surface area, respectively.

Mutagenesis

Alprazolam was negative in the in vitro Ames bacterial reverse mutation assay and DNA Damage/Alkaline Elution Assay and in vivo rat micronucleus genetic toxicology assays.

Impairment of Fertility

Alprazolam produced no impairment of fertility in rats at doses up to 5 mg/kg per day, which is approximately 5 times the maximum recommended human dose of 10 mg per day based on mg/m^2 body surface area.

13.2 Animal Toxicology and/or Pharmacology

When rats were treated with alprazolam at oral doses of 3 mg, 10 mg, and 30 mg/kg day (3 to 29 times the maximum recommended human dose based on mg/m^2 body surface area) for 2 years, a tendency for a dose related increase in the number of cataracts was observed in females and a tendency for a dose related increase in corneal vascularization was observed in males. These lesions did not appear until after 11 months of treatment.

14 CLINICAL STUDIES

14.1 Generalized Anxiety Disorder

XANAX was compared to placebo in double-blind clinical studies (doses up to 4 mg per day) in patients with a diagnosis of anxiety or anxiety with associated depressive symptomatology. XANAX was significantly better than placebo at each of the evaluation periods of these 4-week studies as judged by the following psychometric instruments: Physician’s Global Impressions, Hamilton Anxiety Rating Scale, Target Symptoms, Patient’s Global Impressions, and Self-Rating Symptom Scale.

14.2 Panic Disorder

The effectiveness of XANAX in the treatment of panic disorder was studied in 3 short-term, placebo-controlled studies (up to 10 weeks) in patients with diagnoses closely

corresponding to DSM-III-R criteria for panic disorder.

The average dose of XANAX was 5 mg to 6 mg per day in 2 of the studies, and the doses of XANAX were fixed at 2 mg and 6 mg per day in the third study. In all 3 studies, XANAX was superior to placebo on a variable defined as “the number of patients with zero panic attacks” (range, 37% to 83% met this criterion), as well as on a global improvement score. In 2 of the 3 studies, XANAX was superior to placebo on a variable defined as “change from baseline on the number of panic attacks per week” (range, 3.3 to 5.2), and also on a phobia rating scale. A subgroup of patients who improved on XANAX during short-term treatment in 1 of these trials was continued on an open basis up to 8 months, without apparent loss of benefit.

16 HOW SUPPLIED/STORAGE AND HANDLING

XANAX is supplied in the following strengths and package configurations:

XANAX Tablets

Package Configuration	Tablet Strength (mg)	NDC	Print
Bottles of 100<br>Reverse Numbered Unit dose (100)<br>Bottles of 500<br>Bottles of 1000	0.25 mg	NDC 0009-0029-01<br>NDC 0009-0029-46<br>NDC 0009-0029-02<br>NDC 0009-0029-14	white, oval, scored, imprinted “XANAX 0.25”
Bottles of 100<br>Reverse Numbered Unit dose (100)<br>Bottles of 500<br>Bottles of 1000	0.5 mg	NDC 0009-0055-01<br>NDC 0009-0055-46<br>NDC 0009-0055-03<br>NDC 0009-0055-15	peach, oval, scored, imprinted “XANAX 0.5”
Bottles of 100<br>Bottles of 500<br>Bottles of 1000	1 mg	NDC 0009-0090-01<br>NDC 0009-0090-04<br>NDC 0009-0090-13	blue, oval, scored, imprinted “XANAX 1.0”
Bottles of 100<br>Bottles of 500	2 mg	NDC 0009-0094-01<br>NDC 0009-0094-03	white, oblong, multi-scored, imprinted “XANAX” on one side and “2” on the reverse side

Store at controlled room temperature 20° to 25°C (68° to 77°F) [see USP Controlled Room Temperature].

17 PATIENT COUNSELING INFORMATION

Advise the patient to read the FDA-approved patient labeling (Medication Guide).

Risks from Concomitant Use with Opioids

Advise both patients and caregivers about the risks of potentially fatal respiratory depression and sedation when XANAX is used with opioids and not to use such drugs concomitantly unless supervised by a healthcare provider. Advise patients not to drive or operate heavy machinery until the effects of concomitant use with the opioid have been determined *[see Warnings and Precautions (5.1), Drug Interactions (7.1)]*.

Abuse, Misuse, and Addiction

Inform patients that the use of XANAX, even at recommended dosages, exposes users to risks of abuse, misuse, and addiction, which can lead to overdose and death, especially when used in combination with other medications (e.g., opioid analgesics), alcohol, and/or illicit substances. Inform patients about the signs and symptoms of benzodiazepine abuse, misuse, and addiction; to seek medical help if they develop these signs and/or symptoms; and on the proper disposal of unused drug *[see Warnings and Precautions (5.2), Drug Abuse and Dependence (9.2)]*.

Withdrawal Reactions

Inform patients that the continued use of XANAX may lead to clinically significant physical dependence and that abrupt discontinuation or rapid dosage reduction of XANAX may precipitate acute withdrawal reactions, which can be life-threatening. Inform patients that in some cases, patients taking benzodiazepines have developed a protracted withdrawal syndrome with withdrawal symptoms lasting weeks to more than 12 months. Instruct patients that discontinuation or dosage reduction of XANAX may require a slow taper *[see Warnings and Precautions (5.3), Drug Abuse and Dependence (9.3)]*.

Effects on Driving and Operating Machinery

Advise patients not to drive a motor vehicle or operate heavy machinery while taking XANAX due to its CNS depressant effects. Also advise patients to avoid use of alcohol or other CNS depressants while taking XANAX *[see Warnings and Precautions (5.3)]*.

Patients with Depression

Advise patients, their families, and caregivers to look for signs of suicidality or worsening depression, and to inform the patient’s healthcare provider immediately *[see Warnings and Precautions (5.6)]*.

Concomitant Medications

Advise patients to inform their healthcare provider of all medicines they take, including prescription and nonprescription medications, vitamins and herbal supplements *[see Drug Interactions (7)]*.

Pregnancy

Advise pregnant females that use of XANAX late in pregnancy can result in sedation (respiratory depression, lethargy, hypotonia) and/or withdrawal symptoms (hyperreflexia, irritability, restlessness, tremors, inconsolable crying, and feeding difficulties) in newborns *[see Warnings and Precautions (5.8), Use in Specific Populations (8.1)]*. Instruct patients to inform their healthcare provider if they are pregnant.

Advise patients that there is a pregnancy exposure registry that monitors pregnancy outcomes in women exposed to XANAX during pregnancy *[see Use in Specific Populations (8.1)]*.

<u>Lactation</u>

Advise patients that breastfeeding is not recommended during treatment with XANAX *[see Use in Specific Populations (8.2)]*.

Distributed by:
Viatris Specialty LLC
Morgantown, WV 26505 U.S.A.

UPJ:XNXT:RX2

Medication Guide

**XANAX (ZAN-aks)**<br>**(alprazolam) tablets, C-IV**
**What is the most important information I should know about XANAX?**<br><br>• **XANAX is a benzodiazepine medicine. Taking benzodiazepines with opioid medicines, alcohol, or other central nervous system (CNS) depressants (including street drugs) can cause severe drowsiness, breathing problems (respiratory depression), coma and death.** Get emergency help right away if any of the following happens:<br>o shallow or slowed breathing<br>o breathing stops (which may lead to the heart stopping)<br>o excessive sleepiness (sedation)<br><br>Do not drive or operate heavy machinery until you know how taking XANAX with opioids affects you.<br><br>• **Risk of abuse, misuse, and addiction.** There is a risk of abuse, misuse, and addiction with benzodiazepines, including XANAX, which can lead to overdose and serious side effects including coma and death.<br>o **Serious side effects including coma and death have happened in people who have abused or misused benzodiazepines, including XANAX.** These serious side effects may also include delirium, paranoia, suicidal thoughts or actions, seizures, and difficulty breathing. **Call your healthcare provider or go to the nearest hospital emergency room right away if you get any of these serious side effects.**<br>o **You can develop an addiction even if you take XANAX as prescribed by your healthcare provider.**

- **Take XANAX exactly as your healthcare provider prescribed.**
- Do not share your XANAX with other people.
- Keep XANAX in a safe place and away from children.

- **Physical dependence and withdrawal reactions.** XANAX can cause physical dependence and withdrawal reactions.
  - **Do not suddenly stop taking XANAX.** Stopping XANAX suddenly can cause serious and life-threatening side effects, including, unusual movements, responses, or expressions, seizures, sudden and severe mental or nervous system changes, depression, seeing or hearing things that others do not see or hear, an extreme increase in activity or talking, losing touch with reality, and suicidal thoughts or actions. **Call your healthcare provider or go to the nearest hospital emergency room right away if you get any of these symptoms.**
  - **Some people who suddenly stop benzodiazepines, have symptoms that can last for several weeks to more than 12 months**, including, anxiety, trouble remembering, learning, or concentrating, depression, problems sleeping, feeling like insects are crawling under your skin, weakness, shaking, muscle twitching, burning or prickling feeling in your hands, arms, legs or feet, and ringing in your ears.
  - Physical dependence is not the same as drug addiction. Your healthcare provider can tell you more about the differences between physical dependence and drug addiction.

- Do not take more XANAX than prescribed or take XANAX for longer than prescribed.

What is XANAX?

- XANAX is a prescription medicine used:
  - to treat anxiety disorders
  - for the short-term relief of the symptoms of anxiety
  - to treat panic disorder with or without a fear of places and situations that might cause panic, helplessness, or embarrassment (agoraphobia)

- **XANAX is a federal controlled substance (C-IV) because it contains alprazolam that can be abused or lead to dependence.** Keep XANAX in a safe place to prevent misuse and abuse. Selling or giving away XANAX may harm others, and is against the law. Tell your healthcare provider if you have abused or been dependent on alcohol, prescription medicines or street drugs.
- It is not known if XANAX is safe and effective in children.
- Elderly patients are especially susceptible to dose related adverse effects when taking XANAX.
- It is not known if XANAX is safe and effective when used to treat anxiety disorder for longer than 4 months.
- It is not known if XANAX is safe and effective when used to treat panic disorder for longer than 10 weeks.

Do not take XANAX if:

- you are allergic to alprazolam, other benzodiazepines, or any of the ingredients in XANAX. See the end of this Medication Guide for a complete list of ingredients in XANAX.
- you are taking antifungal medicines including ketoconazole and itraconazole

Before you take XANAX, tell your healthcare provider about all of your medical conditions, including if you:

- have or have had depression, mood problems, or suicidal thoughts or behavior
- have liver or kidney problems
- have lung disease or breathing problems
- are pregnant or plan to become pregnant.
  - Taking XANAX late in pregnancy may cause your baby to have symptoms of sedation (breathing problems, sluggishness, low muscle tone), and/or withdrawal symptoms (jitteriness, irritability, restlessness, shaking, excessive crying, feeding problems).
  - Tell your healthcare provider right away if you become pregnant or think you are pregnant during treatment with XANAX.
  - There is a pregnancy registry for women who take XANAX during pregnancy. The purpose of the registry is to collect information about the health of you and your baby. If you become pregnant during treatment with XANAX, talk to your healthcare provider about registering with the National Pregnancy Registry for Psychiatric Medications. You can register by calling 1-866-961-2388 or visiting https://womensmentalhealth.org/pregnancyregistry/.
- are breastfeeding or plan to breastfeed. XANAX passes into your breast milk.
  - Talk to your healthcare provider about the best way to feed your baby if you take XANAX.
  - Breastfeeding is not recommended during treatment with XANAX.

Tell your healthcare provider about all the medicines you take, including prescription and over-the-counter medicines, vitamins, and herbal supplements. Taking XANAX with certain other medicines can cause side effects or affect how well XANAX or the other medicines work. Do not start or stop other medicines without talking to your healthcare provider.

How should I take XANAX?

- See "What is the most important information I should know about XANAX?"
- Take XANAX exactly as your healthcare provider tells you to take it. Your healthcare provider will tell you how much XANAX to take and when to take it.
- If you take too much XANAX, call your healthcare provider or go to the nearest hospital emergency room right away.

What are the possible side effects of XANAX?
XANAX may cause serious side effects, including:

- See "What is the most important information I should know about XANAX?"
- **Seizures.** Stopping XANAX can cause seizures and seizures that will not stop (status epilepticus).

- **Mania.** XANAX may cause an increase in activity and talking (hypomania and mania) in people who have depression.
    - o **XANAX can make you sleepy or dizzy and can slow your thinking and motor skills.** Do not drive, operate heavy machinery, or do other dangerous activities until you know how XANAX affects you.
    - o **Do not drink alcohol or take other drugs that may make you sleepy or dizzy while taking XANAX without first talking to your healthcare provider.** When taken with alcohol or drugs that cause sleepiness or dizziness, XANAX may make your sleepiness or dizziness much worse.

The most common side effects of XANAX include:

- problems with coordination
- hypotension
- trouble saying words clearly (dysarthria)
- changes in sex drive (libido)

These are not all the possible side effects of XANAX. Call your doctor for medical advice about side effects. You may report side effects to FDA at 1-800-FDA-1088.

How should I store XANAX?

- Store XANAX at room temperature between 68°F to 77°F (20°C to 25°C)
- **Keep XANAX and all medicines out of the reach of children.**

General information about the safe and effective use of XANAX.

- Medicines are sometimes prescribed for purposes other than those listed in a Medication Guide.
- Do not use XANAX for a condition for which it was not prescribed.
- Do not give XANAX to other people, even if they have the same symptoms that you have. It may harm them.
- You can ask your pharmacist or healthcare provider for information about XANAX that is written for health professionals.

What are the ingredients in XANAX?
Active ingredient: alprazolam
Inactive ingredients: Cellulose, corn starch, docusate sodium, lactose, magnesium stearate, silicon dioxide and sodium benzoate. In addition, the 0.5 mg tablet contains FD&C Yellow No. 6 and the 1 mg tablet contains FD&C Blue No. 2.
XANAX® is a registered trademark of UPJOHN US 2 LLC, a Viatris Company.
For more information, call Viatris at 1-877-446-3679 (1-877-4-INFO-RX).
Distributed by:
Viatris Specialty LLC
Morgantown, WV 26505 U.S.A.
UPJ:MG:XNXT:RX2

This Medication Guide has been approved by the U.S. Food and Drug Administration. Revised: 1/2023

PRINCIPAL DISPLAY PANEL - 0.25 mg

ALWAYS DISPENSE WITH MEDICATION GUIDE

NDC 0009-0029-01

Pfizer

Xanax®

alprazolam
tablets, USP

CIV

0.25 mg

100 Tablets **Rx only**

Store at controlled room temperature, 20° to 25°C (68° to 77°F) [see USP].

Protect from light.

Dispense in tight (USP), light-resistant, child-resistant containers.

DOSAGE AND USE:
See accompanying prescribing information.

Each tablet contains 0.25 mg alprazolam.

Distributed by
Pharmacia & Upjohn Co
Division of Pfizer Inc,
NY, NY 10017

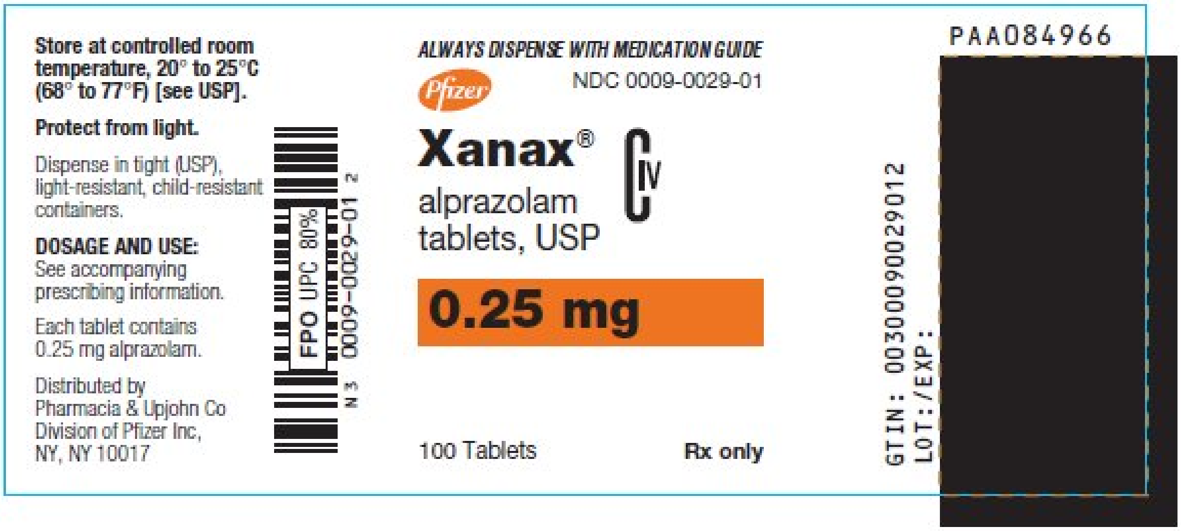

PRINCIPAL DISPLAY PANEL - 0.5 mg

ALWAYS DISPENSE WITH MEDICATION GUIDE

NDC 0009-0055-01

Pfizer

Xanax®

alprazolam
tablets, USP

CIV

0.5 mg

100 Tablets **Rx only**

Store at controlled room temperature, 20° to 25°C (68° to 77°F) [see USP].

Protect from light.

Dispense in tight (USP), light-resistant, child-resistant containers.

DOSAGE AND USE:
See accompanying prescribing information.

Each tablet contains
0.5 mg alprazolam.

Distributed by
Pharmacia & Upjohn Co
Division of Pfizer Inc,
NY, NY 10017

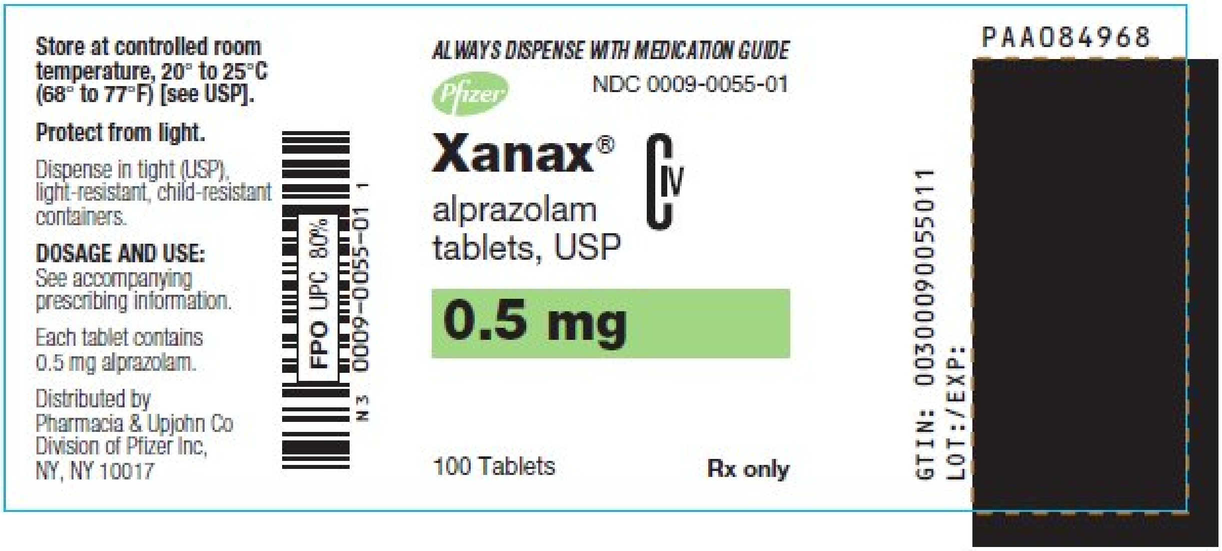

PRINCIPAL DISPLAY PANEL - 1 mg

ALWAYS DISPENSE WITH MEDICATION GUIDE

NDC 0009-0090-01

Pfizer

Xanax®

alprazolam
tablets, USP

CIV

1 mg

100 Tablets **Rx only**

Store at controlled room temperature, 20° to 25°C (68° to 77°F) [see USP].

Protect from light.

Dispense in tight (USP), light-resistant, child-resistant containers.

DOSAGE AND USE:
See accompanying prescribing information.

Each tablet contains
1 mg alprazolam.

Distributed by
Pharmacia & Upjohn Co
Division of Pfizer Inc,
NY, NY 10017

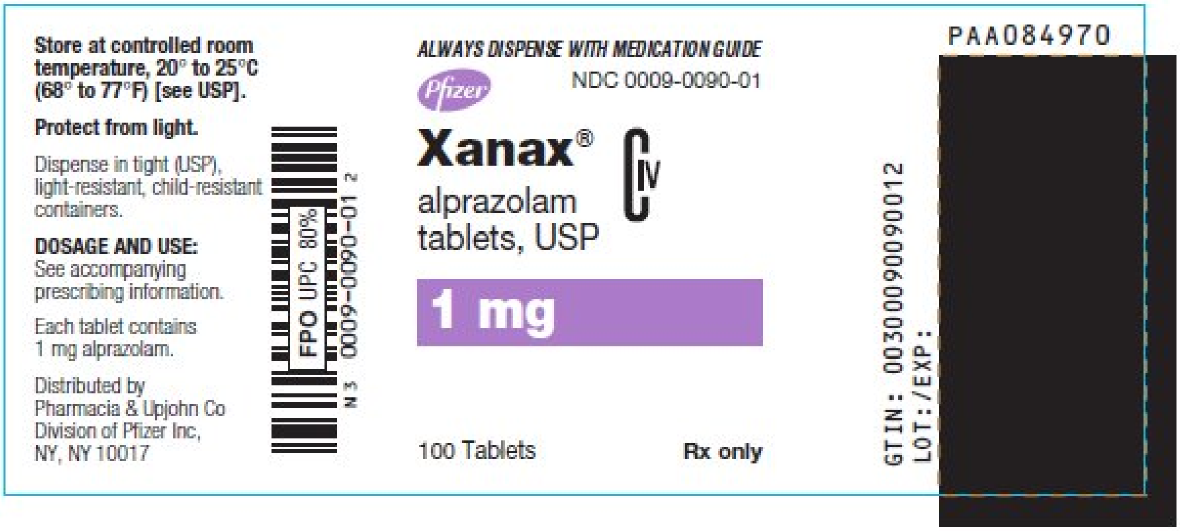

PRINCIPAL DISPLAY PANEL - 2 mg

ALWAYS DISPENSE WITH MEDICATION GUIDE

NDC 0009-0094-01

Pfizer

Xanax®

alprazolam
tablets, USP

CIV

2 mg

100 Tablets **Rx only**

Store at controlled room temperature, 20° to 25°C (68° to 77°F) [see USP].

Protect from light.

Dispense in tight (USP), light-resistant, child-resistant containers.

DOSAGE AND USE:
See accompanying prescribing information.

Each tablet contains
2 mg alprazolam.

Distributed by
Pharmacia & Upjohn Co
Division of Pfizer Inc,

NY, NY 10017

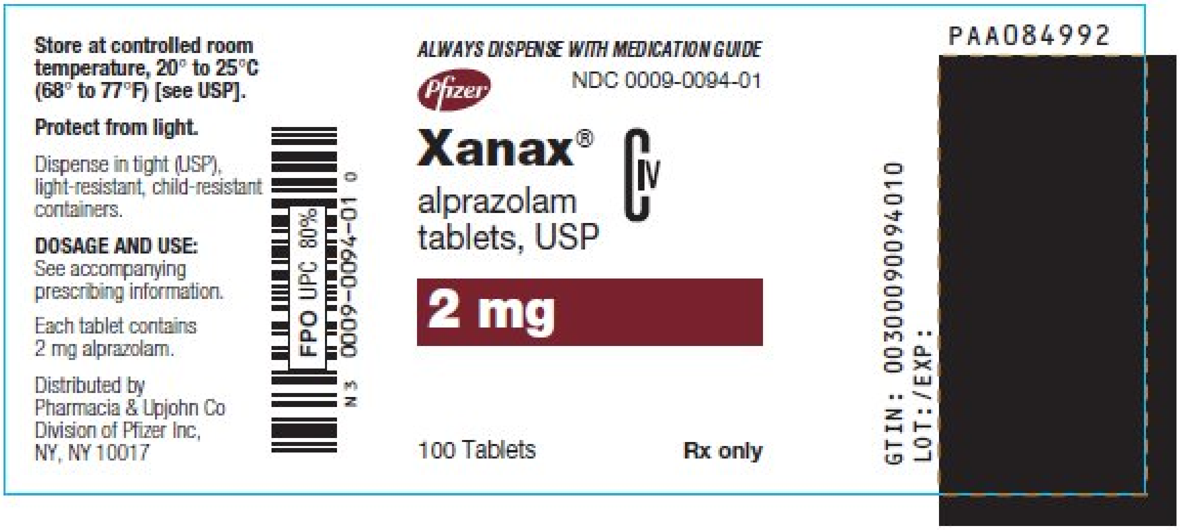

XANAX

alprazolam tablet

Product Information			
**Product Type**	HUMAN PRESCRIPTION DRUG	**Item Code (Source)**	NDC:0009-0029
**Route of Administration**	ORAL	**DEA Schedule**	CIV

Active Ingredient/Active Moiety		
**Ingredient Name**	**Basis of Strength**	**Strength**
**ALPRAZOLAM** (UNII: YU55MQ3IZY) (ALPRAZOLAM - UNII:YU55MQ3IZY)	ALPRAZOLAM	0.25 mg

Inactive Ingredients	
**Ingredient Name**	**Strength**
**POWDERED CELLULOSE** (UNII: SMD1X3XO9M)	
**STARCH, CORN** (UNII: O8232NY3SJ)	
**DOCUSATE SODIUM** (UNII: F05Q2T2JA0)	
**LACTOSE, UNSPECIFIED FORM** (UNII: J2B2A4N98G)	
**MAGNESIUM STEARATE** (UNII: 70097M6I30)	
**SILICON DIOXIDE** (UNII: ETJ7Z6XBU4)	
**SODIUM BENZOATE** (UNII: OJ245FE5EU)	

Product Characteristics			
**Color**	WHITE	**Score**	2 pieces
**Shape**	OVAL	**Size**	9mm
**Flavor**		**Imprint Code**	XANAX;0;25

Contains	

Packaging

#	Item Code	Package Description	Marketing Start Date	Marketing End Date
1	NDC:0009-0029-01	100 in 1 BOTTLE; Type 0: Not a Combination Product	10/16/1981	
2	NDC:0009-0029-46	100 in 1 BOTTLE, UNIT-DOSE; Type 0: Not a Combination Product	10/16/1981	01/01/2006
3	NDC:0009-0029-02	500 in 1 BOTTLE; Type 0: Not a Combination Product	10/16/1981	
4	NDC:0009-0029-14	1000 in 1 BOTTLE; Type 0: Not a Combination Product	10/16/1981	05/05/2004

Marketing Information

Marketing Category	Application Number or Monograph Citation	Marketing Start Date	Marketing End Date
NDA	NDA018276	10/16/1981	

XANAX

alprazolam tablet

Product Information

**Product Type**	HUMAN PRESCRIPTION DRUG	**Item Code (Source)**	NDC:0009-0055
**Route of Administration**	ORAL	**DEA Schedule**	CIV

Active Ingredient/Active Moiety

Ingredient Name	Basis of Strength	Strength
**ALPRAZOLAM** (UNII: YU55MQ3IZY) (ALPRAZOLAM - UNII:YU55MQ3IZY)	ALPRAZOLAM	0.5 mg

Inactive Ingredients

Ingredient Name	Strength
**POWDERED CELLULOSE** (UNII: SMD1X3XO9M)	
**STARCH, CORN** (UNII: O8232NY3SJ)	
**DOCUSATE SODIUM** (UNII: F05Q2T2JA0)	
**LACTOSE, UNSPECIFIED FORM** (UNII: J2B2A4N98G)	
**MAGNESIUM STEARATE** (UNII: 70097M6I30)	
**SILICON DIOXIDE** (UNII: ETJ7Z6XBU4)	
**SODIUM BENZOATE** (UNII: OJ245FE5EU)	
**FD&C YELLOW NO. 6** (UNII: H77VEI93A8)	

Product Characteristics

**Color**	ORANGE (peach)	**Score**	2 pieces
**Shape**	OVAL	**Size**	9mm
**Flavor**		**Imprint Code**	XANAX;0;5
**Contains**			

Packaging

#	Item Code	Package Description	Marketing Start Date	Marketing End Date
1	NDC:0009-0055-01	100 in 1 BOTTLE; Type 0: Not a Combination Product	10/16/1981	
2	NDC:0009-0055-46	100 in 1 BOTTLE, UNIT-DOSE; Type 0: Not a Combination Product	10/16/1981	01/01/2006
3	NDC:0009-0055-03	500 in 1 BOTTLE; Type 0: Not a Combination Product	10/16/1981	
4	NDC:0009-0055-15	1000 in 1 BOTTLE; Type 0: Not a Combination Product	10/16/1981	05/05/2004

Marketing Information

Marketing Category	Application Number or Monograph Citation	Marketing Start Date	Marketing End Date
NDA	NDA018276	10/16/1981	

XANAX

alprazolam tablet

Product Information

**Product Type**	HUMAN PRESCRIPTION DRUG	**Item Code (Source)**	NDC:0009-0090
**Route of Administration**	ORAL	**DEA Schedule**	CIV

Active Ingredient/Active Moiety

Ingredient Name	Basis of Strength	Strength
**ALPRAZOLAM** (UNII: YU55MQ3IZY) (ALPRAZOLAM - UNII:YU55MQ3IZY)	ALPRAZOLAM	1 mg

Inactive Ingredients

Ingredient Name	Strength
**POWDERED CELLULOSE** (UNII: SMD1X3XO9M)	
**STARCH, CORN** (UNII: O8232NY3SJ)	
**DOCUSATE SODIUM** (UNII: F05Q2T2JA0)	
**LACTOSE, UNSPECIFIED FORM** (UNII: J2B2A4N98G)	
**MAGNESIUM STEARATE** (UNII: 70097M6I30)	
**SILICON DIOXIDE** (UNII: ETJ7Z6XBU4)	
**SODIUM BENZOATE** (UNII: OJ245FE5EU)	
**FD&C BLUE NO. 2** (UNII: L06K8R7DQK)	

Product Characteristics

**Color**	BLUE	**Score**	2 pieces
**Shape**	OVAL	**Size**	9mm
**Flavor**		**Imprint Code**	XANAX;1;0
**Contains**			

Packaging

#	Item Code	Package Description	Marketing Start Date	Marketing End Date
1	NDC:0009-0090-01	100 in 1 BOTTLE; Type 0: Not a Combination Product	10/16/1981	
2	NDC:0009-0090-04	500 in 1 BOTTLE; Type 0: Not a Combination Product	10/16/1981	
3	NDC:0009-0090-13	1000 in 1 BOTTLE; Type 0: Not a Combination Product	10/16/1981	05/05/2004

Marketing Information

Marketing Category	Application Number or Monograph Citation	Marketing Start Date	Marketing End Date
NDA	NDA018276	10/16/1981	

XANAX

alprazolam tablet

Product Information

**Product Type**	HUMAN PRESCRIPTION DRUG	**Item Code (Source)**	NDC:0009-0094
**Route of Administration**	ORAL	**DEA Schedule**	CIV

Active Ingredient/Active Moiety

Ingredient Name	Basis of Strength	Strength
**ALPRAZOLAM** (UNII: YU55MQ3IZY) (ALPRAZOLAM - UNII:YU55MQ3IZY)	ALPRAZOLAM	2 mg

Inactive Ingredients

Ingredient Name	Strength
**POWDERED CELLULOSE** (UNII: SMD1X3XO9M)	
**STARCH, CORN** (UNII: O8232NY3SJ)	
**DOCUSATE SODIUM** (UNII: F05Q2T2JA0)	
**LACTOSE, UNSPECIFIED FORM** (UNII: J2B2A4N98G)	
**MAGNESIUM STEARATE** (UNII: 70097M6I30)	
**SILICON DIOXIDE** (UNII: ETJ7Z6XBU4)	
**SODIUM BENZOATE** (UNII: OJ245FE5EU)	

Product Characteristics

**Color**	WHITE	**Score**	4 pieces
**Shape**	OVAL (oblong)	**Size**	15mm
**Flavor**		**Imprint Code**	XANAX;2
**Contains**			

Packaging

#	Item Code	Package Description	Marketing Start Date	Marketing End Date
**1**	NDC:0009-0094-01	100 in 1 BOTTLE; Type 0: Not a Combination Product	10/16/1981	
**2**	NDC:0009-0094-03	500 in 1 BOTTLE; Type 0: Not a Combination Product	10/16/1981	02/16/2007

Marketing Information

Marketing Category	Application Number or Monograph Citation	Marketing Start Date	Marketing End Date
NDA	NDA018276	10/16/1981	

Labeler - PHARMACIA & UPJOHN COMPANY LLC (618054084)

ZOLOFT

ZOLOFT- sertraline hydrochloride tablet, film coated
ZOLOFT- sertraline hydrochloride solution, concentrate
ROERIG

HIGHLIGHTS OF PRESCRIBING INFORMATION
These highlights do not include all the information needed to use ZOLOFT safely and effectively. See full prescribing information for ZOLOFT.

ZOLOFT (sertraline hydrochloride) tablets, for oral use
ZOLOFT (sertraline hydrochloride) oral solution
Initial U.S. Approval: 1991

WARNING: SUICIDAL THOUGHTS AND BEHAVIORS
See full prescribing information for complete boxed warning.

- **Antidepressants increased the risk of suicidal thoughts and behaviors in pediatric and young adult patients (5.1)**
- **Closely monitor for clinical worsening and emergence of suicidal thoughts and behaviors (5.1)**

INDICATIONS AND USAGE

ZOLOFT is a selective serotonin reuptake inhibitor (SSRI) indicated for the treatment of (1):

- Major depressive disorder (MDD)
- Obsessive-compulsive disorder (OCD)
- Panic disorder (PD)
- Posttraumatic stress disorder (PTSD)
- Social anxiety disorder (SAD)
- Premenstrual dysphoric disorder (PMDD)

DOSAGE AND ADMINISTRATION

Indication	Starting Dosage	Maximum Dosage
MDD (2.1)	50 mg per day	200 mg per day
OCD (2.1)	25 mg per day (ages 6-12)<br>50 mg per day (ages ≥ 13)	200 mg per day
PD, PTSD, SAD (2.1)	25 mg per day	200 mg per day
PMDD (2.2) continuous dosing	50 mg per day	150 mg per day
PMDD (2.2) intermittent dosing	50 mg per day during luteal phase only	100 mg per day during luteal phase only

- If inadequate response to starting dosage, titrate in 25-50 mg per day increments once weekly in MDD, OCD, PD, PTSD, and SAD (2.1)
- See Full Prescribing Information for titration in PMDD (2.2)
- Hepatic impairment:
  - Mild: Recommended starting and maximum dosage is half recommended dosage (2.4)
  - Moderate or severe: Not recommended (2.4)
- When discontinuing ZOLOFT, reduce dose gradually (2.6, 5.4)
- Oral solution: Must be diluted before administration (2.7)

DOSAGE FORMS AND STRENGTHS

- Tablets: 25 mg, 50 mg and 100 mg (3)
- Oral solution: 20 mg/mL (3)

CONTRAINDICATIONS

- Concomitant use of monoamine oxidase inhibitors (MAOIs), or use within 14 days of stopping MAOIs (4, 7.1)
- Concomitant use of pimozide (4, 7.1)
- Known hypersensitivity to sertraline or excipients (4, 5.4)
- ZOLOFT oral solution only: Concomitant use of disulfiram (4)

WARNINGS AND PRECAUTIONS

- Serotonin Syndrome: Increased risk when co-administered with other serotonergic agents (e.g., SSRI, SNRI, triptans), but also when taken alone. If it occurs, discontinue ZOLOFT and initiate supportive treatment. (5.2)
- Increased Risk of Bleeding: Concomitant use of aspirin, nonsteroidal anti-inflammatory drugs (NSAIDs), other antiplatelet drugs, warfarin, and other anticoagulants may increase this risk. (5.3)
- Activation of Mania/Hypomania: Screen patients for bipolar disorder. (5.4)
- Seizures: Use with caution in patients with seizure disorders. (5.6)
- Angle Closure Glaucoma: Avoid use of antidepressants, including ZOLOFT, in patients with untreated anatomically narrow angles. (5.7)
- QTc Prolongation: ZOLOFT should be used with caution in patients with risk factors for QTc prolongation. (5.10)
- Sexual Dysfunction: ZOLOFT may cause symptoms of sexual dysfunction. (5.11)

ADVERSE REACTIONS

Most common adverse reactions (≥5% and twice placebo) in pooled placebo-controlled MDD, OCD, PD, PTSD, SAD and PMDD clinical trials were nausea, diarrhea/loose stool, tremor, dyspepsia, decreased appetite, hyperhidrosis, ejaculation failure, and decreased libido. (6.1)

To report SUSPECTED ADVERSE REACTIONS, contact Viatris at 1-877-446-3679 (1-877-4-INFO-RX) or FDA at 1-800-FDA-1088 or www.fda.gov/medwatch.

DRUG INTERACTIONS

- Protein-bound drugs: Monitor for adverse reactions and reduce dosage of ZOLOFT or other protein-bound drugs (e.g., warfarin) as warranted. (7.1, 12.3)
- CYP2D6 substrates: Reduce dosage of drugs metabolized by CYP2D6. (7.1, 12.3)

USE IN SPECIFIC POPULATIONS

- Pregnancy: Third trimester use may increase risk for persistent pulmonary hypertension and withdrawal in the neonate. (8.1)
- Pediatric use: Safety and effectiveness of ZOLOFT in pediatric patients other than those with OCD have not been established. (8.4)

See 17 for PATIENT COUNSELING INFORMATION and Medication Guide.

Revised: 1/2023

FULL PRESCRIBING INFORMATION: CONTENTS*

WARNING: SUICIDAL THOUGHTS AND BEHAVIORS
1 INDICATIONS AND USAGE
2 DOSAGE AND ADMINISTRATION
2.1 Dosage in Patients with MDD, OCD, PD, PTSD, and SAD
2.2 Dosage in Patients with PMDD
2.3 Screen for Bipolar Disorder Prior to Starting ZOLOFT
2.4 Dosage Modifications in Patients with Hepatic Impairment
2.5 Switching Patients to or from a Monoamine Oxidase Inhibitor Antidepressant
2.6 Discontinuation of Treatment with ZOLOFT

2.7 Preparation of ZOLOFT Oral Solution
3 DOSAGE FORMS AND STRENGTHS
4 CONTRAINDICATIONS
5 WARNINGS AND PRECAUTIONS
5.1 Suicidal Thoughts and Behaviors in Pediatric and Young Adult Patients
5.2 Serotonin Syndrome
5.3 Increased Risk of Bleeding
5.4 Activation of Mania or Hypomania
5.5 Discontinuation Syndrome
5.6 Seizures
5.7 Angle-Closure Glaucoma
5.8 Hyponatremia
5.9 False-Positive Effects on Screening Tests for Benzodiazepines
5.10 QTc Prolongation
5.11 Sexual Dysfunction
6 ADVERSE REACTIONS
6.1 Clinical Trials Experience
6.2 Post-marketing Experience
7 DRUG INTERACTIONS
7.1 Clinically Significant Drug Interactions
7.2 Drugs Having No Clinically Important Interactions with ZOLOFT
7.3 False-Positive Screening Tests for Benzodiazepines
8 USE IN SPECIFIC POPULATIONS
8.1 Pregnancy
8.2 Lactation
8.4 Pediatric Use
8.5 Geriatric Use
8.6 Hepatic Impairment
8.7 Renal Impairment
9 DRUG ABUSE AND DEPENDENCE
9.1 Controlled Substance
9.2 Abuse
10 OVERDOSAGE
11 DESCRIPTION
12 CLINICAL PHARMACOLOGY
12.1 Mechanism of Action
12.2 Pharmacodynamics
12.3 Pharmacokinetics
13 NONCLINICAL TOXICOLOGY
13.1 Carcinogenesis, Mutagenesis, Impairment of Fertility
14 CLINICAL STUDIES
14.1 Major Depressive Disorder
14.2 Obsessive-Compulsive Disorder
14.3 Panic Disorder
14.4 Posttraumatic Stress Disorder
14.5 Social Anxiety Disorder
14.6 Premenstrual Dysphoric Disorder
16 HOW SUPPLIED/STORAGE AND HANDLING
17 PATIENT COUNSELING INFORMATION

* Sections or subsections omitted from the full prescribing information are not listed.

FULL PRESCRIBING INFORMATION

WARNING: SUICIDAL THOUGHTS AND BEHAVIORS

Antidepressants increased the risk of suicidal thoughts and behavior in pediatric and young adult patients in short-term studies. Closely monitor all antidepressant-treated patients for clinical worsening, and for emergence of suicidal thoughts and behaviors *[See Warnings and Precautions (5.1)]*.

1 INDICATIONS AND USAGE

ZOLOFT is indicated for the treatment of the following *[See Clinical Studies (14)]*:

- Major depressive disorder (MDD)
- Obsessive-compulsive disorder (OCD)
- Panic disorder (PD)
- Posttraumatic stress disorder (PTSD)
- Social anxiety disorder (SAD)
- Premenstrual dysphoric disorder (PMDD)

2 DOSAGE AND ADMINISTRATION

2.1 Dosage in Patients with MDD, OCD, PD, PTSD, and SAD

The recommended initial dosage and maximum ZOLOFT dosage in patients with MDD, OCD, PD, PTSD, and SAD are displayed in Table 1 below. A dosage of 25 mg or 50 mg per day is the initial therapeutic dosage.

For adults and pediatric patients, subsequent dosages may be increased in case of an inadequate response in 25 to 50 mg per day increments once a week, depending on tolerability, up to a maximum of 200 mg per day. Given the 24-hour elimination half-life of ZOLOFT, the recommended interval between dose changes is one week.

Table 1: Recommended Daily Dosage of ZOLOFT in Patients with MDD, OCD, PD, PTSD, and SAD

Indication	Starting Dose	Therapeutic Range
**Adults**		
MDD	50 mg	50-200 mg
OCD	50 mg	
PD, PTSD, SAD	25 mg	
**Pediatric Patients**		
OCD (ages 6-12 years old)	25 mg	50-200 mg

OCD (ages 13-17 years old)	50 mg	50-200 mg

2.2 Dosage in Patients with PMDD

The recommended starting ZOLOFT dosage in adult women with PMDD is 50 mg per day. ZOLOFT may be administered either continuously (every day throughout the menstrual cycle) or intermittently (only during the luteal phase of the menstrual cycle, i.e., starting the daily dosage 14 days prior to the anticipated onset of menstruation and continuing through the onset of menses). Intermittent dosing would be repeated with each new cycle.

- When dosing continuously, patients not responding to a 50 mg dosage may benefit from dosage increases at 50 mg increments per menstrual cycle up to 150 mg per day.
- When dosing intermittently, patients not responding to a 50 mg dosage may benefit from increasing the dosage up to a maximum of 100 mg per day during the next menstrual cycle (and subsequent cycles) as follows: 50 mg per day during the first 3 days of dosing followed by 100 mg per day during the remaining days in the dosing cycle.

2.3 Screen for Bipolar Disorder Prior to Starting ZOLOFT

Prior to initiating treatment with ZOLOFT or another antidepressant, screen patients for a personal or family history of bipolar disorder, mania, or hypomania *[See Warnings and Precautions (5.4)]*.

2.4 Dosage Modifications in Patients with Hepatic Impairment

Both the recommended starting dosage and therapeutic range in patients with mild hepatic impairment (Child Pugh scores 5 or 6) are half the recommended daily dosage *[See Dosage and Administration (2.1, 2.2)]*. The use of ZOLOFT in patients with moderate (Child Pugh scores 7 to 9) or severe hepatic impairment (Child Pugh scores 10-15) is not recommended *[See Use in Specific Populations (8.6), Clinical Pharmacology (12.3)]*.

2.5 Switching Patients to or from a Monoamine Oxidase Inhibitor Antidepressant

At least 14 days must elapse between discontinuation of a monoamine oxidase inhibitor (MAOI) antidepressant and initiation of ZOLOFT. In addition, at least 14 days must elapse after stopping ZOLOFT before starting an MAOI antidepressant *[See Contraindications (4), Warnings and Precautions (5.2)]*.

2.6 Discontinuation of Treatment with ZOLOFT

Adverse reactions may occur upon discontinuation of ZOLOFT *[See Warnings and Precautions (5.5)]*. Gradually reduce the dosage rather than stopping ZOLOFT abruptly whenever possible.

2.7 Preparation of ZOLOFT Oral Solution

ZOLOFT oral solution must be diluted before use.

- Use the supplied calibrated dropper to measure the amount of ZOLOFT oral solution needed
- Note: The supplied calibrated dropper has 25 mg and 50 mg graduation marks only
- Mix with 4 ounces (1/2 cup) of water, ginger ale, lemon/lime soda, lemonade or orange juice ONLY. After mixing, a slight haze may appear, which is normal.

Instruct patients or caregivers to immediately take the dose after mixing.

3 DOSAGE FORMS AND STRENGTHS

25 mg tablets: light green film-coated, engraved on one side with “ZOLOFT” and on the other side scored and engraved with “25 mg”

50 mg tablets: light blue film-coated, engraved on one side with “ZOLOFT” and on the other side scored and engraved with “50 mg”

100 mg tablets: light yellow film-coated, engraved on one side with “ZOLOFT” and on the other side scored and engraved with “100 mg”

Oral solution: a clear, colorless solution with a menthol scent containing sertraline hydrochloride equivalent to 20 mg of sertraline per mL and 12% alcohol. It is supplied as a 60 mL bottle with an accompanying calibrated dropper that has 25 mg and 50 mg graduation marks.

4 CONTRAINDICATIONS

ZOLOFT is contraindicated in patients:

- Taking, or within 14 days of stopping, MAOIs, (including the MAOIs linezolid and intravenous methylene blue) because of an increased risk of serotonin syndrome *[See Warnings and Precautions (5.2), Drug Interactions (7.1)]*.
- Taking pimozide *[See Drug Interactions (7.1)]*.
- With known hypersensitivity to sertraline (e.g., anaphylaxis, angioedema) *[See Adverse Reactions (6.1, 6.2)]*.

In addition to the contraindications for all ZOLOFT formulations listed above, ZOLOFT oral solution is contraindicated in patients:

- Taking disulfiram. ZOLOFT oral solution contains alcohol, and concomitant use of ZOLOFT and disulfiram may result in a disulfiram-alcohol reaction.

5 WARNINGS AND PRECAUTIONS

5.1 Suicidal Thoughts and Behaviors in Pediatric and Young Adult Patients

In pooled analyses of placebo-controlled trials of antidepressant drugs (SSRIs and other antidepressant classes) that included approximately 77,000 adult patients and over 4,400 pediatric patients, the incidence of suicidal thoughts and behaviors in pediatric and young adult patients was greater in antidepressant-treated patients than in placebo-

treated patients. The drug-placebo differences in the number of cases of suicidal thoughts and behaviors per 1000 patients treated are provided in Table 2.

No suicides occurred in any of the pediatric studies. There were suicides in the adult studies, but the number was not sufficient to reach any conclusion about antidepressant drug effect on suicide.

Table 2: Risk Differences of the Number of Cases of Suicidal Thoughts or Behaviors in the Pooled Placebo-Controlled Trials of Antidepressants in Pediatric and Adult Patients

**Age Range (years)**	**Drug-Placebo Difference in Number of Patients of Suicidal Thoughts or Behaviors per 1000 Patients Treated**
	**Increases Compared to Placebo**
<18	14 additional patients
18-24	5 additional patients
	**Decreases Compared to Placebo**
25-64	1 fewer patient
≥65	6 fewer patients

It is unknown whether the risk of suicidal thoughts and behaviors in pediatric and young adult patients extends to longer-term use, i.e., beyond four months. However, there is substantial evidence from placebo-controlled maintenance trials in adults with MDD that antidepressants delay the recurrence of depression.

Monitor all antidepressant-treated patients for clinical worsening and emergence of suicidal thoughts and behaviors, especially during the initial few months of drug therapy and at times of dosage changes. Counsel family members or caregivers of patients to monitor for changes in behavior and to alert the healthcare provider. Consider changing the therapeutic regimen, including possibly discontinuing ZOLOFT, in patients whose depression is persistently worse, or who are experiencing emergent suicidal thoughts or behaviors.

5.2 Serotonin Syndrome

Serotonin-norepinephrine reuptake inhibitors (SNRIs) and selective serotonin reuptake inhibitors (SSRIs), including ZOLOFT, can precipitate serotonin syndrome, a potentially life-threatening condition. The risk is increased with concomitant use of other serotonergic drugs (including triptans, tricyclic antidepressants, fentanyl, lithium, tramadol, tryptophan, buspirone, amphetamines, and St. John’s Wort) and with drugs that impair metabolism of serotonin, i.e., MAOIs *[See Contraindications (4), Drug Interactions (7.1)]*. Serotonin syndrome can also occur when these drugs are used alone.

Serotonin syndrome signs and symptoms may include mental status changes (e.g., agitation, hallucinations, delirium, and coma), autonomic instability (e.g., tachycardia, labile blood pressure, dizziness, diaphoresis, flushing, hyperthermia), neuromuscular symptoms (e.g., tremor, rigidity, myoclonus, hyperreflexia, incoordination), seizures, and gastrointestinal symptoms (e.g., nausea, vomiting, diarrhea).

The concomitant use of ZOLOFT with MAOIs is contraindicated. In addition, do not initiate ZOLOFT in a patient being treated with MAOIs such as linezolid or intravenous methylene blue. No reports involved the administration of methylene blue by other routes (such as oral tablets or local tissue injection). If it is necessary to initiate treatment with an MAOI such as linezolid or intravenous methylene blue in a patient taking ZOLOFT, discontinue ZOLOFT before initiating treatment with the MAOI *[See Contraindications (4), Drug Interactions (7.1)]*.

Monitor all patients taking ZOLOFT for the emergence of serotonin syndrome. Discontinue treatment with ZOLOFT and any concomitant serotonergic agents immediately if the above symptoms occur, and initiate supportive symptomatic treatment. If concomitant use of ZOLOFT with other serotonergic drugs is clinically warranted, inform patients of the increased risk for serotonin syndrome and monitor for symptoms.

5.3 Increased Risk of Bleeding

Drugs that interfere with serotonin reuptake inhibition, including ZOLOFT, increase the risk of bleeding events. Concomitant use of aspirin, nonsteroidal anti-inflammatory drugs (NSAIDs), other antiplatelet drugs, warfarin, and other anticoagulants may add to this risk. Case reports and epidemiological studies (case-control and cohort design) have demonstrated an association between use of drugs that interfere with serotonin reuptake and the occurrence of gastrointestinal bleeding. Bleeding events related to drugs that interfere with serotonin reuptake have ranged from ecchymosis, hematoma, epistaxis, and petechiae to life-threatening hemorrhages.

Inform patients of the increased risk of bleeding associated with the concomitant use of ZOLOFT and antiplatelet agents or anticoagulants. For patients taking warfarin, carefully monitor the international normalized ratio.

5.4 Activation of Mania or Hypomania

In patients with bipolar disorder, treating a depressive episode with ZOLOFT or another antidepressant may precipitate a mixed/manic episode. In controlled clinical trials, patients with bipolar disorder were generally excluded; however, symptoms of mania or hypomania were reported in 0.4% of patients treated with ZOLOFT. Prior to initiating treatment with ZOLOFT, screen patients for any personal or family history of bipolar disorder, mania, or hypomania.

5.5 Discontinuation Syndrome

Adverse reactions after discontinuation of serotonergic antidepressants, particularly after abrupt discontinuation, include: nausea, sweating, dysphoric mood, irritability, agitation, dizziness, sensory disturbances (e.g., paresthesia, such as electric shock sensations), tremor, anxiety, confusion, headache, lethargy, emotional lability, insomnia, hypomania, tinnitus, and seizures. A gradual reduction in dosage rather than abrupt cessation is recommended whenever possible *[See Dosage and Administration (2.6)]*.

5.6 Seizures

ZOLOFT has not been systematically evaluated in patients with seizure disorders. Patients with a history of seizures were excluded from clinical studies. ZOLOFT should be prescribed with caution in patients with a seizure disorder.

5.7 Angle-Closure Glaucoma

The pupillary dilation that occurs following use of many antidepressant drugs including ZOLOFT may trigger an angle closure attack in a patient with anatomically narrow angles who does not have a patent iridectomy. Avoid use of antidepressants, including ZOLOFT, in patients with untreated anatomically narrow angles.

5.8 Hyponatremia

Hyponatremia may occur as a result of treatment with SNRIs and SSRIs, including ZOLOFT. Cases with serum sodium lower than 110 mmol/L have been reported. Signs and symptoms of hyponatremia include headache, difficulty concentrating, memory impairment, confusion, weakness, and unsteadiness, which may lead to falls. Signs and symptoms associated with more severe or acute cases have included hallucination, syncope, seizure, coma, respiratory arrest, and death. In many cases, this hyponatremia appears to be the result of the syndrome of inappropriate antidiuretic hormone secretion (SIADH).

In patients with symptomatic hyponatremia, discontinue ZOLOFT and institute appropriate medical intervention. Elderly patients, patients taking diuretics, and those who are volume-depleted may be at greater risk of developing hyponatremia with SSRIs and SNRIs *[See Use in Specific Populations (8.5)]*.

5.9 False-Positive Effects on Screening Tests for Benzodiazepines

False-positive urine immunoassay screening tests for benzodiazepines have been reported in patients taking ZOLOFT. This finding is due to lack of specificity of the screening tests. False-positive test results may be expected for several days following discontinuation of ZOLOFT. Confirmatory tests, such as gas chromatography/mass spectrometry, will help distinguish ZOLOFT from benzodiazepines *[See Drug Interactions (7.3)]*.

5.10 QTc Prolongation

During post-marketing use of sertraline, cases of QTc prolongation and Torsade de Pointes (TdP) have been reported. Most reports were confounded by other risk factors. In a randomized, double-blind, placebo- and positive-controlled three-period crossover thorough QTc study in 54 healthy adult subjects, there was a positive relationship between the length of the rate-adjusted QTc interval and serum sertraline concentration. Therefore, ZOLOFT should be used with caution in patients with risk factors for QTc prolongation *[See Drug Interactions (7.1), Clinical Pharmacology (12.2)]*.

5.11 Sexual Dysfunction

Use of SSRIs, including ZOLOFT, may cause symptoms of sexual dysfunction *[see Adverse Reactions (6.1)]*. In male patients, SSRI use may result in ejaculatory delay or failure, decreased libido, and erectile dysfunction. In female patients, SSRI use may result in decreased libido and delayed or absent orgasm.

It is important for prescribers to inquire about sexual function prior to initiation of ZOLOFT and to inquire specifically about changes in sexual function during treatment, because sexual function may not be spontaneously reported. When evaluating changes in sexual function, obtaining a detailed history (including timing of symptom onset) is

important because sexual symptoms may have other causes, including the underlying psychiatric disorder. Discuss potential management strategies to support patients in making informed decisions about treatment.

6 ADVERSE REACTIONS

The following adverse reactions are described in more detail in other sections of the prescribing information:

- Hypersensitivity reactions to sertraline *[See Contraindications (4)]*
- Disulfiram-alcohol reaction when ZOLOFT oral solution is taken with disulfiram *[See Contraindications (4)]*
- QTc prolongation and ventricular arrhythmias when taken with pimozide *[See Contraindications (4), Clinical Pharmacology (12.2)]*
- Suicidal thoughts and behaviors *[See Warnings and Precautions (5.1)]*
- Serotonin syndrome *[See Contraindications (4), Warnings and Precautions (5.2), Drug Interactions (7.1)]*
- Increased risk of bleeding *[See Warnings and Precautions (5.3)]*
- Activation of mania/hypomania *[See Warnings and Precautions (5.4)]*
- Discontinuation syndrome *[See Warnings and Precautions (5.5)]*
- Seizures *[See Warnings and Precautions (5.6)]*
- Angle-closure glaucoma *[See Warnings and Precautions (5.7)]*
- Hyponatremia *[See Warnings and Precautions (5.8)]*
- Sexual Dysfunction *[See Warnings and Precautions (5.11)]*

6.1 Clinical Trials Experience

Because clinical trials are conducted under widely varying conditions, adverse reaction rates observed in the clinical trials of a drug cannot be directly compared to rates in the clinical trials of another drug and may not reflect the rates observed in practice.

The data described below are from randomized, double-blind, placebo-controlled trials of ZOLOFT (mostly 50 mg to 200 mg per day) in 3066 adults diagnosed with MDD, OCD, PD, PTSD, SAD, and PMDD. These 3066 patients exposed to ZOLOFT for 8 to12 weeks represent 568 patient-years of exposure. The mean age was 40 years; 57% were females and 43% were males.

The most common adverse reactions (≥5% and twice placebo) in all pooled placebo-controlled clinical trials of all ZOLOFT-treated patients with MDD, OCD, PD, PTSD, SAD and PMDD were nausea, diarrhea/loose stool, tremor, dyspepsia, decreased appetite, hyperhidrosis, ejaculation failure, and decreased libido (see Table 3). The following are the most common adverse reactions in trials of ZOLOFT (≥5% and twice placebo) by indication that were not mentioned previously.

- MDD: somnolence;
- OCD: insomnia, agitation;
- PD: constipation, agitation;
- PTSD: fatigue;
- PMDD: somnolence, dry mouth, dizziness, fatigue, and abdominal pain;
- SAD: insomnia, dizziness, fatigue, dry mouth, malaise.

Table 3: Common Adverse Reactions in Pooled Placebo-Controlled Trials in Adults with MDD, OCD, PD, PTSD, SAD, and PMDD*

	ZOLOFT (N=3066)	Placebo (N=2293)
**Cardiac disorders**		
Palpitations	4%	2%
**Eye disorders**		
Visual impairment	4%	2%
**Gastrointestinal disorders**		
Nausea	26%	12%
Diarrhea/Loose stools	20%	10%
Dry mouth	14%	9%
Dyspepsia	8%	4%
Constipation	6%	4%
Vomiting	4%	1%
**General disorders and administration site conditions**		
Fatigue	12%	8%
**Metabolism and nutrition disorders**		
Decreased appetite	7%	2%
**Nervous system disorders**		
Dizziness	12%	8%
Somnolence	11%	6%
Tremor	9%	2%
**Psychiatric Disorders**		
Insomnia	20%	13%
Agitation	8%	5%
Libido decreased	6%	2%
**Reproductive system and breast disorders**		
Ejaculation failure†	8%	1%
Erectile dysfunction†	4%	1%
Ejaculation disorder†	3%	0%
Male sexual dysfunction†	2%	0%
**Skin and subcutaneous tissue disorders**		
Hyperhidrosis	7%	3%

* Adverse reactions that occurred greater than 2% in ZOLOFT-treated patients and at least 2% greater in ZOLOFT-treated patients than placebo-treated patients.
† Denominator used was for male patients only (n=1316 ZOLOFT; n=973 placebo).

<u>Adverse Reactions Leading to Discontinuation in Placebo-Controlled Clinical Trials</u>

In all placebo-controlled studies in patients with MDD, OCD, PD, PTSD, SAD and PMDD, 368 (12%) of the 3066 patients who received ZOLOFT discontinued treatment due to an adverse reaction, compared with 93 (4%) of the 2293 placebo-treated patients. In placebo-controlled studies, the following were the common adverse reactions leading to

discontinuation in ZOLOFT-treated patients:

- MDD, OCD, PD, PTSD, SAD and PMDD: nausea (3%), diarrhea (2%), agitation (2%), and insomnia (2%).
- MDD (>2% and twice placebo): decreased appetite, dizziness, fatigue, headache, somnolence, tremor, and vomiting.
- OCD: somnolence.
- PD: nervousness and somnolence.

Male and Female Sexual Dysfunction

Although changes in sexual desire, sexual performance and sexual satisfaction often occur as manifestations of a psychiatric disorder, they may also be a consequence of SSRI treatment. However, reliable estimates of the incidence and severity of untoward experiences involving sexual desire, performance and satisfaction are difficult to obtain, in part because patients and healthcare providers may be reluctant to discuss them. Accordingly, estimates of the incidence of untoward sexual experience and performance cited in labeling may underestimate their actual incidence.

Table 4 below displays the incidence of sexual adverse reactions reported by at least 2% of ZOLOFT-treated patients and twice placebo from pooled placebo-controlled trials. For men and all indications, the most common adverse reactions (>2% and twice placebo) included: ejaculation failure, decreased libido, erectile dysfunction, ejaculation disorder, and male sexual dysfunction. For women, the most common adverse reaction (≥2% and twice placebo) was decreased libido.

Table 4: Most Common Sexual Adverse Reactions (≥2% and twice placebo) in Men or Women from ZOLOFT Pooled Controlled Trials in Adults with MDD, OCD, PD, PTSD, SAD, and PMDD

	**ZOLOFT**	**Placebo**
**Men only**	**(N=1316)**	**(N=973)**
Ejaculation failure	8%	1%
Libido decreased	7%	2%
Erectile dysfunction	4%	1%
Ejaculation disorder	3%	0%
Male sexual dysfunction	2%	0%
**Women only**	**(N=1750)**	**(N=1320)**
Libido decreased	4%	2%

Adverse Reactions in Pediatric Patients

In 281 pediatric patients treated with ZOLOFT in placebo-controlled studies, the overall profile of adverse reactions was generally similar to that seen in adult studies. Adverse reactions that do not appear in Table 3 (most common adverse reactions in adults) yet were reported in at least 2% of pediatric patients and at a rate of at least twice the placebo rate include fever, hyperkinesia, urinary incontinence, aggression, epistaxis, purpura, arthralgia, decreased weight, muscle twitching, and anxiety.

Other Adverse Reactions Observed During the Premarketing Evaluation of ZOLOFT

Other infrequent adverse reactions, not described elsewhere in the prescribing information, occurring at an incidence of < 2% in patients treated with ZOLOFT were:

Cardiac disorders - tachycardia

Ear and labyrinth disorders - tinnitus

Endocrine disorders - hypothyroidism

Eye disorders - mydriasis, blurred vision

Gastrointestinal disorders - hematochezia, melena, rectal hemorrhage

General disorders and administration site conditions - edema, gait disturbance, irritability, pyrexia

Hepatobiliary disorders - elevated liver enzymes

Immune system disorders - anaphylaxis

Metabolism and nutrition disorders - diabetes mellitus, hypercholesterolemia, hypoglycemia, increased appetite

Musculoskeletal and connective tissue disorders - arthralgia, muscle spasms, tightness, or twitching

Nervous system disorders - ataxia, coma, convulsion, decreased alertness, hypoesthesia, lethargy, psychomotor hyperactivity, syncope

Psychiatric disorders - aggression, bruxism, confusional state, euphoric mood, hallucination

Renal and urinary disorders - hematuria

Reproductive system and breast disorders - galactorrhea, priapism, vaginal hemorrhage

Respiratory, thoracic and mediastinal disorders - bronchospasm, epistaxis, yawning

Skin and subcutaneous tissue disorders - alopecia; cold sweat; dermatitis; dermatitis bullous; pruritus; purpura; erythematous, follicular, or maculopapular rash; urticaria

Vascular disorders - hemorrhage, hypertension, vasodilation

6.2 Post-marketing Experience

The following adverse reactions have been identified during postapproval use of ZOLOFT. Because these reactions are reported voluntarily from a population of uncertain size, it is not always possible to reliably estimate their frequency or establish a causal relationship to drug exposure.

Bleeding or clotting disorders - increased coagulation times (altered platelet function)

Cardiac disorders - AV block, bradycardia, atrial arrhythmias, QTc-interval prolongation, ventricular tachycardia (including Torsade de Pointes) *[See Clinical Pharmacology (12.2)]*

Endocrine disorders - gynecomastia, hyperprolactinemia, menstrual irregularities, SIADH

Eye disorders - blindness, optic neuritis, cataract

Hepatobiliary disorders - severe liver events (including hepatitis, jaundice, liver failure with some fatal outcomes), pancreatitis

Hemic and lymphatic disorders - agranulocytosis, aplastic anemia and pancytopenia, leukopenia, thrombocytopenia, lupus-like syndrome, serum sickness

Immune system disorders - angioedema

Metabolism and nutrition disorders - hyponatremia, hyperglycemia

Musculoskeletal and connective tissue disorders - rhabdomyolysis, trismus

Nervous system disorders - serotonin syndrome, extrapyramidal symptoms (including akathisia and dystonia), oculogyric crisis

Psychiatric disorders - psychosis, enuresis, paroniria

Renal and urinary disorders - acute renal failure

Respiratory, thoracic and mediastinal disorders - pulmonary hypertension, eosinophilic pneumonia

Skin and subcutaneous tissue disorders - photosensitivity skin reaction and other severe cutaneous reactions, which potentially can be fatal, such as Stevens-Johnson Syndrome (SJS) and toxic epidermal necrolysis (TEN)

Vascular disorders - cerebrovascular spasm (including reversible cerebral vasoconstriction syndrome and Call-Fleming syndrome), vasculitis

7 DRUG INTERACTIONS

7.1 Clinically Significant Drug Interactions

Table 5 includes clinically significant drug interactions with ZOLOFT *[See Clinical Pharmacology (12.3)]*.

Table 5. Clinically-Significant Drug Interactions with ZOLOFT

**Monoamine Oxidase Inhibitors (MAOIs)**	
Clinical Impact:	The concomitant use of SSRIs including ZOLOFT and MAOIs increases the risk of serotonin syndrome.
Intervention:	ZOLOFT is contraindicated in patients taking MAOIs, including MAOIs such as linezolid or intravenous methylene blue *[See Dosage and Administration (2.5), Contraindications (4), Warnings and Precautions (5.2)].*
Examples:	selegiline, tranylcypromine, isocarboxazid, phenelzine, linezolid, methylene blue
**Pimozide**	
Clinical Impact:	Increased plasma concentrations of pimozide, a drug with a narrow therapeutic index, may increase the risk of QTc prolongation and ventricular arrhythmias.
Intervention:	Concomitant use of pimozide and ZOLOFT is contraindicated *[See Contraindications (4)].*
**Other Serotonergic Drugs**	

Clinical Impact:	The concomitant use of serotonergic drugs with ZOLOFT increases the risk of serotonin syndrome.
Intervention:	Monitor patients for signs and symptoms of serotonin syndrome, particularly during treatment initiation and dosage increases. If serotonin syndrome occurs, consider discontinuation of ZOLOFT and/or concomitant serotonergic drugs *[See Warnings and Precautions (5.2)]*.
Examples:	other SSRIs, SNRIs, triptans, tricyclic antidepressants, fentanyl, lithium, tramadol, tryptophan, buspirone, St. John's Wort
**Drugs that Interfere with Hemostasis (antiplatelet agents and anticoagulants)**	
Clinical Impact:	The concurrent use of an antiplatelet agent or anticoagulant with ZOLOFT may potentiate the risk of bleeding.
Intervention:	Inform patients of the increased risk of bleeding associated with the concomitant use of ZOLOFT and antiplatelet agents and anticoagulants. For patients taking warfarin, carefully monitor the international normalized ratio *[See Warnings and Precautions (5.3)].*
Examples:	aspirin, clopidogrel, heparin, warfarin
**Drugs Highly Bound to Plasma Protein**	
Clinical Impact:	ZOLOFT is highly bound to plasma protein. The concomitant use of ZOLOFT with another drug that is highly bound to plasma protein may increase free concentrations of ZOLOFT or other tightly-bound drugs in plasma *[See Clinical Pharmacology (12.3)]*.
Intervention:	Monitor for adverse reactions and reduce dosage of ZOLOFT or other protein-bound drugs as warranted.
Examples:	warfarin
**Drugs Metabolized by CYP2D6**	
Clinical Impact:	ZOLOFT is a CYP2D6 inhibitor *[See Clinical Pharmacology (12.3)]*. The concomitant use of ZOLOFT with a CYP2D6 substrate may increase the exposure of the CYP2D6 substrate.
Intervention:	Decrease the dosage of a CYP2D6 substrate if needed with concomitant ZOLOFT use. Conversely, an increase in dosage of a CYP2D6 substrate may be needed if ZOLOFT is discontinued.
Examples:	propafenone, flecainide, atomoxetine, desipramine, dextromethorphan, metoprolol, nebivolol, perphenazine, thioridazine, tolterodine, venlafaxine
**Phenytoin**	
Clinical Impact:	Phenytoin is a narrow therapeutic index drug. ZOLOFT may increase phenytoin concentrations.
Intervention:	Monitor phenytoin levels when initiating or titrating ZOLOFT. Reduce phenytoin dosage if needed.
Examples:	phenytoin, fosphenytoin
**Drugs that Prolong the QTc Interval**	
Clinical Impact:	The risk of QTc prolongation and/or ventricular arrhythmias (e.g., TdP) is increased with concomitant use of other drugs which prolong the QTc interval *[See Warnings and Precautions (5.10), Clinical Pharmacology (12.2)]*.
Intervention:	Pimozide is contraindicated for use with sertraline. Avoid the

Intervention:	concomitant use of drugs known to prolong the QTc interval.
Examples:	Specific antipsychotics (e.g., ziprasidone, iloperidone, chlorpromazine, mesoridazine, droperidol); specific antibiotics (e.g., erythromycin, gatifloxacin, moxifloxacin, sparfloxacin); Class 1A antiarrhythmic medications (e.g., quinidine, procainamide); Class III antiarrhythmics (e.g., amiodarone, sotalol); and others (e.g., pentamidine, levomethadyl acetate, methadone, halofantrine, mefloquine, dolasetron mesylate, probucol or tacrolimus).

7.2 Drugs Having No Clinically Important Interactions with ZOLOFT

Based on pharmacokinetic studies, no dosage adjustment of ZOLOFT is necessary when used in combination with cimetidine. Additionally, no dosage adjustment is required for diazepam, lithium, atenolol, tolbutamide, digoxin, and drugs metabolized by CYP3A4, when ZOLOFT is administered concomitantly *[See Clinical Pharmacology (12.3)]*.

7.3 False-Positive Screening Tests for Benzodiazepines

False-positive urine immunoassay screening tests for benzodiazepines have been reported in patients taking ZOLOFT. This finding is due to lack of specificity of the screening tests. False-positive test results may be expected for several days following discontinuation of ZOLOFT. Confirmatory tests, such as gas chromatography/mass spectrometry, will distinguish sertraline from benzodiazepines.

8 USE IN SPECIFIC POPULATIONS

8.1 Pregnancy

<u>Risk Summary</u>

Overall, available published epidemiologic studies of pregnant women exposed to sertraline in the first trimester suggest no difference in major birth defect risk compared to the background rate for major birth defects in comparator populations. Some studies have reported increases for specific major birth defects; however, these study results are inconclusive *[See Data]*. There are clinical considerations regarding neonates exposed to SSRIs and SNRIs, including ZOLOFT, during the third trimester of pregnancy *[See Clinical Considerations]*.

Although no teratogenicity was observed in animal reproduction studies, delayed fetal ossification was observed when sertraline was administered during the period of organogenesis at doses less than the maximum recommended human dose (MRHD) in rats and doses 3.1 times the MRHD in rabbits on a mg/m^2 basis in adolescents. When sertraline was administered to female rats during the last third of gestation, there was an increase in the number of stillborn pups and pup deaths during the first four days after birth at the MRHD *[See Data]*.

The background risk of major birth defects and miscarriage for the indicated population are unknown. In the U.S. general population, the estimated background risk of major birth defects and miscarriage in clinically recognized pregnancies is 2-4% and 15-20%,

respectively. Advise a pregnant woman of possible risks to the fetus when prescribing ZOLOFT.

ZOLOFT oral solution contains 12% alcohol and is not recommended during pregnancy because there is no known safe level of alcohol exposure during pregnancy.

Clinical Considerations

Disease-associated maternal and/or embryo/fetal risk

A prospective longitudinal study followed 201 pregnant women with a history of major depression who were euthymic taking antidepressants at the beginning of pregnancy. The women who discontinued antidepressants during pregnancy were more likely to experience a relapse of major depression than women who continued antidepressants. Consider the risks of untreated depression when discontinuing or changing treatment with antidepressant medication during pregnancy and postpartum.

Fetal/Neonatal adverse reactions

Exposure to SSRIs and SNRIs, including ZOLOFT in late pregnancy may lead to an increased risk for neonatal complications requiring prolonged hospitalization, respiratory support, and tube feeding, and/or persistent pulmonary hypertension of the newborn (PPHN).

When treating a pregnant woman with ZOLOFT during the third trimester, carefully consider both the potential risks and benefits of treatment. Monitor neonates who were exposed to ZOLOFT in the third trimester of pregnancy for PPHN and drug discontinuation syndrome *[See Data]*.

Data

Human Data

Third Trimester Exposure

Neonates exposed to ZOLOFT and other SSRIs or SNRIs late in the third trimester have developed complications requiring prolonged hospitalization, respiratory support, and tube feeding. These findings are based on post-marketing reports. Such complications can arise immediately upon delivery. Reported clinical findings have included respiratory distress, cyanosis, apnea, seizures, temperature instability, feeding difficulty, vomiting, hypoglycemia, hypotonia, hypertonia, hyperreflexia, tremor, jitteriness, irritability, and constant crying. These features are consistent with either a direct toxic effect of SSRIs and SNRIs or, possibly, a drug discontinuation syndrome. In some cases, the clinical picture was consistent with serotonin syndrome *[See Warnings and Precautions (5.2)]*.

Exposure during late pregnancy to SSRIs may have an increased risk for persistent pulmonary hypertension of the newborn (PPHN). PPHN occurs in 1-2 per 1,000 live births in the general population and is associated with substantial neonatal morbidity and mortality. In a retrospective case-control study of 377 women whose infants were born with PPHN and 836 women whose infants were born healthy, the risk for developing PPHN was approximately six-fold higher for infants exposed to SSRIs after the 20th week of gestation compared to infants who had not been exposed to antidepressants during pregnancy. A study of 831,324 infants born in Sweden in 1997-2005 found a

PPHN risk ratio of 2.4 (95% CI 1.2-4.3) associated with patient-reported maternal use of SSRIs “in early pregnancy” and a PPHN risk ratio of 3.6 (95% CI 1.2-8.3) associated with a combination of patient-reported maternal use of SSRIs “in early pregnancy” and an antenatal SSRI prescription “in later pregnancy”.

First Trimester Exposure

The weight of evidence from epidemiologic studies of pregnant women exposed to sertraline in the first trimester suggest no difference in major birth defect risk compared to the background rate for major birth defects in pregnant women who were not exposed to sertraline. A meta-analysis of studies suggest no increase in the risk of total malformations (summary odds ratio=1.01, 95% CI=0.88-1.17) or cardiac malformations (summary odds ratio=0.93, 95% CI=0.70-1.23) among offspring of women with first trimester exposure to sertraline. An increased risk of congenital cardiac defects, specifically septal defects, the most common type of congenital heart defect, was observed in some published epidemiologic studies with first trimester sertraline exposure; however, most of these studies were limited by the use of comparison populations that did not allow for the control of confounders such as the underlying depression and associated conditions and behaviors, which may be factors associated with increased risk of these malformations.

Animal Data

Reproduction studies have been performed in rats and rabbits at doses up to 80 mg/kg/day and 40 mg/kg/day, respectively. These doses correspond to approximately 3.1 times the maximum recommended human dose (MRHD) of 200 mg/day on a mg/m^2 basis in adolescents. There was no evidence of teratogenicity at any dose level. When pregnant rats and rabbits were given sertraline during the period of organogenesis, delayed ossification was observed in fetuses at doses of 10 mg/kg (0.4 times the MRHD on a mg/m2 basis) in rats and 40 mg/kg (3.1 times the MRHD on a mg/m^2 basis) in rabbits. When female rats received sertraline during the last third of gestation and throughout lactation, there was an increase in stillborn pups and pup deaths during the first 4 days after birth. Pup body weights were also decreased during the first four days after birth. These effects occurred at a dose of 20 mg/kg (0.8 times the MRHD on a mg/m^2 basis). The no effect dose for rat pup mortality was 10 mg/kg (0.4 times the MRHD on a mg/m^2 basis). The decrease in pup survival was shown to be due to *in utero* exposure to sertraline. The clinical significance of these effects is unknown.

8.2 Lactation

Risk Summary

Available data from published literature demonstrate low levels of sertraline and its metabolites in human milk *[See Data]*. There are no data on the effects of sertraline on milk production. The developmental and health benefits of breastfeeding should be considered along with the mother’s clinical need for ZOLOFT and any potential adverse effects on the breastfed infant from the drug or from the underlying maternal condition.

Data

In a published pooled analysis of 53 mother-infant pairs, exclusively human milk-fed infants had an average of 2% (range 0% to 15%) of the sertraline serum levels

measured in their mothers. No adverse reactions were observed in these infants.

8.4 Pediatric Use

The safety and efficacy of ZOLOFT have been established in the treatment of OCD in pediatric patients aged 6 to 17 *[See Adverse Reactions (6.1), Clinical Pharmacology (12.3), Clinical Studies (14.2)]*. Safety and effectiveness in pediatric patients in patients with OCD below the age of 6 have not been established. Safety and effectiveness have not been established in pediatric patients for indications other than OCD. Two placebo-controlled trials were conducted in pediatric patients with MDD, but the data were not sufficient to support an indication for use in pediatric patients.

Monitoring Pediatric Patients Treated with ZOLOFT

Monitor all patients being treated with antidepressants for clinical worsening, suicidal thoughts, and unusual changes in behavior, especially during the initial few months of treatment, or at times of dose increases or decreases *[See Boxed Warning, Warnings and Precautions (5.1)]*. Decreased appetite and weight loss have been observed with the use of SSRIs. Monitor weight and growth in pediatric patients treated with an SSRI such as ZOLOFT.

Weight Loss in Studies in Pediatric Patients with MDD

In a pooled analysis of two 10-week, double-blind, placebo-controlled, flexible dose (50-200 mg) outpatient trials for MDD (n=373), there was a difference in weight change between ZOLOFT and placebo of roughly 1 kg, for both children (ages 6-11) and adolescents (ages 12-17), in both age groups representing a slight weight loss for the ZOLOFT group compared to a slight gain for the placebo group. For children, about 7% of the ZOLOFT-treated patients had a weight loss greater than 7% of body weight compared to 0% of the placebo-treated patients; for adolescents, about 2% of ZOLOFT-treated patients had a weight loss > 7% of body weight compared to about 1% of placebo-treated patients.

A subset of patients who completed the randomized controlled trials in patients with MDD (ZOLOFT n=99, placebo n=122) were continued into a 24-week, flexible-dose, open-label, extension study. Those subjects who completed 34 weeks of ZOLOFT treatment (10 weeks in a placebo-controlled trial + 24 weeks open-label, n=68) had weight gain that was similar to that expected using data from age-adjusted peers. However, there are no studies that directly evaluate the long-term effects of ZOLOFT on the growth, development, and maturation in pediatric patients.

Alcohol Content in ZOLOFT Oral Solution

ZOLOFT oral solution contains 12% alcohol.

Juvenile Animal Data

A study conducted in juvenile rats at clinically relevant doses showed delay in sexual maturation, but there was no effect on fertility in either males or females.

In this study in which juvenile rats were treated with oral doses of sertraline at 0, 10, 40 or 80 mg/kg/day from postnatal day 21 to 56, a delay in sexual maturation was observed in males treated with 80 mg/kg/day and females treated with doses ≥10

mg/kg/day. There was no effect on male and female reproductive endpoints or neurobehavioral development up to the highest dose tested (80 mg/kg/day), except a decrease in auditory startle response in females at 40 and 80 mg/kg/day at the end of treatment but not at the end of the drug-free period. The highest dose of 80 mg/kg/day produced plasma levels (AUC) of sertraline 5 times those seen in pediatric patients (6-17 years of age) receiving the maximum recommended dose of sertraline (200 mg/day).

8.5 Geriatric Use

Of the total number of patients in clinical studies of ZOLOFT in patients with MDD, OCD, PD, PTSD, SAD and PMDD, 797 (17%) were ≥ 65 years old, while 197 (4%) were ≥ 75 years old.

No overall differences in safety or effectiveness were observed between these subjects and younger subjects, and other reported clinical experience has not identified differences in responses between the elderly and younger patients. In general, dose selection for an elderly patient should be conservative, usually starting at the low end of the dosing range, reflecting the greater frequency of decreased hepatic, renal, or cardiac function, and of concomitant disease or other drug therapy.

In 354 geriatric subjects treated with ZOLOFT in MDD placebo-controlled trials, the overall profile of adverse reactions was generally similar to that shown in Table 3 *[See Adverse Reactions (6.1)]*, except for tinnitus, arthralgia with an incidence of at least 2% and at a rate greater than placebo in geriatric patients.

SNRIs and SSRIs, including ZOLOFT, have been associated with cases of clinically significant hyponatremia in elderly patients, who may be at greater risk for this adverse reaction *[See Warnings and Precautions (5.8)]*.

8.6 Hepatic Impairment

The recommended dosage in patients with mild hepatic impairment (Child-Pugh score 5 or 6) is half the recommended dosage due to increased exposure in this patient population. The use of ZOLOFT in patients with moderate (Child-Pugh score 7 to 10) or severe hepatic impairment (Child-Pugh score 10-15) is not recommended, because ZOLOFT is extensively metabolized, and the effects of ZOLOFT in patients with moderate and severe hepatic impairment have not been studied *[See Dosage and Administration (2.4), Clinical Pharmacology (12.3)]*.

8.7 Renal Impairment

No dose adjustment is needed in patients with mild to severe renal impairment. Sertraline exposure does not appear to be affected by renal impairment *[See Clinical Pharmacology (12.3)]*.

9 DRUG ABUSE AND DEPENDENCE

9.1 Controlled Substance

ZOLOFT contains sertraline, which is not a controlled substance.

9.2 Abuse

In a placebo-controlled, double-blind, randomized study of the comparative abuse liability of ZOLOFT, alprazolam, and d-amphetamine in humans, ZOLOFT did not produce the positive subjective effects indicative of abuse potential, such as euphoria or drug liking, that were observed with the other two drugs.

10 OVERDOSAGE

The following have been reported with sertraline tablet overdosage:

- Seizures, which may be delayed, and altered mental status including coma.
- Cardiovascular toxicity, which may be delayed, including QRS and QTc interval prolongation. Hypertension most commonly seen, but rarely can see hypotension alone or with co-ingestants including alcohol.
- Serotonin syndrome (patients with a multiple drug overdosage with other proserotonergic drugs may have a higher risk).

Gastrointestinal decontamination with activated charcoal should be considered in patients who present early after a sertraline overdose. Consider contacting a Poison Center (1-800-221-2222) or a medical toxicologist for additional overdosage management recommendations.

11 DESCRIPTION

ZOLOFT contains sertraline hydrochloride, an SSRI. Sertraline hydrochloride has a molecular weight of 342.7 and has the following chemical name: (1S-cis)-4-(3,4-dichlorophenyl)-1,2,3,4-tetrahydro-N-methyl-1-naphthalenamine hydrochloride. The empirical formula $C_{17}H_{17}NCl_2$•HCl is represented by the following structural formula:

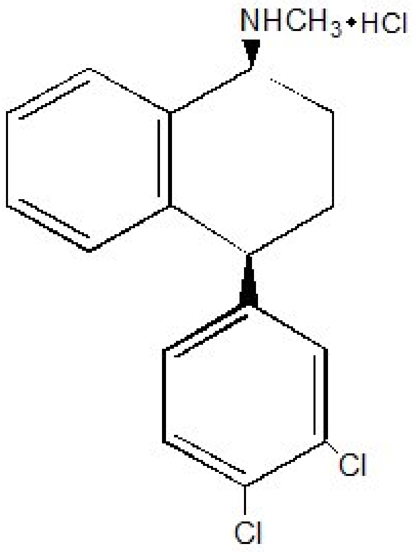

Sertraline hydrochloride is a white crystalline powder that is slightly soluble in water and isopropyl alcohol, and sparingly soluble in ethanol.

ZOLOFT tablets for oral administration contain 28.0 mg, 56.0 mg and 111.9 mg sertraline hydrochloride equivalent to 25, 50 and 100 mg of sertraline and the following inactive ingredients: dibasic calcium phosphate dihydrate, D & C Yellow #10 aluminum lake (in 25 mg tablet), FD & C Blue #1 aluminum lake (in 25 mg tablet), FD & C Red #40 aluminum lake (in 25 mg tablet), FD & C Blue #2 aluminum lake (in 50 mg tablet), hydroxypropyl cellulose, hypromellose, magnesium stearate, microcrystalline cellulose, polyethylene glycol, polysorbate 80, sodium starch glycolate, synthetic yellow iron oxide (in 100 mg tablet), and titanium dioxide.

ZOLOFT oral solution is available in a multidose 60 mL bottle. Each mL of solution contains 22.4 mg sertraline hydrochloride equivalent to 20 mg of sertraline. The solution contains the following inactive ingredients: glycerin, alcohol (12%), menthol, butylated hydroxytoluene (BHT). The oral solution must be diluted prior to administration *[See Dosage and Administration (2.7)]*. The dispenser contains dry natural rubber.

12 CLINICAL PHARMACOLOGY

12.1 Mechanism of Action

Sertraline potentiates serotonergic activity in the central nervous system through inhibition of neuronal reuptake of serotonin (5-HT).

12.2 Pharmacodynamics

Studies at clinically relevant doses have demonstrated that sertraline blocks the uptake of serotonin into human platelets. *In vitro* studies in animals also suggest that sertraline is a potent and selective inhibitor of neuronal serotonin reuptake and has only very weak effects on norepinephrine and dopamine neuronal reuptake. *In vitro* studies have shown that sertraline has no significant affinity for adrenergic (alpha1, alpha2, beta), cholinergic, GABA, dopaminergic, histaminergic, serotonergic (5HT1A, 5HT1B, 5HT2), or benzodiazepine receptors. The chronic administration of sertraline was found in animals to down regulate brain norepinephrine receptors. Sertraline does not inhibit monoamine oxidase.

Alcohol

In healthy subjects, the acute cognitive and psychomotor effects of alcohol were not potentiated by ZOLOFT.

Cardiac Electrophysiology

The effect of sertraline on the QTc interval was evaluated in a randomized, double-blind, placebo- and positive-controlled three-period crossover thorough QTc study in 54 healthy adult subjects. At 2-fold the maximum recommended daily dose (~3-fold the steady-state exposure for sertraline and N-desmethylsertraline), the largest mean ΔΔQTc was 10 ms with upper bound of two-sided 90% confidence interval of 12 ms. The length of the QTc interval was also positively correlated with serum concentrations of sertraline and N- desmethylsertraline concentrations. These concentration-based analyses, however, indicated a lesser effect on QTc at maximally observed concentration than in the primary analysis *[See Warnings and Precautions (5), Adverse Reactions (6), Drug Interactions (7), Overdosage (10)]*.

12.3 Pharmacokinetics

Absorption

Following oral once-daily ZOLOFT dosing over the range of 50 to 200 mg for 14 days, mean peak plasma concentrations (C_{max}) of sertraline occurred between 4.5 to 8.4 hours post-dosing. The average terminal elimination half-life of plasma sertraline is about 26 hours. Consistent with the terminal elimination half-life, there is an approximately two-fold accumulation up to steady-state concentrations, which are achieved after one week of once-daily dosing. Linear dose-proportional pharmacokinetics were demonstrated in a single dose study in which the C_{max} and area under the plasma concentration time curve (AUC) of sertraline were proportional to dose over a range of 50 to 200 mg. The single dose bioavailability of ZOLOFT tablets is approximately equal to an equivalent dose of ZOLOFT oral solution. Administration with food causes a small increase in C_{max} and AUC.

Metabolism

Sertraline undergoes extensive first pass metabolism. The principal initial pathway of metabolism for sertraline is N-demethylation. N-desmethylsertraline has a plasma terminal elimination half-life of 62 to 104 hours. Both *in vitro* biochemical and *in vivo* pharmacological testing have shown N-desmethylsertraline to be substantially less active than sertraline. Both sertraline and N-desmethylsertraline undergo oxidative deamination and subsequent reduction, hydroxylation, and glucuronide conjugation. In a study of radiolabeled sertraline involving two healthy male subjects, sertraline accounted for less than 5% of the plasma radioactivity. About 40-45% of the administered radioactivity was recovered in urine in 9 days. Unchanged sertraline was not detectable in the urine. For the same period, about 40-45% of the administered radioactivity was accounted for in feces, including 12-14% unchanged sertraline.

Desmethylsertraline exhibits time-related, dose dependent increases in AUC (0-24-hour), C_{max} and C_{min}, with about a 5- to 9-fold increase in these pharmacokinetic parameters between day 1 and day 14.

Protein Binding

In vitro protein binding studies performed with radiolabeled 3H-sertraline showed that sertraline is highly bound to serum proteins (98%) in the range of 20 to 500 ng/mL. However, at up to 300 and 200 ng/mL concentrations, respectively, sertraline and N-desmethylsertraline did not alter the plasma protein binding of two other highly protein bound drugs, warfarin and propranolol.

Studies in Specific Populations

Pediatric Patients

Sertraline pharmacokinetics were evaluated in a group of 61 pediatric patients (29 aged 6-12 years, 32 aged 13-17 years) including both males (N=28) and females (N=33). Relative to the adults, pediatric patients aged 6-12 years and 13-17 years showed about 22% lower AUC (0-24 hr) and C_{max} values when plasma concentration was adjusted for weight. The half-life was similar to that in adults, and no gender-associated differences were observed *[See Dosage and Administration (2.1), Use in Specific Populations (8.4)]*.

Geriatric Patients

Sertraline plasma clearance in a group of 16 (8 male, 8 female) elderly patients treated with 100 mg/day of ZOLOFT for 14 days was approximately 40% lower than in a similarly studied group of younger (25 to 32 year old) individuals. Steady-state, therefore, was achieved after 2 to 3 weeks in older patients. The same study showed a decreased clearance of desmethylsertraline in older males, but not in older females *[See Use in Specific Populations (8.5)]*.

Hepatic Impairment

In patients with chronic mild liver impairment (N=10: 8 patients with Child-Pugh scores of 5-6; and 2 patients with Child-Pugh scores of 7-8) who received 50 mg of ZOLOFT per day for 21 days, sertraline clearance was reduced, resulting in approximately 3-fold greater exposure compared to age-matched volunteers with normal hepatic function (N=10). The exposure to desmethylsertraline was approximately 2-fold greater in patients with mild hepatic impairment compared to age-matched volunteers with normal hepatic function. There were no significant differences in plasma protein binding observed between the two groups. The effects of ZOLOFT in patients with moderate and severe hepatic impairment have not been studied *[See Dosage and Administration (2.4), Use in Specific Populations (8.6)]*.

Renal Impairment

Sertraline is extensively metabolized and excretion of unchanged drug in urine is a minor route of elimination. In volunteers with mild to moderate (CLcr=30-60 mL/min), moderate to severe (CLcr=10-29 mL/min) or severe (receiving hemodialysis) renal impairment (N=10 each group), the pharmacokinetics and protein binding of 200 mg sertraline per day maintained for 21 days were not altered compared to age-matched volunteers (N=12) with no renal impairment. Thus sertraline multiple dose pharmacokinetics appear to be unaffected by renal impairment *[See Use in Specific Populations (8.7)]*.

<u>Drug Interaction Studies</u>

Pimozide

In a controlled study of a single dose (2 mg) of pimozide, 200 mg ZOLOFT (once daily) co-administration to steady state was associated with a mean increase in pimozide AUC and C_{max} of about 40%, but was not associated with any changes in ECG. The highest recommended pimozide dose (10 mg) has not been evaluated in combination with ZOLOFT. The effect on QTc interval and PK parameters at doses higher than 2 mg of pimozide are not known *[See Drug Interactions (7.1)]*.

Drugs Metabolized by CYP2D6

Many antidepressant drugs (e.g., SSRIs, including ZOLOFT, and most tricyclic antidepressant drugs) inhibit the biochemical activity of the drug metabolizing isozyme CYP2D6 (debrisoquin hydroxylase), and, thus, may increase the plasma concentrations of co-administered drugs that are metabolized by CYP2D6. The drugs for which this potential interaction is of greatest concern are those metabolized primarily by CYP2D6 and that have a narrow therapeutic index (e.g., tricyclic antidepressant drugs and the

Type 1C antiarrhythmics propafenone and flecainide). The extent to which this interaction is an important clinical problem depends on the extent of the inhibition of CYP2D6 by the antidepressant and the therapeutic index of the co-administered drug. There is variability among the drugs effective in the treatment of MDD in the extent of clinically important 2D6 inhibition, and in fact ZOLOFT at lower doses has a less prominent inhibitory effect on 2D6 than some others in the class. Nevertheless, even ZOLOFT has the potential for clinically important 2D6 inhibition *[See Drug Interactions (7.1)]*.

Phenytoin

Clinical trial data suggested that ZOLOFT may increase phenytoin concentrations *[See Drug Interactions (7.1)]*.

Cimetidine

In a study assessing disposition of ZOLOFT (100 mg) on the second of 8 days of cimetidine administration (800 mg daily), there were increases in ZOLOFT mean AUC (50%), Cmax (24%) and half-life (26%) compared to the placebo group *[See Drug Interactions (7.2)]*.

Diazepam

In a study comparing the disposition of intravenously administered diazepam before and after 21 days of dosing with either ZOLOFT (50 to 200 mg/day escalating dose) or placebo, there was a 32% decrease relative to baseline in diazepam clearance for the ZOLOFT group compared to a 19% decrease relative to baseline for the placebo group ($p<0.03$). There was a 23% increase in Tmax for desmethyldiazepam in the ZOLOFT group compared to a 20% decrease in the placebo group ($p<0.03$) *[See Drug Interactions (7.2)]*.

Lithium

In a placebo-controlled trial in normal volunteers, the administration of two doses of ZOLOFT did not significantly alter steady-state lithium levels or the renal clearance of lithium *[See Drug Interactions (7.2)]*.

Tolbutamide

In a placebo-controlled trial in normal volunteers, administration of ZOLOFT for 22 days (including 200 mg/day for the final 13 days) caused a statistically significant 16% decrease from baseline in the clearance of tolbutamide following an intravenous 1000 mg dose. ZOLOFT administration did not noticeably change either the plasma protein binding or the apparent volume of distribution of tolbutamide, suggesting that the decreased clearance was due to a change in the metabolism of the drug *[See Drug Interactions (7.2)]*.

Atenolol

ZOLOFT (100 mg) when administered to 10 healthy male subjects had no effect on the beta-adrenergic blocking ability of atenolol *[See Drug Interactions (7.2)]*.

Digoxin

In a placebo-controlled trial in normal volunteers, administration of ZOLOFT for 17 days (including 200 mg/day for the last 10 days) did not change serum digoxin levels or digoxin renal clearance *[See Drug Interactions (7.2)]*.

Drugs Metabolized by CYP3A4

In three separate in vivo interaction studies, ZOLOFT was co-administered with CYP3A4 substrates, terfenadine, carbamazepine, or cisapride under steady-state conditions. The results of these studies indicated that ZOLOFT did not increase plasma concentrations of terfenadine, carbamazepine, or cisapride. These data indicate that ZOLOFT's extent of inhibition of CYP3A4 activity is not likely to be of clinical significance. Results of the interaction study with cisapride indicate that ZOLOFT 200 mg (once daily) induces the metabolism of cisapride (cisapride AUC and C_{max} were reduced by about 35%) *[See Drug Interactions (7.2)]*.

Microsomal Enzyme Induction

Preclinical studies have shown ZOLOFT to induce hepatic microsomal enzymes. In clinical studies, ZOLOFT was shown to induce hepatic enzymes minimally as determined by a small (5%) but statistically significant decrease in antipyrine half-life following administration of 200 mg of ZOLOFT per day for 21 days. This small change in antipyrine half-life reflects a clinically insignificant change in hepatic metabolism.

13 NONCLINICAL TOXICOLOGY

13.1 Carcinogenesis, Mutagenesis, Impairment of Fertility

<u>Carcinogenesis</u>

Lifetime carcinogenicity studies were carried out in CD-1 mice and Long-Evans rats at doses up to 40 mg/kg/day. These doses correspond to 1 times (mice) and 2 times (rats) the maximum recommended human dose (MRHD) of 200 mg/day on a mg/m^2 basis. There was a dose-related increase of liver adenomas in male mice receiving sertraline at 10-40 mg/kg (0.25-1.0 times the MRHD on a mg/m^2 basis). No increase was seen in female mice or in rats of either sex receiving the same treatments, nor was there an increase in hepatocellular carcinomas. Liver adenomas have a variable rate of spontaneous occurrence in the CD-1 mouse and are of unknown significance to humans. There was an increase in follicular adenomas of the thyroid in female rats receiving sertraline at 40 mg/kg (2 times the MRHD on a mg/m^2 basis); this was not accompanied by thyroid hyperplasia. While there was an increase in uterine adenocarcinomas in rats receiving sertraline at 10-40 mg/kg (0.5-2.0 times the MRHD on a mg/m2 basis) compared to placebo controls, this effect was not clearly drug related.

<u>Mutagenesis</u>

Sertraline had no genotoxic effects, with or without metabolic activation, based on the following assays: bacterial mutation assay; mouse lymphoma mutation assay; and tests for cytogenetic aberrations *in vivo* in mouse bone marrow and *in vitro* in human lymphocytes.

<u>Impairment of Fertility</u>

A decrease in fertility was seen in one of two rat studies at a dose of 80 mg/kg (3.1 times the maximum recommended human dose on a mg/m^2 basis in adolescents).

14 CLINICAL STUDIES

Efficacy of ZOLOFT was established in the following trials:

- MDD: two short-term trials and one maintenance trials in adults *[See Clinical Studies (14.1)]*.
- OCD: three short-term trials in adults and one short-term trial in pediatric patients *[See Clinical Studies (14.2)]*.
- PD: three short-term trials and one maintenance trial in adults *[See Clinical Studies (14.3)]*.
- PTSD: two short-term trials and one maintenance trial in adults *[See Clinical Studies (14.4)]*.
- SAD: two short-term trials and one maintenance trial in adults *[See Clinical Studies (14.5)]*.
- PMDD: two short-term trials in adult female patients *[See Clinical Studies (14.6)]*.

14.1 Major Depressive Disorder

The efficacy of ZOLOFT as a treatment for MDD was established in two randomized, double-blind, placebo-controlled studies and one double-blind, randomized-withdrawal study following an open label study in adult (ages 18 to 65) outpatients who met the Diagnostic and Statistical Manual of Mental Disorders (DSM-III) criteria for MDD (studies MDD-1 and MDD-2).

- Study MDD-1 was an 8-week, 3-arm study with flexible dosing of ZOLOFT, amitriptyline, and placebo. Adult patients received ZOLOFT (N=126, in a daily dose titrated weekly to 50 mg, 100 mg, or 200 mg), amitriptyline (N=123, in a daily dose titrated weekly to 50 mg, 100 mg, or 150 mg), or placebo (N= 130).
- Study MDD-2 was a 6-week, multicenter parallel study of three fixed doses of ZOLOFT administered once daily at 50 mg (N=82), 100 mg (N=75), and 200 mg (N=56) doses and placebo (N=76) in the treatment of adult outpatients with MDD.

Overall, these studies demonstrated ZOLOFT to be superior to placebo on the Hamilton Rating Scale for Depression (HAMD-17) and the Clinical Global Impression Severity (CGI-S) of Illness and Global Improvement (CGI-I) scores. Study MDD-2 was not readily interpretable regarding a dose response relationship for effectiveness.

A third study (Study MDD-3) involved adult outpatients meeting the DSM-III criteria for MDD who had responded by the end of an initial 8-week open treatment phase on ZOLOFT 50-200 mg/day. These patients (n=295) were randomized to continuation on double-blind ZOLOFT 50-200 mg/day or placebo for 44 weeks. A statistically significantly lower relapse rate was observed for patients taking ZOLOFT compared to those on placebo: ZOLOFT [n=11 (8%)] and placebo [n=31 (39%)]. The mean ZOLOFT dose for completers was 70 mg/day.

Analyses for gender effects on outcome did not suggest any differential responsiveness on the basis of sex.

14.2 Obsessive-Compulsive Disorder

Adults with OCD

The effectiveness of ZOLOFT in the treatment of OCD was demonstrated in three multicenter placebo-controlled studies of adult (age 18-65) non-depressed outpatients (Studies OCD-1, OCD-2, and OCD-3). Patients in all three studies had moderate to severe OCD (DSM-III or DSM-III-R) with mean baseline ratings on the Yale-Brown Obsessive-Compulsive Scale (Y-BOCS) total score ranging from 23 to 25.

- Study OCD-1 was an 8-week randomized, placebo-controlled study with flexible dosing of ZOLOFT in a range of 50 to 200 mg/day, titrated in 50 mg increments every 4 days to a maximally tolerated dose; the mean dose for completers was 186 mg/day. Patients receiving ZOLOFT (N=43) experienced a mean reduction of approximately 4 points on the Y-BOCS total score which was statistically significantly greater than the mean reduction of 2 points in placebo-treated patients (N=44). The mean change in Y-BOCS from baseline to last visit (the primary efficacy endpoint) was -3.79 (ZOLOFT) and -1.48 (placebo).
- Study OCD-2 was a 12-week randomized, placebo-controlled fixed-dose study, including ZOLOFT doses of 50, 100, and 200 mg/day. ZOLOFT (N=240) was titrated to the assigned dose over two weeks in 50 mg increments every 4 days. Patients receiving ZOLOFT doses of 50 and 200 mg/day experienced mean reductions of approximately 6 points on the Y-BOCS total score, which were statistically significantly greater than the approximately 3 point reduction in placebo-treated patients (N=84). The mean change in Y-BOCS from baseline to last visit (the primary efficacy endpoint) was -5.7 (pooled results from ZOLOFT 50 mg, 100 mg, and 150 mg) and -2.85 (placebo).
- Study OCD-3 was a 12-week randomized, placebo controlled study with flexible dosing of ZOLOFT in a range of 50 to 200 mg/day; the mean dose for completers was 185 mg/day. ZOLOFT (N=241) was titrated to the assigned dose over two weeks in 50 mg increments every 4 days. Patients receiving ZOLOFT experienced a mean reduction of approximately 7 points on the Y-BOCS total score which was statistically significantly greater than the mean reduction of approximately 4 points in placebo-treated patients (N=84). The mean change in Y-BOCS from baseline to last visit (the primary efficacy endpoint) was - 6.5 (ZOLOFT) and -3.6 (placebo).

Analyses for age and gender effects on outcome did not suggest any differential responsiveness on the basis of age or sex.

The effectiveness of ZOLOFT was studied in the risk reduction of OCD relapse. In Study OCD-4, patients ranging in age from 18–79 meeting DSM-III-R criteria for OCD who had responded during a 52-week single-blind trial on ZOLOFT 50-200 mg/day (n=224) were randomized to continuation of ZOLOFT or to substitution of placebo for up to 28 weeks of observation for analysis of discontinuation due to relapse or insufficient clinical response. Response during the single-blind phase was defined as a decrease in the Y-BOCS score of ≥ 25% compared to baseline and a CGI-I of 1 (very much improved), 2 (much improved) or 3 (minimally improved). Insufficient clinical response during the double-blind phase indicated a worsening of the patient’s condition that resulted in study discontinuation, as assessed by the investigator. Relapse during the double-blind phase was defined as the following conditions being met (on three consecutive visits for 1 and 2, and condition 3 being met at visit 3):

- Condition 1: Y-BOCS score increased by ≥ 5 points, to a minimum of 20, relative to baseline;
- Condition 2: CGI-I increased by ≥ one point; and
- Condition 3: Worsening of the patient's condition in the investigator's judgment, to justify alternative treatment.

Patients receiving continued ZOLOFT treatment experienced a statistically significantly lower rate of discontinuation due to relapse or insufficient clinical response over the subsequent 28 weeks compared to those receiving placebo. This pattern was demonstrated in male and female subjects.

Pediatric Patients with OCD

The effectiveness of ZOLOFT for the treatment of OCD was demonstrated in a 12-week, multicenter, placebo-controlled, parallel group study in a pediatric outpatient population (ages 6-17) (Study OCD-5). ZOLOFT (N=92) was initiated at doses of either 25 mg/day (pediatric patients ages 6-12) or 50 mg/day (adolescents, ages 13-17), and then titrated at 3 and 4 day intervals (25 mg incremental dose for pediatric patients ages 6-12) or 1 week intervals (50 mg incremental dose adolescents ages 13-17) over the next four weeks to a maximum dose of 200 mg/day, as tolerated. The mean dose for completers was 178 mg/day. Dosing was once a day in the morning or evening. Patients in this study had moderate to severe OCD (DSM-III-R) with mean baseline ratings on the Children's Yale-Brown Obsessive-Compulsive Scale (CY-BOCS) total score of 22. Patients receiving ZOLOFT experienced a mean reduction of approximately 7 units on the CY-BOCS total score which was statistically significantly greater than the 3 unit reduction for placebo patients (n=95). Analyses for age and gender effects on outcome did not suggest any differential responsiveness on the basis of age or sex.

14.3 Panic Disorder

The effectiveness of ZOLOFT in the treatment of PD was demonstrated in three double-blind, placebo-controlled studies (Studies PD-1, PD-2, and PD-3) of adult outpatients who had a primary diagnosis of PD (DSM-III-R), with or without agoraphobia.

- Studies PD-1 and PD-2 were 10-week flexible dose studies of ZOLOFT (N=80 study PD-1 and N=88 study PD-2) compared to placebo (N=176 study PD-1 and PD-2). In both studies, ZOLOFT was initiated at 25 mg/day for the first week, then titrated in weekly increments of 50 mg per day to a maximum dose of 200 mg/day on the basis of clinical response and toleration. The mean ZOLOFT doses for completers to 10 weeks were 131 mg/day and 144 mg/day, respectively, for Studies PD-1 and PD-2. In these studies, ZOLOFT was shown to be statistically significantly more effective than placebo on change from baseline in panic attack frequency and on the Clinical Global Impression Severity (CGI-S) of Illness and Global Improvement (CGI-I) scores. The difference between ZOLOFT and placebo in reduction from baseline in the number of full panic attacks was approximately 2 panic attacks per week in both studies.
- Study PD-3 was a 12-week randomized, double-blind fixed-dose study, including ZOLOFT doses of 50, 100, and 200 mg/day. Patients receiving ZOLOFT (50 mg N=43, 100 mg N=44, 200 mg N=45) experienced a statistically significantly greater reduction in panic attack frequency than patients receiving placebo (N=45). Study PD-3 was not readily interpretable regarding a dose response relationship for

effectiveness.

Subgroup analyses did not indicate that there were any differences in treatment outcomes as a function of age, race, or gender.

In Study PD-4, patients meeting DSM-III-R criteria for PD who had responded during a 52-week open trial on ZOLOFT 50-200 mg/day (n=183) were randomized to continuation of ZOLOFT or to substitution of placebo for up to 28 weeks of observation for discontinuation due to relapse or insufficient clinical response. Response during the open phase was defined as a CGI-I score of 1(very much improved) or 2 (much improved). Insufficient clinical response in the double-blind phase indicated a worsening of the patient's condition that resulted in study discontinuation, as assessed by the investigator. Relapse during the double-blind phase was defined as the following conditions being met on three consecutive visits:

(1) CGI-I ≥ 3;
(2) meets DSM-III-R criteria for PD;
(3) number of panic attacks greater than at baseline.

Patients receiving continued ZOLOFT treatment experienced a statistically significantly lower rate of discontinuation due to relapse or insufficient clinical response over the subsequent 28 weeks compared to those receiving placebo. This pattern was demonstrated in male and female subjects.

14.4 Posttraumatic Stress Disorder

The effectiveness of ZOLOFT in the treatment of PTSD was established in two multicenter placebo-controlled studies (Studies PSTD-1 and PSTD-2) of adult outpatients who met DSM-III-R criteria for PTSD. The mean duration of PTSD for these patients was 12 years (Studies PSTD-1 and PSTD-2 combined) and 44% of patients (169 of the 385 patients treated) had secondary depressive disorder.

Studies PSTD-1 and PSTD-2 were 12-week flexible dose studies. ZOLOFT was initiated at 25 mg/day for the first week, and titrated in weekly increments of 50 mg per day to a maximum dose of 200 mg/day on the basis of clinical response and tolerability. The mean ZOLOFT dose for completers was 146 mg/day and 151 mg/day, respectively, for Studies PSTD-1 and PSTD-2. Study outcome was assessed by the Clinician-Administered PTSD Scale Part 2 (CAPS), which is a multi-item instrument that measures the three PTSD diagnostic symptom clusters of reexperiencing/intrusion, avoidance/numbing, and hyperarousal as well as the patient-rated Impact of Event Scale (IES), which measures intrusion and avoidance symptoms. Patients receiving ZOLOFT (N=99 and N=94, respectively) showed statistically significant improvement compared to placebo (N=83 and N=92) on change from baseline to endpoint on the CAPS, IES, and on the Clinical Global Impressions (CGI-S) Severity of Illness and Global Improvement (CGI-I) scores.

In two additional placebo-controlled PTSD trials (Studies PSTD-3 and PSTD-4), the difference in response to treatment between patients receiving ZOLOFT and patients receiving placebo was not statistically significant. One of these additional studies was conducted in patients similar to those recruited for Studies PSTD-1 and PSTD-2, while the second additional study was conducted in predominantly male veterans.

As PTSD is a more common disorder in women than men, the majority (76%) of patients in Studies PSTD-1 and PSTD-2 described above were women. Post hoc exploratory

analyses revealed a statistically significant difference between ZOLOFT and placebo on the CAPS, IES and CGI in women, regardless of baseline diagnosis of comorbid major depressive disorder, but essentially no effect in the relatively smaller number of men in these studies. The clinical significance of this apparent gender effect is unknown at this time. There was insufficient information to determine the effect of race or age on outcome.

In Study PSTD-5, patients meeting DSM-III-R criteria for PTSD who had responded during a 24-week open trial on ZOLOFT 50-200 mg/day (n=96) were randomized to continuation of ZOLOFT or to substitution of placebo for up to 28 weeks of observation for relapse. Response during the open phase was defined as a CGI-I of 1 (very much improved) or 2 (much improved), and a decrease in the CAPS-2 score of > 30% compared to baseline. Relapse during the double-blind phase was defined as the following conditions being met on two consecutive visits:

(1) CGI-I ≥ 3;
(2) CAPS-2 score increased by ≥ 30% and by ≥ 15 points relative to baseline; and
(3) worsening of the patient's condition in the investigator's judgment.

Patients receiving continued ZOLOFT treatment experienced statistically significantly lower relapse rates over the subsequent 28 weeks compared to those receiving placebo. This pattern was demonstrated in male and female subjects.

14.5 Social Anxiety Disorder

The effectiveness of ZOLOFT in the treatment of SAD (also known as social phobia) was established in two multicenter, randomized, placebo-controlled studies (Study SAD-1 and SAD-2) of adult outpatients who met DSM-IV criteria for SAD.

Study SAD-1 was a 12-week, flexible dose study comparing ZOLOFT (50-200 mg/day), n=211, to placebo, n=204, in which ZOLOFT was initiated at 25 mg/day for the first week, then titrated to the maximum tolerated dose in 50 mg increments biweekly. Study outcomes were assessed by the:

(1) Liebowitz Social Anxiety Scale (LSAS), a 24-item clinician administered instrument that measures fear, anxiety, and avoidance of social and performance situations, and
(2) Proportion of responders as defined by the Clinical Global Impression of Improvement (CGI-I) criterion of CGI-I ≤ 2 (very much or much improved).

ZOLOFT was statistically significantly more effective than placebo as measured by the LSAS and the percentage of responders.

Study SAD-2 was a 20-week, flexible dose study that compared ZOLOFT (50-200 mg/day), n=135, to placebo, n=69. ZOLOFT was titrated to the maximum tolerated dose in 50 mg increments every 3 weeks. Study outcome was assessed by the:

(1) Duke Brief Social Phobia Scale (BSPS), a multi-item clinician-rated instrument that measures fear, avoidance and physiologic response to social or performance situations,
(2) Marks Fear Questionnaire Social Phobia Subscale (FQ-SPS), a 5-item patient-rated instrument that measures change in the severity of phobic avoidance and distress, and

(3) CGI-I responder criterion of ≤ 2.

ZOLOFT was shown to be statistically significantly more effective than placebo as measured by the BSPS total score and fear, avoidance and physiologic factor scores, as well as the FQ-SPS total score, and to have statistically significantly more responders than placebo as defined by the CGI-I. Subgroup analyses did not suggest differences in treatment outcome on the basis of gender. There was insufficient information to determine the effect of race or age on outcome.

In Study SAD-3, patients meeting DSM-IV criteria for SAD who had responded while assigned to ZOLOFT (CGI-I of 1 or 2) during a 20-week placebo-controlled trial on ZOLOFT 50-200 mg/day were randomized to continuation of ZOLOFT or to substitution of placebo for up to 24 weeks of observation for relapse. Relapse was defined as ≥ 2 point increase in the Clinical Global Impression Severity of Illness (CGI-S) score compared to baseline or study discontinuation due to lack of efficacy. Patients receiving ZOLOFT continuation treatment experienced a statistically significantly lower relapse rate during this 24-week period than patients randomized to placebo substitution.

14.6 Premenstrual Dysphoric Disorder

The effectiveness of ZOLOFT for the treatment of PMDD was established in two double-blind, parallel group, placebo-controlled flexible dose trials (Studies PMDD-1 and PMDD-2) conducted over 3 menstrual cycles in adult female patients. The effectiveness of ZOLOFT for PMDD for more than 3 menstrual cycles has not been systematically evaluated in controlled trials.

Patients in Study PMDD-1 met DSM-III-R criteria for Late Luteal Phase Dysphoric Disorder (LLPDD), the clinical entity referred to as PMDD in DSM-IV. Patients in Study PMDD-2 met DSM-IV criteria for PMDD. Study PMDD-1 utilized continuous daily dosing throughout the study, while Study PMDD-2 utilized luteal phase dosing (intermittent dosing) for the 2 weeks prior to the onset of menses. The mean duration of PMDD symptoms was approximately 10.5 years in both studies. Patients taking oral contraceptives were excluded from these trials; therefore, the efficacy of ZOLOFT in combination with oral contraceptives for the treatment of PMDD is unknown.

Efficacy was assessed with the Daily Record of Severity of Problems (DRSP), a patient-rated instrument that mirrors the diagnostic criteria for PMDD as identified in the DSM-IV, and includes assessments for mood, physical symptoms, and other symptoms. Other efficacy assessments included the Hamilton Rating Scale for Depression (HAMD-17), and the Clinical Global Impression Severity of Illness (CGI-S) and Improvement (CGI-I) scores.

- In Study PMDD-1, involving 251 randomized patients, (n=125 on ZOLOFT and n=126 on placebo), ZOLOFT treatment was initiated at 50 mg/day and administered daily throughout the menstrual cycle. In subsequent cycles, ZOLOFT was titrated in 50 mg increments at the beginning of each menstrual cycle up to a maximum of 150 mg/day on the basis of clinical response and tolerability. The mean dose for completers was 102 mg/day. ZOLOFT administered daily throughout the menstrual cycle was statistically significantly more effective than placebo on change from baseline to endpoint on the DRSP total score, the HAMD-17 total score, and the CGI-S score, as well as the CGI-I score at endpoint.
- In Study PMDD-2, involving 281 randomized patients, (n=142 on ZOLOFT and

n=139 on placebo), ZOLOFT treatment was initiated at 50 mg/day in the late luteal phase (last 2 weeks) of each menstrual cycle and then discontinued at the onset of menses (intermittent dosing). In subsequent cycles, patients were dosed in the range of 50-100 mg/day in the luteal phase of each cycle, on the basis of clinical response and tolerability. Patients who received 100 mg/day started with 50 mg/day for the first 3 days of the cycle, then 100 mg/day for the remainder of the cycle. The mean ZOLOFT dose for completers was 74 mg/day. ZOLOFT administered in the late luteal phase of the menstrual cycle was statistically significantly more effective than placebo on change from baseline to endpoint on the DRSP total score and the CGI-S score, as well as the CGI-I score at endpoint (Week 12).

There was insufficient information to determine the effect of race or age on outcome in these studies.

16 HOW SUPPLIED/STORAGE AND HANDLING

ZOLOFT 25 mg tablets: light green, film-coated, capsular-shaped tablets engraved on one side with “ZOLOFT” and on the other side scored and engraved with “25 mg”

NDC 0049-4960-30 Bottles of 30
NDC 0049-4960-50 Bottles of 50

ZOLOFT 50 mg tablets: light blue, film-coated, capsular-shaped tablets engraved on one side with “ZOLOFT” and on the other side scored and engraved with “50 mg”

NDC 0049-4900-30 Bottles of 30
NDC 0049-4900-66 Bottles of 100
NDC 0049-4900-73 Bottles of 500
NDC 0049-4900-94 Bottles of 5000
NDC 0049-4900-41 Unit Dose Packages of 100

ZOLOFT 100 mg tablets: light yellow, film-coated, capsular-shaped, tablets engraved on one side with “ZOLOFT” and on the other side scored and engraved with “100 mg”

NDC 0049-4910-30 Bottles of 30
NDC 0049-4910-66 Bottles of 100
NDC 0049-4910-73 Bottles of 500
NDC 0049-4910-94 Bottles of 5000
NDC 0049-4910-41 Unit Dose Packages of 100

ZOLOFT oral solution: clear, colorless solution with a menthol scent containing sertraline hydrochloride equivalent to 20 mg of sertraline per mL and 12% alcohol

NDC 0049-0050-01 Bottles containing 60 mL, each with an accompanying calibrated dropper that has 25 mg and 50 mg graduation marks.

Store ZOLOFT at 20°C to 25°C (68°F to 77°F); excursions permitted to 15°C to 30°C (59°F to 86°F) [See USP Controlled Room Temperature].

17 PATIENT COUNSELING INFORMATION

Advise the patient to read the FDA-approved patient labeling (Medication Guide).

Suicidal Thoughts and Behaviors

Advise patients and caregivers to look for the emergence of suicidality, especially early during treatment and when the dosage is adjusted up or down, and instruct them to report such symptoms to the healthcare provider *[See Boxed Warning and Warnings and Precautions (5.1)]*.

Important Administration Instructions for Oral Solution

For patients prescribed ZOLOFT oral solution, inform them that:

- ZOLOFT oral solution must be diluted before use. Do not mix in advance.
- Use the dropper provided to remove the required amount of ZOLOFT oral solution and mix with 4 ounces (1/2 cup) of water, ginger ale, lemon/lime soda, lemonade or orange juice ONLY. Do not mix ZOLOFT oral solution with anything other than the liquids listed.
- Take the dose immediately after mixing. At times, a slight haze may appear after mixing; this is normal.
- The dropper dispenser contains dry natural rubber, a consideration for patients with latex sensitivity.

Disulfiram Contraindication for ZOLOFT Oral Solution

Inform patients not to take disulfiram when taking ZOLOFT oral solution. Concomitant use is contraindicated due the alcohol content of the oral solution *[See Contraindication (4)]*.

Serotonin Syndrome

Caution patients about the risk of serotonin syndrome, particularly with the concomitant use of ZOLOFT with other serotonergic drugs including triptans, tricyclic antidepressants, fentanyl, lithium, tramadol, tryptophan, buspirone, amphetamines, St. John's Wort, and with drugs that impair metabolism of serotonin (in particular, MAOIs, both those intended to treat psychiatric disorders and also others, such as linezolid). Patients should contact their health care provider or report to the emergency room if they experience signs or symptoms of serotonin syndrome *[See Warnings and Precautions (5.2), Drug Interactions (7.1)]*.

Increased Risk of Bleeding

Inform patients about the concomitant use of ZOLOFT with aspirin, NSAIDs, other antiplatelet drugs, warfarin, or other anticoagulants because the combined use has been associated with an increased risk of bleeding. Advise patients to inform their health care providers if they are taking or planning to take any prescription or over-the-counter medications that increase the risk of bleeding *[See Warnings and Precautions (5.3)]*.

Activation of Mania/Hypomania

Advise patients and their caregivers to observe for signs of activation of mania/hypomania and instruct them to report such symptoms to the healthcare provider *[See Warnings and Precautions (5.4)]*.

Discontinuation Syndrome

Advise patients not to abruptly discontinue ZOLOFT and to discuss any tapering regimen with their healthcare provider. Adverse reactions can occur when ZOLOFT is

discontinued *[See Warnings and Precautions (5.5)]*.

Sexual Dysfunction

Advise patients that use of ZOLOFT may cause symptoms of sexual dysfunction in both male and female patients. Inform patients that they should discuss any changes in sexual function and potential management strategies with their healthcare provider *[see Warnings and Precautions (5.11)]*.

Allergic Reactions

Advise patients to notify their healthcare provider if they develop an allergic reaction such as rash, hives, swelling, or difficulty breathing *[See Adverse Reactions (6.2)]*.

Pregnancy

Inform pregnant women that ZOLOFT may cause withdrawal symptoms in the newborn or persistent pulmonary hypertension of the newborn (PPHN) *[See Use in Specific Populations (8.1)]*.

This product's label may have been updated. For current full prescribing information, please visit www.zoloft.com.

Distributed by:
Viatris Specialty LLC
Morgantown, WV 26505 U.S.A.

UPJ:ZLFTTOS:RX1

Medication Guide

**ZOLOFT (ZOH-loft)**<br>**(sertraline hydrochloride)**<br>**Tablets and Oral Solution**
**What is the most important information I should know about ZOLOFT?**<br>**ZOLOFT and other antidepressant medicines may cause serious side effects. Call your healthcare provider right away if you have any of the following symptoms, or call 911 if there is an emergency.**<br><br>1. **Suicidal thoughts or actions:**<br>• **ZOLOFT and other antidepressant medicines may increase suicidal thoughts or actions** in some people 24 years of age and younger, especially within the **first few months of treatment or when the dose is changed.**<br>• Depression or other serious mental illnesses are the most important causes of suicidal thoughts or actions.<br>• Watch for these changes and call your healthcare provider right away if you notice new or sudden changes in mood, behavior, actions, thoughts, or feelings, especially if severe.<br><br>o Pay particular attention to such changes when ZOLOFT is started or when the dose is changed.<br>o Keep all follow-up visits with your healthcare provider and call between visits if you are worried about symptoms.

Call your healthcare provider right away if you have any of the following symptoms, or call 911 if an emergency, especially if they are new, worse, or worry you:

- attempts to commit suicide
- acting aggressive or violent
- new or worse depression
- feeling agitated, restless, angry or irritable
- an increase in activity or talking more than what is normal for you
- acting on dangerous impulses
- thoughts about suicide or dying
- new or worse anxiety or panic attacks
- trouble sleeping
- other unusual changes in behavior or mood

2. **Serotonin Syndrome.** This condition can be life-threatening and symptoms may include:

- agitation, hallucinations, coma, or other changes in mental status
- racing heartbeat, high or low blood pressure
- coordination problems or muscle twitching (overactive reflexes)
- nausea, vomiting, or diarrhea
- sweating or fever
- muscle rigidity

3. **Increased chance of bleeding:** ZOLOFT and other antidepressant medicines may increase your risk of bleeding or bruising, especially if you take the blood thinner warfarin (Coumadin®, Jantoven®), a non-steroidal anti-inflammatory drug (NSAIDs, like ibuprofen or naproxen), or aspirin.
4. **Manic episodes.** Symptoms may include:

- greatly increased energy
- racing thoughts
- unusually grand ideas
- severe trouble sleeping
- reckless behavior
- excessive happiness or irritability
- talking more or faster than usual

5. **Seizures or convulsions.**
6. **Glaucoma (angle-closure glaucoma).** Many antidepressant medicines including ZOLOFT may cause a certain type of eye problem called angle-closure glaucoma. Call your healthcare provider if you have eye pain, changes in your vision, or swelling or redness in or around the eye. Only some people are at risk for these problems. You may want to undergo an eye examination to see if you are at risk and receive preventative treatment if you are.
7. **Changes in appetite or weight.** Children and adolescents should have height and weight monitored during treatment.
8. **Low salt (sodium) levels in the blood.** Elderly people may be at greater risk for this. Symptoms may include:

- headache
- weakness or feeling unsteady
- confusion, problems concentrating or thinking, or memory problems

9. **Sexual problems (dysfunction).** Taking selective serotonin reuptake inhibitors (SSRIs), including ZOLOFT, may cause sexual problems.

Symptoms in males may include:

- Delayed ejaculation or inability to have an ejaculation
- Decreased sex drive
- Problems getting or keeping an erection

Symptoms in females may include:

- Decreased sex drive
- Delayed orgasm or inability to have an orgasm

Talk to your healthcare provider if you develop any changes in your sexual function or if you have any questions or concerns about sexual problems during treatment with ZOLOFT. There may be treatments your healthcare provider can suggest.
Do not stop ZOLOFT without first talking to your healthcare provider. Stopping ZOLOFT too quickly may cause serious symptoms including:

- anxiety, irritability, high or low mood, feeling restless or changes in sleep habits
- headache, sweating, nausea, dizziness
- electric shock-like sensations, shaking, confusion

What is ZOLOFT?
ZOLOFT is a prescription medicine used to treat:

- Major Depressive Disorder (MDD)
- Panic Disorder
- Social Anxiety Disorder
- Obsessive Compulsive Disorder (OCD)
- Posttraumatic Stress Disorder (PTSD)
- Premenstrual Dysphoric Disorder (PMDD)

It is important to talk with your healthcare provider about the risks of treating depression and also the risks of not treating it. You should discuss all treatment choices with your healthcare provider.
ZOLOFT is safe and effective in treating children with OCD age 6 to 17 years.
It is not known if ZOLOFT is safe and effective for use in children under 6 years of age with OCD or children with other behavior health conditions.
Talk to your healthcare provider if you do not think that your condition is getting better with ZOLOFT treatment.

Who should not take ZOLOFT?
Do not take ZOLOFT if you:

- take a monoamine oxidase inhibitor (MAOI). Ask your healthcare provider or

pharmacist if you are not sure if you take an MAOI, including the antibiotic linezolid.

- have taken an MAOI within 2 weeks of stopping ZOLOFT unless directed to do so by your healthcare provider.
- have stopped taking an MAOI in the last 2 weeks unless directed to do so by your healthcare provider.
- take any other medicines that contain sertraline (such as sertraline HCl or sertraline hydrochloride).
- take the antipsychotic medicine pimozide (Orap®) because this can cause serious heart problems.
- are allergic to sertraline or any of the ingredients in ZOLOFT. See the end of this Medication Guide for a complete list of ingredients in ZOLOFT.
- take Antabuse® (disulfiram) (if you are taking the liquid form of ZOLOFT) due to the alcohol content.

People who take ZOLOFT close in time to an MAOI may have serious or even life-threatening side effects. Get medical help right away if you have any of these symptoms:

- high fever
- rapid changes in heart rate or blood pressure
- uncontrolled muscle spasms
- confusion
- stiff muscles
- loss of consciousness (pass out)

What should I tell my healthcare provider before taking ZOLOFT?
Before starting ZOLOFT, tell your healthcare provider:

- **if you have:**

- liver problems
- heart problems
- bipolar disorder or mania
- kidney problems.
- or have had seizures or convulsions
- low sodium levels in your blood
- a history of a stroke
- high blood pressure
- or have had bleeding problems

- **are pregnant or plan to become pregnant.** Your baby may have withdrawal symptoms after birth or may be at increased risk for a serious lung problem at birth. Talk to your healthcare provider about the benefits and risks of taking ZOLOFT during pregnancy.
- **are breastfeeding or plan to breastfeed.** A small amount of ZOLOFT may pass into your breast milk. Talk to your healthcare provider about the best way to feed your baby while taking ZOLOFT.

Tell your healthcare provider about all the medicines that you take, including prescription and over-the-counter medicines, vitamins, and herbal supplements. ZOLOFT and some medicines may interact with each other, may not work as well, or may cause serious side effects.
Your healthcare provider or pharmacist can tell you if it is safe to take ZOLOFT with your other medicines. **Do not** start or stop any medicine while taking ZOLOFT without talking

to your healthcare provider first.

How should I take ZOLOFT?

- Take ZOLOFT exactly as prescribed. Your healthcare provider may need to change the dose of ZOLOFT until it is the right dose for you.
- ZOLOFT Tablets may be taken with or without food.
- ZOLOFT Oral Solution may look cloudy or hazy after mixing, this is normal.
- ZOLOFT Oral Solution must be diluted before use:
    - o **Do not** mix ZOLOFT until you are ready to take it.
    - o When diluting ZOLOFT Oral Solution, use **only** water, ginger ale, lemon/lime soda, lemonade, or orange juice.
    - o The oral dropper contains latex. If you are sensitive or allergic to latex, ask your healthcare provider or pharmacist about the best way to measure your medicine.
- If you miss a dose of ZOLOFT, take the missed dose as soon as you remember. If it is almost time for the next dose, skip the missed dose and take your next dose at the regular time. **Do not** take two doses of ZOLOFT at the same time.

If you take too much ZOLOFT, call your healthcare provider or poison control center right away, or go to the nearest hospital emergency room right away.

What should I avoid while taking ZOLOFT?

ZOLOFT can cause sleepiness or may affect your ability to make decisions, think clearly, or react quickly. You should not drive, operate heavy machinery, or do other dangerous activities until you know how ZOLOFT affects you. **Do not** drink alcohol while you take ZOLOFT.

What are the possible side effects of ZOLOFT?

ZOLOFT may cause serious side effects, including:

- **See "What is the most important information I should know about ZOLOFT?"**

The most common side effects in adults who take ZOLOFT include:

- nausea, loss of appetite, diarrhea, or indigestion
- increased sweating
- tremor or shaking
- agitation
- change in sleep habits including increased sleepiness or insomnia
- sexual problems including decreased libido and ejaculation failure
- feeling tired or fatigued
- anxiety

The most common side effects in children and adolescents who take include abnormal increase in muscle movement or agitation, nose bleeds, urinary incontinence, aggressive reaction, possible slowed growth rate, and weight change. Your child's height and weight should be monitored during treatment with ZOLOFT.

Tell your healthcare provider if you have any side effect that bothers you or that does not go away. These are not all the possible side effects of ZOLOFT. For more information, ask your healthcare provider or pharmacist.

Call your doctor for medical advice about side effects. You may report side effects to

the FDA at 1-800-FDA-1088.

How should I store ZOLOFT?

- Store ZOLOFT at room temperature, 68°F to 77°F (20°C to 25°C).
- Keep ZOLOFT bottle closed tightly.

Keep ZOLOFT and all medicines out of the reach of children.

General information about the safe and effective use of ZOLOFT
Medicines are sometimes prescribed for purposes other than those listed in a Medication Guide. Do not use ZOLOFT for a condition for which it was not prescribed. Do not give ZOLOFT to other people, even if they have the same condition. It may harm them.
This Medication Guide summarizes the most important information about ZOLOFT. If you would like more information, talk with your healthcare provider. You may ask your healthcare provider or pharmacist for information about ZOLOFT that is written for healthcare professionals.
For more information about ZOLOFT call 1-877-446-3679 (1-877-4-INFO-RX) or go to www.zoloft.com

What are the ingredients in ZOLOFT?
Active ingredient: sertraline hydrochloride
Inactive ingredients:
Tablets: dibasic calcium phosphate dihydrate, D&C Yellow #10 aluminum lake (in 25 mg tablet), FD&C Blue #1 aluminum lake (in 25 mg tablet), FD&C Red #40 aluminum lake (in 25 mg tablet), FD&C Blue #2 aluminum lake (in 50 mg tablet), hydroxypropyl cellulose, hypromellose, magnesium stearate, microcrystalline cellulose, polyethylene glycol, polysorbate 80, sodium starch glycolate, synthetic yellow iron oxide (in 100 mg tablet), and titanium dioxide.
Oral solution: glycerin, alcohol (12%), menthol, butylated hydroxytoluene (BHT)

This Medication Guide has been approved by the U.S. Food and Drug Administration   Revised: 1/2023

Distributed by:
Viatris Specialty LLC
Morgantown, WV 26505 U.S.A.

UPJ:MG:ZLFTTOS:RX1

PRINCIPAL DISPLAY PANEL - 25 mg Tablet Bottle Label

ALWAYS DISPENSE WITH MEDICATION GUIDE

NDC 0049-4960-30

Pfizer

Zoloft ®
(sertraline hydrochloride)
tablets

25 mg*

30 Tablets
Rx only

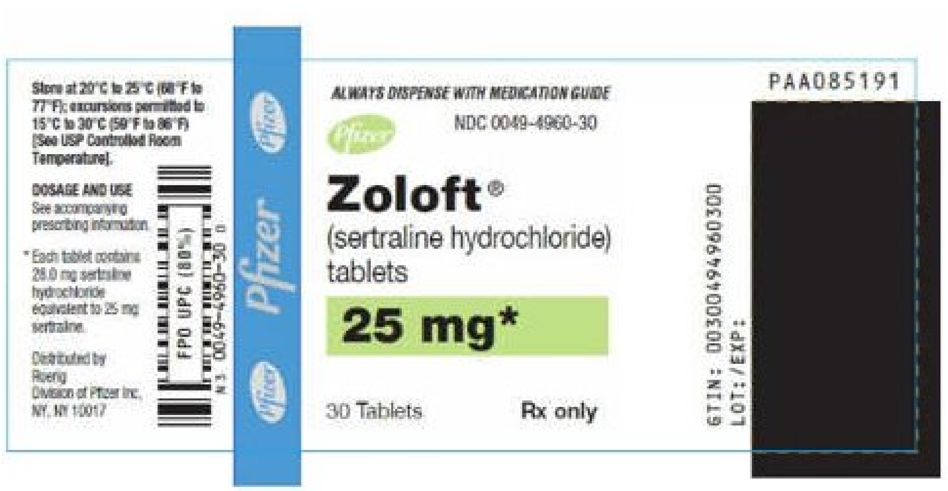

PRINCIPAL DISPLAY PANEL - 50 mg Tablet Bottle Label

ALWAYS DISPENSE WITH MEDICATION GUIDE

NDC 0049-4900-30

Pfizer

Zoloft ®
(sertraline hydrochloride)
tablets

50 mg*

30 Tablets
Rx only

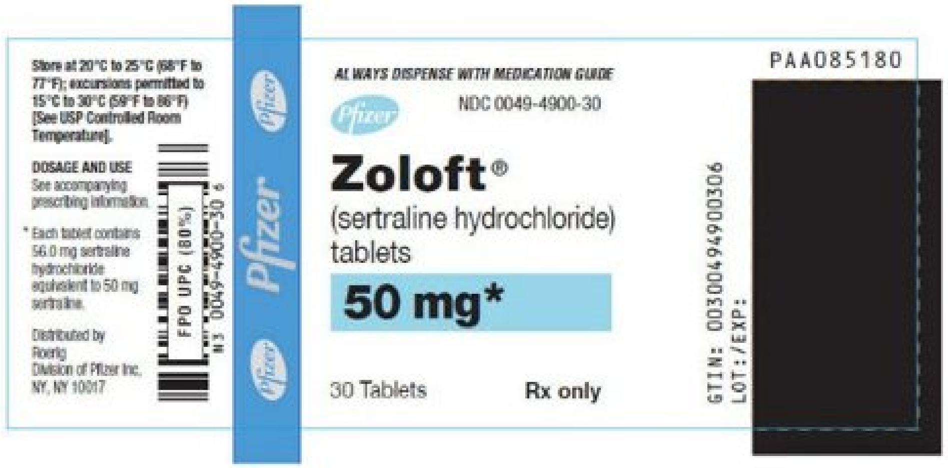

PRINCIPAL DISPLAY PANEL - 50 mg Tablet Blister Pack

Zoloft®
(sertraline HCl) Tablet

50 mg

DISTRIBUTED BY ROERIG
DIV OF PFIZER INC, NY, NY 10017

PAA044551

EXP &
LOT AREA

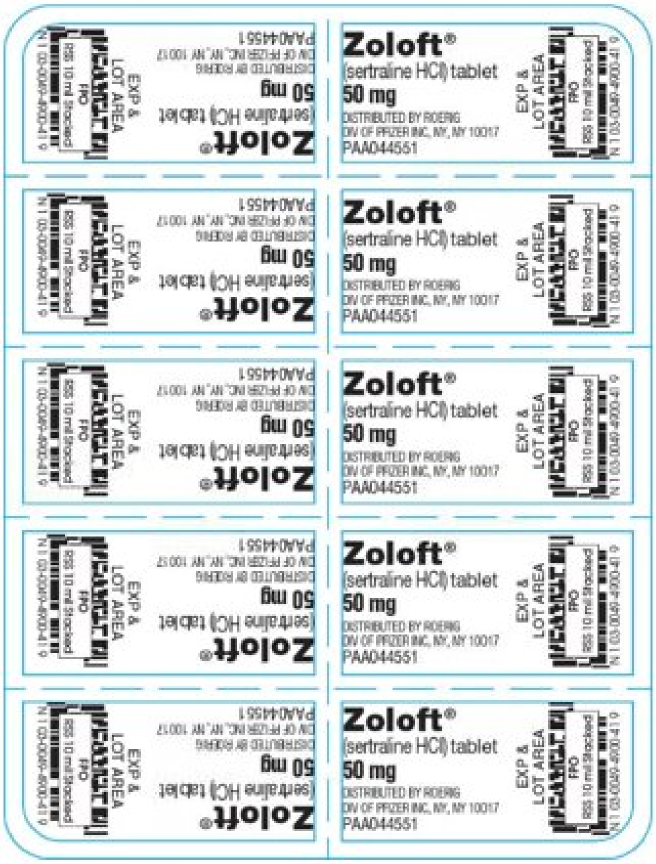

PRINCIPAL DISPLAY PANEL - 50 mg Tablet Blister Pack Carton

UNIT DOSE

ALWAYS DISPENSE WITH MEDICATION GUIDE

NDC 0049-4900-41

Pfizer

Zoloft ®
(sertraline hydrochloride)
tablets

50 mg *

For in-institution use only

100 Tablets
Rx only

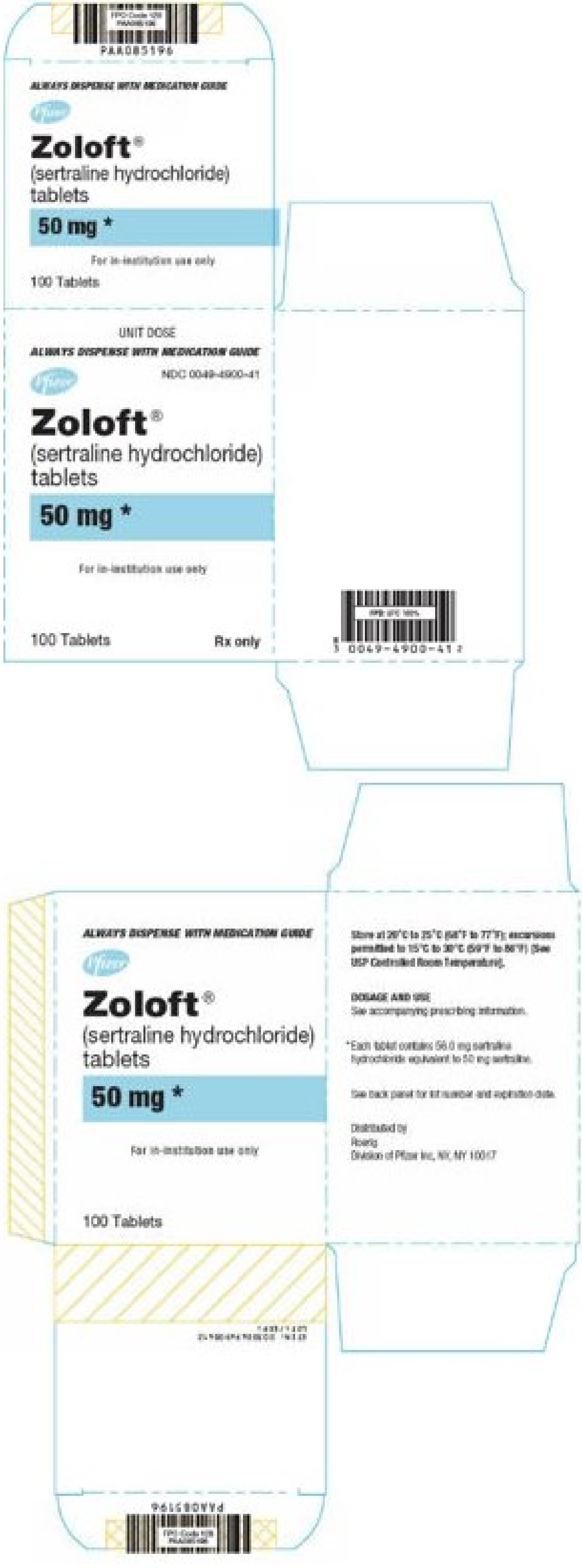

PRINCIPAL DISPLAY PANEL - 100 mg Tablet Bottle Label

ALWAYS DISPENSE WITH MEDICATION GUIDE

NDC 0049-4910-30

Pfizer

Zoloft ®
(sertraline hydrochloride)
tablets

100 mg*

30 Tablets
Rx only

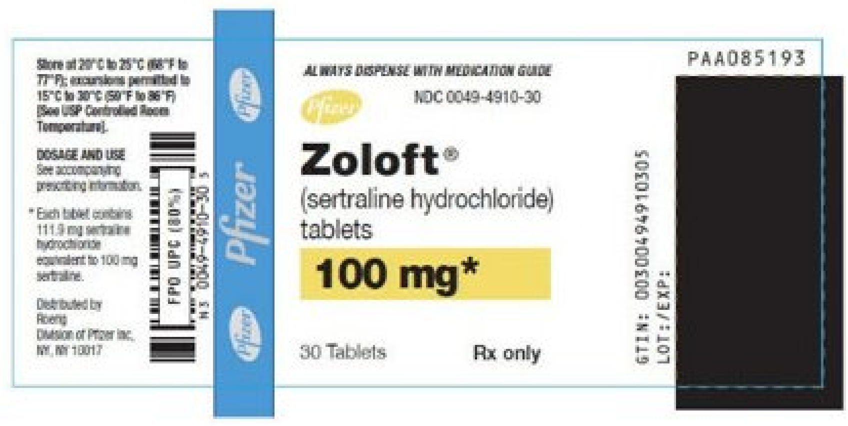

PRINCIPAL DISPLAY PANEL - 100 mg Tablet Blister Pack

Zoloft®
(sertraline HCl) Tablet

100 mg

DISTRIBUTED BY ROERIG
DIV OF PFIZER INC, NY, NY 10017

PAA044548

EXP &
LOT AREA

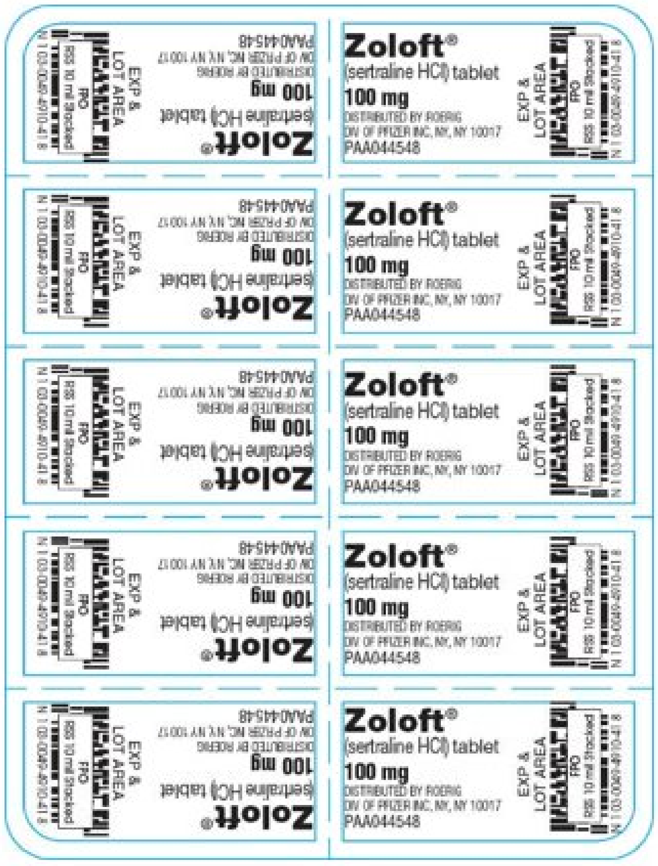

PRINCIPAL DISPLAY PANEL - 100 mg Tablet Blister Pack Carton

UNIT DOSE

ALWAYS DISPENSE WITH MEDICATION GUIDE

NDC 0049-4910-41

Pfizer

Zoloft®

(sertraline hydrochloride)

tablets

100 mg*

For in-institution use only

100 Tablets

Rx only

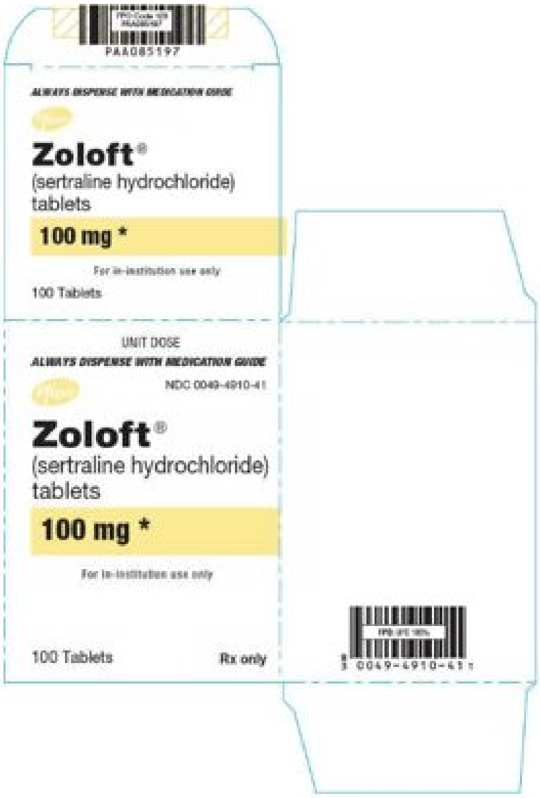

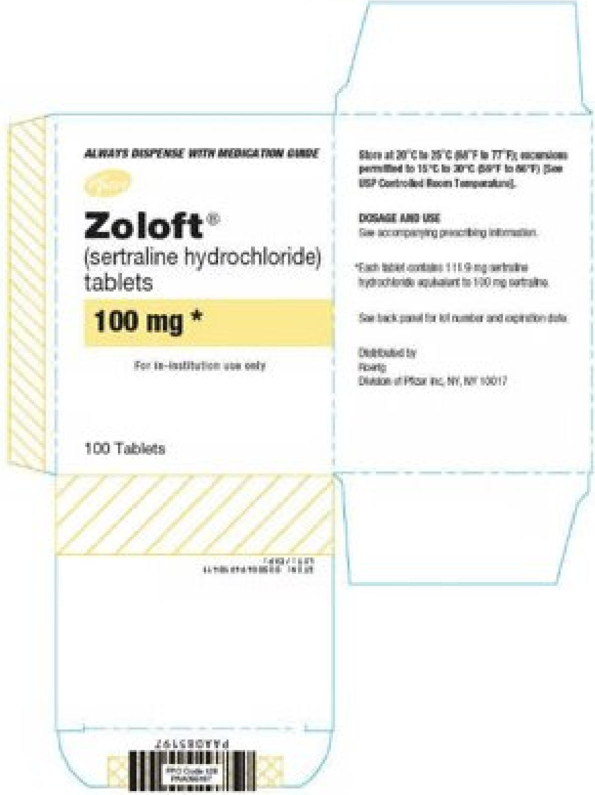

PRINCIPAL DISPLAY PANEL - 60 mL Bottle Label

Pfizer

ALWAYS DISPENSE WITH MEDICATION GUIDE

NDC 0049-0050-01

Zoloft®
(sertraline hydrochloride)
oral solution
equivalent to
20 mg/mL*
of sertraline

Must Be Diluted Before Use
(see side panel for instructions)

60 mL
Rx only

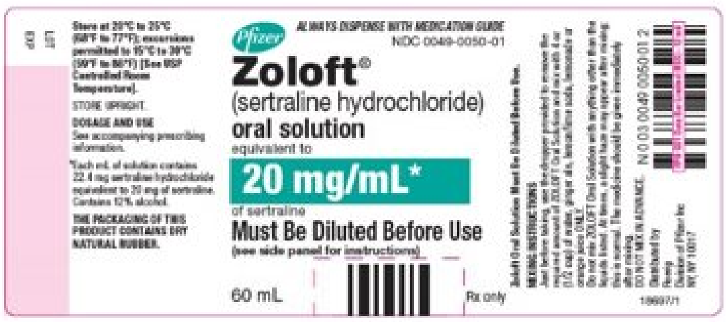

PRINCIPAL DISPLAY PANEL - 60 mL Bottle Carton

ALWAYS DISPENSE WITH MEDICATION GUIDE

Pfizer
NDC 0049-0050-01

Zoloft®
(sertraline hydrochloride)
oral solution
equivalent to
20 mg/mL*
of sertraline

Must Be Diluted
Before Use
(see side panel for instructions)

CALIBRATED DROPPER ENCLOSED

60 mL
Rx only

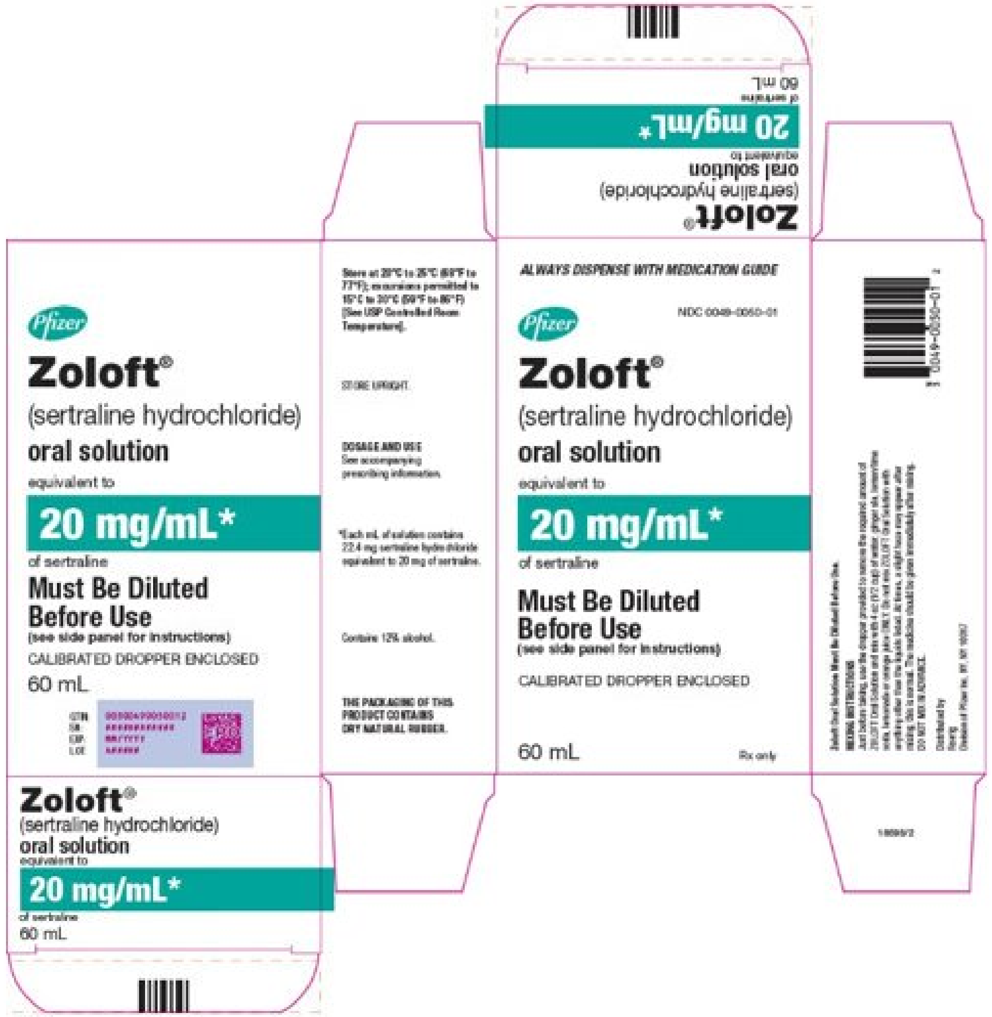

ZOLOFT

sertraline hydrochloride tablet, film coated

Product Information			
**Product Type**	HUMAN PRESCRIPTION DRUG	**Item Code (Source)**	NDC:0049-4960
**Route of Administration**	ORAL		

Active Ingredient/Active Moiety		
**Ingredient Name**	**Basis of Strength**	**Strength**
**SERTRALINE HYDROCHLORIDE** (UNII: UTI8907Y6X) (SERTRALINE - UNII:QUC7NX6WMB)	SERTRALINE	25 mg

Inactive Ingredients	
**Ingredient Name**	**Strength**
**DIBASIC CALCIUM PHOSPHATE DIHYDRATE** (UNII: O7TSZ97GEP)	
**D&C YELLOW NO. 10** (UNII: 35SW5USQ3G)	
**FD&C BLUE NO. 1** (UNII: H3R47K3TBD)	

**FD&C RED NO. 40** (UNII: WZB9127XOA)	
**HYDROXYPROPYL CELLULOSE (1600000 WAMW)** (UNII: RFW2ET671P)	
**HYPROMELLOSE, UNSPECIFIED** (UNII: 3NXW29V3WO)	
**MAGNESIUM STEARATE** (UNII: 70097M6I30)	
**MICROCRYSTALLINE CELLULOSE** (UNII: OP1R32D61U)	
**POLYETHYLENE GLYCOL, UNSPECIFIED** (UNII: 3WJQ0SDW1A)	
**POLYSORBATE 80** (UNII: 6OZP39ZG8H)	
**TITANIUM DIOXIDE** (UNII: 15FIX9V2JP)	
**ALUMINUM OXIDE** (UNII: LMI26O6933)	
**SODIUM STARCH GLYCOLATE TYPE A POTATO** (UNII: 5856J3G2A2)	

Product Characteristics

**Color**	GREEN (light green)	**Score**	2 pieces
**Shape**	OVAL (CAPSULE-SHAPED)	**Size**	8mm
**Flavor**		**Imprint Code**	ZOLOFT;25;mg
**Contains**			

Packaging

#	Item Code	Package Description	Marketing Start Date	Marketing End Date
1	NDC:0049-4960-30	30 in 1 BOTTLE; Type 0: Not a Combination Product	02/11/1992	10/31/2025
2	NDC:0049-4960-50	50 in 1 BOTTLE; Type 0: Not a Combination Product	02/11/1992	07/30/2012

Marketing Information

Marketing Category	Application Number or Monograph Citation	Marketing Start Date	Marketing End Date
NDA	NDA019839	02/11/1992	10/31/2025

ZOLOFT

sertraline hydrochloride tablet, film coated

Product Information

**Product Type**	HUMAN PRESCRIPTION DRUG	**Item Code (Source)**	NDC:0049-4900
**Route of Administration**	ORAL		

Active Ingredient/Active Moiety

Ingredient Name	Basis of Strength	Strength
**SERTRALINE HYDROCHLORIDE** (UNII: UTI8907Y6X) (SERTRALINE - UNII:QUC7NX6WMB)	SERTRALINE	50 mg

Inactive Ingredients

Ingredient Name	Strength
**DIBASIC CALCIUM PHOSPHATE DIHYDRATE** (UNII: O7TSZ97GEP)	
**FD&C BLUE NO. 2** (UNII: L06K8R7DQK)	
**HYDROXYPROPYL CELLULOSE (1600000 WAMW)** (UNII: RFW2ET671P)	
**HYPROMELLOSE, UNSPECIFIED** (UNII: 3NXW29V3WO)	
**MAGNESIUM STEARATE** (UNII: 70097M6I30)	
**MICROCRYSTALLINE CELLULOSE** (UNII: OP1R32D61U)	
**POLYETHYLENE GLYCOL, UNSPECIFIED** (UNII: 3WJQ0SDW1A)	
**POLYSORBATE 80** (UNII: 6OZP39ZG8H)	
**TITANIUM DIOXIDE** (UNII: 15FIX9V2JP)	
**ALUMINUM OXIDE** (UNII: LMI26O6933)	
**SODIUM STARCH GLYCOLATE TYPE A POTATO** (UNII: 5856J3G2A2)	

Product Characteristics

**Color**	BLUE (light blue)	**Score**	2 pieces
**Shape**	OVAL (CAPSULE-SHAPED)	**Size**	10mm
**Flavor**		**Imprint Code**	ZOLOFT;50;mg
**Contains**			

Packaging

#	Item Code	Package Description	Marketing Start Date	Marketing End Date
1	NDC:0049-4900-30	30 in 1 BOTTLE; Type 0: Not a Combination Product	02/11/1992	
2	NDC:0049-4900-66	100 in 1 BOTTLE; Type 0: Not a Combination Product	02/11/1992	07/30/2012
3	NDC:0049-4900-73	500 in 1 BOTTLE; Type 0: Not a Combination Product	02/11/1992	07/30/2012
4	NDC:0049-4900-94	5000 in 1 BOTTLE; Type 0: Not a Combination Product	02/11/1992	07/30/2012
5	NDC:0049-4900-41	100 in 1 CARTON	02/11/1992	
5		1 in 1 BLISTER PACK; Type 0: Not a Combination Product		

Marketing Information

Marketing Category	Application Number or Monograph Citation	Marketing Start Date	Marketing End Date
NDA	NDA019839	02/11/1992	

ZOLOFT

sertraline hydrochloride tablet, film coated

Product Information

**Product Type**	HUMAN PRESCRIPTION DRUG	**Item Code (Source)**	NDC:0049-4910
**Route of Administration**	ORAL		

**Active Ingredient/Active Moiety**		
**Ingredient Name**	**Basis of Strength**	**Strength**
**SERTRALINE HYDROCHLORIDE** (UNII: UTI8907Y6X) (SERTRALINE - UNII:QUC7NX6WMB)	SERTRALINE	100 mg

**Inactive Ingredients**	
**Ingredient Name**	**Strength**
**DIBASIC CALCIUM PHOSPHATE DIHYDRATE** (UNII: O7TSZ97GEP)	
**HYDROXYPROPYL CELLULOSE (1600000 WAMW)** (UNII: RFW2ET671P)	
**HYPROMELLOSE, UNSPECIFIED** (UNII: 3NXW29V3WO)	
**MAGNESIUM STEARATE** (UNII: 70097M6I30)	
**MICROCRYSTALLINE CELLULOSE** (UNII: OP1R32D61U)	
**POLYETHYLENE GLYCOL, UNSPECIFIED** (UNII: 3WJQ0SDW1A)	
**POLYSORBATE 80** (UNII: 6OZP39ZG8H)	
**FERRIC OXIDE YELLOW** (UNII: EX438O2MRT)	
**TITANIUM DIOXIDE** (UNII: 15FIX9V2JP)	
**SODIUM STARCH GLYCOLATE TYPE A POTATO** (UNII: 5856J3G2A2)	

**Product Characteristics**			
**Color**	YELLOW (light yellow)	**Score**	2 pieces
**Shape**	OVAL (CAPSULE-SHAPED)	**Size**	13mm
**Flavor**		**Imprint Code**	ZOLOFT;100;mg
**Contains**			

Packaging

#	Item Code	Package Description	Marketing Start Date	Marketing End Date
1	NDC:0049-4910-30	30 in 1 BOTTLE; Type 0: Not a Combination Product	02/11/1992	
2	NDC:0049-4910-66	100 in 1 BOTTLE; Type 0: Not a Combination Product	02/11/1992	07/30/2012
3	NDC:0049-4910-73	500 in 1 BOTTLE; Type 0: Not a Combination Product	02/11/1992	07/30/2012
4	NDC:0049-4910-94	5000 in 1 BOTTLE; Type 0: Not a Combination Product	02/11/1992	07/30/2012
5	NDC:0049-4910-41	100 in 1 CARTON	02/11/1992	
5		1 in 1 BLISTER PACK; Type 0: Not a Combination Product		

Marketing Information

Marketing Category	Application Number or Monograph Citation	Marketing Start Date	Marketing End Date
NDA	NDA019839	02/11/1992	

ZOLOFT

sertraline hydrochloride solution, concentrate

Product Information			
**Product Type**	HUMAN PRESCRIPTION DRUG	**Item Code (Source)**	NDC:0049-0050
**Route of Administration**	ORAL		

Active Ingredient/Active Moiety

Ingredient Name	Basis of Strength	Strength
**SERTRALINE HYDROCHLORIDE** (UNII: UTI8907Y6X) (SERTRALINE - UNII:QUC7NX6WMB)	SERTRALINE	20 mg in 1 mL

Inactive Ingredients

Ingredient Name	Strength
**GLYCERIN** (UNII: PDC6A3C0OX)	
**ALCOHOL** (UNII: 3K9958V90M)	
**MENTHOL, UNSPECIFIED FORM** (UNII: L7T10EIP3A)	
**BUTYLATED HYDROXYTOLUENE** (UNII: 1P9D0Z171K)	

Packaging

#	Item Code	Package Description	Marketing Start Date	Marketing End Date
1	NDC:0049-0050-01	1 in 1 CARTON	01/06/2017	
1		60 mL in 1 BOTTLE, DROPPER; Type 1: Convenience Kit of Co-Package		

Marketing Information

Marketing Category	Application Number or Monograph Citation	Marketing Start Date	Marketing End Date
NDA	NDA020990	01/06/2017	

Labeler - ROERIG (829076996)

REGULATORY GUIDANCES

Code of Federal Regulations, Title 21: Food and Drugs (21 CFR 201.57)

This content is from the eCFR and is authoritative but unofficial.

Title 21 —Food and Drugs
Chapter I —Food and Drug Administration, Department of Health and Human Services
Subchapter C —Drugs: General
Part 201 —Labeling
Subpart B —Labeling Requirements for Prescription Drugs and/or Insulin

Authority: 21 U.S.C. 321, 331, 343, 351, 352, 353, 355, 358, 360, 360b, 360ccc, 360ccc-1, 360ee, 360gg-360ss, 371, 374, 379e; 42 U.S.C. 216, 241, 262, 264.

Source: 40 FR 13998, Mar. 27, 1975, unless otherwise noted.

Editorial Note: Nomenclature changes to part 201 appear at 69 FR 13717, Mar. 24, 2004.

§ 201.57 Specific requirements on content and format of labeling for human prescription drug and biological products described in § 201.56(b)(1).

The requirements in this section apply only to prescription drug products described in § 201.56(b)(1) and must be implemented according to the schedule specified in § 201.56(c), except for the requirement in paragraph (c)(18) of this section to reprint any FDA-approved patient labeling at the end of prescription drug labeling or accompany the prescription drug labeling, which must be implemented no later than June 30, 2007.

(a) ***Highlights of prescribing information.*** The following information must appear in all prescription drug labeling:

(1) ***Highlights limitation statement.*** The verbatim statement “These highlights do not include all the information needed to use (*insert name of drug product*) safely and effectively. See full prescribing information for (insert name of drug product).”

(2) ***Drug names, dosage form, route of administration, and controlled substance symbol.*** The proprietary name and the established name of the drug, if any, as defined in section 502(e)(3) of the Federal Food, Drug, and Cosmetic Act (the act) or, for biological products, the proper name (as defined in § 600.3 of this chapter) including any appropriate descriptors. This information must be followed by the drug's dosage form and route of administration. For controlled substances, the controlled substance symbol designating the schedule in which the controlled substance is listed must be included as required by § 1302.04 of this chapter.

(3) ***Initial U.S. approval.*** The verbatim statement “Initial U.S. Approval” followed by the four-digit year in which FDA initially approved a new molecular entity, new biological product, or new combination of active ingredients. The statement must be placed on the line immediately beneath the established name or, for biological products, proper name of the product.

(4) ***Boxed warning.*** A concise summary of any boxed warning required by paragraph (c)(1) of this section, not to exceed a length of 20 lines. The summary must be preceded by a heading, in upper-case letters, containing the word “WARNING” and other words that are appropriate to identify the subject of the warning. The heading and the summary must be contained within a box and bolded. The following verbatim statement must be placed immediately following the heading of the boxed warning: “See full prescribing information for complete boxed warning.”

(5) *Recent major changes.* A list of the section(s) of the full prescribing information, limited to the labeling sections described in paragraphs (c)(1), (c)(2), (c)(3), (c)(5), and (c)(6) of this section, that contain(s) substantive labeling changes that have been approved by FDA or authorized under § 314.70(c)(6) or (d)(2), or § 601.12(f)(1) through (f)(3) of this chapter. The heading(s) and, if appropriate, the subheading(s) of the labeling section(s) affected by the change must be listed together with each section's identifying number and the date (month/year) on which the change was incorporated in labeling. These labeling sections must be listed in the order in which they appear in the full prescribing information. A changed section must be listed under this heading in Highlights for at least 1 year after the date of the labeling change and must be removed at the first printing subsequent to the 1 year period.

(6) *Indications and usage.* A concise statement of each of the product's indications, as required under paragraph (c)(2) of this section, with any appropriate subheadings. Major limitations of use (e.g., lack of effect in particular subsets of the population, or second line therapy status) must be briefly noted. If the product is a member of an established pharmacologic class, the concise statement under this heading in Highlights must identify the class in the following manner: "(Drug) is a (name of class) indicated for (indication(s))."

(7) *Dosage and administration.* A concise summary of the information required under paragraph (c)(3) of this section, with any appropriate subheadings, including the recommended dosage regimen, starting dose, dose range, critical differences among population subsets, monitoring recommendations, and other clinically significant clinical pharmacologic information.

(8) *Dosage forms and strengths.* A concise summary of the information required under paragraph (c)(4) of this section, with any appropriate subheadings (e.g., tablets, capsules, injectable, suspension), including the strength or potency of the dosage form in metric system (e.g., 10-milligram tablets) and whether the product is scored.

(9) *Contraindications.* A concise statement of each of the product's contraindications, as required under paragraph (c)(5) of this section, with any appropriate subheadings.

(10) *Warnings and precautions.* A concise summary of the most clinically significant information required under paragraph (c)(6) of this section, with any appropriate subheadings, including information that would affect decisions about whether to prescribe a drug, recommendations for patient monitoring that are critical to safe use of the drug, and measures that can be taken to prevent or mitigate harm.

(11) *Adverse reactions.*

(i) A list of the most frequently occurring adverse reactions, as described in paragraph (c)(7) of this section, along with the criteria used to determine inclusion (e.g., incidence rate). Adverse reactions important for other reasons (e.g., because they are serious or frequently lead to discontinuation or dosage adjustment) must not be repeated under this heading in Highlights if they are included elsewhere in Highlights (e.g., Warnings and Precautions, Contraindications).

(ii) For drug products other than vaccines, the verbatim statement "To report SUSPECTED ADVERSE REACTIONS, contact (*insert name of manufacturer*) at (*insert manufacturer's phone number*) or FDA at (*insert current FDA phone number and Web address for voluntary reporting of adverse reactions*)."

(iii) For vaccines, the verbatim statement "To report SUSPECTED ADVERSE REACTIONS, contact (*insert name of manufacturer*) at (*insert manufacturer's phone number*) or VAERS at (*insert the current VAERS phone number and Web address for voluntary reporting of adverse reactions*)."

(iv) For manufacturers with a Web site for voluntary reporting of adverse reactions, the Web address of the direct link to the site.

(12) ***Drug interactions.*** A concise summary of the information required under paragraph (c)(8) of this section, with any appropriate subheadings.

(13) ***Use in specific populations.*** A concise summary of the information required under paragraph (c)(9) of this section, with any appropriate subheadings.

(14) ***Patient counseling information statement.*** The verbatim statement “See 17 for Patient Counseling Information” or, if the product has FDA-approved patient labeling, the verbatim statement “See 17 for Patient Counseling Information and (insert either FDA-approved patient labeling or Medication Guide).”

(15) ***Revision date.*** The date of the most recent revision of the labeling, identified as such, placed at the end of Highlights.

(b) ***Full prescribing information: Contents.*** Contents must contain a list of each heading and subheading required in the full prescribing information under § 201.56(d)(1), if not omitted under § 201.56(d)(4), preceded by the identifying number required under § 201.56(d)(1). Contents must also contain any additional subheading(s) included in the full prescribing information preceded by the identifying number assigned in accordance with § 201.56(d)(2).

(c) ***Full prescribing information.*** The full prescribing information must contain the information in the order required under paragraphs (c)(1) through (c)(18) of this section, together with the headings, subheadings, and identifying numbers required under § 201.56(d)(1), unless omitted under § 201.56(d)(4). If additional subheadings are used within a labeling section, they must be preceded by the identifying number assigned in accordance with § 201.56(d)(2).

(1) ***Boxed warning.*** Certain contraindications or serious warnings, particularly those that may lead to death or serious injury, may be required by the FDA to be presented in a box. The boxed warning ordinarily must be based on clinical data, but serious animal toxicity may also be the basis of a boxed warning in the absence of clinical data. The box must contain, in uppercase letters, a heading inside the box that includes the word “WARNING” and conveys the general focus of the information in the box. The box must briefly explain the risk and refer to more detailed information in the “Contraindications” or “Warnings and Precautions” section, accompanied by the identifying number for the section or subsection containing the detailed information.

(2) ***1 Indications and usage.*** This section must state that the drug is indicated for the treatment, prevention, mitigation, cure, or diagnosis of a recognized disease or condition, or of a manifestation of a recognized disease or condition, or for the relief of symptoms associated with a recognized disease or condition.

(i) This section must include the following information when the conditions listed are applicable:

(A) If the drug is used for an indication only in conjunction with a primary mode of therapy (e.g., diet, surgery, behavior changes, or some other drug), a statement that the drug is indicated as an adjunct to that mode of therapy.

(B) If evidence is available to support the safety and effectiveness of the drug or biological product only in selected subgroups of the larger population (e.g., patients with mild disease or patients in a special age group), or if the indication is approved based on a

surrogate endpoint under § 314.510 or § 601.41 of this chapter, a succinct description of the limitations of usefulness of the drug and any uncertainty about anticipated clinical benefits, with reference to the "Clinical Studies" section for a discussion of the available evidence.

(C) If specific tests are necessary for selection or monitoring of the patients who need the drug (e.g., microbe susceptibility tests), the identity of such tests.

(D) If information on limitations of use or uncertainty about anticipated clinical benefits is relevant to the recommended intervals between doses, to the appropriate duration of treatment when such treatment should be limited, or to any modification of dosage, a concise description of the information with reference to the more detailed information in the "Dosage and Administration" section.

(E) If safety considerations are such that the drug should be reserved for specific situations (e.g., cases refractory to other drugs), a statement of the information.

(F) If there are specific conditions that should be met before the drug is used on a long term basis (e.g., demonstration of responsiveness to the drug in a short term trial in a given patient), a statement of the conditions; or, if the indications for long term use are different from those for short term use, a statement of the specific indications for each use.

(ii) If there is a common belief that the drug may be effective for a certain use or if there is a common use of the drug for a condition, but the preponderance of evidence related to the use or condition shows that the drug is ineffective or that the therapeutic benefits of the product do not generally outweigh its risks, FDA may require that this section state that there is a lack of evidence that the drug is effective or safe for that use or condition.

(iii) Any statements comparing the safety or effectiveness of the drug with other agents for the same indication must, except for biological products, be supported by substantial evidence derived from adequate and well-controlled studies as defined in § 314.126(b) of this chapter unless this requirement is waived under § 201.58 or § 314.126(c) of this chapter. For biological products, such statements must be supported by substantial evidence.

(iv) For drug products other than biological products, all indications listed in this section must be supported by substantial evidence of effectiveness based on adequate and well-controlled studies as defined in § 314.126(b) of this chapter unless the requirement is waived under § 201.58 or § 314.126(c) of this chapter. Indications or uses must not be implied or suggested in other sections of the labeling if not included in this section.

(v) For biological products, all indications listed in this section must be supported by substantial evidence of effectiveness. Indications or uses must not be implied or suggested in other sections of the labeling if not included in this section.

(3) *2 Dosage and administration.*

(i) This section must state the recommended dose and, as appropriate:

(A) The dosage range,

(B) An upper limit beyond which safety and effectiveness have not been established, or beyond which increasing the dose does not result in increasing effectiveness,

(C) Dosages for each indication and subpopulation,

(D) The intervals recommended between doses,

(E) The optimal method of titrating dosage,

(F) The usual duration of treatment when treatment duration should be limited,

(G) Dosing recommendations based on clinical pharmacologic data (e.g., clinically significant food effects),

(H) Modification of dosage needed because of drug interactions or in special patient populations (e.g., in children, in geriatric age groups, in groups defined by genetic characteristics, or in patients with renal or hepatic disease),

(I) Important considerations concerning compliance with the dosage regimen,

(J) Efficacious or toxic concentration ranges and therapeutic concentration windows of the drug or its metabolites, if established and clinically significant. Information on therapeutic drug concentration monitoring (TDM) must also be included in this section when TDM is necessary.

(ii) Dosing regimens must not be implied or suggested in other sections of the labeling if not included in this section.

(iii) Radiation dosimetry information must be stated for both the patient receiving a radioactive drug and the person administering it.

(iv) This section must also contain specific direction on dilution, preparation (including the strength of the final dosage solution, when prepared according to instructions, in terms of milligrams of active ingredient per milliliter of reconstituted solution, unless another measure of the strength is more appropriate), and administration of the dosage form, if needed (e.g., the rate of administration of parenteral drug in milligrams per minute; storage conditions for stability of the reconstituted drug, when important; essential information on drug incompatibilities if the drug is mixed in vitro with other drugs or diluents; and the following verbatim statement for parenterals: "Parenteral drug products should be inspected visually for particulate matter and discoloration prior to administration, whenever solution and container permit.")

(4) ***3 Dosage forms and strengths.*** This section must contain information on the available dosage forms to which the labeling applies and for which the manufacturer or distributor is responsible, including:

(i) The strength or potency of the dosage form in metric system (e.g., 10 milligram tablets), and, if the apothecary system is used, a statement of the strength in parentheses after the metric designation; and

(ii) A description of the identifying characteristics of the dosage forms, including shape, color, coating, scoring, and imprinting, when applicable. The National Drug Code number(s) for the drug product must not be included in this section.

(5) ***4 Contraindications.*** This section must describe any situations in which the drug should not be used because the risk of use (e.g., certain potentially fatal adverse reactions) clearly outweighs any possible therapeutic benefit. Those situations include use of the drug in patients who, because of their particular age, sex, concomitant therapy, disease state, or other condition, have a substantial risk of being harmed by the drug and for whom no potential benefit makes the risk acceptable.

Known hazards and not theoretical possibilities must be listed (e.g., if severe hypersensitivity to the drug has not been demonstrated, it should not be listed as a contraindication). If no contraindications are known, this section must state “None.”

(6) *5 Warnings and precautions.*

(i) *General.* This section must describe clinically significant adverse reactions (including any that are potentially fatal, are serious even if infrequent, or can be prevented or mitigated through appropriate use of the drug), other potential safety hazards (including those that are expected for the pharmacological class or those resulting from drug/drug interactions), limitations in use imposed by them (e.g., avoiding certain concomitant therapy), and steps that should be taken if they occur (e.g., dosage modification). The frequency of all clinically significant adverse reactions and the approximate mortality and morbidity rates for patients experiencing the reaction, if known and necessary for the safe and effective use of the drug, must be expressed as provided under paragraph (c)(7) of this section. In accordance with §§ 314.70 and 601.12 of this chapter, the labeling must be revised to include a warning about a clinically significant hazard as soon as there is reasonable evidence of a causal association with a drug; a causal relationship need not have been definitely established. A specific warning relating to a use not provided for under the “Indications and Usage” section may be required by FDA in accordance with sections 201(n) and 502(a) of the act if the drug is commonly prescribed for a disease or condition and such usage is associated with a clinically significant risk or hazard.

(ii) *Other special care precautions.* This section must contain information regarding any special care to be exercised by the practitioner for safe and effective use of the drug (e.g., precautions not required under any other specific section or subsection).

(iii) *Monitoring: Laboratory tests.* This section must identify any laboratory tests helpful in following the patient's response or in identifying possible adverse reactions. If appropriate, information must be provided on such factors as the range of normal and abnormal values expected in the particular situation and the recommended frequency with which tests should be performed before, during, and after therapy.

(iv) *Interference with laboratory tests.* This section must briefly note information on any known interference by the product with laboratory tests and reference the section where the detailed information is presented (e.g., “Drug Interactions” section).

(7) *6 Adverse reactions.* This section must describe the overall adverse reaction profile of the drug based on the entire safety database. For purposes of prescription drug labeling, an adverse reaction is an undesirable effect, reasonably associated with use of a drug, that may occur as part of the pharmacological action of the drug or may be unpredictable in its occurrence. This definition does not include all adverse events observed during use of a drug, only those adverse events for which there is some basis to believe there is a causal relationship between the drug and the occurrence of the adverse event.

(i) *Listing of adverse reactions.* This section must list the adverse reactions that occur with the drug and with drugs in the same pharmacologically active and chemically related class, if applicable. The list or lists must be preceded by the information necessary to interpret the adverse reactions (e.g., for clinical trials, total number exposed, extent and nature of exposure).

(ii) *Categorization of adverse reactions.* Within a listing, adverse reactions must be categorized by body system, by severity of the reaction, or in order of decreasing frequency, or by a combination of these, as appropriate. Within a category, adverse reactions must be listed in decreasing order of frequency. If frequency information cannot be reliably determined, adverse reactions must be listed in decreasing order of severity.

(A) *Clinical trials experience.* This section must list the adverse reactions identified in clinical trials that occurred at or above a specified rate appropriate to the safety database. The rate of occurrence of an adverse reaction for the drug and comparators (e.g., placebo) must be presented, unless such data cannot be determined or presentation of comparator rates would be misleading. If adverse reactions that occurred below the specified rate are included, they must be included in a separate listing. If comparative rates of occurrence cannot be reliably determined (e.g., adverse reactions were observed only in the uncontrolled trial portion of the overall safety database), adverse reactions must be grouped within specified frequency ranges as appropriate to the safety database for the drug (e.g., adverse reactions occurring at a rate of less than 1/100, adverse reactions occurring at a rate of less than 1/500) or descriptively identified, if frequency ranges cannot be determined. For adverse reactions with significant clinical implications, the listings must be supplemented with additional detail about the nature, frequency, and severity of the adverse reaction and the relationship of the adverse reaction to drug dose and demographic characteristics, if data are available and important.

(B) *Postmarketing experience.* This section of the labeling must list the adverse reactions, as defined in paragraph (c)(7) of this section, that are identified from domestic and foreign spontaneous reports. This listing must be separate from the listing of adverse reactions identified in clinical trials.

(iii) *Comparisons of adverse reactions between drugs.* For drug products other than biological products, any claim comparing the drug to which the labeling applies with other drugs in terms of frequency, severity, or character of adverse reactions must be based on adequate and well-controlled studies as defined in § 314.126(b) of this chapter unless this requirement is waived under § 201.58 or § 314.126(c) of this chapter. For biological products, any such claim must be based on substantial evidence.

(8) *7 Drug interactions.*

(i) This section must contain a description of clinically significant interactions, either observed or predicted, with other prescription or over-the-counter drugs, classes of drugs, or foods (e.g., dietary supplements, grapefruit juice), and specific practical instructions for preventing or managing them. The mechanism(s) of the interaction, if known, must be briefly described. Interactions that are described in the “Contraindications” or “Warnings and Precautions” sections must be discussed in more detail under this section. Details of drug interaction pharmacokinetic studies that are included in the “Clinical Pharmacology” section that are pertinent to clinical use of the drug must not be repeated in this section.

(ii) This section must also contain practical guidance on known interference of the drug with laboratory tests.

(9) *8 Use in specific populations.* This section must contain the following subsections:

(i) ***8.1 Pregnancy.*** This subsection of the labeling must contain the following information in the following order under the subheadings "Pregnancy Exposure Registry," "Risk Summary," "Clinical Considerations," and "Data":

(A) ***Pregnancy exposure registry.*** If there is a scientifically acceptable pregnancy exposure registry for the drug, contact information needed to enroll in the registry or to obtain information about the registry must be provided following the statement: "There is a pregnancy exposure registry that monitors pregnancy outcomes in women exposed to (*name of drug*) during pregnancy."

(B) ***Risk summary.*** The Risk Summary must contain risk statement(s) based on data from all relevant sources (human, animal, and/or pharmacologic) that describe, for the drug, the risk of adverse developmental outcomes (*i.e.,* structural abnormalities, embryo-fetal and/or infant mortality, functional impairment, alterations to growth). When multiple data sources are available, the statements must be presented in the following order: Human, animal, pharmacologic. The source(s) of the data must be stated. The labeling must state the percentage range of live births in the United States with a major birth defect and the percentage range of pregnancies in the United States that end in miscarriage, regardless of drug exposure. If such information is available for the population(s) for which the drug is labeled, it must also be included. When use of a drug is contraindicated during pregnancy, this information must be stated first in the Risk Summary. When applicable, risk statements as described in paragraphs (c)(9)(i)(B)(*1*) and (*2*) of this section must include a cross-reference to additional details in the relevant portion of the "Data" subheading in the "Pregnancy" subsection of the labeling. If data demonstrate that a drug is not systemically absorbed following a particular route of administration, the Risk Summary must contain only the following statement: "(*Name of drug*) is not absorbed systemically following (route of administration), and maternal use is not expected to result in fetal exposure to the drug."

(*1*) ***Risk statement based on human data.*** When human data are available that establish the presence or absence of any adverse developmental outcome(s) associated with maternal use of the drug, the Risk Summary must summarize the specific developmental outcome(s); their incidence; and the effects of dose, duration of exposure, and gestational timing of exposure. If human data indicate that there is an increased risk for a specific adverse developmental outcome in infants born to women exposed to the drug during pregnancy, this risk must be quantitatively compared to the risk for the same outcome in infants born to women who were not exposed to the drug but who have the disease or condition for which the drug is indicated to be used. When risk information is not available for women with the disease or condition for which the drug is indicated, the risk for the specific outcome must be compared to the rate at which the outcome occurs in the general population. The Risk Summary must state when there are no human data or when available human data do not establish the presence or absence of drug-associated risk.

(*2*) ***Risk statement based on animal data.*** When animal data are available, the Risk Summary must summarize the findings in animals and based on these findings, describe, for the drug, the potential risk of any adverse developmental outcome(s) in humans. This statement must include: The number and type(s) of species affected, timing of exposure, animal doses expressed in terms of human dose or exposure equivalents, and outcomes for pregnant animals and offspring. When animal studies

do not meet current standards for nonclinical developmental toxicity studies, the Risk Summary must so state. When there are no animal data, the Risk Summary must so state.

(*3*) ***Risk statement based on pharmacology.*** When the drug has a well-understood mechanism of action that may result in adverse developmental outcome(s), the Risk Summary must explain the mechanism of action and the potential associated risks.

(C) ***Clinical considerations.*** Under the subheading "Clinical Considerations," the labeling must provide relevant information, to the extent it is available, under the headings "Disease-associated maternal and/or embryo/fetal risk," "Dose adjustments during pregnancy and the postpartum period," "Maternal adverse reactions," "Fetal/Neonatal adverse reactions," and "Labor or delivery":

(*1*) ***Disease-associated maternal and/or embryo/fetal risk.*** If there is a serious known or potential risk to the pregnant woman and/or the embryo/fetus associated with the disease or condition for which the drug is indicated to be used, the labeling must describe the risk.

(*2*) ***Dose adjustments during pregnancy and the postpartum period.*** If there are pharmacokinetic data that support dose adjustment(s) during pregnancy and the postpartum period, a summary of this information must be provided.

(*3*) ***Maternal adverse reactions.*** If use of the drug is associated with a maternal adverse reaction that is unique to pregnancy or if a known adverse reaction occurs with increased frequency or severity in pregnant women, the labeling must describe the adverse reaction and available intervention(s) for monitoring or mitigating the reaction. The labeling must describe, if known, the effect of dose, timing, and duration of exposure on the risk to the pregnant woman of experiencing the adverse reaction.

(*4*) ***Fetal/Neonatal adverse reactions.*** If it is known or anticipated that treatment of the pregnant woman increases or may increase the risk of an adverse reaction in the fetus or neonate, the labeling must describe the adverse reaction, the potential severity and reversibility of the adverse reaction, and available intervention(s) for monitoring or mitigating the reaction. The labeling must describe, if known, the effect of dose, timing, and duration of exposure on the risk.

(*5*) ***Labor or delivery.*** If the drug is expected to affect labor or delivery, the labeling must provide information about the effect of the drug on the pregnant woman and the fetus or neonate; the effect of the drug on the duration of labor and delivery; any increased risk of adverse reactions, including their potential severity and reversibility; and must provide information about available intervention(s) that can mitigate these effects and/or adverse reactions. The information described under this heading is not required for drugs approved for use only during labor and delivery.

(D) ***Data —***

(*1*) ***"Data" subheading.*** Under the subheading "Data," the labeling must describe the data that are the basis for the Risk Summary and Clinical Considerations.

(*2*) ***Human and animal data headings.*** Human and animal data must be presented separately, beneath the headings “Human Data” and “Animal Data,” and human data must be presented first.

(*3*) ***Description of human data.*** For human data, the labeling must describe adverse developmental outcomes, adverse reactions, and other adverse effects. To the extent applicable, the labeling must describe the types of studies or reports, number of subjects and the duration of each study, exposure information, and limitations of the data. Both positive and negative study findings must be included.

(*4*) ***Description of animal data.*** For animal data, the labeling must describe the following: Types of studies, animal species, dose, duration and timing of exposure, study findings, presence or absence of maternal toxicity, and limitations of the data. Description of maternal and offspring findings must include dose-response and severity of adverse developmental outcomes. Animal doses or exposures must be described in terms of human dose or exposure equivalents and the basis for those calculations must be included.

(ii) ***8.2 Lactation.*** This subsection of the labeling must contain the following information in the following order under the subheadings “Risk Summary,” “Clinical Considerations,” and “Data”:

(A) ***Risk summary.*** When relevant human and/or animal lactation data are available, the Risk Summary must include a cross-reference to the “Data” subheading in the “Lactation” subsection of the labeling. When human data are available, animal data must not be included unless the animal model is specifically known to be predictive for humans. When use of a drug is contraindicated during breastfeeding, this information must be stated first in the Risk Summary.

(*1*) ***Drug not absorbed systemically.*** If data demonstrate that the drug is not systemically absorbed by the mother, the Risk Summary must contain only the following statement: “(*Name of drug*) is not absorbed systemically by the mother following (route of administration), and breastfeeding is not expected to result in exposure of the child to (*name of drug*).”

(*2*) ***Drug absorbed systemically.*** If the drug is absorbed systemically, the Risk Summary must describe the following to the extent relevant information is available:

(*i*) ***Presence of drug in human milk.*** The Risk Summary must state whether the drug and/or its active metabolite(s) are present in human milk. If there are no data to assess this, the Risk Summary must so state. If studies demonstrate that the drug and/or its active metabolite(s) *are* not detectable in human milk, the Risk Summary must state the limits of the assay used. If studies demonstrate the presence of the drug and/or its active metabolite(s) in human milk, the Risk Summary must state the concentration of the drug and/or its active metabolite(s) in human milk and the actual or estimated daily dose for an infant fed exclusively with human milk. The actual or estimated amount of the drug and/or its active metabolite(s) ingested by the infant must be compared to the labeled infant or pediatric dose, if available, or to the maternal dose. If studies demonstrate the presence of the drug and/or its active metabolite(s) in human milk but the drug and/or its active metabolite(s) are not expected to be systemically bioavailable to the breast-fed child, the Risk Summary must

describe the disposition of the drug and/or its active metabolite(s). If only animal lactation data are available, the Risk Summary must state only whether or not the drug and/or its active metabolite(s) were detected in animal milk and specify the animal species.

(*ii*) ***Effects of drug on the breast-fed child.*** The Risk Summary must include information, on the known or predicted effects on the child from exposure to the drug and/or its active metabolite(s) through human milk or from contact with breast or nipple skin (for topical products). The Risk Summary also must include information on systemic and/or local adverse reactions. If there are no data to assess the effects of the drug and/or its active metabolite(s) on the breast-fed child, the Risk Summary must so state.

(*iii*) ***Effects of drug on milk production.*** The Risk Summary must describe the effects of the drug and/or its active metabolite(s) on milk production. If there are no data to assess the effects of the drug and/or its active metabolite(s) on milk production, the Risk Summary must so state.

(*3*) ***Risk and benefit statement.*** For drugs absorbed systemically, unless breastfeeding is contraindicated during drug therapy, the following risk and benefit statement must appear at the end of the Risk Summary: “The developmental and health benefits of breastfeeding should be considered along with the mother's clinical need for (*name of drug*) and any potential adverse effects on the breast-fed child from (*name of drug*) or from the underlying maternal condition.”

(B) ***Clinical considerations.*** Under “Clinical Considerations,” the following information must be provided to the extent it is available and relevant:

(*1*) ***Minimizing exposure.*** The labeling must describe ways to minimize exposure in the breast-fed child if: The drug and/or its active metabolite(s) are present in human milk in clinically relevant concentrations; the drug does not have an established safety profile in infants; and the drug is used either intermittently, in single doses, or for short courses of therapy. When applicable, the labeling must also describe ways to minimize a breast-fed child's oral intake of topical drugs applied to the breast or nipple skin.

(*2*) ***Monitoring for adverse reactions.*** The labeling must describe available intervention(s) for monitoring or mitigating the adverse reaction(s) presented in the Risk Summary.

(C) ***Data.*** Under the subheading “Data,” the labeling must describe the data that are the basis for the Risk Summary and Clinical Considerations.

(iii) ***8.3 Females and males of reproductive potential.*** When pregnancy testing and/or contraception are required or recommended before, during, or after drug therapy and/or when there are human and/or animal data that suggest drug-associated fertility effects, this subsection of labeling must contain this information under the subheadings “Pregnancy Testing,” “Contraception,” and “Infertility,” in that order.

(iv) ***8.4 Pediatric use.***

(A) Pediatric population(s)/pediatric patient(s): For the purposes of paragraphs (c)(9)(iv)(B) through (c)(9)(iv)(H) of this section, the terms *pediatric population(s)* and *pediatric patient(s)* are defined as the pediatric age group, from birth to 16 years, including age groups often called neonates, infants, children, and adolescents.

(B) If there is a specific pediatric indication different from those approved for adults that is supported by adequate and well-controlled studies in the pediatric population, it must be described under the "Indications and Usage" section, and appropriate pediatric dosage information must be given under the "Dosage and Administration" section. The "Pediatric use" subsection must cite any limitations on the pediatric indication, need for specific monitoring, specific hazards associated with use of the drug in any subsets of the pediatric population (e.g., neonates), differences between pediatric and adult responses to the drug, and other information related to the safe and effective pediatric use of the drug. Data summarized in this subsection should be discussed in more detail, if appropriate, under the "Clinical Pharmacology" or "Clinical Studies" section. As appropriate, this information must also be contained in the "Contraindications" and/or "Warnings and Precautions" section(s).

(C) If there are specific statements on pediatric use of the drug for an indication also approved for adults that are based on adequate and well-controlled studies in the pediatric population, they must be summarized in the "Pediatric use" subsection and discussed in more detail, if appropriate, under the "Clinical Pharmacology" and "Clinical Studies" sections. Appropriate pediatric dosage must be given under the "Dosage and Administration" section. The "Pediatric use" subsection of the labeling must also cite any limitations on the pediatric use statement, need for specific monitoring, specific hazards associated with use of the drug in any subsets of the pediatric population (e.g., neonates), differences between pediatric and adult responses to the drug, and other information related to the safe and effective pediatric use of the drug. As appropriate, this information must also be contained in the "Contraindications" and/or "Warnings and Precautions" section(s).

(D)

(*1*) When a drug is approved for pediatric use based on adequate and well-controlled studies in adults with other information supporting pediatric use, the "Pediatric use" subsection of the labeling must contain either the following statement or a reasonable alternative:

> The safety and effectiveness of (*drug name*) have been established in the age groups ___ to ___ (note any limitations, e.g., no data for pediatric patients under 2, or only applicable to certain indications approved in adults). Use of (*drug name*) in these age groups is supported by evidence from adequate and well-controlled studies of (*drug name*) in adults with additional data (*insert wording that accurately describes the data submitted to support a finding of substantial evidence of effectiveness in the pediatric population*).

(*2*) Data summarized in the preceding prescribed statement in this subsection must be discussed in more detail, if appropriate, under the "Clinical Pharmacology" or the "Clinical Studies" section. For example, pediatric pharmacokinetic or pharmacodynamic studies and dose response information should be described in the "Clinical Pharmacology" section. Pediatric dosing instructions must be included

in the “Dosage and Administration” section. Any differences between pediatric and adult responses, need for specific monitoring, dosing adjustments, and any other information related to safe and effective use of the drug in pediatric patients must be cited briefly in the “Pediatric use” subsection and, as appropriate, in the “Contraindications,” “Warnings and Precautions,” and “Dosage and Administration” sections.

(E) If the requirements for a finding of substantial evidence to support a pediatric indication or a pediatric use statement have not been met for a particular pediatric population, the “Pediatric use” subsection must contain an appropriate statement such as “Safety and effectiveness in pediatric patients below the age of (__) have not been established.” If use of the drug in this pediatric population is associated with a specific hazard, the hazard must be described in this subsection, or, if appropriate, the hazard must be stated in the “Contraindications” or “Warnings and Precautions” section and this subsection must refer to it.

(F) If the requirements for a finding of substantial evidence to support a pediatric indication or a pediatric use statement have not been met for any pediatric population, this subsection must contain the following statement: “Safety and effectiveness in pediatric patients have not been established.” If use of the drug in premature or neonatal infants, or other pediatric subgroups, is associated with a specific hazard, the hazard must be described in this subsection, or, if appropriate, the hazard must be stated in the “Contraindications” or “Warnings and Precautions” section and this subsection must refer to it.

(G) If the sponsor believes that none of the statements described in paragraphs (c)(9)(iv)(B) through (c)(9)(iv)(F) of this section are appropriate or relevant to the labeling of a particular drug, the sponsor must provide reasons for omission of the statements and may propose alternative statement(s). FDA may permit use of an alternative statement if FDA determines that no statement described in those paragraphs is appropriate or relevant to the drug's labeling and that the alternative statement is accurate and appropriate.

(H) If the drug product contains one or more inactive ingredients that present an increased risk of toxic effects to neonates or other pediatric subgroups, a special note of this risk must be made, generally in the “Contraindications” or “Warnings and Precautions” section.

(v) *8.5 Geriatric use.*

(A) A specific geriatric indication, if any, that is supported by adequate and well-controlled studies in the geriatric population must be described under the “Indications and Usage” section, and appropriate geriatric dosage must be stated under the “Dosage and Administration” section. The “Geriatric use” subsection must cite any limitations on the geriatric indication, need for specific monitoring, specific hazards associated with the geriatric indication, and other information related to the safe and effective use of the drug in the geriatric population. Unless otherwise noted, information contained in the “Geriatric use” subsection must pertain to use of the drug in persons 65 years of age and older. Data summarized in this subsection must be discussed in more detail, if appropriate, under “Clinical Pharmacology” or the “Clinical Studies” section. As appropriate, this information must also be contained in the “Warnings and Precautions” and/or “Contraindications” section(s).

(B) Specific statements on geriatric use of the drug for an indication approved for adults generally, as distinguished from a specific geriatric indication, must be contained in the "Geriatric use" subsection and must reflect all information available to the sponsor that is relevant to the appropriate use of the drug in elderly patients. This information includes detailed results from controlled studies that are available to the sponsor and pertinent information from well-documented studies obtained from a literature search. Controlled studies include those that are part of the marketing application and other relevant studies available to the sponsor that have not been previously submitted in the investigational new drug application, new drug application, biologics license application, or a supplement or amendment to one of these applications (e.g., postmarketing studies or adverse drug reaction reports). The "Geriatric use" subsection must contain the following statement(s) or reasonable alternative, as applicable, taking into account available information:

(*1*) If clinical studies did not include sufficient numbers of subjects aged 65 and over to determine whether elderly subjects respond differently from younger subjects, and other reported clinical experience has not identified such differences, the "Geriatric use" subsection must include the following statement:

Clinical studies of (*name of drug*) did not include sufficient numbers of subjects aged 65 and over to determine whether they respond differently from younger subjects. Other reported clinical experience has not identified differences in responses between the elderly and younger patients. In general, dose selection for an elderly patient should be cautious, usually starting at the low end of the dosing range, reflecting the greater frequency of decreased hepatic, renal, or cardiac function, and of concomitant disease or other drug therapy.

(*2*) If clinical studies (including studies that are part of marketing applications and other relevant studies available to the sponsor that have not been submitted in the sponsor's applications) included enough elderly subjects to make it likely that differences in safety or effectiveness between elderly and younger subjects would have been detected, but no such differences (in safety or effectiveness) were observed, and other reported clinical experience has not identified such differences, the "Geriatric use" subsection must contain the following statement:

> Of the total number of subjects in clinical studies of (*name of drug*), __ percent were 65 and over, while __ percent were 75 and over. (Alternatively, the labeling may state the total number of subjects included in the studies who were 65 and over and 75 and over.) No overall differences in safety or effectiveness were observed between these subjects and younger subjects, and other reported clinical experience has not identified differences in responses between the elderly and younger patients, but greater sensitivity of some older individuals cannot be ruled out.

(*3*) If evidence from clinical studies and other reported clinical experience available to the sponsor indicates that use of the drug in elderly patients is associated with differences in safety or effectiveness, or requires specific monitoring or dosage adjustment, the "Geriatric use" subsection must contain a brief description of observed differences or specific monitoring or dosage requirements and, as appropriate, must refer to more detailed discussions in the "Contraindications," "Warnings and Precautions," "Dosage and Administration," or other sections.

(C)

(*1*) If specific pharmacokinetic or pharmacodynamic studies have been carried out in the elderly, they must be described briefly in the "Geriatric use" subsection and in detail under the "Clinical Pharmacology" section. The "Clinical Pharmacology" and "Drug Interactions" sections ordinarily contain information on drug/disease and drug/drug interactions that is particularly relevant to the elderly, who are more likely to have concomitant illness and to use concomitant drugs.

(*2*) If a drug is known to be substantially excreted by the kidney, the "Geriatric use" subsection must include the statement:

> This drug is known to be substantially excreted by the kidney, and the risk of adverse reactions to this drug may be greater in patients with impaired renal function. Because elderly patients are more likely to have decreased renal function, care should be taken in dose selection, and it may be useful to monitor renal function.

(D) If use of the drug in the elderly appears to cause a specific hazard, the hazard must be described in the "Geriatric use" subsection, or, if appropriate, the hazard must be stated in the "Contraindications" or "Warnings and Precautions" section, and the "Geriatric use" subsection must refer to those sections.

(E) Labeling under paragraphs (c)(9)(v)(A) through (c)(9)(v)(C) of this section may include statements, if they are necessary for safe and effective use of the drug, and reflect good clinical practice or past experience in a particular situation, e.g., for a sedating drug, it could be stated that:

> Sedating drugs may cause confusion and over-sedation in the elderly; elderly patients generally should be started on low doses of (*name of drug*) and observed closely.

(F) If the sponsor believes that none of the requirements described in paragraphs (c)(9)(v)(A) through (c)(9)(v)(E) of this section are appropriate or relevant to the labeling of a particular drug, the sponsor must provide reasons for omission of the statements and may propose an alternative statement. FDA may permit omission of the statements if FDA determines that no statement described in those paragraphs is appropriate or relevant to the drug's labeling. FDA may permit use of an alternative statement if the agency determines that such statement is accurate and appropriate.

(vi) ***Additional subsections.*** Additional subsections may be included, as appropriate, if sufficient data are available concerning the use of the drug in other specified subpopulations (e.g., renal or hepatic impairment).

(10) ***9 Drug abuse and dependence.*** This section must contain the following information, as appropriate:

(i) ***9.1 Controlled substance.*** If the drug is controlled by the Drug Enforcement Administration, the schedule in which it is controlled must be stated.

(ii) ***9.2 Abuse.*** This subsection must state the types of abuse that can occur with the drug and the adverse reactions pertinent to them, and must identify particularly susceptible patient populations. This subsection must be based primarily on human data and human experience, but pertinent animal data may also be used.

(iii) *9.3 Dependence.* This subsection must describe characteristic effects resulting from both psychological and physical dependence that occur with the drug and must identify the quantity of the drug over a period of time that may lead to tolerance or dependence, or both. Details must be provided on the adverse effects of chronic abuse and the effects of abrupt withdrawal. Procedures necessary to diagnose the dependent state and the principles of treating the effects of abrupt withdrawal must be described.

(11) *10 Overdosage.* This section must be based on human data. If human data are unavailable, appropriate animal and in vitro data may be used. The following specific information must be provided:

(i) Signs, symptoms, and laboratory findings associated with an overdosage of the drug;

(ii) Complications that can occur with the drug (for example, organ toxicity or delayed acidosis);

(iii) Concentrations of the drug in biologic fluids associated with toxicity or death; physiologic variables influencing excretion of the drug, such as urine pH; and factors that influence the dose response relationship of the drug, such as tolerance. The pharmacokinetic data given in the "Clinical Pharmacology" section also may be referenced here, if applicable to overdoses;

(iv) The amount of the drug in a single dose that is ordinarily associated with symptoms of overdosage and the amount of the drug in a single dose that is likely to be life threatening;

(v) Whether the drug is dialyzable; and

(vi) Recommended general treatment procedures and specific measures for support of vital functions (e.g., proven antidotes, gastric lavage, forced diuresis, or as per Poison Control Center). Such recommendations must be based on data available for the specific drug or experience with pharmacologically related drugs. Unqualified recommendations for which data are lacking for the specific drug or class of drugs must not be stated.

(12) *11 Description.*

(i) This section must contain:

(A) The proprietary name and the established name, if any, as defined in section 502(e)(2) of the act, of the drug or, for biological products, the proper name (as defined in § 600.3 of this chapter) and any appropriate descriptors;

(B) The type of dosage form(s) and the route(s) of administration to which the labeling applies;

(C) The same qualitative and/or quantitative ingredient information as required under § 201.100(b) for drug labels or §§ 610.60 and 610.61 of this chapter for biological product labels;

(D) If the product is sterile, a statement of that fact;

(E) The pharmacological or therapeutic class of the drug;

(F) For drug products other than biological products, the chemical name and structural formula of the drug; and

(G) If the product is radioactive, a statement of the important nuclear physical characteristics, such as the principal radiation emission data, external radiation, and physical decay characteristics.

(ii) If appropriate, other important chemical or physical information, such as physical constants or pH, must be stated.

(13) *12 Clinical pharmacology.*

(i) This section must contain information relating to the human clinical pharmacology and actions of the drug in humans. Pharmacologic information based on in vitro data using human biomaterials or pharmacologic animal models, or relevant details about in vivo study designs or results (e.g., drug interaction studies), may be included in this section if essential to understand dosing or drug interaction information presented in other sections of the labeling. This section must include the following subsections:

(A) *12.1 Mechanism of action.* This subsection must summarize what is known about the established mechanism(s) of the drug's action in humans at various levels (e.g., receptor, membrane, tissue, organ, whole body). If the mechanism of action is not known, this subsection must contain a statement about the lack of information.

(B) *12.2 Pharmacodynamics.* This subsection must include a description of any biochemical or physiologic pharmacologic effects of the drug or active metabolites related to the drug's clinical effect in preventing, diagnosing, mitigating, curing, or treating disease, or those related to adverse effects or toxicity. Exposure-response relationships (e.g., concentration-response, dose-response) and time course of pharmacodynamic response (including short-term clinical response) must be included if known. If this information is unknown, this subsection must contain a statement about the lack of information. Detailed dosing or monitoring recommendations based on pharmacodynamic information that appear in other sections (e.g., “Warnings and Precautions” or “Dosage and Administration”) must not be repeated in this subsection, but the location of such recommendations must be referenced.

(C) *12.3 Pharmacokinetics.* This subsection must describe the clinically significant pharmacokinetics of a drug or active metabolites, (i.e., pertinent absorption, distribution, metabolism, and excretion parameters). Information regarding bioavailability, the effect of food, minimum concentration (C_{min}), maximum concentration (C_{max}), time to maximum concentration (T_{max}), area under the curve (AUC), pertinent half-lives ($t_{1/2}$), time to reach steady state, extent of accumulation, route(s) of elimination, clearance (renal, hepatic, total), mechanisms of clearance (e.g., specific enzyme systems), drug/drug and drug/food (e.g., dietary supplements, grapefruit juice) pharmacokinetic interactions (including inhibition, induction, and genetic characteristics), and volume of distribution (V_d) must be presented if clinically significant. Information regarding nonlinearity in pharmacokinetic parameters, changes in pharmacokinetics over time, and binding (plasma protein, erythrocyte) parameters must also be presented if clinically significant. This section must also include the results of pharmacokinetic studies (e.g., of metabolism or interaction) that establish the absence of an effect, including pertinent human studies and in vitro data. Dosing recommendations based on clinically significant factors that change the product's pharmacokinetics (e.g., age, gender, race, hepatic or renal dysfunction,

concomitant therapy) that appear in other sections (e.g., "Warnings and Precautions," "Dosage and Administration" or "Use in Specific Populations") must not be repeated in this subsection, but the location of such recommendations must be referenced.

(ii) Data that demonstrate activity or effectiveness in in vitro or animal tests and that have not been shown by adequate and well-controlled clinical studies to be pertinent to clinical use may be included under this section only under the following circumstances:

(A) In vitro data for anti-infective drugs may be included if the data are immediately preceded by the statement "The following in vitro data are available but their clinical significance is unknown."

(B) For other classes of drugs, in vitro and animal data that have not been shown by adequate and well-controlled studies, as defined in § 314.126(b) of this chapter, to be necessary for the safe and effective use may be included in this section only if a waiver is granted under § 201.58 or § 314.126(c) of this chapter.

(14) *13 Nonclinical toxicology.* This section must contain the following subsections as appropriate:

(i) *13.1 Carcinogenesis, mutagenesis, impairment of fertility.* This subsection must state whether long term studies in animals have been performed to evaluate carcinogenic potential and, if so, the species and results. If results from reproduction studies or other data in animals raise concern about mutagenesis or impairment of fertility in either males or females, this must be described. Any precautionary statement on these topics must include practical, relevant advice to the prescriber on the significance of these animal findings. Human data suggesting that the drug may be carcinogenic or mutagenic, or suggesting that it impairs fertility, as described in the "Warnings and Precautions" section, must not be included in this subsection of the labeling.

(ii) *13.2 Animal toxicology and/or pharmacology.* Significant animal data necessary for safe and effective use of the drug in humans that is not incorporated in other sections of labeling must be included in this section (e.g., specifics about studies used to support approval under § 314.600 or § 601.90 of this chapter, the absence of chronic animal toxicity data for a drug that is administered over prolonged periods or is implanted in the body).

(15) *14 Clinical studies.* This section must discuss those clinical studies that facilitate an understanding of how to use the drug safely and effectively. Ordinarily, this section will describe the studies that support effectiveness for the labeled indication(s), including discussion of study design, population, endpoints, and results, but must not include an encyclopedic listing of all, or even most, studies performed as part of the product's clinical development program. If a specific important clinical study is mentioned in any section of the labeling required under §§ 201.56 and 201.57 because the study is essential to an understandable presentation of the information in that section of the labeling, any detailed discussion of the study must appear in this section.

(i) For drug products other than biological products, any clinical study that is discussed in prescription drug labeling that relates to an indication for or use of the drug must be adequate and well-controlled as described in § 314.126(b) of this chapter and must not imply or suggest indications or uses or dosing regimens not stated in the "Indications and Usage" or "Dosage and Administration" section. For biological products, any clinical study that is discussed that relates to an indication for or use of the biological product must constitute or contribute to substantial evidence and must not imply or suggest indications or uses or dosing regimens not stated in the "Indications and Usage" or "Dosage and Administration" section.

(ii) Any discussion of a clinical study that relates to a risk from the use of the drug must also refer to the other sections of the labeling where the risk is identified or discussed.

(16) *15 References.* When prescription drug labeling must summarize or otherwise rely on a recommendation by an authoritative scientific body, or on a standardized methodology, scale, or technique, because the information is important to prescribing decisions, the labeling may include a reference to the source of the information.

(17) *16 How supplied/storage and handling.* This section must contain information on the available dosage forms to which the labeling applies and for which the manufacturer or distributor is responsible. The information must include, as appropriate:

(i) The strength or potency of the dosage form in metric system (e.g., 10 milligram tablets) and, if the apothecary system is used, a statement of the strength in parentheses after the metric designation;

(ii) The units in which the dosage form is ordinarily available for prescribing by practitioners (e.g., bottles of 100);

(iii) Appropriate information to facilitate identification of the dosage forms, such as shape, color, coating, scoring, imprinting, and National Drug Code number; and

(iv) Special handling and storage conditions.

(18) *17 Patient counseling information.* This section must contain information necessary for patients to use the drug safely and effectively (e.g., precautions concerning driving or the concomitant use of other substances that may have harmful additive effects). Any FDA-approved patient labeling must be referenced in this section and the full text of such patient labeling must be reprinted immediately following this section or, alternatively, accompany the prescription drug labeling. Any FDA-approved patient labeling printed immediately following this section or accompanying the labeling is subject to the type size requirements in paragraph (d)(6) of this section, except for a Medication Guide to be detached and distributed to patients in compliance with § 208.24 of this chapter. Medication Guides for distribution to patients are subject to the type size requirements set forth in § 208.20 of this chapter.

(d) *Format requirements.* All labeling information required under paragraphs (a), (b), and (c) of this section must be printed in accordance with the following specifications:

(1) All headings and subheadings required by paragraphs (a) and (c) of this section must be highlighted by bold type that prominently distinguishes the headings and subheadings from other labeling information. Reverse type is not permitted as a form of highlighting.

(2) A horizontal line must separate the information required by paragraphs (a), (b), and (c) of this section.

(3) The headings listed in paragraphs (a)(5) through (a)(13) of this section must be presented in the center of a horizontal line.

(4) If there are multiple subheadings listed under paragraphs (a)(4) through (a)(13) of this section, each subheading must be preceded by a bullet point.

(5) The labeling information required by paragraphs (a)(1) through (a)(4), (a)(11)(ii) through (a)(11)(iv), and (a)(14) of this section must be in bold print.

(6) The letter height or type size for all labeling information, headings, and subheadings set forth in paragraphs (a), (b), and (c) of this section must be a minimum of 8 points, except for labeling information that is on or within the package from which the drug is to be dispensed, which must be a minimum of 6 points.

(7) The identifying numbers required by § 201.56(d) and paragraphs (c)(1) through (c)(18) of this section must be presented in bold print and must precede the heading or subheading by at least two square em's (i.e., two squares of the size of the letter “m” in 8 point type).

(8) The information required by paragraph (a) of this section, not including the information required under paragraph (a)(4) of this section, must be limited in length to an amount that, if printed in 2 columns on a standard sized piece of typing paper ($8\frac{1}{2}$ by 11 inches), single spaced, in 8 point type with $\frac{1}{2}$-inch margins on all sides and between columns, would fit on one-half of the page.

(9) Sections or subsections of labeling that are identified as containing recent major changes under paragraph (a)(5) of this section must be highlighted in the full prescribing information by the inclusion of a vertical line on the left edge of the new or modified text.

(10) For the information required by paragraph (b) of this section, each section heading must be in bold print. Each subheading within a section must be indented and not bolded.

[71 FR 3988, Jan. 24, 2006, as amended at 79 FR 72101, Dec. 4, 2014]

INDICATIONS AND USAGE

Indications and Usage Section of Labeling for Human Prescription Drug and Biological Products — Content and Format

Guidance for Industry

DRAFT GUIDANCE

This guidance document is being distributed for comment purposes only.

Comments and suggestions regarding this draft document should be submitted within 60 days of publication in the *Federal Register* of the notice announcing the availability of the draft guidance. Submit electronic comments to https://www.regulations.gov. Submit written comments to the Dockets Management Staff (HFA-305), Food and Drug Administration, 5630 Fishers Lane, Rm. 1061, Rockville, MD 20852. All comments should be identified with the docket number listed in the notice of availability that publishes in the *Federal Register*.

For questions regarding this draft document, contact (CDER) Iris Masucci at 301-796-2500 or (CBER) the Office of Communication, Outreach and Development at 800-835-4709 or 240-402-8010.

U.S. Department of Health and Human Services
Food and Drug Administration
Center for Drug Evaluation and Research (CDER)
Center for Biologics Evaluation and Research (CBER)

July 2018
Labeling

Indications and Usage Section of Labeling for Human Prescription Drug and Biological Products — Content and Format

Guidance for Industry

Additional copies are available from:

Office of Communications, Division of Drug Information
Center for Drug Evaluation and Research
Food and Drug Administration
10001 New Hampshire Ave., Hillandale Bldg., 4th Floor
Silver Spring, MD 20993-0002
Phone: 855-543-3784 or 301-796-3400; Fax: 301-431-6353
Email: druginfo@fda.hhs.gov
http://www.fda.gov/Drugs/GuidanceComplianceRegulatoryInformation/Guidances/default.htm

and/or

Office of Communication, Outreach and Development
Center for Biologics Evaluation and Research
Food and Drug Administration
10903 New Hampshire Ave., Bldg. 71, Room 3128
Silver Spring, MD 20993-0002
Phone: 800-835-4709 or 240-402-8010
Email: ocod@fda.hhs.gov
http://www.fda.gov/BiologicsBloodVaccines/GuidanceComplianceRegulatoryInformation/Guidances/default.htm

U.S. Department of Health and Human Services
Food and Drug Administration
Center for Drug Evaluation and Research (CDER)
Center for Biologics Evaluation and Research (CBER)

July 2018
Labeling

TABLE OF CONTENTS

I. INTRODUCTION... 1

II. GENERAL PRINCIPLES.. 2

A. Scope of the Indication(s) 3

1. Scope of an Indication Relative to the Population Studied.. 3
2. Age Groups in Indications 4

B. Distribution of Information Among Labeling Sections 6

C. Updating the INDICATIONS AND USAGE Section 6

III. CONTENT AND FORMAT OF THE INDICATIONS AND USAGE SECTION..... 6

A. Indication 7

1. The Disease, Condition, or Manifestation Being Treated, Prevented, Mitigated, Cured, or Diagnosed... 8
2. Other Information Necessary To Describe the Approved Indication 8

B. Limitations of Use 10

1. Situations in Which Limitations of Use Would Be Appropriate.. 11
2. Situations in Which Limitations of Use Generally Would ***Not*** *Be Appropriate........................... 13*

C. Other Considerations for Writing the INDICATIONS AND USAGE Section 14

1. Identification of Outcomes, Endpoints, and Benefit(s) the Drug Conveys................................... 14
2. Accelerated Approval.. 15
3. Required or Recommended Language 15
4. Preferred Wording and Wording Generally To Avoid.. 16

D. Formatting the INDICATIONS AND USAGE Section 16

1. Format for Multiple Indications 16
2. Format for Limitations of Use 17

Indications and Usage Section of Labeling for Human Prescription Drug and Biological Products – Content and Format Guidance for Industry[1]

This draft guidance, when finalized, will represent the current thinking of the Food and Drug Administration (FDA or Agency) on this topic. It does not establish any rights for any person and is not binding on FDA or the public. You can use an alternative approach if it satisfies the requirements of the applicable statutes and regulations. To discuss an alternative approach, contact the FDA staff responsible for this guidance as listed on the title page.

I. INTRODUCTION

This guidance is intended to assist applicants in drafting the INDICATIONS AND USAGE section of labeling as described in the regulations for the content and format of labeling for human prescription drug and biological products[2] (21 CFR 201.57(c)(2)).[3]

Recommendations include the following:

- General principles to consider when drafting the INDICATIONS AND USAGE section of the labeling
- What information to include in the INDICATIONS AND USAGE section
- When to include additional descriptors or qualifiers as part of the indication in the INDICATIONS AND USAGE section
- When to include limitations of use in the INDICATIONS AND USAGE section

---

[1] This guidance has been prepared by the Office of Medical Policy in the Center for Drug Evaluation and Research (CDER) in cooperation with the Center for Biologics Evaluation and Research (CBER) at the Food and Drug Administration.

[2] This guidance applies to drugs, including biological drug products. For the purposes of this guidance, *drug product* or *drug* will be used to refer to human prescription drug and biological products that are regulated as drugs, except when there is a difference in the regulation. In such cases, *biological products* will be used. This guidance does not apply to those biological products that are also devices.

[3] See the final rule "Requirements on Content and Format of Labeling for Human Prescription Drug and Biological Products" (71 FR 3922, January 24, 2006) and additional labeling guidances at https://www.fda.gov/Drugs/GuidanceComplianceRegulatoryInformation/LawsActsandRules/ucm084159.htm. We update guidances periodically. To make sure you have the most recent version of a guidance, check the FDA guidance web page at https://www.fda.gov/RegulatoryInformation/Guidances/default.htm.

- How to write, organize, and format the information within the INDICATIONS AND USAGE section

The purpose of this guidance is to help ensure that the INDICATIONS AND USAGE section is clear, concise, useful, and informative and, to the extent possible, consistent within and across drug and therapeutic classes. Applicants should follow the recommendations in this guidance when developing the INDICATIONS AND USAGE section for a new drug and when revising this section for a currently approved drug, including when seeking approval of a new indication.

In general, FDA's guidance documents do not establish legally enforceable responsibilities. Instead, guidances describe the Agency's current thinking on a topic and should be viewed only as recommendations, unless specific regulatory or statutory requirements are cited. The use of the word *should* in Agency guidances means that something is suggested or recommended, but not required.

II. GENERAL PRINCIPLES

The primary role of the INDICATIONS AND USAGE section of labeling is to enable health care practitioners to readily identify appropriate therapies for patients by clearly communicating the drug's approved indication(s). Among other information, the INDICATIONS AND USAGE section states the disease or condition, or manifestation or symptoms thereof, for which the drug is approved, as well as whether the drug is indicated for the treatment, prevention, mitigation, cure, or diagnosis of that disease or condition, including relief of symptoms (21 CFR 201.57(c)(2)). Other sections of labeling (e.g., DOSAGE AND ADMINISTRATION, CONTRAINDICATIONS, WARNINGS AND PRECAUTIONS, USE IN SPECIFIC POPULATIONS), as applicable, also provide essential details that enable safe and effective use of a drug, and labeling should be considered in its entirety for individual prescribing decisions.

To comply with the general labeling requirements in 21 CFR 201.56 and 201.57, the INDICATIONS AND USAGE section must:

- Reflect the scientific evidence accurately

- Be concisely written to include the information necessary to clearly convey the use(s) for which the drug has been shown to be safe and effective

- Use terminology that is clinically relevant and scientifically valid and understandable to health care practitioners

Additionally, indications that are straightforward, clear, concise, and consistently written will facilitate the indexing of indications in electronic drug databases. This may, in turn, assist health care practitioners in searching indications in electronic medical information systems, thereby providing easier access to the information in FDA-approved labeling needed for clinical decision making.

A. Scope of the Indication(s)

Governing regulations articulate parameters for the evidentiary standard necessary for an indication to be listed in the INDICATIONS AND USAGE section of labeling. For drug products other than biological products, absent an applicable waiver, "all indications listed in the INDICATIONS AND USAGE section must be supported by substantial evidence of effectiveness based on adequate and well-controlled studies" as defined in 21 CFR 314.126(b) (§ 201.57(c)(2)(iv)).[4] For biological products, indications "must be supported by substantial evidence of effectiveness" (§ 201.57(c)(2)(v)). Any statements in this section of the labeling comparing the safety or effectiveness of drug or biological products with other agents for the same indications must be similarly supported – that is, for drugs, they must be supported by substantial evidence of effectiveness based on adequate and well-controlled studies and, for biological products, they must be supported by substantial evidence of effectiveness (§ 201.57(c)(2)(iii)).

Pursuant to the governing regulations, "[i]ndications or uses must not be implied or suggested in other sections of the labeling if not included" in the INDICATIONS AND USAGE section (§ 201.57(c)(2)(iv) and (v)). However, FDA may require a specific warning relating to an unapproved use in the WARNINGS AND PRECAUTIONS section of the labeling if the drug is commonly prescribed for a disease or condition and if such usage is associated with a clinically significant risk or hazard (§ 201.57(c)(6)(i)).[5]

1. Scope of an Indication Relative to the Population Studied

The INDICATIONS AND USAGE section should clearly communicate the scope of the approved indication, including the population to which the determination of safety and effectiveness is applicable. The indicated population may mirror the studied population, for example, in terms of patient demographics or severity of disease or condition, but can sometimes differ. In some cases, FDA's expert reviewers may fairly and responsibly conclude, based on their scientific training and experience, that the available evidence supports approval of an indication that is broader or narrower in scope than the precise population studied.[6] Applicants should discuss the scope of a proposed indication with the applicable review division.[7]

Indications may be written to include certain patient populations that may have been absent or specifically excluded from the clinical studies that supported approval (e.g., geriatric patients,

---

[4] The Director of CDER may, on the Director's own initiative or on the petition of an interested person, waive in whole or in part any of the criteria in 21 CFR 314.126(b) with respect to a specific clinical investigation, either prior to the investigation or in the evaluation of a completed study. A waiver petition must explain why the study, as conducted, will still yield substantial evidence of effectiveness (see 21 CFR 314.126(c)). Additionally, an applicant may submit a request to the Director of CDER or the Director of CBER asking for a waiver of any requirement under 21 CFR 201.56, 201.57 or 201.80 (see 21 CFR 201.58).

[5] See the guidance for industry *Warnings and Precautions, Contraindications, and Boxed Warning Sections of Labeling for Human Prescription Drug and Biological Products — Content and Format*.

[6] See generally 21 U.S.C 355(d).

[7] See 21 CFR 312.41.

pregnant women, patients taking certain concomitant drugs, patients with a different severity or stage of a disease). An indication for a broader population than the patient population studied in controlled trials may be appropriate after careful consideration of the generalizability of the evidence, consistencies in the disease process across different groups, and the drug's overall benefits and risks.

For example, if a study evaluating a drug in adults enrolled patients of a certain age range and excluded patients taking certain concomitant drugs, and available evidence does not suggest the drug would be unsafe or ineffective in adult patients outside that age range or in those taking the other drugs, the indication should be worded to reflect the broader age group (i.e., "in adults") rather than the exact ages studied. In addition, unless available evidence suggests otherwise, the indication should not exclude use in patients taking the concomitant drugs. Recommendations regarding age groups outside of an adult population are discussed in section II.A.2.

Similarly, if a drug were studied only in patients with a moderate form or stage of a disease and there is reason to believe, based on the generalizability of the data, consistencies in the disease process, and the drug's benefits and risks, that the drug would be both safe and effective in a broader group with the condition, an indication covering the broader population may be appropriate. In some cases, an indication covering the overall disease population can be considered. Specifics regarding the patient population studied should be described in the CLINICAL STUDIES section of the labeling.

Conversely, an indication may be approved for a population narrower than that which was studied. For example, a study may enroll and randomize patients, but then stratify participants by the presence or absence of a specific genomic marker. If the study demonstrated benefit only in patients who had tested positive for the marker, FDA's expert reviewers may fairly and responsibly conclude, based on their scientific training and experience, that the available evidence supports approval of an indication in a population that is narrower in scope than the population that was studied.[8]

There may also be circumstances in which the indication should reflect the precise population studied. For example, some study designs such as prognostic enrichment strategies (e.g., enrolling only people with a prior myocardial infarction in a study examining the effects of an antiplatelet drug) and most predictive enrichment strategies (e.g., enrolling only people with a specific genomic marker) may identify the population in which the benefits outweigh the risks or the only population in which effectiveness is reasonably likely.[9] In such cases, the indication should reflect only the population studied, unless and until evidence becomes available to support a determination that broader safety and effectiveness can be expected.

2. Age Groups in Indications

---

[8] See generally 21 USC 355(d).

[9] See the draft guidance for industry *Enrichment Strategies for Clinical Trials to Support Approval of Human Drugs and Biological Products*. When final, this guidance will represent FDA's current thinking on this topic.

Approval of a drug in pediatric patients[10] is generally based on sufficient data from studies in the following populations:

- A pediatric population only
- Both adult and pediatric populations
- Adults, with supporting data in a pediatric population (e.g., safety, pharmacokinetic data) that allow extrapolation of effectiveness to a pediatric population[11]
- One pediatric population that allows extrapolation of effectiveness to another pediatric population[12]

In certain circumstances (see section II.A.1), it may be appropriate to consider an indication for an adult population in an age group broader than the population that was studied. However, this approach is generally not appropriate across pediatric populations or between adult and pediatric populations because of the statutory requirements related to pediatric assessments[13] and the unique clinical considerations for pediatric patients. For example, pediatric patients may metabolize drugs differently from adults (in an age-related manner), are susceptible to different safety risks, and often require different dosing regimens even after correction for weight.

For these reasons, age groups should be included in indications. As such, an indication should state that a drug is approved, for example, "in adults," "in pediatric patients X years of age and older," or "in adults and pediatric patients X years of age and older."

Applicants should discuss the scope of and age groups for a proposed indication with the applicable review division.[14]

---

[10] The labeling regulations define *pediatric patients* as those ranging in age from birth through 16 years (21 CFR 201.57(c)(9)(iv)).

[11] Although it may be appropriate to extrapolate effectiveness, it is generally not appropriate to extrapolate safety with respect to pediatric populations.

[12] See section 505B(a)(2)(B) of the Federal Food, Drug, and Cosmetic Act (FD&C Act) and 21 CFR 201.57(c)(9)(iv). See also the draft guidance for industry and review staff *Pediatric Information Incorporated Into Human Prescription Drug and Biological Products Labeling*. When final, this guidance will represent FDA's current thinking on this topic.

[13] The Pediatric Research Equity Act (Public Law 108-155) generally requires certain applications for, among other things, a new indication to contain a pediatric assessment unless the applicant has obtained a waiver or deferral. Pediatric assessments "shall contain data, gathered using appropriate formulations for each age group for which the assessment is required that are adequate (i) to assess the safety and effectiveness of the drug or the biological product for the claimed indications in all relevant pediatric subpopulations; and (ii) to support dosing and administration for each pediatric subpopulation for which the drug or the biological product is safe and effective" (section 505(B)(a) of the FD&C Act).

[14] See 21 CFR 312.41.

B. Distribution of Information Among Labeling Sections

Generally, the section of the full prescribing information to which particular drug information is most relevant will contain the most detailed discussion of such information. Other sections should discuss only those aspects of the information that are pertinent to those other sections' scopes and purposes. There may be instances when it is necessary to include information in the INDICATIONS AND USAGE section that is discussed in greater detail elsewhere in the labeling. For example, the INDICATIONS AND USAGE section may include a limitation of use that has a cross-reference to a more detailed discussion of the information supporting the limitation in the WARNINGS AND PRECAUTIONS section (see section III.B). Because detailed information about topics such as clinical studies and risks related to limitations of use will generally be found elsewhere in the labeling, the information in the INDICATIONS AND USAGE section should be concise.

C. Updating the INDICATIONS AND USAGE Section

The INDICATIONS AND USAGE section "must be updated when new information becomes available that causes the labeling to be inaccurate, false, or misleading" (§ 201.56(a)(2)).[15] In addition, it is appropriate in certain circumstances for application holders to update this section to reflect current practices for writing indications for a particular group of drugs (for example, when more information becomes available about the drug, drug class, or specific disease or when the endpoints become better established). Application holders should review the INDICATIONS AND USAGE section regularly to ensure that it reflects current science and, to the extent possible, maintains consistency within a pharmacologic or therapeutic class.[16]

III. CONTENT AND FORMAT OF THE INDICATIONS AND USAGE SECTION

The INDICATIONS AND USAGE section includes the indication and, as appropriate, any identified limitations of use.[17] The INDICATIONS AND USAGE section "must state that the drug is indicated for the treatment, prevention, mitigation, cure, or diagnosis of a recognized disease or condition, or of a manifestation of a recognized disease or condition, or for the relief of symptoms associated with a recognized disease or condition" (§ 201.57(c)(2)). When drafting the INDICATIONS AND USAGE section, applicants should consider what information is needed to clearly convey the approved indication and whether other information in addition to the identification of the disease or condition is warranted.

For many drugs, the indication will be sufficiently conveyed by stating the disease or condition being treated, prevented, mitigated, cured, or diagnosed, and the approved age group(s) (see section II.A.). For example, indications may be straightforward for many conditions (e.g.,

---

[15] Application holders update their labeling using the procedures in 21 CFR 314.70 or 601.12, as applicable.

[16] See generally 21 CFR 201.56(a).

[17] See 21 CFR 201.57(c)(2).

symptomatic conditions such as pain, allergic rhinitis). In such circumstances, endpoints and descriptions of benefit should be summarized in the CLINICAL STUDIES section of labeling and should not be included in the indication.

On the other hand, other scenarios may warrant the inclusion of more information in the indication. Such scenarios could include cases in which a drug may target different aspects of a disease (e.g., in multiple sclerosis) or cases where endpoints are not well-standardized (e.g., in heart failure); in these scenarios, the specific benefits of the drug should be stated. For example, for a drug indicated for the treatment of insomnia, the indication should state whether the drug affects sleep onset, sleep maintenance, or both, in order to facilitate appropriate prescribing for an individual patient. Similarly, for many outcome studies, when there is an overall effect on a composite endpoint, the indication should identify the components of the composite (e.g., cardiovascular death, myocardial infarction, and stroke). In such cases, it would be critical to clearly state in the indication what benefit the drug has been shown to convey (see section III.C.1).

Details of studies that describe the basis for approval (e.g., "Effectiveness was demonstrated in two 12-week trials in patients with FEV_1 less than 60% of predicted.") should not be included in the INDICATIONS AND USAGE section. This section is not intended to be a description of the data supporting the determination of effectiveness, and the inclusion of such statements here could have the unintended consequence of inappropriately limiting use of the drug in practice (e.g., inadvertently suggesting short-term use of a drug indicated for a chronic condition). Likewise, discussions of disease definitions (e.g., diagnostic criteria for major depressive disorder) should not be included. These types of details should be discussed in the CLINICAL STUDIES section of labeling (see section III.C.1).

Specific components of and other considerations for the INDICATIONS AND USAGE section are discussed in detail in sections A through D below.

A. Indication

The indication should begin "DRUG-X is indicated" and must include the following elements required under 21 CFR 201.57(c)(2)(i):

- The disease, condition, or manifestation of the disease or condition (e.g., symptom(s)) being treated, prevented, mitigated, cured, or diagnosed

- When applicable, other information necessary to describe the approved indication (e.g., descriptors of the population to be treated, adjunctive or concomitant therapy, or specific tests needed for patient selection)

The following subsections provide details on each element of an indication listed above, along with illustrative examples demonstrating how to draft these elements so they are clear, concise, and easily identifiable and searchable.

1. The Disease, Condition, or Manifestation Being Treated, Prevented, Mitigated, Cured, or Diagnosed

The INDICATIONS AND USAGE section must state that the drug "is indicated for the treatment, prevention, mitigation, cure, or diagnosis of a recognized disease or condition, or of a manifestation of a recognized disease or condition, or for relief of symptoms associated with a recognized disease or condition" (§ 201.57(c)(2)). The disease, condition, or manifestation should be included in the indication using high-level terms that are clinically relevant and scientifically valid (e.g., asthma, diabetes mellitus, pain). Although FDA does not endorse any particular resource for terms used to describe diseases, conditions, or symptoms, all terminology should be well understood and easily recognizable by health care practitioners.

2. Other Information Necessary To Describe the Approved Indication

In addition to identifying the disease, condition, or symptom for which the drug is approved, there may be additional critical aspects of an indication that are important to include. Examples of such situations are described in items a through c below.

a. Selected patient subgroups or disease subpopulations for whom the drug is approved

In some cases, additional descriptors or qualifiers are critical to include as part of the indication to clearly identify the patient population for whom the drug is approved. In addition to including the approved age group(s) (see section II.A.2), other circumstances in which such additional information would be important include, but are not limited to, indicating a drug for patients previously treated with other therapies (e.g., hormone-refractory prostate cancer), patients with a certain classification of a disease (e.g., World Health Organization Group I pulmonary arterial hypertension), or patients with other important identifying variables (e.g., immunocompetent patients). For example, if a drug is for use only in patients with a history of coronary disease events (i.e., as secondary prevention), the indication should clearly convey the patient population for which the drug is approved.

If evidence is available to support the safety and effectiveness of the drug only in selected subgroups of the larger population with the target disease or condition, "this section must include…a succinct description of the limitations of usefulness" (§ 201.57(c)(2)(i)(B)). Thus, the indication should include information on the subgroup(s) for whom the drug is approved. For example:

- DRUG-X is indicated for the treatment of adult and pediatric patients 12 years of age and older with moderate to severe plaque psoriasis who are candidates for phototherapy or systemic therapy.

If a drug should be reserved for use in specific situations (e.g., cases refractory to other drugs) because of safety concerns, "this section must include…a statement of the information" pertaining to such situations (§ 201.57(c)(2)(i)(E)). For example:

- DRUG-X is indicated for the treatment of moderate to severe active rheumatoid arthritis in adult patients who have had an inadequate response to TNF antagonist therapy.

For drugs approved for use only after other drug therapies have failed (e.g., an indication for second-line use), consideration should be given as to whether it is necessary to specify the name of the drug(s) or drug class(es) the patients are to have initially received or instead to word the indication more broadly (e.g., for use in previously treated patients).

b. Adjunctive or concomitant therapy or therapeutic modalities to use before initiating drug therapy, such as diet or exercise or another drug

If the drug is approved for use only in conjunction with a primary mode of therapy (e.g., diet, surgery, behavior changes, or another drug), "[t]his section must include…a statement that the drug is indicated as an adjunct to that mode of therapy" (§ 201.57(c)(2)(i)(A)). For example:

- DRUG-X is indicated in adults for the treatment of high-grade malignant glioma as an adjunct to surgery and radiation.

For drugs approved for use as adjunctive therapy, consideration should be given as to whether it is necessary to specify the name of the drug(s) or drug class(es) the patients are to receive concomitantly or instead to word the indication more broadly (e.g., as adjunctive therapy or as part of a combination regimen).

c. Specific tests needed to select patients in whom to use the drug

If specific tests are necessary for selection or monitoring of patients who need the drug, "[t]his section must include…the identity of such tests" (§ 201.57(c)(2)(i)(C)).[18] For example:

- DRUG-X is indicated for the treatment of adult patients with metastatic non-small cell lung cancer whose tumors are anaplastic lymphoma kinase (ALK)-positive as detected by an FDA-approved test.

In general, information on tests used for monitoring appears in other labeling sections (e.g., DOSAGE AND ADMINISTRATION or WARNINGS AND PRECAUTIONS).[19]

---

[18] When appropriate, the labeling should identify the type of FDA-approved or cleared in vitro companion diagnostic device with which the product is approved, rather than a particular manufacturer's device. See the guidance for industry and FDA staff *In Vitro Companion Diagnostic Devices*.

[19] See the following two guidances for industry: (1) *Dosage and Administration Section of Labeling for Human Prescription Drug and Biological Products — Content and Format* and (2) *Warnings and Precautions, Contraindications, and Boxed Warning Sections of Labeling for Human Prescription Drug and Biological Products — Content and Format*.

B. Limitations of Use

Limitations of use are presented separately from the indication within the INDICATIONS AND USAGE section (see section III.D.2). A limitation of use is included when there is reasonable concern or uncertainty among FDA's expert reviewers, who are qualified by scientific training and experience, about a drug's risk-benefit profile. Limitations of use should be distinguished from contraindications. A contraindication "must describe any situations in which the drug should not be used because the risk of use (e.g., certain potentially fatal adverse reactions) clearly outweighs any possible therapeutic benefit" (§ 201.57(c)(5)). However, there are cases in which the evidence falls short of requiring a contraindication, but suggests that use of the drug may be inadvisable. There are also cases in which there is sufficient uncertainty about the drug's benefits in certain clinical situations to suggest that the drug should generally not be used in those settings. In these cases, a limitation of use may be appropriate. To avoid redundancy within the labeling, contraindications should not be restated as limitations of use in the INDICATIONS AND USAGE section.

Limitations of use should be included in the INDICATIONS AND USAGE section only when the awareness of such information is important for practitioners to ensure the safe and effective use of the drug. In most cases, limitations of use will identify a particular patient population in which a drug should generally not be used. If evidence is available to support the safety and effectiveness of the drug only in selected subgroups of the larger population, the INDICATIONS AND USAGE section "must include…a succinct description of the limitations of usefulness of the drug and any uncertainty about anticipated clinical benefits, with reference to the 'Clinical Studies' section for a discussion of the available evidence" (§ 201.57(c)(2)(i)(B)). Such information would be appropriate to include as a separate limitation of use — rather than narrowing the language of the indication itself — when needed to inform practitioners that there is a reasonable concern or uncertainty about the drug's safety or effectiveness outside the specific population for which the drug is approved.

In contrast, information that essentially narrows or further defines a drug's approved indication and is used to direct appropriate therapy (e.g., identifying particular subsets of the population for whom the drug is approved, drugs to be used only after other drug therapies have failed, or specific tests needed to identify patients to be treated) should be incorporated directly into the indication whenever possible (see section III.A.2). This information should not be presented as a separate limitation of use. Whereas a limitation of use most often will be included to identify a patient population in which the drug should generally *not* be used (i.e., discouraging its use), information that specifies the patient population in which the drug *should* be used (i.e., encouraging its use) should, wherever possible, be incorporated in the indication itself. For example, if a drug should be used only after failure of or as an adjunct to another drug or treatment modality, the indication should include this information rather than having it presented separately as a limitation of use.

Although there are invariably areas of uncertainty about a drug's effectiveness, not all drugs will include limitations of use in the INDICATIONS AND USAGE section. Information considered for a limitation of use should be evaluated to decide if it may be better suited to another section of the labeling (e.g., WARNINGS AND PRECAUTIONS, USE IN SPECIFIC POPULATIONS,

CLINICAL STUDIES). For example, although there may be circumstances in which a limitation of use will be further described in (and cross-referenced to) a subsection in the WARNINGS AND PRECAUTIONS section, most warnings and precautions will typically not be repeated as limitations of use. Only information that provides a clearer understanding of the scope of the approved indication to facilitate safe and effective prescribing decisions should be included as a limitation of use. Moreover, an absence of data in a particular population subset should generally not appear as a limitation of use unless there is reasonable concern about the drug's safety or effectiveness in that group.

1. Situations in Which Limitations of Use Would Be Appropriate

The following are examples of situations in which it may be appropriate to include a separate limitation of use within the INDICATIONS AND USAGE section:

a. Drugs for which there is reasonable concern or uncertainty about effectiveness or safety in a certain clinical situation

As recommended in section II.A.2, the approved age group(s) should be included in an indication. If there is a concern or uncertainty about safety or effectiveness in a population outside the approved age group (e.g., younger patients), a limitation of use should be included about that population. The inclusion of a limitation of use will differentiate between (1) a circumstance in which use of the drug in a certain population outside of the approved population raises a reasonable concern or uncertainty about safety or effectiveness and (2) a circumstance in which an indication is simply directed to a certain group (e.g., patients within a particular age range). The concern that warranted the limitation of use should typically be described elsewhere in labeling (e.g., WARNINGS AND PRECAUTIONS and USE IN SPECIFIC POPULATIONS sections), with a cross-reference in the limitation of use to the section of labeling where this detailed information can be found. For example:

- DRUG-X is indicated for the treatment of hypertension in adults and pediatric patients 1 year of age and older.

  <u>Limitations of Use</u>
  In patients younger than one year of age, DRUG-X can adversely affect kidney development *[see Warnings and Precautions (5.X) and Use in Specific Populations (8.4)]*

The governing regulation states that "[i]f there is a common belief that a drug may be effective for a certain use or if there is a common use of the drug for a condition, but the preponderance of evidence related to the use or condition shows that the drug is ineffective or that the therapeutic benefits do not generally outweigh its risks, FDA may require that [the INDICATIONS AND USAGE] section state that there is a lack of evidence that the drug is effective or safe for that use or condition" (§ 201.57(c)(2)(ii)). A limitation of use may be of particular importance in these circumstances if proven alternative therapies exist for the condition in question. For example:

- DRUG-X is indicated in adults for the acute treatment of migraine headache with or without aura.

  Limitations of Use
  Multiple clinical trials failed to establish the effectiveness of DRUG-X for the prophylaxis of migraine headaches *[see Clinical Studies (14.X)]*.

b. Drugs approved without evidence of benefits known to occur with other drugs in the same class

If a drug is approved without having demonstrated a particular benefit that has been demonstrated with other drugs in the same pharmacologic or therapeutic class, it may be important to convey the differences among products under a "Limitations of Use" heading in the INDICATIONS AND USAGE section. For example, the INDICATIONS AND USAGE section for a new HMG-CoA reductase inhibitor that is approved based on its serum lipid-lowering effects (without evidence of a beneficial effect on cardiovascular morbidity and mortality) would typically be presented as follows:

- DRUG-X is indicated as an adjunctive therapy to diet to reduce elevated total cholesterol, LDL cholesterol, apolipoprotein B, and triglycerides and to increase HDL cholesterol in adult patients with primary hyperlipidemia or mixed lipidemia.

  Limitations of Use
  The effect of DRUG-X on cardiovascular morbidity and mortality has not been determined.

c. Drugs with dose, duration, or long-term use considerations

If information on limitations of use or uncertainty about anticipated benefits is relevant to the recommended dosing intervals, to appropriate treatment duration when treatment should be limited, or to any dosage modification, the INDICATIONS AND USAGE section "must include…a concise description of the information, with a reference to the more detailed information in the 'Dosage and Administration' section" (§ 201.57(c)(2)(i)(D)). Under these circumstances, information about important dose or duration considerations, such as how long a drug can safely be used or uncertainty about the risks and benefits of treatment beyond a certain period (e.g., long-term cumulative toxicity), should be included as a limitation of use. For example:

- DRUG-X is indicated for the management of elevated plasma uric acid levels in adult patients with tumor lysis syndrome.

> Limitations of Use
> The activity of DRUG-X may be neutralized by the development of anti-drug antibodies if more than a single course of treatment is administered *[see Dosage and Administration (2.X) and Warnings and Precautions (5.X)]*.

It is generally not necessary to limit duration of use in the INDICATIONS AND USAGE section unless such a limited duration is essential to ensure the safe and effective use of the drug. If clinical trials evaluated the effectiveness of a drug for a chronic condition only in short-term trials of sufficient duration to support such an approval (e.g., drugs for major depressive disorder or hypertension), but the drug is indicated for long-term use due to the chronic nature of the condition and because there is no known or anticipated safety or efficacy concern from continued use, a description of the duration of use from the clinical trials or information about the lack of longer term data generally should not be included in the INDICATIONS AND USAGE section. Information on the length of the clinical trials should instead be discussed in detail in the CLINICAL STUDIES section of the labeling.

If there are specific conditions that should be met before the drug is used on a long-term basis (e.g., demonstration of responsiveness to the drug after short-term use in an individual patient), the INDICATIONS AND USAGE section "must include…a statement of the conditions; or if the indications for long term use are different from those for short term use, a statement of the specific indications for each use" (§ 201.57(c)(2)(i)(F)). For drugs with these characteristics, a limitation of use may be used to address such issues. For example:

- DRUG-X is indicated for the treatment of severe spasticity in adult patients with spinal cord injury, brain injury, or multiple sclerosis.

  Limitations of Use
  Prior to implantation of a device for chronic intrathecal infusion of DRUG-X, confirm a positive clinical response to DRUG-X in a screening phase *[see Dosage and Administration (2.X)]*.

*2. Situations in Which Limitations of Use Generally Would **Not** Be Appropriate*

Limitations of use generally would ***not*** be appropriate in the following situations:

a. To restate information already included in the indication

For example, if an indication is clearly worded as being approved for use in combination with another drug, there is no need for a limitation of use stating that the subject drug should be used only in combination and not as monotherapy.

b. To address the absence of data in populations in which the drug was not studied

For example, if an oncology drug was studied in and is indicated for use in patients with a cancer of a specific mutation, there should not be a limitation of use about the absence of data in patients with typical (wild-type) forms, unless there is reasonable concern about the drug's safety or effectiveness in such patients. Likewise, if a drug is approved to reduce the risk of rejection in patients receiving a heart transplant, there should not be a limitation of use about the lack of data on use in lung transplants. Similarly, if a vaccine is approved for use in children 12 months through 12 years of age, there should not be a limitation of use about the absence of data in other age groups.

C. Other Considerations for Writing the INDICATIONS AND USAGE Section

1. Identification of Outcomes, Endpoints, and Benefit(s) the Drug Conveys

The approved indication will generally convey the benefit of the treatment (i.e., the disease, condition, manifestation, or symptoms of the disease or condition being treated, prevented, mitigated, cured, or diagnosed), and it is usually not necessary to fully describe the specific way benefit was measured in clinical trials (i.e., identifying outcomes or endpoints) when the treatment affects a broad range of manifestations of the disease (e.g., an indication for the symptoms of allergic rhinitis). In some cases, however, a broad disease indication may not be appropriate because, for example, the drug may affect only certain signs, symptoms, or manifestations of the disease (see section III). An indication identifying an outcome or endpoint may be considered, for example, when the drug's effect on the overall disease is not well understood, when different drugs have different effects on various manifestations of the diseases, when clinical trials evaluated only one or some of the manifestations of the disease, or when the endpoints are different from typical effectiveness measures. For example:

- DRUG-X is indicated to improve walking in adult patients with multiple sclerosis.

For certain other conditions, the drug's indication may be to reduce the risk of significant morbidity and mortality, which describes the demonstrated benefit more accurately than would a more broadly written indication indicating the product simply as a treatment for the condition itself. In such cases, the specific endpoint(s) for which the drug has demonstrated benefits should be incorporated into the indication. For example:

- DRUG-X is indicated to reduce the risk of nonfatal myocardial infarction, fatal and nonfatal stroke, and revascularization procedures in adult patients with clinically evident coronary heart disease.

The CLINICAL STUDIES section of labeling "must discuss those studies that facilitate an understanding of how to use the drug safely and effectively" (§ 201.57(c)(15)). The information presented in that section ordinarily includes, among other things, a description of the study population, endpoints, and results. For example, if an indication were written for an overall effect on a composite endpoint, the details on the endpoints studied and results (e.g., which

component of a composite endpoint drove the overall combined finding) would be discussed in detail in the CLINICAL STUDIES section. Additionally, if only one component of a composite primary endpoint was affected and indicating the drug for the composite would misrepresent the true result, an indication for the single component can be considered, with the explanation of the study results summarized in the CLINICAL STUDIES section.

2. *Accelerated Approval*

If a drug is approved for an indication based on an effect on a surrogate endpoint or an intermediate clinical endpoint under section 506(c) of the Federal Food, Drug, and Cosmetic Act (FD&C Act) (21 U.S.C. 356(c)) and 21 CFR 314.510 or 601.41 (i.e., accelerated approval), the INDICATIONS AND USAGE section "must include…a succinct description of the limitations of usefulness of the drug and any uncertainty about anticipated clinical benefits, with a reference to the Clinical Studies section for a discussion of the available evidence" (§ 201.57(c)(2)(i)(B)).[20]

3. *Required or Recommended Language*

Under governing statutory and regulatory provisions, certain products have required or recommended language for the INDICATIONS AND USAGE section. For example:

- Labeling for systemic antibacterial drug products must include a specific statement in the INDICATIONS AND USAGE section about strategies for reducing the development of drug-resistant bacteria and maintaining the effectiveness of the subject drug and other antibacterial drugs (21 CFR 201.24(b)).

- Section 505(u)(2)(B) of the FD&C Act (21 U.S.C. 355(u)(2)(B)) requires that labeling for certain products containing a single enantiomer of a previously approved racemic drug include a statement that the non-racemic product is not approved, and has not been shown to be safe and effective, for any condition of use of the previously approved racemic drug. For such products approved under 505(u), this information should be presented as a limitation of use.

- Other FDA guidances (e.g., clinical/medical guidances) recommend specific wording for the INDICATIONS AND USAGE section for certain indications.[21]

[20] See the draft guidance for industry *Labeling for Human Prescription Drug and Biological Products Approved Under the Accelerated Approval Regulatory Pathway*. When final, this guidance will represent FDA's current thinking on this topic.

[21] Additional labeling guidances are available on the FDA Drugs guidance web page at http://www.fda.gov/Drugs/GuidanceComplianceRegulatoryInformation/Guidances/default.htm.

4. Preferred Wording and Wording Generally To Avoid[22]

Consistent with this guidance and the regulatory framework, as a general matter, care should be taken when considering use of the following terms and phrases:

a. "Reduce the risk" versus "prevent"

If the indication for a drug is to reduce the risk of the occurrence of a particular clinical outcome, phrases such as "reduce the risk of" or "reduce the incidence of" should be considered rather than using "prevent" in the indication. The use of a term such as *prevent* may imply a guarantee of success that is not supported by the data. However, for certain indications, the use of terms such as *prevent* (e.g., for preventive vaccines) or *prophylaxis* (e.g., drugs for post-exposure prophylaxis) in the indication may be appropriate because, in a given context, these terms are well established and understood by the clinical community.

b. "Only"

The INDICATIONS AND USAGE section should be worded clearly to convey the approved use of the drug, making inclusion of the word "only" unnecessary (i.e., the indication generally should ***not*** state "DRUG-X is indicated only for…").

c. "Also indicated"

When a new indication is added to the INDICATIONS AND USAGE section, the phrase "is also indicated" generally should ***not*** be used because it may imply that the new indication is less important than the existing indication(s).

d. Product identification in the indication

The indication should include the proprietary name (or trade name). If the product does not have a proprietary or trade name, the indication should include the nonproprietary name (i.e., established name for a drug product or proper name for a biological product).

To avoid unnecessary clutter and to enhance clarity, other information (such as the non-proprietary name, dosage form, route of administration) generally should not be included in the indication. The established pharmacologic class appears with the indication only in Highlights (§ 201.57(a)(6)).

D. Formatting the INDICATIONS AND USAGE Section

1. Format for Multiple Indications

When a drug is approved for more than one indication, the format of the INDICATIONS AND USAGE section should be carefully considered. For some drugs, it may be preferable to assign a subsection to each indication (e.g., 1.1 Disease-A, 1.2 Disease-B), but for others, it may be

[22] See generally 21 CFR 201.56.

preferable to present distinct indications using only bullets (e.g., “DRUG-X is indicated for:” followed by a bulleted list) immediately under the main section heading or within a subsection.

2. Format for Limitations of Use

Limitations of use are presented separately from the indication within the INDICATIONS AND USAGE section, under the heading *Limitations of Use* and not usually under a separate numbered subsection. If, however, a drug has multiple indications and the limitations of use apply to all of them, it may be preferable to use a separate numbered subsection for *Limitations of Use* within the section. The INDICATIONS AND USAGE section should be formatted to clearly show if the limitations apply to all or to only some of the indications.

COMPOSITE BINDER FOR THE USE IN SPECIFIC POPULATIONS

Ethical Considerations for Clinical Investigations of Medical Products Involving Children

Guidance for Industry, Sponsors, and IRBs

DRAFT GUIDANCE

This guidance document is being distributed for comment purposes only.

Comments and suggestions regarding this draft document should be submitted within 90 days of publication in the *Federal Register* of the notice announcing the availability of the draft guidance. Submit electronic comments to https://www.regulations.gov. Submit written comments to the Dockets Management Staff (HFA-305), Food and Drug Administration, 5630 Fishers Lane, Rm. 1061, Rockville, MD 20852. All comments should be identified with the docket number listed in the notice of availability that publishes in the *Federal Register*.

For questions regarding this draft document, contact (OPT) Donna Snyder at 301-796-1397.

U.S. Department of Health and Human Services
Food and Drug Administration
Office of Pediatric Therapeutics (OPT)
Center for Drug Evaluation and Research (CDER)
Center for Biologics Evaluation and Research (CBER)
Center for Devices and Radiological Health (CDRH)

September 2022
Clinical/Medical

Ethical Considerations for Clinical Investigations of Medical Products Involving Children
Guidance for Industry, Sponsors, and IRBs

Additional copies are available from:
Office of Pediatric Therapeutics
Office of Clinical Policy and Programs, Office of the Commissioner, FDA
10903 New Hampshire Avenue
Silver Spring, MD 20993
(Tel) 301-796-1397
and/or:
Office of Communications, Division of Drug Information, CDER, FDA
10001 New Hampshire Ave.,
Silver Spring, MD 20993-0002
Email: druginfo@fda.hhs.gov
https://www.fda.gov/drugs/guidance-compliance-regulatory-information/guidances-drugs
and/or:
Office of Communication, Outreach, and Development, CBER, FDA
10903 New Hampshire Ave., Bldg. 71, Room 3128
Silver Spring, MD 20993-0002
Phone: 800-835-4709 or 240-402-8010; Email: ocod@fda.hhs.gov
https://www.fda.gov/vaccines-blood-biologics/guidance-compliance-regulatory-information-biologics/biologics-guidances
and/or:
Office of Policy, Guidance and Policy Development, CDRH, FDA
10903 New Hampshire Ave., Bldg. 66, Room 5431
Silver Spring, MD 20993-0002
Email: CDRH-Guidance@fda.hhs.gov
https://www.fda.gov/medical-devices/device-advice-comprehensive-regulatory-assistance/guidance-documents-medical-devices-and-radiation-emitting-products

U.S. Department of Health and Human Services
Food and Drug Administration
Office of Pediatric Therapeutics (OPT)
Center for Drug Evaluation and Research (CDER)
Center for Biologics Evaluation and Research (CBER)
Center for Devices and Radiological Health (CDRH)

September 2022
Clinical/Medical

Table of Contents

I. INTRODUCTION **1**

II. BACKGROUND **2**

III. ETHICAL FRAMEWORK **2**

A. Principle of Scientific Necessity 3

B. Risk Categories for Interventions or Procedures without Prospect of Direct Benefit 4

C. Prospect of Direct Benefit 5

D. Assessment of Risk for Interventions or Procedures with a Prospect of Direct Benefit 6

E. Component Analysis 7

F. Potential for Review per 21 CFR 50.54 8

G. Parental/Guardian Permission and Child Assent 9

IV. APPLICATION OF SUBPART D TO PEDIATRIC CLINICAL INVESTIGATIONS **10**

A. Data to Support Conducting Pediatric Clinical Investigations 10

B. Design Considerations for Clinical Investigations 11

1. Clinical Investigations of Drugs 11

2. Clinical Investigations of Medical Devices 12

C. Study Procedures in Pediatric Clinical Investigations 13

1. Procedural Sedation in Pediatric Clinical Investigations 13

Ethical Considerations for Clinical Investigations of Medical Products Involving Children
Guidance for Industry, Sponsors, and IRBs

This draft guidance, when finalized, will represent the current thinking of the Food and Drug Administration (FDA or Agency) on this topic. It does not establish any rights for any person and is not binding on FDA or the public. You can use an alternative approach if it satisfies the requirements of the applicable statutes and regulations. To discuss an alternative approach, contact the FDA staff responsible for this guidance as listed on the title page.

I. INTRODUCTION

Clinical investigations[1] in children are essential for obtaining data on the safety and effectiveness of drugs, biological products,[2] and medical devices (collectively referred to as "medical products" herein) in children and to protect children from the risks associated with exposure to medical products that may be unsafe or ineffective. Children[3] are a vulnerable population who cannot consent for themselves and who therefore are afforded additional safeguards when participating in a clinical investigation. Such safeguards are an essential requirement for the initiation and conduct of pediatric investigations as part of a medical product development program. This guidance describes the FDA's current thinking regarding ethical considerations for clinical investigations of medical products in children.[4] Clinical investigations involving FDA-regulated products that are not medical products may have similar ethical considerations to those discussed in this guidance but are outside the scope of this guidance.

In general, FDA's guidance documents do not establish legally enforceable responsibilities. Instead, guidances describe the Agency's current thinking on a topic and should be viewed only as recommendations, unless specific regulatory or statutory requirements are cited. The use of the word *should* in Agency guidances means that something is suggested or recommended, but not required.

[1] FDA's regulations at 21 CFR 50.3(c) define the term *clinical investigation* as "any experiment that involves a test article and one or more human subjects and that either is subject to requirements for prior submission to the Food and Drug Administration under section 505(i) or 520(g) of the Act, or is not subject to requirements for prior submission to the Food and Drug Administration under these sections of the Act, but the results of which are intended to be submitted later to, or held for inspection by, the Food and Drug Administration as part of an application for a research or marketing permit." See also 21 CFR 56.102(c). In this guidance, the terms *trial*, *clinical trial,* and *study* have the same meaning as the term *clinical investigation*.
[2] For purposes of this guidance, references to drugs include drug products approved under section 505 of the Federal Food, Drug, and Cosmetic Act (21 U.S.C. 355) and biological products licensed under section 351 of the Public Health Service Act (42 U.S.C. 262).
[3] FDA's regulations at 21 CFR 50.3(o) define *children* as "persons who have not attained the legal age for consent to treatments or procedures involved in clinical investigations, under the applicable law of the jurisdiction in which the clinical investigation will be conducted." For the purposes of this guidance, *children* include neonates, infants, children, and adolescents who have not reached the legal age of consent in their local jurisdiction.
[4] See section II for information regarding the regulatory requirements.

II. BACKGROUND

The ethical principles for the protection of human subjects in FDA-regulated clinical investigations are reflected in the requirements in 21 CFR parts 50 and 56; additional safeguards for children are included in 21 CFR part 50, subpart D (*Additional Safeguards for Children in Clinical Investigations)*. Institutional review boards (IRBs) are required to follow these regulations when reviewing clinical investigations of FDA-regulated medical products that are intended to enroll children. 21 CFR part 50, subpart D parallels the Department of Health and Human Services regulations found in 45 CFR part 46, subpart D, *Additional Protections for Children Involved as Subjects in Research*.

All FDA-regulated clinical investigations of medical products are subject to the requirements in parts 50 and 56 regardless of whether they require an investigational device exemption (IDE) or an investigational new drug application (IND).[5] For studies requiring an IDE or an IND, sponsors are encouraged to discuss their investigational plan, including plans for pediatric drug development, with the relevant review division prior to submitting a protocol to the IDE or IND.[6]

III. ETHICAL FRAMEWORK

In accordance with 21 CFR 50.50, IRBs must review clinical investigations involving children as subjects and approve only those clinical investigations that satisfy the criteria described in 21 CFR 50.51, 50.52, or 50.53 and the conditions of all other applicable sections of subpart D.

- 21 CFR 50.51 (clinical investigations not involving greater than minimal risk) requires that the IRB find that no greater than minimal risk to children is presented (see section III.B) and adequate provisions are made for soliciting the assent of the children and the permission of their parents or guardians (see section III.G).

- 21 CFR 50.52 (clinical investigations involving greater than minimal risk but presenting the *prospect of direct benefit* to individual subjects) requires that the IRB find that the risk is justified by the anticipated benefit to subjects (see section III.C), the relation of the anticipated benefit to the risk is at least as favorable to the subjects as that presented by available alternative approaches (see section III.C), and adequate provisions are made for soliciting the assent of the children and the permission of their parents or guardians (see section III.G).

---

[5] 21 CFR 50.1 and 56.101.

[6] For additional information regarding clinical investigations of drugs involving children, see the ICH guidance for industry *E11(R1) Addendum: Clinical Investigation of Medicinal Products in the Pediatric Population (*April 2018). See also the FDA guidance for industry *Pediatric Study Plans: Content of and Process for Submitting Initial Pediatric Study Plans and Amended Initial Pediatric Study Plans* (July 2020). We update guidances periodically. For the most recent version of a guidance, check the FDA guidance web page at https://www.fda.gov/regulatory-information/search-fda-guidance-documents.

- 21 CFR 50.53 (clinical investigations involving greater than minimal risk and no *prospect of direct benefit* to individual subjects, but likely to yield generalizable knowledge about the subjects' disorder or condition[7]) requires that the IRB finds that:

    - The risk represents a *minor increase over minimal risk* (see section III.B);

    - The intervention or procedure presents experiences to subjects that are reasonably commensurate with those inherent in their actual or expected medical, dental, physiological, social, or educational situations (see section III.B);

    - The intervention or procedure is likely to yield generalizable knowledge about the subjects' disorder or condition that is of vital importance for the understanding or amelioration of the subjects' disorder or condition; and

    - Adequate provisions are made for soliciting the assent of the children and the permission of their parents or guardians (see section III.G).

  For children to be exposed to the level of risk described in 21 CFR 50.53, the children should either have or be at risk for the specific disorder or condition that will be studied in the clinical investigation.[8] Objective or empiric data should support that the condition proposed for study has the potential to negatively impact the child's health and well-being or increase the risk of developing a health problem in the future, as well as that collection of the data will enhance understanding towards prevention, diagnosis, improvement, or treatment of the condition.

The following are the fundamental concepts for the ethical framework in 21 CFR part 50, including subpart D, and 21 CFR part 56, and that IRBs should consider when reviewing clinical investigations that include children.

A. Principle of Scientific Necessity

The principle of scientific necessity[9] is encompassed in two regulatory requirements: the equitable selection of subjects (21 CFR 56.111(a)(3)) and minimization of risk (21 CFR 56.111(a)(1)). The concept is also grounded in the ethical principles of the Belmont Report,[10] specifically that of justice. IRBs should consider the scientific necessity of conducting a clinical investigation in children. It may be more efficient to consider scientific necessity prior to

[7] For the purposes of this guidance, references to *disorder* and *condition* include *diseases*.
[8] Institute of Medicine (2004); *Committee on Clinical Research Involving Children, Ethical Conduct of Clinical Research Involving Children*; Field MJ, Behrman RE, editors. Washington DC: National Academies Press. Recommendation 4.3. Available at https://www.ncbi.nlm.nih.gov/books/NBK25542/ (accessed September 19, 2022).
[9] Roth-Cline M, Nelson R. The ethical principle of scientific necessity in pediatric research. *Am J Bioeth*. 2014;14(12):14–15.
[10] The Belmont Report, Ethical Principles and Guidelines for the Protection of Human Subjects of Research, 1979, The National Commission for the Protection of Human Subjects of Biomedical and Behavioral Research, available at https://www.hhs.gov/ohrp/regulations-and-policy/belmont-report/read-the-belmont-report/index.html (accessed September 19, 2022).

assessing risk and benefit under 21 CFR part 50, subpart D. Children should not be enrolled into a clinical investigation unless their participation is necessary to answer an important scientific and/or public health question directly relevant to the health and welfare of children. For example, for products that are being developed for use in adults and children, if effectiveness in adults can be extrapolated to children, then effectiveness studies in adults should be conducted to minimize the need to collect effectiveness data in children.[11]

Regarding the equitable selection of subjects, IRBs should consider the purposes of the research and the setting where the research will be conducted and should be aware of the unique challenges of research involving children.[12] Regarding minimization of risk, research procedures should be consistent with sound research design and should not expose subjects to risk unnecessarily. When appropriate, procedures already being performed as part of clinical care should be used to meet research needs.

When it is considered scientifically necessary to conduct a clinical investigation in children, it is imperative that the clinical investigation be well-designed to collect interpretable data. Key elements of well-designed clinical investigations include the selection of appropriate control groups and study endpoints relevant in the pediatric population. Studies that are not well-designed expose children to unnecessary risks, are unlikely to yield informative study results and as a result may be considered unethical. In pediatric drug development, randomized, placebo-controlled trials may be necessary to establish safety and effectiveness.

B. Risk Categories for Interventions or Procedures without Prospect of Direct Benefit

Any intervention or procedure, including the administration of an investigational drug or use of an investigational medical device, undertaken as part of a clinical investigation in children may be associated with risk. The regulations at 21 CFR part 50, subpart D include two categories of risk for procedures or interventions in a clinical investigation that do not offer a *prospect of direct benefit*:

- *Minimal risk* means that the probability and magnitude of harm or discomfort anticipated in the research are not greater in and of themselves than those ordinarily encountered in daily life or during the performance of routine physical or psychological examinations or tests (21 CFR 56.102(i)). The standard of minimal risk should be interpreted as those risks encountered in the daily life of normal, average, healthy children living in safe environments and indexed to the experiences of children of the same age and developmental stage as the subject population. The experiences of a normal 2-year-old may be very different than the experiences of a normal 16-year-old. The duration of the exposure to the risk, the characteristics of the risk, and the reversibility of harm should

[11] See the guidance for industry and FDA staff *Leveraging Existing Clinical Data for Extrapolation to Pediatric Uses of Medical Devices* (June 2016) and the ICH guidance for industry *E11(R1)*. For additional information on pediatric extrapolation, see the ICH draft guidance for industry *E11A Pediatric Extrapolation* (August 2022) (when final, this guidance will represent the FDA's current thinking on this topic).
[12] See the guidance for industry *Enhancing the Diversity of Clinical Trial Populations – Eligibility Criteria, Enrollment Practices, and Trial Designs* (November 2020).

also be considered. Examples of minimal risk interventions or procedures may include a single blood draw, physical exam, chest x-ray,[13] or surveys.[14] Given that investigational drugs generally are considered to have the potential to cause harm, the use of an investigational drug in a clinical investigation that includes children is unlikely to be considered minimal risk under 21 CFR part 50, subpart D. Investigational devices, however, can vary significantly in design and intended use (e.g., monitoring, diagnostic, or therapeutic devices). Depending on the investigational device (e.g., diagnostic versus therapeutic) and how it is used in the investigation, there could be device investigations that meet the criteria for minimal risk under 21 CFR part 50, subpart D.

- *Minor increase over minimal risk* should be understood to mean a slight increase over minimal risk that poses no significant threat to the child's overall health or well-being.[15] Any potential harms with the intervention or procedure should be expected to be transient and reversible and the probability for severe pain, discomfort, or harm should be extremely small or nonexistent. The setting and the experience level of the investigator are important factors to consider when making an assessment as to whether an intervention or procedure meets criteria as a *minor increase over minimal risk.*[16] Examples of interventions or procedures that might be considered a *minor increase over minimal risk* are a urine collection via a catheter, or bone marrow aspirate with topical pain relief,[17] or administering a single dose of an investigational drug with adequate safety information (see section IV.B).

See section IV for additional information regarding risk categories related to the design of the clinical investigation and research related procedures.[18]

C. Prospect of Direct Benefit

The level of certainty required for determining that a *prospect of direct benefit* exists is not commensurate with the rigorous standards for confirming effectiveness.[19] Consequently, effectiveness in adults does not need to be established before studies in children may begin. *Prospect of direct benefit* refers to the potential benefit to the individual child from exposure to

---

[13] Institute of Medicine (2004); *Committee on Clinical Research Involving Children, Ethical Conduct of Clinical Research Involving Children;* Field MJ, Behrman RE, editors. Washington DC: National Academies Press. Table 4.1, page 135.
[14] See the *Federal Register* of November 9, 1998 (63 FR 60353 at 60355).
[15] Department of Health Education and Welfare, Research Involving Children: Report and Recommendations of the National Commission for the Protection of Human Subjects of Biomedical and Behavioral Research. See the *Federal Register* of January 13, 1978 (43 FR 2084 at 2112).
[16] For information on reviewing the qualifications of investigators, see the guidance for IRBs, Clinical Investigators, and Sponsors on *IRB Responsibilities for Reviewing the Qualifications of Investigators, Adequacy of Research Sites, and the Determination of Whether an IND/IDE is Needed* (August 2013).
[17] See footnote 13.
[18] Additional recommendations related to risk are included in The Secretary's Advisory Committee on Human Research Protections (SACHRP): Appendix B: Recommendations regarding risk in research involving children, July 28, 2005. Available at https://www.hhs.gov/ohrp/sachrp-committee/recommendations/2005-july-28-letter-appendix-b/index.html (accessed September 19, 2022).
[19] Bhatnagar M, Sheehan S, Sharma I, Baer G, Green D, McCune S, Joffe S, Snyder D, 2021, Prospect of Direct Benefit in Pediatric Clinical Trials: Practical Challenges and Potential Solutions, Pediatrics, 147(5) e2020049602.

the research intervention or procedure in the clinical investigation in question (21 CFR 50.52). *Prospect of direct benefit* should result from the research intervention or procedure being studied (e.g., the investigational drug or medical device) and not from ancillary interventions or procedures, such as physical exams done as part of the trial. For research interventions or procedures that are considered to offer *prospect of direct benefit*, the IRB must find not only that the risk is justified by the anticipated benefit to the child, but the relation of the anticipated benefit to the risk is at least as favorable as any available alternatives (21 CFR 50.52). When evaluating if an intervention or procedure offers a *prospect of direct benefit,* the IRB should consider whether the evidence establishing proof of concept about a potential beneficial effect is sufficient, and whether the proposed dose (particularly for drugs) and duration of exposure to the intervention or procedure are adequate to offer a potential clinical benefit to the individual child. For a medical device clinical investigation, the device characteristics should be compatible with the child's age and developmental stage such that a benefit is anticipated.[20]

The necessary evidence to determine *prospect of direct benefit* for a pediatric clinical investigation may be based on one or more sources of information. When adult data are available in conditions that exist both in adults and children, evidence of clinical benefit from the drug or device in adults can provide support for *prospect of direct benefit* before clinical investigations are initiated in children. Animal or relevant device modeling and simulation data may provide evidence of *prospect of direct benefit*; and, in conditions that exist in both pediatric and adult populations, may preclude or mitigate the need to preliminarily collect relevant adult data. For pediatric conditions with a phenotype that extends into adulthood, demonstration of a drug's favorable effect on a biomarker(s) or surrogate endpoint(s) linked to the causal pathway of the disease in adults may also support *prospect of direct benefit* in children. For conditions with manifestations that occur exclusively in children, collection of adult data evaluating the drug or device may not be available or feasible, and nonclinical data obtained in a relevant animal or in vitro model for the condition of interest may often be the only source of information to support *prospect of direct benefit*.

See section IV for additional information regarding *prospect of direct benefit* related to the design of the clinical investigation and research related procedures.

D. Assessment of Risk for Interventions or Procedures with a Prospect of Direct Benefit

21 CFR 50.52(a) requires that the IRBs find that the risk is justified by the anticipated benefit to subjects. Assessment of the risk is predicated on adequate safety data. All available clinical safety data—such as data collected from healthy adults, if appropriate; adults with the same condition; or adults or children treated with the same drug or device for a different indication—should be included in the risk analysis. However, if such information is not available, as may be the case for pediatric conditions that present solely or primarily in childhood, safety information

[20] See the guidance for industry and FDA staff *Leveraging Existing Clinical Data for Extrapolation to Pediatric Uses of Medical Devices*.

may be limited to nonclinical studies,[21] which could include:

- Nonclinical studies to evaluate maximum tolerated doses or device performance and safety,
- Juvenile animal studies to support the pediatric age groups being studied, and/or
- Nonclinical studies of sufficient duration to support treatment for chronic conditions.

E. Component Analysis

A research protocol, including a protocol studying a pediatric condition, may, and usually does, include multiple research-related interventions or procedures, some that offer *prospect of direct benefit* and some that do not. Any intervention or procedure conducted solely for research purposes and not needed for clinical management or routine clinical care should be evaluated separately to determine whether it offers *prospect of direct benefit* to the enrolled child (known as a "component analysis" of risk).[22] If a specific intervention or procedure does not offer *prospect of direct benefit*, the risk of the intervention or procedure should be limited to a *minor increase over minimal risk,* and meet the other conditions outlined under 21 CFR 50.53 unless the protocol is referred for review, as per 21 CFR 50.54 (see section III.F).

Failure to carefully evaluate the different components of a clinical investigation may result in an intervention or procedure that does not offer *prospect of direct benefit* exceeding the allowable ceiling of a *minor increase over minimal risk*. For example, for children enrolled in the active study arm of a placebo-controlled clinical investigation, there is *prospect of direct benefit* offered by the investigational medical product. For children in the placebo arm, however, there is no *prospect of direct benefit* from the placebo intervention or procedure. Factors to consider when assessing risk to children in the placebo arm of the trial (evaluated under 21 CFR 50.51 as minimal risk or 21 CFR 50.53, as a *minor increase over minimal risk*) are:

- The placebo intervention (e.g., sugar pill, saline);
- Routes of administration (e.g., oral, infusion, topical) or procedures used for administration (e.g., placement of peripheral catheter);
- Frequency and duration of administration of the placebo;
- Risk of withholding known effective therapy, if such therapy exists and will be withheld; and
- Use of rescue therapy, if appropriate.[23]

---

[21] For information on the design and conduct of nonclinical studies, see guidance for industry *Investigational Enzyme Replacement Therapy Products: Nonclinical Assessment* (October 2019); guidance for industry *Nonclinical Safety Evaluation of Pediatric Drug Products* (February 2006); guidance for industry *Severely Debilitating or Life-Threatening Hematologic Disorders: Nonclinical Development of Pharmaceuticals* (March 2019); ICH guidance for industry *S9 Nonclinical Evaluation for Anticancer Pharmaceuticals* (March 2010); ICH guidance for industry *S9 Nonclinical Evaluation for Anticancer Pharmaceuticals Questions and Answers* (June 2018); ICH guidance for industry *S11 Nonclinical Safety Testing in Support of Development of Paediatric Medicines* (May 2021); and draft guidance for industry and FDA staff *General Considerations for Animal Studies for Medical Devices* (October 2015) (when final, this guidance will represent the FDA's current thinking on this topic).

[22] Final Rule, Additional Safeguards for Children in Clinical Investigations of Food and Drug Administration-Regulated Products, 78 FR 12937 at 12942 (February 26, 2013) and 43 FR 2084 at 2086 (January 13, 1978).

[23] Momper JD, DJ Green, K Park, GJ Burckart, DL Snyder, 2021, Ethical Considerations for Pediatric Placebo-Controlled Trials: FDA Outcomes and Perspectives. TIRS, 55(2): 282-303.

The risks associated with administration of a placebo in a clinical investigation should be part of the component analysis of risk. For example, if an intravenous catheter will be placed solely to administer placebo and is not needed for clinical management or is not needed for routine clinical care, the risk of the insertion and management of the catheter should be considered as part of the risk assessment. A peripheral intravenous catheter should generally be considered as minimal risk or a *minor increase over minimal risk*, whereas a central intravenous catheter should generally be considered to exceed the *minor increase over minimal risk* threshold.[24] Oral administration of a placebo for a short time period should generally be considered minimal risk. A placebo administered by a single injection could be considered minimal risk; it is possible that multiple injections or infusions could be considered as a *minor increase over minimal risk*, but in other circumstances multiple injections or infusions would exceed the *minor increase over minimal risk* threshold. If known effective therapy is withheld, the risk associated with withholding therapy should not exceed a *minor increase over minimal risk*. If withholding or withdrawing a known effective therapy may result in significant harm to the child, the risk may exceed the *minor increase over minimal risk* threshold, and the use of a placebo may not be justified.[25, 26] In some cases, placebo-controlled drug trials requiring injections or infusions administered over the course of one or two years have been justified as a *minor increase over minimal risk* depending on whether appropriate risk mitigation strategies are included as part of the protocol.[27]

F. Potential for Review per 21 CFR 50.54

If an intervention or a procedure in a pediatric protocol exceeds a *minor increase over minimal risk* and does not offer *prospect of direct benefit*, the protocol is not approvable by an IRB under 21 CFR 50.51, 50.52, or 50.53. FDA regulations include provisions under which a clinical investigation that is not otherwise approvable by an IRB may proceed if the following criteria are met[28]:

---

[24] As an example, in 2017 an IRB referred a protocol involving placebo administration via a central access venous device to FDA for review per 21 CFR 50.54. FDA consulted with its Pediatric Advisory Committee and Pediatric Ethics Subcommittee (PAC/PES). A summary of the deliberations of the PAC/PES, the recommendation from FDA's Office of Pediatric Therapeutics to the Deputy Commissioner for Medical Products and Tobacco, and the decision by the Deputy Commissioner is available at https://www.fda.gov/media/105555/download (accessed September 19, 2022).

[25] ICH guidance for industry *E10 Choice of Control Group and Related Issues in Clinical Trials* (May 2001), page 15.

[26] World Medical Association. (2013). Declaration of Helsinki: Ethical Principles for Medical Research Involving Human Subjects. First adopted in Helsinki, Finland, in 1964. Available at https://www.wma.net/policies-post/wma-declaration-of-helsinki-ethical-principles-for-medical-research-involving-human-subjects/ (accessed September 19, 2022).

[27] For examples, see Minutes from the May 11, 2018 joint meeting of the Pediatric Advisory Committee and the Endocrinologic and Metabolic Drugs Advisory Committee, regarding the use of randomized, blinded placebo-controlled trials for products intended for the treatment of achondroplasia, available at https://www.fda.gov/media/114640/download (accessed September 19, 2022); Meeting Minutes from the May 18, 2017 joint meeting of the Pediatric Advisory Committee and Pediatric Ethics Subcommittee, regarding a clinical investigation of a product intended to treat Duchenne Muscular Dystrophy, available at https://www.fda.gov/media/107320/download (accessed September 19, 2022); and footnote 23.

[28] For additional information see the guidance for clinical investigators, institutional review boards, and sponsors *Process for Handling Referrals to FDA Under 21 CFR 50.54 Additional Safeguards for Children in Clinical Investigations* (December 2006).

- The IRB finds that the research presents a reasonable opportunity to further the understanding, prevention, or alleviation of a problem affecting the health or welfare of children; and

- The Commissioner, after consultation with a panel of experts in pertinent disciplines (e.g., science, medicine, education, ethics, law) and following opportunity for public review and comment, determines either:

  – The research in fact satisfies 21 CFR 50.51, 50.52, or 50.53; or

  – The following three conditions described in 21 CFR 50.54 are met:

    1) The research presents a reasonable opportunity to further the understanding, prevention, or alleviation of a serious problem affecting the health or welfare of children;

    2) The research will be conducted in accordance with sound ethical principles; and

    3) Adequate provisions are made for soliciting the assent of children and the permission of their parents or guardians as set forth in 21 CFR 50.55.

G. Parental/Guardian Permission and Child Assent

A clinical investigator must obtain permission from the parent(s) or guardian(s) when a child is enrolled in a clinical investigation (21 CFR 50.55(e)).[29] The parental/guardian permission form must address the required elements of consent, as well as appropriate additional elements (see 21 CFR 50.25) to allow the parent(s) or guardian to make an informed decision. Informed consent is a process. Parents, guardians, and assenting children should be given the opportunity to ask questions when considering study participation, and continue to be provided information as the study progresses and as the situation requires.[30]

Assent means a child has provided affirmative agreement to participate in a clinical investigation; mere failure to object should not be construed as assent (21 CFR 50.3(n)). Unless the IRB waives the requirement, adequate provisions must be made for soliciting assent from the children if the IRB determines that the children are capable of providing assent (21 CFR

[29] FDA's regulations include limited exceptions from the general requirements for informed consent. See 21 CFR 50.23 and 50.24 and guidance for institutional review boards, clinical investigators, and sponsors *Exception from Informed Consent Requirements for Emergency Research* (April 2013). Of note, FDA does not intend to object to an IRB waiving or altering informed consent requirements for certain minimal risk clinical investigations involving children, as described in guidance for sponsors, investigators, and institutional review boards *IRB Waiver or Alteration of Informed Consent for Clinical Investigations Involving No More Than Minimal Risk to Human Subjects* (July 2017).
[30] For additional information on the informed consent process, see draft guidance for IRBs, clinical investigators, and sponsors *Informed Consent Information Sheet* (July 2014). When final, this guidance will represent the FDA's current thinking on this topic.

50.55(a)). Children 7 years of age and older are often considered capable of assent;[31] however, the age, maturity, and psychological state (mental capacity and developmental stage) of the child involved in the research must be considered (21 CFR 50.55(b)).

Assent of the children is not a necessary condition for a clinical investigation to proceed if the IRB finds either 1) that the children's capability is so limited they cannot reasonably be consulted or 2) that the intervention or procedure involved in the clinical investigation holds out a *prospect of direct benefit* that is important to the health or well-being of the children and is available only in the context of the clinical investigation (21 CFR 50.55(c)). Even if the IRB determines that the children are capable of assenting, assent may be waived under 21 CFR 50.55(d) if the IRB finds and documents that all the following criteria are met:

- The clinical investigation involves no more than minimal risk to the subjects;
- The waiver will not adversely affect the rights and welfare of the subjects;
- The clinical investigation could not practicably be carried out without the waiver; and
- Whenever appropriate, the subjects will be provided with additional pertinent information after participation.

Ultimately, the IRB determines whether assent is required and how assent is obtained.

IV. APPLICATION OF SUBPART D TO PEDIATRIC CLINICAL INVESTIGATIONS

IRBs should consider the following when determining if the regulatory criteria for clinical investigations involving children have been met.

A. Data to Support Conducting Pediatric Clinical Investigations

Multiple sources of information may be used to inform the design of an acceptable pediatric clinical investigation. Information from nonclinical studies, bench testing or modeling and simulation (especially in the case of devices), and literature may be used to assess the potential risks and benefits of initiating the investigation in children. Depending on the quality and applicability of these data, collection of relevant adult data prior to initiation of a trial in pediatric subjects may not always be necessary. If relevant adult data are available, those data may inform the trial design for pediatric subjects (see Principle of Scientific Necessity, section III.A). Early inclusion of children in medical product development or initiation of clinical trials directly in children may be appropriate.

In some cases, adult studies may not be ethical or feasible. For example, for a rare disease with high pediatric mortality, there may be few adults with the disease, or adults may have a less severe form with limited applicability to the more severe pediatric form.

[31] See the *Federal Register* of January 13, 1978 (43 FR 2084 at 2110).

B. Design Considerations for Clinical Investigations

Clinical investigations involving children should be designed to maximize the amount of information gained and minimize the number of subjects involved.

The risks posed by the drug or device in a pediatric clinical investigation may vary depending on the particular subgroup of pediatric patients. Factors to consider when designing a clinical investigation and assessing the potential risks to children involved in the study include the:

- Age and degree of physiological maturity of the child;
- Nature and natural history of the clinical condition to be treated;
- Current severity of the condition to be treated in the child;
- Presence of other complicating clinical conditions;
- Safety and effectiveness of the drug or device that may have been demonstrated in older subjects, or that is expected based on other clinical or nonclinical investigations; and
- Likely duration of drug or device use and its impact on the growth and development of the child, including behavioral and psychosocial effects.

The following sections provide additional design considerations for clinical investigations of drugs and clinical investigations of medical devices.

1. Clinical Investigations of Drugs

To offer a *prospect of direct benefit*, any dose planned for use in a pediatric clinical investigation should have the potential to have a therapeutic effect based on available scientific information.[32] If there are adults with the disease, pharmacokinetic (PK) and pharmacodynamic (PD) data in adults may provide useful information to help establish a potentially effective dose for use in children.

If there are a limited number of adults with the condition, PK and PD data from clinical trials in healthy adult volunteers[33] or in adults or children using the product for other indications may be informative in helping to establish initial dosing for children with the condition under study. Such information may also provide some evidence of drug activity to support *prospect of direct benefit* if the activity assessment is relevant to the pediatric population of interest. Extending a dose for a product from another patient population (or different indication) to the new pediatric population should be based on a sound scientific assessment, particularly addressing how the exposure-response for effectiveness and safety in the other population was used to predict the exposure-response relationship in the pediatric population of interest.

Nonclinical studies in disease-specific animal models of a pediatric condition or in vitro data could be used to support an initial pediatric dose if the PD effect on important aspects of the

---

[32] These considerations also apply to investigational gene therapies. For these trials, a single dose of vector is generally administered, with the possibility of a long-lasting duration of action and resulting benefit; the study dose will need to be in the potentially therapeutic range.
[33] Testing in adult normal, healthy volunteers is generally not acceptable for gene therapy trials. See the guidance for industry *Human Gene Therapy for Rare Diseases* (January 2020).

condition in question can be translated into an equivalent human dose that is anticipated to be effective and offers *prospect of direct benefit*. This should be based on scientific criteria that estimate the relationship between the PD effect in the nonclinical model and human physiology.

Clinical investigations should be of sufficient duration to offer a potential clinical benefit to the individual child. This judgement is similar to that made when exposing children to a treatment in clinical practice. Of note, most single-dose studies intended to collect PK data in children do not offer *prospect of direct benefit* because the study duration is too short to offer a clinical benefit. A study intended to collect single-dose PK data might be considered under 21 CFR 50.53 as a *minor increase over minimal risk* if there is adequate safety information to characterize the risk from exposure to the investigational drug and any additional study procedures as no more than a *minor increase over minimal risk*.[34, 35] In this case, the study intervention does not offer benefit but may contribute to generalizable knowledge about the child's disorder or condition.

Multiple-dose studies[36] intended to collect PK data may offer *prospect of direct benefit*, but the dose and duration of exposure to the study intervention should be sufficient to have the potential to result in a clinical benefit or to effect some change in a surrogate of clinical benefit. To provide studies of adequate duration to offer *prospect of direct benefit*, adaptive study designs should be considered when additional dose finding is required within the context of the clinical investigation. Such adaptive designs could combine prospectively planned dose ranging or dose titration with continued dosing after a dose is established.[37]

2. *Clinical Investigations of Medical Devices*

Compared to drugs, devices present different challenges due to the range of technology they incorporate and their varying applications. The available clinical data for the device (e.g., published studies and reports and actual use information) should be considered when designing the clinical trial to maximize the amount of information gained and minimize the number of subjects involved. For indications involving both adults and children, it may be possible to design a single pivotal study that includes both pediatric and adult subjects to reduce the burden of multiple studies and to optimize the sample sizes for both the pediatric and adult study populations. Further, while every effort should be made to gather data that adequately address each targeted pediatric subgroup for the proposed indication for use, in some cases, the expected benefit and safety can be determined without separate studies in each subgroup. That is, it may

[34] See the draft guidance for industry *General Clinical Pharmacology Considerations for Pediatric Studies of Drugs, Including Biological Products* (September 2022). When final, this guidance will represent the FDA's current thinking on this topic.
[35] Roth-Cline M, Nelson RM. Microdosing Studies in Children: A US Regulatory Perspective. Clinical Pharmacology and Therapeutics. 2015; 98(3): 232-233.
[36] Given the complexity of gene therapy products, multiple dose PK studies are unlikely to be conducted. Please contact CBER for additional considerations that may apply.
[37] Guidance for industry *Adaptive Designs for Clinical Trials of Drugs and Biologics* (November 2019).

be extrapolated from one age group to another.[38] In other cases, such as with neonates, clinical data gathered specifically in that subgroup will likely be needed.[39]

C. Study Procedures in Pediatric Clinical Investigations

In the context of a clinical investigation, procedures that are carried out as part of routine clinical care of a child generally are considered to offer a clinical benefit and do not require evaluation as a research intervention under the regulations. Procedures that are carried out solely for research purposes and do not offer *prospect of direct benefit* must meet the minimal risk criteria (21 CFR 50.51) or no more than a *minor increase over minimal risk* criteria (21 CFR 50.53) in order to be included in a clinical investigation unless referred for review under 21 CFR 50.54 (see section III.F).

The potential for harm and the invasiveness and frequency of the planned procedures should be considered when assessing the risk. A single lumbar puncture or a single muscle biopsy have been considered, in many circumstances, to constitute a *minor increase over minimal risk*.[40] However, large organ biopsies—such as liver or kidney biopsies—when done for research purposes only have generally been considered to exceed a *minor increase over minimal risk*, and should not be done in children unless the procedure is performed as part of the routine clinical care for that child in the treatment of their condition. When considering the risk of a procedure, the risk of any sedation not needed for the child's clinical care (i.e., non-therapeutic procedural sedation, see section IV.C.1) or the risk of use of a contrast agent should also be considered. For example, a single MRI without contrast could be considered minimal risk, but the addition of contrast or sedation to the procedure is likely to constitute at least a *minor increase over minimal risk*, depending on the type of contrast being used and the risk of the sedation.

1. Procedural Sedation in Pediatric Clinical Investigations

Procedures in children in a clinical trial may require sedation and the risks of sedation needed for non-beneficial "research-only" (non-therapeutic) procedures should be considered. The Pediatric Ethics Subcommittee of FDA's Pediatric Advisory Committee met in March 2015 to discuss the use of non-therapeutic procedural sedation and came to the following areas of agreement when considering the use of sedation for a non-therapeutic procedure:[41]

- Procedures should be performed at a high-volume center with a dedicated pediatric sedation service;

---

[38] See footnote 20.

[39] For additional information, see the guidance for industry and FDA staff *Premarket Assessment of Pediatric Medical Devices* (March 2014).

[40] Snyder D, Lee C, and Nelson R. (2018). Invasive Placebos, Patient Burdens and Community Advocacy: A Federal Ethics Panel Protocol Review. In Kodish, E and Nelson, R. (Eds). Ethics and Research with Children, A Case-Based Approach (2nd ed.). New York, NY: Oxford University Press.

[41] Minutes of the Pediatric Ethics Subcommittee of FDA's Pediatric Advisory Committee, March 23, 2015. http://wayback.archive-it.org/7993/20180127092544/https://www.fda.gov/downloads/AdvisoryCommittees/CommitteesMeetingMaterials/PediatricAdvisoryCommittee/UCM510177.pdf (accessed September 19, 2022).

- There should be rigorous scientific justification for the need for the non-therapeutic procedures;
- The approach to procedural sedation and risk minimization procedures should be described in the protocol;

- Children with chronic conditions that may place them at higher risk from procedural sedation should be carefully evaluated and potentially excluded from the protocol;

- The non-therapeutic procedure should be terminated if complications of sedation arise or the level of sedation is inadequate, as it would be inappropriate to escalate the approach to procedural sedation beyond what would be considered a *minor increase over minimal risk* rather than to stop the procedure;

- If a particular procedure in a particular patient population is normally accompanied by sedation when performed for clinical reasons, sedation should not be withheld in the non-therapeutic research setting to avoid its risks and thereby attempt to enhance the procedure's approvability under federal research regulations; and

- There should be clear communication with potential subjects (and their parents/guardians) regarding the non-therapeutic nature of the procedures and procedural sedation in child assent and parental permission documents.

FDA recommends that sponsors use these recommendations for minimizing risk in designing and implementing protocols that include non-therapeutic procedural sedation. These recommendations will also aid IRBs when assessing the risk to children. The IRB should consider the cumulative risk if more than one procedure with non-therapeutic procedural sedation is planned. If the IRB determines that the procedure(s) is integral to answering the scientific question and ethical to perform, but that it constitutes more than a *minor increase over minimal risk*, review under 21 CFR 50.54 will be required before the clinical investigation may proceed.

Inclusion of Older Adults in Cancer Clinical Trials
Guidance for Industry

U.S. Department of Health and Human Services
Food and Drug Administration
Oncology Center of Excellence (OCE)
Center for Drug Evaluation and Research (CDER)
Center for Biologics Evaluation and Research (CBER)

March 2022
Clinical/Medical

Inclusion of Older Adults in Cancer Clinical Trials
Guidance for Industry

Additional copies are available from:

Office of Communications, Division of Drug Information
Center for Drug Evaluation and Research
Food and Drug Administration
10001 New Hampshire Ave., Hillandale Bldg., 4th Floor
Silver Spring, MD 20993-0002
Phone: 855-543-3784 or 301-796-3400; Fax: 301-431-6353; Email: druginfo@fda.hhs.gov
https://www.fda.gov/drugs/guidance-compliance-regulatory-information/guidances-drugs

and/or

Office of Communication, Outreach, and Development
Center for Biologics Evaluation and Research
Food and Drug Administration
10903 New Hampshire Ave., Bldg. 71, rm. 3128
Silver Spring, MD 20993-0002
Phone: 800-835-4709 or 240-402-8010; Email: ocod@fda.hhs.gov
https://www.fda.gov/vaccines-blood-biologics/guidance-compliance-regulatory-information-biologics/biologics-guidances

U.S. Department of Health and Human Services
Food and Drug Administration
Oncology Center of Excellence (OCE)
Center for Drug Evaluation and Research (CDER)
Center for Biologics Evaluation and Research (CBER)

March 2022
Clinical/Medical

TABLE OF CONTENTS

I. INTRODUCTION 1
II. BACKGROUND 2
III. RECOMMENDATIONS 3
A. Early Clinical Development 4
B. Clinical Trials 4
C. Postmarket 6

Inclusion of Older Adults in Cancer Clinical Trials Guidance for Industry[1]

This guidance represents the current thinking of the Food and Drug Administration (FDA or Agency) on this topic. It does not establish any rights for any person and is not binding on FDA or the public. You can use an alternative approach if it satisfies the requirements of the applicable statutes and regulations. To discuss an alternative approach, contact the FDA office responsible for this guidance as listed on the title page.

I. INTRODUCTION

This guidance provides recommendations regarding the inclusion of older adult patients in clinical trials of drugs[2] for the treatment of cancer. For the purpose of this guidance, older adults are those 65 years of age and older. Specifically, this guidance includes recommendations for including an adequate representation of older adults in cancer clinical trials to better enable evaluation of the benefit-risk profile of cancer drugs in this population. Most cancer trials do not have an upper age limit for exclusion; however, adults 75 years of age and older are underrepresented in cancer clinical trials.[3] The guidance emphasizes the particular importance of including adults 75 years of age and older in cancer clinical trials. This guidance is intended to assist stakeholders, including sponsors and institutional review boards, responsible for the development and oversight of cancer clinical trials.

Enrolling an adequate representation of the range of patients in a clinical trial that may be exposed to a drug after approval is important. It provides the ability to understand the drug's benefit-risk profile across the patient population likely to use the drug in clinical practice (e.g., to identify whether there are differences in the benefits and/or risks of the drug in different populations). Including information in the labeling describing use in older adults may help promote the safe and effective use of these products in older adults and better inform treatment decisions in clinical practice.[4]

---

[1] This guidance has been prepared by the Oncology Center of Excellence (OCE), Center for Drug Evaluation and Research (CDER), and Center for Biologics Evaluation and Research (CBER) at the Food and Drug Administration.
[2] For the purposes of this guidance, references to drugs includes drugs approved under section 505 of the Federal Food, Drug, and Cosmetic Act (21 U.S.C. 355) and biological products licensed under section 351 of the Public Health Service Act (42 U.S.C. 262).
[3] Singh H, Kanapuru B, Smith C, et al., 2017, FDA Analysis of Enrollment of Older Adults in Clinical Trials for Cancer Drug Registration: A 10-Year Experience by the U.S. Food and Drug Administration, JCO, 35:15 suppl, 10009-10009.
[4] See the draft guidance for industry *Geriatric Information in Human Prescription Drug and Biological Product Labeling* (September 2020). When final this guidance, will represent the FDA's current thinking on this topic. For the most recent version of a guidance, check the FDA guidance web page at https://www.fda.gov/RegulatoryInformation/Guidances/default.htm.

The contents of this document do not have the force and effect of law and are not meant to bind the public in any way, unless specifically incorporated into a contract. This document is intended only to provide clarity to the public regarding existing requirements under the law. FDA guidance documents, including this guidance, should be viewed only as recommendations, unless specific regulatory or statutory requirements are cited. The use of the word should in Agency guidance means that something is suggested or recommended, but not required.

II. BACKGROUND

Adults 65 years of age and older, and especially those 75 years of age and older, are underrepresented in cancer clinical trials despite representing a growing segment of the population of cancer patients.[5,6] Therefore, obtaining more information is important to better inform treatment decisions for older adults with cancer. Cancer is a disease generally associated with age, with the number of cancer cases projected to multiply due to a rapidly aging U.S. population.[7] FDA is engaged with stakeholders to improve the representation of older adults in cancer trials.

The issue of older adults being underrepresented in clinical trials persists in oncology despite FDA's efforts to increase their inclusion in clinical trials. FDA has encouraged the inclusion of older adults in clinical trials, including through interaction with sponsors and through several guidance documents.[8,] In addition, FDA published a series of guidances that encourages sponsors to broaden cancer clinical trial eligibility criteria to enhance the generalizability of trial results and the ability to understand the drug's benefit-risk profile across the patient population likely to use the drug in clinical practice. One guidance in the series, *Cancer Clinical Trial Eligibility Criteria: Patients with Organ Dysfunction or Prior or Concurrent Malignancies* (July 2020) is particularly relevant to older adults. This guidance encourages the inclusion of patients with organ dysfunction and with prior or concurrent malignancies, as appropriate, to better reflect the population that will use the drug in clinical practice. It also includes specific recommendations regarding the inclusion of patients with renal, cardiac, and hepatic dysfunction and of patients with prior or concurrent malignancy, all of which may increase with age.

Differences may exist between younger and older adult patients in drug response and toxicity due to age-related physiologic changes. For example, the pharmacokinetics of the drug, or the pharmacodynamic response to the drug, or both may vary between younger and older adult

[5] Singh H, Kanapuru B, Smith C, et al., 2017, FDA Analysis of Enrollment of Older Adults in Clinical Trials for Cancer Drug Registration: A 10-Year Experience by the U.S. Food and Drug Administration, JCO, 35:15 suppl, 10009-10009.
[6] Smith BD, Smith GL, Hurria A, et al., 2009, Future of Cancer Incidence in the United States: Burdens Upon an Aging, Changing Nation, JCO, 27(17): 2758-65.
[7] Levit L, Singh H, Klepin H, Hurria A, 2018, Expanding the Evidence Base in Geriatric Oncology: Action Items from an FDA-ASCO Workshop, JNCI, 110(11): djy169.
[8] See the guidance for industry *Studies of Drugs Likely to be used in the Elderly* (November 1989), *E7 Studies in Support of Special Populations: Geriatrics* (August 1994) and *E7 Studies in Support of Special Populations: Geriatrics Questions and Answers* (March 2012) and *Enhancing the Diversity of Clinical Trial Populations – Eligibility Criteria, Enrollment Practices, and Trial Designs (November 2020).*

patients. In addition, older adults often have comorbidities and may be taking concomitant medications that could impact the efficacy of the cancer drug and may also impact the incidence and the severity and seriousness of an adverse reaction. It is important that the spectrum of older adults included in clinical trials are representative of the intended population, including those with physiological decline (e.g., frailty). Furthermore, there may be important differences in efficacy in older adult patients compared to the younger or general population, and information describing such differences should be conveyed to patients and healthcare providers where appropriate.

Geriatric (i.e., older adult) use information must be included in labeling, unless clearly inapplicable.[9] FDA's draft guidance for industry: *Geriatric Information in Human Prescription Drug and Biological Product Labeling* (September 2020)[10] assists applicants in determining the appropriate placement and content of geriatric information in labeling so that the information is clear and accessible to health care practitioners and includes content that guides the safe and effective use in geriatric patients. In addition, FDA's Drug Trials Snapshots[11] provides consumers with relevant information about the demographic profile of participants in key clinical trials that supported the original approval of new molecular entities and new biological products since 2015. Snapshots can also highlight differences, if applicable, in benefits and side effects among demographic groups, including, for example, differences based on age when a clinical trial includes a representative population of older adults. Demographic information may also be available on FDA's website within the posted product approval information.[12]

III. RECOMMENDATIONS

Clinical trials should include study populations reflecting the intended population that may receive the intervention being evaluated, if approved. In general, to achieve an unbiased estimate of treatment effect in the general population, sponsors should develop a strategy to enroll diverse populations, including different age groups, that are consistent with the intended use population. For most cancers, clinical trials should include a representative population of older adults.[13] Older adults, including those with physiological decline, should be enrolled in all phases of clinical trials when they can be safely and ethically enrolled.

Sponsors of cancer trials should consider the expected age range of their target population early in development. CDER and CBER are available to discuss plans for enrollment of older adults in cancer clinical trials, particularly when enrollment of adequate representation of older adults may be challenging.

---

[9] See 21 CFR sections 201.56(d)(4), 201.57(c)(9)(v), and 201.80(f)(10).
[10] When final, this guidance will reperesent the FDA's current thinking on this topic.
[11] Available at https://www.fda.gov/drugs/drug-approvals-and-databases/drug-trials-snapshots
[12] See Drugs@FDA.
[13] One source of data that may be considered when estimating the incidence of a cancer in older adults is the National Cancer Institute's Surveillance, Epidemiology, and End Results Program, SEER Incidence database, available at https://seer.cancer.gov/data/.

A strategy regarding inclusion of older adults should include all known information including for example, prevalence of the condition, diagnosis and treatment patterns, prior relevant studies, and differences in outcomes related to safety or efficacy. The guidance for industry *Enhancing the Diversity of Clinical Trial Populations – Eligibility Criteria, Enrollment Practices, and Trial Designs* (November 2020) includes recommendations for inclusive trial practices, trial design and methodological approaches, and other study design and conduct considerations for improving enrollment that sponsors should consider regarding older adults.

To understand potential age-related differences that may be relevant to the clinical development of a cancer drug, FDA recommends the following:

A. Early Clinical Development

- Sponsors should enroll older adults, if appropriate, in early phase studies to obtain information on safety, exposure, and response to better inform the study design and dose selection of later phase studies.

- Sponsors should evaluate drug interactions early in drug development to allow enrollment of older adults who may otherwise be excluded because of their concomitant medication use.

- Sponsors should document co-morbidities and make every effort to safely include these patients as well as those with organ dysfunction and prior/concurrent malignancies.[14]

B. Clinical Trials

- *Trial design*

  Sponsors should make every effort to enroll a representative population of older adults in their pivotal randomized trials. To facilitate the enrollment of older adults in cancer trials, sponsors may consider flexible approaches to trial design, such as age-based stratification or analyses based on hypothesized efficacy differences in older adults compared to the younger adults participants (≤65 years) to allow a focused benefit/risk assessment. If the pivotal trials are not able to enroll a representative sample of older adult patients, alternative trial designs should be proposed. This may include an open-label safety study that can enroll and analyze an older adult population separately in a parallel arm of a trial. Additional considerations for this particular trial design can be found in the guidance for industry *Placebos and Blinding in Randomized Controlled Cancer Trials for Drug and Biological Products* (August 2019). In some cases, the older adult arm(s) can be actively accruing at the time of new drug application (NDA) or biologics license application (BLA) or supplement submission.

[14] See guidance for industry *Cancer Clinical Trial Eligibility Criteria: Patients with Organ Dysfunction or Prior or Concurrent Malignancies* (July 2020).

An example of a possible trial design approach is a randomized controlled trial that enrolls younger and older adults and stratifies by age. The intent-to-treat (ITT) population consists of all enrolled patients, with the primary study hypothesis evaluating effectiveness in randomized patients under 75 years of age. Using a hierarchical testing strategy, after the primary analysis in patients under 75 years of age, subsequent analyses would be done in the ITT population to provide safety and efficacy information about all patients. If the size of the older patient population is adequate and powered to address the hypothesis, results in the older population can also be analyzed separately.

The design of the development program should reflect any important differences in a drug's benefit-risk balance in older adults compared to younger adults. In the design of the clinical development program, we recommend that sponsors consider perspectives of older adults, seeking input from patients and advocacy groups, as well as input from those caring for older adults such as clinicians and caregivers. This information can inform the design of the clinical trials, such as in assuring meaningful endpoints are selected, as well as in the conduct of the trial, such as in facilitating enrollment and retention of older adults.[15]

- *Develop recruitment strategies targeted to older adults*

  Clinical trials do not have an upper age limit for exclusion, however adults 75 years of age and older, continue to be underrepresented. FDA encourages sponsors and clinical trial cooperative groups to develop strategies to recruit patients that are reflective of the intended population. Possible challenges with recruiting older adults that could be mitigated, particularly among patients 75 years of age and older, include: location of clinical trial sites (e.g., sites in community-based settings may be more accessible to older adults than sites located in urban academic centers), format (e.g. digital) and content of informational material for the trial, caregiver support, accommodations needed for impairment (e.g., visual, mobility, etc.), and travel and other logistics. Where feasible, remote monitoring approaches should be considered.

  Sponsors should discuss specific goals for enrollment of older adults with clinical investigators and keep the clinical trial sites updated on the progress of enrolling older adults in the trial. Sponsors should discuss the importance of enrolling older adults during study training provided to the clinical sites. In addition, sponsors should consider getting input on trial design, trial conduct and recruitement strategies from geriatricians, geriatric oncologists, social and behavioral scientists with expertise in treating older adults. Additional input from patient advocates/navigators should also be sought.

- *Consider collecting additional information for older adults*

---

[15] See guidance for industry *Patient-Focused Drug Development: Collecting Comprehensive and Representative Input* (June 2020).

Sponsors should prospectively consider information that should be collected for older adults that will be clinically informative and will provide an understanding of clinical outcomes in older adults. For example, in addition to collection of age and performance status, elements from geriatric assessment tools (e.g. functional status, cognitive function), and a comprehensive assessment of comorbidities should be considered during trial design.[16] Incorporating a patient reported outcome instrument(s) in cancer trials may encourage older adults to participate in clinical trials and the information obtained may inform future research.[17]

- *Consider additional strategies in adverse event monitoring and management*

  Older adult patients' experience with adverse events may differ from younger patients. Developing strategies to capture and manage adverse events in older patients (e.g., supportive care measures, involvement of geriatric oncologists and other health care professionals with expertise in treating older adults) may facilitate older patients completing the trial.

- *Report more discrete age subgroups*

  Because outcomes may differ by increasing age group in patients 65 years of age and older, sponsors should identify further age subgroups to understand the drug's benefits and risks.[18] For example, subgroups such as age 65 years to 74 years of age and 75 years of age and older may be relevant. A particular need exists for information in patients 75 years of age and older. Sponsors may consider combining data across trials of similar design to ensure adequate representation of older adults across discrete age subgroups. Reporting clinical trial data from older adults in a more standardized and granular way can be more clinically useful[19]. FDA's guidance for industry *Integrated Summary of Effectiveness* (October 2015) includes recommendations regarding subpopulation assessment and reporting in the NDA or BLA that are applicable to subgroups of older adults in cancer trials (see section III.D of that guidance).

C. Postmarket

- Ideally, adequate information on older adults should be captured in the premarket clinical trials. However, if older adults are not adequately represented in pre-market clinical trials, it may be appropriate to develop a plan to collect data on older adults in the postmarket setting. This could be accomplished with post-marketing trials examining a broader population, or through collection of real

[16] Singh H, Beaver JA, Kim G, Pazdur R, 2016, Enrollment of Older Adults on Oncology Trials: An FDA Perspective, JGO, 8: 149-50.
[17] See the guidance for industry *Patient-Reported Outcome Measures: Use in Medical Product Development to Support Labeling Claims* (December 2009).
[18] See the guidance for industry *E7 Studies in Support of Special Populations: Geriatrics Questions and Answers* (March 2012).
[19] See footnote 16.

world data in an observational study or registry. In certain situations, FDA may require postmarket studies and clinical trials.[20] Sponsors should prospectively discuss their plan for collecting additional information in the postmarket setting with the CDER or CBER review division or office. Postmarket data may provide clinically useful information, that when appropriate, can be added to the *Geriatric Use* subsection of the labeling or other parts of the labeling.[21]

[20] See section 505(o)(3)(B) of the FD&C Act and draft guidance for industry *Postmarketing Studies and Clinical Trials-Implementation of 505(o)(3) of the Federal Food, Drug and Cosmetic Act* (October 2019). When final, this guidance will represent the FDA's current thinking on this topic

[21] See draft guidance for industry: *Geriatric Information in Human Prescription Drug and Biological Product Labeling* (September 2020). When final this guidance, will represent the FDA's current thinking on this topic.

Clinical Lactation Studies: Considerations for Study Design
Guidance for Industry

DRAFT GUIDANCE

This guidance document is being distributed for comment purposes only.

Comments and suggestions regarding this draft document should be submitted within 60 days of publication in the *Federal Register* of the notice announcing the availability of the draft guidance. Submit electronic comments to https://www.regulations.gov. Submit written comments to the Dockets Management Staff (HFA-305), Food and Drug Administration, 5630 Fishers Lane, Rm. 1061, Rockville, MD 20852. All comments should be identified with the docket number listed in the notice of availability that publishes in the *Federal Register*.

For questions regarding this draft document, contact (CDER) Jian Wang at 301-796-3846 or (CBER) the Office of Communication, Outreach, and Development at 800-835-4709 or 240-402-8010.

U.S. Department of Health and Human Services
Food and Drug Administration
Center for Drug Evaluation and Research (CDER)
Center for Biologics Evaluation and Research (CBER)

May 2019
Clinical/Medical

Clinical Lactation Studies: Considerations for Study Design

Guidance for Industry

Additional copies are available from:

Office of Communications, Division of Drug Information
Center for Drug Evaluation and Research
Food and Drug Administration
10001 New Hampshire Ave., Hillandale Bldg., 4th Floor
Silver Spring, MD 20993-0002
Phone: 855-543-3784 or 301-796-3400; Fax: 301-431-6353; Email: druginfo@fda.hhs.gov
https://www.fda.gov/Drugs/GuidanceComplianceRegulatoryInformation/Guidances/default.htm

and/or

Office of Communication, Outreach, and Development
Center for Biologics Evaluation and Research
Food and Drug Administration
10903 New Hampshire Ave., Bldg. 71, Room 3128
Silver Spring, MD 20993-0002
Phone: 800-835-4709 or 240-402-8010; Email: ocod@fda.hhs.gov
https://www.fda.gov/BiologicsBloodVaccines/GuidanceComplianceRegulatoryInformation/Guidances/default.htm

U.S. Department of Health and Human Services
Food and Drug Administration
Center for Drug Evaluation and Research (CDER)
Center for Biologics Evaluation and Research (CBER)

May 2019
Clinical/Medical

TABLE OF CONTENTS

I. INTRODUCTION....................1

II. BACKGROUND....................2

III. CONSIDERATIONS FOR CLINICAL LACTATION STUDIES....................2

A. Considerations for Conduct of a Clinical Lactation Study....................2

B. Ethical Considerations....................3

C. Study Design Considerations....................4

1. General Study Designs....................4
2. Other Study Design Considerations....................5
3. Study Subject Considerations....................5
4. Sample Size Considerations....................6

D. Milk Sampling Methods....................6

E. Measurement of Infant Milk Intake....................7

F. Pharmacokinetic Analysis....................8

G. Estimation of Infant Dosage....................8

H. Infant Safety Data Collection....................9

I. Data on Effect of Drug on Milk Production....................10

Clinical Lactation Studies: Considerations for Study Design Guidance for Industry[1]

This draft guidance, when finalized, will represent the current thinking of the Food and Drug Administration (FDA or Agency) on this topic. It does not establish any rights for any person and is not binding on FDA or the public. You can use an alternative approach if it satisfies the requirements of the applicable statutes and regulations. To discuss an alternative approach, contact the FDA staff responsible for this guidance as listed on the title page.

I. INTRODUCTION

This guidance provides recommendations for sponsors conducting clinical lactation studies. The Food and Drug Administration (FDA or Agency) has required lactation studies under section 505(o)(3) of the Food, Drug, and Cosmetic Act (FD&C Act) under some circumstances and is considering additional circumstances in which lactation studies may be required. In addition, sponsors in some circumstances may elect to conduct lactation studies absent a requirement or request from the Agency.

This guidance reflects FDA's current recommendations regarding pre- or post-marketing lactation studies by drug sponsors.[2] This guidance provides information to facilitate the conduct of lactation studies. Such studies can inform breastfeeding with drug use recommendations included in the *Lactation* subsection of labeling.

The recommendations in this guidance reflect discussions from the 2007 Pediatric Advisory Committee meeting[3] and the 2016 Lactation Workshop,[4] which considered how data from clinical lactation studies can inform the safety of a drug when used during lactation.[5] This draft guidance replaces the draft guidance for industry *Clinical Lactation Studies — Study Design, Data Analysis, and Recommendations for Labeling*, which published in February 2005.

---

[1] This guidance has been prepared by the Division of Pediatrics and Maternal Health in the Center for Drug Evaluation and Research in cooperation with the Center for Biologics Evaluation and Research at the Food and Drug Administration.

[2] For the purposes of this guidance, all references to *drugs* include both human drugs and therapeutic biological products unless otherwise specified.

[3] See https://wayback.archive-it.org/7993/20170403222238/https://www.fda.gov/ohrms/dockets/ac/oc07.htm#pac.

[4] See https://www.fda.gov/Drugs/NewsEvents/ucm486761.htm.

[5] Wang J, Johnson T, Sahin L, et al., 2017, Evaluation of the Safety of Drugs and Biological Products Used During Lactation: Workshop Summary, Clinical Pharmacol Ther, 101(6):736–744.

This guidance does not address specific lactation labeling recommendations. These topics are addressed in 21 CFR 201.57(c)(9)(ii) and the draft guidance for industry *Pregnancy, Lactation, and Reproductive Potential: Labeling for Human Prescription Drug and Biological Products — Content and Format* (December 2014).[6]

In general, FDA's guidance documents do not establish legally enforceable responsibilities. Instead, guidances describe the Agency's current thinking on a topic and should be viewed only as recommendations, unless specific regulatory or statutory requirements are cited. The use of the word *should* in Agency guidances means that something is suggested or recommended, but not required.

II. BACKGROUND

Despite significant efforts to improve the quantity and quality of information in labeling for drug use during lactation, there remains a paucity of human data. Therefore, lactating women and their health care providers often must make decisions about drug treatment and continuation of breastfeeding during therapy without quality human data in labeling. For that decision to be evidence based, lactating women and health care providers would need information including, at a minimum, the amount of drug in human milk, the effect of the drug on milk production, and an understanding of the risks posed by the drug on the breastfed infant based on expected levels of exposure and adverse drug event data.

Data from clinical lactation studies, along with other relevant data (e.g., drug physicochemical characteristics, mechanism of drug entry into breast milk, data from nonclinical studies, important infant factors) can be analyzed to evaluate the safety of a drug when used during lactation. The data can also be used to develop recommendations to minimize infant exposure, when appropriate.

III. CONSIDERATIONS FOR CLINICAL LACTATION STUDIES

A. Considerations for Conduct of a Clinical Lactation Study

FDA has required lactation studies under section 505(o)(3) of FD&C Act under some circumstances and is considering additional circumstances in which lactation studies may be required. In addition, sponsors in some circumstances may elect to conduct lactation studies absent a requirement or request from the Agency.

FDA encourages sponsors to consider conducting a clinical lactation study whenever such study would be appropriate, even if the study is not being required by the Agency. The following are situations when a sponsor may wish to consider whether conducting a clinical lactation study would be appropriate:

[6] When final, this guidance will represent the FDA's current thinking on this topic. For the most recent version of a guidance, check the FDA guidance web page at https://www.fda.gov/RegulatoryInformation/Guidances/default.htm.

- A drug under review for approval is expected to be used by women of reproductive age

- After approval, use of a drug in lactating women becomes evident (e.g., via reports in the medical literature or lay press)

- A new indication is being sought for an approved drug and there is evidence of use or anticipated use of the drug by lactating women

- Marketed medications that are commonly used by women of reproductive age (e.g., antidepressants, antihypertensives, anti-infectives, diabetic and pain medications)

These and other factors should be considered on a case-by-case basis.

B. Ethical Considerations

FDA-regulated clinical trials, including lactation studies, must conform to all applicable FDA regulations, including those related to human subject protections (21 CFR part 56, Institutional Review Boards, and 21 CFR part 50, Protection of Human Subjects (including subpart D, Additional Safeguards for Children in Clinical Investigations)). Sponsors should consider the following ethical considerations with respect to three populations of lactating women who may potentially participate in clinical lactation studies:[7]

1. Lactating women who are prescribed the drug, which is the subject of the lactation study, as part of standard clinical care

    - If a lactating woman was prescribed and is continuing to take a medically necessary drug, it is not necessary to stop the drug for the purposes of enrollment in a research setting. It would be ethically acceptable to enroll women who have already made a decision to take a medically necessary drug while breastfeeding and allow them to continue breastfeeding while taking the drug. The drug exposure, specifically, to the infant would be considered a clinical risk. Any risks associated with the research would still need to be described.

2. Women in a research setting who are administered an investigational drug

    - In a research setting, where a woman who is currently breastfeeding starts an investigational drug for a disorder or condition, breastfeeding must be discontinued for the duration of the study because the risks of the exposure to the drug in the breastfeeding infant may outweigh the benefits. The potential drug exposure of a breastfeeding infant must be considered a research risk (and offers no clinical benefit to the infant).

7 Wang J, Johnson T, Sahin L, et al., 2017, Evaluation of the Safety of Drugs and Biological Products Used During Lactation: Workshop Summary, Clin Pharmacol Ther, 101(6):736–744.

- It is acceptable to enroll breastfeeding women who are participating in a clinical trial of an investigational drug in clinical lactation studies if the breastfeeding woman agrees to temporarily pump and discard milk to avoid exposing an infant to the investigational drug. The length of time that the milk will need to be discarded should be specified in the protocol and will vary depending on factors such as the half-life of the drug.

3. Women who are healthy volunteers and are administered the investigational drug for the purpose of clinical research

    - In a research setting where a healthy woman who is currently breastfeeding volunteers for a clinical lactation study, breastfeeding must be discontinued for the duration of the study so that an infant is not exposed to the investigational drug.

C. Study Design Considerations

In considering the appropriate type of clinical lactation study to conduct, the sponsor should consider strategies that minimize the burden of data collection on the mother while obtaining adequate data. The study should avoid disruption of the breastfeeding routine and support return to breastfeeding if breastfeeding must be temporarily discontinued. Additionally, use of remote clinical study sites may provide access to a patient population that may not otherwise be willing or able to participate. Home health care nursing visits can be particularly important to successful recruitment and conduct of lactation studies of drugs with longer half-lives, when many visits occur over a period of several weeks.

1. General Study Designs

Sponsors should consider the following types of study designs for clinical lactation studies:

- Lactating woman (milk-only) study

    – A milk-only study can be used to detect the presence of a drug in breast milk, quantify or estimate the total amount of a drug transferred into breast milk (when plasma concentrations are known), and evaluate the effects of a drug on milk production (when milk production in lactating women not taking the drug is known). If the concentration of a drug in breast milk is found to be clinically relevant, this finding could lead to further studies.

    – In general, FDA recommends milk-only studies unless there is a reason to conduct another type of clinical lactation study.

- Lactating woman (milk and plasma) study

    – Milk and plasma collection in lactating women can provide pharmacokinetic (PK) data on a drug in a lactating woman, the amount of drug transferred into breast milk, and the effects of a drug on milk production. In certain situations, the PK data of the

drug may be unknown in lactating women such that obtaining such data would provide additional information in the amount of drug transferred into breast milk (e.g., when there is a concern for accumulation of a drug in breast milk).

- Mother-infant pair study

  – Mother-infant pair studies that include assessment of drug concentrations in infants can provide information on absorption of drugs in infants through breast milk and safety assessments in infants enrolled in these studies. A sponsor should consider this design if information is already available about the extent of drug transfer into breast milk including evidence that the drug accumulates in breast milk and if the drug is likely to be absorbed by the breastfed infant.

2. Other Study Design Considerations

In addition to the type of study design, sponsors should also consider the following study design issues:

- Single-dose design

  – For drugs that are given acutely (e.g., single-dose drug, drugs that do not accumulate with chronic dosing), a single-dose study may be sufficient.

- Longitudinal design

  – For drugs that are administered chronically or given for several treatment cycles, a sponsor may consider a longitudinal study design. Under such a design, samples are obtained from each lactating woman at different time points (e.g., at 2–3 months and then again at 5–6 months).

- Multiple-arm design

  – For drugs that are given acutely (e.g., single dose or short course of therapy), a multiple-arm study can be used to compare different lactating patients at different postpartum times. Under such a study, samples are obtained from different lactating women at different time points (e.g., at 2–3 months, 5–6 months).

3. Study Subject Considerations

The following maternal and infant factors can affect the results of a clinical lactation study. These factors should be collected in all lactation studies.

- Maternal factors

   - Maternal weight, age, gestational age at delivery, stage of lactation, length of time postpartum, smoking, alcohol intake, concomitant drugs, ethnicity, race, and existing medical conditions should be collected and reported for each study subject.

   - The study should specify subjects who exclusively breastfeed versus those who supplement with infant formula. Although FDA recommends that studies include only women who exclusively breastfeed, including women who are supplementing with infant formula provides *real life* data and may allow for easy collection of pumped milk that would otherwise be discarded. However, studies should report the extent of use of infant formula.

- Infant factors (for infants enrolled in mother-infant pair studies)

   - Age, weight, history of prematurity, drugs, existing medical conditions, ethnicity, and race should be collected and reported for each infant enrolled in a mother-infant pair study.

4. Sample Size Considerations

Sponsors should consider the following for sample sizes in clinical lactation studies:

- Sample size considerations include PK variability for the drug being studied, the study design (i.e., single dose versus multiple dose), and the variability in lactation physiology.

- A sponsor should consider the inter- and intra-subject variability for both mother and breastfed infant, depending on the design and primary objective of the study. For example, an increase to the sample size may be warranted if there is evidence of high inter- or intra-subject variability.

D. Milk Sampling Methods

For milk sampling during clinical lactation studies, sponsors should consider the following:

- Type of milk collected

   - The study design should specify the type of milk to be collected. For example, differences in composition of foremilk versus hindmilk should be accounted for with some drugs because transfer of drugs may be affected by the composition of the milk (e.g., foremilk contains more water and less fat which may affect the transfer of lipophilic drugs).

   - Sampling should ideally take place after the development of mature milk (after approximately 10 days postpartum). Colostrum or transitional milk collection may not reflect drug transfer in mature milk because drug transfer may be transiently increased because of a more porous mammary epithelium. However, sampling of colostrum or transitional milk may be important under certain circumstances. For

example, if concern exists about exposure of the drug in the immediate neonatal period, colostrum samples may be needed.

- The specific timing of the milk sample relative to both the dose and days postpartum should routinely be collected.

- Milk sampling method

  - In general, FDA recommends the collection of the entire milk volume from both breasts over 24 hours. Sampling should occur when drug exposure is at steady state during chronic maternal dosing. For drugs with dosing intervals of more than 24 hours, consideration should be made to collect milk over the entire dosing interval or to collect 24-hour samples during the expected time to peak plasma concentration. The sampling schedule should take into consideration a drug's known PK parameters and be adjusted for drugs with longer dosing intervals, balancing the need for adequate data collection with feasibility.

  - After the milk is collected, the necessary aliquots for assay should be saved using proper storage methods. The remainder of the milk collected can be refed to the infant under certain circumstances (see section III. B., Ethical Considerations). If the milk is allowed to be refed to the infant, the amount taken for assay should not deprive the infant of his or her nutritionally required volume.

  - FDA recommends the use of an electric pump rather than hand expression because electric pumps are more efficient in milk extraction. However, *hospital grade* pumps are not necessary; modern personal electric pumps utilize the same technology and are less costly.

E. Measurement of Infant Milk Intake

Sponsors should consider the following for measuring infant milk intake during clinical lactation studies:

- While a 150 mL/kg/day estimated milk intake is a reasonable assumption to estimate daily infant dosage, greater volumes do occur in early infancy and often correlate to the time of most reported infant adverse drug events. Additional consideration should be given to estimates of infant risk based on a 200 mL/kg/day milk intake in early infancy.

- Measurement of milk volume and weighing infants before and after feeding are methods that provide milk volume data for use in calculating infant exposure.

F. Pharmacokinetic Analysis

Analytical methods should be adequately validated, including both blood and breast milk, to address the accuracy, precision, selectivity, sensitivity, reproducibility, and stability of the parent drug and active metabolites of pharmacological importance.[8]

- Milk pharmacokinetics

  - The area under the milk concentration-time curve (AUC) should be calculated.

  - Average concentration should be based on AUC derived from collections at multiple time points, not just concentrations obtained at one sampling time.

  - Total milk concentration data should be used to estimate PK parameters of the parent drug and metabolites.

  - Peak and trough milk concentrations, as well as time to reach peak milk concentration, should be reported.

- Plasma pharmacokinetics (for milk and plasma study)

  - In general, plasma PK parameter estimates can include the following:

    - Area under the plasma concentration curve
    - Peak plasma concentration
    - Time to peak plasma concentration
    - Plasma clearance or apparent oral clearance
    - Apparent volume of distribution
    - Terminal half-life

  - PK parameters should be expressed in terms of total and unbound concentrations. For drugs and metabolites with a relatively low extent of plasma protein binding, FDA recommends that sponsors describe and analyze the pharmacokinetics in terms of total concentrations.

  - FDA also recommends noncompartmental and/or compartmental modeling approaches to parameter estimation.

G. Estimation of Infant Dosage

Sponsors should consider the following for calculating or estimating infant dosage:

[8] See the guidance for industry *Bioanalytical Method Validation* (May 2018).

- The daily infant dosage (total drug present in milk and consumed by the infant per day) should be calculated or estimated. Sponsors should consider the following to calculate daily infant dosage:

  Daily Infant Dosage (mg/day) = Σ (total drug concentration in each milk collection multiplied by the expressed milk volume in each milk collection)

  or

  Estimated Daily Infant Dosage (mg/kg/day) = M/P multiplied by the average maternal plasma concentration multiplied by 150 mL/kg/day

  M/P is the milk-plasma ratio. The calculation of M/P should be based on AUC and on multiple time points over 24 hours and not just a single point in time. Sponsors should consider an estimate of infant risk based on a 200 mL/kg/day infant milk intake in early infancy.

- The relative infant dose (the percent of the weight-adjusted maternal dosage consumed in breast milk over 24 hours) should be calculated. Sponsors should consider the following for relative infant dose:

  Relative Infant Dose = Infant Dosage (mg/kg/day)/Maternal Dosage (mg/kg/day) multiplied by 100

- If the drug has an approved indication for use in pediatric patients younger than 1 year of age, the estimated daily infant dosage should be compared to the approved dose. Calculation of the percentage of estimated daily infant dosage to the approved dose can provide an estimate of the risk to the infant.

- Infant pharmacokinetics (for a mother-infant pair study) should be considered. If infant drug concentration data are not collected, the average infant drug concentration ($C_{ss,ave}$) can be estimated by using the following formula:

  $C_{ss,ave}$ = *F multiplied by infant dosage/CL*

  F is the bioavailability, and CL is the drug clearance in the infant, if these data are known for the pediatric population.

H. Infant Safety Data Collection

An important component of clinical lactation studies is the collection of safety information in the breastfed infant. Follow-up examination or testing of the infant to evaluate for adverse drug events may be considered depending on the specific risk profile of the drug. Adverse drug event data can also be collected about the infant from mothers through surveys conducted electronically, by phone, or through maternal diaries.

I. Data on Effect of Drug on Milk Production

The clinical lactation studies described in this guidance are not formally designed to assess the effect of a drug on milk production. However, a sponsor should consider assessments about the effect of the drug on milk production in clinical lactation studies. For example, clinical lactation studies may include reports from enrolled women of any effects on milk production and, when feasible, a comparison of milk production before (or after discontinuation of) treatment to milk production during treatment.

E11(R1) Addendum: Clinical Investigation of Medicinal Products in the Pediatric Population

Guidance for Industry

U.S. Department of Health and Human Services
Food and Drug Administration
Center for Drug Evaluation and Research (CDER)
Center for Biologics Evaluation and Research (CBER)

April 2018
ICH

E11(R1) Addendum: Clinical Investigation of Medicinal Products in the Pediatric Population

Guidance for Industry

Additional copies are available from:

Office of Communications, Division of Drug Information
Center for Drug Evaluation and Research
Food and Drug Administration
10001 New Hampshire Ave., Hillandale Bldg., 4th Floor
Silver Spring, MD 20993-0002
Phone: 855-543-3784 or 301-796-3400; Fax: 301-431-6353
Email: druginfo@fda.hhs.gov
https://www.fda.gov/Drugs/GuidanceComplianceRegulatoryInformation/Guidances/default.htm

and/or

Office of Communication, Outreach and Development
Center for Biologics Evaluation and Research
Food and Drug Administration
10903 New Hampshire Ave., Bldg. 71, Room 3128
Silver Spring, MD 20993-0002
Phone: 800-835-4709 or 240-402-8010
Email: ocod@fda.hhs.gov
https://www.fda.gov/BiologicsBloodVaccines/GuidanceComplianceRegulatoryInformation/Guidances/default.htm

U.S. Department of Health and Human Services
Food and Drug Administration
Center for Drug Evaluation and Research (CDER)
Center for Biologics Evaluation and Research (CBER)

April 2018
ICH

TABLE OF CONTENTS

I. INTRODUCTION (1)... 1

A. Scope and Objective of the ICH E11 Guidance Addendum (R1) (1.1)................................ 1

II. ETHICAL CONSIDERATIONS (2)... 2

III. COMMONALITY OF SCIENTIFIC APPROACH FOR PEDIATRIC DRUG DEVELOPMENT PROGRAMS (3)... 3

IV. AGE CLASSIFICATION AND PEDIATRIC SUBGROUPS, INCLUDING NEONATES (4)... 4

V. APPROACHES TO OPTIMIZE PEDIATRIC DRUG DEVELOPMENT (5).......... 4

A. The Use of Existing Knowledge in Pediatric Drug Development (5.1)................................ 5

B. The Use of Extrapolation in Pediatric Drug Development (5.2).. 5

C. The Use of Modelling and Simulation in Pediatric Drug Development (5.3)........................ 6

VI. PRACTICALITIES IN THE DESIGN AND EXECUTION OF PEDIATRIC CLINICAL TRIALS (6)... 7

A. Feasibility (6.1)... 7

B. Outcome Assessments (6.2)... 8

C. Long-Term Clinical Aspects, Including Safety (6.3)... 8

VII. PEDIATRIC FORMULATIONS (7)... 8

A. Dosage and Administration (7.1)... 9

B. Excipients (7.2)... 9

C. Palatability and Acceptability (7.3)... 9

D. Neonates (7.4)... 10

GLOSSARY... 11

E11(R1) Addendum: Clinical Investigation of Medicinal Products in the Pediatric Population Guidance for Industry[1]

This guidance represents the current thinking of the Food and Drug Administration (FDA or Agency) on this topic. It does not establish any rights for any person and is not binding on FDA or the public. You can use an alternative approach if it satisfies the requirements of the applicable statutes and regulations. To discuss an alternative approach, contact the FDA office responsible for this guidance as listed on the title page.

I. INTRODUCTION (1)[2]

A. Scope and Objective of the ICH E11 Guidance Addendum (R1) (1.1)

Pediatric drug development has evolved since the original guidance *E11 Clinical Investigation of Medicinal Products in the Pediatric Population* (ICH E11 (2000))[3] published, requiring consideration of regulatory and scientific advances relevant to pediatric populations. This addendum does not alter the scope of the original guidance. ICH E11 (2000), including this addendum (R1); is not intended to be comprehensive; other ICH guidances, as well as documents from regulatory authorities worldwide, the World Health Organization (WHO), and pediatric societies, provide additional detail. The purpose of the addendum is to complement and provide clarification and current regulatory perspective on topics in pediatric drug development.

In general, FDA's guidance documents do not establish legally enforceable responsibilities. Instead, guidances describe the Agency's current thinking on a topic and should be viewed only as recommendations, unless specific regulatory or statutory requirements are cited. The use of the word should in Agency guidances means that something is suggested or

---

[1] This guidance was developed within the Expert Working Group (Multidisciplinary) of the International Council for Harmonisation of Technical Requirements for Pharmaceuticals for Human Use (ICH) (formerly the International Conference on Harmonisation of Technical Requirements for Registration of Pharmaceuticals for Human Use), and has been subject to consultation by the regulatory parties, in accordance with the ICH process. This document was endorsed by the ICH Steering Committee at Step 4 of the ICH process, August 2017. At *Step 4* of the process, the final draft is recommended for adoption to the regulatory bodies of the European Union, Japan, and the United States.

[2] This guidance finalizes the draft guidance *E11(R1) Addendum: Clinical Investigation of Medicinal Products in the Pediatric Population*, issued November 22, 2016 (81 FR 83847). Arabic numbers reflect the organizational breakdown in the document endorsed by the ICH Steering Committee at *Step 4* of the ICH process, August 2017.

[3] We update guidances periodically. To make sure you have the most recent version of a guidance, check the FDA Drugs guidance web page at https://www.fda.gov/Drugs/GuidanceComplianceRegulatoryInformation/Guidances/default.htm.

recommended, but not required, unless specific regulatory or statutory requirements are specified as advised by regulatory authorities worldwide.

In this addendum, section II (2) on ETHICAL CONSIDERATIONS, section IV (4) on AGE CLASSIFICATION AND PEDIATRIC SUBGROUPS INCLUDING NEONATES, and section VII (7) on PEDIATRIC FORMULATIONS, supplement the content in ICH E11 (2000). Section III (3) on COMMONALITY OF SCIENTIFIC APPROACH FOR PEDIATRIC DRUG DEVELOPMENT PROGRAMS addresses issues to aid scientific discussions at various stages of pediatric drug development in different regions. Section V (5) on APPROACHES TO OPTIMIZE PEDIATRIC DRUG DEVELOPMENT includes enhancement to the topic of *Extrapolation*, and introduces *Modelling and Simulation (M&S)*. These sections describe essential considerations intended to provide high-level guidance on the implementation of these important approaches in pediatric drug development, reflecting the evolving nature of these topics. This harmonized addendum will help to define the current recommendations and reduce the likelihood that substantial differences will exist among regions for the acceptance of data generated in pediatric global drug development programs and will ensure timely access to medicines for children.

II. ETHICAL CONSIDERATIONS (2)

ICH E11 (2000) section II.F (2.6) addresses relevant principles for the ethical conduct of pediatric studies, including the roles and responsibilities of the Institutional Review Board/Independent Ethics Committee (IRB/IEC), recruitment of study participants, parental (legal guardian) consent/permission and child assent, and minimization of risk and distress. These ethical principles are also defined in the current legal and regulatory framework of health authorities worldwide responsible for ensuring safeguards for the protection of children participating in research.

A fundamental principle in pediatric drug development requires that children should not be enrolled in a clinical study unless necessary to achieve an important pediatric public health need.[4] When clinical studies are used to obtain information relevant to the use of a medicinal product, such studies should be conducted in pediatric populations having the disease or condition for which the investigational product is intended, unless an exception is justified. Without a prospect of clinical benefit from an experimental intervention or procedure, the foreseeable risks to which a pediatric participant would be exposed must be low.[5] The burden of a procedure or an intended intervention should also be minimized. Experimental interventions or procedures that present greater than low risk should offer a sufficient prospect of clinical benefit to justify exposure of a pediatric population to such risk. Likewise, the balance of risk and anticipated clinical benefit should be at least comparable to the available alternative treatments.[6] There should be a reasonable expectation that a clinical benefit resulting from the clinical study can be made available to this population in the future.

The general principles of ethical considerations for parental (legal guardian) consent/permission and child assent are outlined in ICH E11 (2000) section II.F.3 (2.6.3) and continue to apply. Information regarding the clinical study and the process of parental (legal guardian) consent/permission and child assent should be provided to the parent (legal

[4] See 21 CFR 56.111.
[5] See 21 CFR 50.51 and 50.53.
[6] See 21 CFR 50.52.

guardian) and/or child participant, as appropriate, at the time of enrollment, especially relating to long-term studies or studies that may call for sample retention. When obtaining child assent, relevant elements of informed consent should be provided appropriate to the child's capability to understand. Lack or absence of expression of dissent or objection must not be interpreted as assent.[7] Over the course of a clinical study, it may be necessary to reassess the assent of a child in recognition of the child's evolving maturity and competency. During clinical studies, there may be a requirement for obtaining adequate informed consent from pediatric participants once a child reaches the age of legal consent. Local regulations related to confidentiality and privacy of pediatric participants should be followed.

Policies that promote clinical research transparency are also relevant in pediatric clinical research. A fundamental principle of drug development is the public availability of objective and unbiased clinical study results to enhance clinical research, to avoid unnecessary clinical trials especially in children, and to inform clinical decisions in pediatric practice.

III. COMMONALITY OF SCIENTIFIC APPROACH FOR PEDIATRIC DRUG DEVELOPMENT PROGRAMS (3)

General principles outlined in ICH E11 (2000) section I.D. (1.4) continue to apply. Pediatric drug development programs are increasingly multiregional. Multiregional pediatric drug development programs face specific challenges due to regional differences in pediatric regulatory requirements, operational practicalities, and cultural expectations. These regional differences in some instances limit the ability of health authorities to align regulatory processes. Thus, timely and efficient drug development calls for a common scientific approach for which the following key questions should be addressed:

(1) What is the medical need in one or more pediatric populations that the drug could address?
(2) Who are the appropriate pediatric populations or subgroups that could be considered?
(3) What objectives(s) for the pediatric development program could be considered?
(4) Based on the existing knowledge, including developmental physiology, disease pathophysiology, nonclinical data, data in adult or pediatric populations or subgroups, or data from related compounds, what are the knowledge gaps?
(5) Are there specific juvenile animal studies that should be conducted?
(6) What clinical studies and/or methodological approaches could be considered?
(7) What pediatric-specific clinical study design elements could be considered?
(8) Are there different formulations/dosage forms that should be used for specific pediatric subgroups, both to facilitate an optimal dose-finding strategy, and for treatment of pediatric patients in different subgroups?

A common scientific approach should consider input from stakeholders (e.g., clinicians, patients, experts from academia) and should be based on scientific advances and up-to-date knowledge.

Early consideration of pediatric populations during drug development planning, along with early interactions between drug developers and regulatory authorities worldwide, can

[7] See 21 CFR 50.3(n)

facilitate agreement on a common scientific approach. When differences are identified, established regulatory pathways to minimize the impact of these differences can be used. Therefore, a common scientific approach, not common regional requirements, is at the cornerstone of efficient pediatric drug development and timely delivery of safe and effective medicines for children.

IV. AGE CLASSIFICATION AND PEDIATRIC SUBGROUPS, INCLUDING NEONATES (4)

A rationale for the selection of the pediatric population to be included in clinical studies should be provided. Chronologic age alone may not serve as an adequate categorical determinant to define developmental subgroups in pediatric studies. Physiological development and maturity of organs, pathophysiology of disease or condition, and the pharmacology of the investigational product are factors to be considered in determining the subgroups in pediatric studies. Furthermore, the arbitrary division of pediatric subgroups by chronological age for some conditions may have no scientific basis and could unnecessarily delay development of medicines for children by limiting the population for study. Depending on the condition and treatment, it may be justifiable to include pediatric subpopulations in adult studies or adult subpopulations in pediatric studies.

Advances in medical care have led to better survival of high-risk newborn infants, especially preterm newborn infants, which makes drug development research in newborn infants or "neonates" increasingly important. Neonates include both term and preterm newborn infants. The neonatal period for term newborn infants is defined as birth plus 27 days. The neonatal period for preterm newborn infants is defined as beginning at birth and ending at the expected date of delivery plus 27 days. As the neonatal population represents a broad maturational range, the conditions that affect this population can vary considerably. A rationale for the selection of a neonatal population in clinical studies should be provided.

V. APPROACHES TO OPTIMIZE PEDIATRIC DRUG DEVELOPMENT (5)

The concepts presented in ICH E11 (2000) section II.D (2.4) still apply. The principles outlined in ICH E4, E5, E6, E9, and E10 should be consulted. The number of pediatric studies and knowledge in the field of pediatrics has increased since ICH E11 (2000). Respective regulations for pediatric drug development worldwide have also evolved. However, drug development in pediatrics continues to present challenges and opportunities. In some cases, there are difficulties with generating data across a pediatric population due to a variety of ethical considerations and feasibility issues. Alternative approaches may provide opportunities to address these issues when structured and integrated into the development program as per the principles outlined in this addendum. Early multidisciplinary dialogue regarding the acceptability of such approaches with regulatory authorities is recommended. The planning for development of the drug for children should not begin when development in adults reaches its conclusion.

A. The Use of Existing Knowledge in Pediatric Drug Development (5.1)

To better inform the design of a pediatric drug development program, there is an opportunity to use existing knowledge. Existing knowledge includes evidence already or concurrently generated with the drug that is under development in adult and pediatric populations with the same disease or condition. Existing knowledge also integrates nonclinical data, data about related compounds, disease pathophysiology, as well as consideration of the developmental physiology of the pediatric population or subgroup. Use of such information can optimize pediatric drug development programs without reducing evidentiary standards. Safety and risk consideration based on the existing knowledge should guide the decision whether specific mitigation, such as staggered enrollment based on age group, is necessary. However, any uncertainties related to the use of existing knowledge should be identified and managed prospectively. As data are generated through the drug development cycle, it is possible that the assumptions behind the parameters that have gone into the development strategy and methodology may need to be revisited to take new information into account. This new information will continue to inform the strategy and present an opportunity to further address uncertainties.

Additional approaches to optimize pediatric drug development may include, but are not limited to, statistical and pharmacometric methods, including M&S that integrate and leverage existing knowledge, as well as extrapolation of information from other populations (adults or pediatric subgroups).

B. The Use of Extrapolation in Pediatric Drug Development (5.2)

The concept of *extrapolation* is used in different ways in drug development. *Pediatric extrapolation* is defined as an approach to providing evidence in support of effective and safe use of drugs in the pediatric population when it can be assumed that the course of the disease and the expected response to a medicinal product would be sufficiently similar in the pediatric and reference (adult or other pediatric) population.

When a drug is studied in a pediatric population, consider all factors which may result in different drug responses, such as intrinsic (e.g., developmental) and extrinsic (e.g., geographic) factors that could have an impact on the extrapolation of data from one population to the other.

Where an extrapolation approach is scientifically justifiable, it should be a dynamic process that examines several factors. including disease pathogenesis; criteria for disease diagnosis and classification; measures of disease progression; and pathophysiological, histopathological, and pathobiological characteristics that support the assumptions of similarity of disease and similarity of response to therapy between the pediatric and the reference populations. A thorough understanding of the differences between pediatric and reference populations is critical relative to the pathophysiology of the disease; available biomarker/endpoints; organ systems physiology (i.e., renal, hepatic, central nervous system, skeletal, and immune systems), as well as clinical context of therapeutics; and pharmacological behavior of the drug.

Support for the assumptions of similarity of disease and response to therapy, including exposure-response relationship, and prediction of an effective dose for the intended population, may be derived from existing data, published literature, expert panels and consensus documents, or previous experience with other products in the same therapeutic

class. All data and information gathered can either confirm the extrapolation approach or inform how it might be improved. Ultimately, the exercise should identify whether there is sufficient data to support extrapolation, or if additional clinical information is called for.

When efficacy in the pediatric population can be extrapolated from data obtained in the reference populations, leveraging of safety data from the reference to the pediatric population may be used; however, additional pediatric safety data should usually be used, as data in adults may only provide some information about potential safety concerns related to the use of a drug in the pediatric population (ICH E11 (2000) section II.D (2.4)).

When extrapolation is considered in a pediatric drug development strategy, the following framework of questions should be discussed to assess what additional supportive data are important:

(1) What evidence supports a common pathophysiology of disease, natural history, and similarity of the disease course between the reference and pediatric population(s)?
(2) What is the strength of the evidence of efficacy in the reference populations?
(3) Is there a biomarker or surrogate endpoint in the reference populations that is relevant in the pediatric population?
(4) What evidence supports a similar exposure-response between the reference and intended populations?
(5) What uncertainties do the existing data (e.g., clinical or historical data and published literature) have, and what uncertainties about the pediatric population remain?
(6) If uncertainties remain, what additional information should be generated (e.g., information from M&S, animal, adult, pediatric subgroup studies) to inform the acceptability of the extrapolation approach?

As evidence builds, the acceptability of the proposed extrapolation approach should be reassessed, and it may be appropriate to change the extrapolation approach.

C. The Use of Modelling and Simulation in Pediatric Drug Development (5.3)

Advancement in clinical pharmacology and quantitative M&S techniques has enabled progress in using model-informed approaches (e.g., mathematical/statistical models and simulations based on physiology, pathology, and pharmacology) in drug development. M&S can help quantify available information and assist in defining the design of pediatric clinical studies and/or the dosing strategy. Considering the limited ability to collect data in the pediatric population, pediatric drug development requires tools to address knowledge gaps. M&S is one such tool that can help avoid unnecessary pediatric studies and help ensure appropriate data are generated from the smallest number of pediatric patients. The usefulness of M&S in pediatric drug development includes, but is not limited to, clinical trial simulation, dose selection, choice and optimization of study design, endpoint selection, and extrapolation. With M&S, quantitative mathematical models are built with all available and relevant sources of existing knowledge. Provided it is well conducted, M&S can inform on the pharmacokinetics, pharmacodynamics, efficacy, and safety of a drug.

The incorporation of M&S into pediatric drug development should be based on a strategic plan established through multidisciplinary discussions outlining objectives, methods, assumptions, deliverables, and timelines. When building a model, several criteria should be considered, including the intended use of the model itself, the quality and the extent of the existing data, and the assumptions made. Assumptions are usually structured around five

main areas: (1) clinical pharmacology (the compound and the patient), (2) physiology, (3) disease considerations, (4) existing data, and (5) the mathematical and statistical assumptions underpinning the model.

Complexity in M&S calls for a careful assessment of the impact of each of the above assumptions because the impact of each one can vary between populations. In pediatrics, it is particularly critical to consider the maturation of organ systems with the understanding that data from older subgroups may not necessarily be informative for the younger subgroups. Once assumptions are set, different scenarios should be defined to support the analysis of the impact of potential uncertainty in existing knowledge.

Emerging knowledge is incorporated into the model in an iterative approach to revisit and improve the model. A series of *learn and confirm* cycles should be used for model building and simulation/prediction, and be confirmed as soon as new information is generated. Using several models may be important to support a given pediatric drug development program, depending on the question(s) to be addressed, the confidence in the model, and the emerging data generated.

Risk assessment is a critical part of M&S. The clinical and statistical consequences of a specific approach should be discussed with experts to define the risks to be handled. The risks associated with accepting the M&S assumptions should accordingly be assessed and weighed against the confidence in the model predictions and the validity of the assumptions.

VI. PRACTICALITIES IN THE DESIGN AND EXECUTION OF PEDIATRIC CLINICAL TRIALS (6)

Before deciding which types of methodological approaches are to be used in clinical trial design and execution, one should consider several practical factors that influence the design and execution of pediatric clinical trials. Three key practical factors to consider are feasibility, outcome assessments, and long-term clinical aspects, including safety.

A. Feasibility (6.1)

Pediatric drug development faces unique feasibility issues, including a small number of eligible children for clinical research, limited pediatric specific resources at research centers, and the lack of dedicated pediatric trial networks. Consideration should be given to the available centers willing to participate that have access to eligible pediatric participants. When studying pediatric conditions, it may be important to consider implementing clinical trial operational strategies, including, but not limited to, the use of pediatric research coordinating centers, the development of master protocols for clinical trials planned and conducted in a collaborative manner to evaluate multiple therapies for the same disease or condition with a single control arm, and the enhancement of pediatric clinical research networks. These operational strategies may be challenging to implement, but may result in improved feasibility and increase timely and efficient pediatric drug development.

The expectations of children and their guardians, including the emotional and physical burden, and the convenience of participation, should be considered. Current standards of care can influence physician/patient treatment choices that may have an impact on pediatric clinical trial design. Strategies that foster input from children, their caregivers, and the

advocacy communities can facilitate participation, recruitment, and acceptability of a clinical study.

B. Outcome Assessments (6.2)

As stated in the ICH E11 (2000) section II.D.2 (2.4.2), it may be important to develop, validate, and employ different endpoints for specific age and developmental subgroups. The relevant endpoints and outcome measures for the pediatric population should be identified as early as possible. It is important to include protocol design features that allow pediatric participants at appropriate ages to contribute directly in these measures when possible. Where relevant, it may be prudent to assess potential pediatric endpoints in the adult development program.

C. Long-Term Clinical Aspects, Including Safety (6.3)

The concepts on safety presented in ICH E11 (2000) section II.D.3 (2.4.3) and section II.D.4 (2.4.4) still apply. It is acknowledged that rare events may not be identifiable in pre-registration development, and that pediatric-specific adverse events are unlikely to be detected in development programs that are limited in size and duration. Planned collection of safety data in nonclinical studies, adult clinical studies regardless of dose or indication, or data from other sources (e.g., M&S) should serve to improve the design of pediatric studies and pharmacovigilance activities to address specific pediatric safety concerns.

Long-term effects of drug treatment in children can include impacts on development, growth, and/or maturation of organ/system function. Therefore, adequate baseline assessments of growth/development and organ function, and regular follow-up measurements, should be planned. Early planning for follow-up in a development program offers the opportunity to systematically capture and evaluate long-term effects in a disease or condition, and increase data interpretability.

VII. PEDIATRIC FORMULATIONS (7)

Principal considerations for the development of age-appropriate pediatric formulations to allow for safe and accurate use of pediatric medicines as outlined in ICH E11 (2000) section II.B (2.2) continue to apply. Additional considerations for pediatric formulations to optimize efficacy and reduce the risk for medication and dosing errors should include age-appropriate dosage forms, ease of preparations and instructions for use for caregivers, acceptability (e.g., palatability, tablet size), choice and amount of excipients, delivery systems, and appropriate packaging.

Adult dosage forms are not always appropriate for use in the pediatric population, and if a preparation for adults is used, it may pose a safety risk. When pediatric considerations are not addressed early during the development process, the final medicinal product may call for such manipulation for use in children that it increases the likelihood for inaccurate dosing and changes in stability or bioavailability. Examples of this include multiple small-volume acquisitions from a vial designed for a single adult use, use of an opened adult capsule formulation or crushed tablets to administer a pediatric dose mixed with food, and broken tablets that do not have a score line. Therefore, planning for development of age-appropriate dosage forms for pediatric populations should be incorporated into the earliest stages of

product development. When manipulations of the available form are unavoidable, measures to minimize the impact on dose accuracy, stability and bioavailability must be addressed.

A. Dosage and Administration (7.1)

To achieve the targeted drug exposure, more than one dosage form of the active pharmaceutical ingredient (API) or its strength may be important to cover the range of pediatric populations intended to receive the medicinal product. For pediatric drugs, the environment where the product is likely to be administered should be considered when selecting the formulation for development. For example, long-acting formulations may be of importance in settings where the caregiver is not available (e.g., school, nursery). Furthermore, certain dosage forms that reduce the requirements for handling and storage may be more appropriate than others.

In developing a formulation for pediatric use, considerations should include the ease of accurate measurement and capability to deliver small volumes to minimize the risk for dosing error, especially in neonates, infants, and young children. Such approaches could include clearly marked administration devices designed for accurate measurement of the smallest dose volume and dose increments.

B. Excipients (7.2)

Excipients may lead to adverse reactions in children that are not observed (or not to the same extent) in adults. Thus, the use of excipients in pediatric medicines should take into account factors such as pediatric age group (e.g., term and preterm newborns related to their physiological development), frequency of dosing, and intended duration of treatment. The number of excipients and their quantity in a formulation should be kept to the minimum used to ensure product performance, stability, palatability, microbial control, and dose uniformity. Alternatives to excipients that pose a significant risk to children should always be considered, and the risk posed by the excipient weighed against the severity of the disease and availability of alternative treatments. When selecting excipients, one should always consider the potential impact on absorption and bioavailability of the active ingredient.

C. Palatability and Acceptability (7.3)

Orally administered pediatric medicines should be palatable to ensure dose acceptance and regimen adherence. A formulation strategy for developing palatable drugs includes minimizing/eliminating aversive attributes of the API and formulation of favorable flavor attributes. Taste masking is often used to improve the palatability of the medicine. As pediatric drug development can benefit global populations, the target for taste masking should not only be focused on ensuring a medicine does not taste unpleasant; it should also ensure that the taste has broad cultural acceptance.

Alternative dose administration strategies should be considered for pediatric populations who cannot be accommodated by the intended dosage form (e.g., segmenting or crushing tablets, coadministration with food or liquids). Appropriateness of the alternative strategy for a pediatric population, including patient and caregiver aspects (e.g., taste/palatability, ease and accuracy of manipulation, and potential changes in bioavailability due to a variety of factors) should be investigated before selection of the final market image formulation. Understanding real-world use behaviors in administering pediatric dosage forms and the mitigation of

associated risks will contribute to the development of a formulation that allows for safe dose administration.

D. Neonates (7.4)

Formulation requirements for neonates warrant special attention, such as its effects on electrolyte, fluid, or nutritional balance. Intramuscular injections should be avoided where possible and the tolerability of subcutaneous and intravenous injections evaluated. For neonates, environmental conditions (e.g., temperature, light) and equipment used for drug administration (e.g., enteral feeding tubes) may have an effect on drug delivery and bioavailability. When developing a parenteral dosage form, compatibility with other commonly administered parenteral medicines or parenteral nutrition should also be investigated, as intravenous access is often limited in this population.

GLOSSARY

Parental (legal guardian) consent/permission:
Expression of understanding and agreement by fully informed parent(s) or legal guardian to permit the investigator/sponsor of a clinical study to enroll a child in a clinical investigation. The choice of the terms *parental consent* or *parental permission* in different regions may reflect local legal/regulatory and ethical considerations.

Child assent:
The affirmative agreement of a child to participate in research or to undergo a medical intervention. Lack or absence of expression of dissent or objection should not be interpreted as assent.

Modelling and Simulation (M&S):
A range of quantitative approaches, including pharmacometrics/systems pharmacology and other mathematical/statistical approaches based on physiology, pathology and pharmacology to quantitatively characterize the interactions between a drug and an organic system that could predict quantitative outcomes of the drug and/or system's behavior in future experiments. In modelling and simulation, existing knowledge is often referred to as *prior* knowledge.

Pregnant Women: Scientific and Ethical Considerations for Inclusion in Clinical Trials
Guidance for Industry

DRAFT GUIDANCE

This guidance document is being distributed for comment purposes only.

Comments and suggestions regarding this draft document should be submitted within 60 days of publication in the *Federal Register* of the notice announcing the availability of the draft guidance. Submit electronic comments to https://www.regulations.gov. Submit written comments to the Dockets Management Staff (HFA-305), Food and Drug Administration, 5630 Fishers Lane, Rm. 1061, Rockville, MD 20852. All comments should be identified with the docket number listed in the notice of availability that publishes in the *Federal Register*.

For questions regarding this draft document, contact the Division of Pediatric and Maternal Health (CDER) at (301) 796-2200 or the Office of Communication, Outreach, and Development (CBER) at 800-835-4709 or 240-402-8010.

U.S. Department of Health and Human Services
Food and Drug Administration
Center for Drug Evaluation and Research (CDER)
Center for Biologics Evaluation and Research (CBER)

April 2018
Clinical/Medical
Revision 1

Pregnant Women: Scientific and Ethical Considerations for Inclusion in Clinical Trials
Guidance for Industry

Additional copies are available from:

Office of Communications, Division of Drug Information
Center for Drug Evaluation and Research
Food and Drug Administration
10001 New Hampshire Ave., Hillandale Bldg., 4th Floor
Silver Spring, MD 20993-0002
Phone: 855-543-3784 or 301-796-3400; Fax: 301-431-6353; Email: druginfo@fda.hhs.gov
https://www.fda.gov/Drugs/GuidanceComplianceRegulatoryInformation/Guidances/default.htm

and/or

Office of Communication, Outreach, and Development
Center for Biologics Evaluation and Research
Food and Drug Administration
10903 New Hampshire Ave., Bldg. 71, rm. 3128
Silver Spring, MD 20993-0002
Phone: 800-835-4709 or 240-402-8010; Email: ocod@fda.hhs.gov
http://www.fda.gov/BiologicsBloodVaccines/GuidanceComplianceRegulatoryInformation/Guidances/default.htm

U.S. Department of Health and Human Services
Food and Drug Administration
Center for Drug Evaluation and Research (CDER)
Center for Biologics Evaluation and Research (CBER)

April 2018
Clinical/Medical
Revision 1

TABLE OF CONTENTS

I. INTRODUCTION.......... 1

II. BACKGROUND 2

III. ETHICAL CONSIDERATIONS.......... 4

- **A. FDA Regulations That Govern Research in Pregnant Women.......... 4**
- **B. Research-Related Risks.......... 6**
- **C. General Guidelines for Including Pregnant Women in Clinical Trials 6**

IV. OTHER CONSIDERATIONS.......... 8

- **A. Disease Type and Availability of Therapeutic Options in the Pregnant Population 8**
- **B. Timing of Enrollment 9**
- **C. Pharmacokinetic Data 9**
- **D. Safety Data Collection and Monitoring 10**
- **E. Stopping a Clinical Trial That Enrolls Pregnant Women.......... 10**

REFERENCES.......... 11

Pregnant Women: Scientific and Ethical Considerations for Inclusion in Clinical Trials Guidance for Industry[1]

This draft guidance, when finalized, will represent the current thinking of the Food and Drug Administration (FDA or Agency) on this topic. It does not establish any rights for any person and is not binding on FDA or the public. You can use an alternative approach if it satisfies the requirements of the applicable statutes and regulations. To discuss an alternative approach, contact the FDA staff responsible for this guidance as listed on the title page.

I. INTRODUCTION

This guidance provides recommendations about how and when to include pregnant women in drug development clinical trials for drugs and biological products based on the Food and Drug Administration's (FDA's or Agency's) current thinking on this subject.[2] Specifically, this guidance supports an informed and balanced approach to gathering data on the use of drugs and biological products during pregnancy through judicious inclusion of pregnant women in clinical trials and careful attention to potential fetal risk. This draft guidance is intended to serve as a focus for continued discussions among various entities such as the Agency, pharmaceutical manufacturers, the academic community, institutional review boards (IRBs), and others who are involved with the conduct of clinical trials in pregnant women.[3]

This guidance discusses the scientific and ethical issues that should be addressed when considering the inclusion of pregnant women in drug development clinical trials. From a scientific and ethical standpoint, the population of pregnant women is complex based on the interdependency of maternal and fetal well-being, and the need to take into consideration the risks and benefits of a drug to both woman and fetus (American College of Obstetricians and Gynecologists 2015). The scientific and ethical issues discussed in this guidance apply both to clinical trials that enroll pregnant subjects and to clinical trials that allow enrolled subjects who become pregnant to remain in the trial.

---

[1] This guidance has been prepared by the Division of Pediatric and Maternal Health in the Center for Drug Evaluation and Research (CDER) in cooperation with the Center for Biologics Evaluation and Research and the Office of Good Clinical Practice, Office of Special Medical Programs, in the Office of the Commissioner at the Food and Drug Administration.

[2] Throughout this guidance, the term *drug* means drug and biological products regulated by CDER or CBER.

[3] In addition to consulting guidances, sponsors are encouraged to contact the appropriate review division to discuss specific issues that arise during drug development.

Some of the information provided in this guidance applies to drugs indicated to treat pregnancy-specific conditions (e.g., preterm labor, pre-eclampsia), but the larger focus is on drugs indicated for conditions that occur commonly among females of reproductive potential. Women in this group may require treatment for chronic disease or acute medical problems, and may become pregnant multiple times during the reproductive phase of their lives.

This guidance does not discuss general clinical trial design issues or statistical analysis. Those topics are addressed in the ICH guidances for industry *E9 Statistical Principles for Clinical Trials*, *E10 Choice of Control Group and Related Issues in Clinical Trials*,[4] and the draft ICH guidance for industry *E9(R1) Statistical Principles for Clinical Trials: Addendum: Estimands and Sensitivity Analysis in Clinical Trials*.[5] The draft guidance for industry *Pharmacokinetics in Pregnancy — Study Design, Data Analysis, and Impact on Dosing and Labeling*[6] and certain disease-specific and drug class-specific guidances may provide additional considerations for studying pregnant women during drug development.

In general, FDA's guidance documents do not establish legally enforceable responsibilities. Instead, guidances describe the Agency's current thinking on a topic and should be viewed only as recommendations, unless specific regulatory or statutory requirements are cited. The use of the word *should* in Agency guidances means that something is suggested or recommended, but not required.

II. BACKGROUND

In the interests of promoting maternal/fetal health and informed prescribing decisions during pregnancy, this guidance addresses the challenges of including pregnant women in drug development research. There are more than 60 million women in the United States between the ages of 15 and 44 years, and almost 4 million births per year (U.S. National Vital Statistics Reports). Like women who are not pregnant, some pregnant women need to use drugs to manage chronic disease conditions or treat acute medical problems. To the extent there is labeling information for pregnant women, it is usually based on nonclinical data with or without limited human safety data. The frequent lack of information based on clinical data often leaves the health care provider (HCP) and the patient reluctant to treat the underlying condition, which in some cases may result in more harm to the woman and the fetus than if she had been treated. In addition, pregnant women often use medically necessary drugs without a clear scientific understanding of the risks and benefits to themselves or their developing fetuses (Lyerly et al. 2008).

---

[4] We update guidances periodically. To make sure you have the most recent version of a guidance, check the FDA Drugs or Biologics guidance web page at https://www.fda.gov/Drugs/GuidanceComplianceRegulatoryInformation/Guidances/default.htm or https://www.fda.gov/BiologicsBloodVaccines/GuidanceComplianceRegulatoryInformation/default.htm.

[5] When final, this guidance will represent the FDA's current thinking on this topic.

[6] When final, this guidance will represent the FDA's current thinking on this topic.

Currently, information about drug use in pregnancy generally is collected in the postmarketing setting, using data from observational studies such as pregnancy exposure registries and other cohort studies, case control studies, and surveillance methods. Historically, there have been barriers to obtaining data from pregnant women in clinical trials in an effort to protect them and their fetuses from research-related risks. However, in certain situations, it may be helpful to collect data in pregnant women in the setting of a clinical trial (Goldkind et al. 2010). For example, it may be useful to compare the safety and efficacy of a drug that has been considered the standard of care for pregnant women with a newer treatment (Jones et al. 2010). In other situations, a woman's health and the well-being of her fetus may benefit from clinical trial participation. For example, a pregnant woman may need access to experimental therapies in a clinical trial setting because there are no approved treatment options available. Sometimes a drug treatment offered only through a clinical trial will hold out the prospect of direct benefit to the pregnant woman and/or her fetus beyond otherwise available therapies. For example, some clinical trials for drugs that treat human immunodeficiency virus (HIV), tuberculosis, and malaria enroll pregnant women (or provide that patients who become pregnant can continue enrollment) based on ethical principles and clinical need.

There are multiple reasons for considering the inclusion of pregnant women in clinical trials, including the following:

- Women need safe and effective treatment during pregnancy

- Failure to establish the dose/dosing regimen, safety, and efficacy of treatments during pregnancy may compromise the health of women and their fetuses

- In some settings, enrollment of pregnant women in clinical trials may offer the possibility of direct benefit to the woman and/or fetus that is unavailable outside the research setting

- Development of accessible treatment options for the pregnant population is a significant public health issue

Extensive physiological changes associated with pregnancy may alter drug pharmacokinetics and pharmacodynamics, which directly affects the safety and efficacy of a drug administered to a pregnant woman through alterations in drug absorption, distribution, metabolism, and excretion.[7] Pregnancy-related changes in various organ systems (e.g., gastrointestinal, cardiovascular, and renal) also may alter drug pharmacokinetics and pharmacodynamics. For example, a 30 to 40 percent increase in glomerular filtration rate results in much higher rates of clearance for some drugs during pregnancy (Mattison and Zajicek 2006); therefore, prescribing often occurs in the absence of knowledge regarding the dose required to achieve the desired therapeutic effect (Andrew et al. 2007).

---

[7] See the draft guidance for industry *Pharmacokinetics in Pregnancy — Study Design, Data Analysis, and Impact on Dosing and Labeling*.

Filling the knowledge gaps regarding safe and effective use of drugs in pregnant women is a critical public health need, but one that raises complex issues.

III. ETHICAL CONSIDERATIONS

The inclusion of pregnant women in clinical trials is guided by human subject protection regulations and involves complex risk-benefit assessments that vary depending on the seriousness of the disease, the availability of other treatments, the trial design, and whether the proposed investigation will occur in the premarketing or postmarketing setting. Because of the complex ethical issues involved in designing clinical trials that include pregnant women, sponsors should consider including an ethicist in planning their drug development programs. Moreover, sponsors should consider meeting with the appropriate FDA review division early in the development phase to discuss when and how to include pregnant women in the drug development plan. These discussions should involve FDA experts in bioethics and maternal health.

A. FDA Regulations That Govern Research in Pregnant Women

FDA-regulated clinical trials in pregnant women must conform to all applicable FDA regulations, including those related to human subject protections (21 CFR part 56, Institutional Review Boards, and 21 CFR part 50, subpart B, Informed Consent of Human Subjects). In addition, if the trial is supported or conducted by the Department of Health and Human Services (HHS), then 45 CFR part 46 may also apply, which would include subpart B, Additional Protections for Pregnant Women, Human Fetuses and Neonates Involved in Research.[8] The FDA regulations do not contain a section similar to 45 CFR part 46, subpart B; however, the FDA recommends that these requirements be satisfied for FDA-regulated clinical research. Subpart B requires that trials supported or conducted by HHS meet all of the following 10 conditions:

1. Where scientifically appropriate, nonclinical studies, including studies on pregnant animals, and clinical studies, including studies on nonpregnant women, have been conducted and provide data for assessing potential risks to pregnant women and fetuses;

2. The risk to the fetus is caused solely by interventions or procedures that hold out the prospect of direct benefit for the woman or the fetus; or, if there is no such prospect of benefit, the risk to the fetus is not greater than minimal[9] and the purpose of the research is the development of important biomedical knowledge which cannot be obtained by any other means;

3. Any risk is the least possible for achieving the objectives of the research;

---

[8] See 45 CFR 46.204.

[9] See section III.B., Research-Related Risks, for discussion of minimal risk.

4. The pregnant woman's consent is obtained in accord with the informed consent provisions of 45 CFR part 46, subpart A;

5. If the research holds out the prospect of direct benefit solely to the fetus then the consent of the pregnant woman and the father is obtained in accord with the informed consent provisions of 45 CFR part 46, subpart A, except that the father's consent need not be obtained if he is unable to consent because of unavailability, incompetence, or temporary incapacity or the pregnancy resulted from rape or incest;

6. Each individual providing consent is fully informed regarding the reasonably foreseeable impact of the research on the fetus or neonate;

7. For children as defined in § 46.402(a) who are pregnant, assent and permission are obtained in accord with the provisions of 45 CFR part 46, subpart D;

8. No inducements, monetary or otherwise, will be offered to terminate a pregnancy;

9. Individuals engaged in the research will have no part in any decisions as to the timing, method, or procedures used to terminate a pregnancy; and

10. Individuals engaged in the research will have no part in determining the viability of a neonate.

IRBs are required to possess the professional competence necessary to review the specific research activities that they oversee (21 CFR 56.107(a)). IRBs must include persons who are knowledgeable in areas about the acceptability of proposed research in terms of institutional commitments and regulations, applicable law, and standards of professional conduct and practice (21 CFR 56.107(a)). Therefore, if an IRB regularly reviews research involving pregnant women, the IRB must consider including one or more individuals who are knowledgeable about and experienced in working with such subjects (21 CFR 56.107(a)). When an IRB considers whether to approve a protocol involving pregnant women, it should consider only those risks and benefits (direct to the subjects, or generalizable knowledge) that may result from the research itself (as distinguished from risks and benefits of therapies that subjects would receive even if not participating in the research) (21 CFR 56.111(a)(2)). Additionally, IRBs are required to determine that additional safeguards are included in the trial to protect the rights and welfare of subjects who are pregnant (21 CFR 56.111(b)).

Additional issues are raised by pregnant minors. Depending on state law, a pregnant minor may be considered emancipated by virtue of her pregnancy, a mature minor, or still a child (see the definition of children under 21 CFR 50.3(o)). IRBs should be familiar with applicable law of the jurisdiction in which a trial will be conducted. In the event that a clinical trial regulated by the FDA allows the enrollment of pregnant minors, or a minor becomes pregnant while enrolled in a clinical trial, and the pregnant minor meets the definition of a child under applicable state law, the IRB would have to comply with the applicable requirements of 21 CFR part 50, subpart D, Additional Safeguards for Children in Clinical Investigations.

B. Research-Related Risks

Research-related risks may meet the regulatory definition for *minimal risk* or may involve greater than minimal risk. FDA regulations define minimal risk as follows (21 CFR 50.3(k)):

> "*Minimal risk* means that the probability and magnitude of harm or discomfort anticipated in the research are not greater in and of themselves than those ordinarily encountered in daily life or during the performance of routine physical or psychological examinations or tests."

Research-related risks are the risks specifically associated with the trial interventions or procedures. If a woman is assigned to receive a drug while enrolled in a clinical trial (i.e., the assignment of the drug is determined by the protocol), then the risks associated with the drug would be considered research-related.

In contrast, risks are not research-related when they are independent of the study and not associated with a trial intervention or protocol requirements. In other words, when a study collects data about drug treatment during pregnancy but the drug was prescribed before study enrollment by the patient's HCP, then the risks associated with the drug use are not research-related risks (Sheffield et al. 2014). For example, in a study in which the investigator plans to assess the pharmacokinetics of a particular selective serotonin reuptake inhibitor (SSRI) during pregnancy, the investigator enrolls pregnant women with a history of major depression who are currently managed on this drug. In this study the SSRI does not create research-related risk, because the patients are already using the SSRI (as previously prescribed by their HCPs) to manage their medical conditions. The only risks of the study are those associated with study-specific procedures (e.g., blood sample collection), and potential loss of confidentiality or privacy.

In this situation, the research-related risk to the fetus is minimal, and the purpose of the research is the development of important biomedical knowledge, which cannot be obtained by any other means. Some dedicated pharmacokinetic (PK) studies conducted with pregnant women (such as the previous SSRI example) can offer direct benefit to subjects if the data are used during the trial to adjust the dosing for individual subjects when clinically appropriate. The informed consent process should include discussion of expectations about whether trial data will be monitored and evaluated in a way that can potentially benefit the subject during the trial.

There may be circumstances in which a clinical trial can potentially expose a fetus to greater than minimal risk. Pregnant women can be enrolled in clinical trials that involve greater than minimal risk to the fetuses if the trials offer the potential for direct clinical benefit to the enrolled pregnant women and/or their fetuses. For example, this benefit may result from access to: (1) a needed but otherwise unavailable therapy (e.g., a new antituberculosis drug for multidrug resistant disease); or (2) a drug or biologic that reduces the risk for acquiring a serious health condition (e.g., a vaginal microbicide that reduces transmission of HIV and herpes simplex virus).

C. General Guidelines for Including Pregnant Women in Clinical Trials

This section provides general guidelines and considerations for including pregnant women in clinical trials. However, every drug development situation is unique, and individualized approaches to clinical trial design may be required to facilitate inclusion of pregnant women in specific drug development plans.

The FDA considers it ethically justifiable to include pregnant women with a disease or medical condition requiring treatment in clinical trials under the following circumstances:

<u>In the postmarketing setting (i.e., FDA-approved drugs)</u>

- Adequate nonclinical studies (including studies on pregnant animals) have been completed[10]

  and

- There is an established safety database in nonpregnant women from clinical trials or preliminary safety data from the medical literature and/or other sources regarding use in pregnant women

  and one of the following:

- Efficacy cannot be extrapolated

  and/or

- Safety cannot be assessed by other study methods

<u>In the premarketing setting (i.e., investigational drugs)</u>

- Adequate nonclinical studies (including studies on pregnant animals) have been completed

  and

- The clinical trial holds out the prospect of direct benefit to the pregnant woman and/or fetus that is not otherwise available outside the research setting or cannot be obtained by any other means (e.g., the pregnant woman may not have responded to other approved treatments or there may not be any treatment options)

The above conditions would also apply to a drug that is being developed to treat a pregnancy-specific condition.

---

[10] The phrase *adequate nonclinical studies* refers to recommendations for the design and conduct of reproductive toxicology and other nonclinical studies described in the ICH guidances for industry *M3(R2) Nonclinical Safety Studies for the Conduct of Human Clinical Trials and Marketing Authorization for Pharmaceuticals* and *S5(R2) Detection of Toxicity to Reproduction for Medicinal Products: Addendum on Toxicity to Male Fertility*.

<u>Women who become pregnant while enrolled in a clinical trial</u>

When a pregnancy has been identified during a clinical trial, unblinding should occur so that counseling may be offered based on whether the fetus has been exposed to the investigational drug, placebo, or control. The risks and benefits of continuing versus stopping investigational treatment can be reviewed with the pregnant woman. Pregnant women who choose to continue in the clinical trial should undergo a second informed consent process that reflects these additional risk-benefit considerations.

If fetal exposure has already occurred, a woman who becomes pregnant while enrolled in a clinical trial should be allowed to continue on the investigational drug if the potential benefits of continued treatment for the woman outweigh the risks of ongoing fetal exposure to the investigational drug, of discontinuing maternal therapy, and/or of exposing the fetus to additional drugs if placed on an alternative therapy. Regardless of whether the woman continues in the trial, it is important to collect and report the pregnancy outcome.

IV. OTHER CONSIDERATIONS

Including pregnant women in a trial involves careful risk-benefit assessments. All trials must be designed to minimize risk as much as possible while preserving the ability to achieve the objectives of the research (21 CFR 56.111). Some general considerations for sponsors and investigators include:

- Obtaining adequate reproductive and developmental toxicology data in relevant nonclinical models

- Identifying the trial population that will derive the most benefit while trying to minimize risk

- Considering the gestational timing of exposure to the investigational drug in relation to fetal development

- Choosing appropriate control populations

Sponsors should also consider the issues discussed in the following sections when designing a clinical trial that will include pregnant women.

A. Disease Type and Availability of Therapeutic Options in the Pregnant Population

Sponsors should take into account the incidence of the disease, the severity of the disease (e.g., whether or not it is life-threatening), and the availability of other therapeutic options and their risks. Pregnant patients with no other viable therapeutic options (e.g., drug resistance, drug

intolerance, contraindication, drug allergy) to treat a serious or life-threatening disease or condition may be appropriate candidates to enroll in a clinical trial.

B. Timing of Enrollment

The most appropriate time to include pregnant women in clinical trials during drug development may differ. Nonclinical reproductive and developmental toxicology studies generally should be completed before enrolling pregnant women in clinical trials.[11] In general, phase 1 and phase 2 clinical trials in a nonpregnant population that include females of reproductive potential should be completed before sponsors enroll pregnant women in later phase clinical trials. Sponsors should consider whether any of the following situations apply in determining when to enroll pregnant women in the drug development process.

- *If there are limited safety data or other approved (i.e., safe and effective) treatments are available*: In this situation, it may be more appropriate to complete phase 3 clinical trials in a nonpregnant population before enrolling pregnant women and exposing them to the investigational drug

- *If there are limited therapeutic options*: In these situations, the risk-benefit considerations may favor enrollment of pregnant women in earlier phase trials

- *If there are safety data for a drug that has been studied previously for other indications or populations*: In these situations, the risk-benefit considerations may favor enrollment of pregnant women in earlier phase trials

C. Pharmacokinetic Data

Because of the extensive physiological changes associated with pregnancy, PK parameters may change, sometimes enough to justify changes in dose or dosing regimen. For drug development programs where there are plans to enroll pregnant women in a phase 3 clinical trial, PK data in pregnant women should be collected during the phase 2 clinical trials to guide appropriate dosing in phase 3. In situations where pregnant women are enrolled in phase 3 clinical trials for a marketed drug, PK data should be collected as part of the trial.

In appropriate situations, nonpregnant women who become pregnant while on the investigational drug and consent to remain on the drug can also consent to PK assessments at steady state to collect data on correct dosing during pregnancy. Modeling and simulation have been increasingly used to support the design of clinical PK studies (Xia et al. 2013; Ke et al. 2013). For PK studies including pregnant patients, physiological changes during and after pregnancy that are critical for drug absorption and disposition may need to be considered in the model.

For additional information on PK modeling, study design considerations, and PK studies in pregnant women, refer to the draft guidance for industry *Pharmacokinetics in Pregnancy — Study Design, Data Analysis, and Impact on Dosing and Labeling*.

---

[11] See ICH M3(R2).

D. Safety Data Collection and Monitoring

When pregnant women are enrolled in a clinical trial, data collection elements should include, at a minimum: gestational age at enrollment; gestational timing and duration of drug exposure; and pregnancy outcomes including adverse maternal, fetal, and neonatal events. Enrolled pregnant patients should also receive obstetrical care that meets the recognized standards of care. Infants born to mothers who were exposed to the investigational drug should have follow-up safety information collected. Systemic drug exposure to the fetus/newborn can be evaluated by collecting cord blood or neonatal levels of drug and/or metabolites, depending on the timing of exposure to the drug and its half-life.

Clinical trials that enroll pregnant women should include investigators or consultants who have expertise in obstetrics and/or maternal/fetal medicine, depending on the underlying conditions treated by the investigational drug.

All clinical trials require monitoring (21 CFR 312.50 and 312.56), and no single approach to monitoring is appropriate or necessary for every clinical trial.[12] Clinical trials that involve pregnant women should include a data monitoring plan that includes members with relevant specialty and perinatal expertise to permit ongoing recognition and evaluation of safety concerns that arise during the course of the trial. This facilitates appropriate, expert assessment of adverse event reports.

E. Stopping a Clinical Trial That Enrolls Pregnant Women

There may be situations where it would be appropriate to stop a randomized, controlled clinical trial that is enrolling pregnant women. Examples include the following:

- An appropriately planned interim analysis demonstrates superior efficacy of the control or active comparator arm.

- There are documented serious maternal or fetal adverse events that can be reasonably attributed to drug exposure and are deemed to exceed the potential benefits of drug treatment. This determination should include consideration of alternative effective treatments and the risks of the underlying condition.

[12] See the guidance for clinical trial sponsors *Establishment and Operation of Clinical Trial Data Monitoring Committees* and the guidance for industry *Oversight of Clinical Investigations — A Risk-Based Approach to Monitoring*.

REFERENCES

American College of Obstetricians and Gynecologists, 2015, Ethical Considerations for Including Women as Research Participants, Committee Opinion No. 646, November.

Andrew, MA, TR Easterling, DB Carr, D Shen, ML Buchanan, T Rutherford, R Bennett, P Vicini, and MF Hebert, 2007, Amoxicillin Pharmacokinetics in Pregnant Women: Modeling and Simulations of Dosage Strategies, Clin Pharm and Therapeutics, 81(4):547–556.

Goldkind, S, L Sahin, and B Gallauresi, 2010, Enrolling Pregnant Women in Research — Lessons From the H1N1 Influenza Pandemic, N Engl J Med, Jun 17, 362(24):2241–2243.

Jones, H, K Kaltenbach, S Heil, S Stine, M Coyle, A Arria, K O'Grady, P Selby, P Martin, and G Fischer, 2010, Neonatal Abstinence Syndrome After Methadone or Buprenorphine Exposure, N Engl J Med, Dec 9, 363(24):2320–2331.

Ke, AB, SC Nallani, P Zhao, A Rostami-Hodjegan, and J Unadkat, 2013, Expansion of a PBPK Model to Predict Disposition in Pregnant Women of Drugs Cleared Via Multiple CYP Enzymes, Including CYP2B6, CYP2C9, and CYP2C19, BJCP, 77:3:554–570.

Lyerly, AD, MO Little, and R Faden, 2008, The Second Wave: Toward Responsible Inclusion of Pregnant Women in Research, Int J Feminist Approaches to Bioethics, Fall, 1(2):5–22.

Mattison, D and A Zajicek, 2006, Gaps in Knowledge in Treating Pregnant Women, Gend Med, Sep, 3(3):169–82.

Sheffield, JS, D Siegel, M Mirochnick, RP Heine, C Nguyen, K Bergman, RM Savic, J Long, KE Dooley, and M Nesin, 2014, Designing Drug Trials: Considerations for Pregnant Women, Clinical Infectious Diseases, 59(S7):S437–S444.

U.S. National Vital Statistics Reports Rapid Release, 2017, Births: Provisional Data for 2016, Report No. 002, June.

Xia, B, T Heimbach, R Gollen, C Nanavati, and H He, 2013, A Simplified PBPK Modeling Approach for Prediction of Pharmacokinetics of Four Primarily Renally Excreted and CYP3A Metabolized Compounds During Pregnancy, AAPSJ, 15(4):1012–1024.

Considerations for the Inclusion of Adolescent Patients in Adult Oncology Clinical Trials
Guidance for Industry

U.S. Department of Health and Human Services
Food and Drug Administration
Center for Drug Evaluation and Research (CDER)
Center for Biologics Evaluation and Research (CBER)
Oncology Center of Excellence (OCE)

March 2019
Clinical/Medical

Considerations for the Inclusion of Adolescent Patients in Adult Oncology Clinical Trials
Guidance for Industry

Additional copies are available from:

Office of Communications, Division of Drug Information
Center for Drug Evaluation and Research
Food and Drug Administration
10001 New Hampshire Ave., Hillandale Bldg., 4th Floor
Silver Spring, MD 20993-0002
Phone: 855-543-3784 or 301-796-3400; Fax: 301-431-6353; Email: druginfo@fda.hhs.gov
https://www.fda.gov/Drugs/GuidanceComplianceRegulatoryInformation/Guidances/default.htm

and/or

Office of Communication, Outreach, and Development
Center for Biologics Evaluation and Research
Food and Drug Administration
10903 New Hampshire Ave., Bldg. 71, Room 3128
Silver Spring, MD 20993-0002
Phone: 800-835-4709 or 240-402-8010; Email: ocod@fda.hhs.gov
https://www.fda.gov/BiologicsBloodVaccines/GuidanceComplianceRegulatoryInformation/Guidances/default.htm

U.S. Department of Health and Human Services
Food and Drug Administration
Center for Drug Evaluation and Research (CDER)
Center for Biologics Evaluation and Research (CBER)
Oncology Center of Excellence (OCE)

March 2019
Clinical/Medical

TABLE OF CONTENTS

I. INTRODUCTION 1
II. BACKGROUND 2
III. CRITERIA FOR INCLUDING ADOLESCENT PATIENTS IN ADULT ONCOLOGY CLINICAL TRIALS 2
IV. DOSE SELECTION FOR ADOLESCENT PATIENTS IN ADULT ONCOLOGY CLINICAL TRIALS 3
V. SAFETY MONITORING 4
VI. ETHICAL CONSIDERATIONS 4

Considerations for the Inclusion of Adolescent Patients in Adult Oncology Clinical Trials Guidance for Industry[1]

This guidance represents the current thinking of the Food and Drug Administration (FDA or Agency) on this topic. It does not establish any rights for any person and is not binding on FDA or the public. You can use an alternative approach if it satisfies the requirements of the applicable statutes and regulations. To discuss an alternative approach, contact the FDA staff responsible for this guidance as listed on the title page.

I. INTRODUCTION

The purpose of this guidance is to provide the pharmaceutical industry, clinical investigators, and institutional review boards with information to facilitate the inclusion of adolescent patients (for purposes of this guidance, defined as ages 12 to 17) in relevant adult oncology clinical trials. FDA recommends the inclusion of adolescent patients in disease- and target-appropriate adult oncology clinical trials to enable earlier access to investigational and approved drugs[2] for adolescent patients with cancer. Topics that are discussed in this guidance include the following:

- Appropriate criteria for the inclusion of adolescent patients in adult oncology clinical trials at various stages of drug development

- Dosing and pharmacokinetic and pharmacodynamic evaluations

- Safety monitoring

- Ethical considerations

The information in this guidance is meant to serve as a general guideline for sponsors considering this approach. Because specific details of an adult oncology drug development program that includes adolescent patients will vary depending on the characteristics and development stage of the drug and disease(s) under evaluation, sponsors are encouraged to

[1] This guidance has been prepared by the Divisions of Hematology and Oncology Products and Clinical Pharmacology V in the Center for Drug Evaluation and Research and the Oncology Center of Excellence (OCE) in cooperation with the Center for Biologics Evaluation and Research (CBER) at the Food and Drug Administration.

[2] For purposes of this guidance, references to *drugs* includes drugs and biological products approved under section 505 of the Federal Food, Drug, and Cosmetic Act (21 U.S.C. 355) and biological products licensed under section 351 of the Public Health Service Act (42 U.S.C. 262) that are drugs.

contact the responsible FDA review division to discuss details of the program before implementation.

In addition, enrolling adolescent patients in adult oncology clinical trials may contribute toward addressing pediatric regulatory requirements under section 505A or 505B of the Federal Food, Drug, and Cosmetic Act. Details of these requirements should be discussed with the responsible FDA review division.

In general, FDA's guidance documents do not establish legally enforceable responsibilities. Instead, guidances describe the Agency's current thinking on a topic and should be viewed only as recommendations, unless specific regulatory or statutory requirements are cited. The use of the word *should* in Agency guidances means that something is suggested or recommended, but not required.

II. BACKGROUND

Cancers in young pediatric patients are often different from those in adult patients and require unique treatment approaches; however, some cancers found in adolescent patients—such as some soft tissue and bone sarcomas, central nervous system tumors, leukemias and lymphomas, and melanoma—are similar in histology and biologic behavior to those found in adults. Adolescent patients, because of their age, have historically been ineligible for enrollment in adult oncology clinical trials, and the initial pediatric trials for many drugs are conducted years later, often after the drug is approved in adults. As a result, adolescent patients may have delayed access to potentially effective therapies. In addition, accrual of adolescent patients to pediatric trials evaluating approved drugs may be difficult because patients can receive the drug through off-label use.

III. CRITERIA FOR INCLUDING ADOLESCENT PATIENTS IN ADULT ONCOLOGY CLINICAL TRIALS

Adolescent patients should be eligible for enrollment in adult oncology clinical trials at all stages of drug development when the histology and biologic behavior of the cancer under investigation is the same in, or the molecular target of the drug is relevant to, cancers in both adult and adolescent patients.

The following are recommendations regarding including adolescent patients by stage of drug development:

- First-in-human or dose-escalation trials:
  - Adolescent patients may be enrolled after some initial adult pharmacokinetic and toxicity data are obtained. The sponsor should consult with the responsible FDA review division to determine the amount and type of adult data needed before enrolling adolescent patients.

  - In general, adolescent patients enrolled in these early phase trials should have cancers that are relapsed after or refractory to standard therapy with no curative options or for which no standard therapies with curative intent exist.

- Activity-estimating or confirmatory trials:

  - Adolescent patients can be enrolled simultaneously with adults

IV. DOSE SELECTION FOR ADOLESCENT PATIENTS IN ADULT ONCOLOGY CLINICAL TRIALS

Systemic exposure and clearance of drugs are generally similar in adolescent and adult patients after accounting for the effect of body size on pharmacokinetics. Selection of an appropriate dose for adolescent patients in clinical trials should be based on pharmacokinetic and/or pharmacodynamic characteristics of the investigational drug with consideration of body size effect on drug exposure, toxicity, and activity data (if available); the therapeutic index of the drug; and dose- and exposure-response relationships in adults.[3]

The following are recommendations for dosing based on how the drug is dosed in adults:

- For drugs with **body size-adjusted dosing** for adults, adolescent patients should receive the same body size-adjusted dose (mg/kg or mg/m^2) that is administered in adults.

- For drugs administered as a **fixed dose** based on data showing no clinically meaningful body size effect on drug exposure and toxicity in adults, a minimum body weight threshold should be defined to prevent adolescent patients who have a lower body weight than average from exceeding adult exposures.

  - An FDA analysis of adult population pharmacokinetics of oncology drugs suggested that 40 kg (the approximate median body weight of a 12-year-old[4]) is generally the lower end of the body weight range that has no clinically relevant effect on drug pharmacokinetics or safety. (This cutoff may change based on the characteristics of the drug, including the effect of body size on pharmacokinetics, the therapeutic index, and dose- and exposure-response relationships.)

  - In general, adolescent patients who weigh at least 40 kg can receive the same fixed dose administered in adults.

---

[3] Selection of an appropriate dose for adolescent patients may be more complex for certain biological products that are regulated by CBER. Sponsors of such products should consult with the relevant review division in CBER to determine if there are specific considerations they should take into account with respect to their products.

[4] See the Clinical Growth Charts web page under National Center for Health Statistics at the Centers for Disease Control and Prevention website (https://www.cdc.gov/growthcharts/clinical_charts.htm).

  - In general, adolescent patients who weigh less than 40 kg should switch to a body weight (mg/kg) or body surface area (mg/m^2) adjusted dose. This adjusted dose should be based on an adult reference body size (e.g., the average adult body weight of 70 kg or median body weight or surface area of the adult patient population determined from existing data).

- Pharmacokinetic and/or pharmacodynamic (if available) samples should be collected from adolescent patients included in the adult oncology drug development program.

V. SAFETY MONITORING

Safety data collected during the trial should be examined for any age-related differences.

The evaluation of developmental toxicities (e.g., growth derangements, fertility issues) that require a long duration of follow-up may not be possible in the context of early phase trials; however, the sponsor should develop a plan for longitudinal evaluation of potential developmental toxicities when it is feasible, particularly in trials enrolling patients in earlier lines of therapy.

Adolescent patients enrolled in adult oncology clinical trials should have access to appropriate care providers and facilities necessary to address the clinical management of potentially unique toxicities in this patient population, which may require pediatric oncology expertise.

Juvenile animal studies are not routinely needed before the enrollment of adolescent patients in adult oncology clinical trials, unless clinical and/or nonclinical data do not provide sufficient information on toxicities.[5]

VI. ETHICAL CONSIDERATIONS

Under 21 CFR 50.50, institutional review boards reviewing adult oncology clinical trials that allow for the enrollment of adolescent patients must ensure that the provisions of 21 CFR part 50, subpart D, Additional Safeguards for Children in Clinical Investigations, and, specifically, 21 CFR 50.52, Clinical investigations involving greater than minimal risk but presenting the prospect of direct benefit to individual subjects, are satisfied before approving the studies. Considerations should include disease and/or molecular target, available therapeutic options, and dose level for first-in-human trials.

Enrollment of appropriately selected adolescent patients in relevant adult oncology clinical trials with appropriate dose considerations and adequate safety monitoring is justified given the severe and life-threatening nature of their disease.

---

[5] Leighton, JK, Saber H, Reaman G, and Pazdur R, 2016, An FDA Oncology View of Juvenile Animal Studies in Support of Initial Pediatric Trials for Anticancer Drugs, Regul Toxicol Pharmacol, Aug; 79:142–143.

WARNINGS AND PRECAUTIONS

Guidance for Industry

Warnings and Precautions, Contraindications, and Boxed Warning Sections of Labeling for Human Prescription Drug and Biological Products — Content and Format

U.S. Department of Health and Human Services
Food and Drug Administration
Center for Drug Evaluation and Research (CDER)
Center for Biologics Evaluation and Research (CBER)

October 2011
Labeling

Guidance for Industry

Warnings and Precautions, Contraindications, and Boxed Warning Sections of Labeling for Human Prescription Drug and Biological Products — Content and Format

Additional copies are available from:

Office of Communications
Division of Drug Information
10001 New Hampshire Ave.
Silver Spring, MD 20993
Phone: 301-796-3400; Fax: 301-847-8714
druginfo@fda.hhs.gov
http://www.fda.gov/Drugs/GuidanceComplianceRegulatoryInformation/Guidances/default.htm

or

Office of Communication, Outreach and
Development, HFM-40
Center for Biologics Evaluation and Research
Food and Drug Administration
1401 Rockville Pike, Rockville, MD 20852-1448
http://www.fda.gov/BiologicsBloodVaccines/GuidanceComplianceRegulatoryInformation/Guidances/default.htm
(Tel) 800-835-4709 or 301-827-1800

U.S. Department of Health and Human Services
Food and Drug Administration
Center for Drug Evaluation and Research (CDER)
Center for Biologics Evaluation and Research (CBER)

October 2011
Labeling

TABLE OF CONTENTS

I. INTRODUCTION 2
II. WARNINGS AND PRECAUTIONS SECTION (§ 201.57(c)(6)) 3
A. Adverse Reactions That Should Be Included in the WARNINGS AND PRECAUTIONS Section 3
1. Serious Adverse Reactions 3
2. Otherwise Clinically Significant Adverse Reactions 4
3. Anticipated Adverse Reactions 4
4. Adverse Reactions Associated with Unapproved Uses 5
B. Risks or Other Hazards that Should be Included in the WARNINGS AND PRECAUTIONS Section 5
1. Laboratory Test Interference 5
2. Drug Interactions 5
3. Need for Monitoring to Assess Safety 6
C. Information to Provide in the Description of an Adverse Reaction 6
D. Format 7
1. Individual Subsections 7
2. Order of Adverse Reactions 7
3. Cross-Referencing 8
4. Emphasis in Text 8
III. CONTRAINDICATIONS SECTION (§ 201.57(c)(5)) 8
A. When to Contraindicate 8
1. Observed Adverse Reactions 8
2. Anticipated Adverse Reactions 8
B. Information to Provide 10
C. Format 11
1. Bulleted list 11
2. Order of Contraindications 11
IV. BOXED WARNING (§ 201.57(c)(1)) 11
A. When to Use a Boxed Warning 11
B. Information to Provide 12
C. Format 12
GLOSSARY 13

Guidance for Industry[1]

Warnings and Precautions, Contraindications, and Boxed Warning Sections of Labeling for Human Prescription Drug and Biological Products — Content and Format[2]

This guidance represents the Food and Drug Administration's (FDA's) current thinking on this topic. It does not create or confer any rights for or on any person and does not operate to bind FDA or the public. An alternative approach may be used if such approach satisfies the requirements of the applicable statutes and regulations. If you want to discuss an alternative approach, contact the FDA staff responsible for implementing this guidance. If you cannot identify the appropriate FDA staff, call the appropriate number listed on the title page of this guidance.

I. INTRODUCTION

This guidance is intended to assist applicants and reviewers in drafting the WARNINGS AND PRECAUTIONS, CONTRAINDICATIONS, and BOXED WARNING sections of labeling, as described in the final rule amending the requirements for the content and format of labeling for human prescription drug and biological products (21 CFR 201.56 and 201.57).[3] The recommendations in this guidance are intended to help ensure that the labeling is clear, useful, informative, and, to the extent possible, consistent in content and format.

This guidance provides recommendations on the following:

- How to decide which adverse reactions or other potential safety hazards are significant enough to warrant inclusion in the WARNINGS AND PRECAUTIONS section; what information to include when describing those adverse reactions; and how to organize the WARNINGS AND PRECAUTIONS section

[1] This guidance has been prepared by the Office of Medical Policy in the Center for Drug Evaluation and Research (CDER) and Center for Biologics Evaluation and Research (CBER) at the Food and Drug Administration.
[2] This guidance applies to drugs, including biological drug products. For the purposes of this guidance, *drug product* or *drug* will be used to refer to human prescription drug and biological products that are regulated as drugs.
[3] See the final rule "Requirements on Content and Format of Labeling for Human Prescription Drug and Biological Products," 2006, 71 FR 3922, http://www.fda.gov/Drugs/GuidanceComplianceRegulatoryInformation/LawsActsandRules/ucm084159.htm

Note: We update guidances periodically. To make sure you have the most recent version, check the CDER guidance page at http://www.fda.gov/Drugs/GuidanceComplianceRegulatoryInformation/Guidances/default.htm

- What situations warrant a contraindication; what information to provide in those situations when the use of the product is contraindicated; and how to organize the CONTRAINDICATIONS section
- When to include a boxed warning; and what information to include in the BOXED WARNING section

FDA's guidance documents, including this guidance, do not establish legally enforceable responsibilities. Instead, guidances describe the Agency's current thinking on a topic and should be viewed only as recommendations, unless specific regulatory or statutory requirements are cited. The use of the word *should* in Agency guidances means that something is suggested or recommended, but not required.

II. WARNINGS AND PRECAUTIONS SECTION (§ 201.57(c)(6))

A. Adverse Reactions That Should Be Included in the WARNINGS AND PRECAUTIONS Section

The WARNINGS AND PRECAUTIONS section is intended to identify and describe a discrete set of adverse reactions and other potential safety hazards that are *serious* or are *otherwise clinically significant* because they have implications for prescribing decisions or for patient management. To include an adverse event in the section, there should be reasonable evidence of a causal association between the drug and the adverse event, but a causal relationship need not have been definitively established.[4]

Some factors to consider in assessing whether there is reasonable evidence of a causal relationship include: (1) the frequency of reporting; 2) whether the adverse event rate in the drug treatment group exceeds the rate in the placebo and active-control group in controlled trials; (3) evidence of a dose-response relationship; (4) the extent to which the adverse event is consistent with the pharmacology of the drug; (5) the temporal association between drug administration and the event; (6) existence of dechallenge and rechallenge experience; and (7) whether the adverse event is known to be caused by related drugs.

1. Serious Adverse Reactions

An adverse reaction that results in any of the following outcomes should be considered serious and included in the WARNINGS AND PRECAUTIONS section:

- Death
- A life-threatening adverse event
- Inpatient hospitalization or prolongation of existing hospitalization
- A persistent or significant incapacity or substantial disruption of the ability to conduct normal life functions
- A congenital anomaly or birth defect

[4] See the Glossary for definitions of "adverse event" and "adverse reaction."

Important medical events that may not result in death, be life-threatening, or require hospitalization may be considered serious when, based on appropriate medical judgment, they may jeopardize the patient and may require medical or surgical intervention to prevent one of the outcomes listed in this definition (§§ 312.32(a) and 314.80(a)).

2. Otherwise Clinically Significant Adverse Reactions

Adverse reactions that do not meet the definition of a serious adverse reaction, but are otherwise clinically significant because they have implications for prescribing decisions or patient management, should also be included in the WARNINGS AND PRECAUTIONS section. The following can be factors in determining whether an adverse reaction is otherwise clinically significant:

- Indication

  The relative seriousness of the disease or condition treated should be considered. For example, non-serious adverse reactions (e.g., nausea, pruritis, alopecia) caused by drugs intended to treat minor, self-limiting conditions (e.g., allergic rhinitis, cosmetic conditions, transient insomnia) may be considered clinically significant. However, those same adverse reactions caused by drugs intended to treat serious or life-threatening conditions (e.g., cancer) may be considered much less clinically significant and not appropriate for inclusion in this section.

- Incidence

  A high absolute risk or rate of occurrence of an adverse reaction can be a factor in deciding whether to include the reaction in this section.

The following types of adverse reactions could be considered otherwise clinically significant:

- An adverse reaction that may lead to a potentially serious outcome unless the dosage or regimen is adjusted, the drug is discontinued, or another drug is administered to prevent the serious outcome
- An adverse reaction that could be prevented or managed with appropriate patient selection, monitoring, or avoidance of concomitant therapy, and prevention or management of the adverse reaction is needed to avoid a potentially serious outcome
- An adverse reaction that can significantly affect patient compliance, particularly when noncompliance has potentially serious consequences

3. Anticipated Adverse Reactions

There are circumstances in which an adverse reaction that has not been observed with a drug can nonetheless be anticipated to occur. The WARNINGS AND PRECAUTIONS section should include serious or otherwise clinically significant adverse reactions (as described in section II.A) that are anticipated to occur with a drug if:

- It appears likely that the adverse reaction will occur with the drug based on what is known about the pharmacology, chemistry, or class of the drug (e.g., a drug with a large QT prolongation effect would be likely to cause Torsades des Pointes arrhythmia even if no cases have yet been seen).

  OR

- Animal data raise substantial concern about the potential for occurrence of the adverse reaction in humans (e.g., animal data demonstrating that a drug has teratogenic effects)

Generally, when deemed important for the prescriber, the labeling should acknowledge that the adverse reaction has not been observed with the subject drug, but may be anticipated to occur.

4. Adverse Reactions Associated with Unapproved Uses

FDA may require in the WARNINGS AND PRECAUTIONS section a discussion of an adverse reaction associated with an unapproved use if the drug is commonly prescribed for a disease or condition and such usage is associated with a clinically significant risk or hazard (§ 201.57(c)(6)(i)). The description should include a statement indicating that safety and effectiveness have not been established in that setting and that the use is not approved by FDA.

B. Risks or Other Hazards that Should be Included in the WARNINGS AND PRECAUTIONS Section

1. Laboratory Test Interference

The WARNINGS AND PRECAUTIONS section must briefly note information on any known drug interference with laboratory tests (§ 201.57(c)(6)(iv)). Interference with a laboratory test means that the laboratory test result is inaccurate because the drug interferes with the assay (e.g., a false positive or negative test result is obtained that does not accurately reflect the quantity, presence, or absence of the analyte). It does not refer to a situation in which the test result is accurate, but outside the normal range because of the physiological effects caused by the drug or its metabolites.

Only clinically significant interferences should be included. Interference with a laboratory test would be considered clinically significant if reliance on the erroneous test result would influence clinical decision-making (e.g., false positive hemoccult test).

2. Drug Interactions

The WARNINGS AND PRECAUTIONS section should briefly describe any known or predicted drug interactions with serious or otherwise clinically significant outcomes and cross-reference to any more detailed information elsewhere in the labeling (e.g., DOSAGE AND ADMINISTRATION, DRUG INTERACTIONS, or CLINICAL PHARMACOLOGY sections).

3. Need for Monitoring to Assess Safety

The WARNINGS AND PRECAUTIONS section must identify any laboratory tests that would be helpful or necessary to identify possible adverse reactions (§ 201.57(c)(6)(iii)), or to prevent a serious adverse reaction. Information about the frequency of testing and expected ranges of normal and abnormal values should also be provided if available.

In general, information on monitoring to assess safety appears in WARNINGS AND PRECAUTIONS, and information on monitoring to assess effectiveness appears in DOSAGE AND ADMINISTRATION. In some cases, however, there may not be a clear distinction between monitoring for safety and effectiveness (e.g., cardiac monitoring to assess both safety and effectiveness in patients receiving antiarrhythmic drugs or INR testing to assess both safety and effectiveness in patients receiving warfarin), resulting in some overlap of information in WARNINGS AND PRECAUTIONS and DOSAGE AND ADMINISTRATION. Sections IIB, IIC, and IID in FDA's Dosage and Administration Final Guidance (*Dosage and Administration Section of Labeling for Human Prescription Drug and Biological Products — Content and Format*) state that the DOSAGE AND ADMINISTRATION section of the labeling should contain monitoring information to assess both effectiveness and safety, specifically how such monitoring affects dosing of the drug (e.g., titrating the dose, modifying the dose, or discontinuing treatment).

C. Information to Provide in the Description of an Adverse Reaction

There should be a succinct description of each topic selected for inclusion in the WARNINGS AND PRECAUTIONS section. The description should cross-reference any more detailed discussion of the risk elsewhere in labeling (e.g. ADVERSE REACTIONS, DRUG INTERACTIONS, USE IN SPECIFIC POPULATIONS, CLINICAL STUDIES). The description should be limited to the following information, and information should be included only if known and important to clinical decision making:

- A succinct description of the adverse reaction and outcome (e.g., when the reaction occurs, whether the reaction abates over time despite continued treatment, time to resolution, significant sequelae).
- A numerical estimate of risk or adverse reaction rate[5]

[5] In characterizing overall adverse reaction experience, nonspecific terms that lack a commonly understood or precise meaning should be avoided because use of such terms can be misleading. For example, the terms *rare, infrequent,* and *frequent* do not provide meaningful information about the adverse event's frequency of occurrence.

- Known risk factors for the adverse reaction (e.g., age, gender, race, genetic polymorphism, comorbid conditions, dose, duration of use, coadministered drugs)
- Steps to take to decrease the likelihood, shorten the duration, or minimize the severity of an adverse reaction. These steps could include, for example, necessary evaluation prior to use, titration and other kinds of dose adjustment, monitoring during dose adjustment or prolonged use, avoidance of other drugs or substances, or special care during comorbid events (e.g., dehydration, infection)
- How to treat or otherwise manage an adverse reaction that has occurred

The information and advice provided should be reasonably qualified, where appropriate, to convey whatever uncertainties may exist about judgments and conclusions made (e.g., concerning causality assessments, estimated adverse reaction rates, and value of proposed monitoring).

Ambiguous and uninformative statements (e.g., use with caution) should be avoided. Instead, specific treatment or management strategies should be noted (e.g., consider lower doses or more frequent monitoring). Terminology that generally infers a contraindication (e.g., “Do not use” or “Drug X should not be used”) should not appear in the WARNINGS AND PRECAUTIONS section.

D. Format

1. Individual Subsections

Each adverse reaction, syndrome, or group of reactions with a common pathogenesis (e.g., allergic contact dermatitis, maculopapular drug rash) included in the WARNINGS AND PRECAUTIONS section should have its own numbered subsection. The subsection title should accurately characterize the risk (e.g., 5.1 Thromboembolic Disorders, 5.2 Peripheral Neuropathy). When necessary, information in a subsection can be organized under non-numbered subheadings using formatting techniques such as underlining or italicizing for the subheading titles. For example, the text of a subsection entitled “5.1 Thromboembolic Disorders” can include the subheadings “Deep Vein Thrombosis” and “Thrombotic Stroke” (not “5.1.1 Deep Vein Thrombosis” and “5.1.2 Thrombotic Stroke”). Subsection headings that are not useful for signaling the content of the subsection (e.g., General) should be avoided.

2. Order of Adverse Reactions

The order in which adverse reactions are presented in the WARNINGS AND PRECAUTIONS section should reflect the relative clinical significance of the adverse reactions. Factors to consider include the relative seriousness of the adverse reaction, the ability to prevent or mitigate the adverse reaction, and the likelihood of its occurrence.

Footnote 5 continued: If categorizing adverse reactions by frequency, ranges would be helpful in understanding the drug’s safety profile and the ranges should be clearly defined (e.g., occurring at a rate less than 1/100, occurring at a rate of less than 1/500).

3. Cross-Referencing

When more detailed information about an adverse reaction is included in another labeling section, the WARNINGS AND PRECAUTIONS section should cross-reference that section (e.g., ADVERSE REACTIONS, DRUG INTERACTIONS, CLINICAL PHARMACOLOGY), rather than repeat the same information. To the extent possible, redundancies should be avoided in labeling, and cross-referencing should be used instead.

4. Emphasis in Text

Bolded text or other emphasis can be used to highlight particular adverse reactions or parts of the discussion of particular adverse reactions (e.g., steps to be taken to avoid a problem, subpopulations at particular risk). Emphasis should be used sparingly so that its effect is not diminished. Thus, the entire text of a subsection in WARNINGS AND PRECAUTIONS should not be bolded; rather, bolding should be limited to only one or two sentences. Consider whether information to be emphasized should rise to the level of a Boxed Warning (see Section IV on BOXED WARNING).
.

III. CONTRAINDICATIONS SECTION (§ 201.57(c)(5))

A. When to Contraindicate

A drug should be contraindicated only in those clinical situations for which the risk from use clearly outweighs any possible therapeutic benefit. Only known hazards, and not theoretical possibilities, can be the basis for a contraindication. If there are no known contraindications for a drug, this section must state "None."

1. Observed Adverse Reactions

For observed adverse reactions, the following would ordinarily be reason to contraindicate a drug:

- The risk of the adverse reaction in the clinical situation to which the contraindication applies, based on both likelihood and severity of the adverse reaction, outweighs any potential benefit to any patient.

  AND

- The causal relationship between exposure to the drug and the adverse reaction is well established.

2. Anticipated Adverse Reactions

Adverse reactions that are anticipated to occur when a drug is used in a specific clinical situation can be the basis for a contraindication.

Anticipated adverse reactions are distinguishable from "theoretical possibilities". Anticipated adverse reactions are supported by data (e.g., from known pharmacologic effects, class effect, chemical relationships to other drugs known to cause reactions, animal studies) and may be considered for the CONTRAINDICATIONS section. Adverse reactions based wholly on theory (theoretical possibilities) are not supported by data and would not be appropriate to include in the CONTRAINDICATIONS section.

Ordinarily, a drug should be contraindicated on the basis of an anticipated adverse reaction if the risk of the adverse reaction in the clinical situation to which the contraindication will apply, based on both likelihood and severity of the adverse reaction, outweighs any potential benefit to any patient:

AND EITHER

- Based on what is known about the pharmacology, chemistry, or class of the drug, it appears highly likely that the adverse reaction is caused by the drug.

OR

- Animal data raise substantial concern about the potential for occurrence of the adverse reaction in humans (e.g., animal data demonstrating that a drug has teratogenic effects).

The labeling should acknowledge that the adverse reaction has not yet been observed, but is anticipated to occur.

The following illustrate clinical situations for which a contraindication might be appropriate:

- Use in the presence of a comorbid condition or coexistent physiological state (e.g., existing hepatic disease, renal disease, congenital long QT syndrome, hypokalemia, pregnancy or childbearing potential, CYP 2D6 poor metabolizer[6])

- Use in the presence of a demographic risk factor, such as age, gender or other factors (e.g., contraindication in females of reproductive potential, in children below a certain age)

- Use in a defined subset of patients (e.g., people with mild disease) where the risks of the drug are such that the drug should never be used in that subset of the larger population[7]

[6] Use of a particular drug in a patient with a slow metabolizer status would be contraindicated only in situations where the dose of the drug could not be adequately adjusted.
[7] The INDICATIONS AND USAGE section must contain information about use of the drug when safety considerations are such that the drug should be reserved for certain patients (e.g., patients with severe disease) or situations (e.g., patients refractory to other drugs) (§ 201.57(c)(2)(i)(B) and (E)). In rare cases, when the risks of the drug clearly outweigh any possible therapeutic benefit and the drug should never be used in a selected patient subset, a contraindication for use of the drug in that subset should also be described in the CONTRAINDICATIONS section.

- Use with coadministered drugs where the combination is dangerous (e.g., MAO inhibitor with a tricyclic antidepressant; a drug known to prolong the QT interval with a drug known to interfere with the metabolism of that drug)

- Use of a drug in patients with known hypersensitivity when severe hypersensitivity reactions have been observed to occur with the drug.

  A contraindication in patients with hypersensitivity reactions should be included in labeling only when there are demonstrated cases of hypersensitivity with the product or such reactions may be anticipated based on data from similar drugs (e.g., those in the same pharmacological class or with similar chemical structures, or when cross-sensitivity within a class is a recognized phenomenon). When the risk of using the drug in a patient at risk for such a reaction outweighs the potential benefits (i.e., it would be clinically inappropriate to rechallenge a patient with a history of a hypersensitivity reaction to the drug or a similar drug), a contraindication to use in such patients should be included. Along with the contraindication statement, the labeling should briefly describe the type and nature of the observed (or anticipated) reaction(s), and cross-reference to a more detailed discussion elsewhere in the labeling, as appropriate.

  For example:

  DRUG-X is contraindicated in patients with a history of a hypersensitivity reaction to [active ingredient]. Reactions have included anaphylaxis and anaphylactoid reactions *[see Adverse Reactions (6.2)]*.

  If no such hypersensitivity reactions as noted above have been observed or are unlikely to occur based on the drug's characteristics, no contraindication for hypersensitivity reactions will be included.

Contraindications based on drug interactions with serious outcomes should be described briefly in the CONTRAINDICATIONS section and cross-referenced to more detailed information in the DRUG INTERACTIONS or CLINICAL PHARMACOLOGY sections.

B. Information to Provide

Contraindications should be worded using precise language, e.g., “Drug X is contraindicated in patients with condition Y” (instead of “Drug X should not be used in patients with condition Y”). If a drug has more than one contraindication, use an introductory statement (e.g., "Drug X is contraindicated in:") followed by a bulleted list identifying each contraindication.

For each listed contraindication, provide the following information:

- Brief description of the contraindicated situation or scenario, including any pertinent demographic or identifiable predisposing characteristics

- Description of observed or anticipated consequences of the contraindicated use

C. Format

1. Bulleted list

If a drug has more than one contraindication, FDA recommends that each contraindication be identified in a bulleted list.

2. Order of Contraindications

The order in which contraindications are presented should reflect the relative clinical significance of the listed contraindications. Factors to consider include the severity of the risk and the likelihood of occurrence.

IV. BOXED WARNING (§ 201.57(c)(1))

A. When to Use a Boxed Warning

A boxed warning is ordinarily used to highlight for prescribers one of the following situations:

- There is an adverse reaction so serious in proportion to the potential benefit from the drug (e.g., a fatal, life-threatening or permanently disabling adverse reaction) that it is essential that it be considered in assessing the risks and benefits of using the drug

  OR

- There is a serious adverse reaction that can be prevented or reduced in frequency or severity by appropriate use of the drug (e.g., patient selection, careful monitoring, avoiding certain concomitant therapy, addition of another drug or managing patients in a specific manner, avoiding use in a specific clinical situation)

  OR

- FDA approved the drug with restrictions to ensure safe use because FDA concluded that the drug can be safely used only if distribution or use is restricted (e.g., under 21 CFR 314.520 and 601.42 “Approval with restrictions to assure safe use” or under 505-1(f)(3) of the Federal Food, Drug, and Cosmetic Act (FDCA) "Risk Evaluation and Mitigation Strategies” Elements to assure safe use).

Infrequently, a boxed warning can also be used in other situations to highlight warning information that is especially important to the prescriber (e.g., reduced effectiveness in certain patient populations). Information included in the WARNINGS AND PRECAUTIONS and CONTRAINDICATIONS sections should therefore be evaluated to determine whether it warrants inclusion in a boxed warning.

Boxed warnings are most likely to be based on observed serious adverse reactions, but there are instances when a boxed warning based on an anticipated adverse reaction would be appropriate.

For example, a contraindication to use during pregnancy based on evidence in humans or animals that drugs in a pharmacologic class pose a serious risk of developmental toxicity during pregnancy would usually be in a boxed warning for all drugs in that class, even those in which the adverse reaction has not been observed.

A boxed warning can also be considered for a drug that poses risk–benefit considerations that are unique among drugs in a drug class (e.g., to note when a drug is the only one in its class to have a particular risk and is indicated as second line therapy because of that risk).

B. Information to Provide

A boxed warning provides a brief, concise summary of the information that is critical for a prescriber to consider, including any restriction on distribution or use. There is typically a more detailed discussion of the risk elsewhere in the labeling (e.g., in CONTRAINDICATIONS or WARNINGS AND PRECAUTIONS sections), that must be identified by a cross-reference (§ 201.57(c)(1)).

C. Format

The BOXED WARNING section in the full prescribing information must be formatted in accordance with § 201.57(d). The information in the boxed warning should be in bold print and presented in a bulleted format or some alternative format, such as the use of subheadings, that helps to make the information visually accessible.

GLOSSARY

Adverse Reaction (21 CFR 201.57(c)(7)): For purposes of prescription drug labeling and this guidance, an *adverse reaction* is an undesirable effect, reasonably associated with the use of a drug, that may occur as part of the pharmacological action of the drug or may be unpredictable in its occurrence. This definition does not include all adverse events observed during use of a drug, only those for which there is some basis to believe there is a causal relationship between the drug and the occurrence of the adverse event.

Adverse reactions may include signs and symptoms, changes in laboratory parameters, and changes in other measures of critical bodily function, such as vital signs and electrocardiogram (ECG).

Adverse Event: For the purposes of this guidance, an *adverse event* refers to any untoward medical occurrence associated with the use of a drug in humans, whether or not considered drug related.

Serious Adverse Reaction: For purposes of this guidance, the term *serious adverse reaction* refers to any event or reaction that results in any of the following outcomes: Death, a life-threatening adverse event, inpatient hospitalization or prolongation of existing hospitalization, a persistent or significant incapacity or substantial disruption of the ability to conduct normal life functions, or a congenital anomaly or birth defect. Important medical events that may not result in death, be life-threatening, or require hospitalization may be considered serious when, based upon appropriate medical judgment, they may jeopardize the patient or subject and may require medical or surgical intervention to prevent one of the outcomes listed in this definition.
