# Supplementary material for: Retrieval Augmented Generation (RAG) for Evaluating Regulatory Compliance of Drug Information and Clinical Trial Protocols
Source: CPT Pharmacometrics Syst Pharmacol. 2026 Feb 19;15(3):e70201. doi: 10.1002/psp4.70201 (PMC12917324; doi:10.1002/psp4.70201)
Supplement: Supplementary file 1 — Data S1: psp470201‐sup‐0001‐Supinfo.zip. [file PSP4-15-e70201-s001.zip › PSP-2025-0274-s03.pdf]

SUPPLEMENTARY FILE S3

IMPLEMENTATION DETAILS & RAG SYSTEM RESULTS FOR DRUG INFORMATION EVALUATION

Retrieval Augmented Generation (RAG) for Evaluating Compliance of Drug Information to FDA Regulatory Guidance Documents

Shreyas Waikar, Amruta Gajanan Bhat, and Murali Ramanathan
Artificial Intelligence & Clinical Pharmacology Laboratory, Department of Pharmaceutical Sciences, University at Buffalo, The State University of New York, Buffalo, NY, USA.

CORRESPONDING AUTHOR: Murali Ramanathan
355 Pharmacy, Department of Pharmaceutical Sciences
University at Buffalo, Buffalo, NY 14214-8033.
(716)-645-4846 and FAX 716-829-6569. E-mail Murali@Buffalo.Edu

Running Head: RAGs for Drug Development

Keywords: Artificial Intelligence, AI, LLM, RAG, Pharmacometrics, MIDD, Clinical Pharmacology

SUPPLEMENTARY METHODS

Design and Implementation Details of the Drug Information Evaluation System

Queries: The three queries for the drug information evaluation are summarized in the Methods section.

Scratchpad

A) Instruction "scratchpad" *Please provide a detailed response using only the information found in the selected documents. Your answer must be written as a single paragraph—do not use line breaks or start a new paragraph. Focus solely on the provided context to answer the question accurately.*

Prompt Structure:

You are a document-restricted assistant.

Instructions:{editable_plan}

Context: {selected_texts}

Question: {other_points}

IMPORTANT: Always structure your response in this exact format:

1. Section Content: *List the actual {labeling section} as stated in the document.*

2. Compliance Evaluation:

- General Principles: Assess clarity, conciseness, and alignment with guidance.

- Content Requirements: Check for explicit statements about treatment, prevention, etc., limitations of use, qualifiers, and safety/effectiveness comparisons.

- Format Requirements: Evaluate organization, formatting, and separation of limitations of use.

3. Missing or Inconsistent Components:

- Missing: List any required elements not present.

- Incomplete: Note any elements that are present but not fully addressed.

- Misaligned: Identify any content that does not match guidance requirements.

C) Reference-answer prompt (ref_prompt)

You are an assistant with access to the following documents.

Context: {selected_texts}

Question: {query}

Configuration: The necessary libraries were imported to enable document processing, embedding generation, vector storage, model interaction, and evaluation. The OpenAI Application Programming Interface (API) environment key was set to establish access to the OpenAI services.

Document Ingestion and Preprocessing: PDF documents, including package inserts and regulatory references, are uploaded through a Streamlit interface. Each document is parsed using *PyPDF2.PdfReader* to extract raw text and section headings. Section headings are filtered using a regex-based heuristic that captures numbered, uppercase phrases. Selected sections are extracted for targeted review or annotation.

Sentence Embedding and Indexing: The extracted text from the reference document was segmented into 512-character chunks with 50-character overlap. The chunks were embedded using the *all-MiniLM-L6-v2* model from SentenceTransformers. These embeddings are indexed using the *FAISS* (Facebook AI Similarity Search) library with an *IndexFlatL2* configuration, allowing fast similarity-based retrieval across the reference documents.

Query Processing: The user query information was incorporated with the scratchpad. Regular expression search was used to extract package insert sections relevant to the query. The RAG extracted the top-20 query-relevant chunks from each reference document using embeddings as input to a semantic-similarity search in the *FAISS* library. The query, scratchpad, regular expression search, query relevant chunks, and the remaining elements of the prompt structure comprised the prompt submitted to the LLM.

Language Model Generation: The engineered prompt was passed to the GPT-4o model via the ChatOpenAI method to obtain two generated responses. One response was restricted to the user documents, while the other was output generated by the unrestricted GPT-4o model.

Model Hyperparameters:

**Chunking**	Chunk size = 512 characters with 50-character overlap
**Embedding**	Model = *all-MiniLM-L6-v2* (SentenceTransformers), Embedding dimension = 384, Vector index: *FAISS IndexFlatL2*
**Similarity Search**	Using score and retrieve top 20 most relevant chunks

**ChatOpenAI**	GPT-4o model accessed through the Chat Completions API (*openai* v1.40.0), Maximum tokens = 4000, Temperature = 0 (deterministic decoding), Number of responses = 1, Frequency and presence penalties: default values, Random seed = 42. The model supports a 128k-token context window; the *max_tokens* parameter constrained generated output to approximately 3,000 words per response.
**Metrics**	**Evaluation**: Automatic text-similarity metrics in the Hugging Face *evaluate* library (v0.4.1).<br>**Metrics**: ROUGE-L and METEOR, model = GPT-4o

Metric Evaluation: The document-restricted LLM response was compared to the unrestricted response using the text-similarity metrics in the Hugging Face *evaluate* library. The ROUGE-L and METEOR described in Methods were computed and converted to percentages.

Output Management: Generated answers are stored in a persistent chat history with the user queries. Users can review, export, and download the full chat transcript as a PDF. Additionally, extracted or edited document sections can be saved as PDFs using the *reportlab* library.

Design and Implementation Details of the Clinical Trial Protocol Evaluation System

Queries: As described in Methods, two queries were submitted to the LLM. Query 1: "*Extract and summarize the clinical trial protocol and statistical analysis plan of the given clinical*

trial." Query 2: "*Based on the FDA E9 Statistical Principles for Clinical Trials, evaluate the statistical analysis plan of the clinical trial protocol*."

Scratchpad: Scratchpad set for Query 1:

1. extract the given key sections from the document.

2. summarize each of them briefly.

3. If the information is not given directly, infer from the given context.

4. Ignore the guidance if given.

Scratchpad set for Query 2:

For each key statistical analysis section in the clinical trial protocol:

- Identify any aspects that align with ICH E9 principles.

- Note any gaps, unclear content, or concerns under Comment.

- Provide an Improvement, prefixed by its Criticality (e.g., [Medium]).

- Keep all observations grouped under each key section heading only. Do not create separate headers for comments or recommendations.

Prompt structure: *You are an expert at the United States Food and Drug Administration (FDA) with 20 years of regulatory experience reviewing clinical trial protocols.*

Extract information as accurately as possible based on the context provided and structure your response into clearly labeled sections.

User Query: {query}

Clinical Trial Protocol or SAP chunks: {protocol_chunks}

Regulatory Guidance chunks: {guidance_chunks}

scratchpad: {scratchpad_plan}

If a section is not available in the document, state "Not specified."

Respond concisely but completely using bullet points and short paragraphs.

Configuration: The necessary libraries were imported to enable document processing, embedding generation, vector storage, model interaction, and evaluation. The OpenAI API environment key was set to establish access to the OpenAI services.

Document Ingestion and Preprocessing: The PDF documents, including clinical trial protocols, Statistical Analysis Plan (SAPs), and regulatory guidance, were uploaded through a Streamlit interface. A library of guidance documents (e.g., E9 Statistical Principles for Clinical Trials document) stored locally was retrieved and presented to users through the *Streamlit* user interface for selection and upload. Raw text and tables were extracted by parsing the input documents with *PyMuPDFLoader* (from *langchain_community.document_loaders* (1) library).

Sentence Embedding and Indexing: The uploaded documents were processed using the *LangChain* module. Text and tables were extracted with the *PyMuPDFLoader*, and the documents were divided into smaller chunks using the *RecursiveCharacterTextSplitter* (from

the *langchain.text_splitter* (2) library) based on the default separators: "\n\n", "\n", " ", ".", and "," with a chunk size of 500 and no overlap.

Query Processing: The embeddings of the document chunks were generated using the *OpenAIEmbeddings* method with the *text-embedding-3-small* model, and the resulting embeddings were stored using *FAISS* for efficient similarity search and retrieval.

Once a user entered a query, it was matched against both the guidance and protocol document embeddings using *similarity_search_with_score* method. The top 50 most relevant chunks were selected for further analysis. The retrieved information was incorporated into a predefined prompt structure shown in the prompt section.

Language Model Generation: The engineered prompt was passed to the *ChatOpenAI* method from the *langchain_community.chat_models* module. The *GPT-4o* model was used with one generated response (*n = 1*) and a temperature setting of zero to ensure deterministic outputs.

The model responses were evaluated using DeepEval's *Answer Relevancy* and *Faithfulness* metrics (GPT-4o model; threshold = 0.7). Additionally, a custom *ClinPharm* was developed using GEval (GPT-4o model; threshold = 0).

For each metric, the test case was defined as follows: the input was the user query, the actual response was the model output, and the retrieval context consisted of the top 50 most relevant chunks. DeepEval produced evaluation scores between 0 and 1, which were multiplied by 100 to yield percentage values. The reasoning associated with each score was also reported.

Model Hyperparameters:

**Chunking**	Chunk size = 500 tokens and no overlap
**Embedding**	Model = *text-embedding-3-small*
**Similarity Search**	Using score and retrieve Top 50 most relevant chunks
**ChatOpenAI**	Model = GPT-4o, Number of generated responses = 1 and Temperature = 0
**DeepEval**	**Test case:** input = query, response = LLM's response, retrieved context = top 50 most relevant chunks<br>**Metrics:** Answer Relevancy: Threshold = 0.7, model = GPT-4o<br>Faithfulness: Threshold = 0.7, model = GPT-4o<br>GEval**:** Threshold = 0, model = GPT-4o

Web Context Augmentation (Optional): If enabled, additional context is retrieved from the web using the SerpAPI Google Search engine. Top snippets from search results are extracted and appended to the local document context to enrich the prompt.

Output Management was done using the methods previously described in the Design of the Drug Information Evaluation System section.

Table S1. Results from the RAG system for insulin glargine, atorvastatin calcium, sertraline, alprazolam against each FDA guidance document.

**Drug Name**	**Guidance-1**<br>**Indications**	**Guidance-2**<br>**Use in Specific Populations**	**Guidance-3**<br>**Warnings & Precautions**
**Query**	1. List the indications and usage from "Indications and Usage" section in "Drug_name.pdf".<br>2. Evaluate whether this section complies with the expectations outlined in the guidance documents "Indications.pdf" and "21 CFR 201.57.pdf". Specifically, assess whether the general principles, content, and format requirements are clearly stated.<br>3. Identify any missing or inconsistent components in Drug_name.pdf' relative to the requirements outlined in the relevant guidance documents. Specify which components are absent, incomplete, or misaligned, and reference the applicable sections of the guidance documents where appropriate.	1. List the Use in Specific Populations from "Use in Specific Populations" section in "Drug_name.pdf".<br>2. Evaluate whether this section complies with the expectations outlined in the guidance documents "UseinSpecificPopulation.pdf" and "21 CFR 201.57.pdf". Specifically, assess whether the general principles, content, and format requirements are clearly stated.<br>3. Identify any missing or inconsistent components in Drug_name.pdf' relative to the requirements outlined in the relevant guidance documents. Specify which components are absent, incomplete, or misaligned, and reference the applicable sections of the guidance documents where appropriate.	1. List the Warnings and Precautions from "Warnings and Precautions" section in "Drug_name.pdf".<br>2. Evaluate whether this section complies with the expectations outlined in the guidance documents "Warnings&Precautions.pdf" and "21 CFR 201.57.pdf". Specifically, assess whether the general principles, content, and format requirements are clearly stated.<br>3. Identify any missing or inconsistent components in Drug_name.pdf' relative to the requirements outlined in the relevant guidance documents. Specify which components are absent, incomplete, or misaligned, and reference the applicable sections of the guidance documents where appropriate.
**Insulin glargine (LANTUS®)**	The "Indications and Usage" section in "Lantus.pdf" states that Lantus is a long-acting human insulin analog indicated to improve glycemic control in adult and pediatric patients with diabetes mellitus. It also specifies a limitation of use, stating that Lantus is not recommended for the treatment of diabetic ketoacidosis.<br>Compliance Evaluation:<br>General Principles: The section is straightforward, clear, and concise, which aligns with the general principles outlined in both guidance documents. It clearly communicates the approved indication and the applicable population, enabling health care practitioners to identify appropriate therapies for patients.<br>Content Requirements: The section states the condition (diabetes mellitus) for which the drug is approved and its role in improving glycemic control, which aligns with the content requirements. It also includes a limitation of use,	The "Use in Specific Populations" section in "Lantus.pdf" includes the following usage:<br>8.1 Pregnancy: Discusses the risk summary, clinical considerations, and data related to the use of insulin glargine during pregnancy.<br>8.2 Lactation: Provides a risk summary regarding the presence of insulin glargine in human milk and its potential effects on the breastfed infant and milk production.<br>8.4 Pediatric Use: Discusses the safety and effectiveness of LANTUS in pediatric patients with diabetes mellitus.<br>8.5 Geriatric Use: Discusses the use of LANTUS in geriatric patients, emphasizing caution due to potential difficulty recognizing hypoglycemia in this population.<br>8.6 Renal Impairment: Discusses the lack of study on the effect of kidney impairment on the pharmacokinetics of LANTUS and the potential need for dosage adjustment in patients with kidney impairment.<br>Compliance Evaluation:	The Warnings and Precautions listed in the "Warnings and Precautions" section in "Lantus.pdf" are as follows:<br>Never share a LANTUS SoloStar prefilled pen, insulin syringe, or needle between patients, even if the needle is changed. (5.1)<br>Hyperglycemia or hypoglycemia with changes in insulin regimen: Make changes to a patient's insulin regimen under close medical supervision with increased frequency of blood glucose monitoring. (5.2)<br>Hypoglycemia: May be life-threatening. Increase frequency of glucose monitoring with changes to insulin dosage, concomitant drugs, meal pattern, physical activity; and in patients with renal or hepatic impairment and hypoglycemia unawareness. (5.3)<br>Hypoglycemia due to medication errors: Accidental mix-ups between insulin products can occur. Instruct patients to check insulin labels before injection. (5.4)

	stating that Lantus is not recommended for the treatment of diabetic ketoacidosis.<br>Format Requirements: The section is organized and formatted to be clear and concise, with the indication and limitation of use listed separately, which aligns with the format requirements.<br>Missing or Inconsistent Components:<br>The section does not state whether Lantus is indicated for the treatment, prevention, mitigation, cure, or diagnosis of diabetes mellitus, including relief of symptoms. This is a content requirement outlined in "Indications.pdf".<br>The section does not describe if the drug is only safe and effective for certain subgroups, if specific tests are necessary for patient selection or monitoring, or if there are specific conditions for long-term use. These are content requirements outlined in "21 CFR 201.57.pdf".<br>The section does not include any statements comparing the safety or effectiveness of Lantus with other agents for the same indications. This is a content requirement outlined in "Indications.pdf" and "21 CFR 201.57.pdf".<br>The section does not state if there is a common belief about Lantus's effectiveness for a certain use, but evidence shows it is ineffective or risky. This is a content requirement outlined in "21 CFR 201.57.pdf".	General Principles: The "Use in Specific Populations" section in "Lantus.pdf" does not explicitly mention the ethical considerations for clinical investigations involving children as outlined in "UseinSpecificPopulation.pdf". However, it does provide information on the use of LANTUS in pediatric patients, which may indirectly imply adherence to these principles. The section also aligns with the principles outlined in "21 CFR 201.57.pdf" as it provides information supported by evidence and does not imply or suggest uses not included in the section.<br>Content Requirements: The section meets the content requirements of "21 CFR 201.57.pdf" by providing information on the drug's use in specific populations, including pregnancy, lactation, pediatric use, geriatric use, and renal impairment. It does not explicitly meet the content requirements of "UseinSpecificPopulation.pdf" as it does not mention provisions for soliciting the assent of children or the permission of their parents or guardians.<br>Format Requirements: The section follows the format requirements of "21 CFR 201.57.pdf" by including each heading and subheading required in the full prescribing information and presenting the information in the required order. It does not explicitly meet the format requirements of "UseinSpecificPopulation.pdf" as it does not mention review and approval by Institutional Review Boards (IRBs).<br>Missing or Inconsistent Components:<br>From "UseinSpecificPopulation.pdf": The section does not explicitly mention the ethical considerations for clinical investigations involving children, provisions for soliciting the assent of children or the permission of their parents or guardians, or review and approval by IRBs. These are key requirements outlined in the guidance document.<br>From "21 CFR 201.57.pdf": The section appears to meet all the requirements outlined in this guidance document. However, it does not explicitly state the date of the most recent revision of the labeling, which is a content requirement.	Hypersensitivity reactions: Severe, life-threatening, generalized allergy, including anaphylaxis, can occur. Discontinue LANTUS. Monitor and treat if indicated. (5.5)<br>Hypokalemia: May be life-threatening. Monitor potassium levels in patients at risk of hypokalemia and treat if indicated. (5.6)<br>Fluid retention and heart failure with concomitant use of thiazolidinediones (TZDs): Observe for signs and symptoms of heart failure; consider dosage reduction or discontinuation of TZD if heart failure occurs. (5.7)<br>Compliance Evaluation:<br>General Principles: The "Warnings and Precautions" section in "Lantus.pdf" is clear, informative, and consistent in content and format, which aligns with the general principles outlined in the "Warnings&Precautions.pdf" guidance document.<br>Content Requirements: The section includes serious or clinically significant adverse reactions, which is in line with the content requirements from both "Warnings&Precautions.pdf" and "21 CFR 201.57.pdf" guidance documents.<br>Format Requirements: The section is organized with clear headings and subheadings, which aligns with the format requirements outlined in both "Warnings&Precautions.pdf" and "21 CFR 201.57.pdf" guidance documents.<br>Missing or Inconsistent Components:<br>The "Warnings and Precautions" section in "Lantus.pdf" does not include a BOXED WARNING section. According to the "Warnings&Precautions.pdf" guidance document, a boxed warning should be included when necessary. However, without more context, it's unclear whether a boxed warning is necessary for this drug.<br>The "Warnings and Precautions" section does not identify situations warranting a contraindication, which is a requirement outlined in the "Warnings&Precautions.pdf" guidance document.<br>The "Warnings and Precautions" section does not include the date of the most recent revision of the labeling, which is

			a requirement outlined in the "21 CFR 201.57.pdf" guidance document.<br>The "Warnings and Precautions" section does not contain a list of each heading and subheading required in the full prescribing information, which is a requirement outlined in the "21 CFR 201.57.pdf" guidance document.<br>The "Warnings and Precautions" section does not contain the information in the order required under the FDA guidance, together with the headings, subheadings, and identifying numbers, which is a requirement outlined in the "21 CFR 201.57.pdf" guidance document.
**Atorvastatin calcium (LIPITOR®)**	The "Indications and Usage" section in "Lipitor.pdf" states that Lipitor is indicated to reduce the risk of:<br>Myocardial infarction (MI), stroke, revascularization procedures, and angina in adults with multiple risk factors for coronary heart disease (CHD) but without clinically evident CHD<br>MI and stroke in adults with type 2 diabetes mellitus with multiple risk factors for CHD but without clinically evident CHD<br>Non-fatal MI, fatal and non-fatal stroke, revascularization procedures, hospitalization for congestive heart failure, and angina in adults with clinically evident CHD<br>Compliance Evaluation:<br>General Principles: The section appears to be straightforward, clear, and concise, which aligns with the general principles outlined in both guidance documents. It communicates the approved indications of Lipitor, enabling healthcare practitioners to identify appropriate therapies for patients.<br>Content Requirements: The section states the conditions for which Lipitor is approved, meeting the content requirements from both guidance documents. However, it does not explicitly state whether Lipitor is used for the treatment, prevention, mitigation, cure, or diagnosis of these conditions. It also does not include any limitations of use, additional descriptors or qualifiers as part of the indication, or any statements comparing the safety or	Summary: The "Use in Specific Populations" section in the Lipitor prescribing information provides information on pregnancy, lactation, pediatric use, geriatric use, renal impairment, and hepatic impairment. It indicates that Lipitor is not safe for pregnant or lactating individuals, and its safety and effectiveness in pediatric patients with familial hypercholesterolemia have been established. It also mentions the use of Lipitor in geriatric patients and provides data on lipid-altering effects in adolescent boys and girls with heterozygous familial hypercholesterolemia. The section includes dosing information and references relevant clinical studies.<br>Evaluation: The "Use in Specific Populations" section in the Lipitor prescribing information aligns with the expectations outlined in the guidance documents "UseinSpecificPopulation.pdf" and "21 CFR 201.57.pdf." It includes important ethical considerations by advising against the use of Lipitor in pregnant and lactating individuals. The section provides data on the use of Lipitor in pediatric and geriatric populations and mentions dosing considerations. It also offers information on lipid-altering effects in specific patient populations.<br>However, there are some missing components in the section:<br>While data on pediatric use in patients with heterozygous familial hypercholesterolemia is provided, there is no information on the use of Lipitor in other types of	Warnings and Precautions from "Warnings and Precautions" section in "Lipitor.pdf":<br>Myopathy and Rhabdomyolysis: LIPITOR may cause myopathy (muscle pain, tenderness, or weakness associated with elevated creatine kinase [CK]) and rhabdomyolysis. Acute kidney injury secondary to myoglobinuria and rare fatalities have occurred as a result of rhabdomyolysis in patients treated with statins, including LIPITOR.<br>Risk Factors for Myopathy: Risk factors for myopathy include age 65 years or greater, uncontrolled hypothyroidism, renal impairment, concomitant use with certain other drugs (including other lipid-lowering therapies), and higher LIPITOR dosage.<br>Steps to Prevent or Reduce the Risk of Myopathy and Rhabdomyolysis: LIPITOR exposure may be increased by drug interactions due to inhibition of cytochrome P450 enzyme 3A4 (CYP3A4) and/or transporters (e.g., breast cancer resistant protein [BCRP], organic anion-transporting polypeptide [OATP1B1/OATP1B3]). In patients taking clarithromycin or itraconazole, do not exceed LIPITOR 20 mg once daily.<br>Compliance Evaluation:<br>General Principles: The Warnings and Precautions section in the Lipitor package insert is clear, informative, and consistent in content and format, meeting the general principles outlined in the "Warnings&Precautions.pdf" guidance.

	effectiveness of Lipitor with other agents for the same indications.<br>Format Requirements: The section is organized and formatted to be clear and concise, with each indication listed separately. However, limitations of use are not clearly formatted and listed separately.<br>Missing or Inconsistent Components:<br>Missing: The section does not explicitly state whether Lipitor is used for the treatment, prevention, mitigation, cure, or diagnosis of the conditions listed, as required by both guidance documents (Indications.pdf: Content Requirements; 21 CFR 201.57.pdf: Key Requirements). It also does not include any limitations of use, additional descriptors or qualifiers as part of the indication, or any statements comparing the safety or effectiveness of Lipitor with other agents for the same indications, as required by the Indications.pdf guidance (Content Requirements).<br>Incomplete: The section does not include any limitations of use, which should be clearly formatted and listed separately according to the Indications.pdf guidance (Format Requirements).<br>Misaligned: There are no misaligned components identified in this analysis.	hyperlipidemia in pediatric patients, as required by guidance documents.<br>The section could provide more information on dosing considerations and risk statements for geriatric patients, as outlined in the relevant guidance documents.<br>In conclusion, while the "Use in Specific Populations" section in the Lipitor prescribing information generally complies with the expectations and requirements outlined in the guidance documents, there are some missing components and areas for improvement, particularly regarding the inclusion of data on other types of hyperlipidemia in pediatric patients and more detailed dosing considerations and risk statements for geriatric patients.	Content Requirements: The section includes serious and clinically significant adverse reactions (myopathy and rhabdomyolysis), and risk factors for these reactions, complying with the content requirements of both "Warnings&Precautions.pdf" and "21 CFR 201.57.pdf" guidance.<br>Format Requirements: The section is organized in a clear and informative manner, with each warning and precaution clearly identified and described, meeting the format requirements of both guidance documents.<br>Missing or Inconsistent Components:<br>The Lipitor package insert does not include a BOXED WARNING section. According to the "Warnings&Precautions.pdf" guidance, a boxed warning should be included when necessary. However, without more context, it's unclear whether a boxed warning is necessary for Lipitor.<br>The package insert does not include a CONTRAINDICATIONS section in the Warnings and Precautions section. According to the "Warnings&Precautions.pdf" guidance, situations when the use of the product is contraindicated should be identified in the CONTRAINDICATIONS section. However, this may be present in another section of the label.<br>The package insert does not include the date of the most recent revision of the labeling in the Warnings and Precautions section, which is a requirement according to the "21 CFR 201.57.pdf" guidance. However, this information is present in another section of the label.<br>Please note that the absence of these components in the Warnings and Precautions section does not necessarily mean non-compliance, as they may be present in other sections of the label.
**Alprazolam (XANAX®)**	The "Indications and Usage" section in "Xanax.pdf" states that Xanax is indicated for the acute treatment of generalized anxiety disorder and panic disorder in adults.<br>Compliance Evaluation:<br>General Principles: The indications are supported by substantial evidence of effectiveness based on adequate	The "Use in Specific Populations" section in "Xanax.pdf" includes the following information:<br>Pregnancy: There is a pregnancy exposure registry that monitors pregnancy outcomes in women exposed to XANAX during pregnancy. Healthcare providers are encouraged to register patients. Neonates born to mothers using	Warnings and Precautions from "Warnings and Precautions" section in "Xanax.pdf":<br>Risks from Concomitant Use with Opioids<br>Abuse, Misuse, and Addiction<br>Dependence and Withdrawal Reactions<br>Effects on Driving and Operating Machinery

	and well-controlled studies as required by the guidance document "Indications.pdf". The indications are clearly stated and reflect the scientific evidence accurately.<br>Content: The approved indication, including the applicable population (adults), is clearly communicated as required by "Indications.pdf". The indications are for the acute treatment of generalized anxiety disorder and panic disorder.<br>Format: The "Indications and Usage" section does not explicitly state that it facilitates the indexing of indications in electronic drug databases, as required by "Indications.pdf". However, the clear and concise language used in the section would likely facilitate such indexing.<br>Missing or Inconsistent Components:<br>There are no missing or inconsistent components in the "Indications and Usage" section of "Xanax.pdf" relative to the requirements outlined in the guidance document "Indications.pdf". The section clearly states the approved indication and the applicable population, and it reflects the scientific evidence accurately.<br>The guidance document "21 CFR 201.57.pdf" requires the inclusion of the drug's dosage form, route of administration, and controlled substance symbol in the labeling. However, these requirements are not specific to the "Indications and Usage" section, so they should not be considered missing from this section. The "Indications and Usage" section is not required to contain this information according to the guidance documents.	benzodiazepines late in pregnancy have been reported to experience symptoms of sedation and/or neonatal withdrawal. Patients are instructed to inform their healthcare provider if they are pregnant.<br>Lactation: Breastfeeding is not recommended during treatment with XANAX.<br>Compliance Evaluation:<br>General Principles: The "Use in Specific Populations" section in "Xanax.pdf" complies with the general principles outlined in the "UseinSpecificPopulation.pdf" guidance document. It provides information on the risks associated with using XANAX during pregnancy and lactation, which is in line with the principle of scientific necessity and risk minimization. However, the document does not provide specific requirements for this section, so it's difficult to fully assess compliance.<br>Content Requirements: The section provides adequate information about the risks associated with using XANAX during pregnancy and lactation, which aligns with the content requirements in the "21 CFR 201.57.pdf" guidance document. However, it does not include information about the drug's use in other specific populations such as pediatric, geriatric, or hepatic impairment patients.<br>Format Requirements: The section follows the format requirements outlined in the "21 CFR 201.57.pdf" guidance document. It includes the identifying numbers for each subsection (8.1 for Pregnancy and 8.2 for Lactation) and presents the information in a clear and organized manner.<br>Missing or Inconsistent Components:<br>Missing Information: According to the "21 CFR 201.57.pdf" guidance document, the "Use in Specific Populations" section should include information about the drug's use in other specific populations. In the "Xanax.pdf" document, information about the drug's use in pediatric, geriatric, and hepatic impairment patients is missing from this section.<br>Incomplete Information: The section provides information about the risks associated with using XANAX during pregnancy and lactation, but it does not provide detailed	Interaction with Drugs that Inhibit Metabolism via Cytochrome P450 3A<br>Patients with Depression<br>Mania<br>Neonatal Sedation and Withdrawal Syndrome<br>Risk in Patients with Impaired Respiratory Function<br>Compliance Evaluation:<br>General Principles: The labeling identifies and describes a discrete set of adverse reactions and other potential safety hazards, which aligns with the general principles outlined in the "Warnings&Precautions.pdf" guidance.<br>Content Requirements: The document includes serious or otherwise clinically significant adverse reactions, which is in accordance with the content requirements from the "Warnings&Precautions.pdf" guidance. However, it does not clearly state situations when the use of the product is contraindicated, which is a requirement from the same guidance.<br>Format Requirements: The document is organized in a clear and informative manner, with each warning and precaution listed under a separate subheading, which aligns with the format requirements from the "Warnings&Precautions.pdf" guidance.<br>Missing or Inconsistent Components:<br>Missing Contraindications: The "Warnings and Precautions" section does not clearly state situations when the use of the product is contraindicated, which is a requirement from the "Warnings&Precautions.pdf" guidance (Content Requirements).<br>Missing Boxed Warning: The document does not include a boxed warning, which is a requirement, when necessary, as per the "21 CFR 201.57.pdf" guidance (Regulatory Standards or Criteria).<br>Missing Date of Most Recent Revision: The document does not include the date of the most recent revision of the labeling, which is a requirement from the "21 CFR 201.57.pdf" guidance (Content Requirements).

		information about the potential benefits or the circumstances under which the drug should be used in these populations. This information is required according to the "UseinSpecificPopulation.pdf" guidance document.<br>Inconsistent Information: There are no inconsistencies identified in the "Use in Specific Populations" section in "Xanax.pdf" when compared with the requirements outlined in the relevant guidance documents.	Missing List of Headings and Subheadings: The document does not contain a list of each heading and subheading required in the full prescribing information, which is a requirement from the "21 CFR 201.57.pdf" guidance (Format Requirements).
**Sertraline (ZOLOFT®)**	The "Indications and Usage" section in "Zoloft.pdf" lists the following indications:<br>Major Depressive Disorder (MDD)<br>Obsessive-Compulsive Disorder (OCD)<br>Panic Disorder (PD)<br>Posttraumatic Stress Disorder (PTSD)<br>Social Anxiety Disorder (SAD)<br>Premenstrual Dysphoric Disorder (PMDD)<br>Compliance Evaluation:<br>General Principles: The indications listed are supported by substantial evidence of effectiveness based on adequate and well-controlled studies, as required by the "Indications.pdf" guidance document.<br>Content: The approved indications, including the applicable population, are clearly communicated, meeting the requirements of the "Indications.pdf" guidance document.<br>Format: The "Indications and Usage" section is clearly labeled and the indications are listed in a clear and concise manner, in line with the requirements of the "21 CFR 201.57.pdf" guidance document.<br>Missing or Inconsistent Components:<br>There are no missing or inconsistent components in the "Indications and Usage" section of "Zoloft.pdf" relative to the requirements outlined in the "Indications.pdf" and "21 CFR 201.57.pdf" guidance documents. The section meets all the explicitly stated requirements in the guidance documents.	Use in Specific Populations and Usage from "Use in Specific Populations" section in "Zoloft.pdf":<br>Pregnancy: The document discusses the risk of using Zoloft during pregnancy, including potential risks to the fetus, and advises pregnant women about these risks. It also mentions that Zoloft oral solution contains 12% alcohol and is not recommended during pregnancy.<br>Clinical Considerations: The document discusses the potential risks of untreated depression during pregnancy and postpartum, and the potential neonatal complications from exposure to Zoloft in late pregnancy.<br>Data: The document provides data on the effects of Zoloft exposure in the third trimester of pregnancy, based on post-marketing reports.<br>Compliance Evaluation:<br>General Principles: The Zoloft document does not explicitly mention the principle of scientific necessity or the equitable selection of subjects, as outlined in the "UseinSpecificPopulation.pdf" guidance. However, it does discuss the risks and benefits of using Zoloft during pregnancy, which aligns with the principle of risk minimization.<br>Content Requirements: The Zoloft document provides information on the risks of using Zoloft during pregnancy and the potential neonatal complications, which aligns with the requirement to state the drug's contraindications, warnings and precautions, and adverse reactions from the "21 CFR 201.57.pdf" guidance. However, it does not include provisions for soliciting the assent of children or the	The "Warnings and Precautions" section in "Zoloft.pdf" lists the following warnings and precautions:<br>5.1 Suicidal Thoughts and Behaviors in Pediatric and Young Adult Patients<br>5.2 Serotonin Syndrome<br>5.3 Increased Risk of Bleeding<br>5.4 Activation of Mania or Hypomania<br>5.5 Discontinuation Syndrome<br>5.6 Seizures<br>5.7 Angle-Closure Glaucoma<br>5.8 Hyponatremia<br>5.9 False-Positive Effects on Screening Tests for Benzodiazepines<br>5.10 QTc Prolongation<br>5.11 Sexual Dysfunction<br>Compliance Evaluation:<br>General Principles: The labeling in the "Warnings and Precautions" section is clear, informative, and consistent in content and format, which aligns with the general principles outlined in the "Warnings&Precautions.pdf" guidance document.<br>Content Requirements: The section includes serious or otherwise clinically significant adverse reactions, which meets the content requirements as per the "Warnings&Precautions.pdf" guidance document. The section also includes the drug's contraindications, warnings and precautions, which aligns with the content requirements outlined in the "21 CFR 201.57.pdf" guidance document.

		permission of their parents or guardians, as required by the "UseinSpecificPopulation.pdf" guidance.<br>Format Requirements: The Zoloft document follows the format requirements outlined in the "21 CFR 201.57.pdf" guidance, including the use of headings and subheadings, and the inclusion of the date of the most recent revision.<br>Missing or Inconsistent Components:<br>Missing: The Zoloft document does not include provisions for soliciting the assent of children or the permission of their parents or guardians, as required by the "UseinSpecificPopulation.pdf" guidance (Content Requirements).<br>Incomplete: The Zoloft document does not explicitly mention the principle of scientific necessity or the equitable selection of subjects, as outlined in the "UseinSpecificPopulation.pdf" guidance (General Principles).<br>Misaligned: There are no misaligned components identified in the Zoloft document relative to the requirements outlined in the relevant guidance documents.	Format Requirements: The section is organized in a clear and informative manner with each warning and precaution listed under a separate subheading, which meets the format requirements as per both guidance documents.<br>Missing or Inconsistent Components:<br>The "Warnings and Precautions" section in "Zoloft.pdf" does not appear to have any missing or inconsistent components relative to the requirements outlined in the relevant guidance documents. It includes all the necessary information as per the "Warnings&Precautions.pdf" and "21 CFR 201.57.pdf" guidance documents, and the information is presented in a clear, informative, and consistent manner

Table S2. Results from the RAG system and GPT-4o for the withdrawn drugs.

Drug	Answer from RAG System	Reference Answer from GPT-4o	Scores (%)
Bextra	Bextra was withdrawn from the market due to an unfavorable overall risk versus benefit profile, specifically due to an increased risk of serious adverse cardiovascular events associated with short-term CABG trials. This decision was also influenced by the presence of a boxed warning for serious skin reactions and contraindications for patients with known hypersensitivity to valdecoxib. Bextra failed to comply with FDA regulation for not demonstrating a therapeutic advantage over other NSAIDs and lacking data to support a conclusion that it offered better cardiovascular safety compared to other available agents	Bextra was withdrawn from the market due to safety concerns related to increased risk of cardiovascular events and serious skin reactions. It also failed to comply with FDA regulations regarding proper labeling and the reporting of safety data to the agency.	ROUGE = 37.9<br>METEOR = 54.9
Vioxx	Vioxx was withdrawn from the U.S. market by the sponsor due to safety concerns, specifically a higher incidence of serious cardiovascular thrombotic events compared to patients treated with naproxen. The FDA regulation that Vioxx failed to comply with was related to cardiovascular safety, as evidenced by the findings in the VIGOR study showing an increased risk of myocardial infarction and other serious cardiovascular events in patients using Vioxx. This led to the decision to voluntarily withdraw Vioxx from the market.	Vioxx was withdrawn from the market because it was found to increase the risk of heart attacks and strokes. It failed to comply with FDA regulations regarding the safety and efficacy of the drug. The documents suggest that Merck, the manufacturer of Vioxx, was aware of these risks but failed to adequately disclose them to the public and healthcare professionals, leading to its withdrawal from the market.	ROUGE = 31.1<br>METEOR = 40.1
Rezulin	Rezulin was withdrawn from the market due to severe idiosyncratic hepatocellular injury, including cases of hepatic failure leading to death or liver transplant. The FDA regulation it failed to comply with was related to the safety and effectiveness of the drug. The withdrawal was based on safety concerns regarding hepatotoxicity, specifically severe liver damage experienced by some patients using Rezulin, leading to the decision to remove the drug from the market.	Rezulin was withdrawn from the market due to reports of liver toxicity and failure. It was found to cause severe liver damage in some patients, leading to numerous deaths. Rezulin violated FDA regulations on drug safety and effectiveness, particularly in terms of its potential risks and lack of sufficient warning to healthcare providers and patients.	ROUGE = 33.3<br>METEOR = 44.9

The prompt was: "*Based on the documents DrugName PI.pdf, DrugName Withdrawl.pdf, and DrugName Violated.pdf, why was DrugName withdrawn from the market, and which FDA regulation did it fail to comply with*?"

REFERENCES

(1) Anonymous. *langchain_community.document_loaders.pdf.PyMuPDFLoader*. <https://api.python.langchain.com/en/latest/document_loaders/langchain_community.document_loaders.pdf.PyMuPDFLoader.html> (2024). 2025.

(2) Anonymous. *langchain_text_splitters.character.RecursiveCharacterTextSplitter*. <https://api.python.langchain.com/en/latest/character/langchain_text_splitters.character.RecursiveCharacterTextSplitter.html> (2024). 2025.
