# Supplementary material for: Retrieval Augmented Generation (RAG) for Evaluating Regulatory Compliance of Drug Information and Clinical Trial Protocols
Source: CPT Pharmacometrics Syst Pharmacol. 2026 Feb 19;15(3):e70201. doi: 10.1002/psp4.70201 (PMC12917324; doi:10.1002/psp4.70201)
Supplement: Supplementary file 1 — Data S1: psp470201‐sup‐0001‐Supinfo.zip. [file PSP4-15-e70201-s001.zip › PSP-2025-0274-s04.pdf]

SUPPLEMENTARY FILE S4

INDEPENDENT VERIFICATION

Retrieval Augmented Generation (RAG) for Evaluating Regulatory Compliance of Drug Information and Clinical Trial Protocols

Shreyas Waikar, Amruta Gajanan Bhat, and Murali Ramanathan
Artificial Intelligence & Clinical Pharmacology Laboratory, Department of Pharmaceutical Sciences, University at Buffalo, The State University of New York, Buffalo, NY, USA.

CORRESPONDING AUTHOR: Murali Ramanathan
355 Pharmacy, Department of Pharmaceutical Sciences
University at Buffalo, Buffalo, NY 14214-8033.
(716)-645-4846 and FAX 716-829-6569. E-mail Murali@Buffalo.Edu

Running Head: RAGs for Clinical Pharmacology
Keywords: Artificial Intelligence, AI, LLM, RAG, Pharmacometrics, MIDD, Clinical Pharmacology

INDEPENDENT VERIFICATION

Table S1. The results of the independent verification of the missing or inconsistent components section of HUMIRA® are highlighted in the table below.

Indications and Usage	Use in Specific Populations	Warnings and Precautions
The "Indications and Usage" section in "Humira.pdf" does not explicitly state if Humira is used as an adjunct to a primary mode of therapy, which is a requirement in "21 CFR 201.57.pdf" (Key Requirements for Indications and Usage Sections).<br>**Findings**: Since HUMIRA is the primary mode of therapy this information is missing but not required under 21 CFR 201.57.pdf.<br><br>The section does not describe if the drug is only safe and effective for certain subgroups, nor does it identify any specific tests necessary for patient selection or monitoring. These are requirements in "21 CFR 201.57.pdf" (Key Requirements for Indications and Usage Sections).<br>**Findings**: This is an error. See sections 1.5 and 1.6.<br><br>The section does not state any specific conditions for long-term use, which is a requirement in "21 CFR 201.57.pdf" (Key Requirements for Indications and Usage Sections).<br>**Findings**: There is no section to state any specific conditions for long-term use<br><br>The section does not include any statements comparing the safety or effectiveness of Humira with other agents for the same indications, which is a requirement in "Indications.pdf" (Content Requirements).<br>**Findings**: Comparison of safety and efficacy of Humira with other agents for the same indications is absent in this section. However, the Clinical Studies section contains comparison of safety and efficacy of Humira with Methotrexate.<br><br>The section does not include any information about a common belief about Humira's effectiveness for a certain use, but evidence shows it is ineffective or risky, which is a requirement in "21 CFR 201.57.pdf" (Key Requirements for Indications and Usage Sections).<br>**Findings**: This is an error as it is cleared stated that the effectiveness of Humira is not established.	The section does not explicitly mention the ethical considerations for clinical investigations involving children, which is a requirement outlined in "UseinSpecificPopulation.pdf" (General Principles).<br>The section does not explicitly mention provisions for soliciting the assent of children and the permission of their parents or guardians, which is a requirement outlined in "UseinSpecificPopulation.pdf" (Content Requirements).<br>**Findings**: Ethical considerations and soliciting assent of children are a part of clinical investigation and not to be specified in use in specific population section. This error was noted in the Results section of the manuscript.<br><br>While the section provides information about the use of the drug in specific populations, it does not provide specific information about the use of the drug as an adjunct to a primary mode of therapy, which is a requirement outlined in "21 CFR 201.57.pdf" (Key Requirements for Indications and Usage Sections)<br>**Findings**: This is not relevant to the use in specific populations.	The Warnings and Precautions section in Humira.pdf does not include information on any clinically significant interference with a laboratory test and drug interactions, which is a requirement according to the "Warnings&Precautions.pdf" guidance (Content Requirements).<br>**Findings**: This information is not included in the drug information document (Humira.pdf).<br><br>The section does not use individual subsections to organize the information, nor does it use cross-referencing or emphasis in text to highlight important information, which is suggested by the "Warnings&Precautions.pdf" guidance (Format Requirements).<br>**Findings**: There were eleven subsections and four of these subsections provided cross-references in square brackets.

Table S2. The results of the independent verification of the missing or inconsistent components section of LIPITOR® are highlighted in the table below.

Indications and Usage	Use in Specific Populations	Warnings and Precautions
The section does not explicitly state whether Lipitor is used for the treatment, prevention, mitigation, cure, or diagnosis of the conditions listed, as required by both guidance documents (Indications.pdf: Content Requirements; 21 CFR 201.57.pdf: Key Requirements).<br>**Findings**: Lipitor is used to reduce the risk of heart attack and stroke and as adjunct to diet and exercise.<br><br>It also does not include any limitations of use, additional descriptors or qualifiers as part of the indication, or any statements comparing the safety or effectiveness of Lipitor with other agents for the same indications, as required by the Indications.pdf guidance (Content Requirements).<br>The section does not include any limitations of use, which should be clearly formatted and listed separately according to the Indications.pdf guidance (Format Requirements).<br>**Findings**: The Indications and Usage section does not contain limitations of use.<br><br>There are no misaligned components identified in this analysis.	While data on pediatric use in patients with heterozygous familial hypercholesterolemia is provided, there is no information on the use of Lipitor in other types of hyperlipidemia in pediatric patients, as required by guidance documents.<br>The section could provide more information on dosing considerations and risk statements for geriatric patients, as outlined in the relevant guidance documents.<br>**Findings**: Geriatric Use subsection provides caution but no dosing information.<br><br>In conclusion, while the "Use in Specific Populations" section in the Lipitor prescribing information generally complies with the expectations and requirements outlined in the guidance documents, there are some missing components and areas for improvement, particularly regarding the inclusion of data on other types of hyperlipidemia in pediatric patients and more detailed dosing considerations and risk statements for geriatric patients.<br>**Findings**: Pediatric subsection indicates the safety and effectiveness of LIPITOR in pediatric patients younger than 10 years of age with HeFH or HoFH have not been established.	The Lipitor package insert does not include a BOXED WARNING section. According to the "Warnings&Precautions.pdf" guidance, a boxed warning should be included when necessary. However, without more context, it's unclear whether a boxed warning is necessary for Lipitor.<br>**Findings**: BOXED WARNING section is absent.<br><br>The package insert does not include a CONTRAINDICATIONS section in the Warnings and Precautions section. According to the "Warnings&Precautions.pdf" guidance, situations when the use of the product is contraindicated should be identified in the CONTRAINDICATIONS section. However, this may be present in another section of the label.<br>Findings: CONTRAINDICATIONS is a separate section (see Section 4)<br><br>The package insert does not include the date of the most recent revision of the labeling in the Warnings and Precautions section, which is a requirement according to the "21 CFR 201.57.pdf" guidance. However, this information is present in another section of the label.<br>**Findings**: Revisions are present in the "RECENT MAJOR CHANGES" section on page 1.<br><br>Please note that the absence of these components in the Warnings and Precautions section does not necessarily mean non-compliance, as they may be present in other sections of the label.
