# Supplementary material for: Retrieval Augmented Generation (RAG) for Evaluating Regulatory Compliance of Drug Information and Clinical Trial Protocols
Source: CPT Pharmacometrics Syst Pharmacol. 2026 Feb 19;15(3):e70201. doi: 10.1002/psp4.70201 (PMC12917324; doi:10.1002/psp4.70201)
Supplement: Supplementary file 1 — Data S1: psp470201‐sup‐0001‐Supinfo.zip. [file PSP4-15-e70201-s001.zip › PSP-2025-0274-s05.pdf]

SUPPLEMENTARY FILE S5

PROMPT VARIANTS RESULTS

Retrieval Augmented Generation (RAG) for Evaluating Compliance of Drug Information to FDA Regulatory Guidance Documents

Shreyas Waikar, Amruta Gajanan Bhat, and Murali Ramanathan
Artificial Intelligence & Clinical Pharmacology Laboratory, Department of Pharmaceutical Sciences, University at Buffalo, The State University of New York, Buffalo, NY, USA.

CORRESPONDING AUTHOR: Murali Ramanathan
355 Pharmacy, Department of Pharmaceutical Sciences
University at Buffalo, Buffalo, NY 14214-8033.
(716)-645-4846 and FAX 716-829-6569. E-mail Murali@Buffalo.Edu

Running Head: RAGs for Drug Development
Keywords: Artificial Intelligence, AI, LLM, RAG, Pharmacometrics, MIDD, Clinical Pharmacology

SUPPLEMENTARY METHODS

The impact of prompt variations on the RAG system outcomes for drug information and clinical trial protocol evaluations was examined. Two prompt features were altered: the persona and the inclusion of a fact-checklist to summarize key points.

Prompt Variant 1: The impact of replacing the "*document-restricted assistant*" persona from the drug information evaluation with "*expert at the United States Food and Drug Administration (FDA) with 20 years of regulatory experience reviewing clinical trial protocols*" persona was assessed for HUMIRA® and LIPITOR®. The Indications, Use in Specific Populations, and Warnings and Precautions sections were separately queried

Analogously, we investigated the removal of the persona of an "*expert at the United States Food and Drug Administration (FDA) with 20 years of regulatory experience reviewing clinical trial protocols*" from the clinical trial protocol evaluations (see Supplementary File S1).

Prompt Variant 2: For the fact-checklist prompt variant, the following input was added:

Model instructions:

Produce labeled sections that align with the user query.

After the sections provide a Structured Justification limited to 3–6 bullets that contains only the key verified facts.

Keep output concise: aim for one short paragraph + bullets per section. Use plain language suitable for regulatory reviewers.

SUPPLEMENTARY RESULTS

Effect of Prompt Variants

Drug Information Evaluation: Prompt Variant 1 for HUMIRA® Indications aligned with the original prompt, but it also included limited extraneous Dosage and Administration content as well as compliance notes for elderly and pediatric hazards. Its Use in Specific Populations response did not include the ethical considerations (assent and parental permission) error present in the original response, and it included dosing and safety information belonging to other sections. For Warnings and Precautions, it added detail on laboratory interferences, monitoring, and management, including for pediatric patients with inflammatory bowel disease.

For LIPITOR® Indications, Prompt Variant 1 was largely consistent with the original prompt but provided a more detailed list of approved indications and cited guidance sections (e.g., Indications.pdf p.5, lines 66–70). It incorrectly analyzed "Use in Specific Populations" instead of "Indications and Usage." Warnings and Precautions coverage was more complete, including immune-mediated necrotizing myopathy and liver enzyme monitoring, not noted in the original prompt.

All responses to Prompt Variant 2 included a Structured Justification section with succinct bullet points. For HUMIRA® Indications, Variant 2 was more general and offered a less rigorous compliance assessment than the original prompt. Its Use in Specific Populations response added extraneous material on indications, dosing, and warnings from other label sections and lacked focus on pregnancy, lactation, and pediatrics. For Warnings and

Precautions, however, Variant 2 was more detailed, listing specific infections and pediatric malignancy risks.

Prompt Variant 2 also listed a more complete set of LIPITOR® indications, though its compliance review was less critical than the original. In Use in Special Populations, it omitted pregnancy, lactation, and renal impairment. For Warnings and Precautions, Variant 2 covered additional risks, such as immune-mediated necrotizing myopathy and hepatic dysfunction, while the original prompt provided more detail on myopathy risk factors and drug interactions.

Clinical Trial Protocol Evaluation: Prompt Variant 1 produced Clinical Trial Summary and SAP responses largely similar to the original prompt. However, its response, while concise and operational, omitted details such as the trial's phase (2a), open-label design, and randomization ratio. It correctly identified the primary analysis method (a mixed-model with repeated measures) but provided fewer methodological specifics. Prompt Variant 2 yielded qualitatively similar Clinical Trial Summary and SAP results, and its Structured Justification included document source locations.

As in the original Query 2 response, Prompt Variants 1 and 2 noted limited detail on sample re-estimation methods. However, they did not include the pharmacokinetic or vital-sign analyses present in the original Query 2 response. Prompt Variant 2 supplied a Structured Justification with summary information.

PROMPT VARIANT 1

EFFECTS OF PERSONA

DRUG INFORMATION EVALUATION

1. List the indications and usage from "Indications and Usage" section in "Humira.pdf".
2. Evaluate whether this section complies with the expectations outlined in the guidance documents "Indications.pdf" and "21 CFR 201.57.pdf". Specifically, assess whether the general principles, content, and format requirements are clearly stated.
3. Identify any missing or inconsistent components in 'Humira.pdf' relative to the requirements outlined in the relevant guidance documents. Specify which components are absent, incomplete, or misaligned, and reference the applicable sections of the guidance documents where appropriate.

1. Section Content: The indications and usage for Humira, as stated in the document, include treatment for multiple conditions: rheumatoid arthritis (RA) in adults to reduce symptoms and progression of structural damage; juvenile idiopathic arthritis (JIA) in patients 2 years and older; psoriatic arthritis (PsA) in adults; ankylosing spondylitis (AS) in adults; Crohn's disease (CD) in both adults and pediatric patients 6 years and older; ulcerative colitis (UC) in adults and pediatric patients 5 years and older, with limitations on effectiveness for those intolerant to TNF blockers; plaque psoriasis in adults; hidradenitis suppurativa in patients 12 years and older; and uveitis in adults and pediatric patients 2 years and older.
2. Compliance Evaluation:

- General Principles: The indications and usage section is clear and concise, listing each condition Humira is indicated to treat. It aligns well with the guidance of reflecting scientific evidence and using clinically relevant and valid terminology, making it understandable for health care practitioners.
- Content Requirements: The document meets the requirement to state that the drug is indicated for the treatment of recognized conditions. It includes explicit statements regarding the treatment of specific conditions and limitations of use for patients who have lost response to or were intolerant to TNF blockers.
- Format Requirements: The document utilizes a numbered subsection format for each indication (e.g., 1.1 for Rheumatoid Arthritis), making it easily navigable, which is consistent with the guidance. However, limitations of use should ideally be presented separately under a distinct heading according to the guidance, which is not explicitly done in this document.

3. Missing or Inconsistent Components:

- Missing: There's no explicit separate numbered subsection specifically isolating limitations of use, though they are mentioned within the indications.
- Incomplete: While limitations of use are included in the context of specific conditions, the guidance suggests a separate section to distinctly highlight these, which might not be addressed thoroughly as advised.
- Misaligned: The document could benefit from a distinct separation of limitations of use as specified in the guidance documents, particularly if they apply broadly across multiple indications, to enhance clarity and comply fully with the suggested formatting practices

1. List the Use in Specific Populations from "Use in Specific Populations" section in "Humira.pdf".
2. Evaluate whether this section complies with the expectations outlined in the guidance documents "UseinSpecificPopulation.pdf" and "21 CFR 201.57.pdf". Specifically, assess whether the general principles, content, and format requirements are clearly stated.
3. Identify any missing or inconsistent components in 'Humira.pdf' relative to the requirements outlined in the relevant guidance documents. Specify which components are absent, incomplete, or misaligned, and reference the applicable sections of the guidance documents where appropriate.

1. Section Content: The 'Humira.pdf' document outlines specific populations for which Humira is indicated, including adults and children with rheumatoid arthritis, juvenile idiopathic arthritis, psoriatic arthritis, ankylosing spondylitis, Crohn’s disease, ulcerative colitis, plaque psoriasis, hidradenitis suppurativa, and uveitis. It provides dosage information based on age and weight for conditions such as juvenile idiopathic arthritis and pediatric uveitis, Crohn's disease, and ulcerative colitis.
2. Compliance Evaluation:
    - General Principles: The section is generally clear and concise, listing the specific populations and conditions for which Humira is indicated. It aligns with the general principle outlined in the guidance document regarding clarity in specifying target populations.
    - Content Requirements: The document appears to fulfill the requirement of providing a clear indication of the populations and conditions being treated. It includes limitations of use for patients intolerant to TNF blockers, aligning with the content requirements of specifying indications, limitations of use, and providing dosage based on age and weight.
    - Format Requirements: The section is organized with clear headings for each indication and sub-indication. The separation of sections appears to align with the format requirements detailed in the guidance documents by providing a distinct indication and usage section.
3. Missing or Inconsistent Components:
    - Missing: There are no explicit indications of specific hazards for neonatal or pediatric subgroups, as required if a hazard is associated, which should be referenced from “Warnings and Precautions” as seen in 21 CFR 201.57. There is also no mention of the “Geriatric Use” section, which should be included if there are observed differences or specific requirements for elderly patients.
    - Incomplete: Details on the safety and effectiveness in younger pediatric patients, particularly under the age of 2 for several of its indications, are not thoroughly specified, and the necessary statement that safety and effectiveness are not established should be included.
    - Misaligned: The document does not fully address specific monitoring or dosage adjustments which might be necessary for geriatric use as required by the guidance documents. There is also limited cross-referencing to data specifics as recommended, such as referencing relevant sections within the document that substantiate specific indication claims with clinical evidence

1. List the Warnings and Precautions from "Warnings and Precautions" section in "Humira.pdf".
2. Evaluate whether this section complies with the expectations outlined in the guidance documents "Warnings&Precautions.pdf" and "21 CFR 201.57.pdf". Specifically, assess whether the general principles, content, and format requirements are clearly stated.
3. Identify any missing or inconsistent components in 'Humira.pdf' relative to the requirements outlined in the relevant guidance documents. Specify which components are absent, incomplete, or misaligned, and reference the applicable sections of the guidance documents where appropriate

1. Section Content: In the "Humira.pdf," the warnings and precautions section highlights serious infections and malignancy risks associated with Humira (adalimumab). The risks of serious infections include conditions like tuberculosis (TB), bacterial sepsis, invasive fungal infections, and infections by other opportunistic pathogens, particularly resulting in hospitalization or death. If a serious infection or sepsis develops during treatment, Humira should be discontinued. There is also a requirement to test for latent TB, and if positive, start TB treatment before beginning Humira. Patients need monitoring for active TB even if the initial latent TB test is negative. Moreover, there is an elevated risk of lymphoma and other malignancies, including hepatosplenic T-cell lymphoma (HSTCL), especially in children and adolescents with inflammatory bowel disease.
2. Compliance Evaluation:
   - General Principles: The warning is clear and concise, with a focus on the serious risks of infections and malignancy associated with Humira, aligning with the basic principles of communicating significant safety information. The language used is direct, instructing on what actions to take if infection occurs.
   - Content Requirements: The document does include explicit warning about particular adverse reactions (such as infections and malignancies), but it lacks detailed instructions regarding the differentiation between 'treatment' and 'prevention' for infections. The limitations are not clearly separated; the guidance documents suggest that major limitations of use should be clearly noted, which isn't evident.
   - Format Requirements: The document does appear to meet base formatting requirements by being bolded and inside a box. However, the organization could be improved by providing subheadings for each specific type of risk or instruction, as suggested in the guidance for clarity and visual accessibility.
3. Missing or Inconsistent Components:
   - Missing: In accordance with the guidance, there isn't a clear separation or a distinct subheading for limitations of use. Such separation helps to differentiate and highlight areas where Humira's use is not indicated.
   - Incomplete: Despite covering the major risks, the description of adverse reactions could be more comprehensive by including suggested management or preventive actions for each reaction and providing precise epidemiological information or data as recommended.
   - Misaligned: The section could benefit from a more structured format that includes subheadings for each adverse reaction or caution. For instance, separating the risks and precautions associated with TB, fungal infections, and lymphoma into distinct subsections could align better with format guidance that emphasizes visual clarity and organization

1. List the indications and usage from "Indications and Usage" section in "Lipitor.pdf".
2. Evaluate whether this section complies with the expectations outlined in the guidance documents "Indications.pdf" and "21 CFR 201.57.pdf". Specifically, assess whether the general principles, content, and format requirements are clearly stated.
3. Identify any missing or inconsistent components in 'Lipitor.pdf' relative to the requirements outlined in the relevant guidance documents. Specify which components are absent, incomplete, or misaligned, and reference the applicable sections of the guidance documents where appropriate.

1. Section Content: The "Indications and Usage" section in 'Lipitor.pdf' states the following indications: Lipitor is an HMG-CoA reductase inhibitor (statin) indicated to reduce the risk of myocardial infarction (MI), stroke, revascularization procedures, and angina in adults with multiple risk factors for coronary heart disease (CHD) but without clinically evident CHD; reduce the risk of MI and stroke in adults with type 2 diabetes mellitus with multiple risk factors for CHD but without clinically evident CHD; reduce the risk of non-fatal MI, fatal and non-fatal stroke, revascularization procedures, hospitalization for congestive heart failure, and angina in adults with clinically evident CHD. It is also indicated as an adjunct to diet to reduce low-density lipoprotein (LDL-C) in adults with primary hyperlipidemia, in adults and pediatric patients aged 10 years and older with heterozygous familial hypercholesterolemia (HeFH), and as an adjunct to other LDL-C-lowering therapies to reduce LDL-C in adults and pediatric patients aged 10 years and older with homozygous familial hypercholesterolemia. Additionally, it is indicated as an adjunct to diet for the treatment of adults with primary dysbetalipoproteinemia and hypertriglyceridemia.
2. Compliance Evaluation:
    - General Principles: The Indications and Usage section in 'Lipitor.pdf' is generally clear and concise, aligning with the guidance that recommends using clinically relevant and scientifically valid terminology that is understandable to healthcare practitioners (Indications.pdf, p. 5, line 66-70). The section effectively communicates the uses for which Lipitor has been shown to be safe and effective.
    - Content Requirements: The section includes explicit statements about the conditions Lipitor is indicated to treat and often specifies particular patient populations. It covers treatment purposes, but does not clearly separate limitations of use from the indications, which the guidance suggests including separately if applicable (21 CFR 201.57, section (c)(1)(vi)).
    - Format Requirements: The format follows a bulleted list to present distinct indications, in line with the guidance which allows for bullet points when listing indications (Indications.pdf, p. 9, lines 654-656). However, any specific limitations are not clearly delineated as a separate subsection, as might be preferred when multiple indications exist (Indications.pdf, Sec. D.2).
3. Missing or Inconsistent Components:
    - Missing: The Lipitor document does not seem to have a section explicitly for "Limitations of Use," which the guidance documents suggest should be presented separately, especially if they apply to all indications (Indications.pdf, p. 17, lines 659-663).
    - Incomplete: While the indications include extensive detail about the conditions and subpopulations for which Lipitor is indicated, it lacks comprehensive cross-referencing to other pertinent sections like "Warnings and Precautions" for detailed safety or risk information, as the guidance suggests (21 CFR 201.57, section (c)(1)(ii)).
    - Misaligned: The inclusion of drug interactions or specific patient management directions in the "Indications and Usage" section without clear separation could distract from the focus on the appropriate use of the drug as outlined by the guidance (21 CFR 201.57, section (c)(2)(iv)). Adjustments might be needed to ensure that limitations of use, if applicable beyond contraindications, are clear and easily referenced.

.

1. List the Use in Specific Populations from "Use in Specific Populations" section in "Lipitor.pdf".
2. Evaluate whether this section complies with the expectations outlined in the guidance documents "UseinSpecificPopulation.pdf" and "21 CFR 201.57.pdf". Specifically, assess whether the general principles, content, and format requirements are clearly stated.
3. Identify any missing or inconsistent components in 'Lipitor.pdf' relative to the requirements outlined in the relevant guidance documents. Specify which components are absent, incomplete, or misaligned, and reference the applicable sections of the guidance documents where appropriate.

1. Section Content: The document specifies the use of LIPITOR in certain populations, including as an adjunct to diet to reduce low-density lipoprotein (LDL-C) in adults with primary hyperlipidemia, adults and pediatric patients aged 10 years and older with heterozygous familial hypercholesterolemia (HeFH), and as adjunctive therapy for those with homozygous familial hypercholesterolemia (HoFH). It also mentions the reduction in risk of myocardial infarction, stroke, revascularization procedures, and angina in adults with multiple risk factors for coronary heart disease (CHD) but without clinically evident CHD, and in adults with type 2 diabetes mellitus.
2. Compliance Evaluation:
   - General Principles: The section clearly indicates the conditions for which LIPITOR is indicated, consistent with general FDA guidance principles that require clarity and conciseness. The document aligns with the requirement to describe the population for whom the drug is intended (e.g., adults with primary hyperlipidemia, pediatric patients with HeFH/HoFH).
   - Content Requirements: The content adequately describes indications for treatment and risk reduction, reflecting the requirements for statements regarding treatment of recognized diseases or conditions, prevention, mitigation, and limitations of use when applicable. Although the limitations of use are implied, the document does not explicitly delineate them as separate items or qualify the safety and effectiveness in more specific subpopulations.
   - Format Requirements: The formatting generally follows FDA expectations, with clear section headings like "INDICATIONS AND USAGE" separated from other sections. However, the absence of explicitly separated limitations of use or special population considerations may represent a minor deviation from the ideal organizational format suggested by FDA guidance.
3. Missing or Inconsistent Components:
   - Missing: Clear and separate statements about limitations of use, qualifiers, and explicit safety/effectiveness comparisons for specific subpopulations are not evidently present in the document, despite being a recommended or necessary component according to guidance documents.
   - Incomplete: Elements related to specific guidance on usage among subpopulations, such as more detailed geriatric or pediatric use information, might be considered incomplete since the section does not include information on age-related differences in safety or efficacy, which are required by the 21 CFR 201.57 guidelines.
   - Misaligned: The document could improve alignment with guidance by providing explicit qualifiers and comparisons related to safety and effectiveness between the intended populations versus reference populations, as outlined in the 'Use in Specific Populations' guidance. This would entail explicitly addressing age-related differences or metabolic differences in the pharmacokinetics among subpopulations

1. List the Warnings and Precautions from "Warnings and Precautions" section in "Lipitor.pdf".
2. Evaluate whether this section complies with the expectations outlined in the guidance documents "Warnings&Precautions.pdf" and "21 CFR 201.57.pdf". Specifically, assess whether the general principles, content, and format requirements are clearly stated.
3. Identify any missing or inconsistent components in 'Lipitor.pdf' relative to the requirements outlined in the relevant guidance documents. Specify which components are absent, incomplete, or misaligned, and reference the applicable sections of the guidance documents where appropriate.

1. Section Content: The "Warnings and Precautions" section in the provided "Lipitor.pdf" document includes discussions on Myopathy and Rhabdomyolysis, Immune-Mediated Necrotizing Myopathy (IMNM), and Hepatic Dysfunction. It addresses the risk factors associated with Myopathy and Rhabdomyolysis, conditions under which Lipitor should be discontinued, and the instructions for patient communication regarding these risks. For IMNM, it notes rare reports and advises discontinuation if suspected. Hepatic dysfunction highlights occurrences of increased serum transaminases and rare cases of hepatic failure, advising pre-treatment liver function tests.
2. Compliance Evaluation:
    - General Principles: The section's clarity and conciseness partially align with the general principles outlined in the guidance documents. It provides relevant safety information that can inform patient use but may lack some preventative action details. The structure facilitates understanding but could benefit from more explicit information regarding mitigation strategies.
    - Content Requirements: The section includes explicit statements about conditions under which the drug should be discontinued and identifies potential adverse reactions. However, the emphasis is more on identifying risks than detailing treatment or prevention strategies to mitigate these risks, which is an area the guidance suggests should be included.
    - Format Requirements: The organization of the "Warnings and Precautions" section adheres to a format that identifies risk factors and reactions. There is a clear separation between different types of risks, aligning with the guidelines for organizing by relative clinical significance; however, further emphasis on formatting to distinguish preventative measures could enhance compliance.
3. Missing or Inconsistent Components:
    - Missing: Specific preventative measures associated with the listed warnings are not sufficiently addressed, missing a critical component recommended for inclusion in the "Warnings and Precautions" section.
    - Incomplete: While the document includes necessary warnings, discussions around mitigation strategies for these risks are incomplete. Additionally, comparisons of safety and effectiveness with alternative treatments or interventions are not detailed, which could provide further clinical decision-making context.
    - Misaligned: While largely aligned, more specific details related to actions or changes in therapy to mitigate listed adverse effects are not thoroughly covered, which is encouraged in the guidance to enhance the utility of the information presented

PROMPT VARIANT 2

INCLUSION OF FACT CHECKLIST

DRUG INFORMATION EVALUATION

1. List the indications and usage from "Indications and Usage" section in "Humira.pdf".
2. Evaluate whether this section complies with the expectations outlined in the guidance documents "Indications.pdf" and "21 CFR 201.57.pdf". Specifically, assess whether the general principles, content, and format requirements are clearly stated.
3. Identify any missing or inconsistent components in 'Humira.pdf' relative to the requirements outlined in the relevant guidance documents. Specify which components are absent, incomplete, or misaligned, and reference the applicable sections of the guidance documents where appropriate

1. Section Content: The indications and usage section of Humira, as stated in the document, includes its role as a tumor necrosis factor (TNF) blocker indicated for various conditions. These conditions include Rheumatoid Arthritis (RA), for reducing signs and symptoms, inducing major clinical response, inhibiting the progression of structural damage, and improving physical function in adult patients with moderately to severely active RA. It is also indicated for Juvenile Idiopathic Arthritis (JIA) in patients 2 years of age and older, Psoriatic Arthritis (PsA), Ankylosing Spondylitis (AS), Crohn's Disease (CD) in patients 6 years and older, Ulcerative Colitis (UC) in patients 5 years and older, Plaque Psoriasis (Ps), Hidradenitis Suppurativa (HS), and Uveitis (UV). The document also notes important limitations of use.
2. Compliance Evaluation:
    - General Principles: The section in Humira.pdf is generally clear and concise and uses clinically relevant and scientifically valid terminology that aligns with the guidance, enabling healthcare practitioners to readily identify appropriate therapies.
    - Content Requirements: The section fulfills the requirement of stating approved indications, reflecting the scientific evidence for each condition Humira is intended to treat. Each condition detailed is a recognized disease or condition.
    - Format Requirements: The document uses a structured format with subsection headings for each indication, which aligns with the guidance's preference for organizing multiple indications. However, the document does not indicate whether limitations of use are separately listed as a heading when applicable.
3. Missing or Inconsistent Components:
    - Missing: There is no separate numbered subsection specifically under the heading "Limitations of Use" as recommended when limitations apply to all indications.
    - Incomplete: While the conditions covered are detailed, there may be insufficient cross-references to other labeling sections where necessary, as noted in the Indications guidance for further details about limitations or precautions.
    - Misaligned: The indication section could benefit from a clearer distinction between approved indications and general information, such as warnings or precautions, potentially using cross-references more effectively.

Structured Justification:

- The Humira.pdf lists multiple indications properly using subsections which is consistent with the guidance.
- The document provides clear and specific indications for recognized conditions, reflecting scientific evidence as outlined in guidance.
- Although limitations of use are mentioned, they are not formatted separately with a distinct heading, which deviates from recommended practices.
- The organization supports clarity and conciseness, but might lack robust cross-referencing to precaution sections as advised for comprehensive understanding.
- The content largely aligns with the guidance principles but could improve alignment with format preferences specified for limitations of use

1. List the Use in Specific Populations from "Use in Specific Populations" section in "Humira.pdf".
2. Evaluate whether this section complies with the expectations outlined in the guidance documents "UseinSpecificPopulation.pdf" and "21 CFR 201.57.pdf". Specifically, assess whether the general principles, content, and format requirements are clearly stated.
3. Identify any missing or inconsistent components in 'Humira.pdf' relative to the requirements outlined in the relevant guidance documents. Specify which components are absent, incomplete, or misaligned, and reference the applicable sections of the guidance documents where appropriate

1. Section Content: The document "Humira.pdf" includes a description of the use of HUMIRA for various conditions across different populations, with specific dosages and indications. These include indications for rheumatoid arthritis in adults, juvenile idiopathic arthritis in patients 2 years and older, psoriatic arthritis, ankylosing spondylitis, Crohn's disease, ulcerative colitis, plaque psoriasis, hidradenitis suppurativa, and uveitis. Specific dosage recommendations are detailed for both pediatric and adult populations, outlining starting doses and maintenance doses depending on the condition and patient weight. Furthermore, warnings regarding serious infections, such as tuberculosis, and malignancy risks are prominently included.
2. Compliance Evaluation:
   - General Principles: The section generally aligns with principles of clarity and conciseness, adequately presenting the indications and dosages for each specified condition. The boxed warning section provides crucial safety information prominently, adhering to the requirements for significant risks.
   - Content Requirements: The document covers required content such as indications, usage guidelines, and precautions. However, it is not explicit about specific guidance for populations other than children and adults, such as pregnant women or geriatrics, unless such details are included in other sections not provided in this context.
   - Format Requirements: The organization is somewhat aligned, presenting information orderly, with a clear separation of indications, usage, dosages, and warnings, consistent with standard formatting expectations.
3. Missing or Inconsistent Components:
   - Missing: Potentially absent are fully detailed sections on geriatrics, pregnancy-specific recommendations, and lactation, unless present elsewhere in the documentation.
   - Incomplete: While pediatric recommendations are included, additional data from specific subgroup studies or any differential outcomes across populations may not be fully addressed.
   - Misaligned: The clear identification and discussion of nuances such as specific exposure-response relationships or biomarker applications in pediatric contexts relative to adult data may not be thoroughly described or could be distributed in other supporting documents.

Structured Justification:

- HUMIRA is highlighted for treating various conditions with specific dosing for pediatric patients, following general formatting norms.
- Key content aligns with guidance by including necessary indications and precautions, particularly for serious side effects.
- While dosing information is detailed, specific population subsections, including geriatrics or pregnancy, might lack explicitness.
- Format generally follows guidelines but could improve in integrating nuanced technical data across population groups.
- Missing or incomplete data might include detailed studies' insights on the effects across various subgroups, essential in pediatric evaluations

1. List the Warnings and Precautions from "Warnings and Precautions" section in "Humira.pdf".
2. Evaluate whether this section complies with the expectations outlined in the guidance documents "Warnings&Precautions.pdf" and "21 CFR 201.57.pdf". Specifically, assess whether the general principles, content, and format requirements are clearly stated.
3. Identify any missing or inconsistent components in 'Humira.pdf' relative to the requirements outlined in the relevant guidance documents. Specify which components are absent, incomplete, or misaligned, and reference the applicable sections of the guidance documents where appropriate

1. **Section Content:** The warning and precautions section for HUMIRA outlines the increased risk of serious infections, such as tuberculosis, bacterial sepsis, invasive fungal infections, and infections due to other opportunistic pathogens leading to hospitalization or death. It advises discontinuation of HUMIRA if a patient develops a serious infection or sepsis during treatment. There is a recommendation to test for latent tuberculosis before starting HUMIRA and to monitor all patients for active TB during treatment, even if the initial test is negative. Additionally, there is a warning about the risk of lymphoma and other malignancies in children and adolescents treated with TNF blockers, including HUMIRA, with specific mention of hepatosplenic T-cell lymphoma cases in adolescent and young adults with inflammatory bowel disease.
2. **Compliance Evaluation:**
   - **General Principles:** The section is clearly labeled, concise, and communicates significant risks in a structured manner. It effectively highlights the critical safety concerns, following the guidance to prioritize clinically significant adverse reactions.
   - **Content Requirements:** The warnings include key statements about serious infections and malignancy risks, as explicitly required by the guidance documents. Each warning includes the necessary steps, such as discontinuation of the drug and pre-treatment testing for TB, aligning with the need for specific management strategies.
   - **Format Requirements:** The section is presented with appropriate headings and subheadings, ensuring clarity and accessibility. The format provides a clear hierarchy of information, as recommended in the guidance for structuring the warnings and precautions.
3. **Missing or Inconsistent Components:**
   - **Missing:** The use of bulleted or alternative formats in presenting boxed warnings could enhance visual accessibility. There is no mention of numerical risk estimates for adverse reactions, which should be included if available.
   - **Incomplete:** None of the required elements are only partially addressed; however, regular updates to include any new evidence in risk or administration are necessary to comply with evolving requirements.
   - **Misaligned:** The warnings and precautions do not appear to follow a bulleted format as extensively encouraged for emphasis and clarity in the guidance, potentially affecting how the information is absorbed by readers.

Structured Justification:

- The warnings highlight serious risks such as infections, TB, and malignancies, as recommended.
- Pre-treatment testing and monitoring for TB are aligned with guidance for specific treatment steps.
- Warnings are inappropriately not structured using bulleted formats for emphasis.
- Numerical estimates of risk are not provided, which are advised when relevant data is available.
- The guidance suggests concise but vivid presentation; existing format covers clear hierarchy yet lacks emphasis via bullets.

1. List the indications and usage from "Indications and Usage" section in "Lipitor.pdf".
2. Evaluate whether this section complies with the expectations outlined in the guidance documents "Indications.pdf" and "21 CFR 201.57.pdf". Specifically, assess whether the general principles, content, and format requirements are clearly stated.
3. Identify any missing or inconsistent components in 'Lipitor.pdf' relative to the requirements outlined in the relevant guidance documents. Specify which components are absent, incomplete, or misaligned, and reference the applicable sections of the guidance documents where appropriate.

1. **Section Content**: The indications and usage section in the Lipitor document states that Lipitor (atorvastatin calcium) is indicated for reducing the risk of myocardial infarction (MI), stroke, revascularization procedures, and angina in adults with multiple risk factors for coronary heart disease but without clinically evident coronary heart disease. It is also indicated for reducing MI and stroke in adults with type 2 diabetes mellitus with multiple risk factors for CHD but without clinically evident CHD. In addition, it is indicated for non-fatal MI, fatal and non-fatal stroke, revascularization procedures, hospitalization for congestive heart failure, and angina in adults with clinically evident CHD. Lipitor also serves as an adjunct to diet for reducing LDL-C in adults with primary hyperlipidemia and in adults and pediatric patients aged 10 years and older with heterozygous familial hypercholesterolemia. It is likewise used as an adjunct to other LDL-C-lowering therapies for adults and pediatric patients aged 10 years and older with homozygous familial hypercholesterolemia, and for the treatment of primary dysbetaliproteinemia and hypertriglyceridemia in adults.
2. **Compliance Evaluation**:
   - **General Principles**: The indications and usage section is clear and concise, aligning with the guidance for providing information that is scientifically valid and readily understandable by healthcare practitioners. It accurately reflects the conditions Lipitor is approved to treat without any unnecessary or ambiguous information.
   - **Content Requirements**: The guidance document states that the indications section must clearly state the recognized diseases and conditions the drug is intended for and must be supported by substantial evidence. Lipitor's indications in the document align with this requirement, as they specifically mention the diseases and conditions that Lipitor targets.
   - **Format Requirements**: The document follows a bulleted format to list multiple indications clearly, which aligns with the guidance about presenting distinct indications when a drug is approved for multiple uses. This format helps convey the information cleanly and understandably without confusion or excessive complexity.
3. **Missing or Inconsistent Components**:
   - **Missing**: The document does not appear to be missing any of the primary contents regarding indications, as it comprehensively covers all necessary uses Lipitor is approved for.
   - **Incomplete**: At this section detail level, there are no indications of incomplete elements in need of additional information or clarification.
   - **Misaligned**: There do not appear to be instances of misalignment with the guidance requirements in the Indications and Usage section insofar as it adheres to expectations for clarity, format, and content precision.

Structured Justification:

- Lipitor's indications section provides clear and direct statements on the recognized conditions it is approved to treat.
- The section uses a bulleted format for multiple indications, enhancing clarity and adherence to guidance document expectations.
- Indications are presented concisely, aligning with guidance specifications for clarity and relevance.
- There are no apparent omissions or misalignments in stated indications relative to guidance document expectations.
- The document correctly includes scientifically validated use terms in its indications section, enhancing its applicability to healthcare professionals

1. List the Use in Specific Populations from "Use in Specific Populations" section in "Lipitor.pdf".
2. Evaluate whether this section complies with the expectations outlined in the guidance documents "UseinSpecificPopulation.pdf" and "21 CFR 201.57.pdf". Specifically, assess whether the general principles, content, and format requirements are clearly stated.
3. Identify any missing or inconsistent components in 'Lipitor.pdf' relative to the requirements outlined in the relevant guidance documents. Specify which components are absent, incomplete, or misaligned, and reference the applicable sections of the guidance documents where appropriate.

1. Section Content: The document outlines several specific populations and related dosages: Adults with primary hyperlipidemia and adults and children over 10 years with familial hypercholesterolemia, both indicated for lowering LDL-C, adults with primary dysbetaliproteinemia and hypertriglyceridemia, and reductions in risk of myocardial infarction, stroke, and angina in individuals with coronary heart disease, and for use as an adjunct with other therapies.
2. Compliance Evaluation:
    - General Principles: The document presents information concisely and in alignment with the general directive for clear labeling as per FDA guidance.
    - Content Requirements: The labeling includes specific indications and recommended dosages for both adults and pediatric populations, which is consistent with the content requirements under 21 CFR 201.57, ensuring proper patient information.
    - Format Requirements: The document follows an organized structure with clear subheadings under specific populations, similar to guidelines provided by 21 CFR 201.57.
3. Missing or Inconsistent Components:
    - Missing: The document lacks detailed information on the use in populations with renal or hepatic impairment, which is typically required under specific conditions by 21 CFR 201.57.
    - Incomplete: While dosage forms and strengths are mentioned, further detailed considerations for specific population needs such as geriatrics or lactating women are not thoroughly discussed as might be expected.
    - Misaligned: The document does not visibly misalign with the guidance but could benefit from additional safety data and usage clarification similar to the recommendations in "UseinSpecificPopulation.pdf".

Structured Justification:

- The document provides detailed dosage recommendations for different population groups, aligning well with standardized labeling requirements under 21 CFR 201.57.
- It is organized using clear headings and subheadings that distinguish different use cases, enhancing readability and clarity in alignment with FDA expectations.
- Comprehensive population-specific information such as geriatric use or specialized monitoring needs is insufficient, highlighting areas needing improvement per guidance.
- Information on special population requirements could be expanded, especially regarding safety profile specifics as discussed in "UseinSpecificPopulation.pdf".
- Clarity of section organization helps to meet general FDA principles regarding effective drug labeling, ensuring key details are accessible to prescribers

1. List the Warnings and Precautions from "Warnings and Precautions" section in "Lipitor.pdf".
2. Evaluate whether this section complies with the expectations outlined in the guidance documents "Warnings&Precautions.pdf" and "21 CFR 201.57.pdf". Specifically, assess whether the general principles, content, and format requirements are clearly stated.
3. Identify any missing or inconsistent components in 'Lipitor.pdf' relative to the requirements outlined in the relevant guidance documents. Specify which components are absent, incomplete, or misaligned, and reference the applicable sections of the guidance documents where appropriate

1. Section Content: The warnings and precautions section of the Lipitor document contains information on myopathy and rhabdomyolysis, immune-mediated necrotizing myopathy, and hepatic dysfunction. It advises discontinuation of Lipitor if elevated CK levels occur or myopathy is suspected. The section also mentions the risk factors for myopathy and suggests monitoring liver enzymes due to potential hepatotoxicity.
2. Compliance Evaluation:
    - General Principles: The section generally aligns with the guidance on clarity and conciseness, providing specific instructions regarding drug discontinuation and monitoring requirements. It follows the recommendation of listing the most serious risks associated with Lipitor.
    - Content Requirements: The content includes clinically significant adverse reactions such as myopathy and hepatic dysfunction, aligning with guidelines. However, more detailed structuring or numbering as required might not be explicitly followed.
    - Format Requirements: The format tries to maintain a clear structure with sections dedicated to specific risks. However, the use of subsections or headings does not completely follow the specific formatting techniques of numbering or bolding as recommended by the guidance documents.
3. Missing or Inconsistent Components:
    - Missing: The guidance suggests that headings and subheadings should be appropriately formatted and that information should be presented with clarity and structure, which might not be fully adhered to in the document provided.
    - Incomplete: The section covers major risks but might benefit from more explicit instruction regarding monitoring recommendations to prevent severe outcomes, which are implied but not always explicitly addressed by specific guideline-paragraph references.
    - Misaligned: The guidance suggests using specific subsection titles and consistent formatting with bold or numbered headings, which might not be fully aligned with the layout of the Lipitor document.

Structured Justification:

- The section on Lipitor warnings and precautions includes key adverse reactions such as myopathy, rhabdomyolysis, and hepatic dysfunction.
- The document provides specific advice on discontinuation and monitoring requirements, offering clear risk mitigation strategies.
- General principles of clarity and structure appear to be followed, yet explicit formatting guidelines as per the guidance might not be fully adhered to.
- There is a need for improved alignment with specific formatting requirements, such as numbering and bolding subsection titles.
- Although the section achieves clarity in conveying warnings, organizing content per exact regulatory requirements could enhance compliance.

PROMPT VARIANT 1

EFFECTS OF PERSONA

CLINICAL TRIAL PROTOCOL EVALUATION

Prompt Variant 1

User:

Extract and summarize the clinical trial protocol and statistical analysis plan of the given clinical trial.

Assistant:

Clinical Trial Protocol Summary

- **Study Design:**
- The number of participants per treatment arm was adjusted from 20 to a range of 12-20 participants.
- The study comprises up to 5 cohorts, with 9-15 participants receiving GSK3036656 and 3-5 receiving standard-of-care (SoC) regimen for drug-sensitive tuberculosis (DS-TB).

  Cohorts will proceed sequentially from A through E.

  Study Objectives and Endpoints:

- The primary objective is to investigate the early bactericidal activity, safety, and tolerability of GSK3036656 in participants with drug-sensitive pulmonary tuberculosis.

  Primary and secondary efficacy and safety endpoints will be assessed, with microbiological efficacy/activity assessments reported separately.

  Participant and Study Completion:

  Participants whose respiratory sputum cultures are contaminated may be replaced to ensure critical data for study analysis is not lost.

  Changes Due to COVID-19:

- Additional analyses and safety displays have been added to understand the impact of the COVID-19 pandemic on study conduct and results.

Statistical Analysis Plan Summary

- **Statistical Methodology:**
- A mixed model with repeated measures analysis is the primary analysis for the primary endpoint.
- Dose has been replaced by treatment, and BMI has been added as a covariate.

No formal interim analyses are planned, but safety, tolerability, and pharmacokinetic data will be reviewed before each dose escalation.

Planned Analyses:

- Primary efficacy analyses will use descriptive statistics and graphical presentations.
- Safety data will be summarized descriptively according to GSK's Integrated Data Standards Library (IDSL) standards.

No subgroup analyses are planned for the primary endpoint.

Protocol Deviations:

Important protocol deviations, including those related to COVID-19, will be summarized and listed.

Sample Size Determination:

- Sample size re-estimation was conducted due to lower than anticipated variability in the rate of change of log CFU and slower recruitment.

Regulatory Guidance: - Not specified.

Evaluation Metrics

Metric	Score (%)	Reason

Answer Relevancy	95.45	The score is 0.95 because the output effectively summarizes the clinical trial protocol and statistical analysis plan, addressing the core of the input request. However, the mention of regulatory guidance not being specified slightly detracts from the focus, preventing a perfect score.
Faithfulness	100.0	The score is 1.00 because there are no contradictions, indicating a perfect alignment between the actual output and the retrieval context. Great job maintaining accuracy and consistency!
ClinPharm Metric	84.64	The actual output aligns well with clinical pharmacology principles and includes relevant details on study design, objectives, and statistical methods, which are crucial for drug development. However, it omits specific regulatory guidance details, which are mentioned in the retrieval context.

User:

Based on the FDA E9 Statistical Principles for Clinical Trials, evaluate the statistical analysis plan of the clinical trial protocol.

Assistant:

1. Statistical Methodology Specification

- **Alignment with ICH E9 Principles:**
- The protocol specifies the use of a mixed model with repeated measures analysis for the primary endpoint, which aligns with ICH E9's emphasis on pre-specifying statistical methods.

  Inclusion of covariates such as BMI and treatment-by-day interaction reflects ICH E9's guidance on considering relevant factors in the analysis.

  Comment:

  The protocol does not clearly specify how missing data will be handled, which is a critical aspect under ICH E9.

Improvement:

- [High] Include a detailed plan for handling missing data, specifying methods such as multiple imputation or last observation carried forward, to ensure robustness and compliance with ICH E9.

2. Strategy for Intercurrent (Post-Randomization) Events

- **Alignment with ICH E9 Principles:**

  The protocol includes a strategy for handling intercurrent events, aiming to estimate the treatment effect while on treatment, which aligns with the ICH E9 focus on defining estimands.

  Comment:

  The strategy lacks detail on how different types of intercurrent events will be addressed, such as treatment discontinuation or protocol deviations.

  Improvement:

- [Medium] Provide a more detailed description of how each type of intercurrent event will be managed in the analysis, ensuring clarity and adherence to ICH E9 guidelines.

3. Sample Size Determination

- **Alignment with ICH E9 Principles:**

  The protocol includes a section on sample size re-estimation, which is consistent with ICH E9's guidance on ensuring adequate power and addressing variability.

  Comment:

  The rationale for the original and re-estimated sample sizes is not fully detailed, particularly regarding the assumptions and calculations used.

  Improvement:

- [High] Include comprehensive details on the assumptions and statistical calculations underlying both the original and re-estimated sample sizes to enhance transparency and compliance with ICH E9.

4. Safety Analyses

- **Alignment with ICH E9 Principles:**

  Safety analyses are planned based on the Safety population, aligning with ICH E9's emphasis on pre-specifying analysis populations.

  Comment:

  The protocol does not specify how adverse events will be categorized or analyzed beyond descriptive statistics.

  Improvement:

- [Medium] Specify the use of a common adverse event dictionary, such as MedDRA, and outline the statistical methods for analyzing safety data, including any subgroup analyses, to align with ICH E9 recommendations.

5. Interim Analyses

- **Alignment with ICH E9 Principles:**

  The protocol states that no formal interim analyses are planned, which is consistent with ICH E9's guidance on pre-specifying interim analysis plans.

  Comment:

  The protocol should clarify the criteria for any potential unplanned interim analyses, especially in response to safety concerns.

  Improvement:

- [Low] Provide criteria and procedures for conducting unplanned interim analyses, ensuring that any such analyses are conducted in a manner consistent with ICH E9 principles.

6. Handling of Protocol Deviations

- **Alignment with ICH E9 Principles:**

Protocol deviations are tracked and summarized, which aligns with ICH E9's emphasis on documenting and addressing deviations.

Comment:

The protocol does not specify how protocol deviations will impact the analysis set or the interpretation of results.

Improvement:

- [Medium] Clarify the impact of protocol deviations on the analysis set and results interpretation, ensuring that any potential biases are minimized as per ICH E9 guidance.

7. Exploratory Efficacy Analyses

Not specified.

Comment:

The protocol lacks detail on exploratory efficacy analyses, which could provide additional insights into the treatment effect.

Improvement:

- [Low] Include a section on exploratory efficacy analyses, specifying the methods and objectives, to enhance the understanding of the treatment effect beyond the primary and secondary endpoints.

Evaluation Metrics

Metric	Score (%)	Reason
Answer Relevancy	100.0	The score is 1.00 because the output perfectly aligns with the input request, thoroughly evaluating the statistical analysis plan based on the FDA E9 Statistical Principles for Clinical Trials. There are no irrelevant statements, showcasing a clear and focused response.

Faithfulness	100.0	The score is 1.00 because there are no contradictions, indicating a perfect alignment between the actual output and the retrieval context. Great job maintaining accuracy and consistency!
ClinPharm Metric	69.94	The output aligns with ICH E9 principles and addresses key areas like statistical methodology and intercurrent events, relevant to drug development. However, it omits details on handling missing data, intercurrent events, and sample size rationale, which are critical under ICH E9.

PROMPT VARIANT 2

INCLUSION OF FACT CHECKLIST

CLINICAL TRIAL PROTOCOL EVALUATION

Prompt Variant 2

User:

Extract and summarize the clinical trial protocol and statistical analysis plan of the given clinical trial.

Assistant:

Clinical Trial Protocol Summary

- **Study Design**: The study is a Phase IIa open-label trial investigating the early bactericidal activity, safety, and tolerability of GSK3036656 in participants with drug-sensitive pulmonary tuberculosis. The number of participants per treatment arm has been adjusted from 20 to a range of 12–20.
- **Participant Allocation**: Up to 5 cohorts are planned, with 12–20 participants in each cohort. The allocation is 9–15 participants receiving GSK3036656 and 3–5 receiving standard-of-care (SoC) treatment.
- **Study Phases**: The study will proceed sequentially through cohorts A to E.
- **Primary Endpoint**: The primary endpoint involves the analysis of early bactericidal activity (EBA) using a mixed model with repeated measures. Treatment replaces dose as a factor, and BMI is included as a covariate.
- **COVID-19 Adjustments**: Additional analyses and safety displays have been added to account for the impact of the COVID-19 pandemic on study conduct and results.

Statistical Analysis Plan Summary

- **Primary Analysis**: A mixed model with repeated measures is used for the primary endpoint analysis. This model includes treatment as a factor and BMI as a covariate.
- **Secondary Analyses**: Safety data will be presented in tabular and graphical formats, summarized descriptively. Adverse events will be coded using MedDRA and summarized by severity and relationship to treatment.
- **Interim Analyses**: No formal interim analyses are planned, but safety, tolerability, and pharmacokinetic data will be reviewed before each dose escalation.

- **Sensitivity Analyses**: A sensitivity analysis will exclude participants who withdraw before Day 10 or are replaced during the study.
- **Exploratory Analyses**: Additional covariates will be explored using a mixed model repeated measures analysis.

Structured Justification

- The study design and participant allocation details are derived from protocol amendments and changes specified in the document.
- The primary endpoint and statistical methods are explicitly mentioned in the statistical methodology section.
- Adjustments for COVID-19 are noted in the protocol changes and additional analyses sections.
- The absence of formal interim analyses is confirmed by the planned analyses section.
- Sensitivity and exploratory analyses are detailed in the statistical analysis methods section.

Evaluation Metrics

Metric	Score (%)	Reason
Answer Relevancy	100.0	The score is 1.00 because the output perfectly aligns with the input request, providing a clear and concise summary of the clinical trial protocol and statistical analysis plan without any irrelevant information. Great job!
Faithfulness	100.0	The score is 1.00 because there are no contradictions, indicating a perfect alignment between the actual output and the retrieval context. Great job maintaining accuracy and consistency!
ClinPharm Metric	88.39	The actual output aligns well with clinical pharmacology principles and includes relevant details on study design, participant allocation, and statistical methods, as confirmed by the retrieval context. It addresses COVID-19 adjustments and omits no significant details, making it highly relevant to drug development.

User:

Based on the FDA E9 Statistical Principles for Clinical Trials, evaluate the statistical analysis plan of the clinical trial protocol.

Assistant:

Statistical Methodology Specification

- **Alignment with ICH E9 Principles:**
- The protocol specifies the use of a mixed model with repeated measures analysis for the primary endpoint, which aligns with ICH E9's emphasis on pre-specifying statistical methods.

  Inclusion of covariates such as BMI and treatment-by-day interaction reflects consideration of potential confounding factors, consistent with ICH E9 guidance on model specification.

  Comment:

  The protocol does not clearly specify how missing data will be handled, which is a critical aspect of statistical analysis as per ICH E9.

  Improvement:

- [High] Include a detailed plan for handling missing data, specifying methods such as multiple imputation or last observation carried forward, to ensure robustness of the analysis.

Strategy for Intercurrent Events

- **Alignment with ICH E9 Principles:**

  The protocol plans to estimate the treatment effect while on treatment, which is consistent with ICH E9's guidance on defining estimands that reflect the treatment effect of interest.

  Comment:

  The strategy for handling intercurrent events is not fully detailed, particularly regarding how these events will be incorporated into the analysis.

  Improvement:

- [Medium] Provide a comprehensive strategy for handling intercurrent events, including examples of potential events and their impact on the analysis, to align with ICH E9's emphasis on clear estimand definition.

Sample Size Determination

- **Alignment with ICH E9 Principles:**

  The protocol includes a section on sample size re-estimation, which is in line with ICH E9's recommendation to ensure adequate power and address variability in trial data.

  Comment:

  The rationale for the initial sample size and the criteria for re-estimation are not fully detailed.

  Improvement:

- [Medium] Clearly document the assumptions and calculations used for the initial sample size determination and the criteria for re-estimation to enhance transparency and reproducibility.

Safety Analyses

- **Alignment with ICH E9 Principles:**

  Safety analyses are planned to be based on the Safety population, which aligns with ICH E9's guidance on defining analysis sets.

  Comment:

  The protocol lacks detail on how adverse events will be categorized and analyzed, which is crucial for regulatory assessment.

  Improvement:

- [Low] Include detailed procedures for categorizing and analyzing adverse events, using a standardized dictionary like MedDRA, to ensure consistency and clarity in safety reporting.

Exploratory Efficacy Analyses

- **Alignment with ICH E9 Principles:**

  The protocol includes exploratory analyses, which can provide additional insights into treatment effects, as recommended by ICH E9.

  Comment:

  The specific exploratory analyses planned are not detailed, which may limit the ability to interpret these findings.

  Improvement:

- [Low] Specify the exploratory analyses to be conducted, including the endpoints and statistical methods, to enhance the interpretability of these analyses.

Structured Justification

- The protocol specifies a mixed model with repeated measures for the primary endpoint, aligning with ICH E9's emphasis on pre-specification.
- Strategy for intercurrent events is outlined but lacks detail, which is critical for defining estimands.
- Sample size re-estimation is included, consistent with ICH E9's guidance on ensuring adequate power.
- Safety analyses are based on the Safety population, aligning with ICH E9's recommendations.
- Exploratory analyses are planned but not detailed, which could limit interpretability.

Evaluation Metrics

Metric	Score (%)	Reason
Answer Relevancy	100.0	The score is 1.00 because the output perfectly aligns with the input request, thoroughly evaluating the statistical analysis plan based on the FDA E9 Statistical Principles for Clinical Trials. There are no irrelevant statements, showcasing a clear and focused response.

Faithfulness	100.0	The score is 1.00 because there are no contradictions, indicating a perfect alignment between the actual output and the retrieval context. Great job maintaining accuracy and consistency!
ClinPharm Metric	72.9	The actual output aligns with ICH E9 principles and addresses key areas like statistical methodology, intercurrent events, and sample size, relevant to drug development. However, it omits details on handling missing data, intercurrent events, and exploratory analyses, which are critical for comprehensive evaluation.
