# Supplementary material for: Retrieval Augmented Generation (RAG) for Evaluating Regulatory Compliance of Drug Information and Clinical Trial Protocols
Source: CPT Pharmacometrics Syst Pharmacol. 2026 Feb 19;15(3):e70201. doi: 10.1002/psp4.70201 (PMC12917324; doi:10.1002/psp4.70201)
Supplement: Supplementary file 1 — Data S1: psp470201‐sup‐0001‐Supinfo.zip. [file PSP4-15-e70201-s001.zip › PSP-2025-0274-s06.pdf]

SUPPLEMENTARY FILE S6

GPT-4o RESULTS

Retrieval Augmented Generation (RAG) for Evaluating Compliance of Drug Information to FDA Regulatory Guidance Documents

Shreyas Waikar, Amruta Gajanan Bhat, and Murali Ramanathan
Artificial Intelligence & Clinical Pharmacology Laboratory, Department of Pharmaceutical Sciences, University at Buffalo, The State University of New York, Buffalo, NY, USA.

CORRESPONDING AUTHOR: Murali Ramanathan
355 Pharmacy, Department of Pharmaceutical Sciences
University at Buffalo, Buffalo, NY 14214-8033.
(716)-645-4846 and FAX 716-829-6569. E-mail Murali@Buffalo.Edu

Running Head: RAGs for Drug Development
Keywords: Artificial Intelligence, AI, LLM, RAG, Pharmacometrics, MIDD, Clinical Pharmacology

GPT-4o OUTPUTS
INDICATIONS AND USAGE QUERY 1

Q. 1. List the indications and usage from "Indications and Usage" section in "Lipitor.pdf". 2. Evaluate whether this section complies with the expectations outlined in the relevant FDA guidance documents. Specifically, assess whether the general principles, content, and format requirements are clearly stated. 3. Identify any missing or inconsistent components relative to the requirements outlined in the relevant guidance documents. Specify which components are absent, incomplete, or misaligned, and reference the applicable sections of the guidance documents where appropriate.

Section Content: Lipitor is indicated to reduce the risk of myocardial infarction (MI), stroke, revascularization procedures, and angina in adults with multiple risk factors for coronary heart disease (CHD) but without clinically evident CHD; to reduce the risk of MI and stroke in adults with type 2 diabetes mellitus with multiple risk factors for CHD but without clinically evident CHD; and to reduce the risk of non-fatal MI, fatal and non-fatal stroke, revascularization procedures, hospitalization for congestive heart failure, and angina in adults with clinically evident CHD. It is also indicated as an adjunct to diet to reduce low-density lipoprotein cholesterol (LDL-C) in adults with primary hyperlipidemia and in adults and pediatric patients aged 10 years and older with heterozygous familial hypercholesterolemia (HeFH). Additionally, it may be used as an adjunct to other LDL-C-lowering therapies to reduce LDL-C in patients with homozygous familial hypercholesterolemia (HoFH), and for the treatment of adults with primary dysbetalipoproteinemia and hypertriglyceridemia.

Compliance Evaluation:

General Principles: Lipitor's indications are clearly stated and population-specific. Each clinical scenario aligns with known pathophysiology and uses terminology consistent with standard treatment guidelines. Language separates prevention from treatment uses effectively.

Content Requirements: The section addresses clinically meaningful endpoints like MI, stroke, and LDL-C reduction. Indications identify appropriate risk populations. Pediatric inclusion is clearly limited by age. However, treatment purpose language could be more explicit for lipid disorders.

Format Requirements: The structure is segmented and organized, but lacks an explicit "Limitations of Use" section. Bulleted formatting is used in highlights, while full labeling uses paragraph structure with sub-indents.

Missing or Inconsistent Components:

Missing: No limitations of use are stated. There is no mention of non-indicated populations or conditions where Lipitor has not been shown to be effective.

Incomplete: Treatment language for metabolic indications such as hypertriglyceridemia and dysbetalipoproteinemia lacks direct phrasing like "to treat," instead relying solely on "as adjunct to diet."

Misaligned: Population criteria are mostly appropriate, but certain language (e.g., "to reduce LDL-C in adults with primary hyperlipidemia") could be interpreted as biomarker-focused rather than outcome-focused, which FDA typically discourages in favor of clinical benefit framing.

GPT-4o OUTPUTS
INDICATIONS AND USAGE QUERY 1

Q. 1. List the indications and usage from "Indications and Usage" section in "Xanax.pdf". 2. Evaluate whether this section complies with the expectations outlined in the relevant FDA guidance documents. Specifically, assess whether the general principles, content, and format requirements are clearly stated. 3. Identify any missing or inconsistent components relative to the requirements outlined in the relevant guidance documents. Specify which components are absent, incomplete, or misaligned, and reference the applicable sections of the guidance documents where appropriate.

Section Content: Xanax is indicated for the management of anxiety disorder or for the short-term relief of symptoms of anxiety in adults. It is also indicated for the treatment of panic disorder, with or without agoraphobia. The labeling clarifies that anxiety or tension associated with the stress of everyday life usually does not require treatment with an anxiolytic. Xanax's effectiveness in long-term use (i.e., more than 4 months) has not been systematically evaluated in clinical trials. The continuation of therapy should be periodically reassessed.

Compliance Evaluation:

General Principles: The section presents a clear therapeutic purpose, target populations, and conditions for which Xanax is used. It distinguishes between appropriate clinical indications and non-indicated uses (e.g., everyday stress), aligning with the FDA's requirement for clarity and precision in scope. The indication for panic disorder is clearly defined, and guidance is given on limitations for long-term use.

Content Requirements: The section explicitly mentions the population (adults), conditions (anxiety disorder and panic disorder), and distinguishes symptom relief versus disorder-level diagnosis. It meets content criteria by describing scope of use, relevant limitations, and periodic reassessment. However, the language could be more explicit regarding the risks of long-term use, abuse potential, and criteria for continuation of therapy.

Format Requirements: The format follows a concise narrative style appropriate for FDA labeling, but lacks sub-bullets or visual hierarchy that could make distinctions between indications clearer. Duration limitations and non-indicated conditions are appropriately included. The absence of additional formatting does not violate guidance but could reduce clarity in some contexts.

Missing or Inconsistent Components:

Missing: There is no formal "Limitations of Use" subheading, although limitations are mentioned in the paragraph. A distinct label would better conform to FDA formatting expectations and help highlight non-indicated use cases more clearly.

Incomplete: The long-term use caution is present, but lacks detail about the rationale (e.g., dependency, tolerance). While the indication is clear, the risks associated with long-term benzodiazepine use are not explicitly referenced here, which FDA often recommends be at least cross-referenced.

Misaligned: The indication for "short-term relief of symptoms of anxiety" could be interpreted broadly without additional context on diagnostic thresholds, potentially inviting off-label or casual prescribing, which FDA labeling aims to minimize.

GPT-4o OUTPUTS
SPECIFIC POPULATIONS QUERY 2

Q. 1. List the use in specific population from "Use in Specific Populations" section in "Lantus.pdf". 2. Evaluate whether this section complies with the expectations outlined in the relevant FDA guidance documents. Specifically, assess whether the general principles, content, and format requirements are clearly stated. 3. Identify any missing or inconsistent components relative to the requirements outlined in the relevant guidance documents. Specify which components are absent, incomplete, or misaligned, and reference the applicable sections of the guidance documents where appropriate.

Section Content: The "Use in Specific Populations" section of Lantus includes considerations for pregnant and lactating women, pediatric patients, and geriatric patients. For pregnancy, the labeling notes that available data from published studies and postmarketing reports have not established a drug-associated risk of major birth defects or miscarriage. For lactation, there is mention of insulin being present in human milk, with no adverse effects observed in breastfed infants. In pediatric patients, Lantus is indicated for children aged six years and older with type 1 diabetes mellitus, with a caution that efficacy and safety have not been established in children younger than six years. For geriatric patients, the section acknowledges the increased risk of hypoglycemia due to age-related renal function decline and recommends cautious dosing and close monitoring.

Compliance Evaluation:

General Principles: The section demonstrates clear population-specific information, consistent with the principles outlined in 21 CFR 201.57(c)(9). Each population category is addressed separately, with clinical considerations based on pharmacologic and safety profiles relevant to each demographic.

Content Requirements: Appropriate data sources (postmarketing, published literature) are cited for pregnancy and lactation risk assessments. Pediatric use includes specific age cutoffs and acknowledges gaps in evidence for younger populations. Geriatric use reflects increased sensitivity to hypoglycemic events and the need for renal function monitoring. Risk-benefit discussions are present but do not overstate safety where data are limited.

Format Requirements: The labeling follows structured subsections (8.1, 8.2, 8.3, etc.), with bolded headings and consistent formatting. Each population group is clearly delineated and organized in the order recommended by FDA guidance. Language is non-promotional, informative, and safety-oriented.

Missing or Inconsistent Components:

Missing: There is no mention of data regarding reproductive potential in males or females (as covered in newer FDA guidelines under "Females and Males of Reproductive Potential"), though this may not apply to insulin analogs.

Incomplete: While pediatric safety is addressed, the section could be enhanced by explicitly mentioning the lack of long-term safety data in pediatric populations, particularly around growth or development effects, which FDA encourages when applicable.

Misaligned: The section does not clearly describe how pregnancy category labeling has been replaced with narrative risk summaries under the PLLR format, which may cause confusion for clinicians referencing older classification systems

GPT-4o OUTPUTS
WARNINGS AND PRECAUTIONS QUERY 3

Q. 1. List the Warnings and Precautions from "Warnings and Precautions" section in "Humira.pdf". 2. Evaluate whether this section complies with the expectations outlined in the relevant FDA guidance documents. Specifically, assess whether the general principles, content, and format requirements are clearly stated. 3. Identify any missing or inconsistent components relative to the requirements outlined in the relevant guidance documents. Specify which components are absent, incomplete, or misaligned, and reference the applicable sections of the guidance documents where appropriate.

Section Content: Humira's Warnings and Precautions section includes detailed descriptions of the risks associated with use, including serious infections such as tuberculosis (TB), invasive fungal infections, and opportunistic pathogens; malignancies including lymphoma and hepatosplenic T-cell lymphoma; hypersensitivity reactions; neurologic reactions such as demyelinating disease; hematological reactions including cytopenias; and hepatotoxicity. The section also highlights risk of heart failure, lupus-like syndrome, reactivation of hepatitis B virus (HBV), and considerations around live vaccines. Clinicians are advised to screen and monitor patients accordingly before and during treatment. Recommendations for managing adverse effects and discontinuing treatment under certain conditions are also presented.

Compliance Evaluation:

General Principles: The section follows FDA guidance by prioritizing clinically significant adverse events and outlining steps for monitoring, mitigation, and patient selection. Risks are ordered by severity and potential frequency, and appropriate emphasis is given to boxed warning topics.

Content Requirements: Serious infections and malignancy risks are fully detailed, including mechanisms of immune suppression and recommendations for TB and HBV screening. Each warning includes supporting clinical context or action plans. Specific diseases (e.g., HBV, demyelinating disease) are named, and patient management steps are described. The section also covers risk to special populations and contraindicated scenarios.

Format Requirements: The formatting is consistent with CFR 201.57(c)(6), using numbered subsections with bolded headings and paragraph text for elaboration. Each warning uses direct, actionable language and maintains consistency in scope and style throughout.

Missing or Inconsistent Components:

Missing: No significant omissions were noted. All high-priority risks are addressed, and the boxed warning aligns with detailed warnings below.

Incomplete: The rationale for risk stratification in immunocompromised patients is referenced but could benefit from clearer subgroup differentiation (e.g., pediatric vs. elderly patients) within infection-related warnings.

Misaligned: Although precautions related to vaccines are included, the guidance regarding live vaccines could be more explicitly linked to timing and interaction with immunosuppressive states, which FDA often recommends for clarity

GPT-4o OUTPUTS
WARNINGS AND PRECAUTIONS QUERY 3

Q. 1. List the Warnings and Precautions from "Warnings and Precautions" section in "Zoloft.pdf". 2. Evaluate whether this section complies with the expectations outlined in the relevant FDA guidance documents. Specifically, assess whether the general principles, content, and format requirements are clearly stated. 3. Identify any missing or inconsistent components relative to the requirements outlined in the relevant guidance documents. Specify which components are absent, incomplete, or misaligned, and reference the applicable sections of the guidance documents where appropriate.

Section Content: The Warnings and Precautions section for Zoloft includes important clinical risks such as suicidal thoughts and behaviors in children, adolescents, and young adults; serotonin syndrome when used alone or with other serotonergic drugs; increased risk of bleeding when used with NSAIDs or other anticoagulants; and activation of mania/hypomania in patients with bipolar disorder. It also discusses the risk of seizures, angle-closure glaucoma, hyponatremia—particularly in elderly patients—and withdrawal symptoms upon abrupt discontinuation. Patients are advised to be closely monitored, especially during dose initiation and adjustments.

Compliance Evaluation:

General Principles: The section addresses major, life-threatening risks and adverse reactions that require clinician awareness, aligning with 21 CFR 201.57(c)(6). It prioritizes boxed warning topics like suicidality, and includes pathophysiological rationale for each risk. Monitoring recommendations and intervention strategies are included.

Content Requirements: Each warning provides clinically actionable content, such as identifying high-risk groups (e.g., pediatric patients for suicidality, elderly patients for hyponatremia), and outlining appropriate response actions. Known SSRI class effects, including serotonin syndrome and bleeding risk, are explicitly described. Contraindications are reinforced with discussion of downstream risks (e.g., serotonin syndrome with MAOIs). Discontinuation syndrome is covered with tapering guidance.

Format Requirements: The section uses numbered and titled subsections (e.g., 5.1, 5.2) with bold headers, consistent with FDA formatting expectations. Each item begins with a clear topic header followed by narrative elaboration, and terms like “monitor,” “discontinue,” or “advise patients” are consistently applied for directive clarity.

Missing or Inconsistent Components:

Missing: There is no specific mention of metabolic effects (e.g., weight gain, lipid profiles), which are sometimes included with SSRI class drugs if applicable—even if rare.

Incomplete: The recommendation for seizure history screening is present, but lacks granularity regarding when to initiate alternative therapies or dosage modifications in patients with lower seizure thresholds.

Misaligned: Although withdrawal symptoms are addressed, the label could more clearly emphasize the importance of gradual tapering over weeks, especially for prescribers unfamiliar with SSRI discontinuation protocols.

GPT-4o OUTPUTS
CLINCAL TRIAL PROTOCOL QUERY 1

Evaluate the statistical analysis plan of the clinical trial protocol based on regulatory standards.

To evaluate the **Statistical Analysis Plan (SAP)** of the GSK3036656 Phase IIa clinical trial protocol against **regulatory standards** (e.g. ICH E9, FDA, EMA), we assess it in terms of **completeness, methodological rigor, transparency, regulatory compliance**, and **fitness-for-purpose**.

✅ Summary Evaluation: SAP for GSK3036656 in TB

Trial Type: Phase IIa, open-label, dose-escalation

Study Drug: GSK3036656

Primary Endpoint: EBA CFU0–14 (log10 CFU/mL from sputum)

SAP Document: Reporting and Analysis Plan for Protocol TMF-13331420 SAP_001

---

1. Clarity and Structure

✔ Strengths

- SAP is **well-structured**, covering:
  - Planned analyses (interim/final)
  - Populations
  - Endpoints
  - Covariates
  - Handling of missing data
  - Analysis windows
- Version control and amendment history clearly presented SAP_001 .

🛠️ Areas for Improvement

- Cross-linking of protocol and SAP could be more explicit (e.g. section numbers).
- A "statistical analysis objectives" section could be included up-front for clarity.

---

2. Primary Endpoint Analysis

✔ Method Used

- **Mixed Model with Repeated Measures (MMRM)** is specified for:
  - Primary: EBA CFU0–14
  - Secondary: EBA CFU0–2, CFU2–14, TTP variants
- Covariates:
  - **Treatment**, **Day**, **BMI**, **Treatment-by-Day interaction**
- Includes **SoC** comparator data in model to improve estimate precision SAP_001 .

✔ Regulatory Alignment

- MMRM is **endorsed by EMA, FDA, and ICH E9** as an appropriate method for longitudinal continuous data (such as CFU counts).
- Including baseline covariates and interaction terms complies with **ICH E9 guidance on covariate adjustment**.
- Justified use of **Bayesian bi-linear regression** as a **supportive analysis** is also aligned with EMA's acceptance of Bayesian methods in early-phase TB studies SAP_001 .

---

3. Population Definitions & Protocol Deviations

✔ Definitions Present

- Clear analysis populations:
  - Intent-to-Treat (ITT)
  - Per Protocol
  - Safety
- Protocol deviation handling explained with deviation classification system SAP_001 .

✔ Regulatory Alignment

- Follows **ICH E9** recommendations for handling protocol deviations and specifying pre-defined populations.

---

4. Missing Data Handling

✔ Description Found

- Missing data handling covered in **Appendix 7**:
  - Missing/partial dates
  - Early withdrawal handling
- Imputation rules and conservative approaches are stated SAP_001 .

✔ Regulatory Alignment

- Follows **EMA Reflection Paper** on missing data and **FDA Guidance** requiring a transparent and pre-specified approach.

---

5. Multiplicity and Type I Error Control

⚠ Partial Coverage

- No formal adjustment for multiplicity is described (e.g., alpha spending or hierarchical testing).
- This may be acceptable for **early-phase exploratory trials**, but should be explicitly

acknowledged.

🛠️ Recommendation

- Add a justification for lack of Type I error control, or include a statement on the exploratory nature of multiple secondary endpoints.

---

6. Interim Analysis Plan

✔ Limited Interim Analyses

- No formal interim analysis planned; however:
  - Data reviewed for **dose escalation decisions**
  - Includes safety, PK, and possibly efficacy review
- Not intended for inferential testing

✔ Regulatory Fit

- Fits **early-phase, adaptive designs**.
- Consistent with FDA's "Guidance on Adaptive Designs for Clinical Trials".

---

7. Safety and PK Analyses

✔ Adequate Safety Sections

- Standard analyses for:
  - AEs
  - Laboratory parameters
  - ECGs
- **COVID-19 impact** on safety explicitly addressed SAP_001 .

✔ Pharmacokinetics

- PK parameters: **Cmax, AUC, Tmax**, analyzed using standard descriptive and inferential methods.
- Data sufficiency: PK from ≥7 participants justified based on prior variability.

✔ Regulatory Alignment

- Compliant with **ICH E3 and E9**, and **FDA/EMA PK guidance**.

---

8. COVID-19 Considerations

✔ Dedicated Section

- Additional analyses to assess pandemic's impact on:
  - Safety
  - Protocol deviations
  - Study population enrollment
- Adds robustness and transparency SAP_001 .

✅ Final Verdict: Compliant with Regulatory Standards

Criterion	Evaluation
Clarity and Structure	✅ Clear and comprehensive
Primary/Secondary Analyses	✅ Appropriate models and covariates
Missing Data Handling	✅ Transparent and pre-specified
Safety and PK	✅ In-depth and regulatory-aligned
Dose Escalation Analyses	✅ Fit for early-phase design
Multiplicity Control	⚠ Needs clarification or justification
Interim Analyses	✅ Exploratory, not inferential
COVID-19 Impact	✅ Addressed explicitly

📌 Recommendations

- **Clarify multiplicity handling** (even if not adjusted).
- Consider a short summary section on **statistical analysis objectives**.
- Include a justification for the sample size recalculation within the SAP directly, referencing observed variability.

Let me know if you'd like this formatted as a regulatory checklist or compared to a specific ICH/FDA guidance document.

Sources

Do you like this personality?

ChatGPT 4o

Share ...

GPT-4o OUTPUTS
CLINCAL TRIAL PROTOCOL QUERY 2

SAP_001.pdf
PDF

Prot_000.pdf
PDF

Extract and summarize the clinical trial protocol and statistical analysis plan of the given clinical trial.

Here is a detailed summary of the **clinical trial protocol** and **statistical analysis plan** for the Phase IIa study investigating the drug **GSK3036656** in **drug-sensitive pulmonary tuberculosis (DS-TB)** participants.

📄 Clinical Trial Protocol Summary

Source: Protocol Amendment 05 Prot_000

Title

A Phase IIa open-label trial to investigate the early bactericidal activity, safety and tolerability of GSK3036656.

Objectives and Endpoints

Primary Objective

- To evaluate the **early bactericidal activity (EBA)** of GSK3036656 over **14 days** of once-daily dosing.
- **Primary Endpoint**: Change in **log10 CFU/mL** (colony-forming units) in respiratory sputum from **baseline to Day 14 (EBA CFU0–14)**.

Secondary Objectives

- Assess **safety and tolerability**.

- Evaluate **pharmacokinetics (PK)** of GSK3036656.
- Characterize **EBA** over early (Day 0–2) and late (Day 2–14) intervals.

Secondary Endpoints

- Adverse events (AEs), laboratory values, ECGs, vitals.
- PK parameters: **AUC, Cmax, Tmax**.
- EBA measured via **log10 CFU/mL** and **Time to positivity (TTP)** metrics.

Exploratory

- Additional bacterial endpoints.
- Baseline for bacterial load: Mean of Day –2 and Day –1 sputum (if both available).

Study Design

- **Single-centre, open-label**, randomized (3:1) to:
  - **GSK3036656** (intervention)
  - **Standard-of-care (SoC)**: Rifafour® e-275 or generic equivalent.
- **Dose & Duration**:
  - 5 cohorts with **12–20 participants** each (total n ≈ 100).
  - **15mg loading dose Day 1**, **5mg maintenance Days 2–14**.
  - Follow-up 14 days post-treatment.
- **Dose escalation** based on safety, PK, and available efficacy data. Adjustments allowed (e.g., lowering future doses if needed).
- Participants screened for 7 days, dosed 14 days, followed for 14 more = ~5 weeks total duration per participant.
- **Eligibility refinements**:
  - Adjusted **haemoglobin cutoff criteria** to accommodate population-specific characteristics (e.g., allowed <10g/dL under DEC discretion).

Sample Size Consideration

- Sample size **re-estimated** due to:
  - Lower than expected variability in log10 CFU reduction.

    - Slower participant recruitment.
- Based on updated statistical power requirements Prot_000 .

📊 Statistical Analysis Plan (SAP) Summary

Source: RAP Amendment SAP_001

Purpose of the SAP

- Describes **planned analyses** for the **Clinical Study Report (CSR)** under Protocol TMF-13331420.
- Includes **interim** and **final analyses**, dose escalation support, and **COVID-19 pandemic impact** considerations.

Analysis Populations

- **Efficacy**, **Safety**, and **PK** populations are clearly defined.
- Protocol deviations and their impact on population assignments are detailed.

Efficacy Analyses

Primary Analysis

- **Endpoint**: EBA CFU0–14.
- **Method**: **Mixed model with repeated measures (MMRM)**.
    - Covariates: **Treatment**, **Day**, **BMI**, and **Treatment-by-Day interaction**.
    - Data from **SoC** group included to improve inference.
- A **Bayesian bi-linear regression model** is added as a **supportive** method—important in TB studies where response is often non-linear SAP_001 .

Secondary Analyses

- Similar modeling for:
    - EBA CFU0–2
    - EBA CFU2–14

- TTP0–14, TTP0–2, TTP2–14

Safety Analyses

- Include:
  - **Adverse events**
  - **Clinical labs**
  - **Vital signs**
  - **ECG evaluations**
- Analysis of safety during **COVID-19** pandemic is outlined, including the categorization of AEs based on the pandemic phase SAP_001 .

Pharmacokinetic Analyses

- PK endpoints:
  - **AUC0–t, AUC0–24, Cmax, Tmax**
- **Subgroup analyses** and covariate considerations based on **BMI**, **treatment day**, and dose exposure.
- **PK data** from 7+ participants per cohort considered sufficient due to observed variability.

COVID-19-Specific Analyses

- Impact assessments:
  - On **study conduct**, **safety**, and **efficacy**.
  - Specific analysis tables and categorization windows defined.

📌 Key Amendments and Rationale

1. **Reduced sample size** per cohort (from fixed 20 to range 12–20) to account for lower variability.
2. Inclusion of **SoC comparator group** in modeling.

3. Addition of **BMI as a covariate** in efficacy analyses.
4. Modified **eligibility/stopping criteria** around haemoglobin levels.
5. Specific updates to manage **COVID-19 impact** on data and outcomes.

✅ Summary Conclusion

The trial is a well-designed **early-phase study** aiming to determine the **efficacy**, **safety**, and **PK** of GSK3036656 in DS-TB patients. It utilizes adaptive design elements (like dose escalation based on safety/PK) and incorporates robust statistical methodology including **MMRM** and **Bayesian modeling**. Flexibility is built in to handle **real-world challenges** (e.g., COVID-19 disruptions and local population haemoglobin norms).

Let me know if you'd like a side-by-side comparison with a guidance document or a tailored checklist-based evaluation.

Sources
